# Catalytic Enantioselective Perezone-Type [5 + 2] Cycloaddition

**DOI:** 10.1021/jacs.5c14484

**Published:** 2025-10-17

**Authors:** Liangliang Yang, Chaoshen Zhang, Jianwei Sun

**Affiliations:** Department of Chemistry and the Hong Kong Branch of Chinese National Engineering Research Centre for Tissue Restoration & Reconstruction, 58207 The Hong Kong University of Science and Technology, Clear Water Bay, Kowloon, Hong Kong SAR 999077, China

## Abstract

Here we report the first catalytic enantioselective Perezone-type [5 + 2] cycloaddition, enabling convenient access to a variety of bicyclo[3.2.1]­octanes and other complex carbocycles from readily available quinone ketals and alkenes. Enabled by a new chiral squaramide catalyst, intermolecular C–C bond formation was achieved with excellent enantio- and diastereoselectivities. The highly enantioenriched and densely functionalized products can be transformed into diverse intriguing complex carbocycles that may not be straightforward to access by conventional methods. Mechanistic studies and DFT studies provided important insights into the mechanism, in which substrate solvation is a rate-limiting step, while C–C bond formation is fast and stepwise. Activation of the Lewis acid TESOTf by the chiral hydrogen bond donor catalyst is crucial not only for substrate activation and solvation but also for enantiocontrol via the catalyst-bound chiral counter anion.

## Graphical abstract

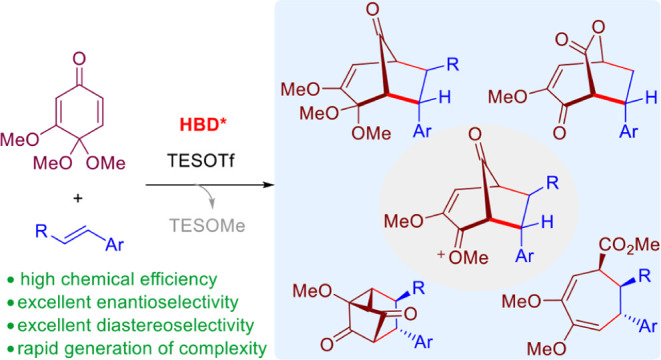

## Introduction

Bicyclo­[3.2.1]­octane is a privileged skeleton in natural product and medicinal chemistry.^1^ Specifically, when bearing a carbonyl (or its reduced alcohol) bridge, this type of framework is the core of numerous complex natural products and bioactive molecules (Scheme 1A).^1,2^ Such bridged structures have also served as valuable synthetic precursors to various other intriguing bicyclic scaffolds and densely functionalized seven-membered carbocycles.^1,2^ As a result, in the past few decades, enormous efforts have been devoted to developing efficient strategies for the construction of these structures, many of which require a cascade entailing multiple steps for sequential C–C bond formations.^3–9^

**Scheme 1:**
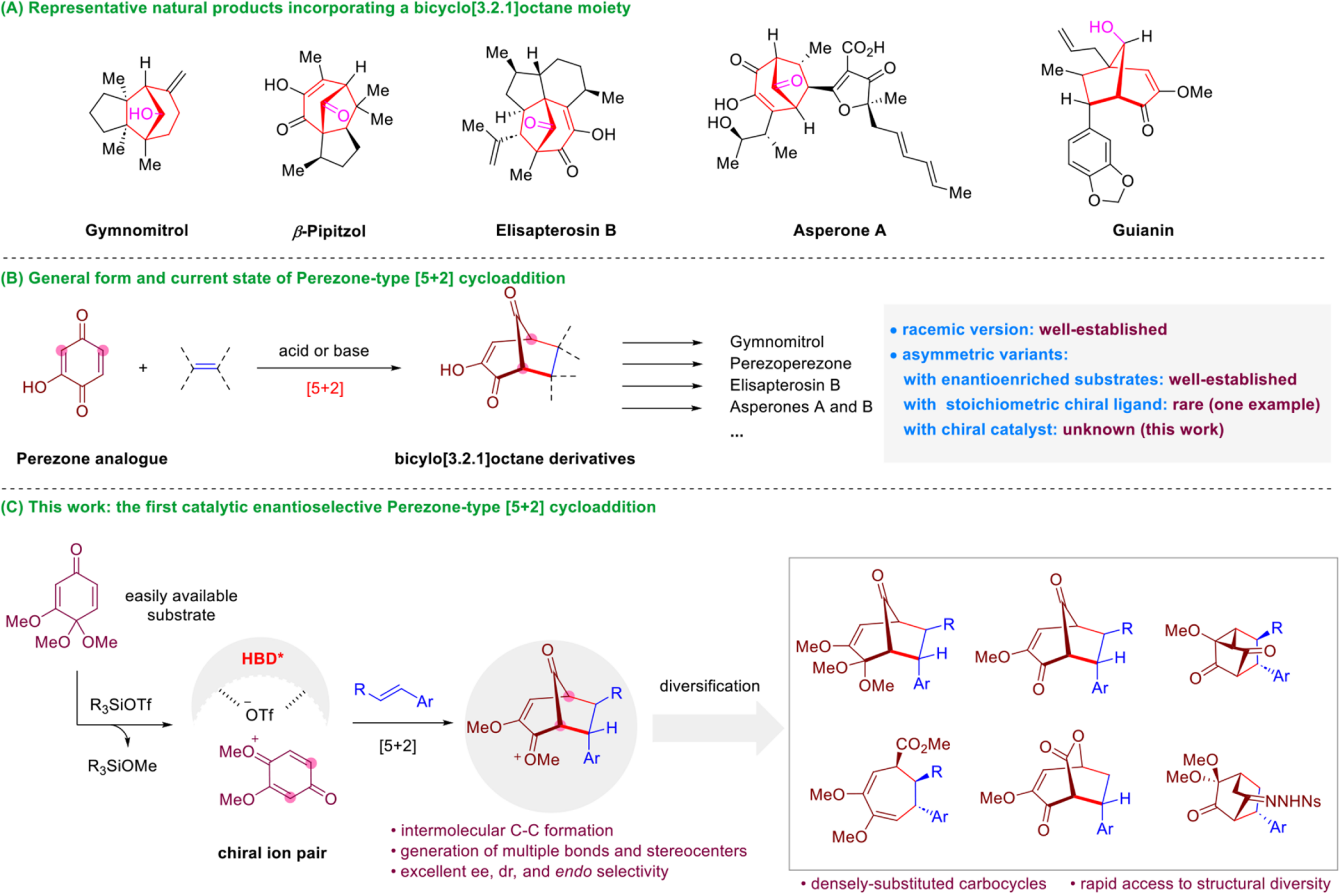
Introduction to Perezone-Type [5 + 2] Cycloaddition and Reaction Design

Among those strategies, the Perezone-type [5 + 2] cycloaddition provides rapid access to the above-mentioned bicyclo[3.2.1]­octane ring system with a carbonyl bridge by the concomitant formation of two C–C bonds between an olefin and the two α positions of the carbonyl group in readily available Perezone analogues (Scheme 1B).^3–7,10^ In fact, this powerful process can be dated back to 1885 since the pioneering report by Anschutz and Leather.^3^ In the past one and half century or so, it has been widely employed as a strategic step in the syntheses of diverse complex natural products.^4–7^

However, despite its long history and widespread applications, there remains an unmet challenge. So far, the asymmetric variants of this important transformation have been limited to the use of enantioenriched precursors or stoichiometric chiral promoters (Scheme 1B).^5,6^ Unfortunately, an efficient enantioselective variant with chirality induced by a small amount of a chiral catalyst remains unknown. In this context, herein we report the first highly efficient catalytic enantioselective protocol for this process, enabled by a chiral hydrogen-bonding-donor (HBD) catalyst. High enantio-, diastereo-, and *endo-*selectivities have been achieved under mild conditions when generating up to four consecutive stereogenic centers, thus enabling expedient access to diverse valuable complex carbocycles (Scheme 1C).

## Results and Discussion

Our investigation began by employing quinone monoketal **1a** and styrene **2a** as model substrates. Initially, chiral phosphoric acid (*R*)-**CPA1** was used as a catalyst, but the reaction did not occur and **1a** was completely recovered (Table 1, entry 1).^11^ We reasoned that this may require a stronger acid to activate the ketal substrate. Next, we first tested a strong achiral acid, HOTf. As expected, the reaction occurred, but the product was not the desired cycloadduct bicyclo[3.2.1]­octane **4a** or **4a′**. Instead, seven-membered-ring structure **5a** was observed (entry 2). We hypothesized that the methanol generated from the activation of the ketal by HOTf likely promoted the cleavage of the ketone bridge in compound **4a**, which resulted in the formation of compound **5a**. Next, we employed TESOTf as the promoter to avoid methanol formation, which successfully yielded cycloadduct **4a**, albeit in low yield (entry 3). Lowering the reaction temperature to −60 °C increased the yield to 60% (entry 4), but the reaction was completely suppressed at −78 °C (entry 5). Inspired by the pioneering study by the Jacobsen laboratory on Lewis acid enhancement by chiral hydrogen-bond donors (HBDs),^12,13^ we examined several representative HBD catalysts for this cycloaddition (entries 6–8). While thiourea **3a** and urea **3b** could not promote this transformation, squaramide **3c** exhibited much higher catalytic activity, forming **4a** in 90% yield and 77:23 enantiomeric ratio (er, entry 8).^14^ Further screening of structurally diverse chiral squaramides revealed that the new structure **3i** provided the highest enantioselectivity (entry 10 and more details in the Supporting Information). Interestingly, when the reaction was quenched with saturated aqueous NaHCO_3_, diketone **4a′** was obtained as the major product with equally good enantioselectivity (entry 11). Moreover, this reaction is extremely sensitive to the solvent. When DCM was used as solvent, a complete loss of enantioselectivity was observed, likely due to the good solubility of **1a** in DCM and thus fast background reaction (entry 12 and more details in the Supporting Information). In addition, the combined use of HOTf and **3i** provided product **5a** in good yield and slightly decreased enantioselectivity (entry 13). Alternatively, when the reaction mixture of entry 10 was quenched by 1 equiv of methanol, **5a** was also formed in good yield and excellent er (entry 14). The product dependence on the quenching method suggested that the direct cycloadduct was probably an oxonium but not the finally observed ketal or ketone (*vide infra*).

**Table 1:**
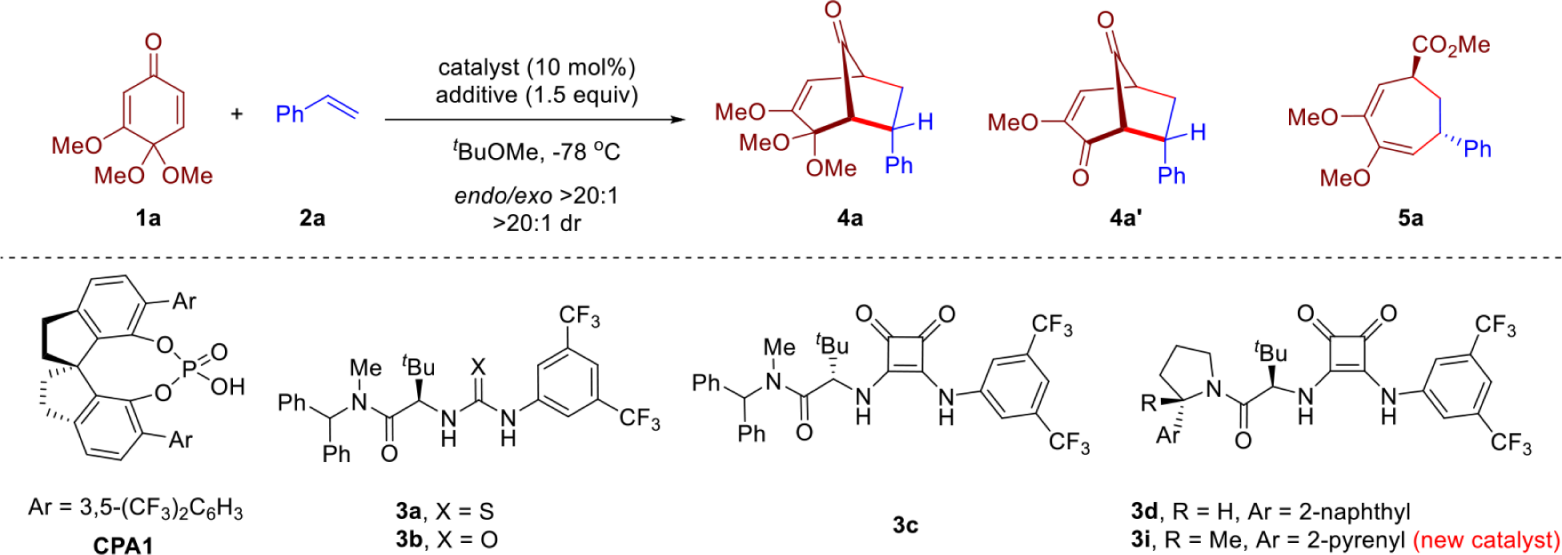
Evaluation of Reaction Conditions^a^

entry	catalyst	additive	yield (%)	er
1^b^	(*R*)-**CPA1**	–	N.R.	–
2^b^	–	HOTf	15 (**5a**)	–
3^b^	–	TESOTf	12	–
4^c^	–	TESOTf	60	–
5	–	TESOTf	trace	–
6	**3a**	TESOTf	trace	–
7	**3b**	TESOTf	trace	–
8	**3c**	TESOTf	90	77:23
9	**3d**	TESOTf	>95	85:15
10	**3i**	TESOTf	>95	95:5
11^d^	**3i**	TESOTf	75 (**4a′**)	95:5
12^e^	**3i**	TESOTf	33	50:50
13	**3i**	HOTf	85 (**5a**)	93:7
14^f^	**3i**	TESOTf	80 (**5a**)	95:5

a Reaction conditions: **1a** (0.05 mmol), **2a** (0.075 mmol), TESOTf (1.5 equiv), catalyst (10 mol %), ^*t*^BuOMe (0.5 mL), 48 h. The reaction was quenched with MeOH/Et_3_N (3:1). Yield was determined by analysis of the ^1^H NMR spectrum of the crude product using CH_2_Br_2_ as an internal standard. Er was determined by HPLC with a chiral stationary phase.

b Run at rt.

c Run at −60 °C.

d Quenched with saturated NaHCO_3_ (aq.).

e Run in CH_2_Cl_2_.

f Quenched with MeOH (1.5 equiv) followed by MeOH/Et_3_N (3:1).

Under the optimized conditions, we investigated the reaction scope (Scheme 2). At a 0.4 mmol scale, the loading of TESOTf can be reduced to 1.2 equiv without compromise in efficiency. Aryl alkenes with substituents of varying steric and electronic properties provided the corresponding products **4a–n′** in high yields and with good enantioselectivities. Notably, terminal olefins exhibited a higher reactivity than internal alkenes and alkynes. For example, high chemoselectivity was observed for the synthesis of bicyclo[3.2.1]­octanes **4o** and **4p′**, in which the internal alkyne and trisubstituted alkene motifs remained intact. Other functionalities, such as ester, ether, aryl halide, and ketone, were also well tolerated under mild conditions. Moreover, a complex biologically active steroid scaffold can also be incorporated into the cycloadduct (**4q′**) structure, indicating the power of this protocol for the facile modification of olefin-containing complex structures including bioactive molecules and natural products. It is worth noting that all of these products were generated essentially as a single diastereomer with *endo* selectivity. Unfortunately, aliphatic olefins (e.g., 1-hexene, vinylcyclohexane) failed to yield the desired products, and only decomposition of the quinone monoketal substrate was observed.

**Scheme 2:**
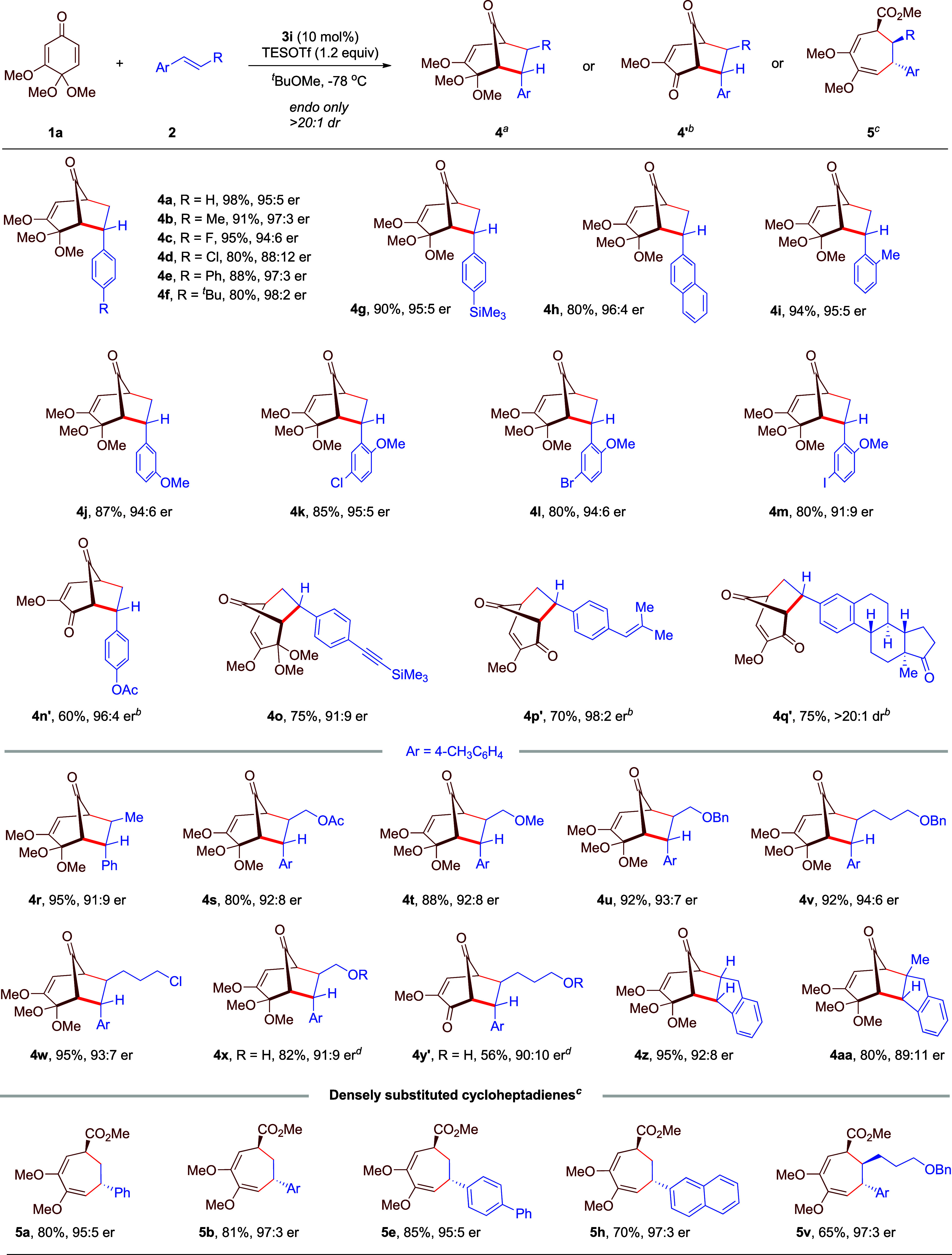
Reaction Scope^a^ ^b^ ^c^ [Fn sch2-fn4]

a Reaction conditions: **1a** (0.4 mmol), **2** (0.48 mmol), TESOTf (1.2 equiv), **3i** (10 mol %), ^*t*^BuOMe (4.0 mL). Isolated yield. Er was determined by HPLC with a chiral stationary phase. Quench with a MeOH/Et_3_N (3:1) solution.
b Quench with saturated aqueous NaHCO_3_.
c Quench with MeOH first and then a MeOH/Et_3_N (3:1) solution.
d
*R* = TBS in the starting olefin, desilylation by TBAF or HCl (aq.) was performed during workup.

Next, we evaluated di- and trisubstituted olefin partners. Under the optimized conditions, a range of 1,2-disubstituted aryl olefins with varying alkyl chain lengths all reacted to form the desired bridged bicyclic products **4r**–**4w** with good efficiency and enantioselectivity. Notably, four new consecutive stereogenic centers were generated in each of these cases. Ester (**4s**), ether (**4t**–**4v**), and halogen (**4w**) functionalities were well tolerated. However, the *tert*-butyldimethylsilyl (TBS) group in the silyl ether functionality was partially transformed into a triethylsilyl (TES) group during the standard conditions (**4x** and **4y′**). Thus, removal of the silyl group by TBAF or HCl (aq.) was performed purposely to simplify purification and characterization. Disubstituted and trisubstituted cyclic olefins also participated in this process, leading to bridged polycyclic products **4z** and **4aa** with high efficiency. Finally, when quenched initially with MeOH, this annulation process can directly provide access to densely substituted cycloheptadienes bearing multiple stereogenic centers, which may not be straightforward to establish by conventional strategies (**5a**, **b**, **e**, **h**, and **v**). It is worth noting that the two versatile enol ether motifs in this 7-membered carbocycle may permit plenty of opportunities for further functionalization.

We further demonstrated the utility and potential applications of the above enantioselective [5 + 2] cycloadditions (Scheme 3). The synthesis of enantiomerically enriched bicyclo[3.2.1]­octane **4b** was performed at 2 mmol scale with virtually no compromise in yield or enantioselectivity. The squaramide catalyst **3i** was also recovered in 96% yield, and the recovered catalyst showed no loss of catalytic activity. Next, diverse transformations of the enantioenriched bicyclo[3.2.1]­octane products were performed. Various nucleophilic additions to the carbonyl bridge, such as reduction (**6**) with NaBH_4_ and addition (**7**) with an allyl Grignard reagent, were easily achieved with excellent diastereoselectivities. The relative stereochemistry of alcohol **7** was confirmed by the NOESY spectrum (see Supporting Information for more details). Moreover, this ketone was also converted to epoxide **8** and olefin **9** under Corey–Chaykovsky and Wittig olefination conditions, respectively. Transformations on the bicyclic skeleton were also investigated. Upon facile hydrolysis of the ketal functionality, enantioenriched bicyclo[3.2.1]­octadienones **4b′** (CCDC 2413151) and **4v′** could be obtained from **4b** and **4v**, respectively. Notably, the two ketone units in **4b′** could be selectively converted. With a vinyl Grignard reagent, only the conjugated ketone unit reacted, resulting in tertiary alcohol **10** with high efficiency and diastereoselectivity. In contrast, chemoselective transformation on the bridge carbonyl could also be achieved. For example, under the Baeyer–Villiger oxidation conditions with *m*-CPBA, **4b′** was converted exclusively to the lactone **11**, whose structure was unambiguously confirmed by X-ray crystallography (CCDC 2413152). More intriguingly, photoirradiation of **4b′** and **4v′** produced different types of skeletons, tricyclo­[3.2.1.0 ^2,7^ ]­octane structures **12** and **13**, respectively, as single diastereomers. Mechanistically, it is believed that diradical intermediates might be involved in these skeletal reorganizations.^4i^ The highly strained donor–acceptor cyclopropane motif in **12** could easily undergo nucleophilic ring-opening. Upon treatment with MeOH and NsNHNH_2_, bicyclo[2.2.2]­octane **14** bearing a hydrazone motif was obtained. Its structure was unambiguously confirmed by X-ray crystallography (CCDC 2413153). Finally, the structure of cycloheptadiene **5h** was also determined by X-ray crystallography (CCDC 2413154) of its derivative **16**, after sequential reduction and protection.

**Scheme 3:**
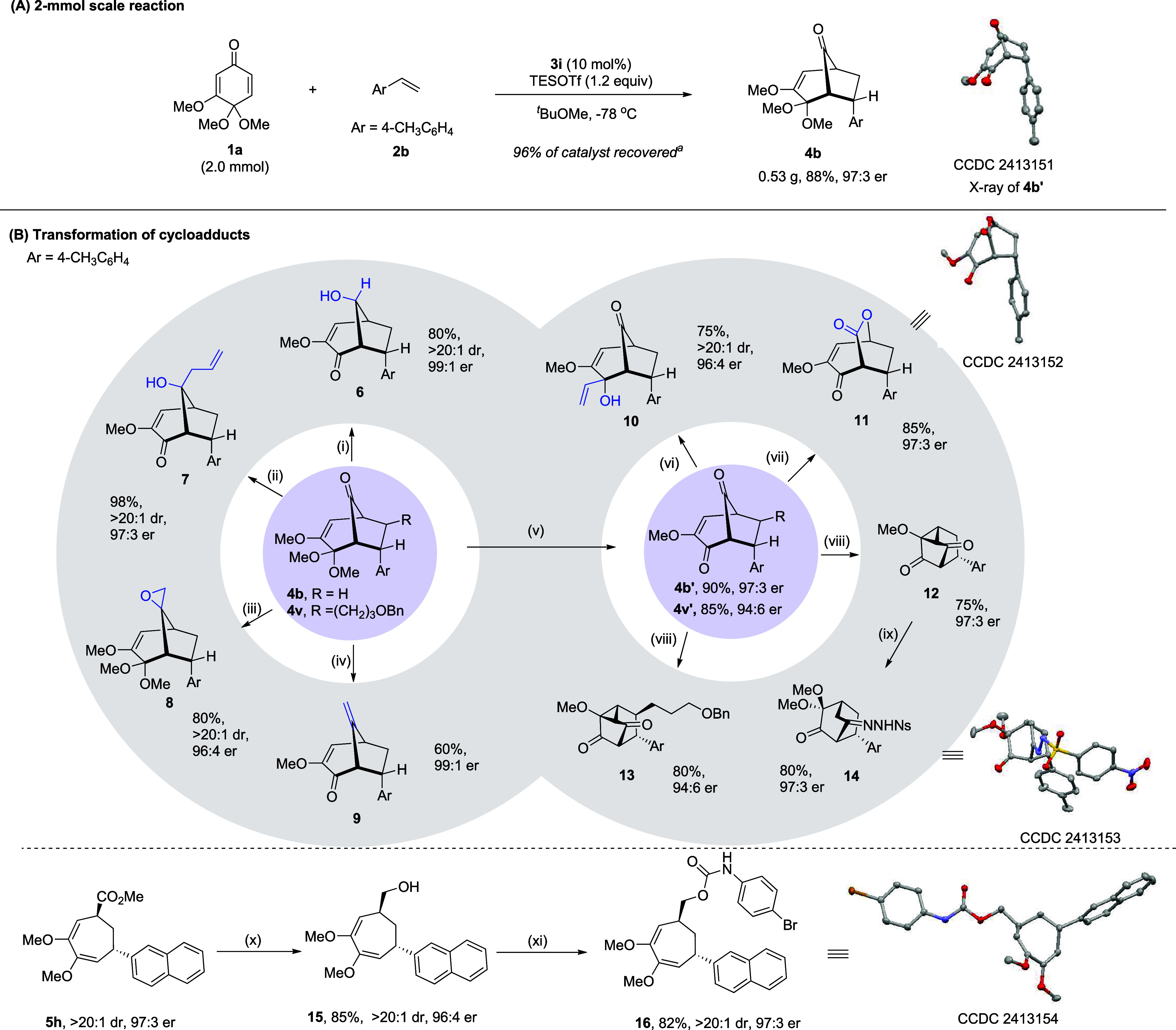
Scale-up Reaction and Product Transformations^a^

a The recovered catalyst **3i** showed similarly good catalytic activity (88% yield, 96:4 er). Reaction conditions: (i) NaBH_4_, MeOH; HCl (1N), acetone. (ii) Allylmagnesium bromide, THF; HCl (1N), acetone. (iii) Me_3_SO^+^I^–^, NaH, DMSO. (iv) Ph_3_MeP^+^I^–^, ^*n*^BuLi, THF; HCl (1N), acetone. (v) HCl (1N), acetone. (vi) Vinylmagnesium bromide, THF. (vii) *m*-CPBA, CH_2_Cl_2_. (viii) LED (390 nm), MeCN. (ix) NsNHNH_2_, MeOH, rt. (x) Red-Al, THF, 0 °C. (xi) 1-Bromo-4-isocyanatobenzene, Et_3_N, CH_2_Cl_2_, rt.

Next, we conducted a series of experiments to help us understand the mechanism (Figure 1). It is worth noting that substrate **1a** has a low solubility in ^*t*^BuOMe and thus, this reaction is heterogeneous. In fact, this feature is crucial to the observed high enantioselectivity since the background reaction is effectively suppressed by a low concentration of **1a** and thus a high catalyst/**1a** ratio in solution. Notably, substrate **1b** (*R* = Et) is completely soluble in ^*t*^BuOMe, resulting in a homogeneous reaction (Figure 1). However, in sharp contrast, almost completely lost enantiocontrol was observed in the reaction of **1b** (58:42 er, entry 1, Figure 1A-a). Nevertheless, increasing the catalyst loading to 50 and 100 mol % recovered high enantioselectivity. We also monitored the progress of the reactions of **1a** and **1b** separately, with or without catalyst **3i** (Figure 1A-b). In the absence of a catalyst, almost no conversion of **1a** was observed. However, obvious rate acceleration was observed when the catalyst was added. We believe that catalyst activation of **1a** enhanced its solubility. In contrast, the reaction of **1b** proceeded steadily in the absence of a catalyst, and little acceleration was observed when the catalyst was added, suggesting a strong background reaction in this case. All these results are consistent with the above rationale about the background reaction and the influence of solubility.

**Figure 1:**
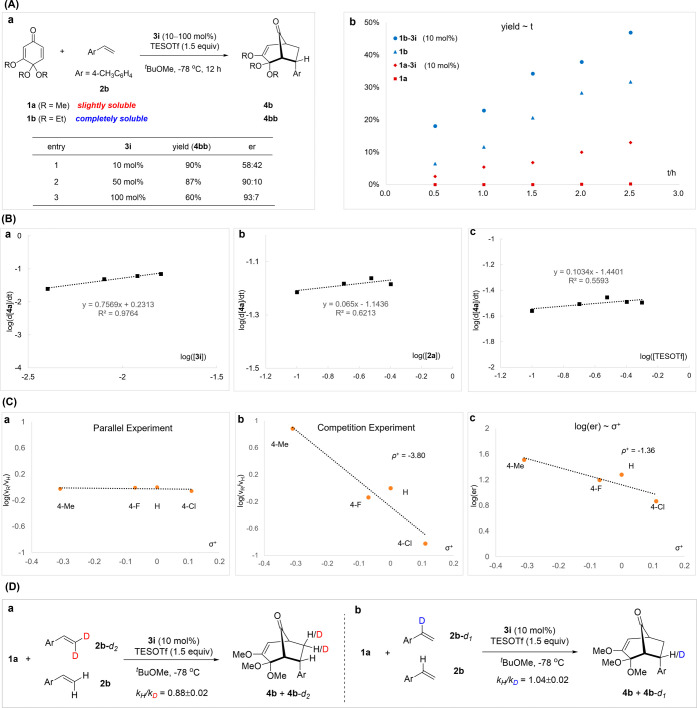
Mechanistic studies. (A) (a) The reaction outcome of **1b** at different loadings of **3i**. (b) Reaction progress of **1a** and **1b** in the presence or absence of **3i**. (B) Kinetic profiles of the standard reaction of **1a** and **2a**. (C) The Hammett plot between relative rates and σ^+^ values with **2a**–**2d** in (a) parallel reactions and (b) a one-pot competition reaction. (c) The influence of σ^+^ values with **2a**–**2d** on the enantioselectivity (log­(er)). (D) “Kinetic isotope effect” determined by competition experiments.

We also examined the kinetic profile of the model reactions of **1a** and **2a**. The reaction order in squaramide **3i** was around 0.75.^15a^ Furthermore, the reaction showed almost zeroth order in both TESOTf and **2a**. These results suggested that (1) the intermolecular C–C bond formation step with **2a** is a fast step; (2) The initial activation of catalyst **3i** by TESOTf is probably favorable by formation of a thermodynamically more stable adduct, thus **3i** is saturated by TESOTf; (3) The rate-determining step is perhaps the activation of **1a** to form the key oxonium intermediate or the dissolution of **1a** (phase transfer).

To gain more insight into the rate- and enantiodetermining steps, investigations on Hammett correlation were performed using different *p*-substituted styrenes **2a**–**2d**. Interestingly, when reaction rates were measured separately in parallel, essentially no influence of substituent constants σ^+^ on the reaction rates was observed (Figure 1C-a). This result is consistent with the above analysis that the rate-determining step is prior to the involvement of the olefin partner, but not the C–C bond formation. In contrast, when this comparison was performed in a one-pot competition reaction, a linear correlation with a large negative slope (ρ^+^ = −3.80) was observed between the relative rates and substituent constants σ^+^ (Figure 1C-b). This result suggested that the accumulation of positive charge might be involved in the bond formation of the styrene partner, thus the cycloaddition is likely a stepwise process. Upon the first C–C bond formation, a benzylic carbocation is generated. Moreover, a linear correlation with a moderate negative slope (ρ^+^ = −1.36) was also observed between the log­(er) and σ^+^ values, in agreement with the fact that this C–C bond-forming step is an irreversible and enantiodetermining step. To gain further evidence, we also studied the kinetic isotope effect with deuterated forms of styrene **2b**. With deuterium incorporation at the terminal position (**2b**-*d*_2_), the competition with **2b** indicated an “inverse secondary isotope effect” (*k*_H_/*k*_D_ = 0.88), implying a possible change of hybridization from *sp* ^2^ to *sp* ^3^ in the reacting carbon atom.^15b^ However, with deuterium in the internal position (**2b**-*d*_1_), this isotope effect was negligible (*k*_H_/*k*_D_ = 1.04). These observations are consistent with the stepwise but not concerted cycloaddition event.

A possible reaction mechanism is proposed based on the above results and related literature,^14^ together with preliminary DFT studies (Figure 2A and more details in the Supporting Information). The reaction begins with a thermodynamically favorable interaction between squaramide catalyst **3i** and TESOTf, forming an activated Lewis acid **IM1**. Subsequent activation of the ketal substrate **1a** by **IM1** triggers the dissociation of a methoxy group to generate the key oxonium ion pair **IM2**. This also drags the insoluble substrate into solution, which might be the rate-determining step based on the kinetic results. Next, the olefin partner approaches **IM2** to form a relatively stable adduct **IM3** due to the favorable π interaction. This also sets the stage for stereocontrolled intermolecular C–C bond formation in a stepwise manner via carbocation **IM4**. The resulting chiral bicyclo[3.2.1]­octane oxonium intermediate **IM5** then rapidly reacts with TESOTf to regenerate the turnover catalyst **IM1**. Meanwhile, the immediate product **IM6** can be diversified into different observed products **4a** or **5a** depending on the quenching method.

**Figure 2:**
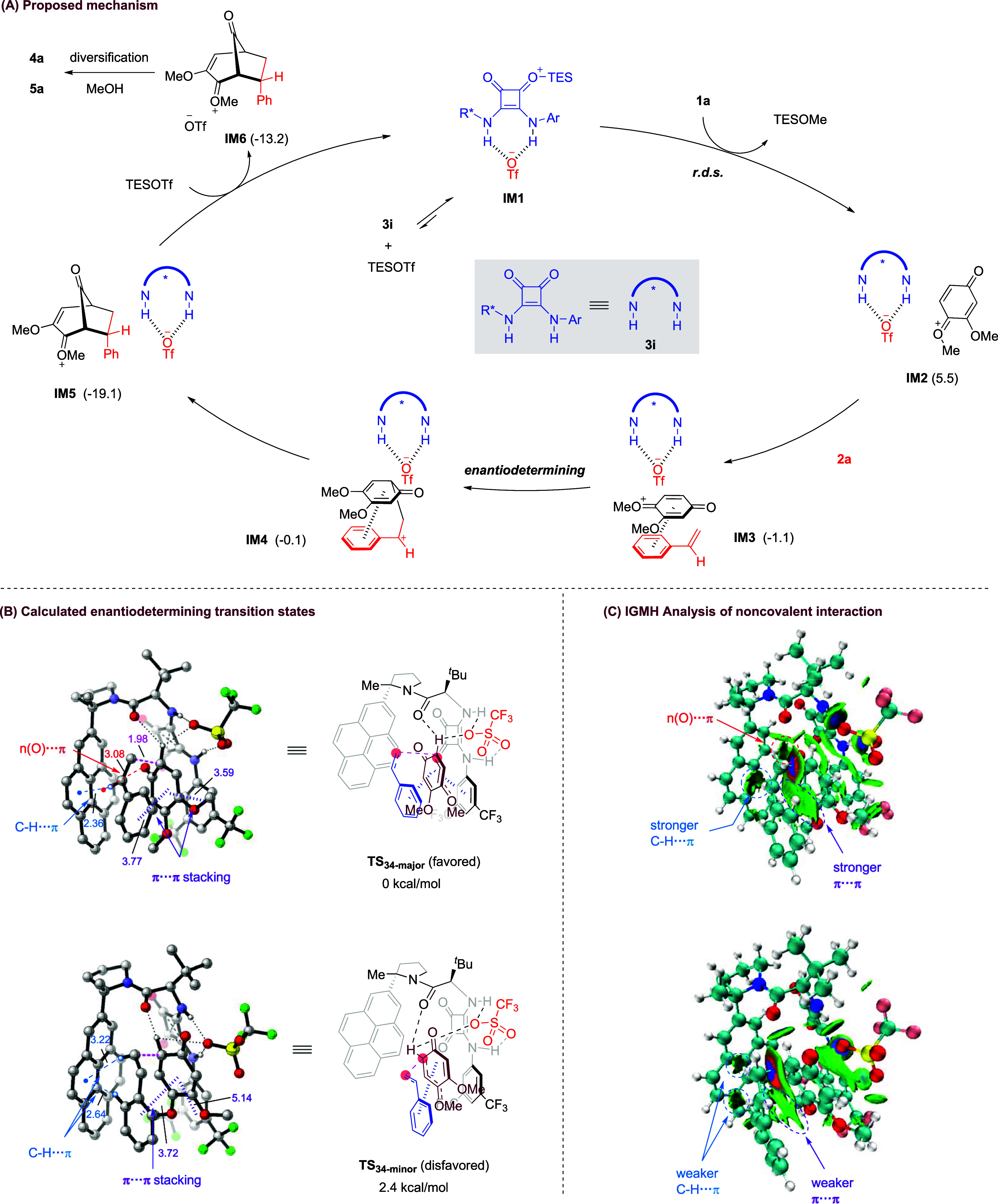
Proposed mechanism and DFT studies. M062X-D3/6-31+G­(d,p)//B3LYP-D3BJ/6-31G­(d) level of theory for all atoms. Relative free energy is given in kcal/mol. The marked distances are given in ångströms. All molecular structures were rendered in CYLView20.

The origins of enantioselectivity were also investigated by comparing the lowest-energy conformers calculated for the enantiodetermining transition states (**TS**_**34‑major**_ and **TS**_**34 minor**_) connecting **IM3** to **IM4**, as illustrated in Figure 2B (see also the Supporting Information). M062X-D3/6-31+G­(d,p) single-point calculations indicated that the reaction barrier difference leading to the two product enantiomers (ΔΔ*G*‡ = 2.4 kcal/mol) is consistent with the experimental outcome. Both transition states feature a hydrogen-bonding network involving the pentadienyl cation, triflate counterion, and catalyst amide backbone. However, weak interaction analysis (Independent Gradient Model based on Hirshfeld partition, IGMH)^16,17^ revealed that the favorable π···π stacking, C–H···π, and *n*(O)···π interactions are stronger in **TS**_**34‑major**_ than those in **TS**_**34 minor**_ (Figure 2B,C), suggesting that enhanced noncovalent interactions are crucial in governing enantioselectivity. Furthermore, π···π stacking between the pentadienyl cation and the olefin partner constitutes the key factor for the preference of *endo* product formation (Figures S1 and S2).

## Conclusion

We have developed the first efficient catalytic enantioselective perezone-type [5 + 2] cycloaddition, a powerful process with long history and broad application but lacking an effective enantioselective protocol. Enabled by the combined use of a new chiral hydrogen bond donor catalyst and a Lewis acid additive, our protocol allowed not only extraordinary bond formation efficiency but also high enantio- and diastereoselectivities in the expedient construction of complex densely functionalized bicyclo[3.2.1]­octanes from readily available quinone ketals and olefins. This protocol also features mild conditions and good functional group tolerance. Different quenching methods and postreaction derivatizations also enabled the assembly of diverse other valuable skeletons with high selectivity. Control experiments and kinetic (isotope) studies provided important insights into the mechanism. Activation of the Lewis acid TESOTf by the chiral squaramide catalyst proved crucial not only to substrate activation and solvation but also to the enantioselective C–C bond formation induced by the well-organized catalyst-bound chiral counteranion. The proper choice of this catalytic system and a suitable solvent was equally essential to the effective inhibition of the background reaction. Notably, substrate solvation is likely the rate-determining step, and C–C bond formation is fast and stepwise. DFT studies further supported the proposed mechanism and provided insights into the origins of the *endo* selectivity and enantioselectivity owing to multiple weak interactions, including π–π stacking and hydrogen bonds.

## Supplementary Material



## Notes

Additional experimental and computational details, and spectroscopic data of all compounds (PDF)
