# Supplementary material for: Catalytic Enantioselective Perezone-Type [5 + 2] Cycloaddition
Source: J Am Chem Soc. 2025 Oct 17;147(43):39860–9. doi: 10.1021/jacs.5c14484 (PMC12576829; doi:10.1021/jacs.5c14484)
Supplement: Supplementary file 1 [file ja5c14484_si_001.pdf]

Supporting Information

Catalytic Enantioselective Perezone-Type [5+2] Cycloaddition

Liangliang Yang, Chaoshen Zhang, and Jianwei Sun*

Department of Chemistry and the Hong Kong Branch of Chinese National Engineering Research Centre for Tissue Restoration & Reconstruction, The Hong Kong University of Science and Technology, Hong Kong, China

Table of Contents

I. General Information S-2
II. Substrate Preparation S-3
III. Catalyst Preparation S-5
IV. Condition Optimization S-11
V. Catalytic Enantioselective [5+2] Cycloaddition S-16
VI. Product Derivatizations S-40
VII. Mechanistic Studies S-52
VIII. Determination of Product Structures S-73
IX. DFT Calculations S-82
X. References S-177
NMR Spectra and HPLC Traces

I. General Information

Flash column chromatography was performed over silica gel (200-300 mesh) purchased from Qindao Haiyang Co., China or SiliCycle® Inc., Canada. All air or moisture sensitive reactions were conducted in oven-dried glassware under nitrogen atmosphere using anhydrous solvents. Tetrahydrofuran was distilled from sodium/benzophenone. Anhydrous dimethyl formamide, dichloromethane, methanol, toluene, diethyl ether, acetonitrile, hexane were purified by the Innovative® solvent purification system. Anhydrous solvents were purchased from Sigma-Aldrich®, J&K® and Energy® and used as received. Chemicals were purchased from commercial suppliers such as Sigma-Aldrich®, J&K®, Energy® and so on and used without further purification unless otherwise stated. NMR spectra were recorded with a Bruker AVII, AVIII or NEO 400 at 400 MHz (^{1}H NMR), 101 MHz (^{13}C NMR), and 376 MHz (^{19}F NMR). Chemical shifts (δ values) were reported in ppm down field from internal standard (^{1}H NMR: Me_4Si at 0.00 ppm and ^{13}C NMR: $CDCl_3$ at 77.00 ppm). Data for ^{1}H NMR are recorded as follows: chemical shift (δ, ppm), multiplicity (s = singlet; d = doublet; t = triplet; q = quarter; p = pentet; sept = septet; m = multiplet; br = broad), coupling constant (Hz), integration. The mass spectra were collected from a Waters Xevo G2-XS Tof, with ESI source; or a Waters GCT premier with EI/CI source. Optical rotations were measured on JASCO P-2000 polarimeter or AUTOPOL I Automatic polarimeter with $[\alpha]_D$ values reported in degrees; concentration (c) is in 10 mg/mL. Enantioselectivities were recorded on Agilent HPLC instrument, using a chiral stationary phase column (Daicel CHIRALPAK® AD-3, IA-3, IB N-3, IC-3, ID-3, IN-3, CHIRALCEL® OD-3, OX-3). The chiral HPLC methods were calibrated with the corresponding racemic mixtures.

II. Substrate Preparation

Quinone monoketals **1** were synthesized according to the literature procedure.[1] Alkenes **2a–j**, **2n**, **2r**, **2z**, and **2aa** are commercially available. Alkenes **2k–m**,[2] **2o**,[3] **2p**,[4] **2q**,[5] **2s**,[6] **2t**,[7] **2u**,[8] and **2v**[9] were synthesized according to the literature method. Alkenes **2w**, **2x**, and **2y** were synthesized according to the procedures shown below.

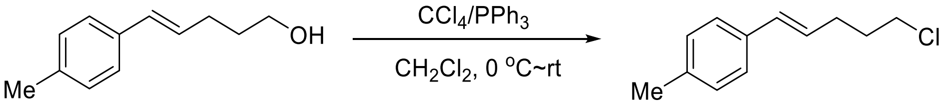

2w

(*E*)-1-(5-Chloropent-1-en-1-yl)-4-methylbenzene (2w). In a nitrogen atmosphere at 0 °C, PPh_3 (1.3 g, 5.0 mmol, 2.0 equiv), dry CH_2Cl_2 (12.5 mL, 0.2 M), and CCl_4 (0.81 g, 5.3 mmol, 2.1 equiv) were added to a 100-mL round-bottom flask. A solution of (*E*)-5-(*p*-tolyl)pent-4- en-1-ol[9] (0.53 g, 3.0 mmol, 1.0 equiv) in dry CH_2Cl_2 (2 mL) was then added dropwise. The reaction mixture was stirred at room temperature for 24 hours. After concentration, the residue was purified by column chromatography (*n*-hexane/EtOAc = 20:1) to yield the pure **2w** as a colorless oil in 75% yield (0.36 g).

^{1}H NMR (400 MHz, $CDCl_3$) δ 7.23 (d, *J* = 8.0 Hz, 2H), 7.10 (d, *J* = 7.9 Hz, 2H), 6.40 (d, *J* = 15.8 Hz, 1H), 6.10 (dt, *J* = 15.8, 7.0 Hz, 1H), 3.57 (t, *J* = 6.6 Hz, 2H), 2.40 – 2.27 (m, 5H), 1.93 (p, *J* = 6.8 Hz, 2H) ppm.

^{13}C NMR (101 MHz, $CDCl_3$) δ 136.8, 134.6, 131.0, 129.2, 127.5, 125.9, 44.3, 32.1, 30.0, 21.1 ppm.

HRMS (CI) Calcd for $C_{12}H_{15}Cl$ $[M]^+$: 194.0862, found: 194.0858.

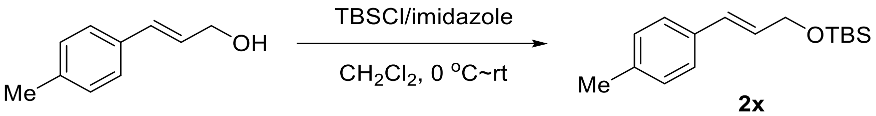

2x

(*E*)-*tert*-Butyldimethyl((3-(*p*-tolyl)allyl)oxy)silane (2x). In a nitrogen atmosphere at 0 °C, TBSCl (0.51 g, 3.4 mmol, 1.3 equiv) and imidazole (0.28 g, 4.1 mmol, 1.5 equiv) were added to a 100-mL oven-dried Schlenk flask containing (*E*)-3-(*p*-tolyl)prop-2-en-1-ol

(0.4 g, 2.7 mmol, 1.0 equiv) and dry CH_2Cl_2 (27 mL, 0.1 M). The reaction mixture was stirred at room temperature for 2 hours. Water was then added to quench the reaction. The mixture was extracted with CH_2Cl_2, and the organic layers were concentrated and purified by column chromatography (*n*-hexane/EtOAc = 20:1) to yield the pure **2x** as a colorless oil in 85% yield (0.6 g).

^{1}H NMR (400 MHz, $CDCl_3$) δ 7.31 (d, *J* = 7.8 Hz, 2H), 7.15 (d, *J* = 7.8 Hz, 2H), 6.59 (d, *J* = 15.8 Hz, 1H), 6.27 (dt, *J* = 15.8, 5.2 Hz, 1H), 4.38 (d, *J* = 4.9 Hz, 2H), 2.37 (s, 3H), 0.98 (s, 9H), 0.15 (s, 6H) ppm.

^{13}C NMR (101 MHz, $CDCl_3$) δ 137.1, 134.3, 129.4, 129.2, 128.1, 126.3, 64.0, 26.0, 21.1, 18.4, -5.1 ppm.

HRMS (CI) Calcd for $C_{16}H_{26}OSi$ $[M]^+$: 262.1753, found: 262.1754.

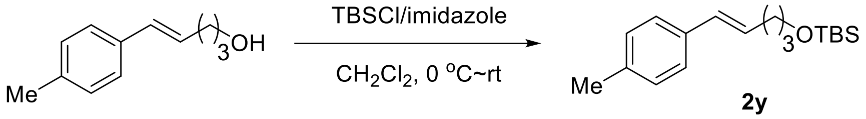

2y

(*E*)-*tert*-Butyldimethyl((5-(*p*-tolyl)pent-4-en-1-yl)oxy)silane (**2y**) was prepared as a colorless oil in 90% yield according to the same procedure for **2x**.

^{1}H NMR (400 MHz, $CDCl_3$) δ 7.28 (d, *J* = 7.9 Hz, 2H), 7.15 (d, *J* = 7.8 Hz, 2H), 6.42 (d, *J* = 15.8 Hz, 1H), 6.22 (dt, *J* = 15.8, 6.9 Hz, 1H), 3.71 (t, *J* = 6.4 Hz, 2H), 2.37 (s, 3H), 2.31 (q, *J* = 7.0 Hz, 2H), 1.74 (p, *J* = 6.7 Hz, 2H), 0.97 (s, 9H), 0.12 (s, 6H).

^{13}C NMR (101 MHz, $CDCl_3$) δ 136.4, 135.0, 129.9, 129.4, 129.1, 125.8, 62.5, 32.5, 29.3, 26.0, 21.1, 18.3, -5.3.

HRMS (CI) Calcd for $C_{18}H_{30}OSi$ $[M + H]^+$: 291.2139, found: 291.2135.

III. Catalyst Preparation

Catalyst **3i** is a new compound, and the detailed synthetic procedure is shown below.

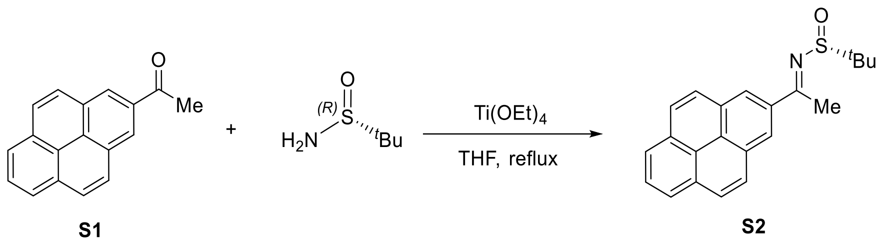

(*R*,*E*)-2-Methyl-*N*-(1-(pyren-2-yl)ethylidene)propane-2-sulfinamide (S2). To an oven-dried round-bottom flask charged with 1-(pyren-2-yl)ethan-1-one **S1**[10] (3.2 g, 13.3 mmol, 1.0 equiv), (*S*)-*tert*-butylsulfinamide (1.8 g, 14.7 mmol, 1.1 equiv), and anhydrous THF (13 mL) was added titanium ethoxide (6.14 mL, 29.3 mmol, 2.2 equiv). The mixture was heated to reflux at 85 °C. The progress of the reaction was monitored by TLC. Upon completion (~for 48 hours), the mixture was then cooled to room temperature, diluted with ethyl acetate, and quenched by the addition of saturated aqueous Na_2SO_4 (5 mL). The resulting slurry was stirred for 15 minutes, after which $MgSO_4$ was added, and the mixture was stirred for an additional 5 minutes. The slurry was filtered through a plug of celite in a Buchner funnel, and the filter cake was washed with ethyl acetate. The filtrate was dried over $MgSO_4$, filtered, and concentrated under reduced pressure. The residue was purified by silica gel flash chromatography (*n*-hexane/EtOAc = 5:3) to afford **S2** as a yellow solid (3.5 g, 75% yield).

$[\alpha]_D^{24}$ = +81.8 (*c* = 2.0, $CHCl_3$).

^{1}H NMR (400 MHz, $CDCl_3$) δ 8.54 (s, 2H), 8.11 (d, *J* = 7.6 Hz, 2H), 8.03 – 7.95 (m, 5H), 2.98 (s, 3H), 1.41 (s, 9H).

^{13}C NMR (101 MHz, $CDCl_3$) δ 176.7, 135.6, 131.4, 130.7, 128.0, 127.6, 126.7, 126.0, 125.2, 124.1, 123.3, 57.6, 22.6, 20.3.

HRMS (ESI) Calcd for $C_{22}H_{21}NaNOS$ $[M + Na]^+$: 370.1236, found: 370.1250.

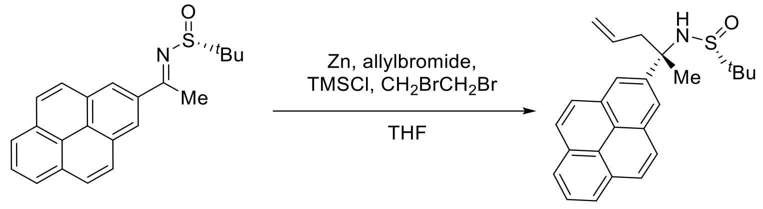

S2 **S3**

(*R*)-2-Methyl-*N*-((*S*)-2-(pyren-2-yl)pent-4-en-2-yl)propane-2-sulfinamide (S3). To a 100-mL oven-dried Schlenk flask equipped with a stir bar were added Zn dust (0.79 g, 12 mmol, 2.0 equiv) and anhydrous THF (24 mL). The flask was placed under nitrogen. Then, 1,2-dibromoethane (60 μL) and TMSCl (60 μL) were added to initiate the reaction. Subsequently, allylbromide (1.45 g, 12.0 mmol, 2.0 equiv) was added. The mixture was stirred for one hour, during which it transitioned from a heterogeneous gray solution to a homogeneous pale solution, indicating the consumption of zinc. Once the zinc was fully consumed, a solution of ketimine **S2** (2.08 g, 6.0 mmol, 1.0 equiv) in anhydrous THF (5 mL) was added. The mixture was stirred overnight before a saturated sodium bicarbonate aqueous solution (10 mL) and ethyl acetate (10 mL) were added. An emulsion was formed initially, but two distinct layers appeared after vigorous stirring for 2 hours. The two layers were separated, and the aqueous layer was washed with ethyl acetate (50 mL). The combined organic layers were washed with brine, dried over $MgSO_4$, and concentrated under reduced pressure to yield a yellow foam. The residue was purified by silica gel flash chromatography (*n*-hexane/EtOAc = 1:1) to afford **S3** as a yellow solid (1.84 g, 80% yield).

$[\alpha]_D^{24}$ = –83.4 (*c* = 1.0, $CHCl_3$).

^{1}H NMR (400 MHz, $CDCl_3$) δ 8.25 (s, 2H), 8.16 (d, *J* = 7.6 Hz, 2H), 8.11 – 8.01 (m, 4H), 7.98 (dd, *J* = 8.0, 7.2 Hz, 1H), 5.60 (ddt, *J* = 17.4, 10.1, 7.3 Hz, 1H), 5.31 – 5.20 (m, 1H), 5.13 (dd, *J* = 10.2, 2.1 Hz, 1H), 4.00 (s, 1H), 3.01 – 2.82 (m, 2H), 2.05 (s, 3H), 1.27 (s, 9H).

^{13}C NMR (101 MHz, $CDCl_3$) δ 143.0, 133.1, 131.03, 130.96, 127.6, 127.4, 125.9, 125.0, 124.4, 123.6, 123.1, 120.5, 60.4, 56.3, 49.9, 28.2, 22.8.

HRMS (ESI) Calcd for $C_{25}H_{27}NaNOS$ $[M + Na]^+$: 412.1705, found: 412.1719.

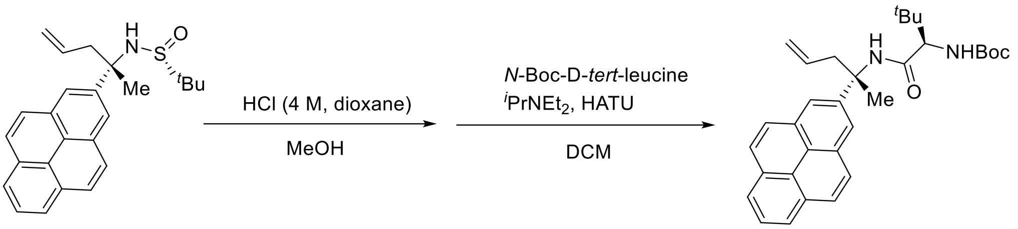

S3 S4

tert-Butyl ((R)-3,3-dimethyl-1-oxo-1-(((S)-2-(pyren-2-yl)pent-4-en-2-yl)amino) butan-2-yl)carbamate (S4).

Step 1: An oven-dried 100-mL round bottom flask equipped with a stir bar was charged with sulfinamide **S3** (1.84 g, 4.73 mmol, 1.0 equiv). Anhydrous methanol (47 mL) was added via syringe, and the reaction vessel was cooled to 0 °C using an ice-water bath. Over approximately 3 minutes, a solution of HCl in dioxane (24 mL, 4 M, 94.5 mmol, 20.0 equiv) was added. The ice bath was then removed, and the mixture was stirred for 30 minutes. The reaction completion was confirmed by TLC analysis. The reaction was bubbled with nitrogen for 30 minutes to remove dissolved HCl, and the solvent was subsequently removed under reduced pressure. The residue was placed under high vacuum (0.5 Torr) to remove remaining solvent.

Step 2: The above-prepared amine (as HCl salt) was re-dissolved in CH_2Cl_2 (18.9 mL, 0.25 M). The reaction vessel was fitted with a stir bar, sealed with a rubber septum, and placed under nitrogen. The solution was cooled to 0 °C in an ice-water bath, and DIPEA (2.5 mL, 1.84 g, 14.2 mmol, 3.0 equiv) was added over 2 minutes, causing the solution to become slightly cloudy. After stirring for 15 minutes, *N*-boc-D-*tert*-leucine (1.42 g, 6.15 mmol, 1.3 equiv) and HATU (2.05 g, 6.15 mmol, 1.3 equiv) were added. The mixture was then stirred overnight, allowing the ice-water bath to gradually melt during the first few hours. The solvent was then evaporated *in vacuo*, and the crude product was purified by silica gel flash chromatography (*n*-hexane/EtOAc = 10:1) to afford **S4** as a white solid (1.7 g, 73% yield).

$[\alpha]_D^{24} = -16.8$ (c = 1.0, $CHCl_3$).

^{1}H NMR (400 MHz, $CDCl_3$) δ 8.16 – 8.10 (m, 4H), 8.05 – 7.98 (m, 4H), 7.95 (dd, *J* = 8.0, 7.2 Hz, 1H), 6.32 (s, 1H), 5.62 (ddt, *J* = 17.2, 10.0, 7.3 Hz, 1H), 5.30 – 5.09 (m, 3H), 3.92 (d, *J* = 9.4 Hz, 1H), 2.92 (dd, *J* = 13.8, 7.5 Hz, 1H), 2.73 (dt, *J* = 13.7, 7.3 Hz, 1H), 2.03 (s, 3H), 1.48 (s, 9H), 1.05 (s, 9H).

^{13}C NMR (101 MHz, $CDCl_3$) δ 170.0, 156.1, 143.0, 133.1, 131.08, 130.96, 127.6, 127.4, 125.6, 124.8, 124.4, 123.5, 121.8, 119.9, 79.7, 62.8, 58.4, 48.4, 34.4, 28.3, 26.7, 25.6.

HRMS (ESI) Calcd for $C_{32}H_{38}NaN_2O_3$ $[M + Na]^+$: 521.2774, found: 521.2782.

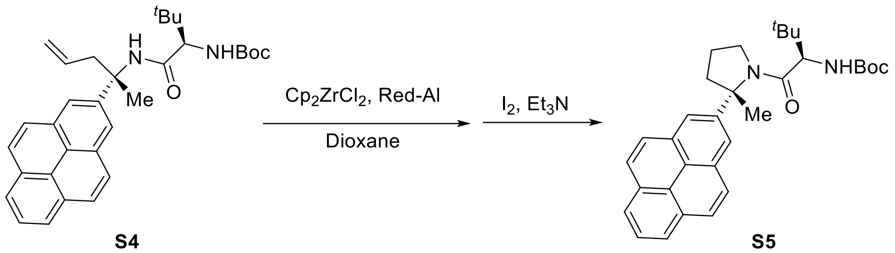

S4 **S5**

***tert*-Butyl ((*R*)-3,3-dimethyl-1-((*S*)-2-methyl-2-(pyren-2-yl)pyrrolidin-1-yl)-1-oxobutan-2-yl)carbamate.**

Step 1: An oven-dried 100-mL round bottom flask equipped with a stir bar and connected to a nitrogen/vacuum line was charged with zirconocene dichloride (7.02 g, 24.0 mmol, 4.0 equiv). The system was cycled between vacuum and nitrogen twice and then maintained under nitrogen. Dry dioxane (48 mL, 0.125 M with respect to allylamine) was added via syringe, and the suspension was stirred at room temperature. A solution of 60 wt% Red-Al in toluene (3.5 mL, 12.0 mmol, 2.0 equiv) was added dropwise, resulting in an off-white suspension that was stirred for 2.5 hours. The suspension was then cooled to 0 °C in an ice-water bath, and a solution of allyl amide **S4** (2.99 g, 6.0 mmol, 1.0 equiv) in 48 mL of DCM (0.125 M) was added dropwise. The ice bath was removed, and the resulting solution was stirred overnight at room temperature.

Step 2: The following day, the solution was cooled to 0 °C, and I_2 (6.4 g, 25.2 mmol, 4.2 equiv) and triethylamine (4.2 mL, 3.0 g, 5.0 equiv) were added simultaneously. The

ice bath was then removed, and the solution was stirred at room temperature for 3 hours. The solution was diluted with additional DCM and passed through a silica plug, eluting with ethyl acetate. The resulting filtrate was concentrated under reduced pressure and purified by silica gel flash chromatography (*n*-hexane/EtOAc = 10:1) to afford **S5** as a white solid (2.65 g, 88% yield).

$[\alpha]_D^{24}$ = –45.1 (*c* = 2.0, $CHCl_3$).

^{1}H NMR (400 MHz, $CDCl_3$) δ 8.16 (d, *J* = 7.6 Hz, 2H), 8.04 (d, *J* = 6.7 Hz, 5H), 8.03 – 7.94 (m, 2H), 5.29 (d, *J* = 10.0 Hz, 1H), 4.55 (d, *J* = 9.9 Hz, 1H), 4.43 (dq, *J* = 10.4, 4.6, 3.7 Hz, 1H), 3.96 (dt, *J* = 10.0, 7.6 Hz, 1H), 2.28 – 2.13 (m, 5H), 1.94 (qt, *J* = 11.9, 4.5 Hz, 2H), 1.60 (s, 9H), 1.12 (s, 9H).

^{13}C NMR (101 MHz, $CDCl_3$) δ 170.0, 156.2, 144.0, 130.9, 130.8, 127.6, 127.1, 125.4, 124.6, 124.4, 123.2, 121.4, 79.4, 67.5, 58.7, 50.1, 44.9, 34.9, 28.4, 26.4, 25.5, 22.4.

HRMS (ESI) Calcd for $C_{32}H_{38}NaN_2O_3$ $[M + Na]^+$: 521.2774, found: 521.2783.

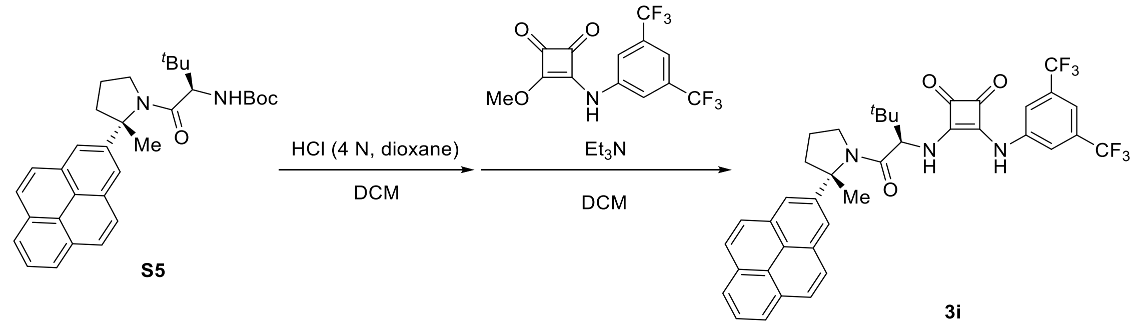

3-((3,5-Bis(trifluoromethyl)phenyl)amino)-4-(((*R*)-3,3-dimethyl-1-((*S*)-2-methyl-2-(pyren-2-yl)pyrrolidin-1-yl)-1-oxobutan-2-yl)amino)cyclobut-3-ene-1,2-dione (3i).

Step 1: Under nitrogen, an oven-dried 100-mL round bottom flask charged with the Boc-protected amine **S5** (2.65 g, 1.05 mmol, 1.0 equiv) was cooled to 0 °C in an ice-water bath. A solution of HCl in dioxane (26.5 mL, 4 N, 106 mmol, 20.0 equiv) was added dropwise. The mixture was then stirred at room temperature for 2 hours and concentrated to yield a yellow foam.

Step 2: Under nitrogen, the amine•HCl salt (1.61 g, 3.7 mmol, 1.0 equiv) was dissolved in DCM (20 mL, 0.19 M) in an oven-dried 100-mL round bottom flask, which was

cooled to 0 °C in an ice-water bath. Triethylamine (2.2 mL, 1.61 g, 15.9 mmol, 3.0 equiv) was then added. After stirring for 15 minutes, squaric ester (1.25 g, 3.7 mmol, 1.0 equiv) was added. Next, the mixture was allowed to warm to room temperature and stirred for 48 hours before it was concentrated under reduced pressure and purified by silica gel flash chromatography (*n*-hexane/EtOAc = 5:2) to afford **3i** as a white solid (1.8 g, 70% yield).

$[\alpha]_D^{23}$ = –84.9 (c = 2.0, $CHCl_3$).

^{1}H NMR (400 MHz, $CDCl_3$) δ 8.07 (d, J = 7.7 Hz, 2H), 7.94 (t, J = 7.6 Hz, 1H), 7.84 (d, J = 8.9 Hz, 2H), 7.63 (d, J = 12.5 Hz, 4H), 7.49 (d, J = 15.9 Hz, 1H), 5.27 – 4.78 (m, 1H), 4.39 (s, 1H), 4.00 (s, 1H), 2.28 – 1.91 (m, 7H), 1.68 (d, J = 4.2 Hz, 2H), 0.95 (s, 9H).

^{13}C NMR (101 MHz, $CDCl_3$) δ 168.5, 143.3, 132.9, 132.6, 130.8, 130.5, 127.5, 127.1, 125.8, 125.1, 123.9, 122.9, 120.8, 118.3, 116.6, 67.9, 62.5, 50.3, 44.7, 35.5, 25.8, 24.4, 23.0.

^{19}F NMR (376 MHz, $CDCl_3$) δ –62.7 (s, 6F) ppm.

HRMS (ESI) Calcd for $C_{39}H_{33}NaF_6N_3O_3$ $[M + Na]^+$: 728.2318, found: 728.2318.

IV. Condition Optimization

(1) Table S1. Screening of Catalysts[a]

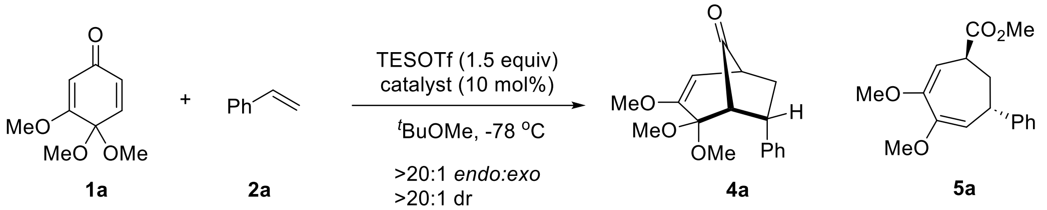

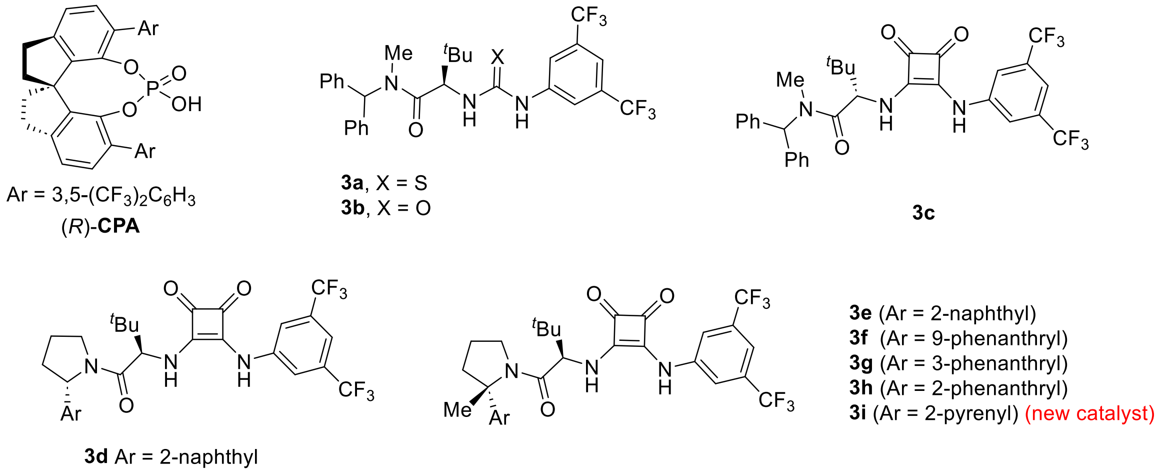

entry	catalyst	yield (**4a**)	er
1[b]	(*R*)-**CPA**	N.R.	---
2[c]	w/o	15% (**5a**)	---
3[d]	w/o	12%	---
4[e]	w/o	60%	---
5	w/o	trace	---
6	**3a**	trace	---
7	**3b**	trace	---
8	**3c**	90%	77:23
9	**3d**	>95%	85:15
10	**3e**	>95%	91:9
11	**3f**	>95%	92:8
12	**3g**	>95%	91:9
13	**3h**	>95%	94:6

14	**3i**	>95%	95:5

[a]Reaction conditions: **1a** (0.05 mmol), **2a** (0.075 mmol), TESOTf (1.5 equiv), catalyst (10 mol%), ^{t}BuOMe (0.5 mL), 48 h. The reaction was quenched with MeOH/Et_3N (3:1). Yields were based on the analysis of the ^{1}H NMR spectrum of the crude reaction mixture using CH_2Br_2 as an internal standard; er is determined by HPLC with a chiral stationary phase. (*R*)-**CPA**, rt. [c]HOTf (1.5 equiv), rt. [d]rt. [e]–60 °C.

(2) Table S2. Screening of Solvents[a]

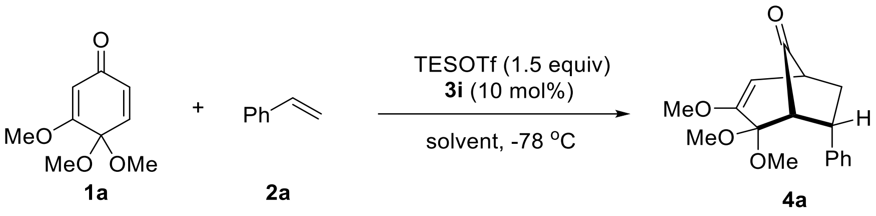

entry	solvent	yield (**4a**)	er
1	*ᵗ*BuOMe	>95%	95:5
2	Et_2O	80%	95:5
3	THF	75%	50:50
4	CH_2Cl_2	33%	50:50
5	toluene	trace	64:36
6	CPME	83%	95:5

[a]Reaction conditions: **1a** (0.05 mmol), **2a** (0.075 mmol), TESOTf (1.5 equiv), catalyst (10 mol%), solvent (0.5 mL), 48 h. The reaction was quenched with MeOH/Et_3N (3:1). Yields were based on the analysis of the ^{1}H NMR spectrum of the crude reaction mixture using CH_2Br_2 as an internal standard; er was determined by HPLC with a chiral stationary phase.

(3) Table S3. Screening of Additives[a]

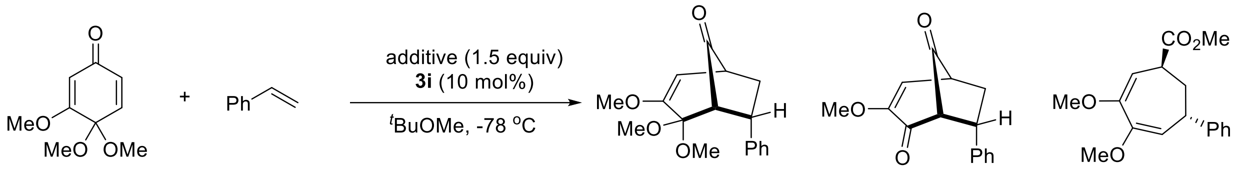

entry	additive	yield (**4a**)	er
1	TESOTf	>95%	95:5
2	TMSOTf	60%	95:5
3	TBSOTf	95%	95:5
4	TIPSOTf	75%	95:5
5	HOTf	85% (**5a**)	93:7
6[b]	TESOTf	75% (**4a'**)	95:5
7[c]	TESOTf	80% (**5a**)	95:5

[a]Reaction conditions: **1a** (0.05 mmol), **2a** (0.075 mmol), additive (1.5 equiv), **3i** (10 mol%), ^{t}BuOMe (0.5 mL), 48 h. The reaction was quenched with MeOH/Et_3N (3:1). Yields were based on the analysis of the ^{1}H NMR spectrum of the crude reaction mixture using CH_2Br_2 as an internal standard; er was determined by HPLC with a chiral stationary phase. [b]The reaction was quenched with saturated aqueous $NaHCO_3$. [c]The reaction was quenched with MeOH (1.5 equiv) and then stirred for 3~5 h at the same temperature before it was quenched with MeOH/Et_3N (3:1).

(4) Table S4. Effect of Concentration[a]

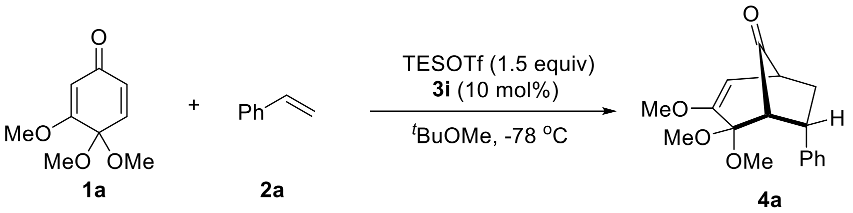

entry	M (mol/L)	yield (**4a**)	er
1	0.025	90%	93:7
2	0.05	>95%	93:7
3	0.1	>95%	95:5
4	0.2	90%	95:5
5	0.4	53%	94:6

[a]Reaction conditions: **1a** (0.05 mmol), **2a** (0.075 mmol), TESOTf (1.5 equiv), **3i** (10 mol%), ^{t}BuOMe (0.5 mL), 48 h. The reaction was quenched with MeOH/Et_3N (3:1). Yields were based on the analysis of the ^{1}H NMR spectrum of the crude reaction mixture using CH_2Br_2 as an internal standard; er was determined by HPLC with a chiral stationary phase.

V. Catalytic Enantioselective [5+2] Cycloaddition

General Procedure A.

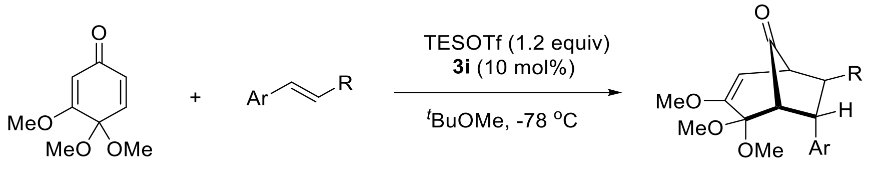

1a **2** **4**

An oven-dried 20-mL vial was charged with **1a** (0.4 mmol, 1.0 equiv), **2** (0.48 mmol, 1.2 equiv), catalyst **3i** (28.3 mg, 0.04 mmol, 10 mol%), and dry ^{t}BuOMe (0.1 M, 4.0 mL). The vial was sealed, and the reaction mixture was cooled to –78 °C in a low-temperature reactor. TESOTf (109.0 μL, 0.48 mmol, 1.2 equiv) was added via syringe. The reaction was stirred for 48 hours and then quenched by the addition of MeOH/Et_3N (0.5 mL, v/v = 3:1) via syringe. The mixture was allowed to stir at –78 ℃ for 5 minutes before warming to room temperature. The crude reaction mixture was concentrated and purified by silica gel flash chromatography (*n*-hexane/EtOAc = 10:1 to 5:2). The enantiomeric excess was determined by chiral HPLC following chromatographic purification on silica gel.

General Procedure B.

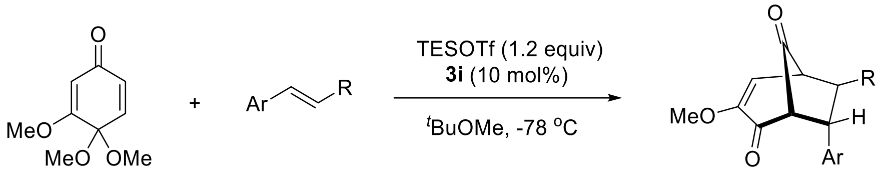

1a **2** **4'**

An oven-dried 20-mL vial was charged with **1a** (0.4 mmol, 1.0 equiv), **2** (0.48 mmol, 1.2 equiv), catalyst **3i** (28.3 mg, 0.04 mmol, 10 mol%), and dry ^{t}BuOMe (0.1 M, 4.0 mL). The vial was sealed, and the reaction mixture was cooled to –78 °C in a low-temperature reactor. TESOTf (109.0 μL, 0.48 mmol, 1.2 equiv) was added via syringe. The reaction was stirred for 48 hours and then quenched by the addition of a saturated $NaHCO_3$ solution (0.5 mL) via syringe, followed by warming to room temperature.

Water was added to dilute the reaction, and the mixture was extracted with ethyl acetate. The combined organic layers were concentrated and purified by silica gel flash chromatography (n-hexane/EtOAc = 5:1 to 1:1). The enantiomeric excess was determined by chiral HPLC following chromatographic purification on silica gel.

General Procedure C.

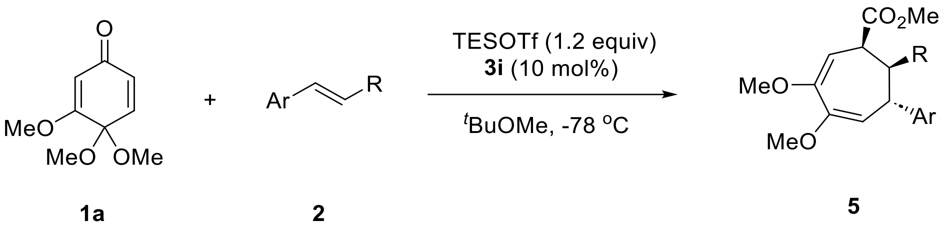

An oven-dried 20-mL vial was charged with **1a** (0.4 mmol, 1.0 equiv), **2** (0.48 mmol, 1.2 equiv), catalyst **3i** (28.3 mg, 0.04 mmol, 10 mol%), and dry ^{t}BuOMe (0.1 M, 4.0 mL). The vial was sealed, and the reaction mixture was cooled to –78 ^{o}C in a low-temperature reactor. TESOTf (109.0 μL, 0.48 mmol, 1.2 equiv) was added via syringe. The reaction was stirred for 48 hours at –78 ℃, and then MeOH (24.2 μL, 19.2 mg, 0.6 mmol, 1.5 equiv) was added via syringe and allowed to stir at –78 ℃ for 3 to 5 hours. The reaction was then quenched by the addition of MeOH/Et_3N solution (0.5 mL, v/v = 3:1) via syringe. The mixture was stirred at –78 ^{o}C for 5 minutes before warming to room temperature. The crude reaction mixture was concentrated and purified by silica gel flash chromatography (n-hexane/EtOAc = 10:1 to 5:1). The enantiomeric excess was determined by chiral HPLC following chromatographic purification on silica gel.

Unless otherwise noted, all racemic products (used as HPLC references for determining the enantiomeric ratio) were prepared by the same reaction at –60 ^{o}C for 5 to 6 hours without a chiral catalyst.

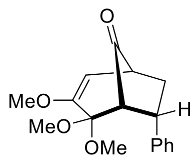

4a

(1*S*,5*S*,6*S*)-3,4,4-Trimethoxy-6-phenylbicyclo[3.2.1]oct-2-en-8-one (4a) was prepared as a thick oil according to the General Procedure A (eluent: *n*-hexane/EtOAc = 5:1, 113 mg, 98% yield, 95:5 er).

$[\alpha]_D^{24}$: –161.6 (c = 2.0, $CHCl_3$). HPLC analysis of the product: Daicel CHIRALPAK® ID-3 column; 10% *i*-PrOH in *n*-hexane; 1.0 mL/min; retention times: 12.0 min (minor), 13.6 min (major).

^{1}H NMR (400 MHz, $CDCl_3$) δ 7.34 – 7.25 (m, 4H), 7.24 – 7.16 (m, 1H), 5.21 (d, J = 7.4 Hz, 1H), 3.68 (s, 3H), 3.58 (ddd, J = 11.4, 7.8, 5.3 Hz, 1H), 3.18 (s, 3H), 3.10 (dd, J = 7.8, 1.5 Hz, 1H), 2.87 (ddd, J = 7.3, 5.6, 1.5 Hz, 1H), 2.59 (s, 3H), 2.42 (ddd, J = 13.2, 11.4, 5.8 Hz, 1H), 2.18 (dd, J = 13.1, 5.4 Hz, 1H).

^{13}C NMR (101 MHz, $CDCl_3$) δ 207.3, 154.3, 140.6, 129.6, 127.4, 126.5, 102.7, 100.3, 55.2, 54.7, 50.4, 47.4, 43.6, 37.8, 34.5.

HRMS (ESI) Calcd for $C_{17}H_{20}NaO_4$ $[M + Na]^+$: 311.1254, found: 311.1268.

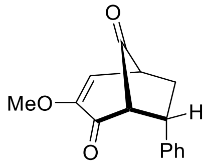

4a'

(1*S*,5*S*,7*S*)-3-Methoxy-7-phenylbicyclo[3.2.1]oct-3-ene-2,8-dione (4a') was prepared as a white solid according to the General Procedure B (eluent: *n*-hexane/EtOAc = 1:1, 73 mg, 75% yield, 95:5 er).

$[\alpha]_D^{23}$: –322.7 (c = 1.0, $CHCl_3$). HPLC analysis of the product: Daicel CHIRALPAK® IB N-3 column; 30% *i*-PrOH in *n*-hexane; 1.0 mL/min; retention times: 17.7 min (major), 23.0 min (minor).

^{1}**H NMR** (400 MHz, $CDCl_3$) δ 7.31 – 7.23 (m, 2H), 7.20 (t, *J* = 7.2 Hz, 1H), 7.11 – 7.03 (m, 2H), 6.49 (d, *J* = 8.5 Hz, 1H), 3.92 – 3.75 (m, 2H), 3.69 (s, 3H), 3.40 (t, *J* = 7.5 Hz, 1H), 2.76 (ddd, *J* = 13.5, 10.4, 6.6 Hz, 1H), 2.19 (dd, *J* = 13.4, 6.1 Hz, 1H).

^{13}**C NMR** (101 MHz, $CDCl_3$) δ 200.3, 189.9, 154.8, 138.3, 128.6, 127.9, 127.2, 118.7, 69.3, 55.6, 46.2, 39.0, 33.2.

HRMS (ESI) Calcd for $C_{15}H_{14}NaO_3$ $[M + Na]^+$: 265.0835, found: 265.0840.

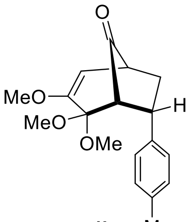

4b

(1*S*,5*S*,6*S*)-3,4,4-Trimethoxy-6-(p-tolyl)bicyclo[3.2.1]oct-2-en-8-one (4b) was prepared as a white solid according to the General Procedure A (eluent: *n*-hexane/EtOAc = 5:1, 110 mg, 91% yield, 97:3 er).

$[\alpha]_D^{23}$: –152.5 (*c* = 2.0, $CHCl_3$). HPLC analysis of the product: Daicel CHIRALPAK® ID-3 column; 10% *i*-PrOH in *n*-hexane; 1.0 mL/min; retention times: 11.4 min (minor), 12.1 min (major).

^{1}**H NMR** (400 MHz, $CDCl_3$) δ 7.16 (d, *J* = 8.2 Hz, 2H), 7.07 (d, *J* = 7.6 Hz, 2H), 5.19 (d, *J* = 7.3 Hz, 1H), 3.68 (s, 3H), 3.55 (ddd, *J* = 11.4, 7.8, 5.1 Hz, 1H), 3.18 (s, 3H), 3.08 (dd, *J* = 7.8, 1.6 Hz, 1H), 2.86 (ddd, *J* = 7.2, 5.5, 1.5 Hz, 1H), 2.62 (s, 3H), 2.40 (ddd, *J* = 13.1, 11.4, 5.8 Hz, 1H), 2.30 (s, 3H), 2.17 (dd, *J* = 13.1, 5.2 Hz, 1H).

^{13}**C NMR** (101 MHz, $CDCl_3$) δ 207.5, 154.3, 137.6, 136.0, 129.4, 128.2, 102.8, 100.2, 55.1, 54.7, 50.3, 47.5, 43.7, 37.3, 34.4, 20.8.

HRMS (ESI) Calcd for $C_{18}H_{22}NaO_4$ $[M + Na]^+$: 325.1410, found: 325.1425.

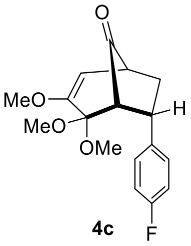

4c

(1S,5S,6S)-6-(4-Fluorophenyl)-3,4,4-trimethoxybicyclo[3.2.1]oct-2-en-8-one (4c) was prepared as a thick oil according to the General Procedure A (eluent: *n*-hexane/EtOAc = 5:1, 117 mg, 96% yield, 94:6 er).

$[\alpha]_D^{23}$: –151.4 (c = 2.0, $CHCl_3$). HPLC analysis of the product: Daicel CHIRALPAK® ID-3 column; 10% *i*-PrOH in *n*-hexane; 1.0 mL/min; retention times: 11.4 min (minor), 13.3 min (major).

^{1}H NMR (400 MHz, $CDCl_3$) δ 7.29 – 7.22 (m, 2H), 6.99 – 6.92 (m, 2H), 5.22 (d, J = 7.4 Hz, 1H), 3.69 (s, 3H), 3.58 (ddd, J = 11.5, 7.8, 5.2 Hz, 1H), 3.19 (s, 3H), 3.09 (dd, J = 7.8, 1.5 Hz, 1H), 2.88 (ddd, J = 7.3, 5.5, 1.5 Hz, 1H), 2.63 (s, 3H), 2.45 (ddd, J = 13.2, 11.4, 5.8 Hz, 1H), 2.13 (dd, J = 13.2, 5.3 Hz, 1H).

^{13}C NMR (101 MHz, $CDCl_3$) δ 207.1, 161.5 (d, J = 246.4 Hz), 154.3, 136.4 (d, J = 3.0 Hz), 131.0 (d, J = 8.1 Hz), 114.3 (d, J = 21.2 Hz), 102.7, 100.4, 55.0, 54.8, 50.4, 47.4, 43.6, 37.1, 34.8.

^{19}F NMR (376 MHz, $CDCl_3$) δ –116.3 (s, 1F) ppm.

HRMS (ESI) Calcd for $C_{17}H_{19}NaFO_4$ $[M + Na]^+$: 329.1159, found: 329.1170.

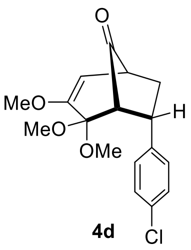

4d

(1S,6S)-6-(4-Chlorophenyl)-3,4,4-trimethoxybicyclo[3.2.1]oct-2-en-8-one (4d) was prepared as a white solid according to the General Procedure A (eluent: *n*-hexane/EtOAc = 5:1, 103 mg, 80% yield, 88:12 er).

$[\alpha]_D^{23}$: –151.6 (c = 2.0, $CHCl_3$). HPLC analysis of the product: Daicel CHIRALPAK® ID-3 column; 10% *i*-PrOH in *n*-hexane; 1.0 mL/min; retention times: 12.8 min (minor), 14.0

min (major).

^{1}H NMR (400 MHz, $CDCl_3$) δ 7.28 – 7.17(m, 4H), 5.21 (d, *J* = 7.4 Hz, 1H), 3.69 (s, 3H), 3.61 – 3.50 (m, 1H), 3.18 (s, 3H), 3.09 (d, *J* = 7.7 Hz, 1H), 2.88 (t, *J* = 6.3 Hz, 1H), 2.64 (s, 3H), 2.43 (ddd, *J* = 13.2, 11.4, 5.7 Hz, 1H), 2.13 (dd, *J* = 13.2, 5.1 Hz, 1H).

^{13}C NMR (101 MHz, $CDCl_3$) δ 206.8, 154.3, 139.4, 132.3, 130.8, 127.6, 102.7, 100.3, 55.0, 54.8, 50.4, 47.5, 43.6, 37.2, 34.5.

HRMS (CI) Calcd for $C_{17}H_{19}ClO_4$ $[M]^+$: 322.0972, found: 322.0979.

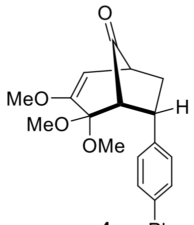

4e

(1*S*,5*S*,6*S*)-6-([1,1'-Biphenyl]-4-yl)-3,4,4-trimethoxybicyclo[3.2.1]oct-2-en-8-one (4e) was prepared as a white solid according to the General Procedure A (eluent: *n*-hexane/EtOAc = 5:1, 127 mg, 88% yield, 97:3 er).

$[\alpha]_D^{23}$: –147.5 (*c* = 2.0, $CHCl_3$). HPLC analysis of the product: Daicel CHIRALPAK® ID-3 column; 10% *i*-PrOH in *n*-hexane; 1.0 mL/min; retention times: 15.5 min (minor), 16.4 min (major).

^{1}H NMR (400 MHz, $CDCl_3$) δ 7.62 – 7.57 (m, 2H), 7.52 (d, *J* = 8.4 Hz, 2H), 7.44 – 7.39 (m, 2H), 7.36 – 7.28 (m, 3H), 5.22 (d, *J* = 7.4 Hz, 1H), 3.69 (s, 3H), 3.61 (ddd, *J* = 11.4, 7.7, 5.3 Hz, 1H), 3.20 (s, 3H), 3.14 (dd, *J* = 7.8, 1.5 Hz, 1H), 2.88 (ddd, *J* = 7.2, 5.5, 1.5 Hz, 1H), 2.65 (s, 3H), 2.44 (ddd, *J* = 13.2, 11.4, 5.8 Hz, 1H), 2.19 (dd, *J* = 13.1, 5.3 Hz, 1H).

^{13}C NMR (101 MHz, $CDCl_3$) δ 207.4, 154.4, 140.5, 139.9, 139.2, 130.1, 128.5, 127.0, 126.8, 126.2, 102.9, 100.4, 55.3, 54.8, 50.5, 47.6, 43.7, 37.6, 34.7.

HRMS (ESI) Calcd for $C_{23}H_{24}NaO_4$ $[M + Na]^+$: 387.1567, found: 387.1581.

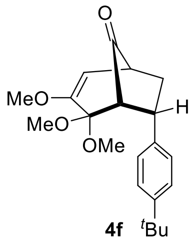

4f

(1*S*,5*S*,6*S*)-6-(4-(*tert*-Butyl)phenyl)-3,4,4-trimethoxybicyclo[3.2.1]oct-2-en-8-one (4f) was prepared as a white solid according to the General Procedure A (eluent: *n*-hexane/EtOAc = 5:1, 110 mg, 80% yield, 98:2 er).

$[\alpha]_D^{23}$: –159.8 (c = 2.0, $CHCl_3$). HPLC analysis of the product: Daicel CHIRALPAK® IB N-3 column; 10% *i*-PrOH in *n*-hexane; 1.0 mL/min; retention times: 7.9 min (major), 8.7 min (minor).

^{1}H NMR (400 MHz, $CDCl_3$) δ 7.29 (d, J = 8.5 Hz, 2H), 7.21 (d, J = 8.4 Hz, 2H), 5.23 (d, J = 7.5 Hz, 1H), 3.70 (s, 3H), 3.55 (ddd, J = 11.4, 7.6, 5.6 Hz, 1H), 3.20 (s, 3H), 3.09 (dd, J = 7.7, 1.5 Hz, 1H), 2.86 (ddd, J = 7.3, 5.6, 1.5 Hz, 1H), 2.57 (s, 3H), 2.43 (ddd, J = 13.1, 11.5, 6.0 Hz, 1H), 2.15 (dd, J = 13.0, 5.6 Hz, 1H), 1.30 (s, 9H).

^{13}C NMR (101 MHz, $CDCl_3$) δ 207.6, 154.4, 149.3, 137.5, 129.5, 124.4, 102.9, 100.6, 55.5, 54.8, 50.5, 47.5, 43.7, 37.6, 35.0, 34.2, 31.2.

HRMS (ESI) Calcd for $C_{21}H_{28}NaO_4$ $[M + Na]^+$: 367.1880, found: 367.1898.

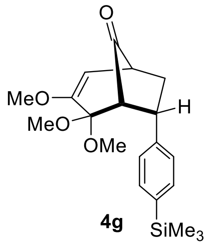

4g

(1*S*,5*S*,6*S*)-3,4,4-Trimethoxy-6-(4-(trimethylsilyl)phenyl)bicyclo[3.2.1]oct-2-en-8-one (4g) was prepared as a colorless oil according to the General Procedure A (eluent: *n*-hexane/EtOAc = 5:1, 129 mg, 90% yield, 95:5 er).

$[\alpha]_D^{23}$: –127.6 (c = 2.0, $CHCl_3$). HPLC analysis of the product: Daicel CHIRALPAK® IB N-3 column; 10% *i*-PrOH in *n*-hexane; 1.0 mL/min; retention times: 7.7 min (major), 8.6 min (minor).

^{1}H NMR (400 MHz, $CDCl_3$) δ 7.46 – 7.40 (m, 2H), 7.29 – 7.25 (m, 2H), 5.22 (d, *J* = 7.5 Hz, 1H), 3.70 (s, 3H), 3.57 (ddd, *J* = 11.5, 7.8, 5.5 Hz, 1H), 3.19 (s, 3H), 3.11 (dd, *J* = 7.7, 1.6 Hz, 1H), 2.59 (s, 3H), 2.48 – 2.36 (m, 1H), 2.18 (dd, *J* = 13.1, 5.4 Hz, 1H), 0.24 (s, 9H).

^{13}C NMR (101 MHz, $CDCl_3$) δ 207.4, 154.4, 141.3, 138.3, 132.6, 129.2, 102.8, 100.5, 55.4, 54.8, 50.5, 47.5, 43.7, 37.9, 34.7, –1.2.

HRMS (ESI) Calcd for $C_{20}H_{28}NaO_4Si$ $[M + Na]^+$: 383.1649, found: 383.1667.

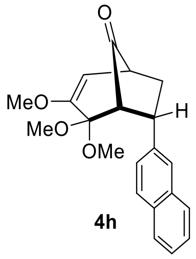

4h

(1*S*,5*S*,6*S*)-3,4,4-Trimethoxy-6-(naphthalen-2-yl)bicyclo[3.2.1]oct-2-en-8-one (4h) was prepared as a white solid according to the General Procedure A (eluent: *n*-hexane/EtOAc = 5:1, 109 mg, 80% yield, 96:4 er).

$[\alpha]_D^{23}$: –183.6 (*c* = 2.0, $CHCl_3$). HPLC analysis of the product: Daicel CHIRALPAK® ID-3 column; 10% *i*-PrOH in *n*-hexane; 1.0 mL/min; retention times: 14.8 min (minor), 18.6 min (major).

^{1}H NMR (400 MHz, $CDCl_3$) δ 7.80 – 7.70 (m, 3H), 7.67 (s, 1H), 7.46 – 7.36 (m, 3H), 5.21 (d, *J* = 7.4 Hz, 1H), 3.76 – 3.66 (m, 4H), 3.20 – 3.12 (m, 4H), 2.92 – 2.86 (m, 1H), 2.50 (s, 3H), 2.47 – 2.38 (m, 1H), 2.27 (dd, *J* = 13.2, 5.2 Hz, 1H).

^{13}C NMR (101 MHz, $CDCl_3$) δ 207.2, 154.4, 138.2, 132.6, 132.1, 128.1, 127.8, 127.33, 127.29, 126.9, 125.6, 125.3, 102.8, 100.3, 55.3, 54.7, 50.4, 47.4, 43.7, 37.9, 34.3.

HRMS (ESI) Calcd for $C_{21}H_{22}NaO_4$ $[M + Na]^+$: 361.1410, found: 361.1426.

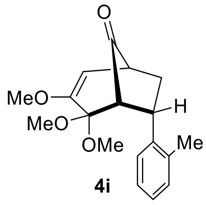

(1S,5S,6S)-3,4,4-Trimethoxy-6-(o-tolyl)bicyclo[3.2.1]oct-2-en-8-one (4i) was prepared as a colorless oil according to the General Procedure A (eluent: n-hexane/EtOAc = 5:1, 113 mg, 94% yield, 95:5 er).

$[\alpha]_D^{23}$: –188.9 (c = 2.0, $CHCl_3$). HPLC analysis of the product: Daicel CHIRALCEL® OX-3 column; 10% i-PrOH in n-hexane; 1.0 mL/min; retention times: 11.0 min (major), 12.9 min (minor).

^{1}H NMR (400 MHz, $CDCl_3$) δ 7.31 – 7.27 (m, 1H), 7.20 – 7.15 (m, 1H), 7.14 – 7.08 (m, 2H), 5.21 (d, J = 7.5 Hz, 1H), 3.79 (ddd, J = 11.3, 7.6, 5.7 Hz, 1H), 3.68 (s, 3H), 3.18 (s, 3H), 3.14 (dd, J = 7.5, 1.5 Hz, 1H), 2.89 (ddd, J = 7.3, 5.5, 1.5 Hz, 1H), 2.56 (s, 3H), 2.45 – 2.35 (m, 4H), 2.25 (dd, J = 13.0, 5.6 Hz, 1H).

^{13}C NMR (101 MHz, $CDCl_3$) δ 207.7, 154.5, 138.7, 135.8, 129.4, 129.3, 126.3, 125.6, 102.9, 100.2, 54.7, 53.6, 50.5, 47.3, 43.6, 34.3, 33.0, 20.0.

HRMS (ESI) Calcd for $C_{18}H_{22}NaO_4$ $[M + Na]^+$: 325.1410, found: 325.1425.

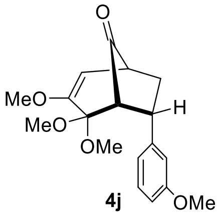

(1S,5S,6S)-3,4,4-Trimethoxy-6-(3-methoxyphenyl)bicyclo[3.2.1]oct-2-en-8-one (**4j**) was prepared as a colorless oil according to the General Procedure A (eluent: n-hexane/EtOAc = 5:2, 110 mg, 87% yield, 94:6 er).

$[\alpha]_D^{23}$: –132.4 (c = 2.0, $CHCl_3$). HPLC analysis of the product: Daicel CHIRALPAK® ID-3 column; 10% i-PrOH in n-hexane; 1.0 mL/min; retention times: 16.5 min (minor), 20.3 min (major).

^{1}H NMR (400 MHz, $CDCl_3$) δ 7.18 (t, J = 7.9 Hz, 1H), 6.90 – 6.81 (m, 2H), 6.75 (ddd, J = 8.3, 2.6, 0.9 Hz, 1H), 5.19 (d, J = 7.3 Hz, 1H), 3.77 (s, 3H), 3.68 (s, 3H), 3.60 – 3.50 (m,

1H), 3.18 (s, 3H), 3.11 (dd, *J* = 7.8, 1.6 Hz, 1H), 2.86 (ddd, *J* = 7.2, 5.5, 1.5 Hz, 1H), 2.66 (s, 3H), 2.40 (ddd, *J* = 13.2, 11.4, 5.8 Hz, 1H), 2.18 (dd, *J* = 13.1, 5.2 Hz, 1H).

^{13}C NMR (101 MHz, $CDCl_3$) δ 207.4, 158.7, 154.4, 142.4, 128.3, 122.1, 115.9, 111.3, 102.8, 100.1, 55.2, 54.8, 54.7, 50.4, 47.7, 43.7, 37.7, 34.3.

HRMS (ESI) Calcd for $C_{18}H_{22}NaO_5$ [M + Na]$^+$: 341.1359, found: 341.1375.

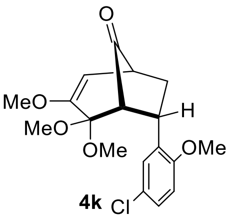

4k

(1*S*,5*S*,6*S*)-6-(5-Chloro-2-methoxyphenyl)-3,4,4-trimethoxybicyclo[3.2.1]oct-2-en-8-one (4k) was prepared as a colorless oil according to the General Procedure A (eluent: *n*-hexane/EtOAc = 5:2, 113 mg, 85% yield, 95:5 er).

$[\alpha]_D^{23}$: –160.0 (*c* = 1.0, $CHCl_3$). HPLC analysis of the product: Daicel CHIRALPAK® AD-3 column; 10% *i*-PrOH in *n*-hexane; 1.0 mL/min; retention times: 6.9 min (minor), 7.6 min (major).

^{1}H NMR (400 MHz, $CDCl_3$) δ 7.18 – 7.10 (m, 2H), 6.73 (d, *J* = 8.6 Hz, 1H), 5.15 (d, *J* = 7.1 Hz, 1H), 4.03 (ddd, *J* = 10.9, 7.8, 4.9 Hz, 1H), 3.81 (s, 3H), 3.67 (s, 3H), 3.24 (dd, *J* = 7.7, 1.5 Hz, 1H), 3.15 (s, 3H), 2.87 (ddd, *J* = 6.2, 4.1, 1.2 Hz, 1H), 2.76 (s, 3H), 2.32 – 2.15 (m, 2H).

^{13}C NMR (101 MHz, $CDCl_3$) δ 207.7, 155.9, 154.3, 131.4, 129.8, 126.8, 125.2, 110.1, 103.0, 99.6, 55.3, 54.9, 53.0, 50.5, 47.6, 44.0, 32.4, 29.1.

HRMS (ESI) Calcd for $C_{18}H_{21}NaClO_5$ [M + Na]$^+$: 375.0970, found: 375.0977.

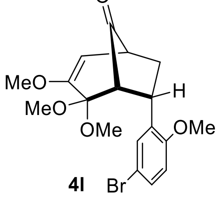

4l

(1*S*,5*S*,6*S*)-6-(5-Bromo-2-methoxyphenyl)-3,4,4-trimethoxybicyclo[3.2.1]oct-2-en-8-

one (4l) was prepared as a colorless oil according to the General Procedure A (eluent: *n*-hexane/EtOAc = 5:2, 119 mg, 80% yield, 94:6 er).

$[\alpha]_D^{23}$: –143.0 (*c* = 1.0, $CHCl_3$). HPLC analysis of the product: Daicel CHIRALPAK® AD-3 column; 10% *i*-PrOH in *n*-hexane; 1.0 mL/min; retention times: 7.1 min (minor), 8.1 min (major).

^{1}H NMR (400 MHz, $CDCl_3$) δ 7.30 – 7.23 (m, 2H), 6.69 (d, *J* = 8.6 Hz, 1H), 5.15 (d, *J* = 7.1 Hz, 1H), 4.02 (ddd, *J* = 10.7, 7.8, 5.1 Hz, 1H), 3.81 (s, 3H), 3.68 (s, 3H), 3.24 (dd, *J* = 7.8, 1.5 Hz, 1H), 3.14 (s, 3H), 2.90 – 2.83 (m, 1H), 2.77 (s, 3H), 2.31 – 2.16 (m, 2H).

^{13}C NMR (101 MHz, $CDCl_3$) δ 207.6, 156.3, 154.2, 132.6, 131.8, 129.7, 112.6, 110.6, 103.0, 99.5, 55.2, 54.9, 52.9, 50.4, 47.6, 44.0, 32.3, 29.1.

HRMS (ESI) Calcd for $C_{18}H_{21}NaBrO_5$ $[M + Na]^+$: 419.0464, found: 419.0461.

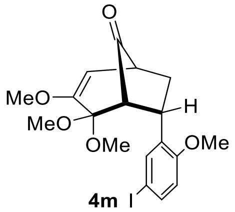

4m

(1*S*,5*S*,6*S*)-6-(5-Iodo-2-methoxyphenyl)-3,4,4-trimethoxybicyclo[3.2.1]oct-2-en-8-one (**4m**) was prepared as a colorless oil according to the General Procedure A (eluent: *n*-hexane/EtOAc = 5:2, 142 mg, 80% yield, 91:9 er).

$[\alpha]_D^{23}$: –95.0 (*c* = 2.0, $CHCl_3$). HPLC analysis of the product: Daicel CHIRALPAK® AD-3 column; 10% *i*-PrOH in *n*-hexane; 1.0 mL/min; retention times: 7.3 min (minor), 8.9 min (major).

^{1}H NMR (400 MHz, $CDCl_3$) δ 7.51 – 7.41 (m, 2H), 6.58 (d, *J* = 8.5 Hz, 1H), 5.13 (d, *J* = 7.1 Hz, 1H), 3.99 (ddd, *J* = 10.0, 7.7, 5.2 Hz, 1H), 3.80 (s, 3H), 3.70 (s, 3H), 3.23 (dd, *J* = 7.8, 1.4 Hz, 1H), 3.14 (s, 3H), 2.89 – 2.84 (m, 1H), 2.77 (s, 3H), 2.29 – 2.16 (m, 2H).

^{13}C NMR (101 MHz, $CDCl_3$) δ 207.6, 157.1, 154.2, 138.5, 135.7, 132.3, 111.2, 103.0, 99.4, 82.7, 55.1, 55.0, 52.9, 50.4, 47.6, 44.0, 32.2, 28.9.

HRMS (ESI) Calcd for $C_{18}H_{21}NaIO_5$ $[M + Na]^+$: 467.0326, found: 467.0339.

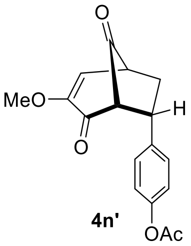

4n'

4-((1*S*,5*S*,6*S*)-3-Methoxy-4,8-dioxobicyclo[3.2.1]oct-2-en-6-yl)phenyl acetate (**4n'**) was prepared as a white solid according to the General Procedure B (eluent: *n*-hexane/EtOAc = 1:2, 72 mg, 60% yield, 96:4 er).

$[\alpha]_D^{23}$: –253.9 (*c* = 1.0, $CHCl_3$). HPLC analysis of the product: Daicel CHIRALPAK® IB N-3 column; 30% *i*-PrOH in *n*-hexane; 1.0 mL/min; retention times: 24.4 min (major), 36.5 min (minor).

^{1}H NMR (400 MHz, $CDCl_3$) δ 7.09 (d, *J* = 8.7 Hz, 2H), 7.00 (d, *J* = 8.6 Hz, 2H), 6.50 (d, *J* = 8.5 Hz, 1H), 3.91 – 3.78 (m, 2H), 3.68 (s, 3H), 3.39 (ddd, *J* = 8.5, 6.3, 1.9 Hz, 1H), 2.77 (ddd, *J* = 13.5, 10.2, 6.6 Hz, 1H), 2.26 (s, 3H), 2.13 (dd, *J* = 13.5, 6.1 Hz, 1H).

^{13}C NMR (101 MHz, $CDCl_3$) δ 200.1, 189.8, 169.2, 154.8, 149.6, 136.0, 129.0, 121.7, 118.9, 69.1, 55.6, 46.2, 38.4, 33.7, 21.0.

HRMS (ESI) Calcd for $C_{17}H_{16}NaO_5$ $[M + Na]^+$: 323.0890, found: 323.0897.

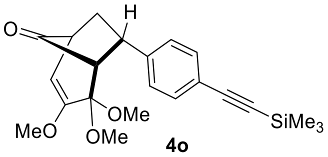

4o

(1*S*,5*S*,6*S*)-3,4,4-Trimethoxy-6-(4-((trimethylsilyl)ethynyl)phenyl)bicyclo [3.2.1]oct-2-en-8-one (4o) was prepared as a colorless oil according to the General Procedure A (eluent: *n*-hexane/EtOAc = 5:2, 114 mg, 75% yield, 91:9 er).

$[\alpha]_D^{23}$: –132.5 (*c* = 2.0, $CHCl_3$). HPLC analysis of the product: Daicel CHIRALPAK® IB N-3 column; 10% *i*-PrOH in *n*-hexane; 1.0 mL/min; retention times: 91.2 min (major), 11.3 min (minor).

^{1}H NMR (400 MHz, CD_3OD) δ 7.35 – 7.25 (m, 4H), 5.33 (d, *J* = 7.4 Hz, 1H), 3.65 (s, 3H), 3.64 – 3.58 (m, 1H), 3.10 (s, 4H), 2.81 (ddd, *J* = 7.2, 5.5, 1.5 Hz, 1H), 2.60 (s, 3H), 2.37 (ddd, *J* = 13.1, 11.4, 5.8 Hz, 1H), 2.17 (dd, *J* = 13.1, 5.2 Hz, 1H), 0.22 (s, 9H).

^{13}**C NMR** (101 MHz, CD_3OD) δ 209.1, 155.5, 143.7, 132.0, 131.2, 122.5, 106.5, 104.3, 102.1, 94.1, 56.5, 55.3, 50.9, 48.1, 45.3, 39.0, 35.1, 0.0.

HRMS (ESI) Calcd for $C_{22}H_{28}NaO_4Si$ $[M + Na]^+$: 407.1649, found: 407.1654.

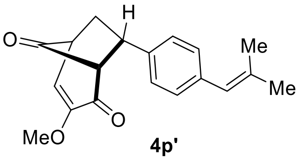

4p'

(1*S*,5*S*,7*S*)-3-Methoxy-7-(4-(2-methylprop-1-en-1-yl)phenyl)bicyclo[3.2.1]oct-3-ene-2,8-dione (4p') was prepared as a white solid according to the General Procedure B (eluent: *n*-hexane/EtOAc = 1:2, 83 mg, 70% yield, 98:2 er).

$[\alpha]_D^{23}$: –331.3 (*c* = 1.0, $CHCl_3$). HPLC analysis of the product: Daicel CHIRALPAK® IB N-3 column; 30% *i*-PrOH in *n*-hexane; 1.0 mL/min; retention times: 10.0 min (major), 14.3 min (minor).

^{1}**H NMR** (400 MHz, $CDCl_3$) δ 7.12 (d, *J* = 7.8 Hz, 2H), 7.00 (d, *J* = 7.9 Hz, 2H), 6.48 (d, *J* = 8.6 Hz, 1H), 6.16 (s, 1H), 3.84 – 3.75 (m, 2H), 3.68 (s, 3H), 3.38 (t, *J* = 7.6 Hz, 1H), 2.79 – 2.66 (m, 1H), 2.21 – 2.10 (m, 1H), 1.87 (s, 3H), 1.81 (s, 3H).

^{13}**C NMR** (101 MHz, $CDCl_3$) δ 200.3, 190.0, 154.8, 137.6, 135.63, 135.62, 128.9, 127.6, 124.3, 118.8, 69.3, 55.6, 46.2, 38.7, 33.3, 26.7, 19.2.

HRMS (ESI) Calcd for $C_{19}H_{20}NaO_3$ $[M + Na]^+$: 319.1305, found: 319.1314.

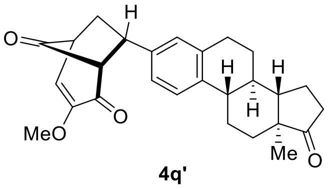

4q'

(1*S*,5*S*,7*S*)-3-Methoxy-7-((9S,13S,14S)-13-methyl-17-oxo-7,8,9,11,12,13,14,15,16, 17-decahydro-6H-cyclopenta[a]phenanthren-3-yl)bicyclo[3.2.1]oct-3-ene-2,8-dione (4q') was prepared as a white solid according to the General Procedure B (eluent: *n*-hexane/EtOAc = 1:2, 120 mg, 75% yield, >20:1 dr).

$[\alpha]_D^{23}$: –166.3 (*c* = 1.0, $CHCl_3$).

^{1}H NMR (400 MHz, $CDCl_3$) δ 7.19 (d, *J* = 8.0 Hz, 1H), 6.86 – 6.79 (m, 2H), 6.50 (d, *J* = 8.5 Hz, 1H), 3.84 – 3.75 (m, 2H), 3.71 (s, 3H), 3.40 (ddd, *J* = 8.2, 6.2, 1.8 Hz, 1H), 2.90 – 2.81 (m, 2H), 2.74 (ddd, *J* = 13.1, 10.2, 4.1 Hz, 1H), 2.49 (dd, *J* = 18.7, 8.7 Hz, 1H), 2.41 – 2.31 (m, 1H), 2.25 – 1.88 (m, 6H), 1.69 – 1.34 (m, 6H), 0.89 (s, 3H).

^{13}C NMR (101 MHz, $CDCl_3$) δ 220.6, 200.4, 190.0, 154.9, 138.8, 136.6, 135.7, 128.9, 125.6, 124.9, 118.6, 69.3, 55.6, 50.2, 47.7, 46.3, 44.0, 38.5, 37.8, 35.7, 33.1, 31.4, 29.1, 26.2, 25.4, 21.4, 13.6.

HRMS (ESI) Calcd for $C_{27}H_{30}NaO_4$ [M + Na]$^+$: 441.2036, found: 441.2041.

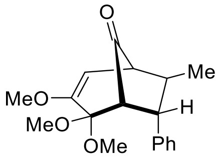

4r

(1*S*,5*S*,6*S*,7*S*)-3,4,4-Trimethoxy-7-methyl-6-phenylbicyclo[3.2.1]oct-2-en-8-one (4r) was prepared as a white solid according to the General Procedure A (eluent: *n*-hexane/EtOAc = 5:1, 115 mg, 95% yield, 91:9 er).

$[\alpha]_D^{23}$: –121.2 (*c* = 2.0, $CHCl_3$). HPLC analysis of the product: Daicel CHIRALPAK® ID-3 column; 10% *i*-PrOH in *n*-hexane; 1.0 mL/min; retention times: 9.6 min (minor), 10.6 min (major).

^{1}H NMR (400 MHz, $CDCl_3$) δ 7.30 – 7.24 (m, 4H), 7.24 – 7.19 (m, 1H), 5.23 (d, *J* = 7.4 Hz, 1H), 3.69 (s, 3H), 3.19 (s, 3H), 3.13 (dd, *J* = 7.6, 1.6 Hz, 1H), 2.92 (dd, *J* = 7.7, 5.1 Hz, 1H), 2.59 – 2.49 (m, 5H), 1.05 (d, *J* = 7.0 Hz, 3H).

^{13}C NMR (101 MHz, $CDCl_3$) δ 206.7, 154.0, 139.7, 129.9, 127.4, 126.6, 102.4, 99.7, 56.6, 54.7, 50.8, 50.4, 48.6, 47.3, 42.9, 21.3.

HRMS (ESI) Calcd for $C_{18}H_{22}NaO_4$ [M + Na]$^+$: 325.1410, found: 325.1417.

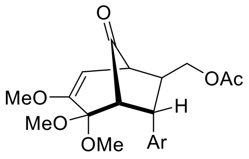

Ar = 4-$CH_3C_6H_4$

4s

((1*S*,5*S*,6*S*,7*S*)-2,2,3-Trimethoxy-8-oxo-7-(*p*-tolyl)bicyclo[3.2.1]oct-3-en-6-yl)methyl acetate (4s) was prepared as a white solid according to the General Procedure A (eluent: *n*-hexane/EtOAc = 5:2, 120 mg, 80% yield, 92:8 er).

$[\alpha]_D^{23}$: –117.8 (c = 2.0, $CHCl_3$). HPLC analysis of the product: Daicel CHIRALPAK® ID-3 column; 20% *i*-PrOH in *n*-hexane; 1.0 mL/min; retention times: 17.3 min (minor), 22.8 min (major).

^{1}H NMR (400 MHz, $CDCl_3$) δ 7.14 (d, J = 8.1 Hz, 2H), 7.07 (d, J = 8.1 Hz, 2H), 5.22 (d, J = 7.6 Hz, 1H), 4.08 (dd, J = 11.2, 5.1 Hz, 1H), 3.81 (dd, J = 11.2, 7.2 Hz, 1H), 3.69 (s, 3H), 3.18 (s, 3H), 3.14 – 3.04 (m, 2H), 2.80 (dd, J = 7.6, 1.5 Hz, 1H), 2.77 – 2.70 (m, 1H), 2.56 (s, 3H), 2.30 (s, 3H), 1.95 (s, 3H).

^{13}C NMR (101 MHz, $CDCl_3$) δ 206.1, 170.7, 154.7, 136.5, 135.9, 129.7, 128.4, 102.6, 99.1, 65.5, 56.3, 54.9, 50.6, 47.5, 47.3, 46.8, 42.2, 20.9, 20.4.

HRMS (ESI) Calcd for $C_{21}H_{26}NaO_6$ $[M + Na]^+$: 397.1622, found: 397.1630.

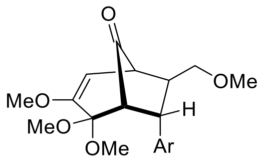

Ar = 4-$CH_3C_6H_4$

4t

(1*S*,5*S*,6*S*,7*S*)-3,4,4-Trimethoxy-7-(methoxymethyl)-6-(*p*-tolyl)bicyclo[3.2.1] oct-2-en-8-one (4t) was prepared as a white solid according to the General Procedure A (eluent: *n*-hexane/EtOAc = 5:2, 122 mg, 88% yield, 92:8 er).

$[\alpha]_D^{23}$: –118.9 (c = 2.0, $CHCl_3$). HPLC analysis of the product: Daicel CHIRALPAK® IB N-3 column; 10% *i*-PrOH in *n*-hexane; 1.0 mL/min; retention times: 8.6 min (major), 9.7 min (minor).

^{1}H NMR (400 MHz, $CDCl_3$) δ 7.17 (d, *J* = 7.8 Hz, 2H), 7.08 (d, *J* = 7.8 Hz, 2H), 5.23 (d, *J* = 7.6 Hz, 1H), 3.68 (s, 3H), 3.31 – 3.24 (m, 4H), 3.19 (s, 3H), 3.15 – 3.06 (m, 2H), 2.99 (dd, *J* = 7.7, 5.6 Hz, 1H), 2.88 (dd, *J* = 7.6, 1.7 Hz, 1H), 2.72 – 2.65 (m, 1H), 2.57 (s, 3H), 2.31 (s, 3H).

^{13}C NMR (101 MHz, $CDCl_3$) δ 206.8, 154.4, 136.4 (2C signal), 129.7, 128.3, 102.6, 99.7, 74.3, 58.7, 56.2, 54.8, 50.5, 48.5, 47.5, 46.9, 41.8, 20.9.

HRMS (ESI) Calcd for $C_{20}H_{26}NaO_5$ $[M + Na]^+$: 369.1672, found: 369.1680.

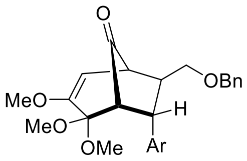

Ar = 4-$CH_3C_6H_4$

4u

(1*S*,5*S*,6*S*,7*S*)-7-((Benzyloxy)methyl)-3,4,4-trimethoxy-6-(*p*-tolyl)bicyclo[3.2.1] oct-2-en-8-one (4u) was prepared as a colorless oil according to the General Procedure A (eluent: *n*-hexane/EtOAc = 5:2, 156 mg, 92% yield, 93:7 er).

$[\alpha]_D^{23}$: –115.7 (*c* = 2.0, $CHCl_3$). HPLC analysis of the product: Daicel CHIRALPAK® AD-3 column; 10% *i*-PrOH in *n*-hexane; 1.0 mL/min; retention times: 8.2 min (major), 11.9 min (minor).

^{1}H NMR (400 MHz, $CDCl_3$) δ 7.34 – 7.28 (m, 2H), 7.27 – 7.22 (m, 3H), 7.14 (d, *J* = 8.0 Hz, 2H), 7.07 (d, *J* = 7.8 Hz, 2H), 5.20 (d, *J* = 7.5 Hz, 1H), 4.47 – 4.37 (m, 2H), 3.66 (s, 3H), 3.39 (dd, *J* = 9.4, 4.5 Hz, 1H), 3.26 – 3.16 (m, 4H), 3.10 (dd, *J* = 7.7, 1.6 Hz, 1H), 3.03 (dd, *J* = 7.7, 5.4 Hz, 1H), 2.91 (dd, *J* = 7.5, 1.6 Hz, 1H), 2.75 – 2.66 (m, 1H), 2.57 (s, 3H), 2.30 (s, 3H).

^{13}C NMR (101 MHz, $CDCl_3$) δ 206.6, 154.3, 137.8, 136.4, 136.3, 129.7, 128.2, 128.1, 127.39, 127.37, 102.6, 99.7, 73.0, 71.9, 56.2, 54.7, 50.5, 48.5, 47.4, 47.0, 41.8, 20.8.

HRMS (ESI) Calcd for $C_{26}H_{30}NaO_5$ $[M + Na]^+$: 445.1985, found: 445.1989.

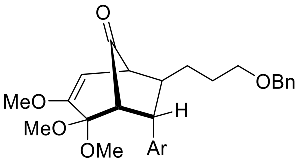

Ar = 4-$CH_3C_6H_4$
4v

(1*S*,5*S*,6*S*,7*S*)-7-(3-(Benzyloxy)propyl)-3,4,4-trimethoxy-6-(*p*-tolyl)bicyclo[3.2. 1]oct-2-en-8-one (4v) was prepared as a colorless oil according to the General Procedure A (eluent: *n*-hexane/EtOAc = 5:2, 165 mg, 92% yield, 94:6 er).

$[\alpha]_D^{23}$: –89.4 (*c* = 2.0, $CHCl_3$). HPLC analysis of the product: Daicel CHIRALPAK® AD-3 column; 10% *i*-PrOH in *n*-hexane; 1.0 mL/min; retention times: 7.0 min (major), 10.4 min (minor).

^{1}H NMR (400 MHz, $CDCl_3$) δ 7.34 – 7.28 (m, 2H), 7.27 – 7.23 (m, 3H), 7.13 (d, *J* = 7.9 Hz, 2H), 7.05 (d, *J* = 7.8 Hz, 2H), 5.15 (d, *J* = 7.5 Hz, 1H), 4.39 (s, 2H), 3.65 (s, 3H), 3.32 (t, *J* = 6.1 Hz, 2H), 3.18 (s, 3H), 3.08 (dd, *J* = 7.7, 1.6 Hz, 1H), 2.95 (dd, *J* = 7.7, 5.1 Hz, 1H), 2.63 (dd, *J* = 7.5, 1.6 Hz, 1H), 2.57 (s, 3H), 2.46 – 2.35 (m, 1H), 2.30 (s, 3H), 1.59 – 1.34 (m, 4H).

^{13}C NMR (101 MHz, $CDCl_3$) δ 207.1, 154.1, 138.3, 137.2, 136.1, 129.6, 128.3, 128.1, 127.4, 127.3, 102.5, 99.7, 72.7, 69.7, 56.3, 54.7, 50.4, 49.2, 48.2, 47.4, 46.6, 32.9, 27.6, 20.9.

HRMS (ESI) Calcd for $C_{28}H_{34}NaO_5$ $[M + Na]^+$: 473.2298, found: 473.2301.

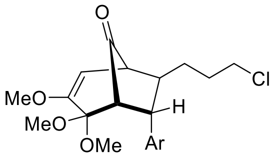

Ar = 4-$CH_3C_6H_4$
4w

(1*S*,5*S*,6*S*,7*S*)-7-(3-Chloropropyl)-3,4,4-trimethoxy-6-(*p*-tolyl)bicyclo[3.2.1] oct-2-en-8-one (4w) was prepared as a colorless oil according to the General Procedure A (eluent: *n*-hexane/EtOAc = 5:1, 150 mg, 95% yield, 93:7 er).

$[\alpha]_D^{23}$: –99.2 (*c* = 2.0, $CHCl_3$). HPLC analysis of the product: Daicel CHIRALPAK® AD-3 column; 10% *i*-PrOH in *n*-hexane; 1.0 mL/min; retention times: 5.9 min (major), 9.5 min (minor).

^{1}**H NMR** (400 MHz, $CDCl_3$) δ 7.16 (d, *J* = 7.6 Hz, 2H), 7.08 (d, *J* = 7.7 Hz, 2H), 5.21 (d, *J* = 7.4 Hz, 1H), 3.69 (s, 3H), 3.38 (s, 2H), 3.18 (s, 3H), 3.14 – 3.07 (m, 1H), 3.02 – 2.91 (m, 1H), 2.69 – 2.52 (m, 4H), 2.46 – 2.36 (m, 1H), 2.30 (s, 3H), 1.75 – 1.61 (m, 2H), 1.61 – 1.37 (m, 2H).

^{13}**C NMR** (101 MHz, $CDCl_3$) δ 206.7, 154.2, 136.8, 136.2, 129.5, 128.3, 102.4, 99.4, 56.2, 54.7, 50.4, 49.1, 47.6, 47.4, 46.4, 44.3, 33.3, 30.2, 20.8.

HRMS (ESI) Calcd for $C_{21}H_{27}NaClO_4$ $[M + Na]^+$: 401.1490, found: 401.1491.

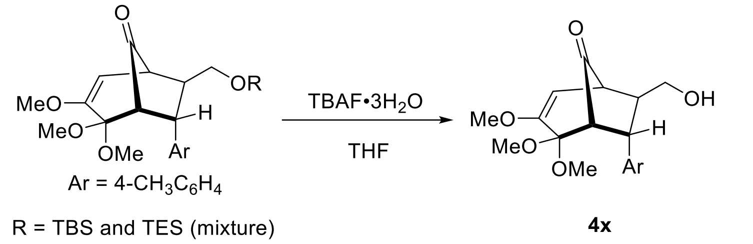

4x

(1*S*,5*S*,6*S*,7*S*)-7-(Hydroxymethyl)-3,4,4-trimethoxy-6-(*p*-tolyl)bicyclo[3.2.1] oct-2-en-8-one (4x) was prepared according to the General Procedure A from **2x**, except that the work-up procedure was modified. A mixture of the TBS and TES ethers was initially obtained. Then, TBAF•$3H_2O$ (0.8 mmol) was added to the mixture in THF (0.1 M), and the reaction was stirred at room temperature for 30 minutes. Water was subsequently added. The mixture was extracted with EtOAc, and the combined organic layers were concentrated and purified by column chromatography (*n*-hexane/EtOAc = 5:3) to yield the pure product **4x** as a colorless oil (109 mg, 82% yield, 91:9 er).

$[\alpha]_D^{23}$: –119.5 (*c* = 2.0, $CHCl_3$). HPLC analysis of the product: Daicel CHIRALPAK® IB N-3 column; 20% *i*-PrOH in *n*-hexane; 1.0 mL/min; retention times: 11.2 min (major), 13.0 min (minor).

^{1}**H NMR** (400 MHz, $CDCl_3$) δ 7.17 (d, *J* = 7.9 Hz, 2H), 7.08 (d, *J* = 7.8 Hz, 2H), 5.22 (d, *J* = 7.6 Hz, 1H), 3.69 (s, 3H), 3.58 (dd, *J* = 10.8, 4.6 Hz, 1H), 3.39 (dd, *J* = 10.8, 7.6 Hz, 1H), 3.19 (s, 3H), 3.14 – 3.06 (m, 2H), 2.89 (dd, *J* = 7.5, 1.5 Hz, 1H), 2.66 – 2.60 (m, 1H), 2.58 (s, 3H), 2.30 (s, 3H), 2.16 (br, 1H).

^{13}**C NMR** (101 MHz, $CDCl_3$) δ 207.5, 154.5, 136.6, 136.5, 129.9, 128.4, 102.7, 99.6, 64.4,

56.4, 54.9, 50.7, 50.6, 47.6, 46.9, 41.7, 20.9.

HRMS (ESI) Calcd for $C_{19}H_{24}NaO_5$ [M + Na]$^+$: 355.1516, found: 355.1523.

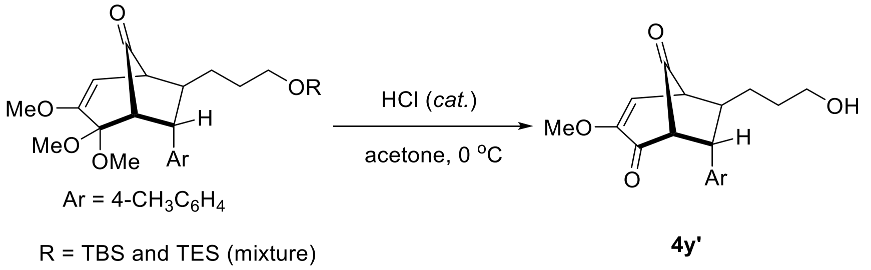

4y'

(1*S*,5*S*,6*S*,7*S*)-6-(3-Hydroxypropyl)-3-methoxy-7-(p-tolyl)bicyclo[3.2.1]oct-3-ene-2,8-dione (4y') was prepared according to the General Procedure A from **2y**, except that the work-up procedure was modified. A mixture of the TBS and TES ethers was initially obtained. Then, one drop of concentrated hydrochloride acid was then added to the mixture in acetone (0.1 M), and the reaction was stirred at 0 °C for 30 minutes. Water was subsequently added to quench the reaction. The mixture was extracted with EtOAc, and the organic layers were concentrated and purified by column chromatography (*n*-hexane/EtOAc = 1:1) to yield the pure product.

$[\alpha]_D^{23}$: –184.3 (c = 2.0, $CHCl_3$). HPLC analysis of the product: Daicel CHIRALPAK® IB N-3 column; 30% *i*-PrOH in *n*-hexane; 1.0 mL/min; retention times: 10.5 min (major), 16.7 min (minor).

^{1}H NMR (400 MHz, $CDCl_3$) δ 7.07 (d, J = 7.8 Hz, 2H), 6.92 (d, J = 7.9 Hz, 2H), 6.48 (d, J = 8.6 Hz, 1H), 3.73 (dd, J = 7.1, 2.1 Hz, 1H), 3.69 (s, 3H), 3.50 (t, J = 6.1 Hz, 2H), 3.24 (t, J = 6.5 Hz, 1H), 3.13 (dd, J = 8.6, 2.1 Hz, 1H), 2.43 (q, J = 6.9 Hz, 1H), 2.26 (s, 3H), 2.07 (br, 1H), 1.68 – 1.56 (m, 2H), 1.55 – 1.45 (m, 2H).

^{13}C NMR (101 MHz, $CDCl_3$) δ 200.3, 190.1, 154.2, 137.0, 134.9, 129.4, 127.9, 117.8, 70.6, 61.9, 55.6, 52.0, 47.7, 47.3, 32.3, 30.4, 20.8.

HRMS (ESI) Calcd for $C_{19}H_{22}NaO_4$ [M + Na]$^+$: 337.1410, found: 337.1420.

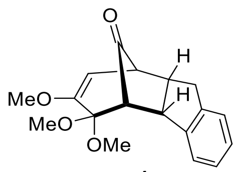

4z

(4b*S*,5*S*,9*S*,9a*R*)-6,6,7-Trimethoxy-4b,5,6,9,9a,10-hexahydro-5,9-methano benzo[a]azulen-11-one (4z) was prepared as a white solid according to the General Procedure A (eluent: *n*-hexane/EtOAc = 5:1, 111 mg, 95% yield, 92:8 er).

$[\alpha]_D^{23}$: –160.1 (c = 2.0, $CHCl_3$). HPLC analysis of the product: Daicel CHIRALPAK® ID-3 column; 10% *i*-PrOH in *n*-hexane; 1.0 mL/min; retention times: 14.4 min (minor), 16.3 min (major).

^{1}H NMR (400 MHz, $CDCl_3$) δ 7.27 – 7.23 (m, 1H), 7.13 – 7.06 (m, 3H), 4.79 (d, J = 6.8 Hz, 1H), 3.97 (t, J = 9.1 Hz, 1H), 3.36 (s, 3H), 3.30 (dd, J = 8.4, 1.5 Hz, 1H), 3.27 (s, 3H), 3.16 (s, 3H), 3.12 – 3.01 (m, 2H), 2.99 – 2.90 (m, 1H), 2.84 (ddd, J = 7.0, 5.6, 1.4 Hz, 1H).

^{13}C NMR (101 MHz, $CDCl_3$) δ 206.3, 154.3, 144.2, 140.2, 126.7, 126.5, 125.3, 123.3, 102.7, 94.2, 54.6, 52.1, 50.2, 48.3, 46.9, 44.4, 39.0, 32.7.

HRMS (ESI) Calcd for $C_{18}H_{20}NaO_4$ $[M + Na]^+$: 323.1254, found: 323.1262.

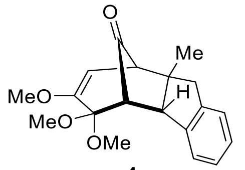

4aa

(4b*R*,5*S*,9*R*,9a*R*)-6,6,7-Trimethoxy-9a-methyl-4b,5,6,9,9a,10-hexahydro-5,9-methanobenzo[a]azulen-11-one (4aa) was prepared as a white solid according to the General Procedure A (eluent: *n*-hexane/EtOAc = 5:1, 100 mg, 80% yield, 89:11 er).

$[\alpha]_D^{23}$: –123.7 (c = 2.0, $CHCl_3$). HPLC analysis of the product: Daicel CHIRALPAK® IB N-3 column; 10% *i*-PrOH in *n*-hexane; 1.0 mL/min; retention times: 8.3 min (major), 11.7 min (minor).

^{1}H NMR (400 MHz, $CDCl_3$) δ 7.26 – 7.19 (m, 1H), 7.16 – 7.09 (m, 2H), 7.09 – 7.02 (m, 1H), 4.86 (d, J = 7.0 Hz, 1H), 3.44 (d, J = 8.3 Hz, 1H), 3.38 – 3.31 (m, 5H), 3.29 (s, 3H), 3.17 (s, 3H), 2.79 (d, J = 17.0 Hz, 1H), 2.54 (d, J = 6.9 Hz, 1H), 1.23 (s, 3H).

^{13}**C NMR** (101 MHz, $CDCl_3$) δ 206.5, 153.8, 143.8, 140.3, 126.9, 126.5, 125.4, 123.3, 102.6, 95.9, 54.6, 53.9, 53.4, 53.0, 50.4, 48.4, 46.2, 41.4, 27.9.

HRMS (ESI) Calcd for $C_{19}H_{22}NaO_4$ $[M + Na]^+$: 337.1410, found: 337.1420.

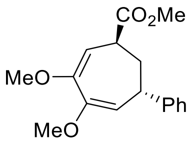

5a

Methyl (1*S*,6*S*)-3,4-dimethoxy-6-phenylcyclohepta-2,4-diene-1-carboxylate (5a) was prepared as a colorless oil according to the General Procedure C (eluent: *n*-hexane/EtOAc = 5:1, 92 mg, 80% yield, 95:5 er).

$[\alpha]_D^{23}$: +68.4 (c = 0.5, $CHCl_3$). HPLC analysis of the product: Daicel CHIRALPAK® AD-3 column; 10% *i*-PrOH in *n*-hexane; 1.0 mL/min; retention times: 5.9 min (major), 6.9 min (minor).

^{1}**H NMR** (400 MHz, $CDCl_3$) δ 7.36 – 7.27 (m, 4H), 7.25 – 7.18 (m, 1H), 5.56 (d, J = 5.9 Hz, 1H), 5.26 (d, J = 5.9 Hz, 1H), 3.72 (s, 3H), 3.70 (s, 3H), 3.61 (s, 3H), 3.35 (dt, J = 12.9, 6.6 Hz, 1H), 3.24 (ddd, J = 12.9, 7.4, 5.8 Hz, 1H), 2.52 (td, J = 12.7, 7.4 Hz, 1H), 2.34 (td, J = 12.7, 7.2 Hz, 1H).

^{13}**C NMR** (101 MHz, $CDCl_3$) δ 175.2, 151.6, 151.1, 144.5, 128.6, 127.8, 126.4, 107.1, 100.6, 55.2, 55.0, 52.0, 49.3, 41.8, 41.7.

HRMS (ESI) Calcd for $C_{17}H_{20}NaO_4$ $[M + Na]^+$: 311.1254, found: 311.1254.

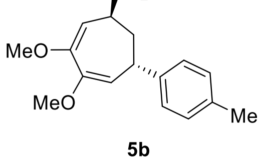

5b

Methyl (1*S*,6*S*)-3,4-dimethoxy-6-(*p*-tolyl)cyclohepta-2,4-diene-1-carboxylate (5b) was prepared as a colorless oil according to the General Procedure C (eluent: *n*-hexane/EtOAc = 5:1, 98 mg, 81% yield, 97:3 er).

$[\alpha]_D^{23}$: +79.2 (c = 0.5, $CHCl_3$). HPLC analysis of the product: Daicel CHIRALPAK® AD-

3 column; 10% *i*-PrOH in *n*-hexane; 1.0 mL/min; retention times: 5.9 min (major), 7.2 min (minor).

^{1}H NMR (400 MHz, $CDCl_3$) δ 7.19 (d, *J* = 8.1 Hz, 2H), 7.13 (d, *J* = 8.0 Hz, 2H), 5.55 (d, *J* = 5.9 Hz, 1H), 5.23 (d, *J* = 5.9 Hz, 1H), 3.72 (s, 3H), 3.70 (s, 3H), 3.60 (s, 3H), 3.32 (dt, *J* = 12.8, 6.6 Hz, 1H), 3.22 (ddd, *J* = 12.9, 7.4, 5.8 Hz, 1H), 2.50 (td, *J* = 12.7, 7.4 Hz, 1H), 2.37 – 2.25 (m, 4H).

^{13}C NMR (101 MHz, $CDCl_3$) δ 175.2, 151.6, 150.9, 141.5, 136.0, 129.3, 127.7, 107.3, 100.5, 55.1, 55.0, 52.0, 49.4, 41.7, 41.4, 20.9.

HRMS (ESI) Calcd for $C_{18}H_{22}NaO_4$ $[M + Na]^+$: 325.1410, found: 325.1423.

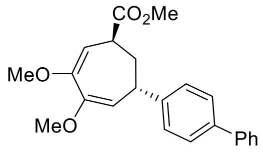

5e

Methyl (1*S*,6*S*)-6-([1,1'-biphenyl]-4-yl)-3,4-dimethoxycyclohepta-2,4-diene-1-carboxylate (5e) was prepared as a colorless oil according to the General Procedure C (eluent: *n*-hexane/EtOAc = 5:1, 124 mg, 85% yield, 95:5 er).

$[\alpha]_D^{23}$: +74.7 (*c* = 1.0, $CHCl_3$). HPLC analysis of the product: Daicel CHIRALPAK® AD-3 column; 10% *i*-PrOH in *n*-hexane; 1.0 mL/min; retention times: 7.8 min (major), 11.2 min (minor).

^{1}H NMR (400 MHz, $CDCl_3$) δ 7.62 – 7.52 (m, 4H), 7.46 – 7.40 (m, 2H), 7.40 – 7.30 (m, 3H), 5.58 (d, *J* = 5.8 Hz, 1H), 5.28 (d, *J* = 5.9 Hz, 1H), 3.74 (s, 3H), 3.71 (s, 3H), 3.62 (s, 3H), 3.40 (dt, *J* = 12.8, 6.6 Hz, 1H), 3.26 (ddd, *J* = 12.9, 7.4, 5.8 Hz, 1H), 2.56 (td, *J* = 12.7, 7.4 Hz, 1H), 2.38 (td, *J* = 12.7, 7.1 Hz, 1H).

^{13}C NMR (101 MHz, $CDCl_3$) δ 175.2, 151.6, 151.1, 143.6, 140.8, 139.4, 128.7, 128.2, 127.3, 127.2, 127.0, 106.9, 100.6, 55.2, 55.0, 52.0, 49.3, 41.7, 41.5.

HRMS (ESI) Calcd for $C_{23}H_{24}NaO_4$ $[M + Na]^+$: 387.1567, found: 387.1573.

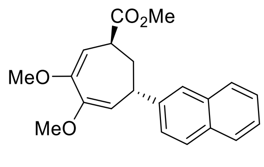

5h

Methyl (1*S*,6*S*)-3,4-dimethoxy-6-(naphthalen-2-yl)cyclohepta-2,4-diene-1-carboxylate (5h) was prepared as a colorless oil according to the General Procedure C (eluent: *n*-hexane/EtOAc = 5:1, 95 mg, 70% yield, 97:3 er).

$[\alpha]_D^{23}$: +72.2 (*c* = 1.0, $CHCl_3$). HPLC analysis of the product: Daicel CHIRALPAK® AD-3 column; 10% *i*-PrOH in *n*-hexane; 1.0 mL/min; retention times: 7.1 min (major), 9.1 min (minor).

^{1}H NMR (400 MHz, $CDCl_3$) δ 7.88 – 7.76 (m, 3H), 7.72 (s, 1H), 7.51 – 7.37 (m, 3H), 5.60 (d, *J* = 5.8 Hz, 1H), 5.33 (d, *J* = 5.9 Hz, 1H), 3.75 (s, 3H), 3.71 (s, 3H), 3.62 (s, 3H), 3.53 (dt, *J* = 12.8, 6.5 Hz, 1H), 3.29 (dt, *J* = 12.8, 6.9 Hz, 1H), 2.65 (td, *J* = 12.7, 7.4 Hz, 1H), 2.42 (td, *J* = 12.6, 7.1 Hz, 1H).

^{13}C NMR (101 MHz, $CDCl_3$) δ 175.2, 151.6, 151.1, 141.8, 133.5, 132.2, 128.3, 127.6 (2C), 126.4, 126.1, 126.0, 125.5, 106.9, 100.6, 55.2, 55.1, 52.0, 48.9, 41.9, 41.8.

HRMS (ESI) Calcd for $C_{21}H_{22}NaO_4$ $[M + Na]^+$: 361.1410, found: 361.1415.

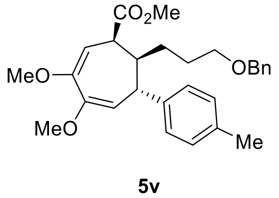

5v

Methyl (1*S*,6*S*,7*S*)-7-(3-(benzyloxy)propyl)-3,4-dimethoxy-6-(*p*-tolyl)cyclohepta- 2,4-diene-1-carboxylate (5v) was prepared as a colorless oil according to the General Procedure C (eluent: *n*-hexane/EtOAc = 4:1, 117 mg, 65% yield, 97:3 er).

$[\alpha]_D^{23}$: +10.3 (*c* = 1.0, $CHCl_3$). HPLC analysis of the product: Daicel CHIRALPAK® AD-3 column; 10% *i*-PrOH in *n*-hexane; 1.0 mL/min; retention times: 5.0 min (major), 6.0 min (minor).

^{1}H NMR (400 MHz, $CDCl_3$) δ 7.34 – 7.28 (m, 2H), 7.28 – 7.26 (m, 1H), 7.25 – 7.21 (m, 2H), 7.17 (d, *J* = 8.1 Hz, 2H), 7.11 (d, *J* = 7.9 Hz, 2H), 5.64 (d, *J* = 6.7 Hz, 1H), 5.10 (d, *J* =

5.5 Hz, 1H), 4.33 (s, 2H), 3.73 (s, 3H), 3.69 (s, 3H), 3.55 (s, 3H), 3.44 (t, *J* = 6.3 Hz, 1H), 3.25 – 3.12 (m, 2H), 2.99 (dd, *J* = 11.2, 5.5 Hz, 1H), 2.95 – 2.84 (m, 1H), 2.33 (s, 3H), 1.42 – 1.20 (m, 4H).

^{13}C NMR (101 MHz, $CDCl_3$) δ 174.6, 151.3, 150.5, 141.5, 138.6, 136.0, 129.3, 128.4, 128.2, 127.5, 127.4, 108.5, 100.2, 72.5, 70.5, 58.8, 55.2, 55.0, 51.7, 47.8, 46.7, 26.8, 26.2, 21.0.

HRMS (ESI) Calcd for $C_{28}H_{34}NaO_5$ $[M + Na]^+$: 473.2298, found: 473.2304.

VI. Product Derivatizations

(a) Semi-gram synthesis of 4b

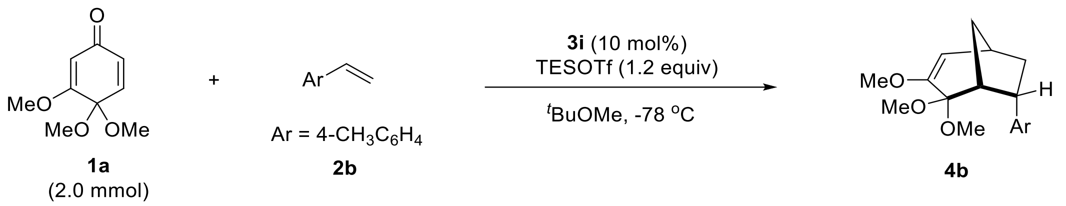

An oven-dried 100-mL round-bottom flask was charged with **1a** (368.4 mg, 2.0 mmol, 1.0 equiv), **2b** (283.7 mg, 2.4 mmol, 1.2 equiv), catalyst **3i** (142.0 mg, 0.2 mmol, 10 mol%), and dry ^{t}BuOMe (0.1 M, 20 mL). The flask was sealed, and the reaction mixture was cooled to –78 °C in a low temperature reactor. TESOTf (545.0 μL, 2.4 mmol, 1.2 equiv) was added via syringe. The reaction was stirred for 48 hours and then quenched by the addition of MeOH/Et_3N (2.0 mL, v/v = 3:1) via syringe and allowed to stir at –78 °C for 5 minutes before warming to room temperature. The crude reaction mixture was concentrated and purified by silica gel flash chromatography (*n*-hexane/EtOAc = 10:1→5:2) to afford the desired product **4b** as a pale solid (0.53 g, 88% yield, 97:3 er).

Note: The catalyst was recovered in 96% yield, and the recovered catalyst showed similarly good catalytic activity (88% yield and 96:4 er) for the standard reaction.

(b) Derivatization of the perezone-type [5+2] cycloaddition products

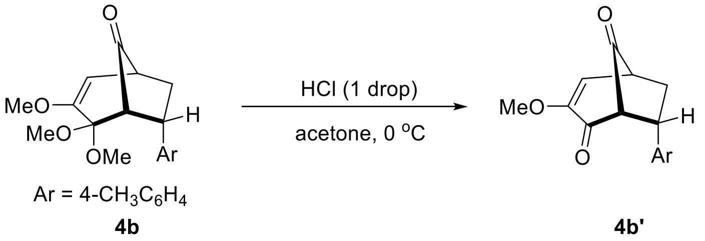

(1*S*,5*S*,7*S*)-3-Methoxy-7-(*p*-tolyl)bicyclo[3.2.1]oct-3-ene-2,8-dione (4b'). At 0 °C, a solution of the cycloadduct **4b** (59.0 mg, 0.2 mmol, 1.0 equiv) in acetone (2 mL, 0.1 M) was treated with one drop of aqueous HCl solution (1 N). The mixture was stirred for 30 minutes and then concentrated under reduced pressure. The resulting residue was

purified by silica gel column chromatography (n-hexane/EtOAc = 5:1 to 5:3) to afford **4b'** as a white solid (45.0 mg, 88% yield, 97:3 er).

$[\alpha]_D^{23}$: –285.72 (c = 0.5, $CHCl_3$). HPLC analysis of the product: Daicel CHIRALPAK® IB N-3 column; 30% i-PrOH in n-hexane; 1.0 mL/min; retention times: 10.3 min (major), 14.7 min (minor).

^{1}H NMR (400 MHz, $CDCl_3$) δ 7.09 (d, J = 8.0 Hz, 2H), 6.96 (d, J = 8.1 Hz, 2H), 6.47 (d, J = 8.5 Hz, 1H), 3.88 – 3.75 (m, 2H), 3.70 (s, 3H), 3.43 – 3.34 (m, 1H), 2.75 (ddd, J = 13.6, 10.3, 6.6 Hz, 1H), 2.28 (s, 3H), 2.18 (dd, J = 13.5, 5.9 Hz, 1H).

^{13}C NMR (101 MHz, $CDCl_3$) δ 200.5, 190.0, 154.9, 136.9, 135.2, 129.4, 127.8, 118.6, 69.4, 55.7, 46.3, 38.7, 33.2, 20.8.

HRMS (ESI) Calcd for $C_{16}H_{16}NaO_3$ [M + Na]$^+$: 279.0992, found: 279.1006.

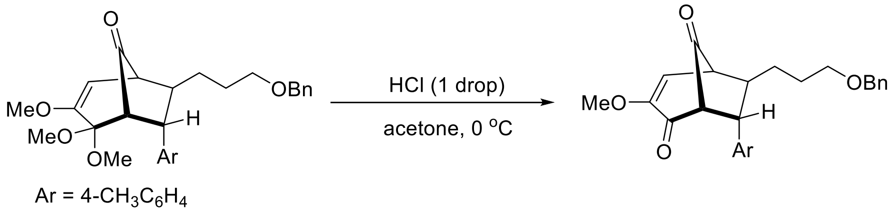

4v 4v'

(1*S*,5*S*,6*S*,7*S*)-6-(3-(Benzyloxy)propyl)-3-methoxy-7-(*p*-tolyl)bicyclo[3.2.1]oct-3-ene-2,8-dione (4v') was prepared as a colorless oil (190.0 mg, 85% yield, 94:6 er) from **4v** (165.0 mg, 0.37 mmol) according to the same procedure for the preparation of **4b'**.

$[\alpha]_D^{23}$: –189.9 (c = 1.0, $CHCl_3$). HPLC analysis of the product: Daicel CHIRALPAK® AD-3 column; 30% i-PrOH in n-hexane; 1.0 mL/min; retention times: 6.9 min (major), 8.6 min (minor).

^{1}H NMR (400 MHz, $CDCl_3$) δ 7.35 – 7.22 (m, 5H), 7.05 (d, J = 7.8 Hz, 2H), 6.90 (d, J = 7.9 Hz, 2H), 6.40 (d, J = 8.6 Hz, 1H), 4.42 (d, J = 2.4 Hz, 2H), 3.72 (dd, J = 7.0, 2.1 Hz, 1H), 3.64 (s, 3H), 3.42 – 3.32 (m, 2H), 3.22 (t, J = 6.6 Hz, 1H), 3.11 (dd, J = 8.6, 2.1 Hz, 1H), 2.42 (q, J = 6.7 Hz, 1H), 2.26 (s, 3H), 1.68 – 1.51 (m, 4.0 Hz, 4H).

^{13}C NMR (101 MHz, $CDCl_3$) δ 200.1, 190.0, 154.1, 138.1, 136.9, 134.9, 129.4, 128.2, 127.9, 127.45, 127.41, 117.8, 72.8, 70.6, 69.5, 55.5, 51.9, 47.7, 47.3, 32.7, 27.6, 20.8.

HRMS (ESI) Calcd for $C_{26}H_{28}NaO_4$ [M + Na]$^+$: 427.1880, found: 427.1881.

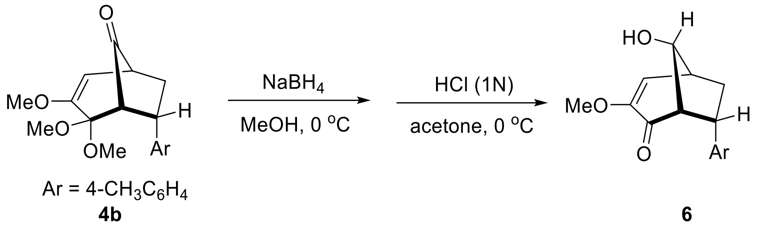

(1*S*,5*S*,7*S*,8*R*)-8-Hydroxy-3-methoxy-7-(*p*-tolyl)bicyclo[3.2.1]oct-3-en-2-one (6). At 0 °C, to a solution of the cycloadduct **4b** (30.2 mg, 0.1 mmol, 1.0 equiv) in MeOH (1.0 mL, 0.1 M) was treated with sodium borohydride (3.8 mg, 0.1 mmol, 1.0 equiv). The mixture was stirred for 30 minutes at the same temperature and then quenched with a saturated aqueous solution of NH_4Cl (2 mL). The organic layer was separated, and the aqueous layer was extracted with EtOAc (3 x 5 mL). The combined organic layers were concentrated, and the residue was dissolved in acetone. Several drops of aqueous HCl solution (1 N) were added to the mixture at 0 °C, and the mixture was stirred for an additional 0.5 hours. The reaction was then concentrated and purified by silica gel flash chromatography (*n*-hexane/EtOAc = 5:1 to 5:3), yielding alcohol **6** as a white powder (21.0 mg, 80% yield, dr >20:1, 99:1 er). The product stereochemistry was assigned by analogy to that of **7**, which was confirmed by NOESY analysis.

$[\alpha]_D^{23}$: –15.4 (*c* = 0.5, $CHCl_3$). HPLC analysis of the product: Daicel CHIRALPAK® ID-3 column; 20% *i*-PrOH in *n*-hexane; 1.0 mL/min; retention times: 22.1 min (minor), 22.8 min (major).

^{1}H NMR (400 MHz, $CDCl_3$) δ 7.03 (d, *J* = 7.7 Hz, 2H), 6.94 (d, *J* = 7.9 Hz, 2H), 6.00 (d, *J* = 6.0 Hz, 1H), 4.37 (t, *J* = 4.0 Hz, 1H), 3.72 (dt, *J* = 11.5, 5.9 Hz, 1H), 3.65 (s, 3H), 3.31 – 3.22 (m, 1H), 3.07 (q, *J* = 5.8 Hz, 1H), 2.57 – 2.50 (m, 1H), 2.25 (s, 3H), 2.07 (dd, *J* = 13.5, 5.3 Hz, 1H), 1.73 (br, 1H).

^{13}C NMR (101 MHz, $CDCl_3$) δ 194.0, 153.7, 136.7, 136.2, 129.2, 127.7, 115.7, 79.9, 63.0, 55.0, 40.7, 40.6, 34.1, 20.9.

HRMS (ESI) Calcd for $C_{16}H_{18}NaO_3$ $[M + Na]^+$: 281.1148, found: 281.1150.

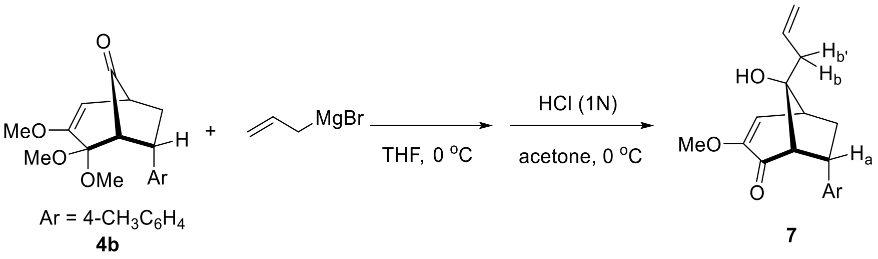

(1*S*,5*S*,7*S*,8*R*)-8-Allyl-8-hydroxy-3-methoxy-7-(*p*-tolyl)bicyclo[3.2.1]oct-3-en-2-one (7). At 0 °C under nitrogen, a solution of the cycloadduct **4b** (45.4 mg, 0.15 mmol, 1.0 equiv) in THF (1.5 mL, 0.1 M) was treated with allylmagnesium bromide (1.0 M, 0.45 mL, 0.45 mmol, 3.0 equiv). The mixture was stirred for 2.5 hours at the same temperature and then quenched with water (2 mL). The organic layer was separated, and the aqueous layer was extracted with EtOAc (3 x 5 mL). The combined organic layers were concentrated, and the residue was dissolved in acetone. Several drops of 1N HCl were added to the solution at 0 °C, and the mixture was stirred for additional 30 minutes. The reaction was then concentrated and purified by silica gel flash chromatography (*n*-hexane/EtOAc = 5:1 to 5:3) to afford alcohol **7** as a white powder (44.0 mg, 98% yield, dr >20:1, 97:3 er). The relative stereochemistry was confirmed by the observation of the nOe NMR experiment, which indicated correlation between H_a and H_b in the two-dimensional spectrum.

$[\alpha]_D^{23}$: –37.0 (*c* = 1.0, $CHCl_3$). HPLC analysis of the product: Daicel CHIRALPAK® IB N-3 column; 20% *i*-PrOH in *n*-hexane; 1.0 mL/min; retention times: 12.0 min (major), 17.6 min (minor).

^{1}H NMR (400 MHz, $CDCl_3$) δ 7.02 (d, *J* = 7.8 Hz, 2H), 6.93 (d, *J* = 7.8 Hz, 2H), 6.03 – 5.86 (m, 2H), 5.29 – 5.15 (m, 2H), 3.73 (t, *J* = 5.7 Hz, 1H, H_a), 3.61 (s, 3H), 3.08 (d, *J* = 7.1 Hz, 1H), 2.87 (t, *J* = 6.9 Hz, 1H), 2.57 – 2.38 (m, 3H, including H_b+$H_{b'}$), 2.24 (s, 3H), 2.09 (dd, *J* = 13.8, 4.8 Hz, 1H).

^{13}C NMR (101 MHz, $CDCl_3$) δ 194.6, 154.1, 136.5, 136.1, 132.0, 129.1, 127.7, 119.6, 116.3, 84.9, 65.2, 55.0, 43.5, 41.5, 40.9, 33.7, 20.8.

HRMS (ESI) Calcd for $C_{19}H_{22}NaO_3$ $[M + Na]^+$: 321.1461, found: 321.1460.

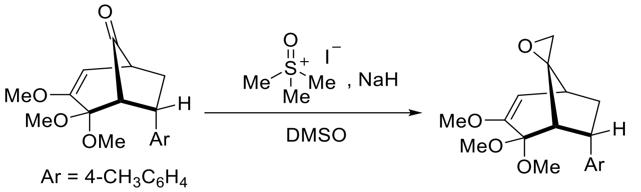

4b **8**

(1*S*,5*S*,6*S*,8*R*)-3,4,4-Trimethoxy-6-(*p*-tolyl)spiro[bicyclo[3.2.1]octane-8,2'-oxiran]-2-ene (8). At 0 °C, an oven-dried 4-mL vial was charged with trimethylsulphoxonium iodide (26.4 mg, 0.12 mmol, 1.2 equiv) and anhydrous DMSO (1.0 mL, 0.1 M). NaH (60 wt% in mineral oil, 6.0 mg, 0.15 mmol, 1.5 equiv) was added in one portion. The resulting white suspension was stirred at 0 °C for 5 minutes and then at room temperature for additional 30 minutes. Next, cycloadduct **4b** (30.2 mg, 0.1 mmol, 1.0 equiv) was added in one portion at 0 °C. After stirring for 1 hour at room temperature, the mixture was cooled to 0 °C and quenched with water (2 mL). The organic layer was separated, and the aqueous layer was extracted with EtOAc (3 x 2 mL). The combined organic layers were dried over $MgSO_4$, filtered, and concentrated under reduced pressure. The crude residue was purified by silica gel column chromatography (*n*-hexane/EtOAc = 15:1 to 6:1) to afford colorless cyclopropane **8** (25.0 mg, 80% yield, dr >20:1, 96:4 er). The stereochemistry of the product was assigned by analogy to that of **7**, which was confirmed by NOESY analysis.

$[\alpha]_D^{23}$: –73.0 (*c* = 0.3, CH_2Cl_2). HPLC analysis of the product: Daicel CHIRALPAK® IB N-3 column; 5% *i*-PrOH in *n*-hexane; 1.0 mL/min; retention times: 5.1 min (minor), 5.6 min (major).

^{1}H NMR (400 MHz, CD_2Cl_2) δ 7.05 (d, *J* = 7.7 Hz, 2H), 6.93 (d, *J* = 7.8 Hz, 2H), 5.04 (d, *J* = 7.2 Hz, 1H), 3.60 (dt, *J* = 11.9, 6.2 Hz, 1H), 3.51 (s, 3H), 3.03 (s, 3H), 2.98 (d, *J* = 4.5 Hz, 1H), 2.86 (d, *J* = 4.5 Hz, 1H), 2.43 (dt, *J* = 11.7, 5.9 Hz, 1H), 2.36 (s, 3H), 2.24 – 2.17 (m, 4H), 2.01 (t, *J* = 6.0 Hz, 1H), 1.86 (dd, *J* = 12.5, 5.0 Hz, 1H).

^{13}C NMR (101 MHz, CD_2Cl_2) δ 155.1, 139.6, 135.8, 130.3, 128.1, 102.3, 101.8, 70.2, 54.7, 52.02, 51.99, 50.4, 47.4, 43.3, 39.9, 38.9, 21.1.

HRMS (ESI) Calcd for $C_{19}H_{24}NaO_4$ $[M + Na]^+$: 339.1567, found: 339.1563.

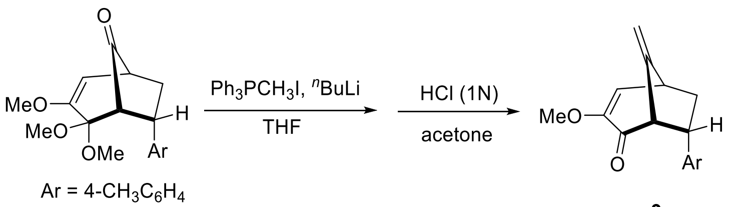

(1*S*,5*S*,7*S*)-3-Methoxy-8-methylene-7-(*p*-tolyl)bicyclo[3.2.1]oct-3-en-2-one (9). At 0 °C under nitrogen, to an oven-dried 10-mL Schlenk tube charged with Ph_3PCH_3I (107.2 mg, 0.3 mmol, 3.0 equiv) and dried THF (1.0 mL, 0.1 M) was added dropwise ^{n}BuLi (1.6 M, 0.32 mL, 0.5 mmol, 5.0 equiv). The mixture became clear and turned yellow. Next, the cycloadduct **4b** (30.2 mg, 0.1 mmol, 1.0 equiv) was added in one portion at 0 °C. After stirring overnight, the mixture was quenched with a saturated aqueous NH_4Cl solution (2 mL). The organic layer was separated, and the aqueous layer was extracted with EtOAc (3 x 2 mL). The combined organic layers were concentrated, and the residue was dissolved in acetone. Several drops of an aqueous solution of HCl (1N) were added at 0 °C, and the mixture was stirred for additional 30 minutes. The reaction was concentrated and purified by silica gel flash chromatography (*n*-hexane/EtOAc = 8:1 to 5:1) to give ketone **9** as a white solid (16.0 mg, 60% yield, 99:1 er).

$[\alpha]_D^{23}$: –20.4 (*c* = 0.2, $CHCl_3$). HPLC analysis of the product: Daicel CHIRALPAK® ID-3 column; 20% *i*-PrOH in *n*-hexane; 1.0 mL/min; retention times: 9.6 min (major), 11.7 min (minor).

^{1}H NMR (400 MHz, $CDCl_3$) δ 7.04 (d, *J* = 7.9 Hz, 2H), 6.97 (d, *J* = 8.2 Hz, 2H), 6.35 (d, *J* = 7.7 Hz, 1H), 4.74 (s, 1H), 4.71 (s, 1H), 3.82 – 3.72 (m, 1H), 3.69 (dd, *J* = 7.1, 1.7 Hz, 1H), 3.61 (s, 3H), 3.40 (ddd, *J* = 7.8, 6.1, 1.7 Hz, 1H), 2.57 (ddd, *J* = 13.0, 10.5, 6.2 Hz, 1H), 2.26 (s, 3H), 2.08 (dd, *J* = 12.9, 5.2 Hz, 1H).

^{13}C NMR (101 MHz, $CDCl_3$) δ 194.3, 153.6, 152.8, 136.8, 136.3, 129.2, 127.8, 121.6, 102.1, 65.1, 55.1, 43.7, 42.3, 36.4, 20.9.

HRMS (ESI) Calcd for $C_{17}H_{18}NaO_2$ $[M + Na]^+$: 277.1199, found: 277.1203.

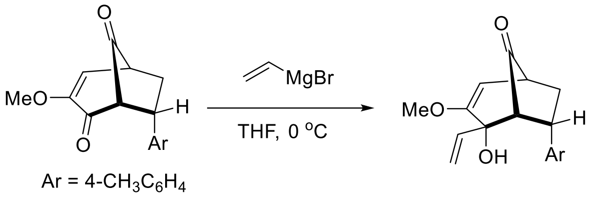

4b'   10

(1*S*,4*S*,5*R*,6*S*)-4-Hydroxy-3-methoxy-6-(*p*-tolyl)-4-vinylbicyclo[3.2.1]oct-2-en-8-one (10). At 0 °C under nitrogen, a solution of the cycloadduct **4b'** (25.6 mg, 0.1 mmol, 1.0 equiv) in THF (1.0 mL, 0.1 M) was treated with vinylmagnesium bromide (1.0 M, 0.3 mL, 0.3 mmol, 3.0 equiv). The mixture was stirred for 30 minutes at the same temperature and then quenched with a saturated aqueous NH_4Cl solution (2 mL). The organic layer was separated, and the aqueous layer was extracted with EtOAc (3 x 5 mL). The combined organic layers were dried over $MgSO_4$, filtered, and concentrated under reduced pressure. The residue was purified by silica gel column chromatography (*n*-hexane/EtOAc = 8:1 to 5:1) to afford the alcohol **10** as a colorless oil (21.0 mg, 75% yield, dr >20:1, 96:4 er). The stereochemistry of the product was assigned by analogy to the literature.[11]

$[\alpha]_D^{23}$: –62.6 (*c* = 0.5, $CHCl_3$). HPLC analysis of the product: Daicel CHIRALPAK® IB N-3 column; 20% *i*-PrOH in *n*-hexane; 1.0 mL/min; retention times: 6.2 min (major), 7.2 min (minor).

^{1}H NMR (400 MHz, $CDCl_3$) δ 7.29 (d, *J* = 8.0 Hz, 2H), 7.19 (d, *J* = 8.0 Hz, 2H), 5.74 (dd, *J* = 16.9, 10.5 Hz, 1H), 5.17 – 5.08 (m, 3H), 3.78 – 3.69 (m, 1H), 3.63 (s, 3H), 2.88 – 2.82 (m, 1H), 2.79 (dd, *J* = 7.4, 1.8 Hz, 1H), 2.47 – 2.39 (m, 2H), 2.33 (s, 3H), 1.75 (s, 1H).

^{13}C NMR (101 MHz, $CDCl_3$) δ 207.5, 156.2, 138.7, 137.9, 137.3, 129.9, 129.1, 114.8, 98.4, 81.1, 60.5, 55.4, 44.2, 39.2, 34.7, 21.0.

HRMS (ESI) Calcd for $C_{18}H_{20}NaO_3$ $[M + Na]^+$: 307.1305, found: 307.1304.

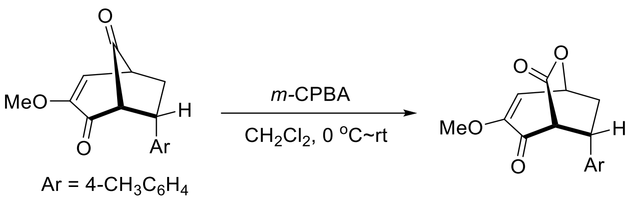

(1*R*,5*S*,8*S*)-3-Methoxy-8-(*p*-tolyl)-6-oxabicyclo[3.2.2]non-3-ene-2,7-dione (11). At 0 °C, to a solution of the cycloadduct **4b'** (38.5 mg, 0.15 mmol, 1.0 equiv) in CH_2Cl_2 (1.5 mL, 0.1 M) was added *m*-CPBA (80% purity, 65.0 mg, 0.3 mmol, 2.0 equiv). The mixture was allowed to warm to room temperature and stirred overnight. The reaction was quenched with a saturated aqueous solution of $NaHCO_3$ (2 mL). The organic layer was separated, and the aqueous layer was extracted with EtOAc (3 x 5 mL). The combined organic layers were dried over $MgSO_4$, filtered, and concentrated under reduced pressure. The residue was purified by silica gel column chromatography (*n*-hexane/EtOAc = 5:1 to 5:3) to afford white solid **11** (35.0 mg, 85% yield, 97:3 er).

$[\alpha]_D^{23}$: –126.2 (*c* = 0.5, $CHCl_3$). HPLC analysis of the product: Daicel CHIRALCEL® OD-3 column; 50% *i*-PrOH in *n*-hexane; 1.0 mL/min; retention times: 7.0 min (major), 10.1 min (minor).

^{1}H NMR (400 MHz, $CDCl_3$) δ 7.10 (d, *J* = 7.9 Hz, 2H), 6.95 (d, *J* = 8.2 Hz, 2H), 6.32 (d, *J* = 8.9 Hz, 1H), 5.43 (dd, *J* = 8.7, 6.4 Hz, 1H), 4.15 (d, *J* = 5.7 Hz, 1H), 3.75 (dt, *J* = 10.7, 5.3 Hz, 1H), 3.68 (s, 3H), 2.93 (ddd, *J* = 14.7, 10.8, 6.4 Hz, 1H), 2.43 (dd, *J* = 14.7, 4.9 Hz, 1H), 2.28 (s, 3H).

^{13}C NMR (101 MHz, $CDCl_3$) δ 184.9, 168.1, 155.9, 137.5, 134.9, 129.7, 127.3, 115.9, 71.5, 65.5, 55.6, 36.4, 32.9, 20.9.

HRMS (ESI) Calcd for $C_{16}H_{16}NaO_4$ $[M + Na]^+$: 295.0941, found: 295.0935.

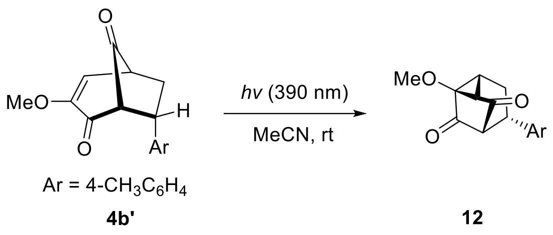

(1*S*,2*R*,4*S*,5*S*)-1-Methoxy-4-(*p*-tolyl)tricyclo[3.2.1.0^{2,7}]octane-6,8-dione (12). In an glove box, MeCN (2 mL, 0.05 M) was added to a 4-mL vial charged with the cycloadduct **4b'** (25.6 mg, 0.1 mmol). The vial was sealed and removed from the glove box. The reaction mixture was stirred overnight under 390 nm light. Then, the mixture was concentrated and purified by silica gel column chromatography (*n*-hexane/EtOAc = 8:1 to 5:2) to yield ketone **11** as a colorless oil (19.0 mg, 75% yield, 97:3 er). The stereochemistry of **12** was assigned by analogy to the literature.[12]

$[\alpha]_D^{23}$: +95.9 (*c* = 1.0, $CHCl_3$). HPLC analysis of the product: Daicel CHIRALPAK® ID-3 column; 30% *i*-PrOH in *n*-hexane; 1.0 mL/min; retention times: 15.0 min (minor), 22.3 min (major).

^{1}H NMR (400 MHz, $CDCl_3$) δ 7.12 (d, *J* = 7.8 Hz, 2H), 7.02 (d, *J* = 7.7 Hz, 2H), 3.68 – 3.63 (m, 1H), 3.61 (s, 3H), 3.08 (d, *J* = 8.9 Hz, 1H), 2.94 (d, *J* = 9.0 Hz, 1H), 2.74 (s, 1H), 2.63 (ddd, *J* = 13.6, 10.3, 2.5 Hz, 1H), 2.37 – 2.28 (m, 4H).

^{13}C NMR (101 MHz, $CDCl_3$) δ 200.4, 199.8, 138.2, 137.3, 129.5, 126.8, 83.8, 62.2, 58.1, 50.4, 48.4, 43.2, 26.8, 20.9.

HRMS (ESI) Calcd for $C_{16}H_{16}NaO_3$ $[M + Na]^+$: 279.0992, found: 279.0997.

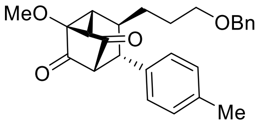

13

(1*S*,2*R*,3*S*,4*S*,5*S*)-3-(3-(Benzyloxy)propyl)-1-methoxy-4-(*p*-tolyl)tricyclo[3.2.1.0^{2,7}] octane-6,8-dione (13) was prepared as a colorless oil (19 mg, 80% yield, 94:6 er) from **4v'** (24.0 mg, 0.06 mmol) according to the same procedure for the preparation of **12**.

$[\alpha]_D^{23}$: +10.7 (*c* = 0.5, $CHCl_3$). HPLC analysis of the product: Daicel CHIRALPAK® IB N-3 column; 20% *i*-PrOH in *n*-hexane; 1.0 mL/min; retention times: 13.6 min (major),

16.4 min (minor).

^{1}H NMR (400 MHz, $CDCl_3$) δ 7.35 – 7.24 (m, 5H), 7.10 (d, *J* = 7.7 Hz, 2H), 6.98 (d, *J* = 7.8 Hz, 2H), 4.42 (s, 2H), 3.60 (s, 3H), 3.42 – 3.33 (m, 2H), 3.10 (d, *J* = 3.6 Hz, 1H), 3.08 – 3.02 (m, 1H), 2.89 (dd, *J* = 8.9, 1.8 Hz, 1H), 2.62 – 2.53 (m, 2H), 2.31 (s, 3H), 1.69 – 1.58 (m, 4H).

^{13}C NMR (101 MHz, $CDCl_3$) δ 200.1, 199.9, 138.2, 137.8, 137.4, 129.6, 128.4, 127.62, 127.60, 127.3, 83.1, 73.0, 69.6, 61.9, 58.1, 57.5, 47.73, 47.70, 38.3, 32.0, 27.3, 21.0.

HRMS (ESI) Calcd for $C_{26}H_{28}NaO_4$ $[M + Na]^+$: 427.1880, found: 427.1881.

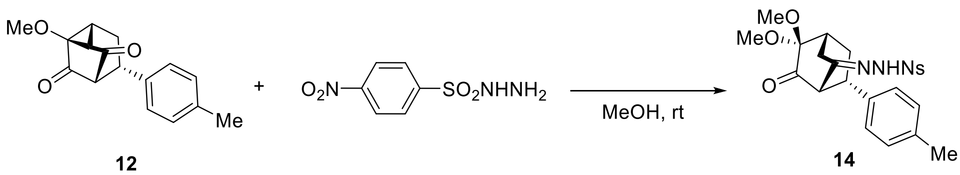

(1*S*,4*R*,7*S*,*E*)-3,3-Dimethoxy-6-(2-((4-nitrophenyl)sulfonyl)-1l4-diazaneylidene)-7-(*p*-tolyl)bicyclo[2.2.2]octan-2-one (14). A solution of **12** (25.6 mg, 0.1 mmol, 1.0 equiv) in MeOH (2.0 mL, 0.5 M) was treated with 4-nitrobenzenesulfonohydrazide (19.6 mg, 0.105 mmol, 1.05 equiv). The mixture was stirred overnight at room temperature and then directly purified by silica gel column chromatography (*n*-hexane/EtOAc = 5:1 to 5:3) to yield **14** as a white solid (35.0 mg, 80% yield, 98:2 er).

$[\alpha]_D^{23}$: –28.9 (*c* = 0.5, $CHCl_3$). HPLC analysis of the product: Daicel CHIRALPAK® IC-3 column; 30% *i*-PrOH in *n*-hexane; 1.0 mL/min; retention times: 16.4 min (major), 32.1 min (minor).

^{1}H NMR (400 MHz, $CDCl_3$) δ 8.37 (d, *J* = 8.9 Hz, 2H), 8.14 (d, *J* = 8.8 Hz, 2H), 7.82 (s, 1H), 7.09 (d, *J* = 7.9 Hz, 2H), 7.02 (d, *J* = 8.2 Hz, 2H), 3.37 (s, 4H), 3.19 (s, 3H), 3.17 (d, *J* = 2.1 Hz, 1H), 2.75 – 2.62 (m, 2H), 2.36 (dd, *J* = 17.2, 2.1 Hz, 1H), 2.30 (s, 3H), 2.20 – 2.13 (m, 2H).

^{13}C NMR (101 MHz, $CDCl_3$) δ 200.3, 156.9, 150.5, 143.5, 138.2, 137.0, 129.5, 129.4, 127.3, 124.2, 97.6, 60.7, 50.3, 49.6, 42.3, 33.5, 27.6, 27.4, 21.0.

HRMS (ESI) Calcd for $C_{23}H_{26}NaN_3O_7S$ $[M – H]^-$: 486.1340, found: 486.1339.

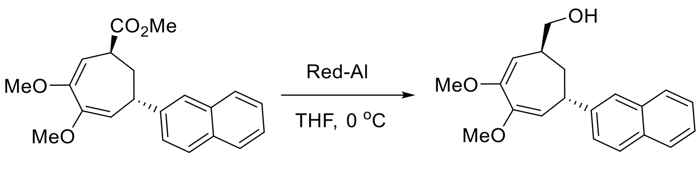

5h, >20:1 dr, 95:5 e.r. **15**

((1*S*,6*S*)-3,4-Dimethoxy-6-(naphthalen-2-yl)cyclohepta-2,4-dien-1-yl)methanol (15). At 0 °C, a solution of **5h** (55.0 mg, 0.16 mmol, 1.0 equiv) in THF (10 mL) was added Red-Al (3.5 M, 90 μL, 0.32 mmol, 2.0 equiv). The mixture was stirred for 20 minutes at 0 °C before it was quenched with a saturated aqueous NH_4Cl solution (5 mL). The organic layer was separated, and the aqueous layer was extracted with EtOAc (3 x 5 mL). The combined organic layers were dried over $MgSO_4$, filtered, and concentrated under reduced pressure. The residue was purified by silica gel column chromatography (*n*-hexane/EtOAc = 5:1 to 5:3) to afford **15** as a colorless oil (42.0 mg, 85% yield, 97:3 er).

$[\alpha]_D^{23}$: +92.1 (*c* = 1.0, $CHCl_3$). HPLC analysis of the product: Daicel CHIRALPAK® IB N-3 column; 20% *i*-PrOH in *n*-hexane; 1.0 mL/min; retention times: 10.0 min (minor), 11.9 min (major).

^{1}H NMR (400 MHz, $CDCl_3$) δ 7.82 – 7.76 (m, 3H), 7.70 (s, 1H), 7.47 – 7.39 (m, 3H), 5.31 (d, *J* = 5.9 Hz, 1H), 5.18 (d, *J* = 5.5 Hz, 1H), 3.72 – 3.65 (d, *J* = 9.9 Hz, 5H), 3.60 (s, 3H), 3.56 – 3.49 (m, 1H), 2.50 (dq, *J* = 12.4, 5.6 Hz, 1H), 2.30 (td, *J* = 12.6, 7.0 Hz, 1H), 2.06 (td, *J* = 12.4, 6.9 Hz, 1H), 1.73 (br, 1H).

^{13}C NMR (101 MHz, $CDCl_3$) δ 151.7, 151.1, 142.7, 133.5, 132.1, 128.1, 127.5 (2C), 126.5, 126.0, 125.7, 125.3, 107.3, 103.9, 66.0, 55.0, 54.9, 47.3, 42.0, 38.5.

HRMS (ESI) Calcd for $C_{20}H_{22}NaO_3$ $[M + Na]^+$: 333.1461, found: 333.1466.

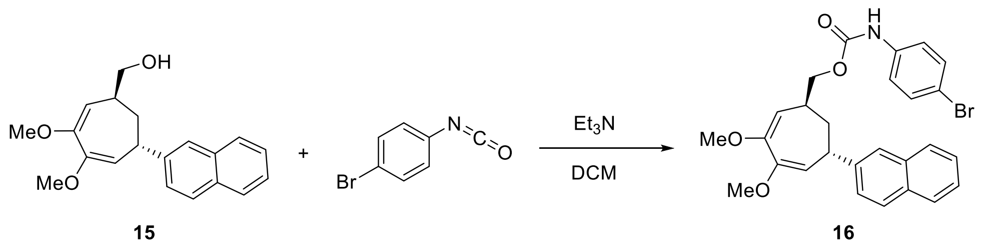

((1*S*,6*S*)-3,4-Dimethoxy-6-(naphthalen-2-yl)cyclohepta-2,4-dien-1-yl)methyl (4-bromophenyl)carbamate (16). A solution of **15** (19.0 mg, 0.06 mmol, 1.0 equiv) and 1-bromo-4-isocyanatobenzene (18.0 mg, 0.09 mmol, 1.5 equiv) in CH_2Cl_2 (2 mL) was treated with Et_3N (15 μL, 0.09 mmol, 1.5 equiv). The mixture was stirred overnight at room temperature, then concentrated under reduced pressure and purified by silica gel column chromatography (*n*-hexane/EtOAc = 10:1 to 5:1) to yield **16** as a white solid (26 mg, 82% yield, 97:3 er).

$[\alpha]_D^{23}$: +58.7 (*c* = 1.0, $CHCl_3$). HPLC analysis of the product: Daicel CHIRALPAK® IA-3 column; 10% *i*-PrOH in *n*-hexane; 1.0 mL/min; retention times: 25.7 min (major), 28.3 min (minor).

^{1}H NMR (400 MHz, $CDCl_3$) δ 7.85 – 7.77 (m, 3H), 7.71 (s, 1H), 7.50 – 7.43 (m, 3H), 7.43 – 7.37 (m, 2H), 7.24 – 7.20 (m, 1H), 6.69 (br, 1H), 6.58 – 6.50 (m, 1H), 5.33 (d, *J* = 5.9 Hz, 1H), 5.14 (d, *J* = 5.5 Hz, 1H), 4.23 (d, *J* = 6.3 Hz, 2H), 3.70 (s, 3H), 3.62 (s, 3H), 3.58 – 3.50 (m, 1H), 2.70 (td, *J* = 12.3, 6.2 Hz, 1H), 2.34 (td, *J* = 12.6, 7.0 Hz, 1H), 2.11 (td, *J* = 12.5, 7.0 Hz, 1H).

^{13}C NMR (101 MHz, $CDCl_3$) δ 153.3, 152.0, 151.0, 142.4, 136.9, 133.5, 132.2, 132.0, 128.3, 127.6, 127.5, 126.4, 126.1, 125.8, 125.5, 120.1, 116.7, 107.2, 103.3, 68.1, 55.1, 55.0, 47.5, 42.0, 35.6.

HRMS (ESI) Calcd for $C_{27}H_{26}NaBrNO_4$ $[M + Na]^+$: 530.0943, found: 530.0944.

VII. Mechanistic Studies

Note: At –78 °C, **1a** was slightly soluble in ^{t}BuOMe, and the resulting oxonium salts was also slightly soluble in ^{t}BuOMe. Under these reaction conditions, the dissolution of **1a** reached saturation, and the concentration remained unchanged at low conversion. In this case, we measured the reaction order of the other components.

(1) Comparison between 1a and 1b.

a) Enantioselectivities of 4bb at different loadings of 3i.

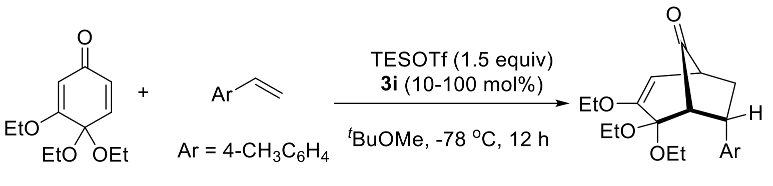

entry	**3i**	yield (**4bb**)	er
1	10 mol%	90%	58:42
2	50 mol%	87%	90:10
3	100 mol%	60%	93:7

An oven-dried 5-mL vial was charged with **1b** (11.3 mg, 0.05 mmol, 1.0 equiv), **2b** (8.9 mg, 0.075 mmol, 1.5 equiv), catalyst **3i** (10 to 100 mol%), and dry ^{t}BuOMe (0.1 M, 0.5 mL). The vial was sealed and cooled to –78 °C in a low-temperature reactor. TESOTf (17.1 μL, 19.8 mg, 0.075 mmol, 1.5 equiv) was added via syringe. The reaction was stirred for 12 hours and then quenched by the addition of EtOH/Et_3N (0.2 mL, v/v = 3:1) via syringe. The mixture was stirred at –78 °C for 5 minutes before warming to room temperature. The crude reaction mixture was concentrated, and CH_2Br_2 (7.0 μL, 17.4 mg, 0.1 mmol) was added. The yield was determined by ^{1}H NMR of the crude mixture. The mixture was then concentrated and purified by silica gel flash chromatography (*n*-hexane/EtOAc = 7:1 to 5:2). The enantiomeric excess was determined by chiral HPLC following chromatographic purification on silica gel.

(1*S*,6*S*)-3,4,4-Triethoxy-6-(*p*-tolyl)bicyclo[3.2.1]oct-2-en-8-one (**4bb**)

$[\alpha]_D^{23}$: –97.7 (*c* = 0.5, $CHCl_3$) (93:7 er). HPLC analysis of the product: Daicel CHIRALPAK® IN-3 column; 10% *i*-PrOH in *n*-hexane; 1.0 mL/min; retention times: 8.0 min (major), 10.0 min (minor).

^{1}H NMR (400 MHz, $CDCl_3$) δ 7.18 (d, *J* = 8.1 Hz, 2H), 7.07 (d, *J* = 7.8 Hz, 2H), 5.09 (d, *J* = 7.5 Hz, 1H), 3.98 – 3.86 (m, 1H), 3.84 – 3.73 (m, 1H), 3.60 – 3.42 (m, 3H), 3.20 (dq, *J* = 8.9, 7.1 Hz, 1H), 3.07 (dd, *J* = 7.7, 1.6 Hz, 1H), 2.80 (ddd, *J* = 7.4, 5.7, 1.6 Hz, 1H), 2.70 (dq, *J* = 8.9, 7.0 Hz, 1H), 2.40 (ddd, *J* = 13.0, 11.4, 5.9 Hz, 1H), 2.31 (s, 3H), 2.11 (dd, *J* = 12.9, 5.5 Hz, 1H), 1.36 (t, *J* = 7.0 Hz, 3H), 1.06 (t, *J* = 7.1 Hz, 3H), 0.45 (t, *J* = 7.0 Hz, 3H).

^{13}C NMR (101 MHz, $CDCl_3$) δ 208.3, 154.7, 138.4, 135.9, 129.9, 128.3, 102.8, 100.2, 63.4, 58.0, 56.3, 55.6, 44.0, 37.9, 35.2, 20.9, 15.4, 14.4, 14.0.

HRMS (ESI) Calcd for $C_{21}H_{28}NaO_4$ $[M + Na]^+$: 367.1880, found: 367.1887.

b) Monitoring the yield of 4b and 4bb.

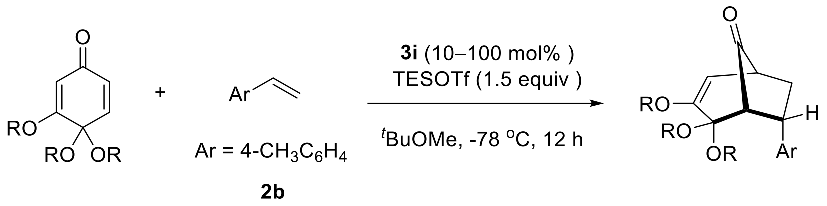

1a (R = Me) *slightly soluble* **4b**

1b (R = Et) *complete dissolution* **4bb**

An oven-dried 5-mL vial was charged with **1** (0.05 mmol), **2b** (8.9 mg, 0.075 mmol, 1.5 equiv), catalyst **3i** (0 or 10 mol%), and dry ^{t}BuOMe (0.1 M, 0.5 mL). The vial was sealed, and the reaction mixture was cooled to –78 °C in a low-temperature reactor. TESOTf (17.1 μL, 19.8 mg, 0.075 mmol, 1.5 equiv) was added via syringe. The reaction was stirred for the designated length of time and then quenched by the addition of MeOH (**1a**)/Et_3N (0.2 mL, v/v = 3:1) or EtOH (**1b**)/Et_3N (0.2 mL, v/v = 3:1) via syringe. The mixture was allowed to stir at –78 °C for 5 minutes before warming to room temperature. The crude reaction mixture was concentrated, and CH_2Br_2 (7.0 μL, 17.4 mg, 0.1 mmol) was added. The yield dependence on time was measured by ^{1}H NMR and is listed in the following table.

yield / time	**1a** w/o **3i**	**1a**/10 mol% **3i**	**1b** w/o **3i**	**1b**/10 mol% **3i**
0.5 h	0	2.50%	6.50%	18.1%
1.0 h	0	5.40%	11.65%	22.88%
1.5 h	0	6.80%	20.66%	34.25%
2.0 h	0.1%	10.0%	28.33%	37.88%
2.5 h	0.2%	13.0%	31.75%	46.95%

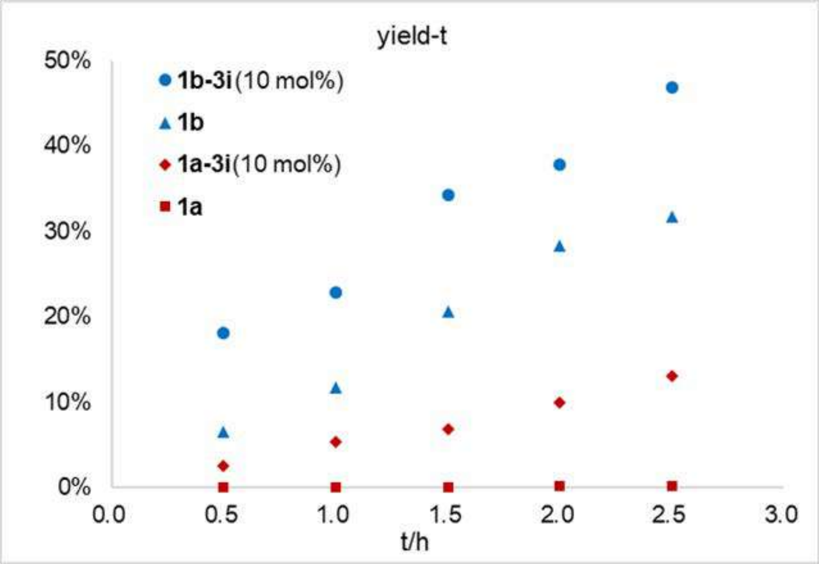

(2) Reaction order of the catalyst

(a) Initial rate analysis

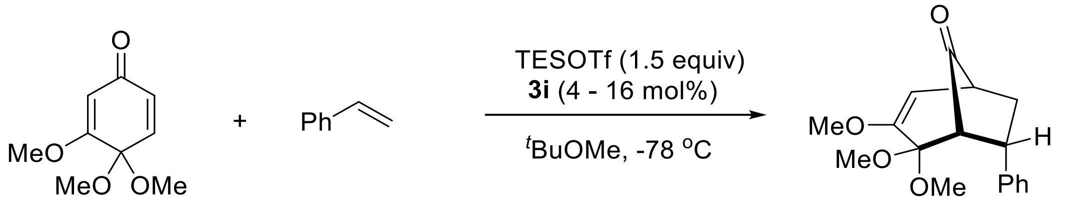

1a **2a** **4a**

An oven-dried 5-mL vial was charged with **1a** (9.2 mg, 0.05 mmol, 1.0 equiv), **2a** (7.8 mg, 0.075 mmol, 1.5 equiv), catalyst **3i** (4-16 mol%), and dry ^{t}BuOMe (0.1 M, 0.5 mL). The vial was sealed, and the reaction mixture was cooled to –78 °C in a low-

temperature reactor. TESOTf (17.1 μL, 19.8 mg, 0.075 mmol, 1.5 equiv) was added via syringe. The reaction was stirred for the designated length of time and then quenched by the addition of MeOH/Et_3N (0.2 mL, v/v = 3:1) via syringe. The mixture was stirred at –78 °C for 5 minutes before warming to room temperature. The crude reaction mixture was concentrated, and CH_2Br_2 (7.0 μL, 17.4 mg, 0.1 mmol) was added. The yield dependence on catalyst loading and time was measured by ^{1}H NMR and is listed in the following table.

yield / catalyst loading / time	4% (0.004 M)	8% (0.008 M)	12% (0.012 M)	16% (0.016 M)
0.5 h	1.50%	2.80%	4.20%	5.20%
1.0 h	2.40%	4.70%	6.40%	8.30%
1.5 h	3.90%	7.00%	9.00%	11.50%
2.0 h	5.60%	10.50%	14.00%	14.60%
2.5 h	6.10%	12.00%	15.60%	19.60%

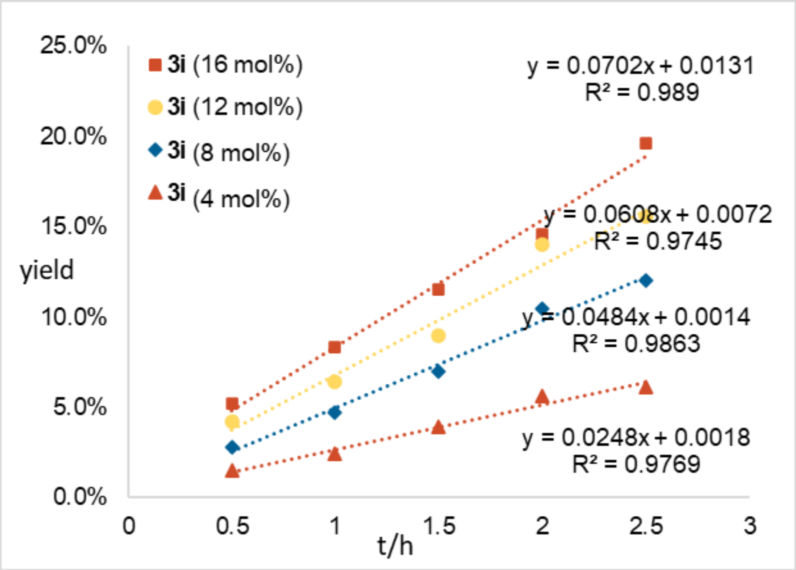

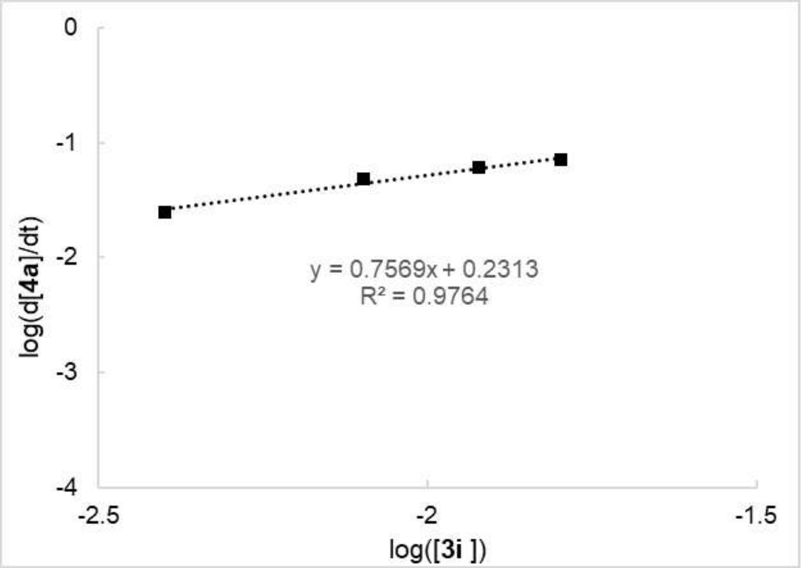

(b) Visual kinetic analysis

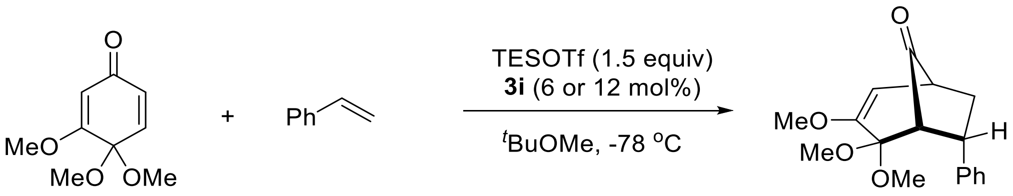

An oven-dried 5-mL vial was charged with **1a** (9.2 mg, 0.05 mmol, 1.0 equiv), **2a** (7.8 mg, 0.075 mmol, 1.5 equiv), catalyst **3i** (6 or 12 mol%), and dry ^{t}BuOMe (0.1 M, 0.5 mL). The vial was sealed, and the reaction mixture was cooled to –78 °C in a low-temperature reactor. TESOTf (17.1 μL, 19.8 mg, 0.075 mmol, 1.5 equiv) was added via syringe. The reaction was stirred for the designated length of time and then quenched by the addition of MeOH/Et_3N (0.2 mL, v/v = 3:1) via syringe. The mixture was stirred at –78 °C for 5 minutes before warming to room temperature. The crude reaction mixture was concentrated, and CH_2Br_2 (7.0 μL, 17.4 mg, 0.1 mmol) was added. The yield dependence on catalyst loading and time was measured by ^{1}H NMR and is listed in the following table.

The reaction order of the catalyst was determined with Variable Time Normalisation Analysis (VTNA) established by the Burés group.[13] Concentrarion plots of product formation with time scales normalized to different reaction orders of the catalyst (0th,

0.5th, 0.75th, 1.0th). The best overlap was found when the reaction profiles are normalized to a 0.75 order dependence in catalyst concentration.

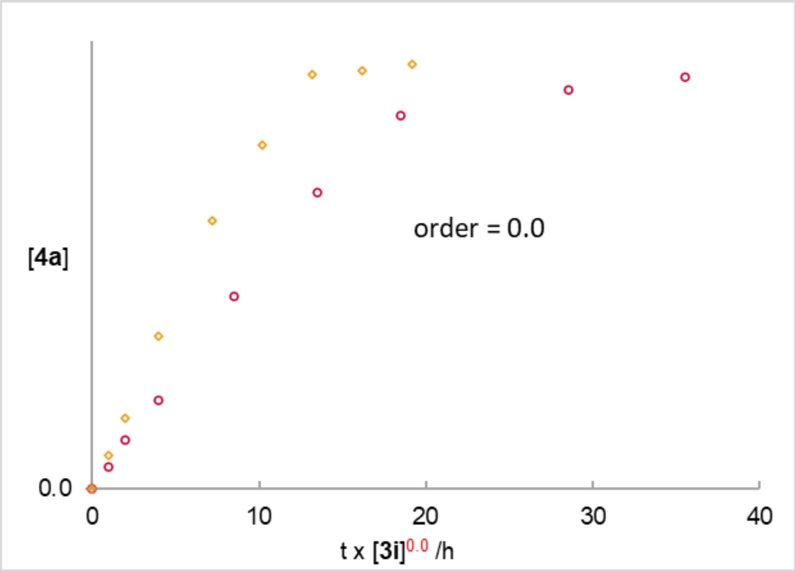

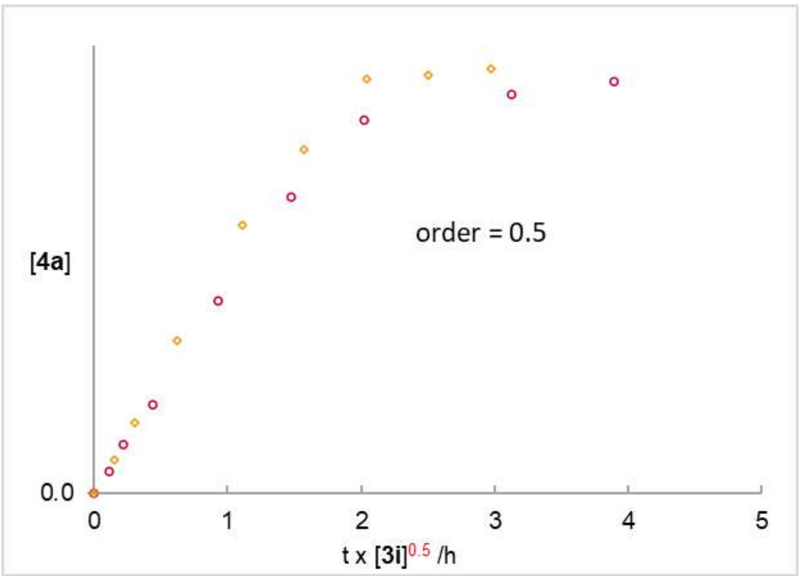

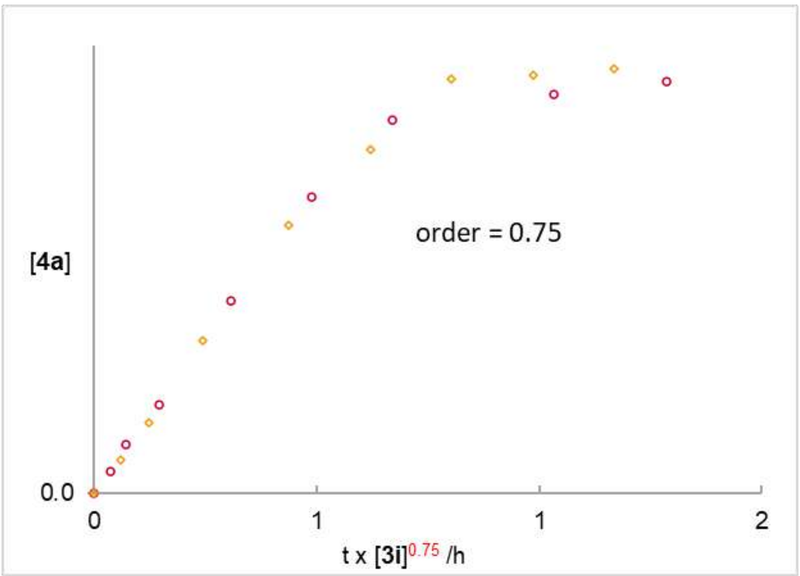

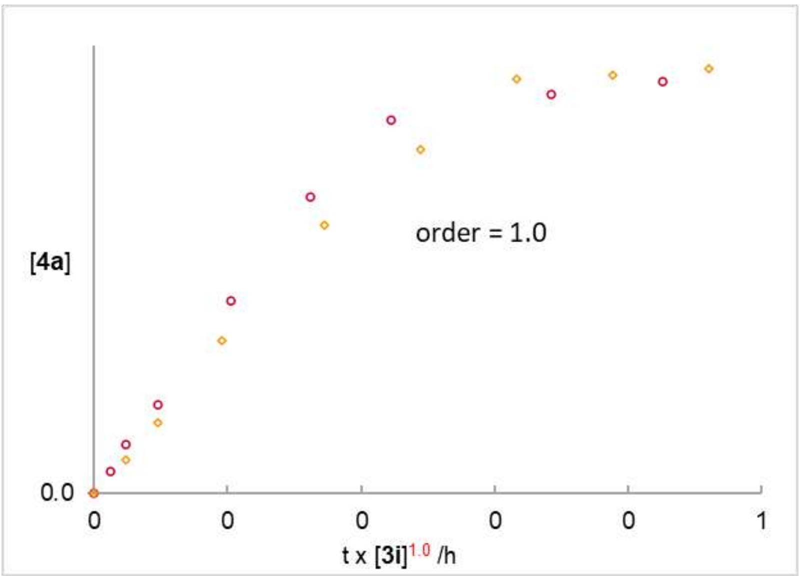

(3) Reaction order of 2a

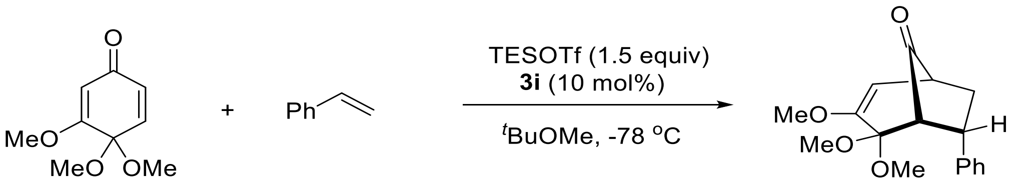

1a **2a** **4a**

An oven-dried 5-mL vial was charged with **1a** (9.2 mg, 0.05 mmol, 1.0 equiv), **2a** (0.1-0.4 M), catalyst **3i** (3.53 mg, 10 mol%), and dry ^{t}BuOMe (0.1 M, 0.5 mL). The vial was sealed, and the reaction mixture was cooled to –78 ^{o}C in a low-temperature reactor. TESOTf (17.1 μL, 19.8 mg, 0.075 mmol, 1.5 equiv) was added via syringe. The reaction

was stirred for the designated length of time and then quenched by the addition of MeOH/Et_3N (0.2 mL, v/v = 3:1) via syringe. The mixture was stirred at –78 °C for 5 minutes before warming to room temperature. The crude reaction mixture was concentrated, and CH_2Br_2 (7.0 μL, 17.4 mg, 0.1 mmol) was added. The yield dependence on **2a** and time was measured by ^1H NMR and is listed in the following table, indicating that the reaction is zero order in **2a**.

yield / [**2a**] / time	0.1 M	0.2 M	0.3 M	0.4 M
1.0 h	6.10%	5.40%	5.50%	5.20%
1.5 h	7.50%	7.20%	7.20%	7.70%
2.0 h	10.10%	9.50%	11.00%	10.70%
2.5 h	13.00%	14.80%	15.00%	14.20%
3.0 h	18.60%	18.00%	18.80%	18.30%

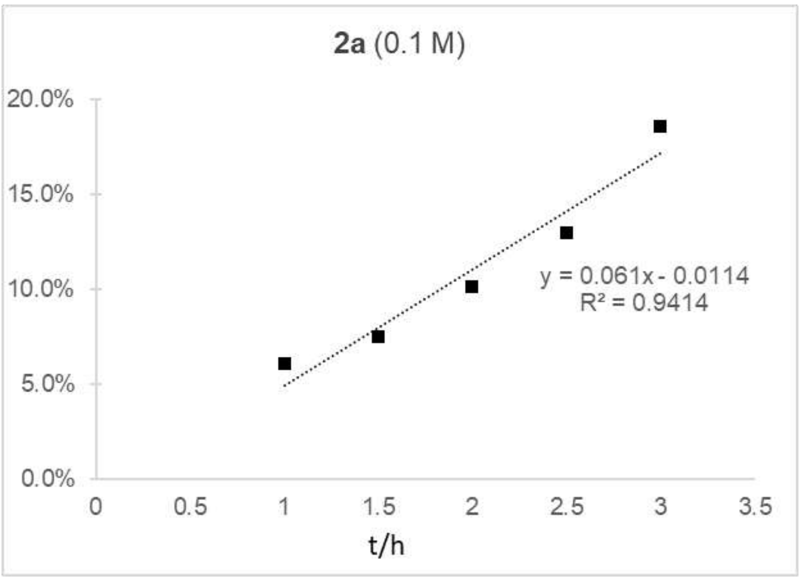

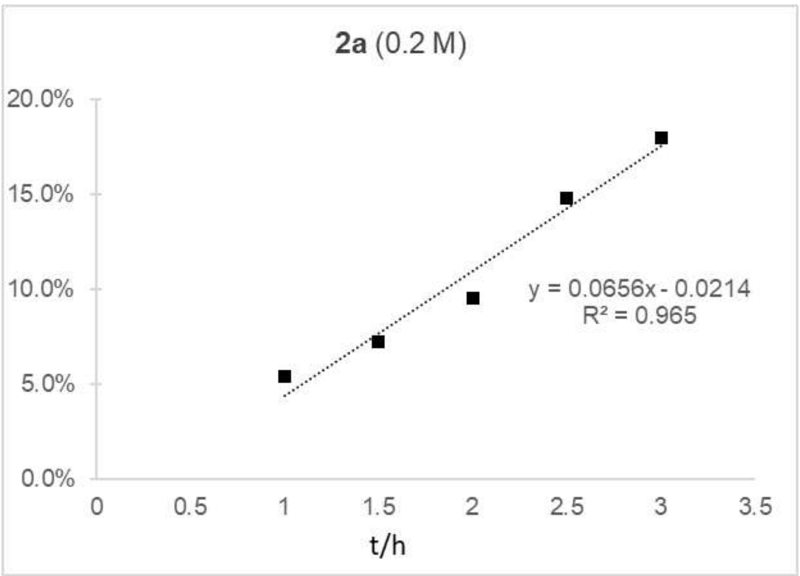
2a (0.2 M)
20.0%
15.0%
10.0%
5.0%
0.0%
0
0.5
1
1.5
2
2.5
3
3.5
t/h
y = 0.0656x - 0.0214
R² = 0.965

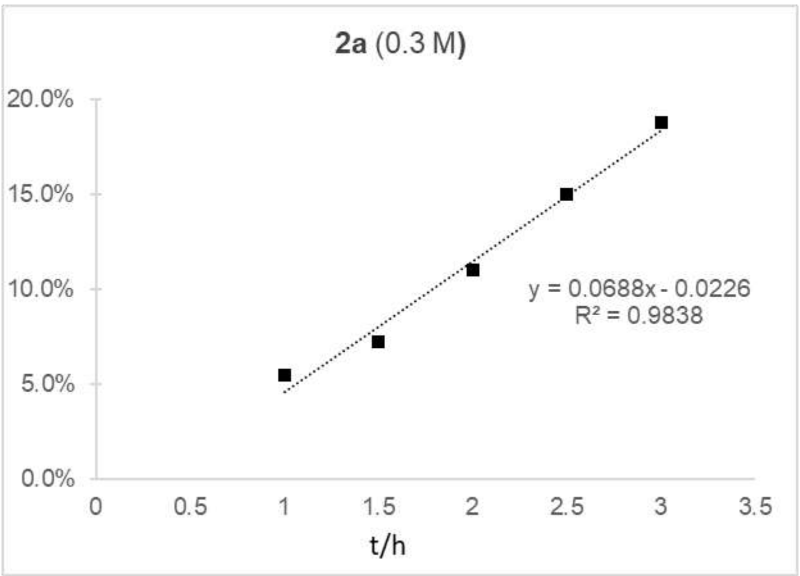
2a (0.3 M)
20.0%
15.0%
10.0%
5.0%
0.0%
0
0.5
1
1.5
2
2.5
3
3.5
t/h
y = 0.0688x - 0.0226
R² = 0.9838

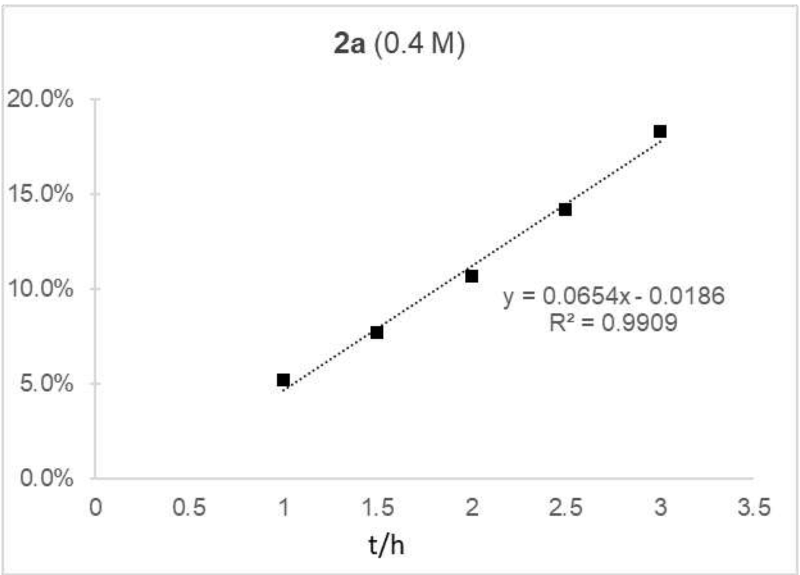

2a (0.4 M)
20.0%
15.0%
10.0%
5.0%
0.0%
0
0.5
1
1.5
2
2.5
3
3.5
t/h
y = 0.0654x - 0.0186
R² = 0.9909

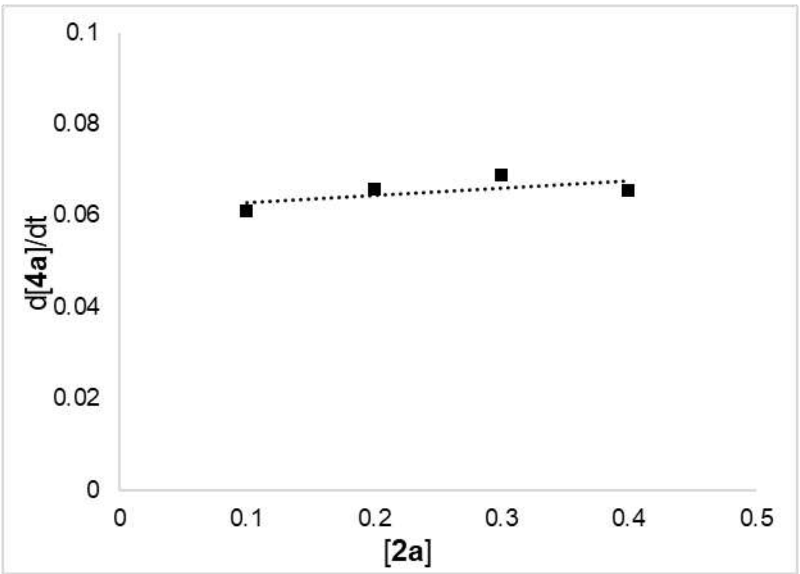

0.1
0.08
0.06
0.04
0.02
0
d[4a]/dt
0
0.1
0.2
0.3
0.4
0.5
[2a]

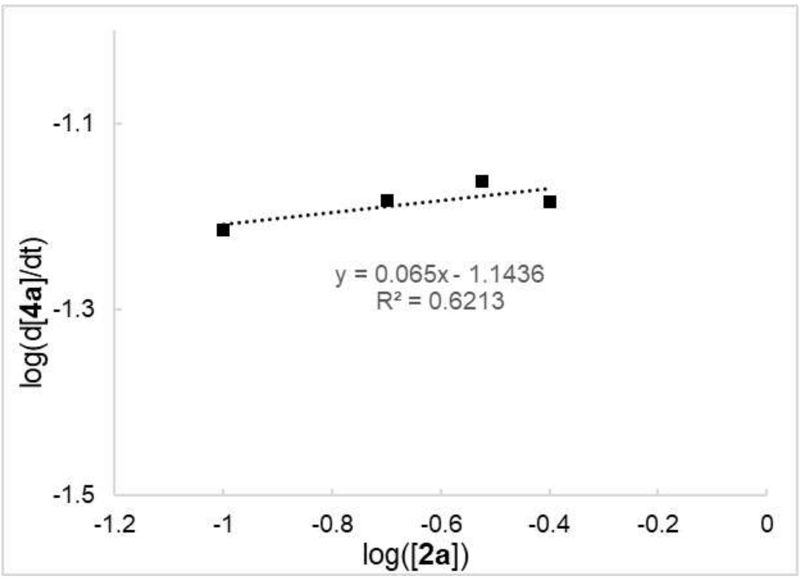

-1.1
-1.3
-1.5
log(d[4a]/dt)
-1.2
-1
-0.8
-0.6
-0.4
-0.2
0
log([2a])
y = 0.065x - 1.1436
R² = 0.6213

(4) Reaction order of TESOTf

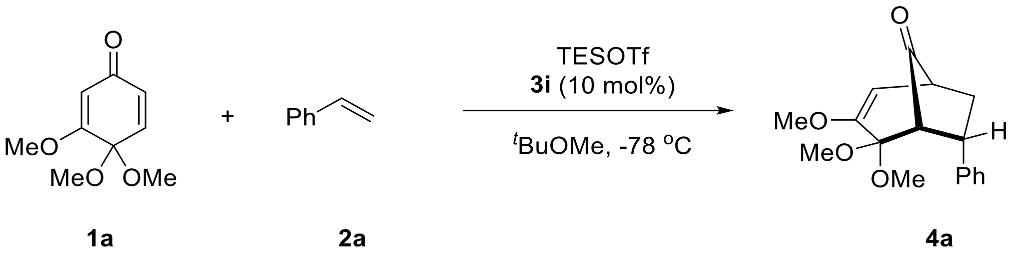

An oven-dried 5-mL vial was charged with **1a** (9.2 mg, 0.05 mmol, 1.0 equiv), **2a** (7.8 mg, 0.075 mmol, 1.5 equiv), catalyst **3i** (3.53 mg, 10 mol%), and dry ^{t}BuOMe (0.1 M, 0.5 mL). The vial was sealed, and the reaction mixture was cooled to –78 °C in a low-temperature reactor. TESOTf (0.1-0.5 M) was added via syringe. The reaction was stirred for the designated length of time and then quenched by the addition of MeOH/Et_3N (0.2 mL, v/v = 3:1) via syringe. The mixture was stirred at –78 °C for 5 minutes before warming to room temperature. The crude reaction mixture was concentrated, and CH_2Br_2 (7.0 μL, 17.4 mg, 0.1 mmol) was added. The yield dependence on TESOTf and time was measured by ^{1}H NMR and is listed in the following table, indicating that the reaction is zero order in TESOTf.

yield / [TESOTf] / time	0.1 M	0.2 M	0.3 M	0.4 M	0.5 M
0.5 h	2.70%	2.84%	2.63%	2.62%	2.61%
1.0 h	3.65%	4.44%	4.19%	4.10%	3.77%
1.5 h	5.26%	5.77%	5.64%	5.14%	4.51%
2.0 h	7.13%	7.13%	7.04%	7.50%	7.99%
2.5 h	7.87%	9.30%	9.99%	9.02%	8.52%

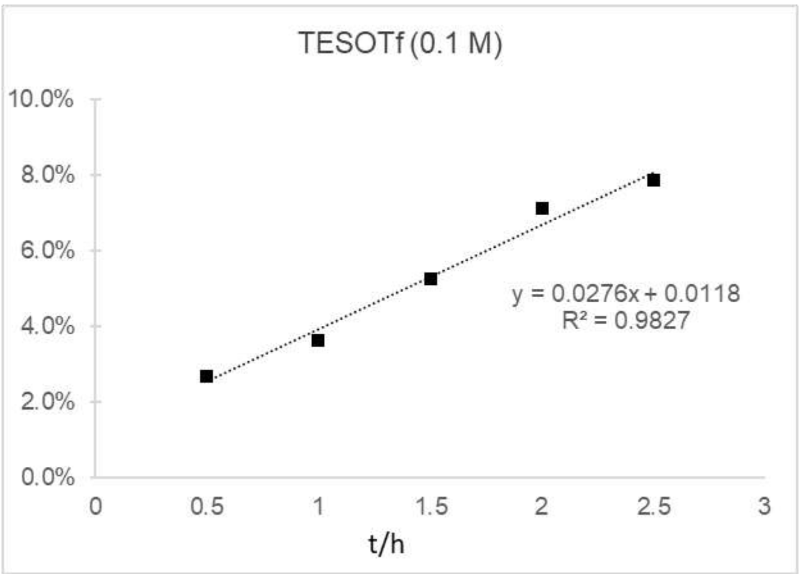
TESOTf (0.1 M)
10.0%
8.0%
6.0%
4.0%
2.0%
0.0%
0
0.5
1
1.5
2
2.5
3
t/h
y = 0.0276x + 0.0118
R² = 0.9827

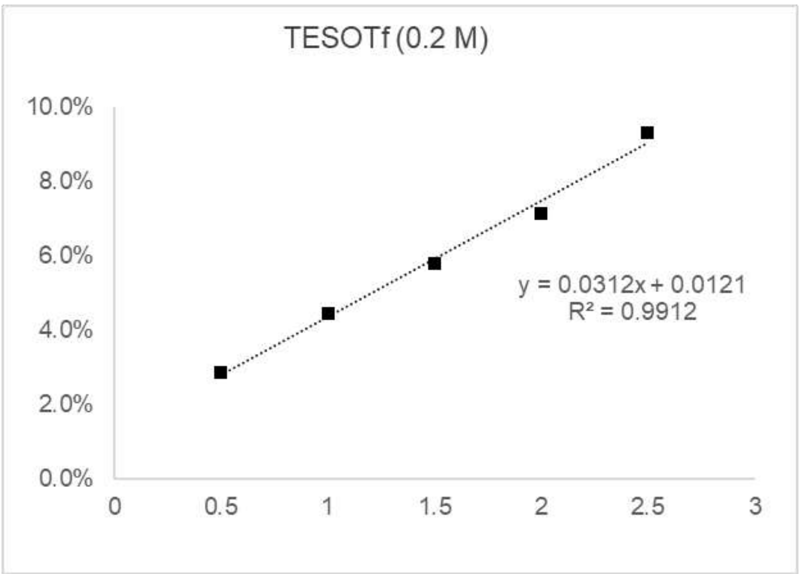
TESOTf (0.2 M)
10.0%
8.0%
6.0%
4.0%
2.0%
0.0%
0
0.5
1
1.5
2
2.5
3
y = 0.0312x + 0.0121
R² = 0.9912

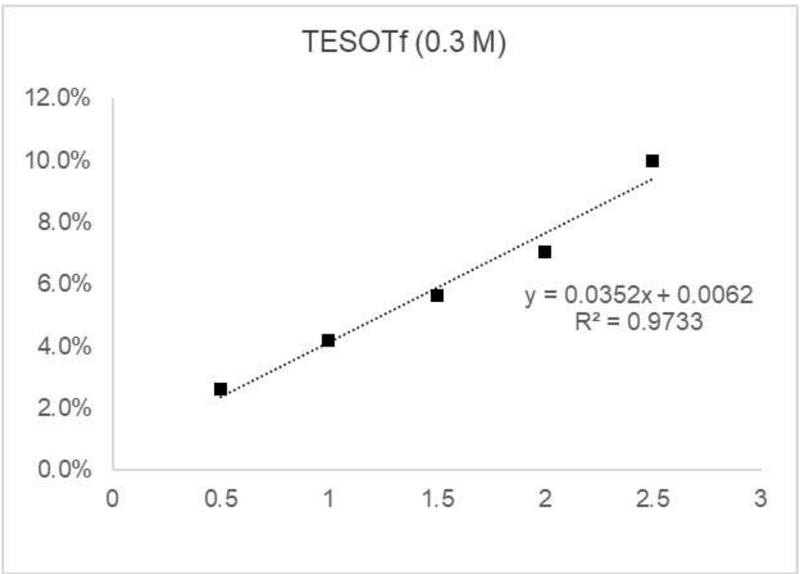
TESOTf (0.3 M)
12.0%
10.0%
8.0%
6.0%
4.0%
2.0%
0.0%
0
0.5
1
1.5
2
2.5
3
y = 0.0352x + 0.0062
R² = 0.9733

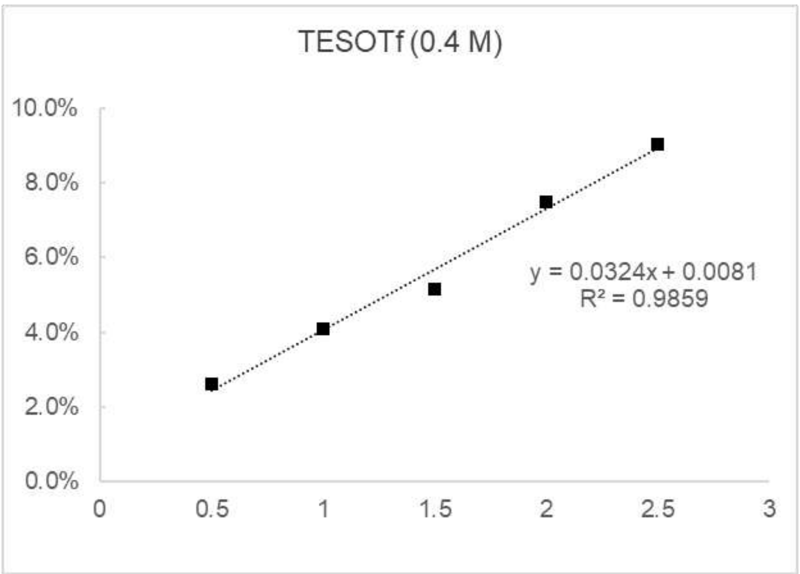
TESOTf (0.4 M)
10.0%
8.0%
6.0%
4.0%
2.0%
0.0%
0
0.5
1
1.5
2
2.5
3
y = 0.0324x + 0.0081
R² = 0.9859

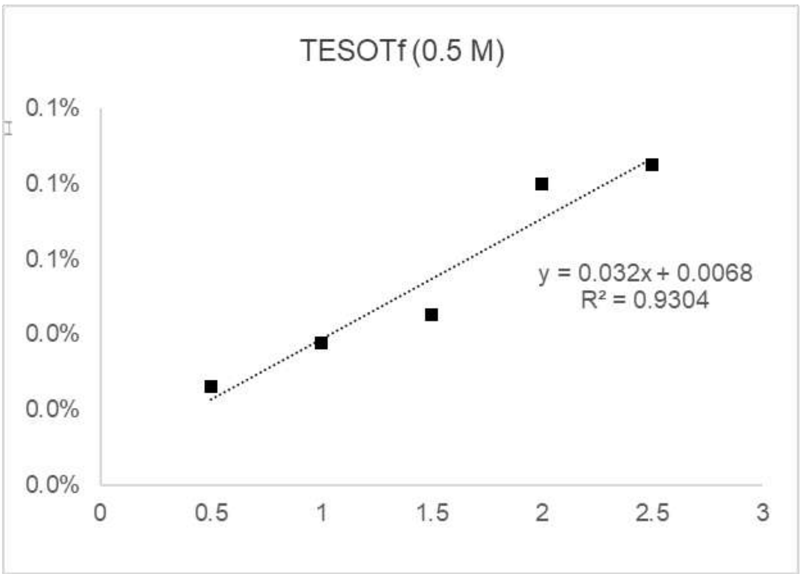
TESOTf (0.5 M)
0.1%
0.1%
0.1%
0.0%
0.0%
0.0%
0
0.5
1
1.5
2
2.5
3
y = 0.032x + 0.0068
R² = 0.9304

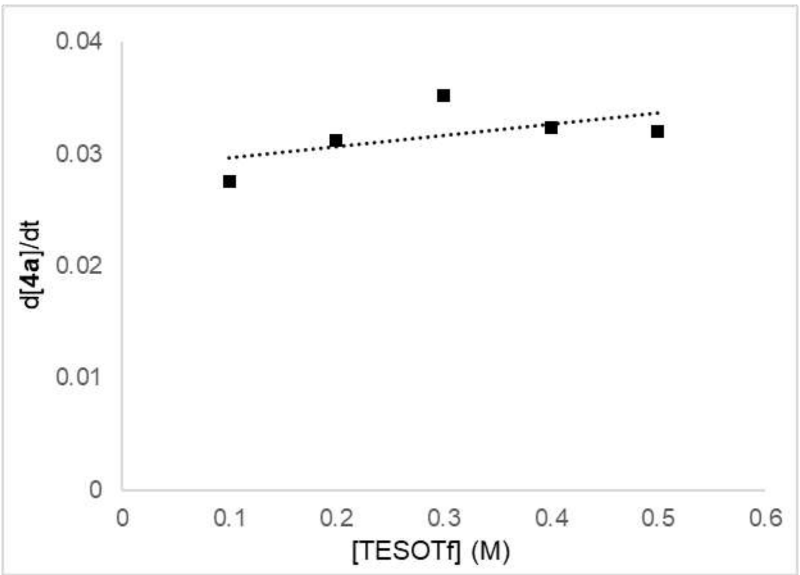
0.04
0.03
0.02
0.01
0
d[4a]/dt
0
0.1
0.2
0.3
0.4
0.5
0.6
[TESOTf] (M)

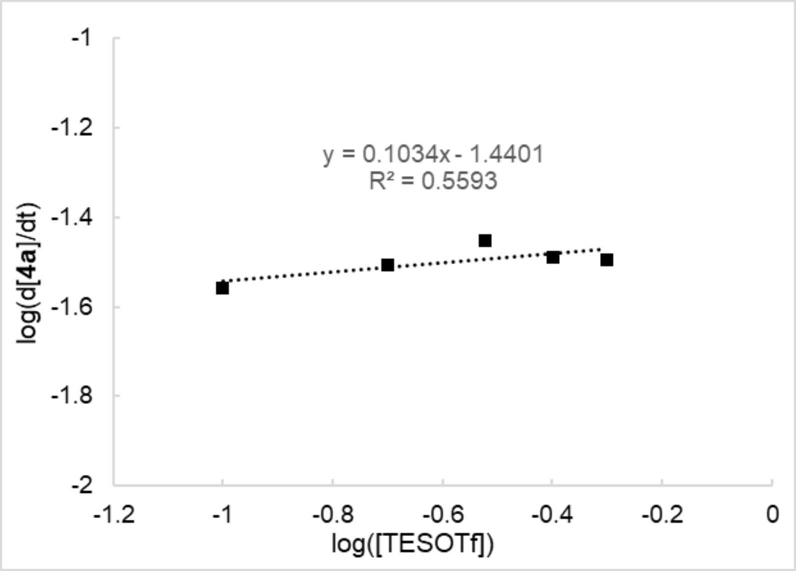

(5) Hammett plot

(a) Parallel experiment

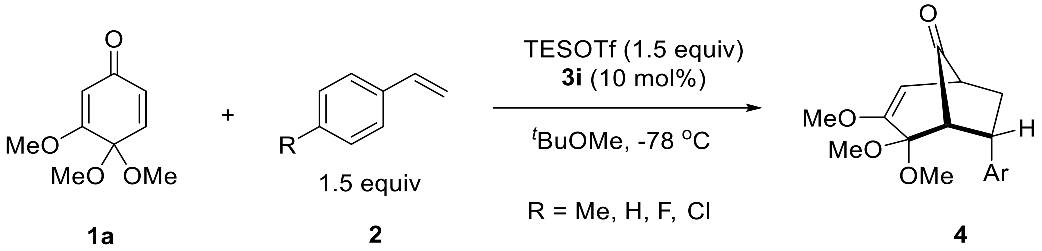

An oven-dried 5-mL vial was charged with **1a** (9.2 mg, 0.05 mmol, 1.0 equiv), **2** (0.075 mmol, 1.5 equiv), catalyst **3i** (3.53 mg, 10 mol%), and dry ^{t}BuOMe (0.1 M, 0.5 mL). The vial was sealed, and the reaction mixture was cooled to –78 °C in a low-temperature reactor. TESOTf (17.1 μL, 19.8 mg, 0.075 mmol, 1.5 equiv) was added via syringe. The reaction was stirred for the designated length of time and then quenched by the addition of MeOH/Et_3N (0.2 mL, v/v = 3:1) via syringe. The mixture was stirred at –78 °C for 5 minutes before warming to room temperature. The crude reaction mixture was concentrated, and CH_2Br_2 (7.0 μL, 17.4 mg, 0.1 mmol) was added. The yield dependence on substituent R and time was measured by ^{1}H NMR and is listed in the following table.

yield / R/ time	4-Me	H	4-F	4-Cl
σ^+	-0.31	0	-0.07	0.11
1.0 h	5.30%	5.12%	5.40%	5.20%
1.5 h	6.50%	6.60%	7.30%	6.90%
2.0 h	10.14%	10.20%	10.30%	9.40%
2.5 h	11.60%	12.70%	12.90%	11.00%
3.0 h	16.30%	17.40%	18.00%	15.20%

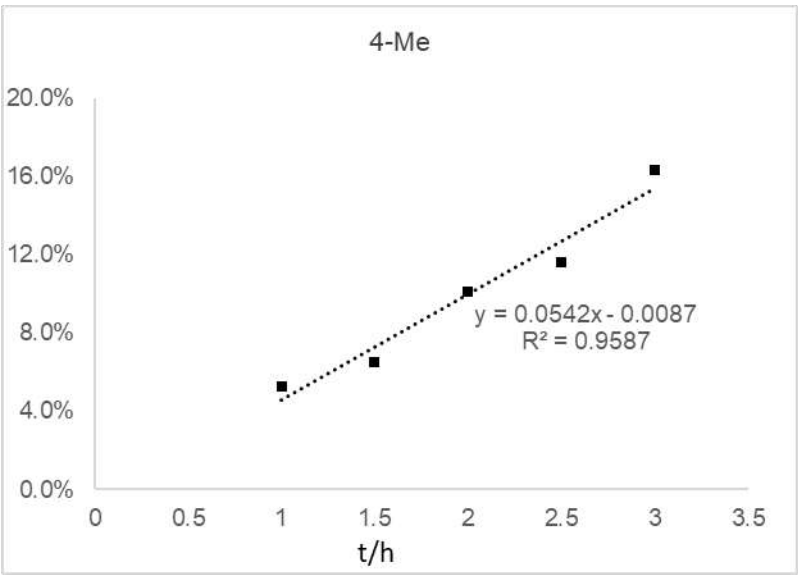

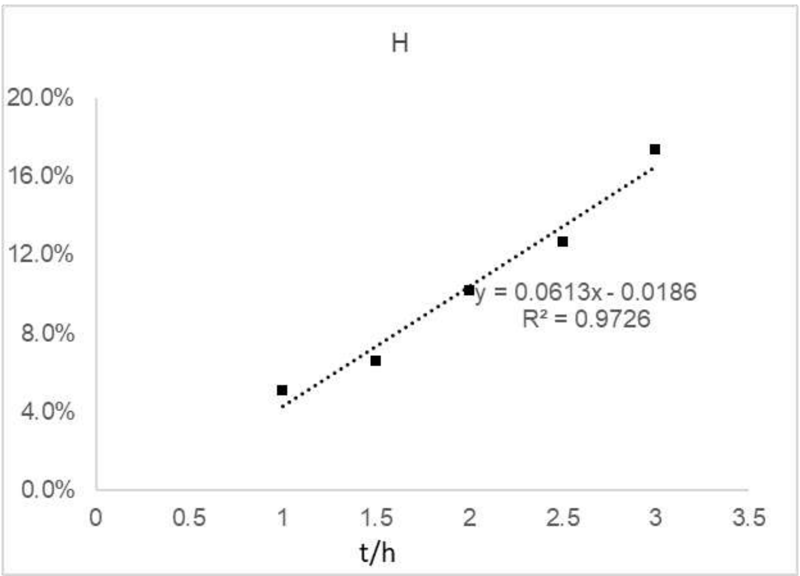

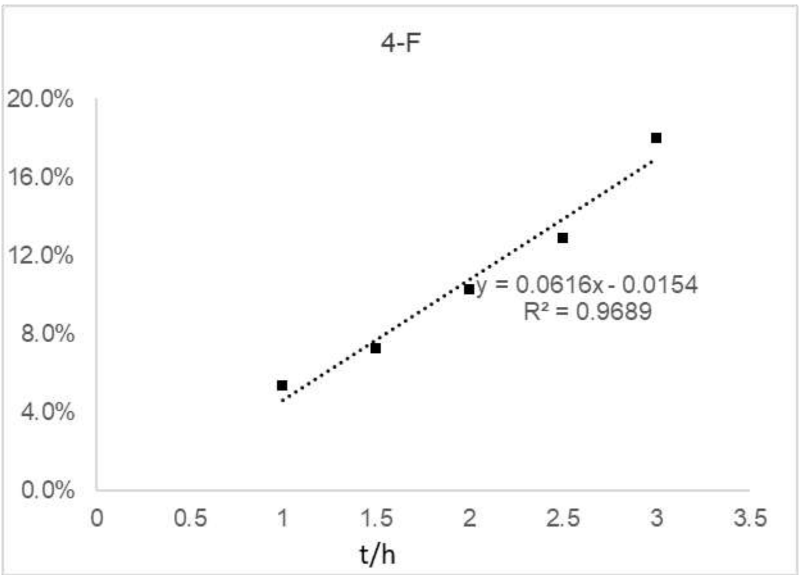
4-F
20.0%
16.0%
12.0%
8.0%
4.0%
0.0%
0
0.5
1
1.5
2
2.5
3
3.5
t/h
y = 0.0616x - 0.0154
R² = 0.9689

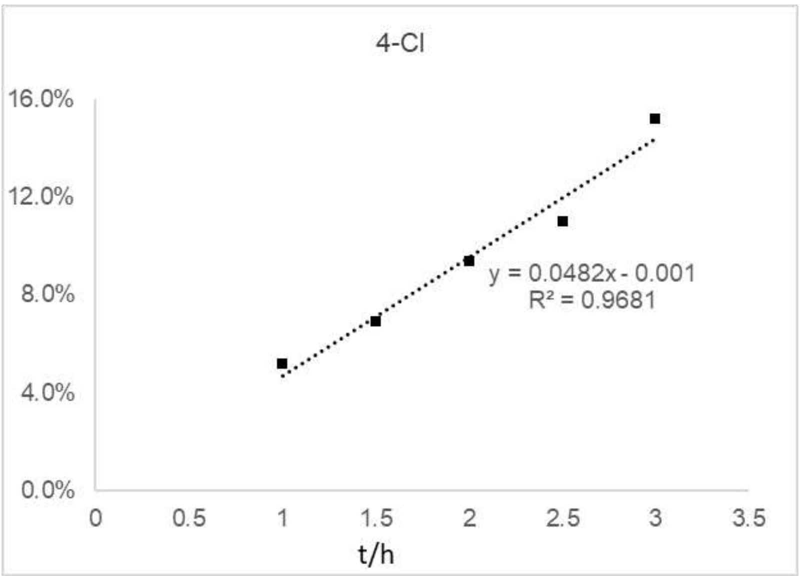
4-Cl
16.0%
12.0%
8.0%
4.0%
0.0%
0
0.5
1
1.5
2
2.5
3
3.5
t/h
y = 0.0482x - 0.001
R² = 0.9681

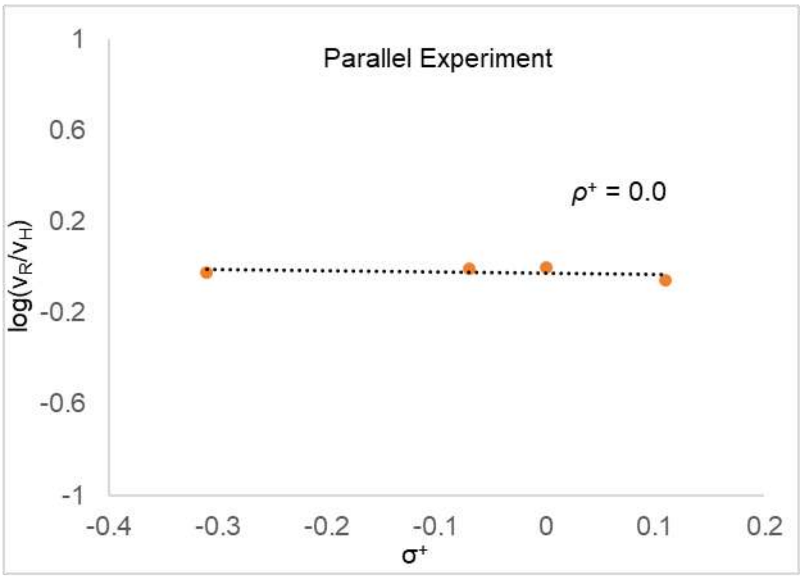
Parallel Experiment
1
0.6
0.2
-0.2
-0.6
-1
log(vR/vH)
ρ+ = 0.0
-0.4
-0.3
-0.2
-0.1
0
0.1
0.2
σ+

(b) Competition experiment

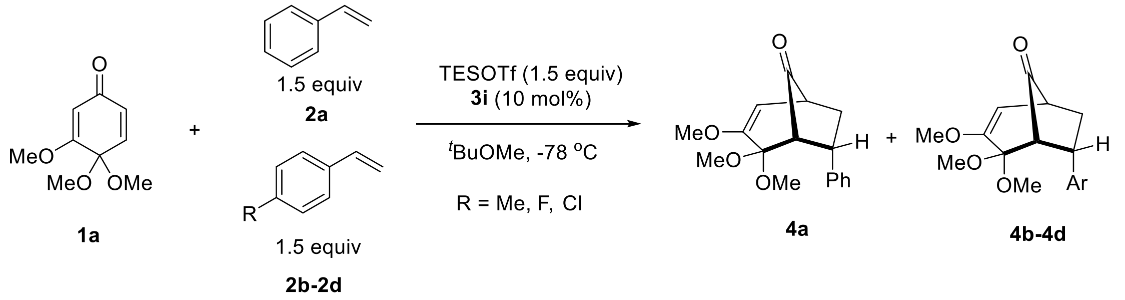

An oven-dried 5-mL vial was charged with **1a** (9.2 mg, 0.05 mmol, 1.0 equiv), **2a** (7.8 mg, 0.075 mmol, 1.5 equiv), **2b** to **2d** (0.075 mmol, 1.5 equiv), catalyst **3i** (3.53 mg, 10 mol%), and dry ^{t}BuOMe (0.1 M, 0.5 mL). The vial was sealed, and the reaction mixture was cooled to –78 °C in a low-temperature reactor. TESOTf (17.1 μL,19.8 mg, 0.075 mmol, 1.5 equiv) was added via syringe. The reaction was stirred for 2 hours and then quenched by the addition of MeOH/Et_3N (0.2 mL, v/v = 3:1) via syringe. The mixture was allowed to stir at –78 °C for 5 minutes before warming to room temperature. The crude reaction mixture was concentrated and CH_2Br_2 (7.0 μL, 0.1 mmol) was added. The conversion was below 20%. The yield ratio (**4**(**b**, **c** or **d**)/**4a**) was measured by ^{1}H NMR and is listed in the following table.

R	4-Me	H	4-F	4-Cl
σ^+	-0.31	0	-0.07	0.11
k_R/k_H	7.7	1	0.73	0.15
$\log(k_R/k_H)$	0.886	0	-0.137	-0.824

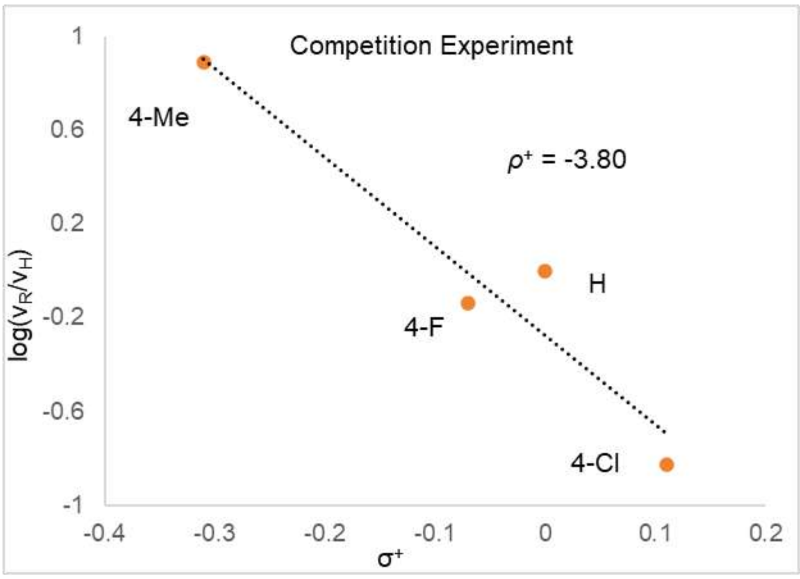

(c) Hammett plot (log(er)-σ^+)

R	4-Me	H	4-F	4-Cl
σ^+	-0.31	0	-0.07	0.11
er	97:3	95:5	94:6	88:12
log(er)	1.51	1.28	1.19	0.87

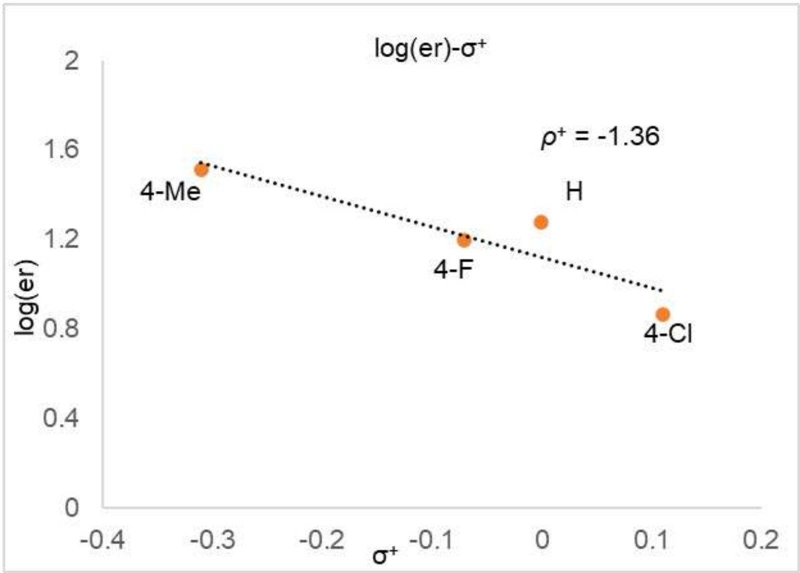

(6) Secondary kinetic isotope effect studies

An oven-dried 5-mL vial was charged with **1a** (9.2 mg, 0.05 mmol, 1.0 equiv), **2b**-d_2 or **2b**-d_1 (0.25 mmol, 5.0 equiv), **2b** (0.25 mmol, 5.0 equiv), catalyst **3i** (3.53 mg, 10 mol%), and dry ^{t}BuOMe (0.1 M, 0.5 mL). The vial was sealed, and the reaction mixture was cooled to –78 °C in a low-temperature reactor. TESOTf (17.1 μL, 0.075 mmol, 1.5 equiv) was added via syringe. The reaction was stirred for 48 hours and then quenched by the addition of MeOH/Et_3N (0.2 mL, v/v = 3:1) via syringe. The mixture was stirred at –78 °C for 5 minutes before warming to room temperature. The crude reaction mixture was concentrated and CH_2Br_2 (7.0 μL, 17.4 mg, 0.1 mmol) was added. The yield was determined by ^{1}H NMR of the crude mixture. And then the mixture was concentrated and purified by silica gel flash chromatography (*n*-hexane/EtOAc = 7:1 to 5:2). The yield ratio (**4b**-d_2 or **4b**-d_1/**4b**) was measured by ^{1}H NMR. The spectrogram is listed below.

(a) Competition experiment between 2b-d_2 and 2b

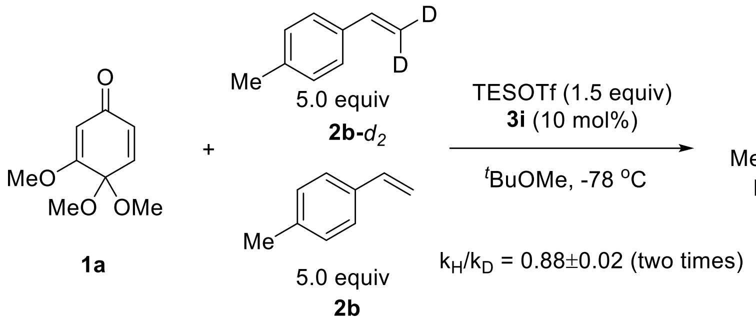

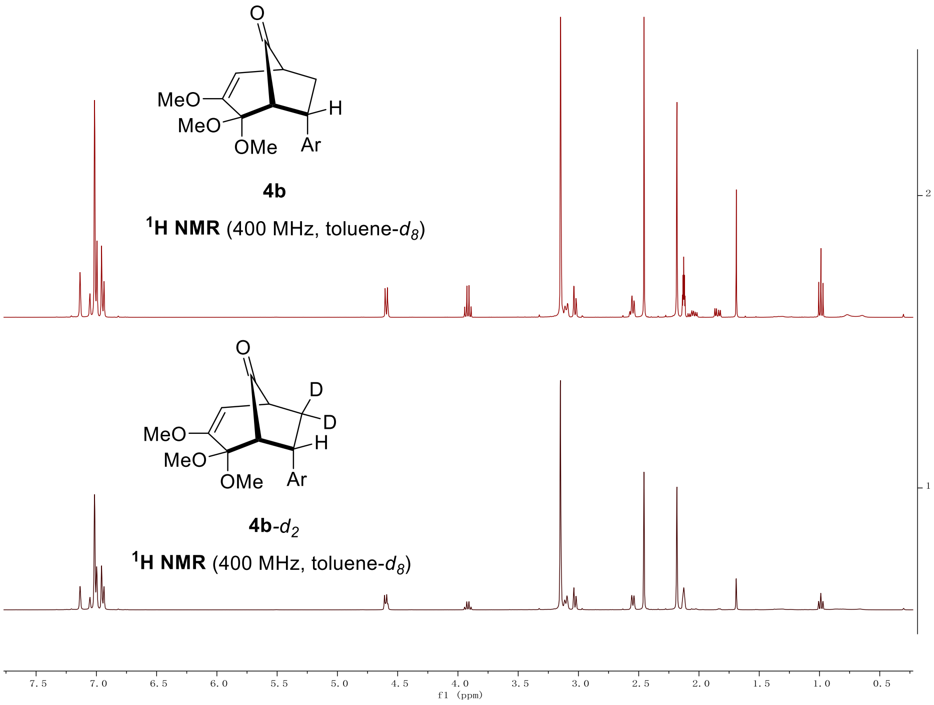

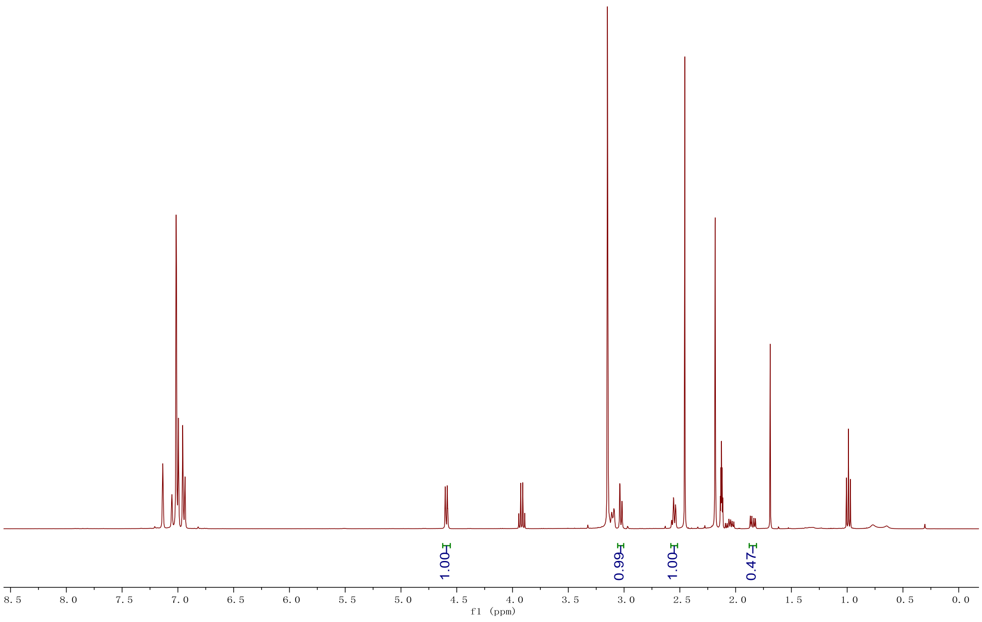

(b) Competition experiment between 2b-d_1 and 2b

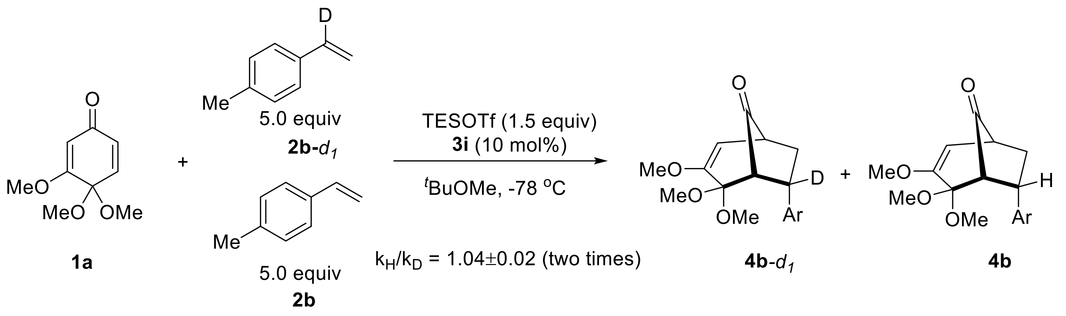

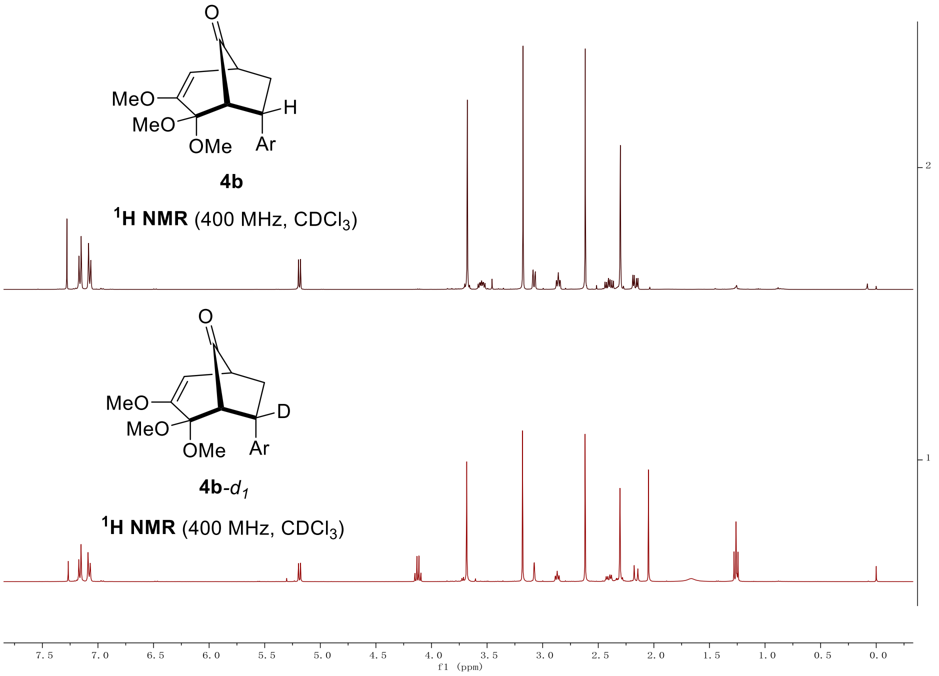

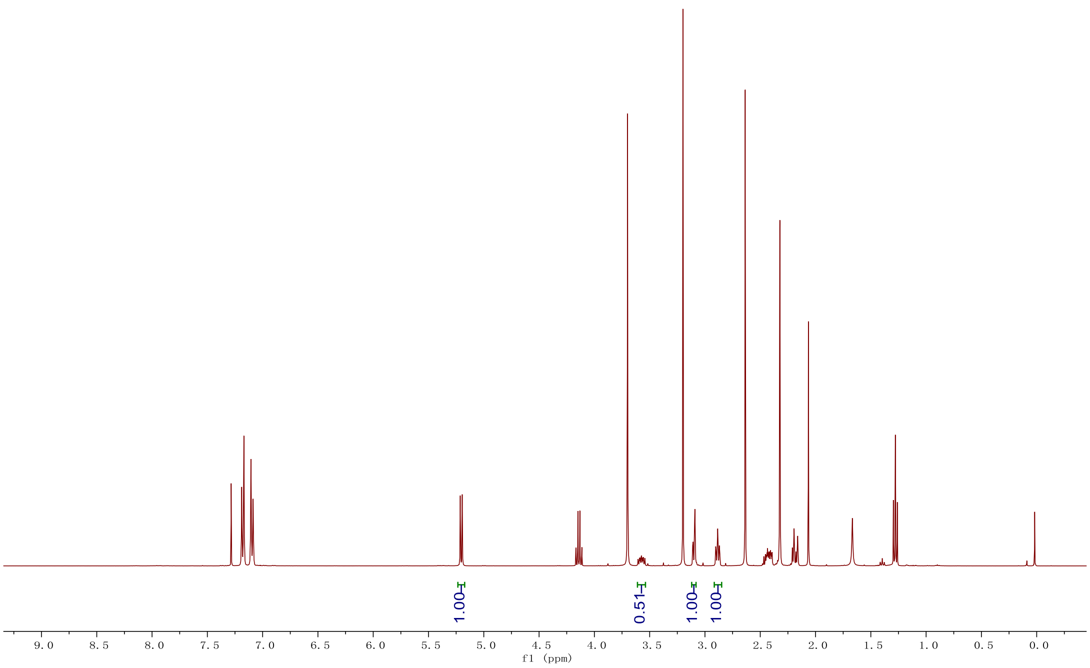

VIII. Determination of Product Structures

The structure and absolute stereochemistry of compounds **4b'**, **11**, **14** and **16** were determined by single-crystal X-ray diffraction. The data have been deposited at the Cambridge Crystallographic Data Centre (**4b'**, CCDC 2413151; **11**, CCDC 2413152; **14**, CCDC 2413153; **16** CCDC 2413154) and are available free of charge at www.ccdc.cam.ac.uk/conts/retrieving.html.

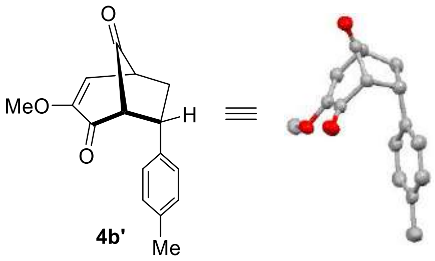

Table S5. Crystal Data and Structure Refinement for 4b'

Identification code	**4b'**
Empirical formula	$C_{16}H_{16}O_3$
Formula weight	256.29

Temperature/K	100.00
Crystal system	monoclinic
Space group	$P2_1$
a/Å	6.8898(5)
b/Å	9.9175(8)
c/Å	9.9355(8)
α/°	90
β/°	104.257(3)
γ/°	90
Volume/Å^3	657.98(9)
Z	2
ρ_{calc}g/cm^3	1.294
μ/mm^{-1}	0.457
F(000)	272.0
Crystal size/mm^3	0.125 × 0.115 × 0.02
Radiation	GaKα (λ = 1.34139)
2Θ range for data collection/°	7.988 to 114.214
Index ranges	$-8 \le h \le 8$, $-12 \le k \le 12$, $-12 \le l \le 11$
Reflections collected	17222
Independent reflections	2683 [R_{int} = 0.0972, R_{sigma} = 0.0759]
Data/restraints/parameters	2683/1/175
Goodness-of-fit on F^2	1.051
Final R indexes [I>=2σ (I)]	R_1 = 0.0623, wR_2 = 0.1575
Final R indexes [all data]	R_1 = 0.0968, wR_2 = 0.1750
Largest diff. peak/hole / e Å^{-3}	0.27/-0.24
Flack parameter	0.0(4)

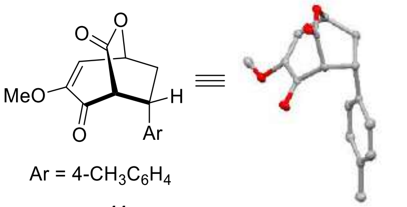

11

Table S6. Crystal Data and Structure Refinement for 11

Identification code	**11**
Empirical formula	$C_{16}H_{16}O_4$
Formula weight	272.29
Temperature/K	100.00
Crystal system	monoclinic
Space group	$P2_1$
a/Å	6.9729(3)
b/Å	10.2523(4)
c/Å	10.0126(3)
α/°	90
β/°	107.584(2)
γ/°	90
Volume/Å^3	682.34(5)
Z	2
ρ_{calc}g/cm^3	1.325
μ/mm^{-1}	0.498
F(000)	288.0
Crystal size/mm^3	0.07 × 0.06 × 0.03
Radiation	GaKα (λ = 1.34139)
2Θ range for data collection/°	11.018 to 118.372
Index ranges	$-8 \le h \le 8$, $-13 \le k \le 13$, $-12 \le l \le 12$
Reflections collected	12151

Independent reflections	2982 [R_{int} = 0.0666, R_{sigma} = 0.0521]
Data/restraints/parameters	2982/1/183
Goodness-of-fit on F^2	1.097
Final R indexes [I>=2σ (I)]	R_1 = 0.0470, wR_2 = 0.1089
Final R indexes [all data]	R_1 = 0.0506, wR_2 = 0.1107
Largest diff. peak/hole / e $Å^{-3}$	0.28/-0.25
Flack / Hooft's parameter	0.1(2) / 0.02(16)

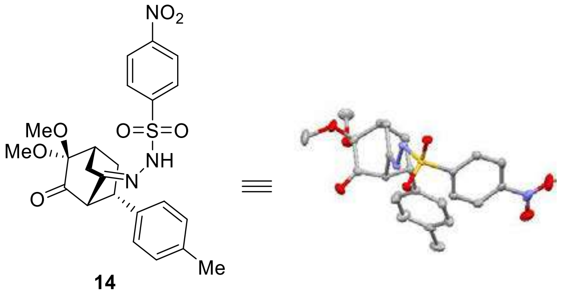

14

Table S7. Crystal Data and Structure Refinement for 14

Identification code	**14**
Empirical formula	$C_{23}H_{25}N_3O_7S$
Formula weight	487.52
Temperature/K	99.97(10)
Crystal system	orthorhombic
Space group	$P2_12_12_1$
a/Å	7.55543(6)
b/Å	11.77316(10)
c/Å	25.6317(2)
α/°	90
β/°	90
γ/°	90
Volume/Å^3	2279.97(3)
Z	4
ρ_{calc}g/cm^3	1.420
μ/mm^{-1}	1.702
F(000)	1024.0
Crystal size/mm^3	0.25 × 0.12 × 0.1
Radiation	Cu Kα (λ = 1.54184)
2Θ range for data collection/°	6.898 to 148.778
Index ranges	-9 ≤ h ≤ 7, -14 ≤ k ≤ 14, -31 ≤ l ≤ 30

Reflections collected	13082
Independent reflections	4582 [R_{int} = 0.0181, R_{sigma} = 0.0180]
Data/restraints/parameters	4582/0/310
Goodness-of-fit on F^2	1.048
Final R indexes [I>=2σ (I)]	R_1 = 0.0274, wR_2 = 0.0731
Final R indexes [all data]	R_1 = 0.0278, wR_2 = 0.0735
Largest diff. peak/hole / e Å^{-3}	0.31/-0.20
Flack parameter	0.000(6)

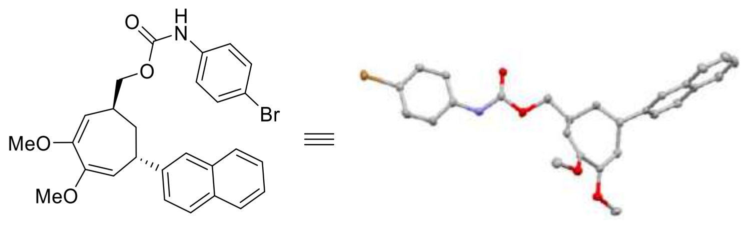

rac-**16**

Table S8. Crystal Data and Structure Refinement for *rac*-16

Identification code	*rac*-**16**
Empirical formula	$C_{27}H_{26}BrNO_4$
Formula weight	508.40
Temperature/K	100.00(10)
Crystal system	triclinic
Space group	P-1
a/Å	9.3777(4)
b/Å	12.3613(5)
c/Å	31.3321(12)
α/°	79.734(3)
β/°	84.161(3)
γ/°	83.236(3)
Volume/Å^3	3536.9(2)
Z	6
ρ_{calc}g/cm^3	1.432
μ/mm^{-1}	2.652
F(000)	1572.0
Crystal size/mm^3	0.25 × 0.03 × 0.03
Radiation	CuKα (λ = 1.54184)
2Θ range for data collection/°	5.752 to 150.328
Index ranges	$-10 \le h \le 11$, $-15 \le k \le 15$, $-38 \le l \le 37$
Reflections collected	21696

Independent reflections	13766 [R_{int} = 0.0783, R_{sigma} = 0.1358]
Data/restraints/parameters	13766/370/1006
Goodness-of-fit on F^2	1.007
Final R indexes [I>=2σ (I)]	R_1 = 0.0591, wR_2 = 0.1164
Final R indexes [all data]	R_1 = 0.1020, wR_2 = 0.1412
Largest diff. peak/hole / e Å^{-3}	0.81/-1.01

IX. DFT Calculations

Computational details

Calculations were performed using the Gaussian 16 (rev. C.01)[14] program on the HPC cluster at Hong Kong University of Science and Technology. The geometries of all ground-state and transition structures were fully optimized at the B3LYP/6-31G(d)[15] level of theory for all atoms, without solvent correction. The dispersion energy corrections by D3 version Grimme's dispersion with original D3[16] damping function were used. Default optimization convergence criteria, tight SCF convergence criteria, and default integration settings were used. Stationary points were characterized by the presence of all positive eigenvalues of the Hessian for minima or a single negative eigenvalue for transition structures. All molecular structures were rendered in CYLView20.[17] Single point energies were evaluated at the M062X[18]-D3/6-31+G(d,p) level of theory, and the reported energies are uncorrected electronic energies. The independent gradient model based on Hirshfeld partition (IGMH) analysis was performed using the software Multiwfn 3.8(dev)[19] to study the origin of the enantioselectivity. The isosurface was visualized using VMD (version1.9.3)[20] with the isovalue of 0.007.

Table S9. Energies of all calculated stationary structures of pentadienyl cationic [5+2] cycloaddition process from **INT1** to **INT3** (Hartrees).

Entry	Gibbs energy corrections	Single-point energies	Gibbs energy	ΔG
**INT1**	0.2503000	-844.9573751	-844.7070751	0.0
**TS1**	0.2483010	-844.9522845	-844.7039835	1.9
**TS2**	0.2478550	-844.9455258	-844.6976708	5.9
**TS3**	0.2487160	-844.9417309	-844.6930149	8.8
**TS4**	0.2491420	-844.9355132	-844.6863712	13.0
**INT2**	0.2530880	-844.9683702	-844.7152822	-5.2
**INT2'**	0.2513880	-844.9668537	-844.7154657	-5.3
**TSrc1**	0.2538850	-844.9675981	-844.7137131	-4.2
**TSrc2**	0.2532580	-844.965722	-844.712464	-3.4
endo-**INT3**	0.2558880	-844.999127	-844.743239	-22.7
exo-**INT3**	0.2545700	-844.9934407	-844.7388707	-19.9

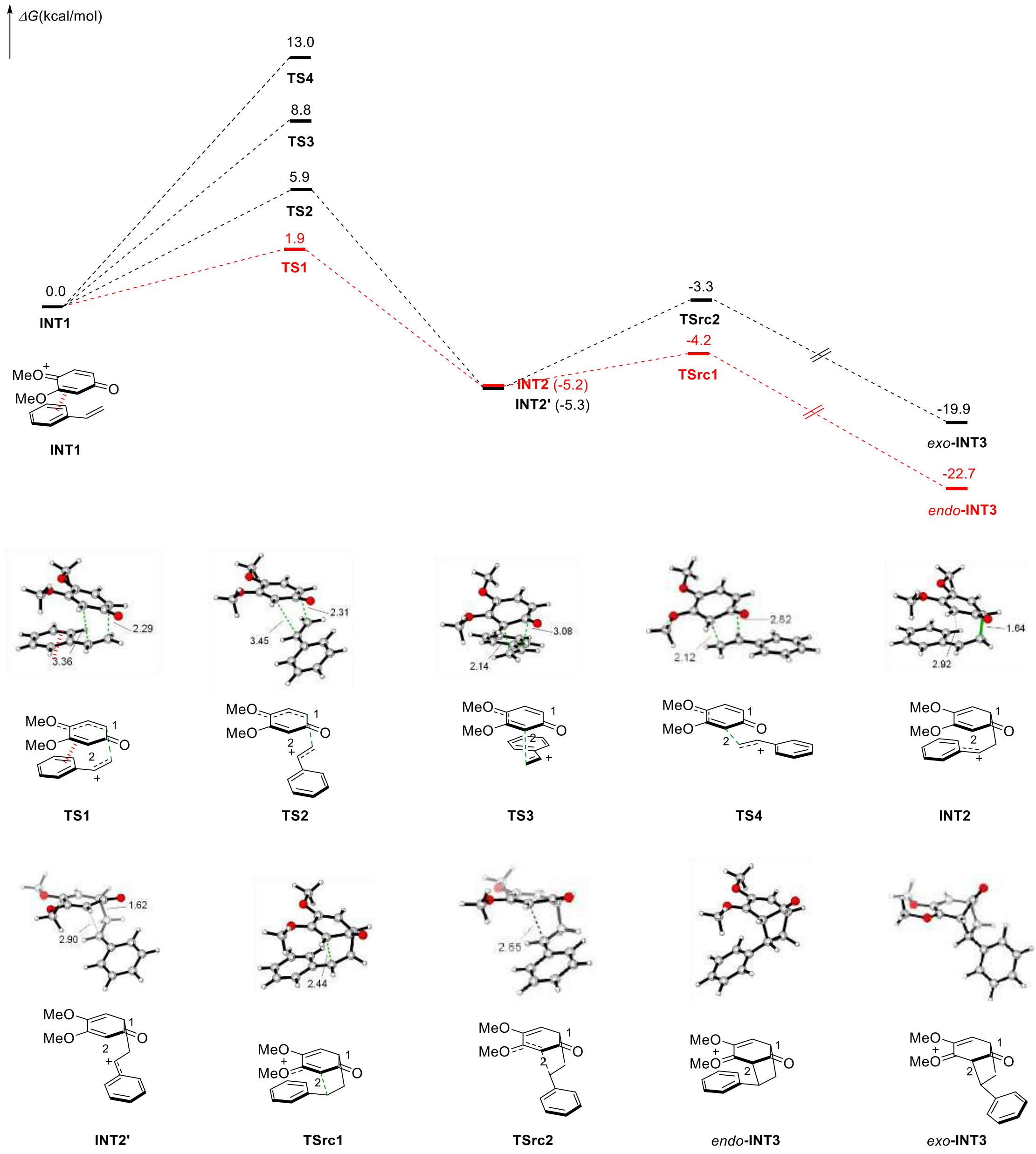

Figure S1. Free energy diagram for the [5+2] cycloaddition of pentadienyl cation and styrene.

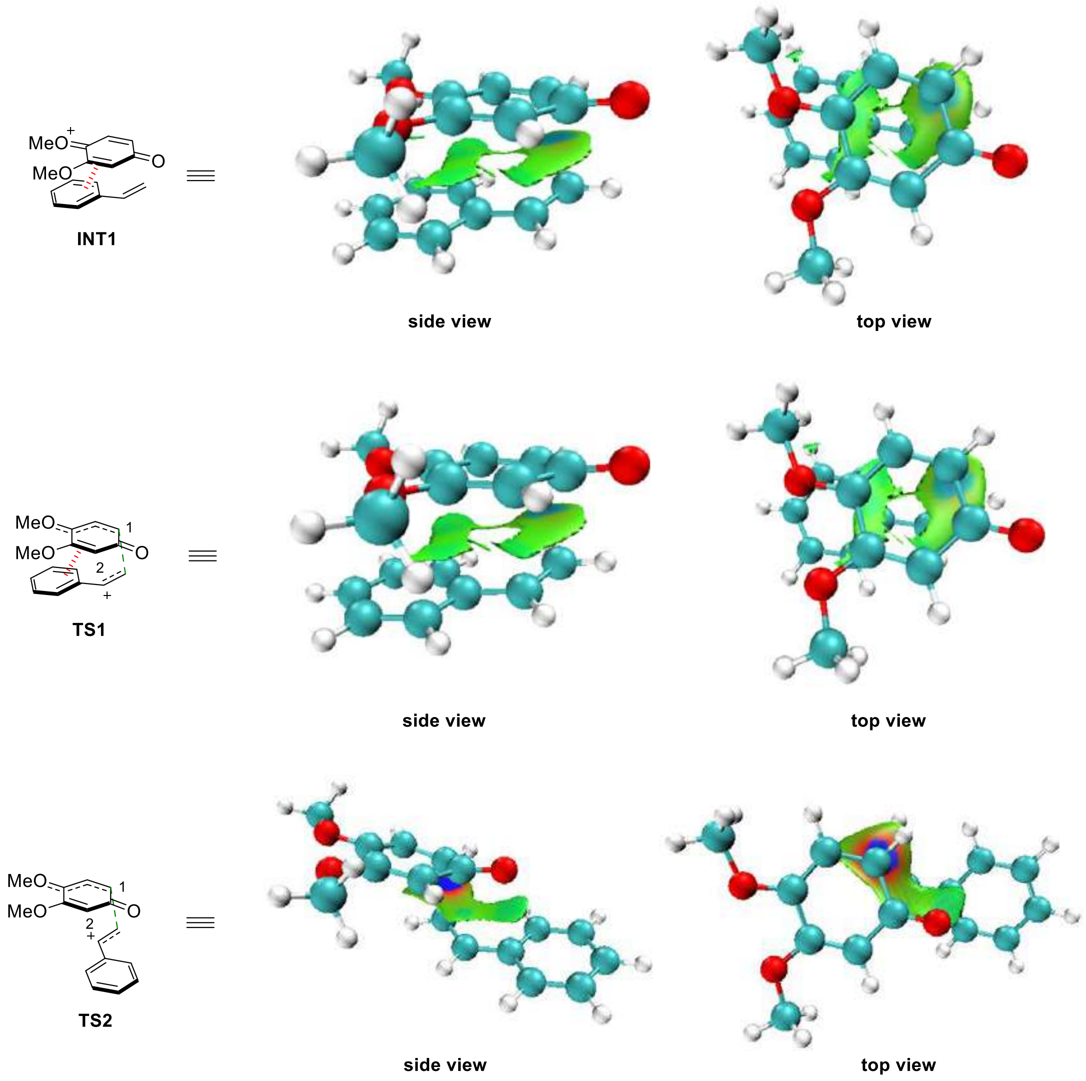

Figure S2. IGMH analysis of the noncovalent interactions in **INT1**, **TS1** and **TS2**. **TS1** has a stronger van der Waals interaction between pentadienyl cation and styrene than **TS2**.

Table S10. Energies of all calculated stationary structures of asymmetric [5+2] cycloaddition between **1a** and **2a** using **3i** as the catalyst.

Entry	Gibbs energy corrections	Single-point energies	Gibbs energy	ΔG
**1a**	0.16255200	-650.7678	-650.6053	---
**2a**	0.10229100	-309.5203	-309.4180	---
**TMSOTf**	0.09847900	-1370.4373	-1370.3388	---
**3i**	0.580925	-2494.262344	-2493.681419	---
**1a+2a+TMSOTf +3i**	---	---	---	0.0
**IM1**	0.704546	-3864.728506	-3864.02396	-2.3
**TMSOMe**	0.119258	-524.2912597	-524.1720017	---
**IM2**	0.74810200	-3991.1929	-3990.4448	5.5
**IM3**	0.875019	-4300.748216	-4299.873197	-1.1
$\mathbf{TS_{34\text{-}major}}$	0.87855300	-4300.7470	-4299.868431	1.9
**IM4**	0.879939	-4300.751584	-4299.871645	-0.1
$\mathbf{TS_{45}}$	0.880664	-4300.750966	-4299.870302	0.73
**IM5**	0.88206	-4300.783921	-4299.901861	-19.1
**IM6**	0.274014	-1806.46216	-1806.188146	-13.2

To further elucidate the mechanisms, we conducted DFT calculations. Supplementary Figure 3 depicts the energy profile for the most favorable pathway, which mainly consists of three steps, pentadienyl cation formation, cycloaddition, and product release. First, a more thermodynamically stable chiral Lewis acid complex **IM1** is formed as a resting-state complex, which is consistent with the experiment results and previous report.[21] This complex then activates the substrate by forming a pentadienyl cation intermediate **IM2** via dissociation of the methoxyl group of the ketal. The energy level intermediate **IM1** was calculated to be 5.5 kcal/mol. The barrier for this dissociation is expected to be higher than 7.8 kcal/mole (the difference between **IM1**

and **IM2**), which make it certain that this step is rate-determining since the following steps are all facile. This is consistent with the kinetic results.

Next, **IM2** and **2a** form a relatively stable adduct **IM3**, setting the stage for [5+2] cycloaddition. The calculation suggests that this cycloaddition is a stepwise with one C–C bond formed first, which is consistent with the competitive Hammett correlation (log (ν_X/ν_H) vs σ^+) and second order kinetic isotope results. The enantio-determining carbon-carbon bond formation transition state **TS**$_{34}$ has a higher energy barrier than ring closure **TS**$_{45}$, which also is consistent with the competitive Hammett correlation outcome. The final step releases the cycloadduct **IM6** by exchange with another molecule of TMSOTf to complete the catalytic cycle.

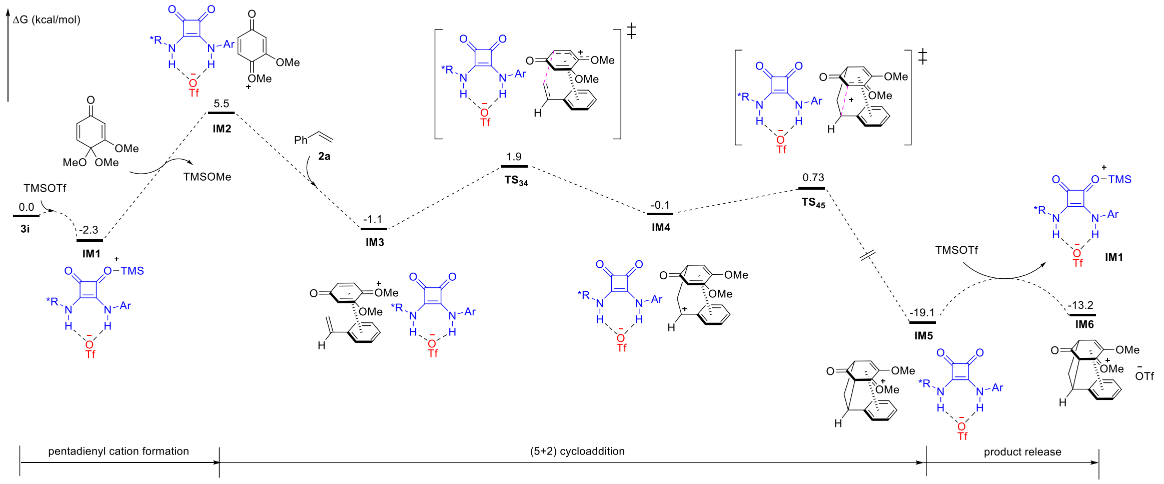

Figure S3. Free energy diagram for asymmetric [5+2] cycloaddition between **1a** and **2a** using **3i** as the catalyst. M062X-D3/6-31+G(d,p)//B3LYP-D3BJ/6-31G(d) level of theory for all atoms without solvent correction.

Table S11. Energies of all 8 transition structures leading to the major and minor products (Hartrees).

Entry	Gibbs energy corrections	Single-point energies	Gibbs energy	ΔG
**A1($TS_{34\text{-}major}$)**	0.87855300	-4300.7469845	-4299.8684315	0.0
**A2**	0.87984700	-4300.7386935	-4299.8588465	6.0
**A3**	0.87584200	-4300.7338017	-4299.8579597	6.6
**A4**	0.87469600	-4300.7312315	-4299.8565355	7.5
**B1($TS_{34\text{-}minor}$)**	0.87646400	-4300.7411431	-4299.8646791	2.4
**B2**	0.87817300	-4300.7391235	-4299.8609505	4.7
**B3**	0.87794800	-4300.7388217	-4299.8608737	4.7
**B4**	0.88003800	-4300.7386300	-4299.8585920	6.2

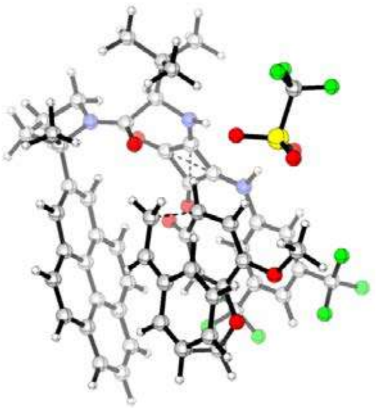

A1

0.0 kcal/mol

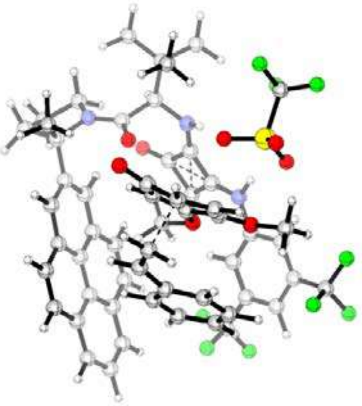

A2

6.0 kcal/mol

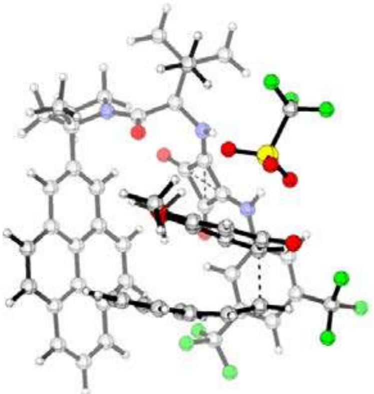

A3

6.6 kcal/mol

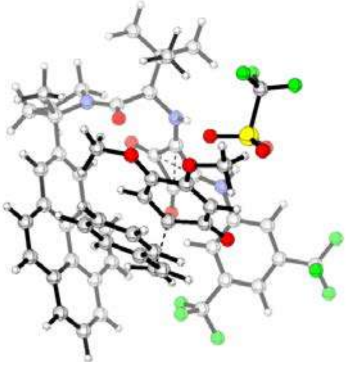

A4

7.5 kcal/mol

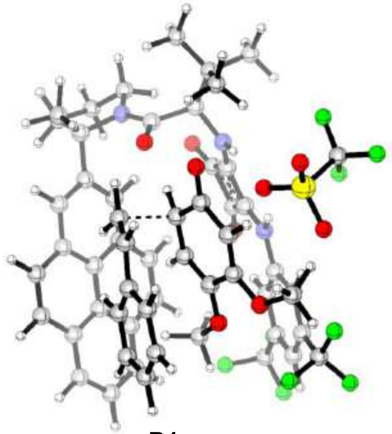

B1

2.4 kcal/mol

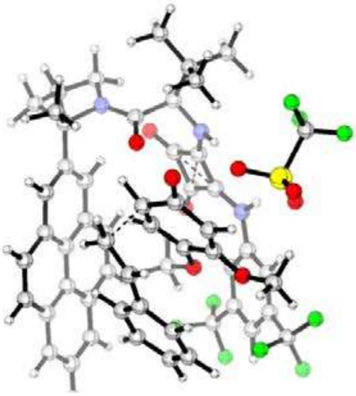

B2

4.7 kcal/mol

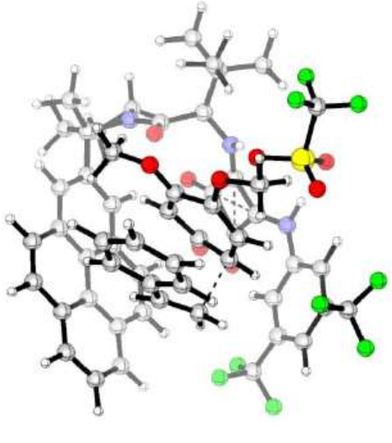

B3

4.7 kcal/mol

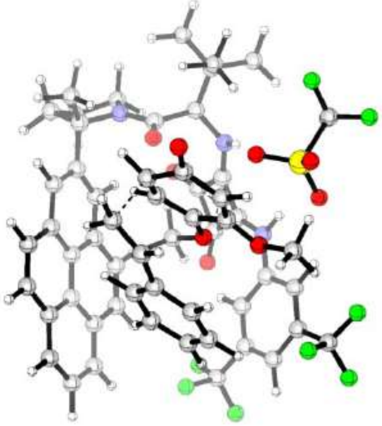

B4

6.2 kcal/mol

Figure S4. Computational analysis of 8 transition structures leading to the major and minor products. M062X-D3/6-31+G(d,p) //B3LYP-D3BJ/6-31G(d) level of theory for all atoms without solvent correction; All molecular structures were rendered in CYLView20.

Table S12. Computed KIEs for compounds **2a** and **2a**-d_2 using M062X-D3/6-31+G(d,p)//B3LYP-D3BJ/6-31G(d) level of theory for all atoms (298 K).

entry	Gibbs energy corrections	single-point energies	Gibbs energy	ΔG
**IM3**	0.875019	-4300.748216	-4299.873197	0.0
**TS**$_{\mathbf{34\text{-}major}}$	0.87855300	-4300.746984	-4299.868431	2.99
**IM3**(**2a**-d_2)	0.868274	-4300.748216	-4299.879942	0.0
**TS**$_{\mathbf{34\text{-}major}}$(**2a**-$d_2$)	0.871631	-4300.746984	-4299.875353	2.88
ΔΔG				0.11

$k_H/k_D = \frac{v_H}{v_D} \times e^{\frac{-\Delta\Delta G}{RT}}$

The Gibbs free energy difference between the unlabeled substrate **2a** and its deuterated analog **2a**-d_2 is 0.11 kcal/mol. Using the Arrhenius equation at 298 K, the predicted KIE is 0.830. Furthermore, the imaginary frequencies of the corresponding transition states are -416.59 cm^{-1} for **2a** and -415.48 cm^{-1} for **2a**-d_2. After frequency correction, the corrected KIE value is 0.832. These computed values are consistent with experimental results.

36

INT1

C	-2.00761400	0.97556000	-0.29993900
C	-0.78314900	1.34003300	0.16775700
C	-0.12350500	0.52337400	1.20311300
C	-0.72399000	-0.65982000	1.68947200
C	-1.91748100	-1.07896400	1.15709900
C	-2.68934800	-0.21464600	0.21166500
O	-3.83777800	-0.50371000	-0.09458700
O	-0.06818400	2.40808000	-0.18603400
C	-0.62758700	3.29479500	-1.16511800
O	0.98959100	1.03216300	1.65209400
C	1.68432400	0.42732900	2.76869500
H	-2.55336500	1.56830000	-1.02281600
H	-0.22840300	-1.23534300	2.46064300
H	-2.44780000	-1.93566500	1.55849800
H	0.11037400	4.08410200	-1.29733400
H	-0.79010700	2.76649400	-2.11098400
H	-1.57103500	3.71734300	-0.80520400
H	2.56339800	1.05007700	2.91896500
H	1.04210300	0.44936700	3.65242900
H	1.97702600	-0.59241900	2.51587500
C	2.81355900	-1.34804300	0.04144300
C	3.26367900	-0.18829500	-0.59811700
C	2.45655800	0.44857700	-1.54808600
C	1.20589700	-0.06874400	-1.84895100
C	0.71496400	-1.21809600	-1.18875400
C	1.54783400	-1.85099900	-0.23436300

C	-0.62643800	-1.67124900	-1.47937900
C	-1.31544300	-2.61696700	-0.78435100
H	3.45790200	-1.86315300	0.74756800
H	4.25007800	0.20597200	-0.37453100
H	2.81237200	1.34032400	-2.05317700
H	0.57974100	0.41663000	-2.59221300
H	1.21701000	-2.76163400	0.25318200
H	-1.14199500	-1.15085400	-2.28321900
H	-2.33527800	-2.86808500	-1.05535400
H	-0.84460900	-3.24679000	-0.03722000

36

TS2

C	0.71112500	0.84238200	0.91953700
C	1.92617500	0.95700800	0.31003600
C	2.71191100	-0.25619300	-0.00467000
C	2.19728700	-1.52709600	0.24481200
C	0.91758600	-1.66315600	0.78897300
C	0.18183400	-0.46255700	1.29859100
O	-0.82062200	-0.60115800	1.99518400
O	2.54390400	2.08215400	-0.04514600
C	1.91048700	3.33608800	0.25161200
O	3.90362300	-0.00034500	-0.49540100
C	4.80713100	-1.07340400	-0.83574300
H	0.12483200	1.70535900	1.20920600
H	2.78951800	-2.40764500	0.02634200
H	0.61836900	-2.61070200	1.22336400

H	2.59452100	4.09914300	-0.11669500
H	0.94803400	3.41226100	-0.26552400
H	1.76844800	3.44677900	1.33164000
H	5.69931000	-0.57977900	-1.21643600
H	5.04904100	-1.65833300	0.05608200
H	4.36626800	-1.70877400	-1.60916200
C	-4.30622300	-0.37497100	0.71477600
C	-4.75650200	0.79447700	0.09400100
C	-4.03218400	1.36310500	-0.96491000
C	-2.86147400	0.76262900	-1.39692400
C	-2.38982400	-0.42912200	-0.78814000
C	-3.13848900	-0.98819600	0.28135100
C	-1.18131800	-1.01763900	-1.29074100
C	-0.58683300	-2.19581400	-0.88031400
H	-4.87059000	-0.80757400	1.53451000
H	-5.67548700	1.26478800	0.43140100
H	-4.39040000	2.26885500	-1.44403200
H	-2.29793500	1.19570900	-2.21942300
H	-2.80251300	-1.89608100	0.76740600
H	-0.68859000	-0.46559000	-2.09034500
H	0.19983400	-2.62939800	-1.48669900
H	-1.11202900	-2.87948000	-0.22222700

36

TS3

C	-2.08667400	-0.57522200	-0.30409600
C	-1.55689700	0.72534100	-0.25994900

C	-0.71545700	1.08705900	0.82444700
C	-0.43085900	0.14058500	1.87654400
C	-1.01588800	-1.08403800	1.91799200
C	-2.04346500	-1.44352700	0.92106100
O	-2.79901300	-2.38949900	1.05664500
O	-1.72064900	1.66199600	-1.21099200
C	-2.78784100	1.51499100	-2.16192800
O	-0.26844800	2.33127500	0.81862100
C	0.52741600	2.84598300	1.90473000
H	-2.91803400	-0.78804300	-0.96806000
H	0.25905300	0.43532400	2.65926200
H	-0.84331300	-1.77797900	2.73405800
H	-2.78244500	2.43658600	-2.74272900
H	-2.61559500	0.66338600	-2.82882500
H	-3.74984200	1.40433100	-1.65026600
H	0.70321000	3.89022800	1.65140500
H	-0.01979300	2.77929000	2.84941000
H	1.47974700	2.31178500	1.96767200
C	2.73505700	0.87210300	-1.18164600
C	3.85015300	0.43711900	-0.45841200
C	3.82648300	-0.79838100	0.20732900
C	2.69294400	-1.59181300	0.14881300
C	1.55445800	-1.18002000	-0.59387700
C	1.59813100	0.07449000	-1.25790300
C	0.40917700	-2.03950200	-0.63717400
C	-0.74297400	-1.82853700	-1.39556800
H	2.76107700	1.82515400	-1.70078300
H	4.74248800	1.05494100	-0.41720500
H	4.69678000	-1.13275200	0.76334500

H	2.67086700	-2.54999200	0.66124100
H	0.75373500	0.41641900	-1.84662900
H	0.45313100	-2.94109100	-0.02931500
H	-1.45871900	-2.64125700	-1.47177400
H	-0.71004800	-1.15629800	-2.24717500

36

TS4

C	-0.98819400	-1.13887300	0.44711000
C	-2.26367900	-0.64126500	0.10633100
C	-2.48774800	0.75480900	0.15561500
C	-1.43280200	1.64070800	0.54375500
C	-0.22161900	1.17990800	0.97375900
C	-0.02450900	-0.26040800	1.18116600
O	0.88803100	-0.72630900	1.84577200
O	-3.27936200	-1.38034300	-0.35667000
C	-3.18144200	-2.80982900	-0.30075700
O	-3.69857500	1.15719900	-0.20516800
C	-4.06478800	2.54536000	-0.11826700
H	-0.86917900	-2.19798400	0.64584900
H	-1.61239800	2.70867900	0.49658600
H	0.56335000	1.84901800	1.30782500
H	-4.13348900	-3.17773500	-0.68001200
H	-2.36748700	-3.17411100	-0.93679900
H	-3.03611800	-3.14874900	0.73056000
H	-5.11492700	2.57796900	-0.40294000
H	-3.94268000	2.91260300	0.90505600

H	-3.47265600	3.14491200	-0.81664800
C	4.23251600	-0.95003400	0.41253800
C	4.96582100	0.23093600	0.27093400
C	4.40764300	1.34170300	-0.38004800
C	3.12085100	1.26887600	-0.88275900
C	2.36129200	0.07768100	-0.75721300
C	2.94407600	-1.03386700	-0.09616300
C	1.03286900	0.05809500	-1.28423300
C	0.16781400	-1.05187800	-1.32498700
H	4.66887600	-1.80397100	0.91953100
H	5.97535900	0.28974700	0.66563700
H	4.98407500	2.25468600	-0.48716500
H	2.68088300	2.12544000	-1.38620100
H	2.38809000	-1.95643800	0.01727900
H	0.68600500	0.97558300	-1.75191800
H	-0.67141400	-1.01416100	-2.00981200
H	0.58434900	-2.04140600	-1.16898000

36

INT2

C	-1.75162800	0.32913800	-1.16259000
C	-0.65144100	1.05131000	-0.78350800
C	-0.34312700	1.26373000	0.64175700
C	-1.03647800	0.57611900	1.59539800
C	-2.06494000	-0.40964400	1.22275700
C	-2.67460900	-0.20916100	-0.16424500
O	-3.79250000	-0.61249000	-0.42561100

O	0.22746100	1.62148100	-1.60043900
C	0.01085600	1.53277300	-3.01650000
O	0.61856000	2.16699600	0.84661300
C	0.95411200	2.50220000	2.19645600
H	-2.04504300	0.23311600	-2.20059300
H	-0.83690100	0.73827300	2.64780500
H	-2.85457600	-0.48042300	1.97413400
H	0.84126800	2.07116400	-3.47010100
H	0.01651700	0.48658400	-3.33987700
H	-0.93768500	2.00510600	-3.28878800
H	1.73529600	3.25735700	2.12463900
H	0.08377300	2.91190000	2.71966800
H	1.33076300	1.62234700	2.73014600
C	2.70496200	-0.67534000	1.10824100
C	3.37260500	-0.62855500	-0.11859300
C	2.77340300	-1.12861300	-1.29023400
C	1.50194700	-1.65674100	-1.23390200
C	0.78118300	-1.68420400	-0.00112600
C	1.42325200	-1.20185700	1.17740000
C	-0.56565000	-2.07379800	-0.00640600
C	-1.47804700	-1.94051800	1.12877300
H	3.19720100	-0.31839200	2.00660600
H	4.37660800	-0.21811400	-0.16679700
H	3.31423800	-1.09693800	-2.22996400
H	1.01753000	-2.03184700	-2.13080600
H	0.92854200	-1.29036000	2.13585700
H	-0.97136500	-2.46184100	-0.93723100
H	-2.35959200	-2.57190600	1.00860300
H	-1.00313400	-2.14496100	2.09011400

36

INT2'

C	-0.49955200	0.46684500	-1.15669100
C	-1.63315500	0.86628900	-0.48826200
C	-2.55224600	-0.12005900	0.10976100
C	-2.20981100	-1.43266100	0.12327000
C	-0.91880900	-1.90147300	-0.43140200
C	-0.21928200	-0.93943000	-1.38855500
O	0.65571200	-1.33665100	-2.14632500
O	-2.02196600	2.12096200	-0.31479300
C	-1.24483800	3.18046900	-0.89392600
O	-3.67543100	0.42826800	0.58914400
C	-4.65209200	-0.44254500	1.16677700
H	0.16005500	1.17833200	-1.63716100
H	-2.86796500	-2.17297900	0.56222500
H	-1.01759800	-2.88787600	-0.89201400
H	-1.76614700	4.09810200	-0.62713500
H	-0.23294900	3.18563400	-0.47568200
H	-1.20312500	3.07143900	-1.98178800
H	-5.47425200	0.20246600	1.47312700
H	-5.00315000	-1.17192800	0.42845300
H	-4.23999300	-0.96257100	2.03924100
C	4.16749800	-0.42471500	-0.42590800
C	4.56746400	0.77341700	0.18141100
C	3.73029000	1.44120600	1.09267700
C	2.49182100	0.91231300	1.38981500
C	2.06677400	-0.31683200	0.79779700

C	2.93768900	-0.97962500	-0.11940000
C	0.78933700	-0.80256700	1.11161600
C	0.21595800	-2.09370000	0.70331700
H	4.82422400	-0.91956500	-1.13301700
H	5.54043900	1.19351500	-0.05530400
H	4.05840700	2.36674400	1.55344600
H	1.82756300	1.41837700	2.08457300
H	2.62693200	-1.89766800	-0.60058700
H	0.17608600	-0.18371800	1.76408500
H	-0.26202800	-2.57877700	1.55944400
H	0.96470800	-2.76177300	0.28009300

36

TSrc1

C	1.43665200	-0.91595500	0.97095600
C	0.69633600	0.23886500	1.16073100
C	0.83548500	1.37088600	0.24177900
C	1.57952200	1.21800400	-0.89034600
C	2.22953800	-0.07157600	-1.21416700
C	2.57251200	-0.89180300	0.02431200
O	3.56386700	-1.57629000	0.13158800
O	-0.20969000	0.42167500	2.10169000
C	-0.40395400	-0.58788200	3.11012300
O	0.17894300	2.46751400	0.63807100
C	0.25616900	3.63004600	-0.19149200
H	1.41141700	-1.72213600	1.69423500
H	1.68669500	2.03456100	-1.59447000

H	3.09886900	0.05691400	-1.86098100
H	-1.17055200	-0.18613000	3.76993000
H	-0.74954500	-1.51905900	2.65351000
H	0.52442800	-0.75258100	3.66389900
H	-0.32949900	4.39313700	0.31876700
H	1.29424600	3.96384100	-0.29622900
H	-0.17299700	3.42807200	-1.18006900
C	-2.77790500	0.62608700	-1.25611000
C	-3.66467400	-0.04569900	-0.40931400
C	-3.29277200	-1.25250300	0.20312000
C	-2.03167700	-1.77401500	-0.02014100
C	-1.10954600	-1.10285400	-0.86677300
C	-1.51429800	0.10329900	-1.49395500
C	0.20292100	-1.63210800	-1.01368000
C	1.22696300	-1.09777800	-1.94778600
H	-3.08478300	1.54627400	-1.74200900
H	-4.65592700	0.36147100	-0.23576900
H	-3.99455600	-1.77434400	0.84522500
H	-1.73116400	-2.70545300	0.45231100
H	-0.84582000	0.60991400	-2.17869600
H	0.37429300	-2.62541900	-0.60906100
H	1.85323200	-1.91509500	-2.31355100
H	0.77907000	-0.58725100	-2.80138100

36

TSrc2

C	0.43638700	0.33525300	1.10173000

C	1.57543100	0.83000100	0.49961000
C	2.56374000	-0.07616400	-0.10572300
C	2.28137400	-1.40134600	-0.19758400
C	0.99165600	-1.95297200	0.28092000
C	0.27330400	-1.09999000	1.32208500
O	-0.53255500	-1.57507600	2.10295700
O	1.89279500	2.11001100	0.39622500
C	1.03358400	3.09902000	0.98799900
O	3.67473400	0.54767000	-0.51616000
C	4.71142100	-0.24707200	-1.10027100
H	-0.27435300	0.98660400	1.59440600
H	2.98597100	-2.08701800	-0.65271600
H	1.09598100	-2.98431800	0.62461600
H	1.50891200	4.05490900	0.77571300
H	0.03861300	3.06212000	0.53301500
H	0.96236500	2.94297000	2.06837000
H	5.51222100	0.44986800	-1.34262800
H	5.07061800	-0.99699300	-0.38688200
H	4.35509100	-0.73871200	-2.01276400
C	-4.17107900	-0.55122700	0.34971400
C	-4.60795100	0.68211700	-0.15126500
C	-3.77041400	1.47197900	-0.95595900
C	-2.49626600	1.02976200	-1.25253800
C	-2.03613100	-0.22996200	-0.76988300
C	-2.90399000	-1.01630500	0.04063600
C	-0.71972100	-0.62937100	-1.08137800
C	-0.14083900	-1.97286700	-0.86251000
H	-4.82744600	-1.14482500	0.97678100
H	-5.60801000	1.03247500	0.08549100

H	-4.12409700	2.42351300	-1.33841800
H	-1.83372900	1.63211400	-1.86797100
H	-2.56791600	-1.96373400	0.44260800
H	-0.12472300	0.06561200	-1.66900400
H	0.32220200	-2.32963900	-1.78709500
H	-0.89452700	-2.69092700	-0.54120100

36

***endo*-INT3**

C	0.51184700	-1.14744900	0.79348100
C	0.78495000	0.29766800	0.87959300
C	1.71303600	0.91433600	-0.04147500
C	2.33092900	0.11570600	-0.96749800
C	1.99695200	-1.33111200	-1.10448400
C	1.81534300	-1.86115500	0.32830400
O	2.42719300	-2.69088400	0.92609400
O	0.26528300	1.08419700	1.75926600
C	-0.75145100	0.63558100	2.70352000
O	1.88652900	2.22879700	0.15048400
C	2.83251300	2.91037400	-0.68397300
H	0.11871400	-1.55115900	1.72501000
H	3.07588200	0.53447300	-1.63456600
H	2.76289400	-1.87144700	-1.66009900
H	-1.09467800	1.55017500	3.18138200
H	-1.56196700	0.14959900	2.15881700
H	-0.29413100	-0.03035800	3.43792600
H	2.80761100	3.94961800	-0.36066000

H	3.83850800	2.50005300	-0.54264300
H	2.53975500	2.84040500	-1.73783600
C	-2.76625500	1.44611500	-1.03744100
C	-3.95377500	0.95634400	-0.49126300
C	-3.99456200	-0.33822400	0.02802300
C	-2.84877400	-1.13421900	0.00824500
C	-1.65038400	-0.64965100	-0.53222300
C	-1.62216500	0.64875500	-1.05910600
C	-0.41296400	-1.51370300	-0.45421000
C	0.54702200	-1.52709500	-1.67546000
H	-2.73091700	2.44777400	-1.45442000
H	-4.84427000	1.57673200	-0.47990300
H	-4.91754300	-0.73240600	0.44166500
H	-2.88724900	-2.14431100	0.40932500
H	-0.71272900	1.04613500	-1.49998800
H	-0.72132600	-2.54230800	-0.24521000
H	0.48838900	-2.48773100	-2.19235900
H	0.30743200	-0.74575100	-2.39739000

36

***exo*-INT3**

C	0.35926900	-1.51276700	1.18998400
C	-0.80352500	-2.04120200	0.29465200
C	0.70357000	-0.09144700	0.65635800
C	-0.09424600	0.03002000	-0.72828200
C	-0.33175400	-1.47588400	-1.05652900
O	-0.09395900	-2.03899700	-2.07899300

C	-2.10312600	-1.38088900	0.60749600
C	-1.41719400	0.63677700	-0.52172000
C	-2.38967700	-0.08281300	0.27302100
O	-1.58663100	1.77648000	-1.10583600
C	-2.81407700	2.57830200	-1.14146200
O	-3.52804700	0.58233400	0.55741500
C	-4.55033600	-0.10584700	1.29293000
C	2.17538900	0.17148000	0.44587200
C	2.78815700	1.24059700	1.10669400
C	4.14851900	1.49535500	0.93012800
C	4.90431700	0.68913300	0.07993400
C	4.29705300	-0.37500200	-0.59182400
C	2.94099700	-0.63382400	-0.40982300
H	1.21442100	-2.18249600	1.07169200
H	0.08394000	-1.49797400	2.24571800
H	-0.89328200	-3.12769900	0.29316500
H	0.31077100	0.66417000	1.33979400
H	0.45899700	0.55880200	-1.50205400
H	-2.85678500	-1.97719400	1.10948500
H	-2.55639300	3.40427200	-1.80054200
H	-3.04813300	2.92493400	-0.13725700
H	-3.62678500	1.98111500	-1.55042000
H	-5.35126400	0.61978500	1.42496700
H	-4.17170100	-0.42512800	2.27002200
H	-4.91803100	-0.96864300	0.72710300
H	2.20187500	1.87306400	1.76876100
H	4.61425100	2.32342800	1.45521500
H	5.96209700	0.88758900	-0.06108200
H	4.88040300	-1.00410600	-1.25682100

H	2.48470100	-1.46437700	-0.94300800

25
1a

C	-1.61351600	0.52061100	-0.23927700
C	-0.29168000	0.78532100	-0.17488400
C	0.77003100	-0.30319400	-0.03463500
C	0.17536900	-1.69131100	0.05851400
C	-1.13494700	-1.93974700	-0.00484100
C	-2.13091400	-0.84938200	-0.16798200
O	-3.33252500	-1.09716400	-0.22718100
O	0.28075400	1.99782000	-0.20027700
C	-0.57154100	3.12874900	-0.35434900
C	2.71616900	-1.02157700	-1.25456600
O	1.58461700	-0.15770200	-1.16812400
O	1.59303900	-0.06499100	1.10263900
C	0.91461900	0.02951000	2.34704800
H	-2.35999000	1.29888800	-0.34141100
H	0.89699400	-2.49076400	0.20060700
H	-1.54122000	-2.94494500	0.06132600
H	0.08642600	3.99785800	-0.35953300
H	-1.12528300	3.07213600	-1.29847100
H	-1.28008800	3.20090600	0.47940300
H	3.33798100	-0.60902500	-2.05150200
H	3.28138000	-1.03176400	-0.31849200
H	2.42720800	-2.04504700	-1.52636200
H	1.69247900	0.10293200	3.10973800

H	0.28153700	0.92502500	2.39205400
H	0.29396500	-0.85378400	2.54601700

16

2a

C	1.35770100	1.33010200	-0.00000700
C	2.26210400	0.26399100	-0.00005400
C	1.78041900	-1.04447800	-0.00007200
C	0.40705500	-1.28181500	-0.00004300
C	-0.51465600	-0.22246200	0.00000600
C	-0.01221600	1.09084600	0.00002300
C	-1.95203800	-0.53184100	0.00003700
C	-2.97084500	0.33594800	0.00009700
H	1.72438200	2.35294600	0.00000700
H	3.33152100	0.45450300	-0.00007600
H	2.47304800	-1.88155100	-0.00011000
H	0.03599600	-2.30394200	-0.00005700
H	-0.69957200	1.93124300	0.00005800
H	-2.18424000	-1.59671100	0.00000700
H	-3.99929100	-0.01105700	0.00011500
H	-2.82699100	1.41282400	0.00013200

21

TMSOTf

Si	-2.00183200	0.19255200	-0.01916600

C	-2.87877500	1.02520200	-1.44481400
C	-1.71184800	1.34186500	1.42681700
C	-2.78380600	-1.42772900	0.48629300
O	-0.45225100	-0.18075500	-0.75475700
S	0.79196400	-0.88201200	-0.05859200
O	1.37138300	-1.85511800	-0.96640500
O	0.48180800	-1.21529500	1.32881700
C	1.93973600	0.58543300	-0.01960400
F	3.07872500	0.22103800	0.56592300
F	1.37705500	1.57559400	0.68174300
F	2.18509200	1.00683700	-1.25772900
H	-2.96293300	0.35184200	-2.30426100
H	-2.34078500	1.92277300	-1.76807200
H	-3.89124700	1.32665100	-1.15056100
H	-1.07959200	0.86395400	2.18075900
H	-1.22672900	2.27086200	1.11099900
H	-2.66797000	1.60155400	1.89713400
H	-2.19277800	-1.91251900	1.26934200
H	-2.85362700	-2.11417500	-0.36462000
H	-3.79733100	-1.26498800	0.87226300

84

3i

C	-3.12944900	2.33793400	0.90957800
C	-3.70298100	2.78576100	2.28293600
H	-4.55048600	3.46003100	2.11168600
H	-2.95365900	3.32775100	2.86390100

C	-4.18848200	1.50329400	2.96386800
H	-4.95798100	1.68887600	3.71790200
H	-3.35906100	0.98780500	3.45480100
C	-4.72662700	0.66510200	1.79869700
H	-5.78049200	0.89335800	1.60622200
H	-4.63548300	-0.40851300	1.98163200
C	-3.67681400	0.37742100	-0.47809200
C	-4.43899500	-0.94342100	-0.72925500
H	-4.74961700	-1.41097600	0.20679500
C	-2.46634100	-2.25536300	-0.47809500
C	-2.49767500	-3.03033100	0.77719100
C	-0.93729400	-2.97980700	0.75369600
C	-1.08900000	-2.04238700	-0.42256700
C	1.02940800	-0.97462600	-1.15013100
C	1.90611800	-1.88116200	-0.54826900
H	1.52820000	-2.72067200	0.02449800
C	3.27997300	-1.66078100	-0.64378600
C	2.90857200	0.33543900	-1.91683000
C	1.53560100	0.14209800	-1.82966500
H	0.85689800	0.86294500	-2.27256300
C	-5.69296500	-0.78629600	-1.63849000
C	-6.36410000	-2.16483900	-1.75710100
H	-6.68582300	-2.53280700	-0.77576500
H	-7.24803300	-2.10123600	-2.40168600
H	-5.67535800	-2.89940200	-2.18267900
C	-6.67710500	0.20223900	-0.99148500
H	-6.24208000	1.20202500	-0.88417800
H	-7.57351600	0.29493200	-1.61375900
H	-6.99772000	-0.14487600	-0.00293400

C	-5.31076400	-0.27518300	-3.03886000
H	-4.67803400	-0.99450000	-3.57033600
H	-6.21692900	-0.13915900	-3.63906600
H	-4.77959000	0.67944000	-2.99389700
N	-3.89541800	1.07933900	0.65249800
N	-3.44326400	-1.84199000	-1.34542400
H	-3.03674100	-1.35341000	-2.14124200
N	-0.36055700	-1.14555500	-1.12461500
H	-0.91420900	-0.38311800	-1.51635200
O	-2.84142900	0.73854200	-1.32455400
O	-3.35775700	-3.39623700	1.55081400
O	-0.02671500	-3.47911100	1.37122400
F	3.77288500	-3.09188500	1.17744500
F	4.30059500	-3.78238200	-0.81091100
F	3.81873500	1.08729200	-3.95959500
F	4.55820100	2.01936700	-2.14565500
C	3.79700400	-0.56587400	-1.32898700
H	4.86470200	-0.40066700	-1.37996200
C	3.45433200	1.48462400	-2.71751100
C	4.20672000	-2.67807800	-0.03506800
F	5.45978900	-2.19915400	0.12407600
F	2.55314300	2.47832900	-2.87217300
C	3.02990200	3.25483400	0.25608900
H	3.77327300	3.91561100	-0.17992400
C	4.84808300	1.78456000	1.10725400
H	5.58823100	2.44817200	0.66899600
C	3.48629900	2.09208500	0.96387600
C	5.24872000	0.63920900	1.79081400
H	6.30604600	0.41072900	1.88647800

C	4.30553800	-0.22556800	2.34362300
H	4.62683300	-1.12647800	2.85655800
C	2.51615500	1.20659400	1.51771200
C	1.70288300	3.51864300	0.10653500
H	1.37575600	4.39765200	-0.44243100
C	0.15396700	0.58413500	1.87848600
C	2.93375100	0.03373700	2.21378800
C	1.12961200	1.48185700	1.35667400
C	0.70343600	2.64516800	0.65440800
C	-0.66954200	2.89602300	0.51036000
C	-1.63236300	2.02119500	1.01844000
C	-1.20448700	0.86659700	1.68468300
H	-1.92884000	0.14403800	2.04532100
C	-3.45612800	3.38894800	-0.15919300
H	-4.54292000	3.46259400	-0.26707600
H	-3.02280900	3.12562300	-1.12379800
H	-3.08612600	4.37163100	0.14944800
H	-0.96925900	3.78952900	-0.02560200
C	1.92919900	-0.85347600	2.72969600
C	0.60334100	-0.58983600	2.57330400
H	-0.13787400	-1.28090300	2.96137600
H	2.24945900	-1.76104400	3.23192900

105

IM1

C	-3.69410400	2.10122500	-2.20797700
C	-4.07985500	3.59805600	-2.19671300

H	-5.08779600	3.69205200	-2.61643500
H	-3.41635200	4.19854500	-2.82194500
C	-4.10722900	4.00579900	-0.72324900
H	-4.69299200	4.91050700	-0.53829100
H	-3.09764000	4.16674000	-0.33575000
C	-4.71935200	2.77855800	-0.05203100
H	-5.81657200	2.81399200	-0.09575700
H	-4.41114400	2.69640800	0.98870900
C	-4.30194600	0.33702800	-0.51829800
C	-4.09011600	0.00723800	0.97926400
H	-4.07769300	0.91317100	1.58038600
C	-1.70863100	0.31824000	1.23661800
C	-1.63200900	1.75768700	1.66302200
C	-0.18209000	1.66893800	1.44623500
C	-0.24758300	0.26478800	1.17521300
C	1.95832700	-0.84988700	0.92122900
C	2.80374600	0.24881400	1.08960800
H	2.40619600	1.23820900	1.25866300
C	4.18097300	0.06659700	1.00154500
C	3.87160400	-2.26576700	0.54474600
C	2.49337400	-2.11493700	0.64959300
H	1.83212400	-2.96016500	0.49620900
C	-5.10279700	-0.96393000	1.62652900
C	-4.73312000	-1.10816100	3.11334000
H	-4.71552700	-0.13577400	3.62145900
H	-5.46550200	-1.74260500	3.62361400
H	-3.74930800	-1.57494900	3.23248500
C	-6.48271800	-0.29306000	1.49697900
H	-6.77864700	-0.19687600	0.44630900

H	-7.24281200	-0.89739800	2.00273200
H	-6.49184700	0.70531800	1.95226800
C	-5.14061000	-2.35418900	0.97286400
H	-4.19114900	-2.88173700	1.07796600
H	-5.90897000	-2.95770000	1.46980700
H	-5.36832900	-2.29344600	-0.09153800
N	-4.23035300	1.65237000	-0.86842100
N	-2.69095500	-0.49727300	0.99111200
H	-2.50298400	-1.40800200	0.50756600
N	0.55373600	-0.77448800	0.98523000
H	0.08924800	-1.70601100	0.96825400
O	-4.31419300	-0.58735300	-1.32494500
O	-2.42187300	2.60245700	2.05843700
O	0.79595300	2.52549400	1.49268300
F	4.50487200	2.41388900	0.91219500
F	5.44549900	1.33523400	2.54412100
F	5.72489200	-3.73341300	0.41124400
F	4.30924100	-3.73846200	-1.24219200
C	4.72994200	-1.18475000	0.73115000
H	5.80109100	-1.30952200	0.63892400
C	4.41695000	-3.59669400	0.10535800
C	5.09164500	1.23792600	1.24075700
F	6.23498600	1.14391000	0.52901900
F	3.75162200	-4.62475900	0.66144400
C	1.51834800	-1.35492400	-2.40484500
H	1.87247200	-2.37996900	-2.46187400
C	3.86686000	-0.52596500	-2.40111700
H	4.22455900	-1.54585000	-2.49754800
C	2.48565700	-0.29282700	-2.33717200

C	4.77262600	0.53035300	-2.31008100
H	5.83885200	0.32830900	-2.34622200
C	4.32668800	1.83930900	-2.14920900
H	5.04091600	2.65324200	-2.06255900
C	2.02097700	1.04853500	-2.18708400
C	0.18171200	-1.09856400	-2.37886200
H	-0.53136700	-1.91316800	-2.42033600
C	0.14250900	2.65045300	-2.03565500
C	2.95361300	2.12252500	-2.08987100
C	0.62189900	1.31499400	-2.15314000
C	-0.31403500	0.24782400	-2.27338400
C	-1.68575700	0.53419300	-2.29650900
C	-2.17075700	1.84905700	-2.19306700
C	-1.24269900	2.88655700	-2.04911000
H	-1.57307000	3.91561000	-1.96499500
C	-4.36499700	1.42116000	-3.41033800
H	-5.45322600	1.49995400	-3.32080600
H	-4.10922400	0.36841700	-3.49677000
H	-4.05259700	1.94167700	-4.32290700
H	-2.37469400	-0.29522000	-2.38832700
C	2.44511300	3.45904100	-1.95504800
C	1.10602900	3.71255700	-1.93695100
H	0.74497200	4.73596300	-1.87406200
H	3.15939200	4.27657600	-1.89517000
S	-1.10042100	-3.69343900	-0.11351300
O	-0.47550600	-3.27316000	1.18366900
O	-2.13516500	-2.71079000	-0.55573500
O	-0.16129900	-4.13921500	-1.14948100
F	-1.24096400	-6.17517700	0.75980500

F	-2.90570400	-4.91714500	1.37535100
C	-2.06899700	-5.20455100	0.36373100
F	-2.78620100	-5.63758800	-0.67616100
Si	0.75310100	4.18033000	2.07450100
C	0.32350100	4.05817300	3.89173300
H	1.02961700	3.41855100	4.43240400
H	0.34430800	5.04978800	4.35962600
C	2.49739400	4.74424800	1.73543900
H	3.23348300	4.13209600	2.26439600
H	2.71517200	4.67220800	0.66526000
H	2.63169000	5.78711300	2.04575200
C	-0.53659800	5.13208500	1.11451400
H	-0.37342000	5.04569900	0.03749700
H	-1.53579600	4.75724200	1.35163800
H	-0.49161400	6.19524200	1.38099600
H	-0.68592800	3.65154100	4.01713000

18

TMSOMe

Si	-0.37394900	-0.00000100	0.00392400
C	-1.72875400	-0.00062600	-1.29295800
C	-0.48406400	1.54014700	1.08642500
C	-0.48373300	-1.53936500	1.08757400
O	1.06110500	-0.00016200	-0.86056100
C	2.33065300	-0.00003300	-0.23618000
H	-1.65763600	-0.88607100	-1.93425500
H	-1.65763300	0.88421300	-1.93509200

H	-2.72168400	-0.00040300	-0.82760700
H	0.31568000	1.57437100	1.83571300
H	-0.41540100	2.45226000	0.48249700
H	-1.43841500	1.56802500	1.62652800
H	0.31584600	-1.57272800	1.83707700
H	-0.41460500	-2.45191700	0.48436100
H	-1.43820200	-1.56717300	1.62747300
H	3.09736600	-0.00015900	-1.01738000
H	2.47824500	0.89148200	0.39037600
H	2.47826700	-0.89132300	0.39069000

112

IM2

C	4.06699700	-3.16874100	-0.90137200
C	4.91718400	-4.32723500	-0.30999600
H	5.92064900	-4.28989900	-0.75105000
H	4.47976500	-5.29913300	-0.55053500
C	4.99877600	-4.04594200	1.19111200
H	5.85814000	-4.52698500	1.66634400
H	4.09755300	-4.39392800	1.70257100
C	5.09469800	-2.51898700	1.25631500
H	6.13908600	-2.19515000	1.18785600
H	4.65530200	-2.12115400	2.17099600
C	3.84086200	-0.81839100	-0.15022900
C	4.09136700	0.28801400	0.90479000
H	4.34420300	-0.14890600	1.87017700
C	1.87821000	0.48301300	1.87704400

C	1.85445500	-0.71031100	2.78306600
C	0.35833000	-0.46668700	2.99087200
C	0.49222200	0.72445800	2.10003200
C	-1.71337300	1.62897500	1.44841800
C	-2.50151600	0.61485600	2.00581400
H	-2.05098000	-0.17597600	2.59597500
C	-3.87133000	0.61021900	1.76389300
C	-3.69059300	2.62728900	0.48097000
C	-2.31503900	2.65112100	0.69294900
H	-1.70503100	3.45204700	0.29261000
C	5.24444200	1.26367400	0.51177900
C	5.24132800	2.44609800	1.49799500
H	5.24736600	2.10120900	2.53905900
H	6.13585300	3.05870400	1.34054400
H	4.37169100	3.09282400	1.35402600
C	6.57383700	0.49995700	0.64534800
H	6.61789600	-0.34988300	-0.04430600
H	7.40790900	1.16594600	0.40104400
H	6.72891500	0.12907900	1.66525200
C	5.12811900	1.79455900	-0.92827200
H	4.19748300	2.33718900	-1.10119900
H	5.95848700	2.48553000	-1.11507200
H	5.18594800	0.98537100	-1.66050500
N	4.33974600	-2.06297100	0.07107400
N	2.81442400	0.99803100	1.10395200
H	2.56932300	1.74674000	0.44103600
N	-0.31565500	1.62954400	1.51741300
H	0.12507700	2.47910000	1.12347100
O	3.19218200	-0.53697800	-1.15847500

O	2.66289100	-1.54408500	3.13967200
O	-0.52917000	-1.04862100	3.58386500
F	-4.06272300	-1.70193600	2.21714700
F	-4.91110500	-0.33100500	3.67429000
F	-5.26189400	4.38723700	0.41042200
F	-5.02465100	3.19296800	-1.38521200
C	-4.48213000	1.60305600	0.99763300
H	-5.54691300	1.57549100	0.80286100
C	-4.35687700	3.70840200	-0.32579400
C	-4.69081800	-0.50393200	2.35790600
F	-5.89567300	-0.61978700	1.75551000
F	-3.47143800	4.60587200	-0.81084000
C	-1.51835300	-4.91982500	-3.15936900
H	-2.04547100	-5.20028900	-4.06773900
C	-3.65046700	-5.07866100	-1.87679300
H	-4.17775400	-5.36363000	-2.78362000
C	-2.26769000	-4.83717000	-1.93607800
C	-4.34467900	-4.94358100	-0.67509800
H	-5.41514400	-5.12688100	-0.64869400
C	-3.67996900	-4.56956200	0.49291800
H	-4.23134000	-4.44582000	1.42066400
C	-1.57772600	-4.47634700	-0.73998700
C	-0.18474500	-4.64095900	-3.19562800
H	0.36091300	-4.69235200	-4.13441700
C	0.51198800	-3.84959200	0.41011600
C	-2.29449600	-4.34047400	0.48798000
C	-0.17824900	-4.21654100	-0.77682500
C	0.53358300	-4.26748800	-2.00962400
C	1.89735800	-3.92852000	-2.03276000

C	2.57510600	-3.53462100	-0.87662500
C	1.87143000	-3.51400600	0.33265000
H	2.35079100	-3.15609400	1.23439900
C	4.58073600	-2.81690600	-2.30381700
H	5.60025700	-2.42708800	-2.22189600
H	3.95661900	-2.06405200	-2.78291500
H	4.61836700	-3.71624800	-2.92754900
H	2.41102500	-3.95301500	-2.98765800
C	-1.57097000	-3.95211100	1.66900300
C	-0.22741300	-3.73403000	1.63494000
H	0.29691300	-3.41539300	2.53005900
H	-2.12370500	-3.81347700	2.59350100
S	1.21479200	4.17323800	-0.62456300
O	0.48875800	4.13081300	0.67874600
O	2.10361000	2.99153300	-0.81464500
O	0.38740000	4.52160900	-1.79391100
F	1.75356800	6.72403500	-0.21184500
F	3.20269400	5.34018500	0.64044400
C	2.40810100	5.57838600	-0.41540500
F	3.17081400	5.69669500	-1.50676900
C	-1.60579400	-0.85941200	-0.76232300
C	-2.42262300	-0.05023100	-1.49491500
C	-1.87472300	1.16214300	-2.10392000
C	-0.48681300	1.43192600	-2.06067300
C	0.34074600	0.59286700	-1.36711400
C	-0.20131500	-0.53172500	-0.60560000
O	0.52811200	-1.13090200	0.20738200
O	-3.73606300	-0.21923600	-1.70993100
C	-4.30164800	-1.46637800	-1.28603800

O	-2.75362800	1.92167000	-2.71057200
C	-2.33933800	3.17595800	-3.30563000
H	-1.97482400	-1.72251400	-0.22550200
H	-0.08707800	2.31396800	-2.54330100
H	1.41327300	0.73615900	-1.33986800
H	-5.34494200	-1.43403200	-1.59839500
H	-3.78521000	-2.30130100	-1.76778600
H	-4.24168500	-1.58071200	-0.20293500
H	-3.27130500	3.68293200	-3.54408000
H	-1.74581900	3.76238700	-2.60244600
H	-1.76406400	2.97076500	-4.21235400

128

IM3

C	-4.75482100	-2.52407100	0.65628200
C	-5.84033100	-3.43843500	0.02320200
H	-6.79056700	-3.27961900	0.54760800
H	-5.57274500	-4.49311700	0.12255500
C	-5.96158400	-2.96765500	-1.42640000
H	-6.91490200	-3.24368300	-1.88548400
H	-5.15927600	-3.38555500	-2.04065400
C	-5.80211700	-1.45052900	-1.30908200
H	-6.76483400	-0.97711900	-1.08541700
H	-5.38288800	-1.01339300	-2.21420600
C	-4.10268200	-0.19678100	0.09802300
C	-4.26244700	1.06814200	-0.77755500
H	-4.70885200	0.81419200	-1.73827900

C	-2.16914200	1.00956700	-2.00239500
C	-2.44293300	-0.08432600	-2.97870500
C	-0.97144200	-0.03802100	-3.37688400
C	-0.80787500	1.07277000	-2.39590400
C	1.57792200	1.60271700	-2.02575600
C	2.11153600	0.51280500	-2.72304200
H	1.46392100	-0.16690900	-3.26688900
C	3.48637600	0.29249600	-2.69417300
C	3.80841700	2.24315100	-1.34383600
C	2.43859200	2.48452700	-1.34631100
H	2.02532800	3.34604900	-0.83521500
C	-5.17004500	2.15183300	-0.11336700
C	-5.10400200	3.43211700	-0.96580000
H	-5.32274500	3.22233400	-2.01974200
H	-5.84534900	4.15342800	-0.60425900
H	-4.12358700	3.91060800	-0.90721900
C	-6.61717400	1.62825600	-0.11066300
H	-6.71299200	0.71383200	0.48446600
H	-7.28025900	2.38012600	0.32990800
H	-6.97453500	1.42125100	-1.12603900
C	-4.76359100	2.47561400	1.33564900
H	-3.74118400	2.84990900	1.41031000
H	-5.43224600	3.25327700	1.72302300
H	-4.84973800	1.59838300	1.98197800
N	-4.87314200	-1.28344500	-0.17191900
N	-2.91952400	1.58954800	-1.07639100
H	-2.47863300	2.21432800	-0.39205200
N	0.20243500	1.81481100	-1.88664100
H	-0.04520300	2.67870700	-1.38131600

O	-3.28750000	-0.17759900	1.02376500
O	-3.40861000	-0.76599100	-3.27454200
O	-0.26650800	-0.69267500	-4.12411900
F	3.31883700	-2.03192100	-3.09242500
F	3.92015000	-0.78008800	-4.76301400
F	5.60516200	3.77040200	-1.47292700
F	5.49747000	2.50739700	0.28757900
C	4.35026900	1.13915700	-2.00315100
H	5.41480900	0.94451200	-1.97428500
C	4.74470400	3.17200400	-0.62153800
C	4.01653800	-0.90830300	-3.42814300
F	5.31196500	-1.15670300	-3.13246300
F	4.08902300	4.14953000	0.04355100
C	0.52429000	-5.44447900	2.34069800
H	1.02980800	-5.92418100	3.17498600
C	2.61922200	-5.58186400	0.99422600
H	3.13135400	-6.04498400	1.83397900
C	1.26482800	-5.23171800	1.12643400
C	3.29929100	-5.34692200	-0.20041300
H	4.34645900	-5.62396900	-0.28617700
C	2.64932600	-4.76444500	-1.28895900
H	3.18982600	-4.57393500	-2.21157900
C	0.59523300	-4.62954300	0.01853900
C	-0.77216900	-5.04220800	2.46077300
H	-1.30722300	-5.18775700	3.39563500
C	-1.42219500	-3.58503700	-0.94360100
C	1.29489300	-4.40271400	-1.20668300
C	-0.76277800	-4.21986100	0.14510400
C	-1.45388000	-4.38418100	1.38018200

C	-2.74332400	-3.84675600	1.52205300
C	-3.36397000	-3.15008200	0.48052100
C	-2.70620900	-3.06033900	-0.75019900
H	-3.15001000	-2.50166800	-1.56438300
C	-5.10880400	-2.23517800	2.12169000
H	-6.04540000	-1.66980300	2.15573400
H	-4.33272300	-1.65298200	2.61747900
H	-5.26720400	-3.17059400	2.66795600
H	-3.23496700	-3.95105900	2.48340800
C	0.58992900	-3.79128600	-2.30176800
C	-0.71019900	-3.41058600	-2.17918000
H	-1.21214700	-2.91333000	-3.00261500
H	1.12925200	-3.60240800	-3.22494900
S	-0.63308300	4.35839900	0.66197400
O	-0.05001100	4.33927300	-0.70945700
O	-1.62807500	3.26813700	0.87466600
O	0.33835500	4.52745400	1.75977800
F	-0.90595400	6.97987100	0.51816000
F	-2.57117700	5.84967600	-0.31142000
C	-1.66973200	5.89700900	0.68263800
F	-2.32213900	5.99401200	1.84610900
C	1.57731700	-0.98138600	0.13593500
C	2.56876500	-0.30389600	0.77515800
C	2.25722600	0.94177400	1.48784100
C	0.92693600	1.38557200	1.62453400
C	-0.08458400	0.66714400	1.04095000
C	0.21077000	-0.47927800	0.15995000
O	-0.68052500	-0.94796200	-0.55274600
O	3.86607800	-0.63260400	0.82222200

C	4.23264600	-1.90302800	0.27640700
O	3.30100900	1.57484900	1.95689900
C	3.12794700	2.83847200	2.64016700
H	1.76686200	-1.86305600	-0.46106500
H	0.70707100	2.29852200	2.16197700
H	-1.11708300	0.98486500	1.09985900
H	5.30082600	-2.00365500	0.46723900
H	3.68016400	-2.70637000	0.77188900
H	4.03931900	-1.94175700	-0.79664300
H	4.13874400	3.21893800	2.76683200
H	2.52496900	3.52408700	2.04336000
H	2.65415200	2.65575700	3.60709500
C	2.82216800	0.12991900	4.96144800
C	3.92839500	-0.69709800	4.75834800
C	3.78959600	-1.86948600	4.00915700
C	2.55833900	-2.20274300	3.45943200
C	1.43784500	-1.36492400	3.62902400
C	1.59114300	-0.19208500	4.39644000
C	0.19881400	-1.71380600	2.95097400
C	-0.94557700	-0.99634800	2.94343200
H	2.92093200	1.03075200	5.56060400
H	4.89094300	-0.43454600	5.18676800
H	4.64576100	-2.51911400	3.85270700
H	2.44712600	-3.10999900	2.87174600
H	0.74026100	0.46126800	4.55744800
H	0.23774500	-2.61707700	2.35249200
H	-1.79620000	-1.29626600	2.34336500
H	-1.07896700	-0.10456500	3.54881700

128

A1(TS34-major)

C	-4.19948300	-3.22081200	0.75081200
C	-5.08901100	-4.34275900	0.14202800
H	-6.02731300	-4.40117500	0.70703200
H	-4.59609900	-5.31564300	0.20746500
C	-5.37340400	-3.88952500	-1.29064000
H	-6.26208700	-4.36157500	-1.71872100
H	-4.52574500	-4.10713600	-1.94683500
C	-5.54128700	-2.37779000	-1.13997600
H	-6.56194400	-2.13016800	-0.82516500
H	-5.29822600	-1.84650400	-2.05805400
C	-4.00882100	-0.82492000	0.15802100
C	-4.46781700	0.39107300	-0.67608900
H	-4.93353700	0.05788300	-1.60260900
C	-2.49260300	0.68787000	-2.03820000
C	-2.64809600	-0.44377700	-2.98595500
C	-1.23509000	-0.17272200	-3.47493900
C	-1.18558700	0.95873600	-2.50424900
C	1.11586300	1.79343100	-2.21266100
C	1.75206900	0.76600900	-2.92329300
H	1.17257100	0.03278300	-3.47315000
C	3.14241300	0.69820500	-2.92894700
C	3.27999000	2.65419300	-1.55613900
C	1.89331800	2.75549000	-1.54194600
H	1.40278400	3.56170100	-1.00910100
C	-5.51247900	1.27728700	0.07285100

C	-5.79609800	2.51965100	-0.78979800
H	-6.09326000	2.23827000	-1.80722800
H	-6.61371400	3.09928900	-0.34645400
H	-4.92289400	3.17177400	-0.85746500
C	-6.81322900	0.46896200	0.22532900
H	-6.66193400	-0.43001100	0.83272700
H	-7.57196600	1.08111200	0.72427900
H	-7.21622900	0.16568700	-0.74777800
C	-5.04047300	1.71716300	1.47034400
H	-4.12539400	2.31122500	1.43727500
H	-5.82205600	2.33364400	1.92979900
H	-4.85673800	0.85748700	2.12021300
N	-4.57908600	-2.03504100	-0.07378800
N	-3.27838700	1.15712800	-1.06724500
H	-2.86765200	1.78559500	-0.37009400
N	-0.27322100	1.87167400	-2.08405300
H	-0.60706200	2.66407100	-1.52269000
O	-3.13815900	-0.66500900	1.02087900
O	-3.50705200	-1.28208800	-3.21708000
O	-0.48183900	-0.72715700	-4.25818300
F	3.96618700	-1.51679100	-2.76331500
F	3.08183700	-0.92891300	-4.65578900
F	4.77360300	4.47826700	-1.72414600
F	5.07518100	3.08766800	-0.08638300
C	3.92570700	1.62334000	-2.24175100
H	5.00678600	1.55452900	-2.24738000
C	4.12565300	3.67646000	-0.84932400
C	3.80042600	-0.45889900	-3.62377000
F	5.03372300	-0.15207500	-4.07643700

F	3.39751700	4.47921400	-0.04001100
C	1.51596300	-5.13394800	2.42066600
H	2.09896100	-5.51408300	3.25592600
C	3.59710100	-4.93253500	1.06377100
H	4.18279900	-5.29392300	1.90564400
C	2.20352000	-4.81483400	1.19852400
C	4.22178000	-4.60505700	-0.13920100
H	5.29983900	-4.70582200	-0.23022500
C	3.47738100	-4.16223200	-1.23261900
H	3.97407300	-3.90603700	-2.16245300
C	1.43687200	-4.34617200	0.08793100
C	0.17064900	-4.95537700	2.54413900
H	-0.33011000	-5.18399500	3.48155500
C	-0.73320000	-3.65665600	-0.87068200
C	2.08211000	-4.02602200	-1.14593800
C	0.02995400	-4.16561200	0.21885000
C	-0.61638500	-4.43592400	1.45964700
C	-1.97795800	-4.13123100	1.60355900
C	-2.71606500	-3.56304000	0.56129800
C	-2.08852400	-3.35838300	-0.67331400
H	-2.63333000	-2.89723700	-1.48901400
C	-4.58176400	-2.98637200	2.21982400
H	-5.61218500	-2.61998400	2.26293700
H	-3.92924000	-2.24952800	2.68869300
H	-4.54113700	-3.92236300	2.78587700
H	-2.44135600	-4.31854100	2.56608000
C	1.28008200	-3.55430000	-2.24279400
C	-0.06364600	-3.38433300	-2.11416900
H	-0.64100100	-2.97748200	-2.93820600

H	1.77693100	-3.29042100	-3.17172300
S	-1.31845300	4.13418700	0.70513800
O	-0.74601200	4.29422400	-0.65848900
O	-2.00456200	2.82245100	0.89283600
O	-0.43471600	4.52192400	1.82140100
F	-2.25486100	6.60563000	0.70113800
F	-3.53021500	5.15398500	-0.30017700
C	-2.71694100	5.35395400	0.75025000
F	-3.42866700	5.19448700	1.87338700
C	1.99080300	-0.57205000	0.16959300
C	2.77955500	0.29129500	0.86971700
C	2.18183100	1.43118600	1.58567500
C	0.81059500	1.52681300	1.73340900
C	-0.03282300	0.53156300	1.17807800
C	0.54665300	-0.36653200	0.12491900
O	-0.18561000	-0.90777200	-0.69076600
O	4.10823100	0.23386100	0.99010800
C	4.77865200	-0.87936500	0.39106400
O	3.06605300	2.31797300	2.00988100
C	2.58830400	3.50219700	2.67584400
H	2.39819200	-1.35985400	-0.44957000
H	0.36283700	2.37766500	2.23090400
H	-1.07702800	0.79117500	1.03385000
H	5.82887000	-0.75988300	0.65646300
H	4.39137200	-1.82333100	0.78817600
H	4.65796400	-0.86739700	-0.69314400
H	3.47438300	4.11662600	2.82267700
H	1.84965600	4.02499700	2.06584700
H	2.14633700	3.22713300	3.63873800

C	2.88919200	0.57932500	4.94522100
C	4.09334600	-0.11532900	4.84307100
C	4.16399400	-1.30918700	4.10795200
C	3.04002900	-1.79093100	3.46328200
C	1.81210500	-1.08421800	3.52863700
C	1.75303600	0.10444300	4.29546800
C	0.71925100	-1.53008800	2.72692000
C	-0.48207700	-0.83876800	2.54062400
H	2.83554000	1.49183600	5.53057200
H	4.98020700	0.26442500	5.34132800
H	5.10441300	-1.84678600	4.03676100
H	3.08754600	-2.69745800	2.86737500
H	0.81499200	0.63603300	4.39858900
H	0.88416200	-2.42384200	2.13778400
H	-1.28327600	-1.31756000	1.98572300
H	-0.82004400	-0.13469600	3.29555100

128

IM4

C	-3.99692600	-3.42009200	0.75749500
C	-4.82264500	-4.58878000	0.14602900
H	-5.74849500	-4.71138100	0.72115100
H	-4.27005200	-5.53016900	0.19422400
C	-5.15174100	-4.13714700	-1.27752500
H	-6.01324500	-4.65963300	-1.70257300
H	-4.29911200	-4.29123500	-1.94545300
C	-5.41240300	-2.64116100	-1.10467200

H	-6.44096400	-2.46217100	-0.76900900
H	-5.21921700	-2.08492400	-2.01972200
C	-3.95147700	-1.01316900	0.18794600
C	-4.49724200	0.18142000	-0.62447000
H	-4.96360300	-0.17104700	-1.54341300
C	-2.58228100	0.61117000	-2.03599100
C	-2.69650900	-0.51463200	-2.99546100
C	-1.31083700	-0.16140000	-3.50817500
C	-1.30347500	0.96025400	-2.52686800
C	0.96092700	1.88835700	-2.23406600
C	1.63867000	0.88722000	-2.94548300
H	1.08873100	0.13453900	-3.49874600
C	3.03024200	0.87474400	-2.95005800
C	3.08959800	2.83056400	-1.57089400
C	1.69982000	2.87627300	-1.55730900
H	1.17784900	3.65851900	-1.01853700
C	-5.57560100	0.99733900	0.15560900
C	-5.95984700	2.22116000	-0.69425200
H	-6.27015100	1.92295600	-1.70296500
H	-6.79723500	2.74992200	-0.22490800
H	-5.12950200	2.92401800	-0.78655300
C	-6.81857500	0.10833700	0.33868400
H	-6.59547900	-0.78166600	0.93717800
H	-7.60083800	0.66997200	0.86030200
H	-7.22740900	-0.21640400	-0.62504400
C	-5.09430400	1.46234700	1.54160100
H	-4.21861500	2.11146000	1.48642100
H	-5.89947400	2.02825200	2.02483500
H	-4.83925100	0.61411700	2.18233500

N	-4.45432500	-2.25277600	-0.05091800
N	-3.36514200	1.01975300	-1.03485300
H	-2.96171800	1.64731100	-0.33246800
N	-0.43051800	1.91533300	-2.11434700
H	-0.79292400	2.67768500	-1.53031800
O	-3.07808100	-0.81576800	1.03953200
O	-3.51066900	-1.39995100	-3.21611500
O	-0.53932800	-0.67201600	-4.30448500
F	4.02626000	-1.26937300	-2.77589100
F	3.01449400	-0.79709900	-4.63697600
F	4.48971700	4.72590000	-1.74441900
F	4.88348400	3.33872000	-0.12357300
C	3.77610300	1.82939200	-2.26027500
H	4.85926000	1.80593000	-2.26825000
C	3.89398500	3.88701500	-0.86582700
C	3.74023700	-0.24740400	-3.65004200
F	4.92556200	0.13824400	-4.16603700
F	3.14068400	4.64753700	-0.04109400
C	1.81573400	-5.09184300	2.34758700
H	2.42089700	-5.46660800	3.16945700
C	3.87448200	-4.78172300	0.97710800
H	4.48140700	-5.14152700	1.80457400
C	2.47857100	-4.72123200	1.12634000
C	4.47481800	-4.40113400	-0.22262400
H	5.55500100	-4.45793500	-0.32528200
C	3.70373200	-3.96053000	-1.29830000
H	4.18145200	-3.66155500	-2.22517000
C	1.68381300	-4.25676800	0.03351200
C	0.46548400	-4.96884500	2.48641000

H	-0.01729700	-5.23855100	3.42236500
C	-0.52145200	-3.63231500	-0.89123400
C	2.30496400	-3.88091800	-1.19705400
C	0.27173500	-4.13567000	0.17966800
C	-0.35228800	-4.46209900	1.41873100
C	-1.72567600	-4.22689500	1.57694200
C	-2.49811600	-3.67328700	0.55189900
C	-1.88853600	-3.40527100	-0.67982800
H	-2.46200000	-2.95402400	-1.48158000
C	-4.37682100	-3.22163000	2.23274000
H	-5.42610700	-2.91566900	2.28909400
H	-3.76271000	-2.45167100	2.70047300
H	-4.27704100	-4.15824700	2.79042100
H	-2.17366000	-4.45980200	2.53678400
C	1.47541500	-3.41186800	-2.27434200
C	0.12707600	-3.29722000	-2.13068400
H	-0.47221500	-2.89226900	-2.93990600
H	1.95259600	-3.10475800	-3.19999400
S	-1.51977000	4.05549100	0.75153800
O	-0.97674100	4.28667100	-0.61369800
O	-2.10557400	2.69314000	0.91632500
O	-0.65777500	4.48385400	1.86906100
F	-2.63511900	6.45153200	0.81433600
F	-3.80912000	4.93548300	-0.21483800
C	-3.00347300	5.16825100	0.83510200
F	-3.69386100	4.93067100	1.95839700
C	1.99996400	-0.46775700	0.18106000
C	2.75091700	0.43897000	0.88081500
C	2.10670200	1.53916700	1.60863000

C	0.75288300	1.52050700	1.80694600
C	-0.06646200	0.42273200	1.29151700
C	0.54788800	-0.30589800	0.11449000
O	-0.14778300	-0.81148000	-0.74804800
O	4.07765100	0.43074400	0.99513300
C	4.80211900	-0.62991600	0.36038100
O	2.95612800	2.48838100	2.00603000
C	2.41270000	3.62733300	2.68953800
H	2.44871100	-1.22105500	-0.45281100
H	0.25145300	2.33860800	2.30972600
H	-1.08783300	0.73868200	1.08500200
H	5.84522900	-0.46465700	0.62865200
H	4.46568700	-1.60424500	0.72818600
H	4.67692400	-0.58836700	-0.72213200
H	3.25518800	4.30265800	2.82933000
H	1.62826200	4.10633700	2.10012200
H	2.00485200	3.32031000	3.65971100
C	2.94825500	0.58711900	4.94953700
C	4.17267800	-0.07627200	4.89155000
C	4.31385200	-1.25965200	4.14448200
C	3.23864800	-1.76054400	3.44015000
C	1.98631500	-1.08391200	3.45811800
C	1.85465200	0.08942700	4.24780300
C	0.96011700	-1.51633300	2.59074100
C	-0.28854400	-0.80837800	2.37856600
H	2.84494700	1.48822800	5.54515900
H	5.02481900	0.31846000	5.43674400
H	5.27209400	-1.76845000	4.11315400
H	3.33804000	-2.65215400	2.82852400

H	0.89394900	0.58039900	4.33063900
H	1.14032400	-2.41708100	2.01811700
H	-1.06007900	-1.42211300	1.91474000
H	-0.68395200	-0.32058000	3.27085600

128

TS45

C	-3.63241800	-3.75542100	0.74770700
C	-4.35619300	-4.98679700	0.13022400
H	-5.25772600	-5.20206700	0.71665400
H	-3.71859500	-5.87378400	0.15597800
C	-4.74743800	-4.54624800	-1.28083500
H	-5.56118800	-5.14168900	-1.70398200
H	-3.89344400	-4.60732400	-1.96205100
C	-5.14692400	-3.08507700	-1.07810500
H	-6.18228400	-3.01099700	-0.72420400
H	-5.02276300	-2.49794800	-1.98586600
C	-3.82692300	-1.34627300	0.22339100
C	-4.50293500	-0.19668800	-0.55606900
H	-4.95378200	-0.57865900	-1.47087800
C	-2.67846100	0.44688400	-2.00715900
C	-2.71027000	-0.66173500	-2.99309900
C	-1.37149500	-0.17251800	-3.51982000
C	-1.44660200	0.92143800	-2.51246300
C	0.73901200	2.01800500	-2.20409100
C	1.49306600	1.08271500	-2.92944400
H	1.00296300	0.29867500	-3.49490600
C	2.88084000	1.18646100	-2.94292200
C	2.78812200	3.12194800	-1.53645700
C	1.39935900	3.05291100	-1.51565800
H	0.81795100	3.78410500	-0.96607400
C	-5.63753500	0.49755500	0.26123100
C	-6.16210600	1.68756800	-0.56107000

H	-6.46797000	1.37230600	-1.56598600
H	-7.03475600	2.12588000	-0.06360100
H	-5.40724200	2.46957500	-0.66386100
C	-6.78109900	-0.51314000	0.45986000
H	-6.45556000	-1.38511100	1.03754300
H	-7.60133500	-0.03958200	1.00973000
H	-7.18076100	-0.86292400	-0.49892400
C	-5.16999400	0.99115800	1.64201300
H	-4.36580000	1.72604100	1.57485200
H	-6.01500000	1.46734700	2.15312400
H	-4.81475300	0.16508400	2.26365400
N	-4.21314400	-2.62419500	-0.03134600
N	-3.46764900	0.75423900	-0.97595300
H	-3.10702900	1.40571800	-0.27179400
N	-0.64962500	1.93572700	-2.08531300
H	-1.06873300	2.65582000	-1.48594500
O	-2.95969200	-1.08313200	1.06357500
O	-3.44339700	-1.61420700	-3.21768800
O	-0.56744600	-0.59720100	-4.33491500
F	4.12100500	-0.83127000	-2.82055300
F	2.97981000	-0.48039700	-4.63323500
F	4.03878200	5.11836500	-1.70247300
F	4.53815800	3.76002700	-0.08658900
C	3.55006100	2.19074300	-2.24424600
H	4.63124900	2.26019300	-2.26297300
C	3.50872500	4.23416300	-0.82589600
C	3.68122100	0.14998000	-3.67684300
F	4.79052000	0.66735000	-4.24346100
F	2.70012500	4.93203100	0.00048600

C	2.32864200	-4.99664300	2.17506400
H	2.97821600	-5.35232700	2.97096800
C	4.32750800	-4.48310800	0.77793500
H	4.97732900	-4.82850000	1.57848200
C	2.93468700	-4.53448900	0.95559500
C	4.87187600	-4.00738700	-0.41453300
H	5.95078700	-3.97693300	-0.53884200
C	4.04708700	-3.58162100	-1.45559200
C	2.08387700	-4.08854500	-0.10207500
C	0.97680900	-4.97914500	2.34521100
H	0.53652300	-5.31621100	3.28023000
C	-0.18253300	-3.60255100	-0.96104200
C	2.64859300	-3.61448500	-1.32597700
C	0.66985800	-4.08202300	0.07472700
C	0.09963200	-4.50099900	1.31206500
C	-1.28490000	-4.38545000	1.50189200
C	-2.12113300	-3.86474900	0.50997000
C	-1.55980100	-3.50025700	-0.71971500
H	-2.18542200	-3.07284600	-1.49503500
C	-3.99905300	-3.61837400	2.23336400
H	-5.07055300	-3.41097500	2.31476000
H	-3.44867200	-2.80372100	2.70457700
H	-3.80334900	-4.55153700	2.77100100
H	-1.69342100	-4.68928200	2.45954800
C	1.76261600	-3.16547000	-2.36663300
C	0.41242000	-3.16448200	-2.19529500
H	-0.23298600	-2.77846400	-2.97788900
H	2.19440000	-2.78637200	-3.28757000
S	-1.89513300	3.94176400	0.80316000

O	-1.38117100	4.24011900	-0.56028000
O	-2.33446900	2.52408900	0.95540000
O	-1.08099800	4.44612300	1.92428800
F	-3.25344500	6.20807700	0.89561200
F	-4.26247500	4.59293500	-0.15743200
C	-3.48651500	4.89341500	0.89789700
F	-4.15018000	4.56979400	2.01591500
H	4.48093600	-3.20820500	-2.37682900
C	1.94194100	-0.35672300	0.30124300
C	2.62637200	0.66851000	0.92526800
C	1.90120200	1.72088000	1.63182800
C	0.55986500	1.57597700	1.85069700
C	-0.16686200	0.38847400	1.37492400
C	0.47925000	-0.24371500	0.16049000
O	-0.15252400	-0.74013400	-0.74722400
O	3.94431200	0.77553400	0.99951800
C	4.75699900	-0.20463400	0.33413800
O	2.67182000	2.74812000	2.00695100
C	2.04354500	3.82575800	2.71435700
H	2.45135100	-1.08590500	-0.31597500
H	-0.00755600	2.34788600	2.35684900
H	-1.22605300	0.58574200	1.22695700
H	5.78365700	0.07619800	0.56550200
H	4.54572600	-1.20761400	0.71372600
H	4.58526300	-0.17363100	-0.74172300
H	2.82470000	4.57288900	2.84534600
H	1.20684900	4.24026100	2.14798700
H	1.68619200	3.47422600	3.69008500
C	3.12647200	0.52050600	5.03621000

C	4.36820100	-0.11357700	4.98444200
C	4.56823000	-1.21641700	4.14038800
C	3.53593900	-1.66728700	3.33773200
C	2.27102900	-1.02634600	3.36232800
C	2.07850600	0.06536500	4.24308600
C	1.26364000	-1.44250800	2.44250800
C	-0.09152400	-0.87443900	2.39584600
H	2.97325300	1.36393200	5.70179000
H	5.18272700	0.24291600	5.60788400
H	5.53500800	-1.70942700	4.11150300
H	3.68409400	-2.50318400	2.66115000
H	1.10883800	0.54115800	4.31290500
H	1.45765700	-2.34339000	1.87596500
H	-0.81556800	-1.58485300	1.99415500
H	-0.43542900	-0.51436200	3.36674900

128

IM5

C	3.37291200	-3.97194800	-0.62105600
C	3.99020100	-5.22261300	0.06732700
H	4.87890900	-5.53575600	-0.49412600
H	3.28553900	-6.05771600	0.07567000
C	4.39734900	-4.74150600	1.46008500
H	5.15347600	-5.37746900	1.92849100
H	3.53140000	-4.69813000	2.12767300
C	4.91856400	-3.32989300	1.19015500
H	5.96455400	-3.35737900	0.86143500

H	4.82524200	-2.68694600	2.06373200
C	3.78477000	-1.56460400	-0.24757800
C	4.49702800	-0.43294500	0.52463200
H	4.88544600	-0.80816000	1.46962600
C	2.71116600	0.40129800	1.93326900
C	2.72912800	-0.60318400	3.02745800
C	1.41191400	-0.02337400	3.51953000
C	1.49468200	0.95284600	2.40184300
C	-0.66642400	2.09620100	2.05032100
C	-1.43814800	1.26346200	2.87887600
H	-0.96671000	0.49213000	3.47657600
C	-2.80790600	1.48995600	2.98906900
C	-2.67254700	3.31300600	1.44307700
C	-1.30032000	3.12531500	1.32593800
H	-0.70667700	3.77740400	0.69650200
C	5.70138500	0.16839200	-0.26248200
C	6.28987400	1.32115500	0.56854700
H	6.55909900	0.98578600	1.57754700
H	7.19688300	1.70259400	0.08600900
H	5.58452300	2.14901600	0.66150000
C	6.76982500	-0.92810600	-0.42177800
H	6.39465500	-1.77963400	-1.00021500
H	7.63832600	-0.52423200	-0.95281000
H	7.11581600	-1.29669400	0.55076200
C	5.30762200	0.68351900	-1.65767200
H	4.55505000	1.47265400	-1.61477600
H	6.19577600	1.09809400	-2.14862400
H	4.91165100	-0.12002200	-2.28383300
N	4.05235600	-2.85355100	0.09238100

N	3.49528400	0.58287100	0.87172700
H	3.14578500	1.18023500	0.11661200
N	0.70901700	1.93491800	1.88426000
H	1.13404200	2.58907600	1.21895600
O	2.98462700	-1.28232100	-1.14536500
O	3.44100400	-1.54823500	3.33302200
O	0.60760600	-0.33932700	4.38389900
F	-4.40639100	-0.25495900	3.11768600
F	-2.91245300	-0.16269300	4.69657100
F	-4.01427000	5.25190200	1.51195400
F	-4.31226800	3.89589300	-0.15312300
C	-3.44579100	2.50485400	2.27684900
H	-4.51110500	2.67573800	2.38210100
C	-3.37078400	4.40309500	0.67741700
C	-3.64378200	0.60389300	3.86864500
F	-4.51435700	1.31034800	4.61663900
F	-2.52408900	5.14114000	-0.07018800
C	-2.68645700	-5.01372000	-1.79254200
H	-3.37395900	-5.43635000	-2.52062000
C	-4.61814100	-4.28040300	-0.39889700
H	-5.30551300	-4.69773700	-1.13050800
C	-3.23549300	-4.39312700	-0.61698300
C	-5.10739400	-3.64352800	0.74136700
H	-6.18000400	-3.56650200	0.89538700
C	-4.23538900	-3.11457200	1.69217600
C	-2.33568000	-3.84737000	0.34812900
C	-1.34213200	-5.05974800	-2.00771300
H	-0.94604500	-5.51821700	-2.91019500
C	-0.02591500	-3.37696500	1.09092700

C	-2.84370600	-3.20704100	1.52005400
C	-0.92901200	-3.91939200	0.13273200
C	-0.41377200	-4.50254000	-1.06158700
C	0.97021500	-4.49051500	-1.28749100
C	1.86081200	-3.93198100	-0.36500100
C	1.35013400	-3.40134400	0.82429900
H	2.01997700	-2.95024200	1.54708100
C	3.73085000	-3.96238600	-2.11485300
H	4.81401600	-3.84387000	-2.21785800
H	3.23886600	-3.14170100	-2.63629300
H	3.45810100	-4.91285200	-2.58450700
H	1.33938400	-4.92338000	-2.21122600
C	-1.90819900	-2.67340500	2.47377700
C	-0.56400300	-2.76578600	2.27557600
H	0.11873300	-2.35125300	3.00964200
H	-2.29393700	-2.18556200	3.36287000
S	2.13930800	3.77232400	-1.04629800
O	1.43982100	4.15672800	0.20842500
O	2.43172400	2.31001600	-1.11068300
O	1.57930700	4.32543500	-2.29251300
F	3.71769000	5.88556400	-0.92563000
F	4.36143300	4.20590000	0.29972800
C	3.81431000	4.55569700	-0.87711900
F	4.62251700	4.13986700	-1.86095600
H	-4.62400900	-2.62003300	2.57608800
C	-1.63270600	-0.59850000	-1.03158900
C	-2.26639600	0.72046100	-1.18343500
C	-1.55297200	1.76526900	-1.86746700
C	-0.25133100	1.53001000	-2.23491500

C	0.42407700	0.21858800	-2.00751100
C	-0.13872100	-0.31645400	-0.69020900
O	0.39743700	-0.54091300	0.34979400
O	-3.44477800	1.01951400	-0.74295800
C	-4.26341800	0.03710500	-0.05229200
O	-2.25657800	2.89641500	-2.02801600
C	-1.62226400	3.99074100	-2.70607400
H	-2.11848300	-1.22377800	-0.28732200
H	0.34392700	2.33193700	-2.65812400
H	1.50547800	0.32088300	-1.98438800
H	-5.18533000	0.56864400	0.17121500
H	-4.44807100	-0.81003400	-0.71366100
H	-3.77700500	-0.27391100	0.86896300
H	-2.36775100	4.78333800	-2.71780900
H	-0.72271800	4.31873800	-2.18149600
H	-1.35968500	3.69846400	-3.73001700
C	-3.84217400	0.19126100	-5.07244100
C	-4.99433100	-0.56617600	-4.86653800
C	-4.98718200	-1.57870400	-3.90582000
C	-3.83739400	-1.82446400	-3.15773900
C	-2.67545000	-1.06296500	-3.34871600
C	-2.69255400	-0.05462700	-4.32010200
C	-1.48544400	-1.32570000	-2.44605400
C	-0.08350200	-0.90595500	-2.96034400
H	-3.83394200	0.97928600	-5.81974100
H	-5.88804800	-0.37231000	-5.45214100
H	-5.87524600	-2.18238300	-3.74058000
H	-3.83628300	-2.61526200	-2.41516700
H	-1.81111100	0.55214300	-4.49461300

H	-1.47297400	-2.38817900	-2.20139400
H	0.61355700	-1.74317700	-2.86579200
H	-0.09072300	-0.58277100	-4.00182000

44

IM6

C	-0.59580100	0.61594000	-1.15086100
C	-0.03091000	0.25616700	0.23048300
C	-0.35470000	1.29625100	1.28310200
C	-0.82897400	2.50650400	0.94058600
C	-1.18095800	2.85194900	-0.49015400
C	-0.21493200	2.08708700	-1.39121100
O	0.59699800	2.51537800	-2.16994800
O	-0.23754400	-0.98031400	0.72856700
C	-0.32024500	-2.12857900	-0.13642400
O	-0.04535700	0.86025100	2.52390000
C	-0.19052900	1.78757200	3.58869700
H	-0.15920800	-0.04057000	-1.90111400
H	-1.02040600	3.26055000	1.69500200
H	-1.14464000	3.92855900	-0.66316100
H	-0.36322500	-2.97781500	0.54529800
H	-1.23407100	-2.09304200	-0.73059200
H	0.55379100	-2.20581700	-0.77983600
H	0.12528400	1.26105700	4.48975500
H	0.44379800	2.66874600	3.43019200
H	-1.23550600	2.10784500	3.69505800
C	-4.01684800	-1.42140900	1.29125900

C	-4.59906600	-2.39194900	0.47399400
C	-4.40232200	-2.33629300	-0.90540000
C	-3.61937100	-1.32150200	-1.45635400
C	-3.01850600	-0.34942100	-0.64594600
C	-3.23999700	-0.40662700	0.73640000
C	-2.16097400	0.71965700	-1.28640300
C	-2.52748800	2.19133700	-0.87386800
H	-4.16671000	-1.45432400	2.36669000
H	-5.20557700	-3.18098800	0.90910200
H	-4.85801100	-3.07906400	-1.55401500
H	-3.46657600	-1.28379800	-2.53250900
H	-2.79175200	0.33739500	1.38543400
H	-2.32064400	0.64010300	-2.36645400
H	-2.99466400	2.71018200	-1.71703700
H	-3.23086800	2.21089600	-0.03898700
S	2.62420200	-0.08360500	-0.82813400
O	1.53332400	0.55089500	0.14249000
O	2.02883500	-0.91933400	-1.87018100
O	3.61008100	0.93319500	-1.14451300
F	3.96621100	-0.56347400	1.39051400
F	2.55121700	-2.12521100	0.85147900
C	3.44308100	-1.24913000	0.37709200
F	4.40949300	-1.91110600	-0.26145600

128

A2

C	-4.39624500	-2.80020600	0.51434800

C	-5.34728900	-3.89971300	-0.03335000
H	-6.33045700	-3.78493800	0.43885700
H	-4.97453800	-4.89921700	0.20367200
C	-5.45356900	-3.62389200	-1.53501700
H	-6.35370800	-4.05370800	-1.98303500
H	-4.58847800	-4.02931500	-2.06719200
C	-5.45185200	-2.09385400	-1.60935200
H	-6.46694600	-1.69819900	-1.49471900
H	-5.02271900	-1.72697700	-2.54217600
C	-3.91120900	-0.54038400	-0.32559800
C	-4.11537100	0.60833400	-1.33214200
H	-4.46103000	0.22189300	-2.29066900
C	-1.92951500	0.60734100	-2.37736300
C	-2.06668200	-0.56111200	-3.28389700
C	-0.57814900	-0.44713900	-3.58645000
C	-0.53947700	0.70767800	-2.64440100
C	1.79762500	1.31399200	-2.12349800
C	2.39565200	0.10882100	-2.52818800
H	1.80448700	-0.68296900	-2.97057700
C	3.77601800	-0.04124000	-2.41226900
C	3.97760400	2.16329400	-1.50523600
C	2.60359600	2.34281000	-1.60396100
H	2.13693900	3.26431000	-1.27941800
C	-5.15976600	1.65629600	-0.83402400
C	-5.20545700	2.81066800	-1.84920400
H	-5.42900100	2.44059600	-2.85734500
H	-5.99160700	3.52049900	-1.56802800
H	-4.26055600	3.35344700	-1.88826700
C	-6.54633200	0.99091300	-0.78461200

H	-6.57334400	0.16126100	-0.07051800
H	-7.29375500	1.72535900	-0.46624500
H	-6.84839100	0.61420600	-1.76872300
C	-4.82339500	2.19581900	0.56754000
H	-3.83622300	2.65672600	0.61763000
H	-5.56184100	2.95706700	0.84396700
H	-4.85270800	1.40201300	1.31904600
N	-4.61324300	-1.69272600	-0.46255500
N	-2.80067800	1.22129000	-1.57493000
H	-2.41513300	1.81097300	-0.82858800
N	0.41662800	1.53311400	-2.15190800
H	0.11911600	2.35819100	-1.61939200
O	-3.10038900	-0.38238700	0.59295300
O	-2.96895000	-1.33144100	-3.58174700
O	0.22233600	-1.09428200	-4.24188900
F	4.77757500	-2.00973600	-1.58641900
F	3.63723900	-2.18765100	-3.43443700
F	6.00829900	3.37724400	-1.53398600
F	5.16529900	2.88952500	0.40603900
C	4.58543400	0.97637300	-1.91343400
H	5.65994600	0.85008000	-1.84755000
C	4.83981400	3.21977200	-0.88114000
C	4.42998400	-1.35309600	-2.73487300
F	5.57394700	-1.19303100	-3.43229300
F	4.23536800	4.42530200	-0.82321200
C	1.06597600	-5.05010300	2.61649200
H	1.58047400	-5.41344600	3.50270100
C	3.17565600	-5.26799700	1.30664100
H	3.69373000	-5.61958600	2.19509000

C	1.80954900	-4.95700700	1.38885600
C	3.86638700	-5.11807600	0.10526700
H	4.92531100	-5.35530300	0.06075000
C	3.21536200	-4.65977900	-1.03941700
H	3.76533800	-4.52771400	-1.96505800
C	1.13505200	-4.48993600	0.22038400
C	-0.24428100	-4.67813600	2.68407900
H	-0.78723300	-4.74434700	3.62388300
C	-0.91623200	-3.65886100	-0.87477200
C	1.84852400	-4.34158000	-1.00803400
C	-0.24285100	-4.13801800	0.28652100
C	-0.94693900	-4.20106600	1.52369300
C	-2.28101500	-3.76718200	1.57862300
C	-2.93769200	-3.27270300	0.44849300
C	-2.24847900	-3.24194100	-0.76903900
H	-2.72030900	-2.83009900	-1.65308700
C	-4.82984000	-2.37636500	1.92503900
H	-5.82053500	-1.91489700	1.86703100
H	-4.13600000	-1.66047800	2.36673800
H	-4.91197900	-3.25143300	2.57822900
H	-2.78964800	-3.79933600	2.53618400
C	1.14172400	-3.85845200	-2.16363400
C	-0.18232300	-3.54698900	-2.10546500
H	-0.69383600	-3.17494800	-2.98703400
H	1.68803800	-3.73062300	-3.09181900
S	-0.83049500	4.08249000	0.30304300
O	0.19208600	4.09168300	-0.77808600
O	-1.51423400	2.76403900	0.43203800
O	-0.43345500	4.69258400	1.58362200

F	-1.69587200	6.47576500	-0.40593600
F	-2.47536800	4.84133500	-1.61206800
C	-2.14108800	5.21731300	-0.36322800
F	-3.24287600	5.17402500	0.39885500
C	-0.80682500	0.11304400	2.56134800
C	0.17868700	0.73606400	1.84816200
C	0.89831200	1.89034300	2.40409400
C	0.72234600	2.24108700	3.73555800
C	-0.11402300	1.45844100	4.55941000
C	-1.13146100	0.54643500	3.90649500
O	-2.10235100	0.15504000	4.54303200
O	0.61773900	0.37807300	0.65045000
C	0.07320500	-0.83174400	0.10312200
O	1.67156600	2.51329600	1.54280200
C	2.38068500	3.69363600	1.96478200
H	-1.44374600	-0.63641400	2.11226800
H	1.23977500	3.09825500	4.14548600
H	-0.44414900	1.88797000	5.50178900
H	0.65255700	-1.04084000	-0.79150300
H	0.19618100	-1.64474900	0.81908700
H	-0.98389700	-0.69970800	-0.11973400
H	2.89785500	4.04833400	1.07984200
H	1.66092200	4.44007600	2.30060300
H	3.10141500	3.42725100	2.74434800
C	4.12116100	0.56590400	2.41395700
C	4.15615600	-0.33546000	1.34806300
C	3.33221400	-1.47156300	1.34268300
C	2.45023100	-1.67701700	2.38578200
C	2.36115400	-0.75110200	3.45873300

C	3.24139600	0.36163900	3.47061900
C	1.31045500	-0.91197400	4.40683300
C	0.98368200	-0.00701600	5.42595000
H	4.77450200	1.43072200	2.40580200
H	4.82661600	-0.16110700	0.51493400
H	3.37493800	-2.16939900	0.51437900
H	1.78629500	-2.53358400	2.38133800
H	3.23690000	1.04865900	4.30892300
H	0.66073600	-1.77164900	4.26114300
H	0.23904800	-0.32232100	6.15064600
H	1.75465000	0.64805300	5.81927200

128

A3

C	5.39406000	0.80051500	0.80719100
C	6.72212900	1.40569400	0.27567400
H	7.56308700	0.92322900	0.78880700
H	6.77601100	2.47886000	0.47464900
C	6.74552800	1.05273000	-1.21241500
H	7.74999400	1.08335600	-1.64349400
H	6.11216500	1.73795000	-1.78360900
C	6.14969000	-0.35728900	-1.24059400
H	6.92441400	-1.11265600	-1.06362100
H	5.64709300	-0.56804100	-2.18438400
C	4.07930700	-1.12666100	0.01540100
C	3.83774500	-2.27564900	-0.98506700
H	4.36811700	-2.09311800	-1.91909000

C	1.91708700	-1.40619000	-2.18823600
C	2.56706900	-0.42904600	-3.10884400
C	1.16927700	0.02290400	-3.53616500
C	0.62811400	-0.98297600	-2.58842000
C	-1.79757500	-0.65823700	-2.40385000
C	-1.81379800	0.73262900	-2.60295100
H	-0.89142800	1.28196600	-2.73476100
C	-3.03573700	1.40219100	-2.65216800
C	-4.21781800	-0.64976100	-2.28943900
C	-3.01569800	-1.34708200	-2.25847900
H	-3.00115800	-2.41408100	-2.07310700
C	4.30675300	-3.65791500	-0.43647800
C	3.92893000	-4.73888500	-1.46380300
H	4.33748500	-4.50475000	-2.45442700
H	4.33540700	-5.70727700	-1.15089900
H	2.84635800	-4.84363400	-1.55637900
C	5.83757000	-3.62894400	-0.28374000
H	6.15853300	-2.86606100	0.43357900
H	6.19070800	-4.59867800	0.08282300
H	6.33356900	-3.43414300	-1.24174300
C	3.67720600	-3.99069000	0.92768800
H	2.58641400	-3.99828000	0.89967400
H	4.00830200	-4.98742300	1.24120000
H	3.97717300	-3.27053900	1.69305200
N	5.18329100	-0.34420000	-0.12484300
N	2.40585800	-2.28433500	-1.31395900
H	1.76205100	-2.64375500	-0.60077100
N	-0.61827600	-1.38646200	-2.23903600
H	-0.73889200	-2.27636300	-1.73560500

O	3.25757100	-0.93844800	0.91398800
O	3.71719600	-0.11044500	-3.36474200
O	0.71142700	0.88106200	-4.27081200
F	-2.82066600	3.46673400	-1.52990100
F	-2.08322300	3.37367000	-3.57315800
F	-6.46312300	-0.61241700	-1.55549300
F	-5.35516700	-2.46839400	-1.29344500
C	-4.24744900	0.73136200	-2.49077300
H	-5.18615600	1.26940800	-2.51300200
C	-5.50968100	-1.40060800	-2.11229100
C	-3.04238400	2.90178000	-2.75246100
F	-4.22435800	3.38118500	-3.18750700
F	-6.00335800	-1.85921100	-3.27745900
C	1.52020500	5.43286400	2.49116700
H	1.26222300	6.10528800	3.30593100
C	-0.29330600	6.42792300	1.09904400
H	-0.56545300	7.08653700	1.91985900
C	0.77548300	5.53384300	1.26463900
C	-1.00447200	6.47089000	-0.09912000
H	-1.83320600	7.16452400	-0.20743700
C	-0.66319900	5.63477200	-1.16070700
H	-1.22546200	5.66915300	-2.08826300
C	1.12359600	4.66566300	0.18636200
C	2.51600600	4.51488700	2.64466200
H	3.05950400	4.44572700	3.58361100
C	2.55110300	2.87722900	-0.74036800
C	0.40112600	4.72697400	-1.04432600
C	2.18468900	3.72915200	0.34070400
C	2.88459300	3.62590700	1.57706600

C	3.90124700	2.66742600	1.71259200
C	4.25363000	1.81741900	0.65996200
C	3.58184900	1.94703600	-0.55989200
H	3.83178500	1.29611300	-1.38817200
C	5.57079200	0.30663800	2.24993400
H	6.28910100	-0.51924400	2.25721800
H	4.62593800	-0.04388300	2.66473300
H	5.97673800	1.10296800	2.88232000
H	4.40828800	2.59296700	2.66886800
C	0.79428000	3.85493700	-2.11822500
C	1.83155100	2.98397700	-1.97940400
H	2.12713100	2.35812000	-2.81357500
H	0.25081000	3.90424800	-3.05544300
S	-0.78977900	-3.81343200	0.49882500
O	-1.56052400	-3.57742200	-0.75789600
O	0.40838700	-2.93046600	0.59884900
O	-1.58858400	-3.92423000	1.72748600
F	-1.04464100	-6.42334700	0.17295600
F	0.61289200	-5.53378800	-0.92127900
C	-0.07646000	-5.50732900	0.23514600
F	0.76006300	-5.82530900	1.23189600
C	-2.79017200	-1.69230600	3.71432800
C	-1.68257100	-0.92644100	3.52615100
C	-1.27299200	-0.50794600	2.17357800
C	-2.06300300	-0.81829200	1.08203600
C	-3.29658100	-1.49543000	1.25396500
C	-3.60685400	-2.13667700	2.58799900
O	-4.55808700	-2.89186300	2.71639100
O	-0.85631200	-0.48390800	4.48297600

C	-1.11946200	-0.88860800	5.82363100
O	-0.11577800	0.11538700	2.13925900
C	0.50195700	0.39411200	0.86218400
H	-3.08406200	-2.05690900	4.68996200
H	-1.72814200	-0.56479200	0.08845300
H	-3.67634600	-2.05306700	0.40396500
H	-0.32576700	-0.44641400	6.42532800
H	-2.09514000	-0.51592800	6.15897100
H	-1.09329400	-1.98004900	5.91192000
H	1.46130400	0.84050900	1.10299800
H	0.65467200	-0.54776400	0.33934200
H	-0.12049900	1.08330700	0.28612000
C	-1.76725800	2.90307100	1.49714900
C	-1.29109700	3.19421700	2.77603500
C	-1.97076700	2.72802600	3.91315300
C	-3.10115400	1.94816300	3.76988700
C	-3.55786800	1.56901900	2.47977900
C	-2.87383000	2.07655500	1.34401400
C	-4.56505800	0.57346000	2.37093600
C	-4.77846700	-0.19267900	1.21755800
H	-1.26836800	3.30646600	0.62381800
H	-0.39565900	3.79360900	2.89073700
H	-1.59746800	2.97085200	4.90283500
H	-3.61987500	1.56036900	4.64220900
H	-3.23997500	1.86326100	0.34779900
H	-5.06512400	0.26947400	3.28731800
H	-5.60644700	-0.89441900	1.21270600
H	-4.54140300	0.23350200	0.24880600

128

A4

C	-5.23837000	-0.23010500	-0.98222800
C	-6.26884000	-0.56434300	-2.10222800
H	-7.25483600	-0.18766000	-1.80505200
H	-6.35403100	-1.64342600	-2.25058900
C	-5.77485300	0.18947900	-3.33900900
H	-6.56711300	0.37193400	-4.07030000
H	-4.96920900	-0.35542700	-3.83976400
C	-5.22066900	1.48501000	-2.74861500
H	-6.02202700	2.21125200	-2.56225100
H	-4.46219100	1.92610700	-3.39089600
C	-3.58144100	1.59394300	-0.81238500
C	-2.93003100	2.87680200	-1.35519200
H	-3.18347100	3.01850600	-2.40454800
C	-0.86412700	1.99556000	-2.25376400
C	-1.35364200	1.32409500	-3.48244800
C	0.05878300	0.79411500	-3.70088600
C	0.45749300	1.53956300	-2.47318300
C	2.79641900	1.03220400	-1.94830100
C	2.81012100	-0.27646200	-2.44163500
H	1.91090900	-0.72399500	-2.84182400
C	4.00044800	-0.99908700	-2.42637100
C	5.15916100	0.87416500	-1.48316400
C	3.98232000	1.61218800	-1.47564700
H	3.96201700	2.61744300	-1.07126900
C	-3.39460400	4.15151800	-0.58891000
C	-2.68031800	5.36280700	-1.21245500

H	-2.91580000	5.44872000	-2.28033900
H	-3.00581400	6.28521100	-0.71880800
H	-1.59727100	5.28658900	-1.10778300
C	-4.91248600	4.31480800	-0.78283100
H	-5.47005500	3.47232700	-0.35873700
H	-5.25440200	5.22536200	-0.27928800
H	-5.17479300	4.40259300	-1.84358000
C	-3.09066200	4.06860200	0.91738000
H	-2.03665100	3.88350200	1.12675000
H	-3.36052300	5.01823300	1.39298700
H	-3.66252300	3.26855300	1.39430000
N	-4.63032700	1.04125200	-1.47286200
N	-1.47401000	2.71099600	-1.30346700
H	-1.01028200	2.76501300	-0.38970100
N	1.60960200	1.76348100	-1.81465000
H	1.64468800	2.52786900	-1.12500100
O	-3.11242500	1.08522100	0.21118100
O	-2.43412800	1.21564200	-4.04872400
O	0.58778400	0.02112700	-4.48184700
F	3.45844000	-3.22074300	-1.81484600
F	3.15914400	-2.66712900	-3.89206600
F	7.51928300	1.01145500	-1.52597600
F	6.54576000	1.13889700	0.41075400
C	5.18335000	-0.44083700	-1.94816100
H	6.10297700	-1.01174800	-1.94145800
C	6.40993300	1.46522300	-0.89474900
C	3.96083200	-2.44241400	-2.82728800
F	5.17690000	-2.94025200	-3.11982200
F	6.42172700	2.81161100	-0.97164200

C	-2.37773900	-5.35747000	1.21196800
H	-2.44055400	-6.14127400	1.96246300
C	-0.18425700	-6.26641900	0.45128000
H	-0.23471700	-7.03512300	1.21793700
C	-1.23472200	-5.34188100	0.33917400
C	0.91302500	-6.20131700	-0.40596100
H	1.71957900	-6.92102900	-0.30130900
C	0.98829500	-5.22296700	-1.39672800
H	1.85516800	-5.16667500	-2.04646900
C	-1.15784500	-4.32613500	-0.66213900
C	-3.35633200	-4.41306500	1.11726400
H	-4.21349500	-4.44137200	1.78605400
C	-2.10912700	-2.32412100	-1.75376900
C	-0.03637400	-4.27550200	-1.54635200
C	-2.18642700	-3.34593900	-0.76264500
C	-3.29308900	-3.36546500	0.13445400
C	-4.26450500	-2.35569500	0.04720700
C	-4.17293400	-1.32992500	-0.89674000
C	-3.10761900	-1.34380900	-1.80423000
H	-3.02162600	-0.55972000	-2.54667800
C	-5.96707900	0.01558400	0.34680700
H	-6.62984800	0.87873600	0.23212500
H	-5.26603700	0.22295600	1.15568400
H	-6.58915300	-0.84336800	0.61804200
H	-5.08788300	-2.37881300	0.75365100
C	0.01023600	-3.23999300	-2.54358800
C	-0.98536600	-2.31649700	-2.65199300
H	-0.93873300	-1.56356600	-3.43068600
H	0.85781000	-3.20923300	-3.22040000

S	1.31911400	3.50099800	1.34800100
O	2.08857800	3.76895600	0.10257600
O	0.14334900	2.61116200	1.11470700
O	2.11741100	3.18453100	2.54519300
F	1.52442800	6.02774000	2.09553600
F	-0.10377600	5.64418500	0.70612900
C	0.57348900	5.15239500	1.75862000
F	-0.28323600	5.02975800	2.78490600
C	1.88147300	0.26567600	2.70362400
C	0.65339100	0.27541900	3.29211700
C	-0.51649200	-0.26920400	2.59233300
C	-0.38999100	-0.81397600	1.32237800
C	0.89218900	-0.94017800	0.72858900
C	2.05757500	-0.18854200	1.33586100
O	3.11866200	-0.08108400	0.73741700
O	0.37036100	0.74476700	4.50739700
C	1.40853600	1.44791000	5.20631000
O	-1.63841400	-0.15004500	3.27507000
C	-2.85510800	-0.67003000	2.70909600
H	2.74386700	0.72353300	3.16495400
H	-1.26730600	-1.12697400	0.77183100
H	0.92481800	-1.03545300	-0.35420400
H	0.94017400	1.81464100	6.11944000
H	2.22744800	0.76349800	5.45921900
H	1.77452700	2.27516000	4.59332600
H	-3.61241800	-0.50643700	3.47573200
H	-3.09736700	-0.13203000	1.79271200
H	-2.74377800	-1.73835100	2.50961400
C	-1.30388500	-3.38896400	4.18766900

C	-0.94670100	-3.07476700	5.49673300
C	0.37560900	-2.71137500	5.80428500
C	1.32477300	-2.65603200	4.80261800
C	0.97499200	-2.93851800	3.45637300
C	-0.36022300	-3.31028900	3.16763000
C	1.93484600	-2.73516600	2.42442300
C	1.62926100	-2.72469300	1.05397500
H	-2.31718700	-3.69538900	3.95054200
H	-1.68916900	-3.12058600	6.28778900
H	0.64709500	-2.46919800	6.82678300
H	2.34460600	-2.35577800	5.02601600
H	-0.64571900	-3.57965000	2.16102200
H	2.94075000	-2.45943300	2.72975800
H	2.45581800	-2.67384200	0.35207700
H	0.77943300	-3.29547000	0.70100000

128

B1(TS34-minor)

C	4.91430600	-2.33281900	-0.14461700
C	5.84561700	-3.16828200	0.78434300
H	6.83905300	-3.23299200	0.32528000
H	5.46892200	-4.18633600	0.90831400
C	5.92895600	-2.38230600	2.09584100
H	6.83185700	-2.60926200	2.66924400
H	5.06484800	-2.58284800	2.73587500
C	5.88973600	-0.92665900	1.63138400
H	6.88332300	-0.58091000	1.31699700

H	5.49881800	-0.27134300	2.40658500
C	4.20498600	0.01863800	-0.02207500
C	4.13407400	1.36843700	0.71582300
H	4.61149200	1.28454000	1.69027400
C	2.08180200	1.15878200	2.02080000
C	2.53338500	0.38084000	3.19951600
C	1.07824800	0.31563200	3.65552200
C	0.72673200	1.07730100	2.42658900
C	-1.72151800	1.21892300	2.06236100
C	-2.12984100	0.36097300	3.09283600
H	-1.40518500	-0.04099500	3.79487300
C	-3.48457900	0.04825800	3.21947000
C	-4.01767800	1.43827700	1.34107800
C	-2.67974700	1.77839800	1.19650700
H	-2.37243600	2.47200300	0.41922600
C	4.84025600	2.52050900	-0.05782200
C	4.91370400	3.73900100	0.87804500
H	5.49433800	3.51364800	1.78122500
H	5.39710400	4.57790100	0.36533200
H	3.91552400	4.06362900	1.18226200
C	6.26580400	2.05815500	-0.40724700
H	6.25738400	1.22407000	-1.11769000
H	6.82017800	2.88135600	-0.86981200
H	6.81951300	1.74541000	0.48653300
C	4.09376100	2.90395700	-1.34825600
H	3.13228000	3.38286300	-1.14581300
H	4.69383100	3.63075500	-1.90724800
H	3.90674100	2.04224200	-1.99114500
N	4.98447100	-0.97281600	0.46992300

N	2.72366300	1.67250700	0.97007300
H	2.16729300	2.09186500	0.22285000
N	-0.38468600	1.52419400	1.80405800
H	-0.20550900	2.05658600	0.94120200
O	3.51041300	-0.16651300	-1.03078800
O	3.60043000	-0.07769900	3.58302900
O	0.48163000	-0.21539600	4.57892800
F	-3.24852000	-0.72427600	5.44414200
F	-5.21828000	-0.89877500	4.53128500
F	-6.16370000	2.35675900	0.97418900
F	-5.41734900	0.94222900	-0.49177500
C	-4.43930300	0.56732400	2.34936400
H	-5.48621700	0.31332500	2.45879900
C	-5.03731600	1.93464200	0.35286300
C	-3.89315000	-0.93161000	4.28331200
F	-3.60449200	-2.21627700	3.90773000
F	-4.58288100	2.95208700	-0.40155000
C	-0.23841200	-5.42657400	-1.94338100
H	-0.65332900	-6.05315400	-2.72864300
C	-2.42993000	-5.46987300	-0.75318700
H	-2.84599400	-6.09638800	-1.53829600
C	-1.08751700	-5.06429200	-0.84121900
C	-3.21985700	-5.08317800	0.32904100
H	-4.25680100	-5.40271100	0.37842600
C	-2.69142800	-4.29884400	1.35447500
H	-3.31350100	-3.99574400	2.19081500
C	-0.54269200	-4.25190000	0.19837000
C	1.05151200	-4.99192000	-2.01964000
H	1.67547300	-5.27179000	-2.86471600

C	1.35892800	-2.99961200	1.15714500
C	-1.35205100	-3.87943900	1.31536800
C	0.80759900	-3.80534300	0.11860100
C	1.62199700	-4.15829400	-0.99618900
C	2.93822900	-3.67220500	-1.06683200
C	3.47133100	-2.85127100	-0.07001200
C	2.67691700	-2.54111400	1.04040800
H	3.07160600	-1.90749400	1.82606300
C	5.47745500	-2.31245900	-1.57234800
H	6.44744000	-1.80576300	-1.56392300
H	4.81204800	-1.78149200	-2.25308300
H	5.64066700	-3.33160000	-1.93812900
H	3.53426600	-3.93791500	-1.93341200
C	-0.76417800	-3.08157200	2.35692700
C	0.53256600	-2.67313500	2.28701300
H	0.95967000	-2.10250200	3.10248100
H	-1.37699300	-2.81292300	3.21024800
S	-0.06254500	4.06329500	-0.90644600
O	-1.53402900	4.07480900	-0.75378500
O	0.53325200	2.73435300	-0.49495600
O	0.48923600	4.61657400	-2.14462500
F	0.04702400	6.39402500	0.34410400
F	0.21132100	4.65764600	1.64951900
C	0.55682800	5.16594500	0.45481900
F	1.89764000	5.25143700	0.40672500
C	-0.71939000	1.82715300	-3.18556600
C	-1.81006500	1.29440100	-2.56907000
C	-1.67810500	0.15091800	-1.64605700
C	-0.43379700	-0.34981200	-1.31919700

C	0.72166400	0.13169400	-1.98730900
C	0.62404300	1.34301100	-2.89561000
O	1.62927800	1.80810600	-3.41407400
O	-3.07089400	1.71104400	-2.68678600
C	-3.30008700	2.98393200	-3.30755000
O	-2.83188000	-0.25079300	-1.14184700
C	-2.83872000	-1.32965500	-0.19705900
H	-0.78750900	2.69380800	-3.82898500
H	-0.32767600	-1.12062700	-0.56529900
H	1.68308600	0.04313900	-1.48030100
H	-4.36116100	3.18068100	-3.16198500
H	-3.06648800	2.93642500	-4.37745600
H	-2.69926800	3.74824100	-2.80839300
H	-3.88671200	-1.48511600	0.04738400
H	-2.28167000	-1.05427600	0.70030300
H	-2.40906100	-2.22540700	-0.64485800
C	-2.79859800	-2.81572100	-3.26506900
C	-3.72979300	-2.20660200	-4.10434000
C	-3.31776800	-1.26558100	-5.06168100
C	-1.98078100	-0.93182800	-5.16487300
C	-1.02083900	-1.50902600	-4.29413900
C	-1.45437300	-2.46286600	-3.34308700
C	0.31485400	-1.01280300	-4.31589000
C	1.27161000	-1.21845500	-3.31633200
H	-3.11554100	-3.55471800	-2.53722400
H	-4.78120000	-2.46554800	-4.02336700
H	-4.04930100	-0.79586900	-5.71119400
H	-1.65352300	-0.18627600	-5.88375000
H	-0.73645800	-2.95194900	-2.69889000

H	0.55062500	-0.28556900	-5.08884100
H	2.27186600	-0.83261700	-3.48373400
H	1.22020400	-2.09016200	-2.67272900

128

B2

C	-4.76061100	-4.44887800	-0.51714300
H	-5.76148400	-4.58431300	-0.09022500
H	-4.21484800	-5.38756200	-0.39510700
C	-4.87387100	-3.98784000	-1.97236000
H	-5.67016900	-4.50061300	-2.51889400
H	-3.93619900	-4.15089200	-2.51148400
C	-5.13981800	-2.48801100	-1.83703300
H	-6.20528900	-2.29207600	-1.66599800
H	-4.79993100	-1.93399600	-2.70999200
C	-3.81561200	-0.90208800	-0.35441600
C	-4.09969900	0.30028300	-1.27253400
H	-4.40757800	-0.03923100	-2.26029000
C	-1.93282500	0.58491900	-2.32262300
C	-1.97293800	-0.53859500	-3.29033700
C	-0.53009400	-0.23969200	-3.64496700
C	-0.57997400	0.88184100	-2.65827600
C	1.69141900	1.76157200	-2.23153900
C	2.39519800	0.88033200	-3.06128100
H	1.87115300	0.25570000	-3.77738200
C	3.77630500	0.75220500	-2.91066000
C	3.77494200	2.41941300	-1.19569000

C	2.39857400	2.56336400	-1.31884600
H	1.85687500	3.24836800	-0.67932500
C	-5.23829300	1.20921900	-0.71365100
C	-5.42370600	2.39452300	-1.67575800
H	-5.63949700	2.04574200	-2.69324700
H	-6.26594600	3.01263600	-1.34524500
H	-4.53733700	3.02798800	-1.71474800
C	-6.54303300	0.39277600	-0.68363300
H	-6.47025000	-0.46779200	-0.00992700
H	-7.36271900	1.02437500	-0.32534800
H	-6.81457800	0.02975700	-1.68169200
C	-4.94158600	1.72056000	0.70721600
H	-4.01497200	2.29274100	0.76519900
H	-5.75773500	2.37796900	1.02768100
H	-4.86403900	0.89757400	1.42221100
N	-4.35547500	-2.11351600	-0.64654900
N	-2.84224400	1.03992400	-1.46119800
H	-2.51805900	1.64168500	-0.69530200
N	0.28716000	1.81039900	-2.18245800
H	-0.07689800	2.54937400	-1.56867500
O	-3.09073500	-0.74252800	0.63203000
O	-2.79233800	-1.38996200	-3.60869400
O	0.29534900	-0.77310200	-4.37094000
F	3.98532500	-0.50168300	-4.91259900
F	5.80434900	-0.12257100	-3.77602200
F	5.77734600	3.43789500	-0.44583900
F	4.55493800	2.43057500	1.03975300
C	4.48374700	1.50495100	-1.97747200
H	5.55404600	1.38765000	-1.86433200

C	4.49827500	3.16239900	-0.11024300
C	4.47575400	-0.33609000	-3.67268500
F	4.32806100	-1.53750500	-3.03166600
F	3.89993300	4.32971100	0.20887600
C	1.47524100	-5.09013900	2.58511300
H	1.95756900	-5.50126300	3.46816600
C	3.68588800	-4.96214300	1.44105200
H	4.17017800	-5.36598300	2.32613600
C	2.29304600	-4.78618500	1.44256800
C	4.44480600	-4.60556300	0.32712700
H	5.52296600	-4.73520900	0.34810400
C	3.83613000	-4.08083200	-0.81362100
H	4.43545900	-3.79091200	-1.67148500
C	1.66175900	-4.25727300	0.27630900
C	0.13083900	-4.86119500	2.57826200
H	-0.47029200	-5.09637900	3.45347100
C	-0.38197000	-3.51873600	-0.90025500
C	2.44344000	-3.91419000	-0.86958900
C	0.25374400	-4.04128800	0.26423500
C	-0.53037600	-4.32106400	1.42091700
C	-1.90919800	-4.05521800	1.39694700
C	-2.53401800	-3.52566600	0.26481400
C	-1.76041700	-3.27385500	-0.87470300
H	-2.22058000	-2.84573900	-1.75738500
C	-4.66629900	-3.09018300	1.61910800
H	-5.71485000	-2.79923700	1.50471000
H	-4.15007000	-2.31168800	2.18136100
H	-4.64343300	-4.02595300	2.18704900
H	-2.48534100	-4.26666100	2.29175000

C	1.77431500	-3.39504400	-2.03301400
C	0.42366400	-3.22216000	-2.05473900
H	-0.05831500	-2.83114700	-2.94501400
H	2.37249900	-3.12119900	-2.89593000
S	-1.17513600	4.02402500	0.45969000
O	-0.11758800	4.14491400	-0.58070900
O	-1.70542000	2.63239700	0.56296400
O	-0.89934900	4.68265000	1.74523600
F	-2.28362900	6.29440800	-0.31496200
F	-2.80781900	4.56816600	-1.53273500
C	-2.57850300	4.99357300	-0.27473300
F	-3.70172700	4.82590700	0.43698200
C	-0.25718700	1.96114300	3.33826800
C	0.66449400	1.73309900	2.36036100
C	0.58802700	0.52464800	1.52328500
C	-0.39276700	-0.42503500	1.73802300
C	-1.26634000	-0.29001100	2.83953800
C	-1.37581300	1.04977700	3.53099000
O	-2.32802700	1.28734100	4.26168900
O	1.69688000	2.51226000	2.04415100
C	1.91045600	3.71168300	2.80549800
O	1.50025800	0.47159500	0.57116300
C	1.51082900	-0.68189200	-0.28448300
H	-0.27733800	2.88717500	3.89583800
H	-0.51548700	-1.26862500	1.07399600
H	-2.19739400	-0.84293700	2.78203100
H	2.81902600	4.14321300	2.39283800
H	2.05334800	3.45921200	3.86290600
H	1.06290400	4.38696800	2.67422000

H	2.41436000	-0.60379900	-0.88011400
H	0.62871300	-0.67566300	-0.92452900
H	1.52889700	-1.59047800	0.31311900
C	3.31920700	-1.47651000	2.29020500
C	4.16937300	-0.38842400	2.47792700
C	3.92177200	0.54238500	3.50083900
C	2.80948300	0.39759500	4.30453300
C	1.87794500	-0.65072300	4.07362500
C	2.16697000	-1.60037000	3.06148400
C	0.62673500	-0.61893500	4.75172600
C	-0.51281500	-1.33357000	4.35509000
H	3.53825800	-2.21235400	1.52576800
H	5.03134700	-0.25543100	1.83240500
H	4.58973800	1.38609700	3.63510000
H	2.59045900	1.12881700	5.07740700
H	1.50975100	-2.44625400	2.90561000
H	0.51124100	0.12539200	5.53540800
H	-1.37908300	-1.29036700	5.00884100
H	-0.39576800	-2.26506800	3.80881600

128

B3

C	-5.18575500	-1.24501600	0.11447700
C	-6.32233400	-1.89897300	-0.72895100
H	-7.28889700	-1.67276600	-0.26328800
H	-6.21365900	-2.98557100	-0.76030500
C	-6.24096400	-1.23600100	-2.10662300

H	-7.18662000	-1.27852100	-2.65436400
H	-5.46863200	-1.70041700	-2.72608700
C	-5.82671500	0.19659500	-1.77518600
H	-6.69263700	0.80549000	-1.48303300
H	-5.31107700	0.66471100	-2.60986400
C	-3.88528000	0.82007300	-0.25855100
C	-3.58286800	2.09326200	-1.07038400
H	-4.09663700	2.06788900	-2.03018500
C	-1.66145900	1.45137000	-2.41356300
C	-2.25779400	0.49160100	-3.38026300
C	-0.84602300	0.21774000	-3.89671000
C	-0.37213900	1.26254900	-2.95602800
C	2.06446700	1.27613700	-2.66253500
C	2.28663300	-0.00694700	-3.19164600
H	1.49487700	-0.52661100	-3.71817300
C	3.52419200	-0.61276700	-3.01246400
C	4.33159600	1.30107600	-1.81992700
C	3.11026600	1.94593300	-2.00714600
H	2.93898500	2.91967900	-1.56382300
C	-4.04808700	3.38773200	-0.33501100
C	-3.57135100	4.60310200	-1.15059600
H	-3.90853800	4.53809500	-2.19227500
H	-3.98207200	5.52269300	-0.71881800
H	-2.48200400	4.68927500	-1.15204300
C	-5.58628000	3.37928500	-0.28843200
H	-5.96756700	2.51395300	0.26573000
H	-5.95068600	4.28125100	0.21491200
H	-6.01912500	3.35975200	-1.29576900
C	-3.50450500	3.48189700	1.10237800

H	-2.41931800	3.37727400	1.14523600
H	-3.76366400	4.46178500	1.51924400
H	-3.93576500	2.70905900	1.74307000
N	-4.91871900	0.02201300	-0.62922700
N	-2.15180700	2.13362200	-1.37780700
H	-1.50350800	2.40342700	-0.63086300
N	0.80505900	1.86945600	-2.65597200
H	0.73535500	2.74743100	-2.12727700
O	-3.16676600	0.54685100	0.70811200
O	-3.38041800	0.07344300	-3.61880300
O	-0.34790300	-0.59411600	-4.65928500
F	3.62608100	-2.89139400	-2.39332100
F	2.80516800	-2.43834500	-4.35574200
F	6.61064500	1.58291000	-1.23522400
F	5.16300400	1.42811800	0.37865300
C	4.55902500	0.01829800	-2.31742700
H	5.50938600	-0.47753900	-2.16085300
C	5.34514000	1.90795500	-0.89478300
C	3.72109300	-2.03358200	-3.45823100
F	4.94386000	-2.23651500	-3.99052600
F	5.26343300	3.24371700	-0.82269300
C	-0.93945700	-5.25307200	2.38197900
H	-0.67928900	-5.85461500	3.24973400
C	1.20480600	-5.90388000	1.29284800
H	1.47833400	-6.48177100	2.17186000
C	-0.02984900	-5.23593400	1.26863700
C	2.07832200	-5.81523500	0.21034300
H	3.03587600	-6.32655900	0.24947700
C	1.73717700	-5.07960000	-0.92472800

H	2.42876400	-5.00831300	-1.75798000
C	-0.38190400	-4.47053700	0.11468900
C	-2.09428400	-4.52853400	2.36520600
H	-2.76594800	-4.54537000	3.22041100
C	-1.95039100	-2.96229900	-1.05391900
C	0.50730200	-4.40649800	-1.00256000
C	-1.60065300	-3.73487400	0.09119900
C	-2.46061100	-3.73139300	1.22677100
C	-3.61259100	-2.93036800	1.21318400
C	-3.93492400	-2.13252600	0.11306400
C	-3.11243300	-2.18126300	-1.01909500
H	-3.33917700	-1.56067000	-1.87775700
C	-5.70170900	-0.92363600	1.52444200
H	-6.51796200	-0.19879700	1.44630500
H	-4.91643800	-0.49650100	2.14881600
H	-6.10166000	-1.82055600	2.00841600
H	-4.23920000	-2.92429400	2.09872600
C	0.11556400	-3.64139900	-2.15520700
C	-1.06455100	-2.96495700	-2.18628600
H	-1.32912700	-2.37808700	-3.05952500
H	0.79699400	-3.58842900	-2.99882900
S	0.82196200	3.81202700	0.31935000
O	0.70827900	4.25746500	-1.09463100
O	-0.12244700	2.70844200	0.65699500
O	2.20339200	3.62411300	0.81152500
F	0.97942800	6.32557200	1.11423100
F	-1.05419000	5.57013700	0.96040000
C	0.20269600	5.25637700	1.30723900
F	0.21838600	4.94357200	2.61481000

C	-0.34232600	-0.52296500	1.32096100
C	-0.04657500	0.10180900	2.49489400
C	1.20466500	0.86049300	2.64358100
C	2.19447300	0.78616100	1.67897200
C	1.97735600	0.01651500	0.51643900
C	0.56849000	-0.41020100	0.19478000
O	0.25329600	-0.68195600	-0.95671800
O	-0.81066400	0.12829600	3.58854200
C	-2.06218100	-0.57035500	3.52499700
O	1.24878800	1.61140300	3.72884400
C	2.35659900	2.51487300	3.89725400
H	-1.30823300	-0.96816400	1.13190300
H	3.09573600	1.37543200	1.75637500
H	2.57869900	0.26610100	-0.34651200
H	-2.53091900	-0.40829700	4.49586900
H	-1.88787000	-1.63987800	3.36237800
H	-2.67718300	-0.16960600	2.71673000
H	2.13336200	3.06709500	4.80902700
H	2.42219200	3.18451400	3.03685900
H	3.28534100	1.94799900	4.01958700
C	3.10999400	-0.93126100	4.95378300
C	2.08277700	-1.40209500	5.76851400
C	1.10167200	-2.26161800	5.24819700
C	1.14242000	-2.63347500	3.91788600
C	2.15996700	-2.13978400	3.06135800
C	3.15013300	-1.28673300	3.60740300
C	2.07470400	-2.40892800	1.66422000
C	2.84356100	-1.77690300	0.68343100
H	3.87797800	-0.28353900	5.36496400

H	2.04427300	-1.10985600	6.81359100
H	0.30475900	-2.62476700	5.88956200
H	0.37221900	-3.27370500	3.49851800
H	3.96685900	-0.93895900	2.98657500
H	1.26855800	-3.05379400	1.33535400
H	2.73542200	-2.11957900	-0.33975700
H	3.82497100	-1.38026300	0.92641800

128

B4

C	4.37260100	2.89281400	0.42669500
C	5.28341200	3.99262000	-0.18888500
H	6.27118000	3.94311300	0.28476400
H	4.87512300	4.99024300	-0.01005600
C	5.39846700	3.63256300	-1.67221200
H	6.28568200	4.06232900	-2.14532600
H	4.52185700	3.97704300	-2.22803300
C	5.44172100	2.10287700	-1.65418200
H	6.46347200	1.74493100	-1.48489800
H	5.04948500	1.67009700	-2.57363200
C	3.88128400	0.60125700	-0.32576300
C	4.12517300	-0.58992100	-1.27183500
H	4.49948400	-0.24141200	-2.23380500
C	1.97755100	-0.64802300	-2.38329400
C	2.12573700	0.51679600	-3.29218300
C	0.64765100	0.39156700	-3.62610300
C	0.59831300	-0.77484500	-2.69759800

C	-1.74093700	-1.40772000	-2.20936500
C	-2.34880000	-0.23465800	-2.68365800
H	-1.76539800	0.53032100	-3.18262100
C	-3.72757600	-0.07797800	-2.55515500
C	-3.91166300	-2.23161200	-1.52969400
C	-2.53765600	-2.41019900	-1.62699000
H	-2.06614200	-3.30941100	-1.25078300
C	5.16910600	-1.59838800	-0.69590900
C	5.27828800	-2.78466100	-1.66827500
H	5.54739100	-2.44345600	-2.67550000
H	6.05829800	-3.47391900	-1.32582800
H	4.34411500	-3.34144300	-1.73689400
C	6.54120600	-0.90564800	-0.61771500
H	6.52761700	-0.04939500	0.06467100
H	7.28702100	-1.61473700	-0.24322500
H	6.87608500	-0.56100300	-1.60279900
C	4.78917900	-2.09373800	0.71056300
H	3.80665600	-2.56557000	0.74314000
H	5.52647200	-2.83436000	1.04038100
H	4.78340000	-1.27236100	1.43262100
N	4.57859100	1.75118400	-0.51013900
N	2.82766900	-1.22980700	-1.53542300
H	2.42603100	-1.80948500	-0.78832800
N	-0.35639600	-1.61267400	-2.22414900
H	-0.05745100	-2.43324200	-1.68162700
O	3.05588700	0.48240800	0.58769400
O	3.02443300	1.29835600	-3.57116700
O	-0.14351600	1.05253500	-4.28072300
F	-4.62532800	1.98153500	-1.83279500

F	-3.60280500	1.97454200	-3.75329500
F	-5.91152300	-3.49279600	-1.55663500
F	-5.13613400	-2.90707200	0.38378600
C	-4.52830900	-1.06633600	-1.98791400
H	-5.60225800	-0.93924600	-1.91544000
C	-4.76554900	-3.28020200	-0.87814800
C	-4.37100900	1.22153500	-2.94288500
F	-5.56050700	1.04099700	-3.55243100
F	-4.13452500	-4.46490800	-0.75244600
C	-1.06839500	5.02632400	2.68549800
H	-1.56431200	5.37847800	3.58657400
C	-3.21330700	5.22246000	1.43372200
H	-3.71150500	5.56340500	2.33739800
C	-1.84239800	4.92687500	1.47801400
C	-3.93472500	5.06784000	0.25125900
H	-4.99705100	5.29270600	0.23649500
C	-3.31063500	4.61713200	-0.91111900
H	-3.88585100	4.47472400	-1.81971200
C	-1.19316600	4.47496800	0.28905700
C	0.25093300	4.68286100	2.71336300
H	0.82097700	4.76294400	3.63612500
C	0.83775500	3.66952300	-0.86484700
C	-1.93969500	4.31459500	-0.91828100
C	0.19056500	4.14177800	0.31514900
C	0.92780900	4.22122700	1.53164600
C	2.27209900	3.81918700	1.54632800
C	2.90485100	3.33437800	0.39910700
C	2.18132700	3.28108900	-0.79788600
H	2.63760800	2.87174300	-1.69188000

C	4.86394900	2.51917600	1.83195500
H	5.87026400	2.09636700	1.75545700
H	4.20846500	1.78085700	2.29541100
H	4.92586500	3.40596600	2.47101900
H	2.80950700	3.87159500	2.48713900
C	-1.26438800	3.82316600	-2.08918700
C	0.06500300	3.52852500	-2.06969300
H	0.55058300	3.14523200	-2.96157000
H	-1.84261200	3.66965700	-2.99457800
S	0.86803700	-4.06756500	0.31476500
O	-0.10448000	-4.09213900	-0.81259900
O	1.51140100	-2.73097200	0.48136400
O	0.43718000	-4.70607600	1.56691500
F	1.83072500	-6.43581200	-0.34994800
F	2.57359200	-4.78982400	-1.56260900
C	2.23087500	-5.16281500	-0.31417500
F	3.31855500	-5.07329500	0.46463400
C	-0.46597300	-2.19809000	3.68984300
C	-0.83215700	-1.83090500	2.42661800
C	-0.26577800	-0.61998100	1.80682200
C	0.58289400	0.20847300	2.52515500
C	0.80481700	-0.03955200	3.89835200
C	0.48191400	-1.40830400	4.45784400
O	0.95084300	-1.75871100	5.53371500
O	-1.70782400	-2.45506500	1.64790500
C	-2.34806400	-3.63592700	2.15860900
O	-0.63107500	-0.42182500	0.55993700
C	-0.16818800	0.78529500	-0.07148600
H	-0.80241200	-3.12576400	4.13216400

H	1.07978300	1.03883300	2.04314200
H	1.68197200	0.41735100	4.35057500
H	-3.02690900	-3.95583500	1.37386200
H	-2.90996000	-3.38738900	3.06616100
H	-1.59675900	-4.40343600	2.34700300
H	-0.69103700	0.84382700	-1.01909000
H	0.91223000	0.73773500	-0.20106600
H	-0.43367000	1.64388300	0.54296300
C	-3.00345900	1.61212400	1.42197600
C	-3.98329400	0.65265700	1.17360500
C	-4.28352600	-0.32844100	2.13337900
C	-3.59058600	-0.35117400	3.32617400
C	-2.54466700	0.57913500	3.58006400
C	-2.27495600	1.57632700	2.60621800
C	-1.73639700	0.40017400	4.73511100
C	-0.49256200	1.01567500	4.95058400
H	-2.80482900	2.37580200	0.68150800
H	-4.52125100	0.67680200	0.23357100
H	-5.03775500	-1.07628700	1.92093100
H	-3.79557300	-1.11872700	4.06719400
H	-1.52803900	2.33782400	2.79566100
H	-2.04270300	-0.37544700	5.43240900
H	-0.03930800	0.88068500	5.92851500
H	-0.29273000	1.97590600	4.48326800

X. References

(1) Evans, D. A.; Cain, P. A.; Wong, R. Y. A General Approach to the Synthesis of Phenanthrenoid Compounds. An Alternative to Oxidative Phenolic Coupling. *J. Am. Chem. Soc.* **1977**, *99*, 7083–7085.

(2) Fu, Z.; Song, C.; Qi, Y.; Pei, J.; Zhu, X.; Zhao, Y.; Luo, Y.; Li, X. Polar Comonomers Regulate and Control the Copolymerization of Conjugated Dienes and Methoxystyrenes Catalyzed by a CGC-type Rare-earth Metal Catalyst. *Polym. Chem.* **2024**, *15*, 516–521.

(3) Malkoch, M.; Thibault, R. J.; Drockenmuller, E.; Messerschmidt, M.; Voit, B.; Russell, T. P.; Hawker, C. J. Orthogonal Approaches to the Simultaneous and Cascade Functionalization of Macromolecules Using Click Chemistry. *J. Am. Chem. Soc.* **2005**, *127*, 14942–14949.

(4) Ge, L.; Wang, D.-X.; Xing, R.; Ma, D.; Walsh, P. J.; Feng, C. Photoredox-Catalyzed Oxo-amination of Aryl Cyclopropanes. *Nat. Commun.* **2019**, *10*, 4367.

(5) Huang, C.-Y.; Doyle, A. G. Nickel-catalyzed Negishi Alkylations of Styrenyl Aziridines. *J. Am. Chem. Soc.* **2012**, *134*, 9541–9544.

(6) Iwasaki, M.; Tezuka, M. Carbonylative Coupling of Allyl Esters with Terminal Alkynes Catalyzed by Palladium Complexes. *Saitama Kogyo Daigaku Kiyo*, **2002**, *11-12*, 69–72.

(7) Carlet, F.; Bertarini, G.; Broggini, G.; Pradal, A.; Poli, G. Oxoammonium-mediated Allylsilane–ether Coupling Reaction. *Eur. J. Org. Chem.* **2021**, 2162–2168.

(8) Benoit, G.; Charette, A. B. Diastereoselective Borocyclopropanation of Allylic Ethers Using a Boromethylzinc Carbenoid. *J. Am. Chem. Soc.* **2017**, *139*, 1364–1367.

(9) Uchida, I.; Itoh, Y.; Namiki, T.; Nishikawa, M.; Hashimoto, M. Structure and Synthesis of WF 3681, A Novel Aldose Reductase Inhibitor. *Tetrahedron Lett.* **1986**, *27*, 2015–2018.

(10) Laali, K. K.; Arrica, M. A.; Okazaki, T.; Bunge, S. D. Synthesis and Stable-ion Studies of Regioisomeric Acetylnitropyrenes and Nitropyrenyl Carbinols and GIAO-DFT Study of Nitro Substituent Effects on α-Pyrenyl Carbocations. *Eur. J. Org. Chem.* **2008**, 6093–6105.

(11) Goodell, J. R.; McMullen, J. P.; Zaborenko, N.; Maloney, J. R.; Ho, C. X.; Jensen, K. F.; Porco, J. A., Jr.; Beeler, A. B. Development of an Automated Microfluidic Reaction Platform for Multidimensional Screening: Reaction Discovery Employing Bicyclo[3.2.1]octanoid Scaffolds. *J. Org. Chem.* **2009**, *74*, 6169–6180.

(12) Liu, Y.; Wang, X.; Chen, S.; Fu, S.; Liu, B. Iron-catalyzed Intramolecular Perezone-type [5 + 2] Cycloaddition: Access to Tricyclo[6.3.1.0^{1,6}]dodecane. *Org. Lett.* **2018,** *20*, 2934–2938.

(13) (a) Nielsen, C. D.-T.; Burés, J. Visual Kinetic Analysis. *Chem. Sci.* **2019**, *10*, 348–353. (b) Burés, J. A Simple Graphical Method to Determine the Order in Catalyst. *Angew. Chem., Int. Ed.* **2016**, *55*, 2208–2031. (c) Burés, J. Variable Time Normalization Analysis: General Graphical Elucidation of Reaction Orders from Concentration Profiles. *Angew. Chem., Int. Ed.* **2016**, *55*, 16084–16087.

(14) Frisch, M. J., Trucks, G. W., Schlegel, H. B., Scuseria, G. E., Robb, M. A., Cheeseman, J. R., Scalmani, G., Barone, V., Petersson, G. A., Nakatsuji, H., Li, X., Caricato, M., Marenich, A. V., Bloino, J., Janesko, B. G., Gomperts, R., Mennucci, B., Hratchian, H. P., Ortiz, J. V., Izmaylov, A. F., Sonnenberg, J. L., Williams-Young, D., Ding, F., Lipparini, F., Egidi, F., Goings, J., Peng, B., Petrone, A., Henderson, T., Ranasinghe, D., Zakrzewski, V. G., Gao, J., Rega, N., Zheng, G., Liang, W., Hada, M., Ehara, M., Toyota, K., Fukuda, R., Hasegawa, J., Ishida, M., Nakajima, T., Honda, Y., Kitao, O., Nakai, H., Vreven, T., Throssell, K., Montgomery, J. A., Jr., Peralta, J. E., Ogliaro, F., Bearpark, M. J., Heyd, J. J., Brothers, E. N., Kudin, K. N., Staroverov, V. N., Keith, T. A., Kobayashi, R., Normand, J., Raghavachari, K., Rendell, A. P., Burant, J. C., Iyengar, S. S., Tomasi, J., Cossi, M., Millam, J. M., Klene, M., Adamo, C., Cammi, R., Ochterski, J. W.,

Martin, R. L., Morokuma, K., Farkas, Ö., Foresman, J. B. & Fox, D. J. *Gaussian 16*, C.01. Gaussian, Inc.: Wallingford, CT, 2019.

(15) Ditchfield, R., Hehre, W. J.; Pople, J. A. Self-consistent Molecular-orbital Methods. IX. An Extended Gaussian-type Basis for Molecular-orbital Studies of Organic Molecules. *J. Chem. Phys.* **1971**, *54*, 724–728.

(16) Grimme, S., Antony, J., Ehrlich, S. & Krieg, H. A Consistent and Accurate ab Initio Parametrization of Density Functional Dispersion Correction (DFT-D) for the 94 Elements H-Pu. *J. Chem. Phys.* **2010**, *132*, 154104.

(17) CYLview20; Legault, C. Y., Université de Sherbrooke, 2020 (http://www.cylview.org).

(18) Zhao, Y.; Truhlar, D. G. The M06 Suite of Density Functionals for Main Group Thermochemistry, Thermochemical Kinetics, Noncovalent Interactions, Excited States, and Transition Elements: Two New Functionals and Systematic Testing of Four M06-class Functionals and 12 Other Functionals. *Theor. Chem. Acc.* **2008**, *120*, 215–241.

(19) Lu, T.; Chen, F. Multiwfn: A Multifunctional Wavefunction Analyzer. *J. Comput. Chem.* **2012**, *33*, 580–592.

(20) Humphrey, W.; Dalke, A.; Schulten, K. VMD: Visual Molecular Dynamics. *J. Mol. Graph.* **1996**, *14*, 33–38.

(21) Banik, S. M.; Levina, A.; Hyde, A. M.; Jacobsen, E. N. Lewis Acid Enhancement by Hydrogen-bond Donors for Asymmetric Catalysis. *Science* **2017**, *358*, 761–764.

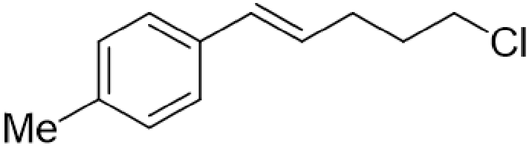

2w

[1]**H NMR** (400 MHz, $CDCl_3$)

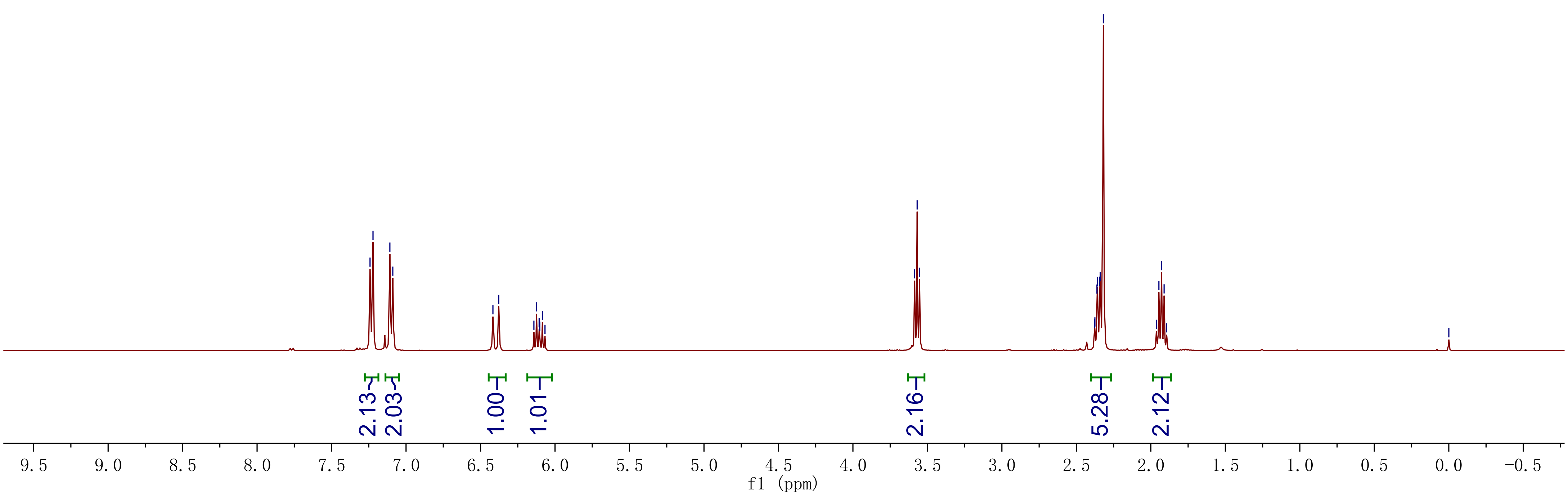

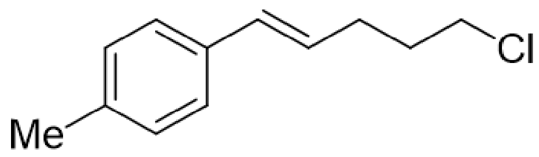

2w

^{13}C NMR (101 MHz, $CDCl_3$)

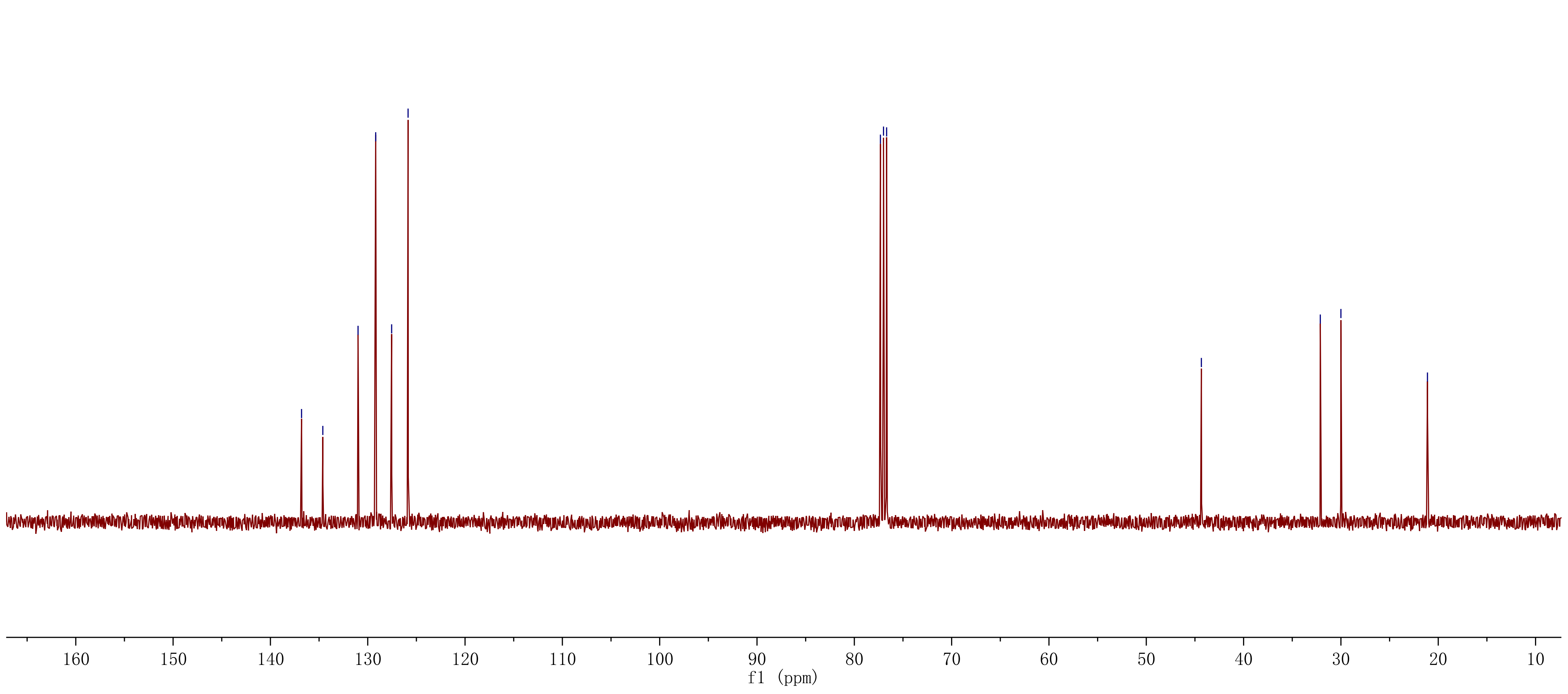

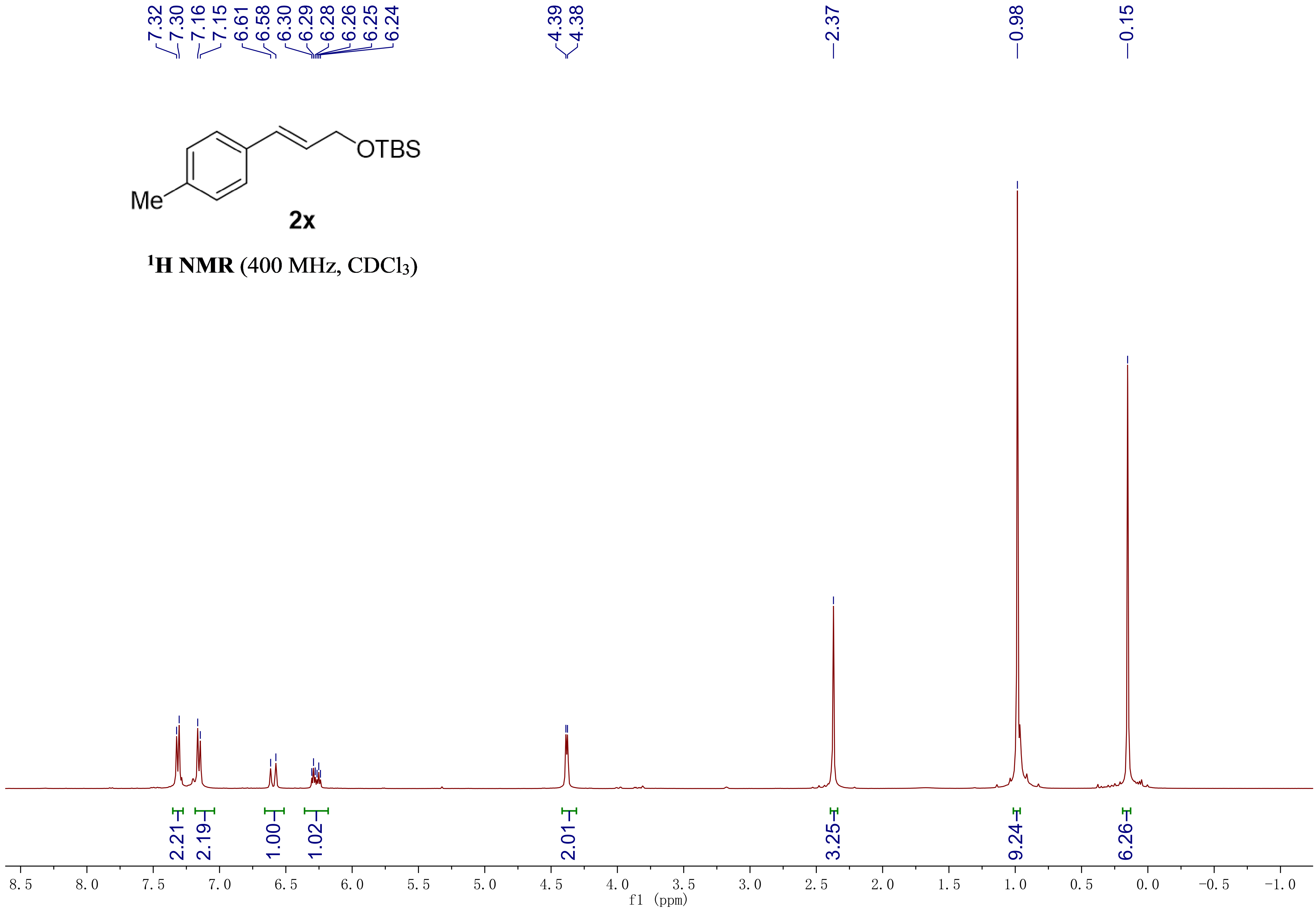

7.32
7.30
7.16
7.15
6.61
6.58
6.30
6.29
6.28
6.26
6.25
6.24
4.39
4.38
2.37
0.98
0.15
OTBS
Me
2x
1H NMR (400 MHz, CDCl3)
2.21
2.19
1.00
1.02
2.01
3.25
9.24
6.26
8.5
8.0
7.5
7.0
6.5
6.0
5.5
5.0
4.5
4.0
3.5
3.0
2.5
2.0
1.5
1.0
0.5
0.0
-0.5
-1.0
f1 (ppm)

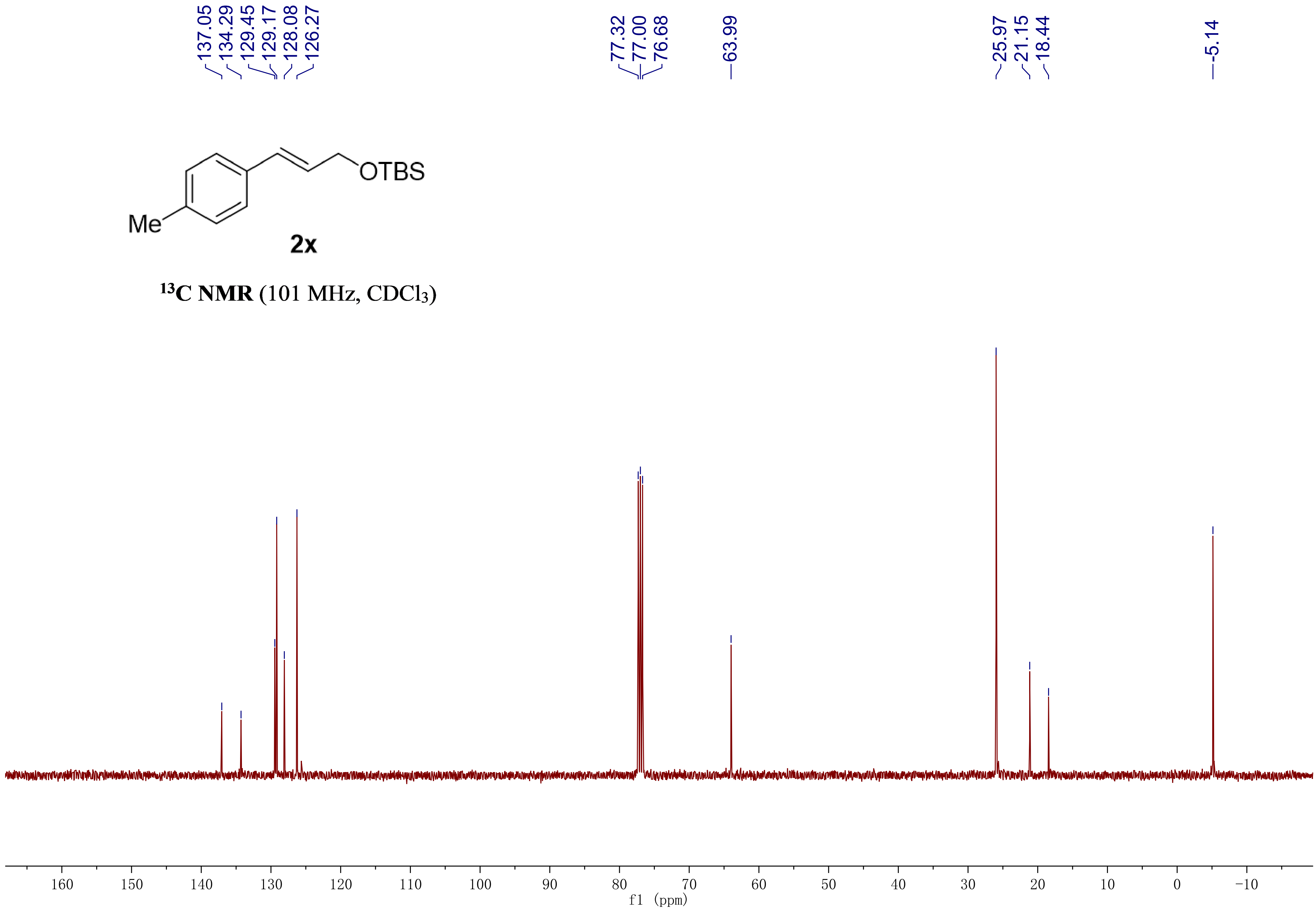

^{13}C NMR (101 MHz, $CDCl_3$)

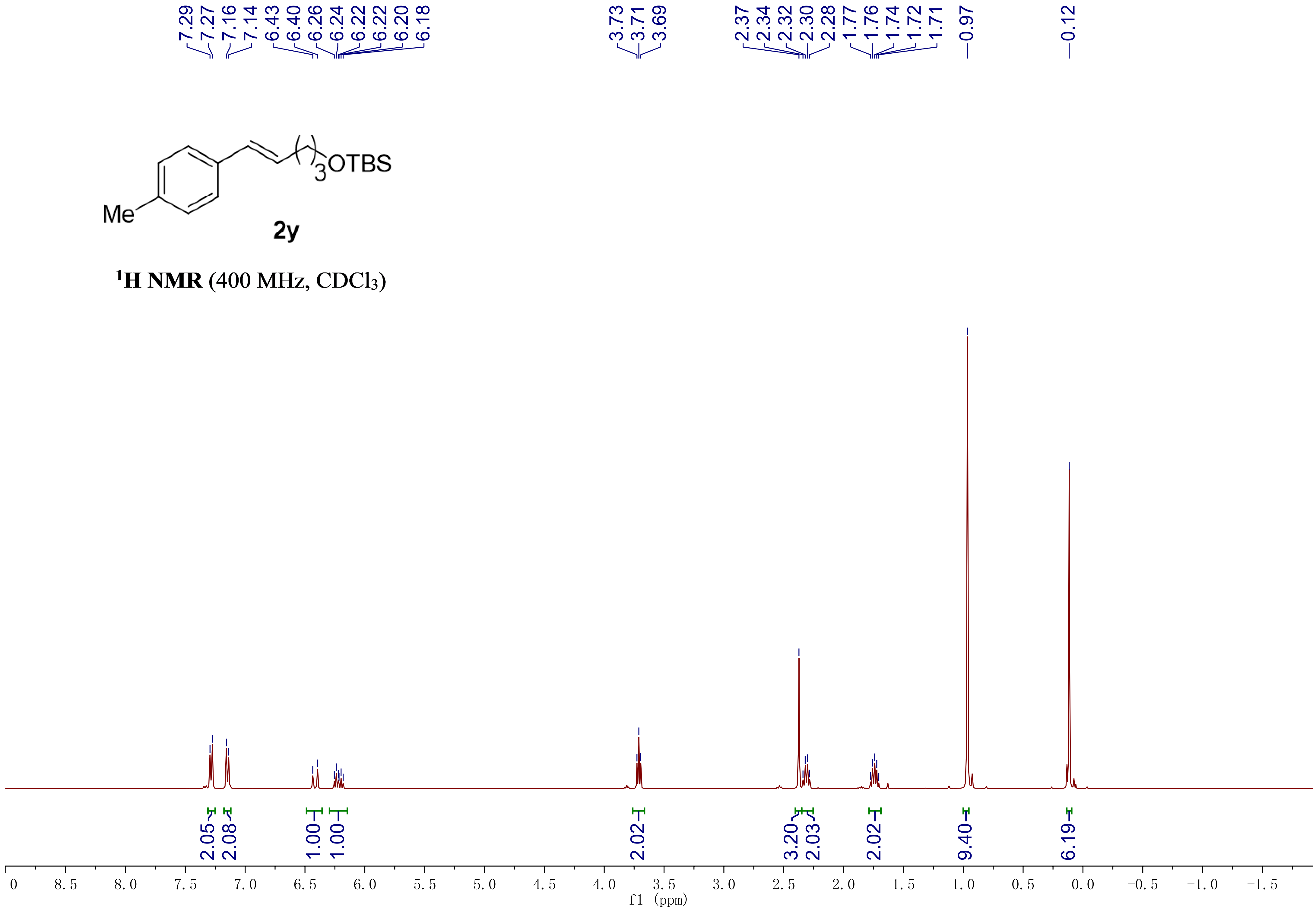
7.29
7.27
7.16
7.14
6.43
6.40
6.26
6.24
6.22
6.22
6.20
6.18
3.73
3.71
3.69
2.37
2.34
2.32
2.30
2.28
1.77
1.76
1.74
1.72
1.71
0.97
0.12
Me
3
OTBS
2y
1H NMR (400 MHz, CDCl3)
2.05
2.08
1.00
1.00
2.02
3.20
2.03
2.02
9.40
6.19
f1 (ppm)

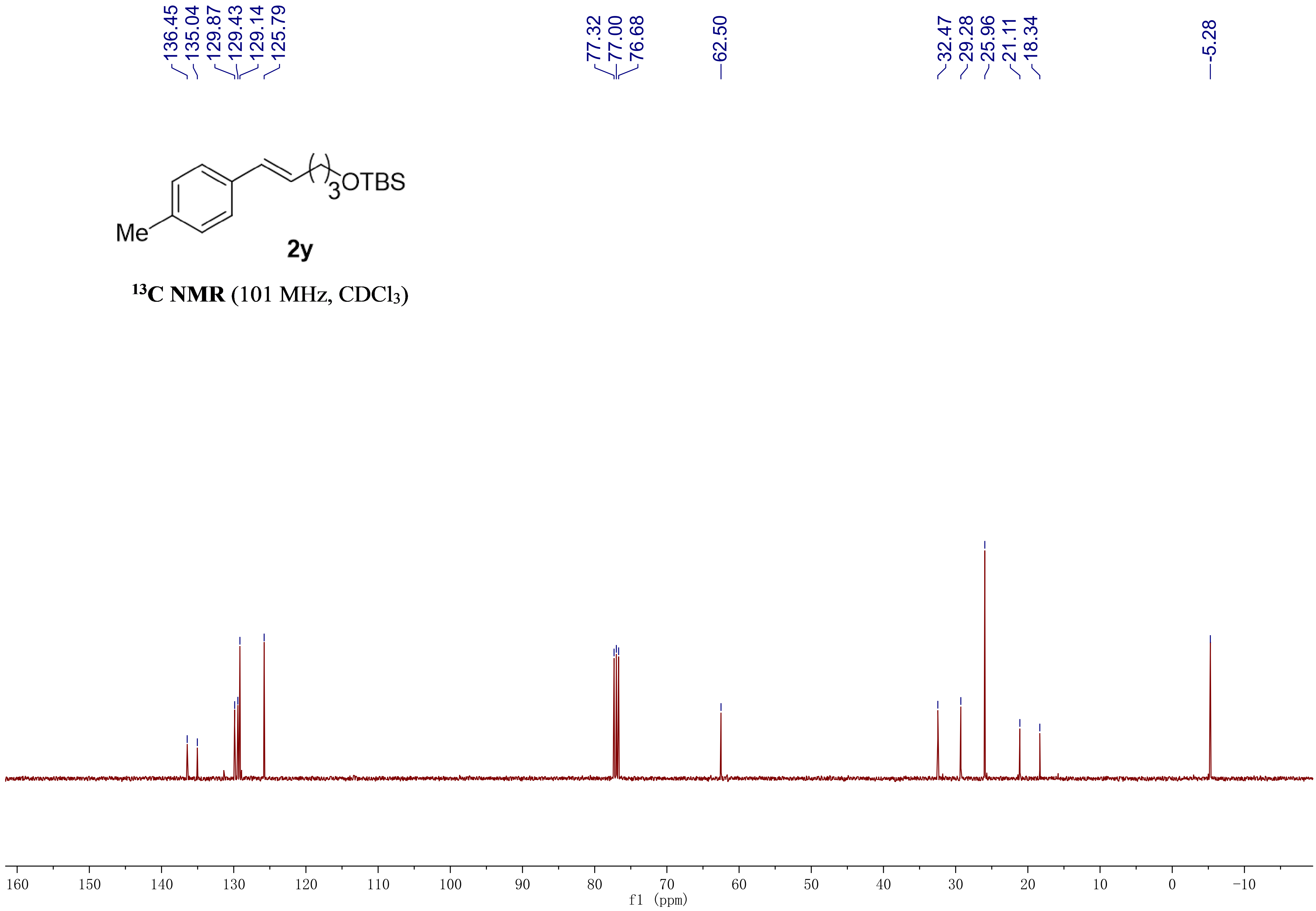

136.45
135.04
129.87
129.43
129.14
125.79
77.32
77.00
76.68
62.50
32.47
29.28
25.96
21.11
18.34
-5.28
Me
3OTBS
2y
13C NMR (101 MHz, CDCl3)
160 150 140 130 120 110 100 90 80 70 60 50 40 30 20 10 0 −10
f1 (ppm)

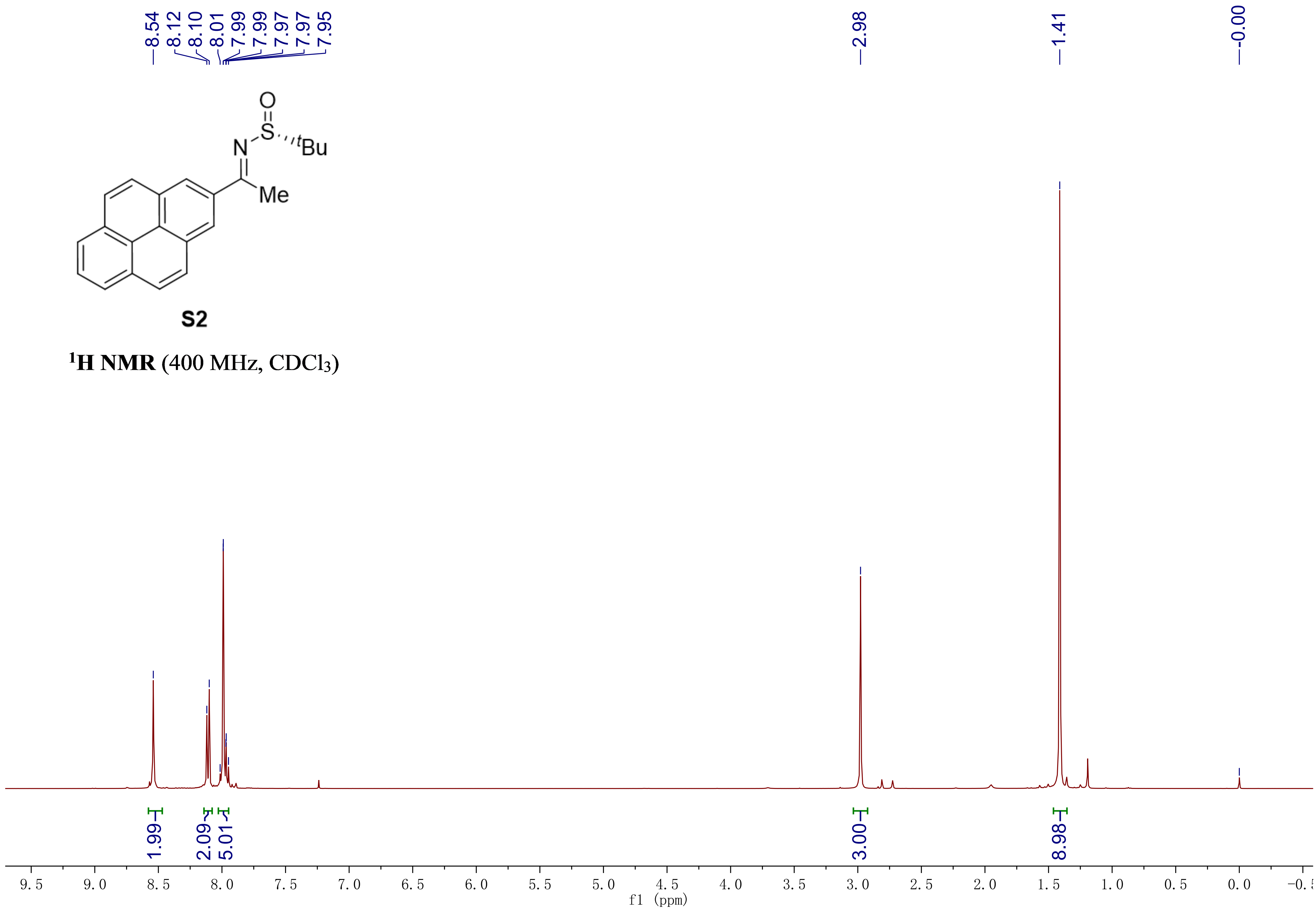

S2

^{1}H NMR (400 MHz, $CDCl_3$)

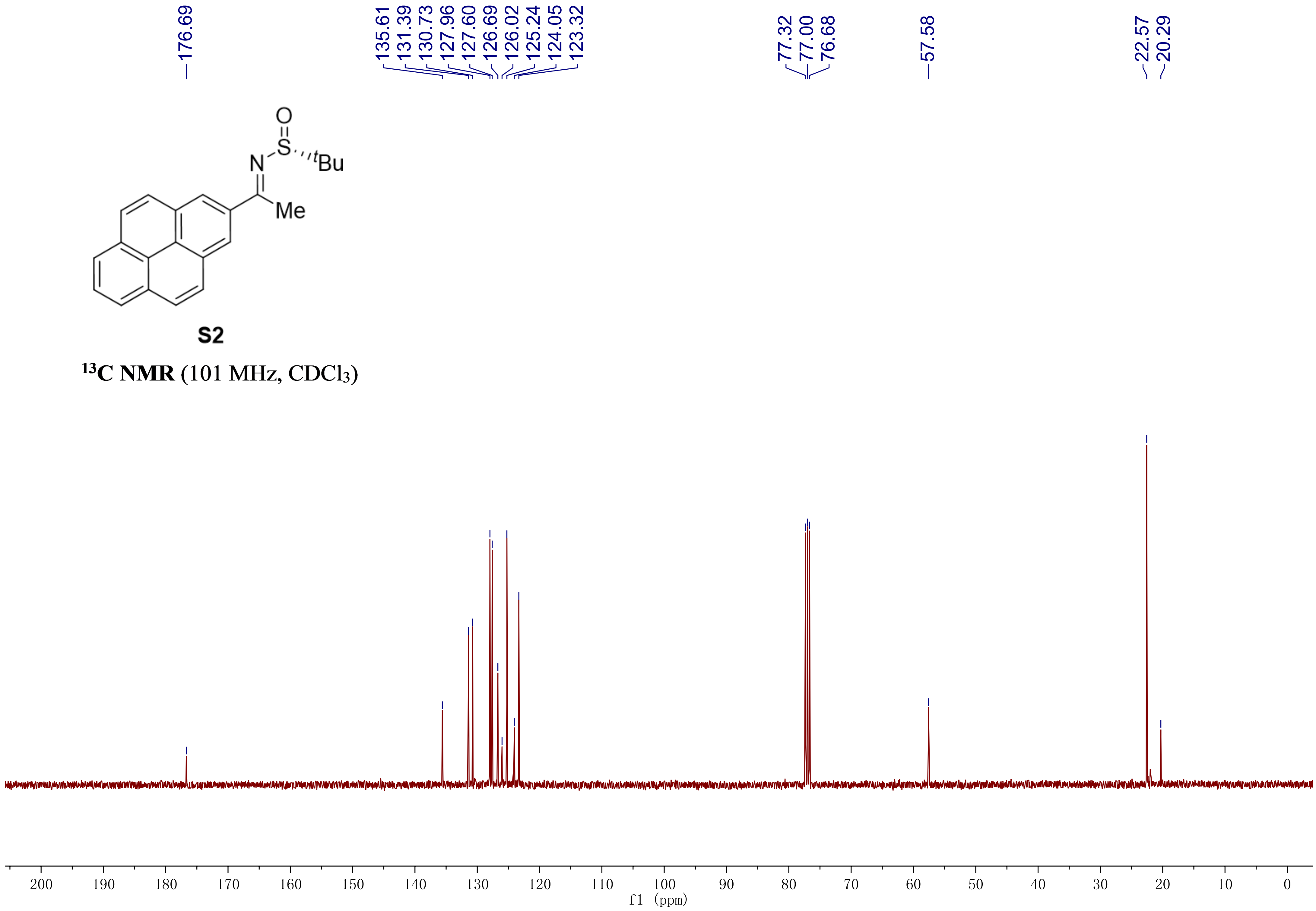

S2

^{13}C NMR (101 MHz, $CDCl_3$)

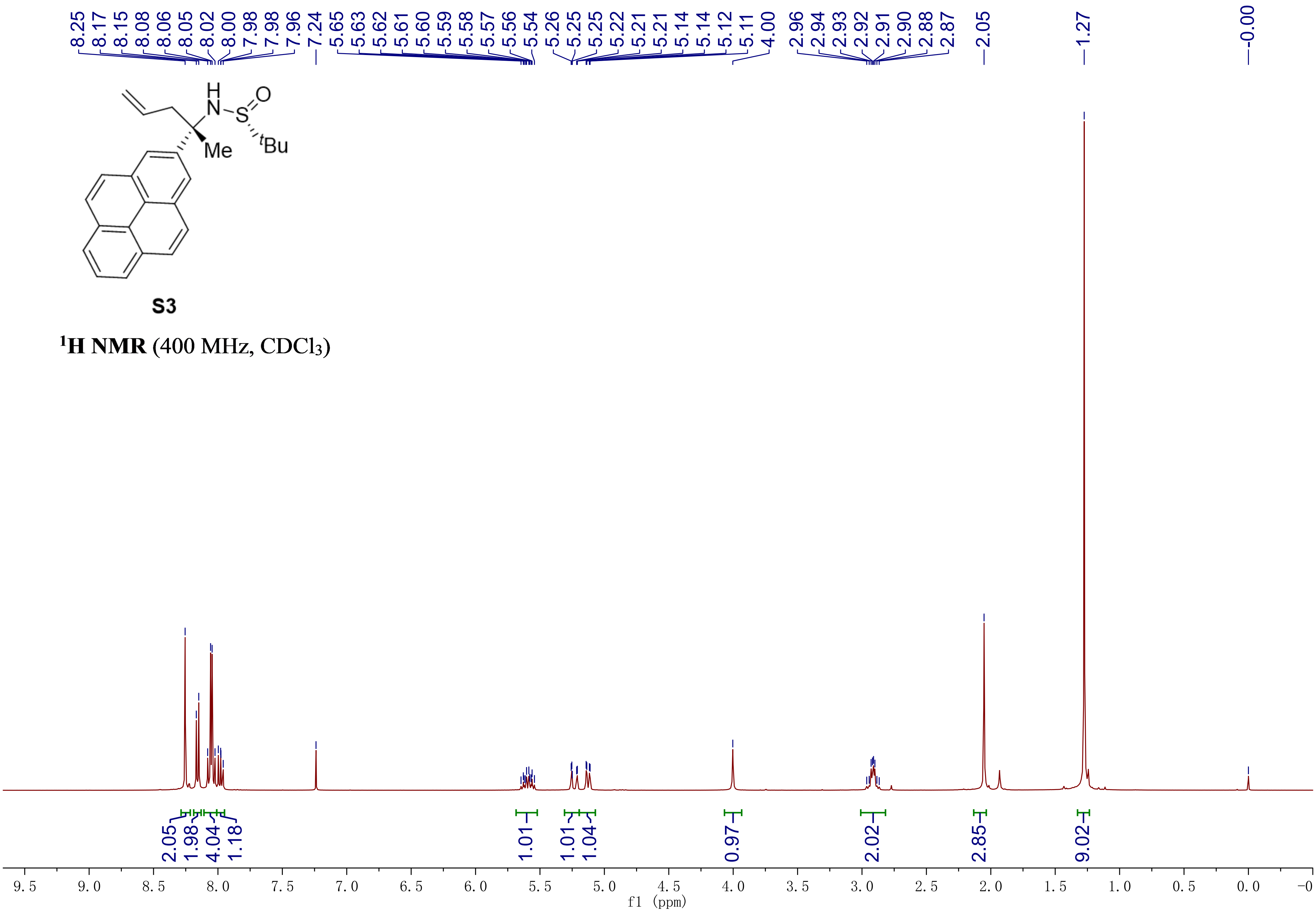

8.25 8.17 8.15 8.08 8.06 8.05 8.02 8.00 7.98 7.98 7.96 7.24 5.65 5.63 5.62 5.61 5.60 5.59 5.58 5.57 5.56 5.54 5.26 5.25 5.25 5.22 5.21 5.21 5.14 5.14 5.12 5.11 4.00 2.96 2.94 2.93 2.92 2.91 2.90 2.88 2.87 2.05 1.27 -0.00
H
N
S
O
Me
tBu
S3
1H NMR (400 MHz, CDCl3)
2.05 1.98 4.04 1.18 1.01 1.01 1.04 0.97 2.02 2.85 9.02
9.5 9.0 8.5 8.0 7.5 7.0 6.5 6.0 5.5 5.0 4.5 4.0 3.5 3.0 2.5 2.0 1.5 1.0 0.5 0.0 -0
f1 (ppm)

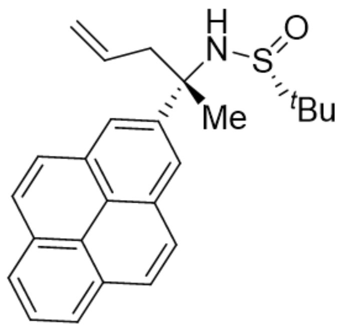

S3

^{13}C NMR (101 MHz, $CDCl_3$)

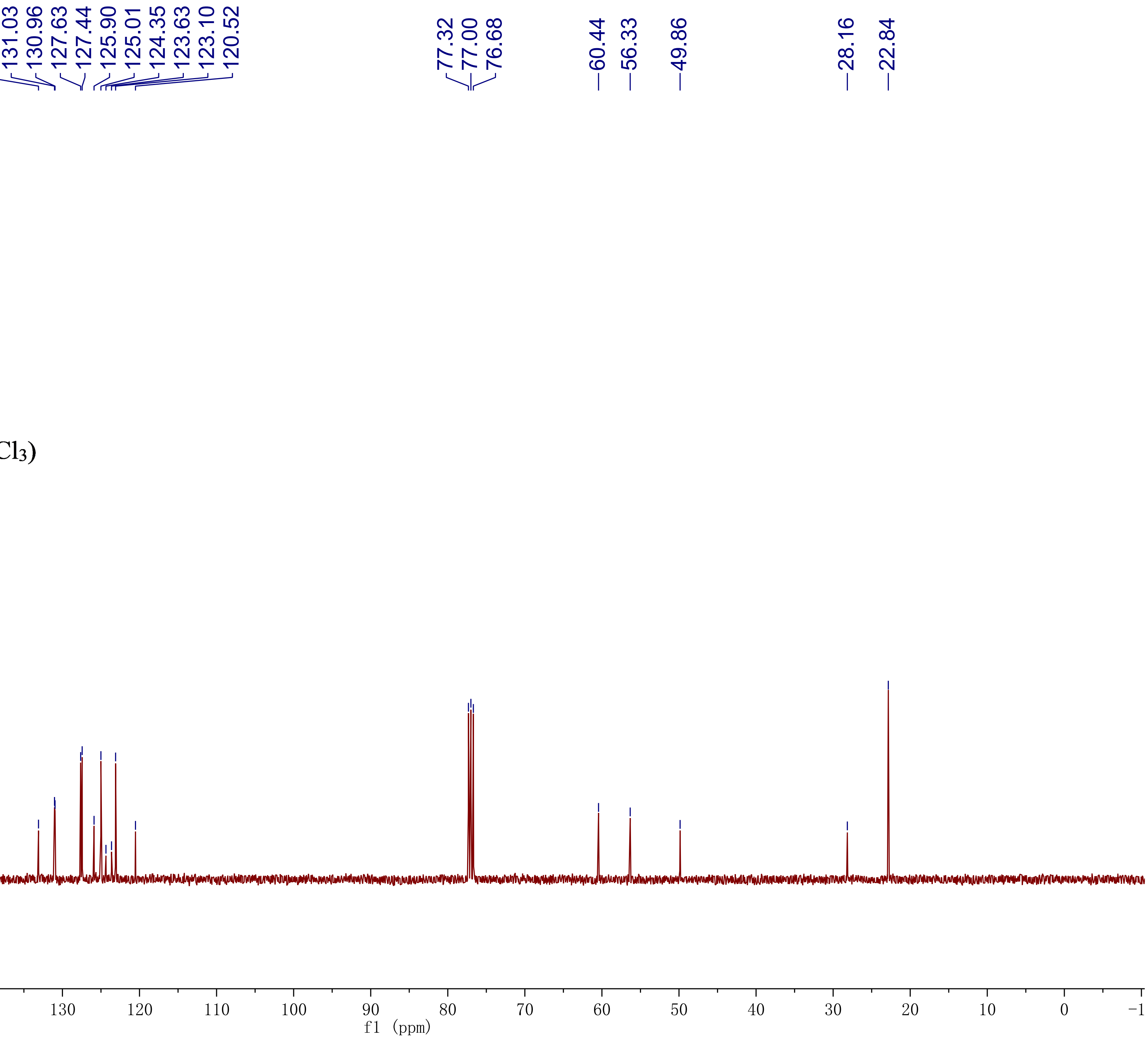

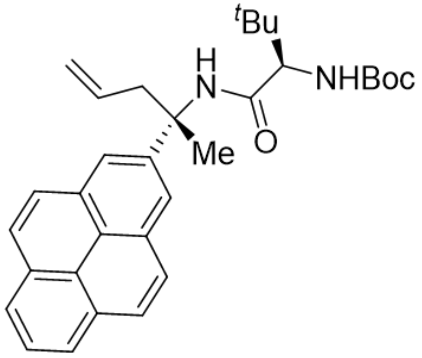

S4

^{1}H NMR (400 MHz, $CDCl_3$)

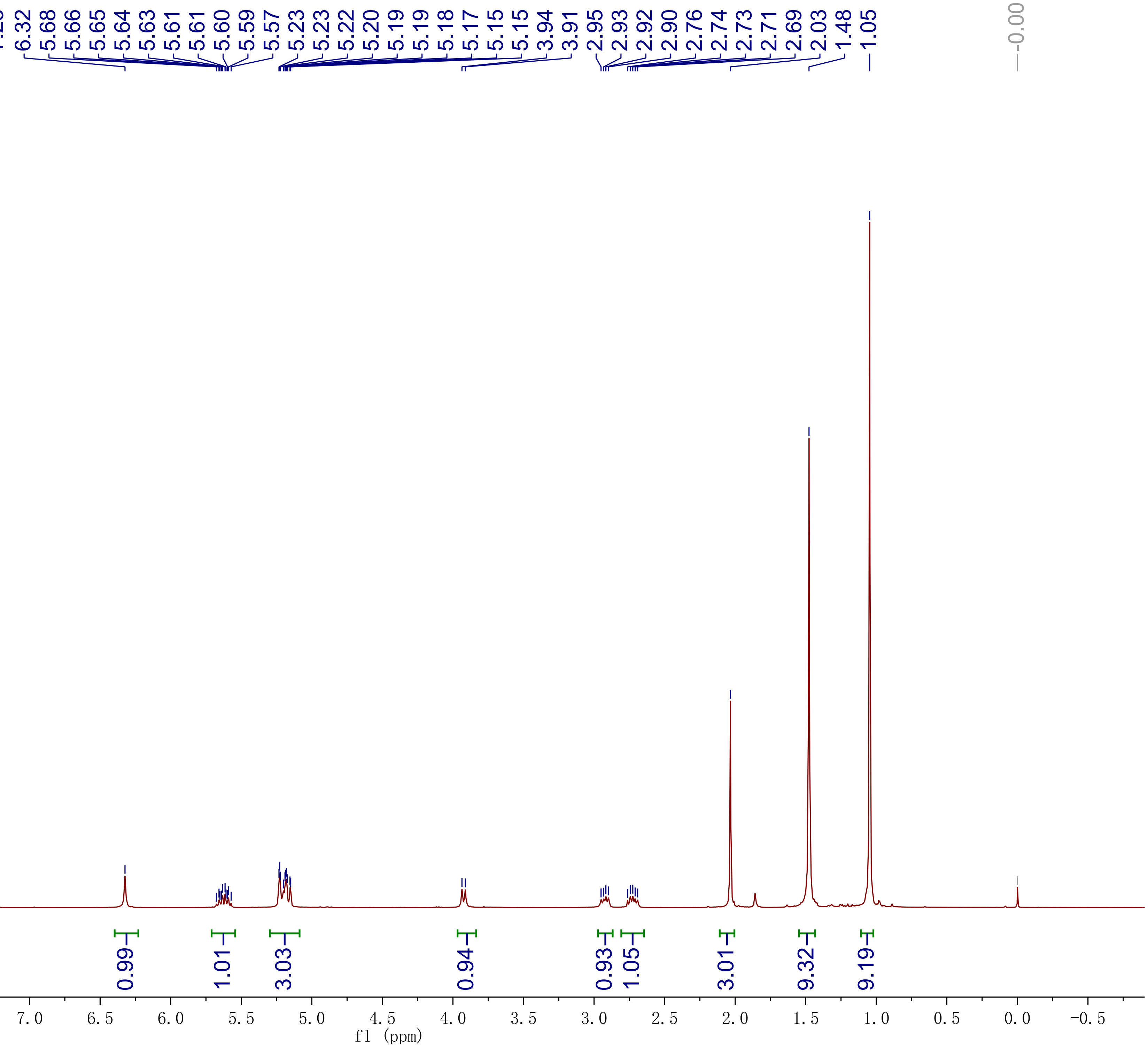

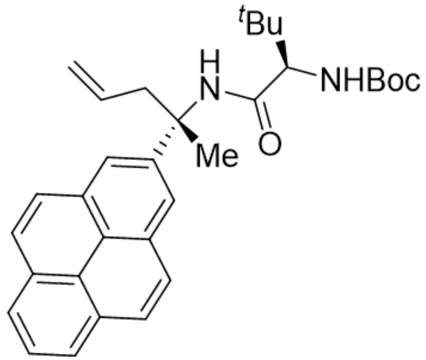

S4

^{13}C NMR (101 MHz, $CDCl_3$)

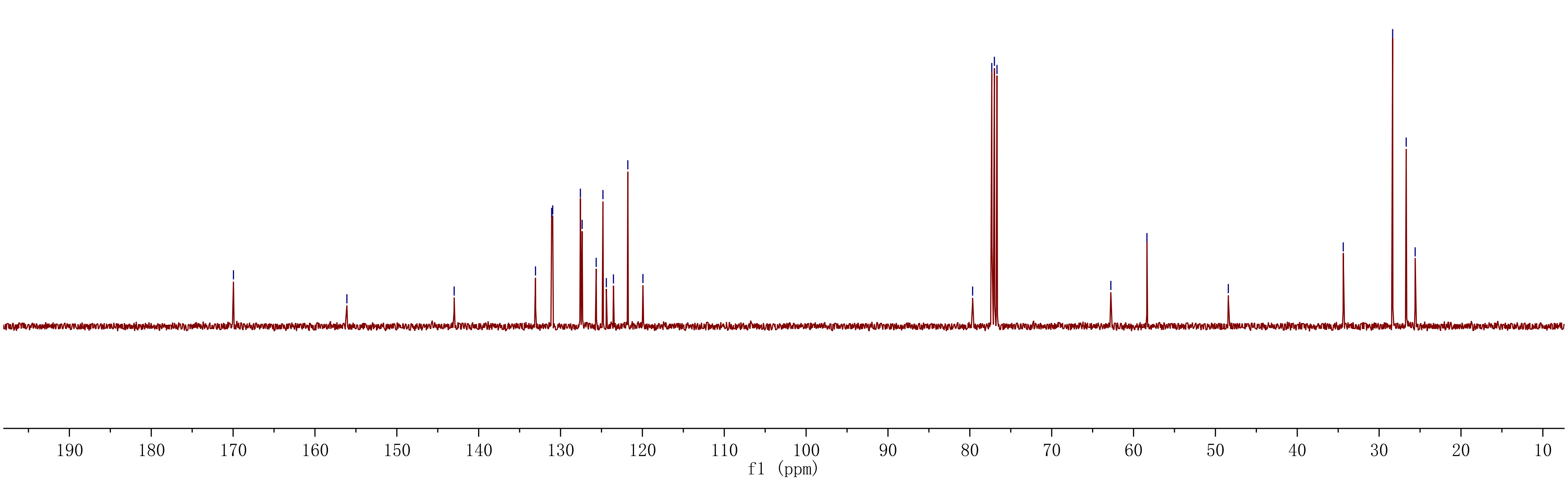

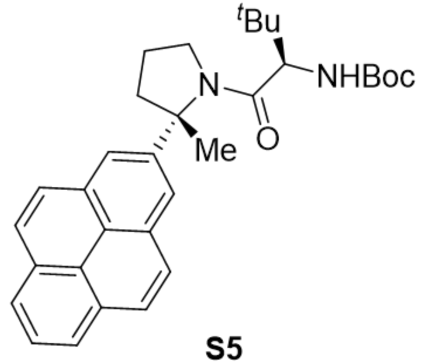

S5

^{1}H NMR (400 MHz, $CDCl_3$)

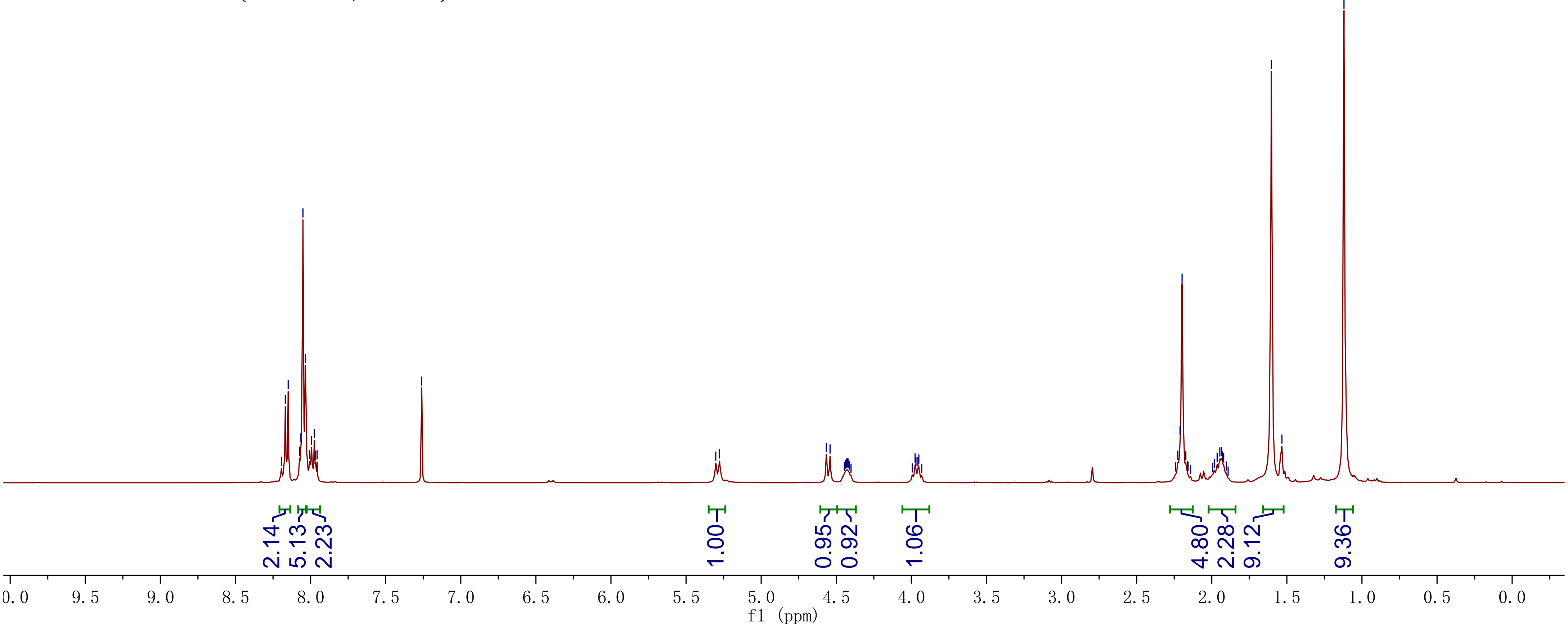

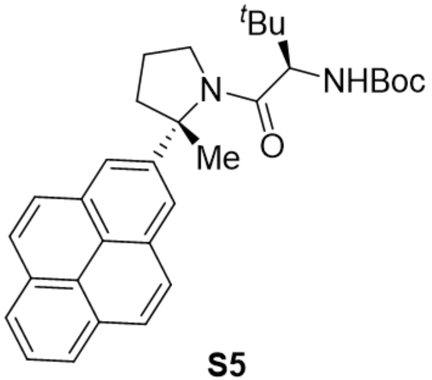

S5

^{13}C NMR (101 MHz, $CDCl_3$)

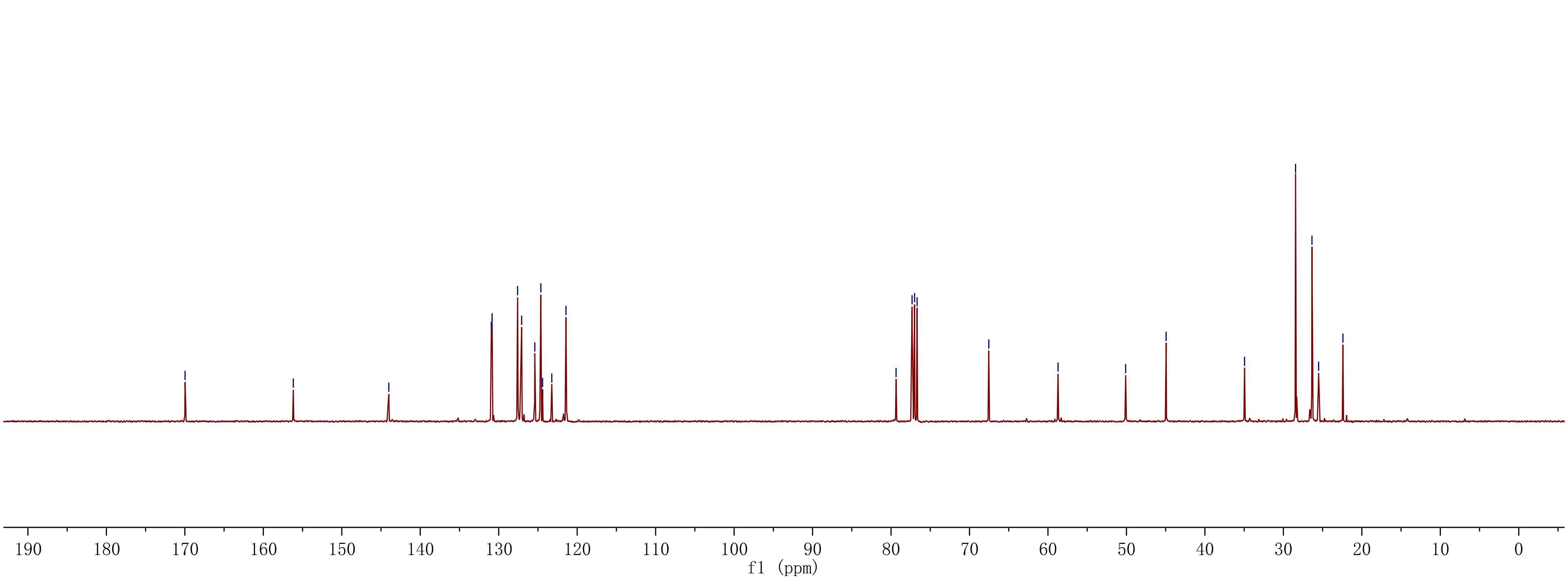

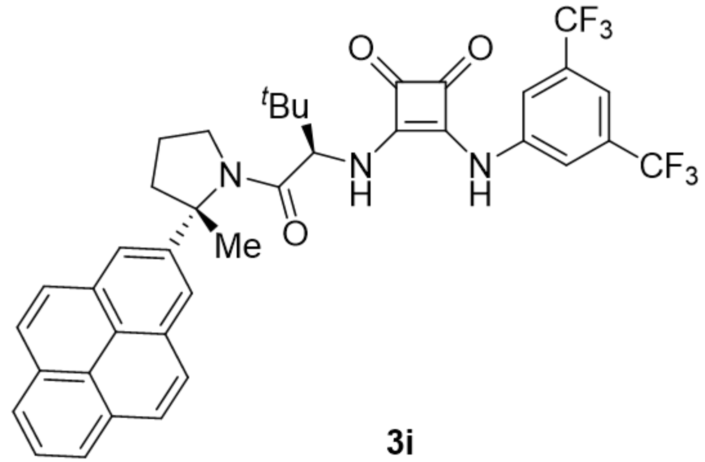

3i

^{1}H NMR (400 MHz, $CDCl_3$)

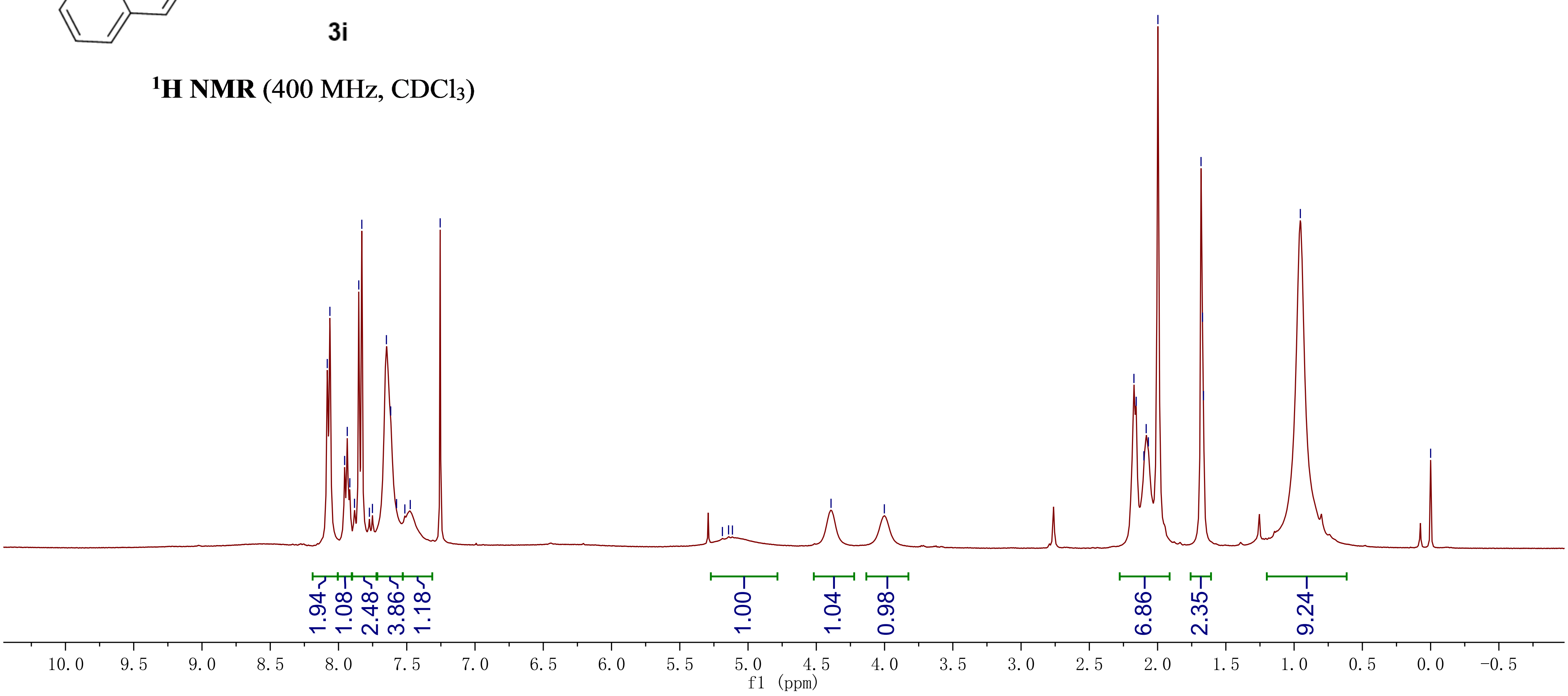

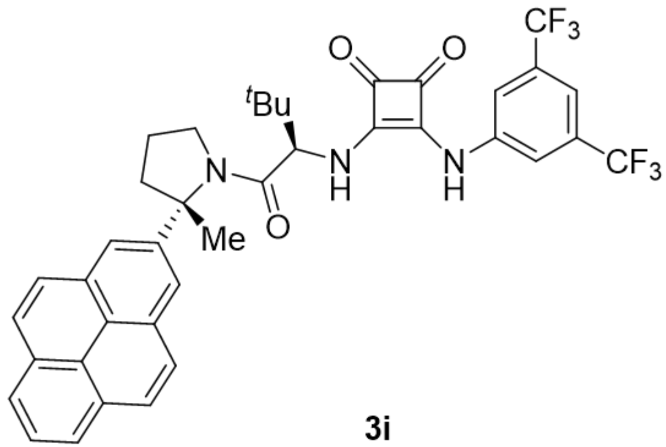

3i

^{13}C NMR (101 MHz, $CDCl_3$)

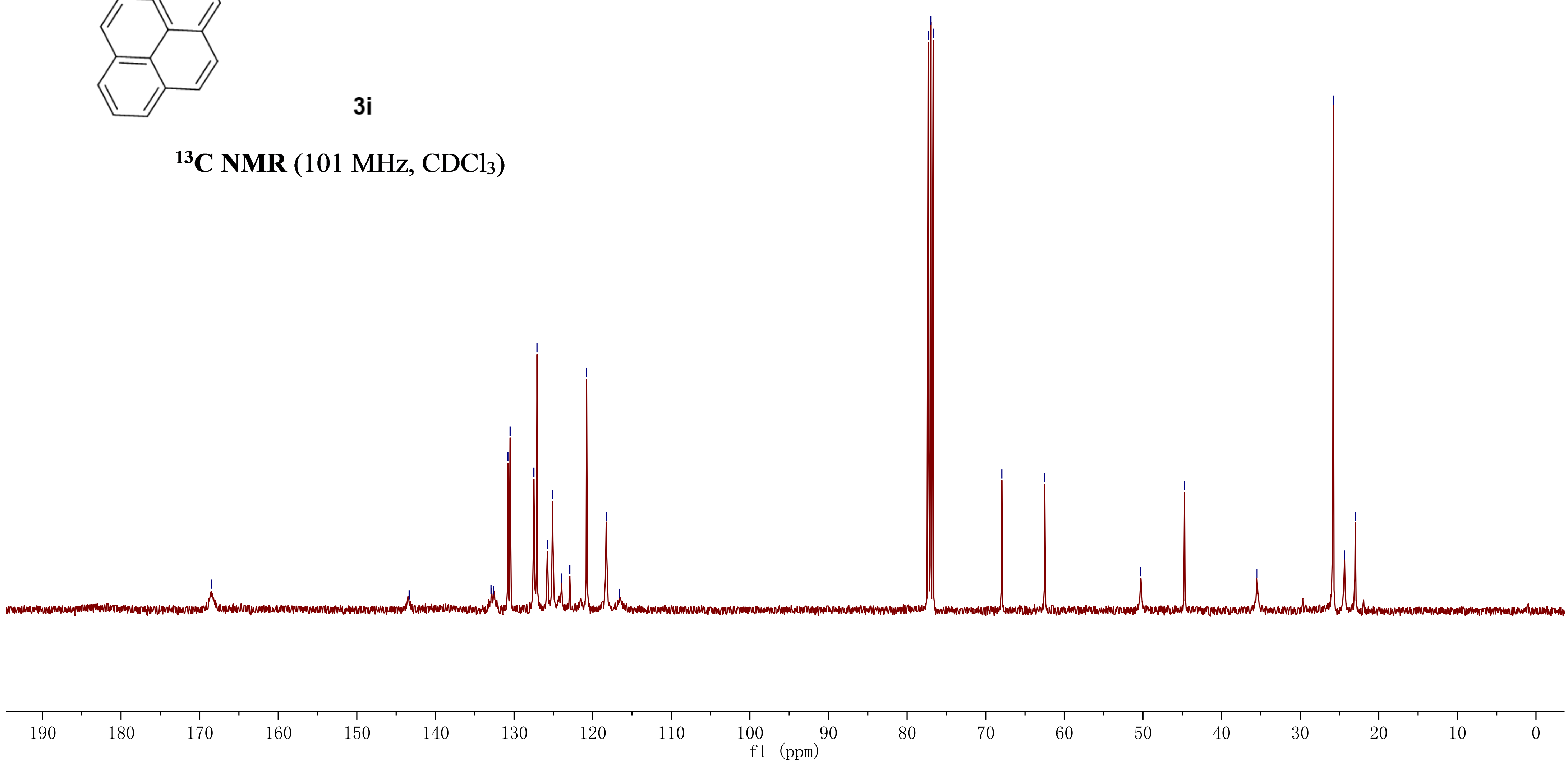

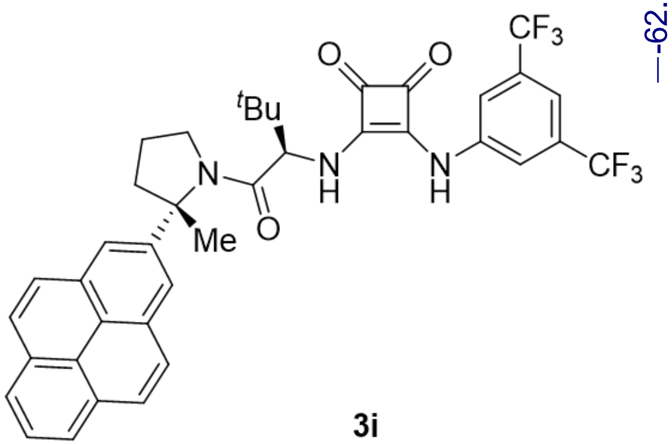

3i

^{19}F NMR (376 MHz, $CDCl_3$)

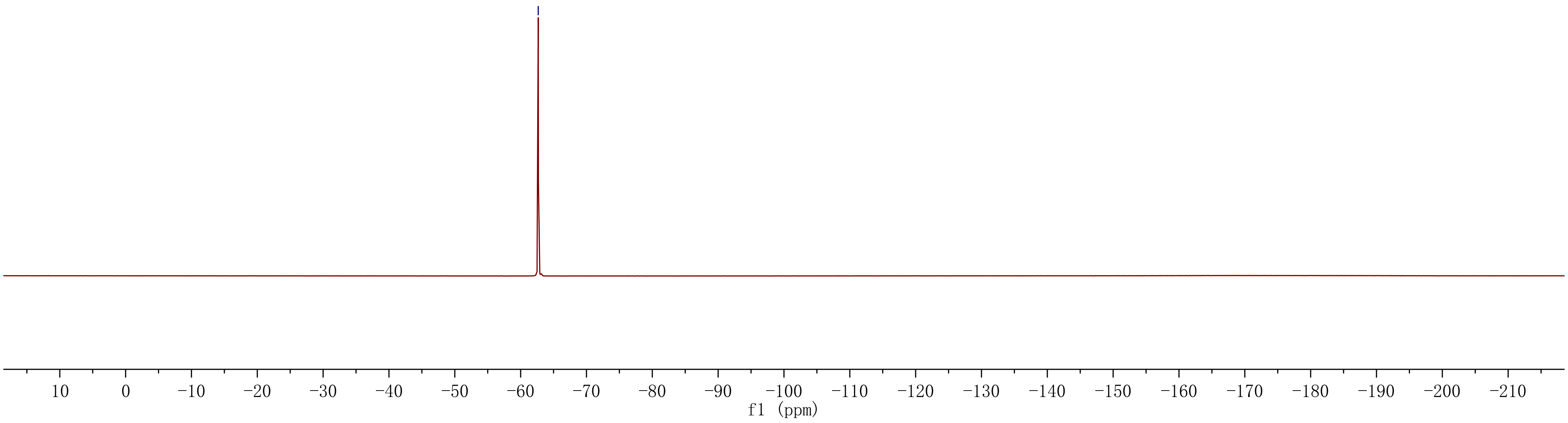

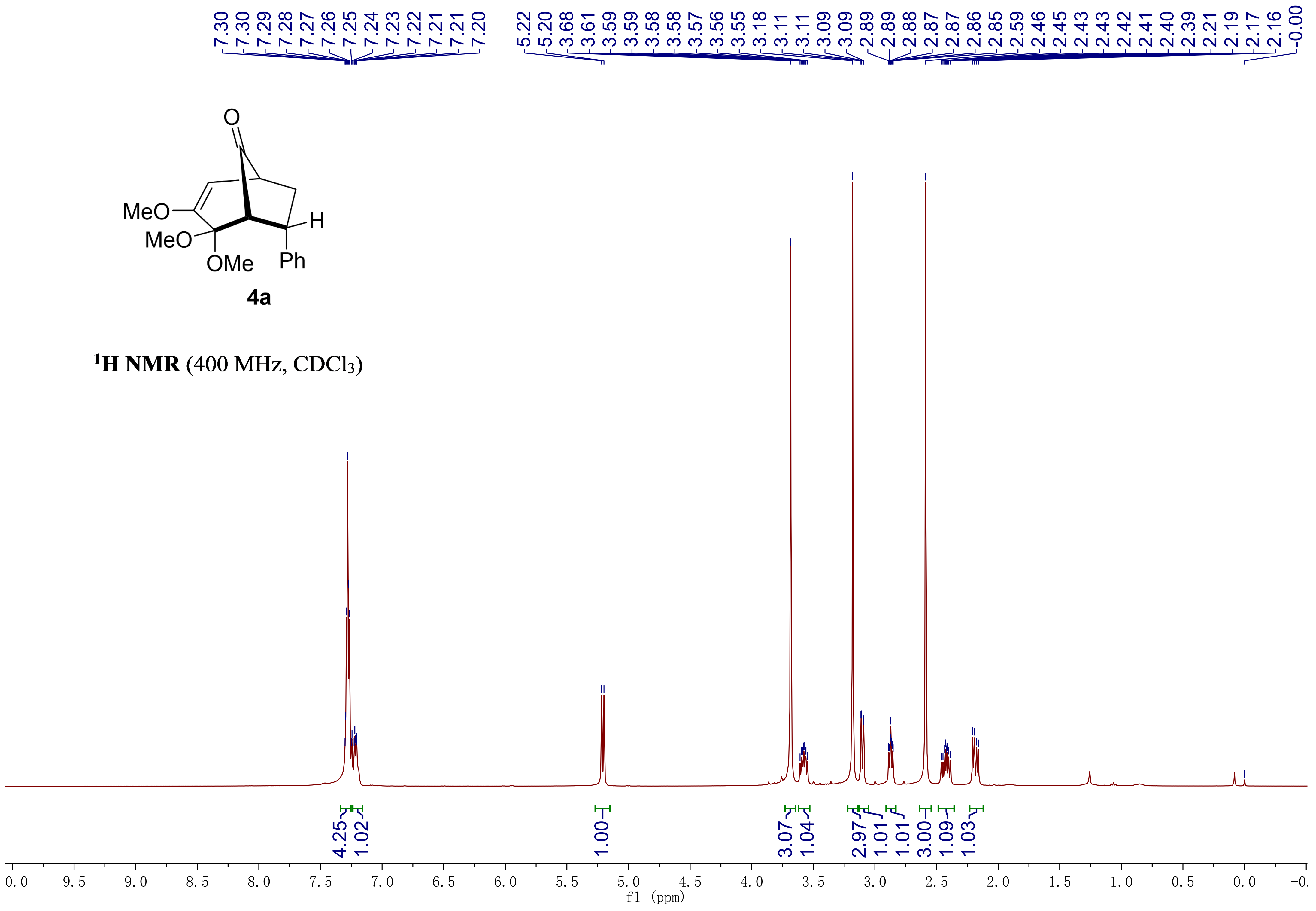

7.30 7.30 7.29 7.28 7.27 7.26 7.25 7.24 7.23 7.22 7.21 7.21 7.20
5.22 5.20 3.68 3.61 3.59 3.59 3.58 3.58 3.57 3.56 3.55 3.18 3.11 3.11 3.09 3.09 2.89 2.89 2.88 2.87 2.87 2.86 2.85 2.59 2.46 2.45 2.43 2.43 2.42 2.41 2.40 2.39 2.21 2.19 2.17 2.16 -0.00
O
MeO
MeO
OMe
H
Ph
4a
1H NMR (400 MHz, CDCl3)
4.25
1.02
1.00
3.07
1.04
2.97
1.01
1.01
3.00
1.09
1.03
f1 (ppm)

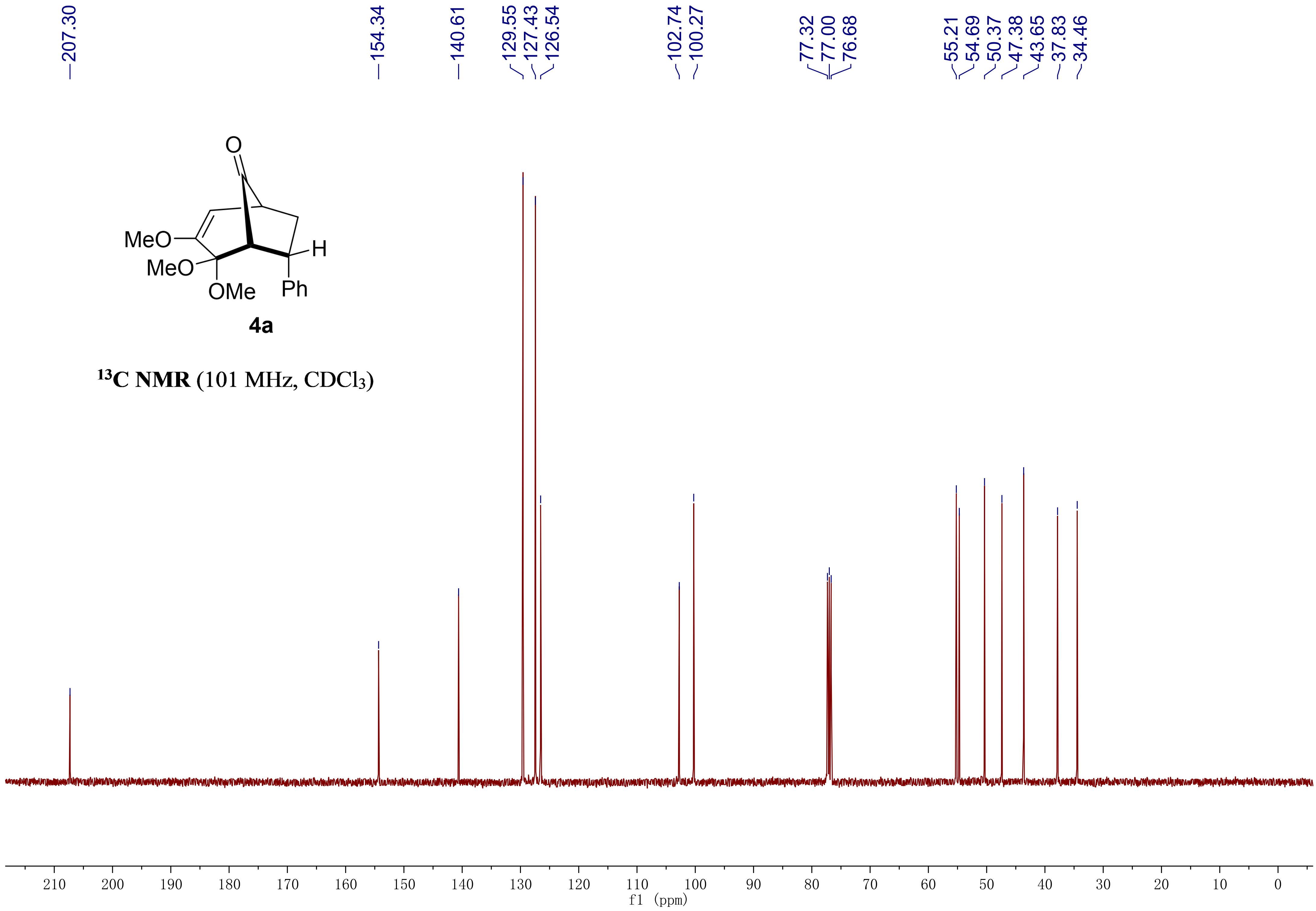

207.30
154.34
140.61
129.55
127.43
126.54
102.74
100.27
77.32
77.00
76.68
55.21
54.69
50.37
47.38
43.65
37.83
34.46
O
MeO
MeO
OMe
H
Ph
4a
13C NMR (101 MHz, CDCl3)
210 200 190 180 170 160 150 140 130 120 110 100 90 80 70 60 50 40 30 20 10 0
f1 (ppm)

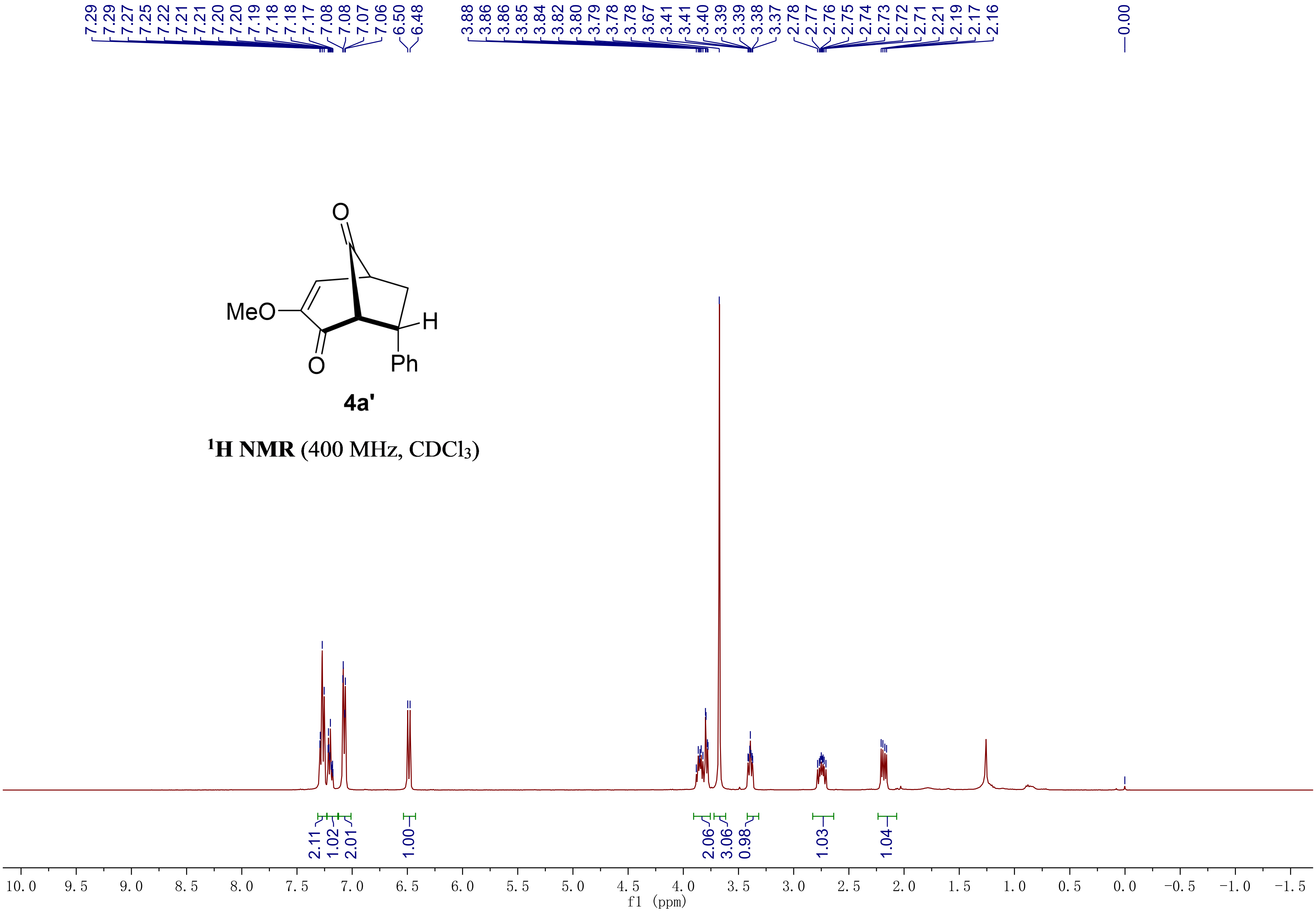

4a'

^{1}H NMR (400 MHz, $CDCl_3$)

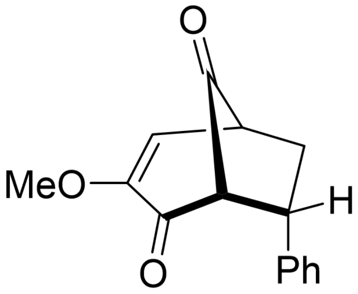

4a'

^{13}C NMR (101 MHz, $CDCl_3$)

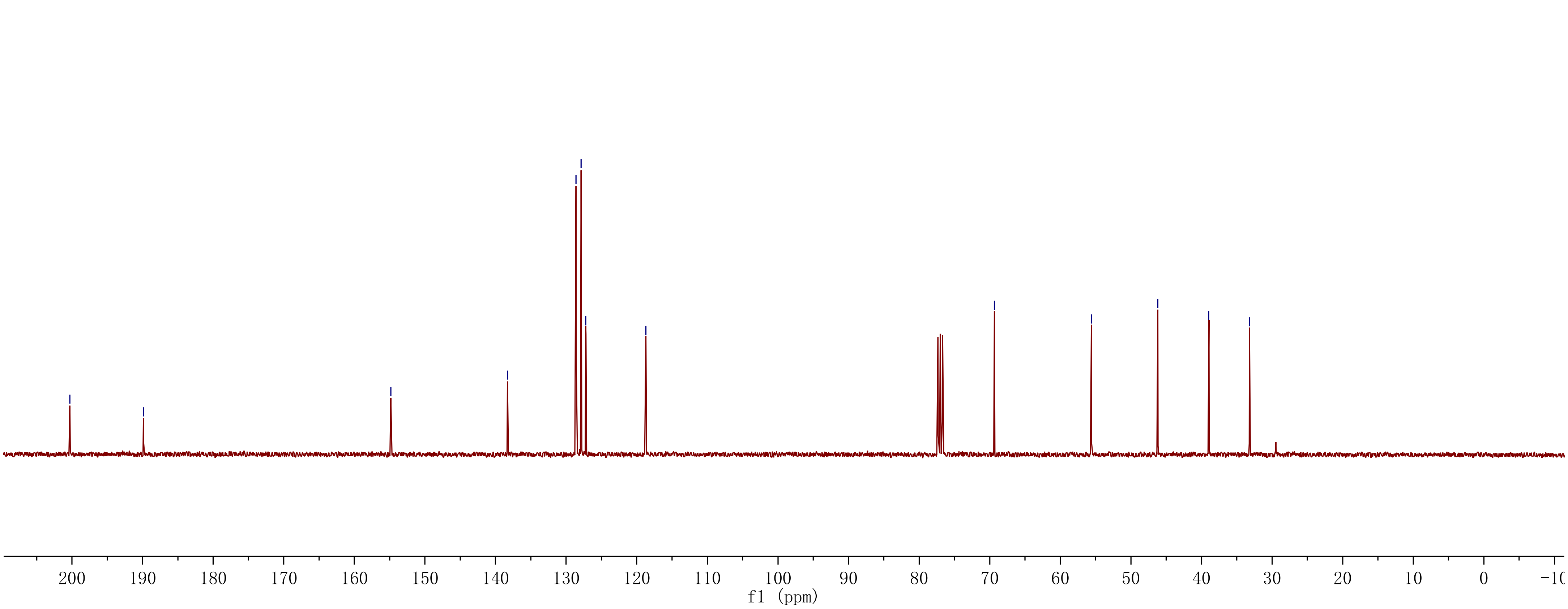

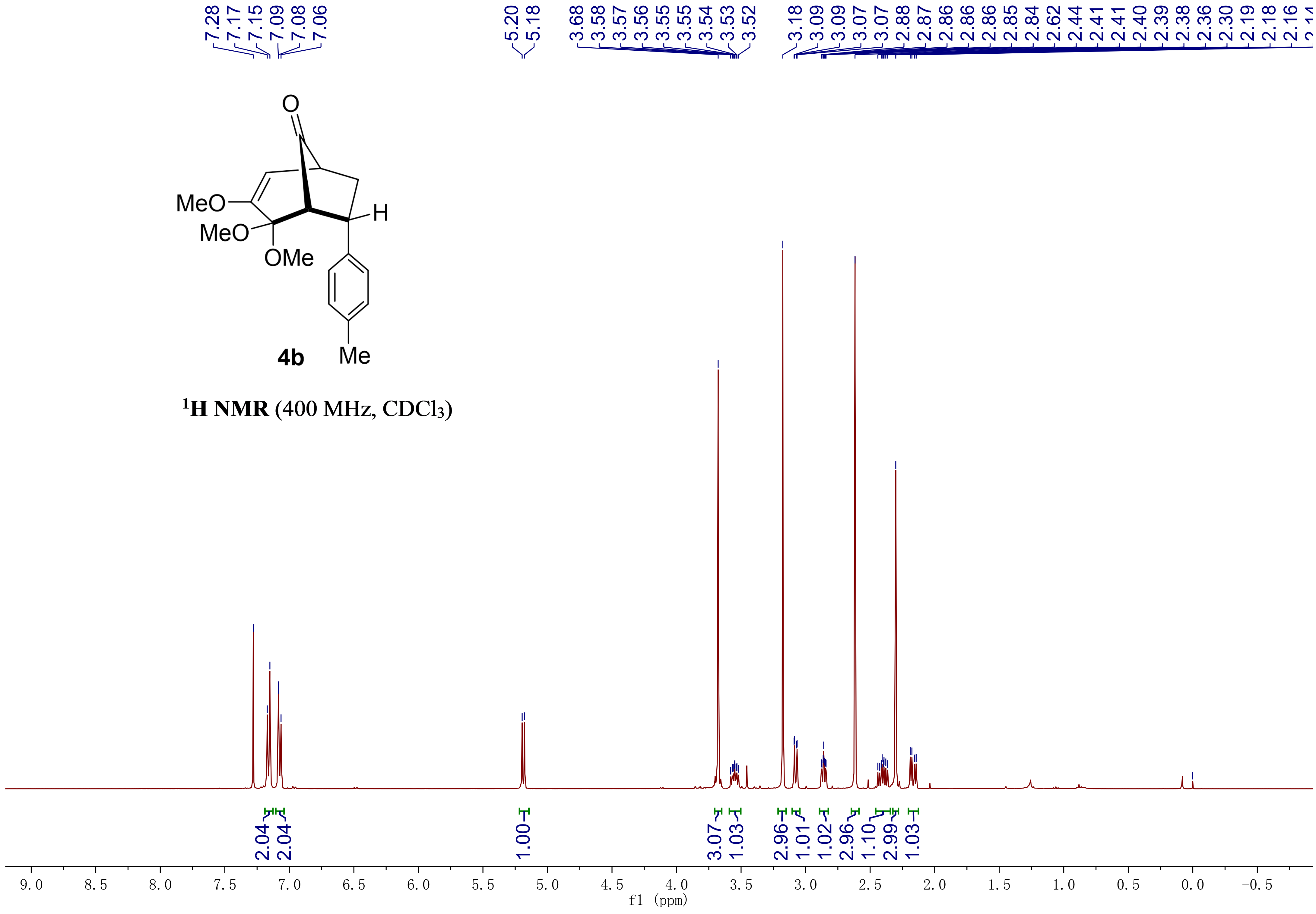

7.28 7.17 7.15 7.09 7.08 7.06
5.20 5.18
3.68 3.58 3.57 3.56 3.55 3.55 3.54 3.53 3.52
3.18 3.09 3.09 3.07 3.07 2.88 2.87 2.86 2.86 2.86 2.85 2.84 2.62 2.44 2.41 2.41 2.40 2.39 2.38 2.36 2.30 2.19 2.18 2.16
O
MeO
MeO
OMe
H
Me
4b
¹H NMR (400 MHz, CDCl3)
2.04 2.04 1.00 3.07 1.03 2.96 1.01 1.02 2.96 1.10 2.99 1.03
9.0 8.5 8.0 7.5 7.0 6.5 6.0 5.5 5.0 4.5 4.0 3.5 3.0 2.5 2.0 1.5 1.0 0.5 0.0 -0.5
f1 (ppm)

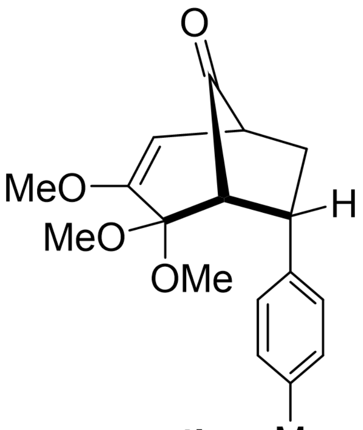

4b

^{13}C NMR (101 MHz, $CDCl_3$)

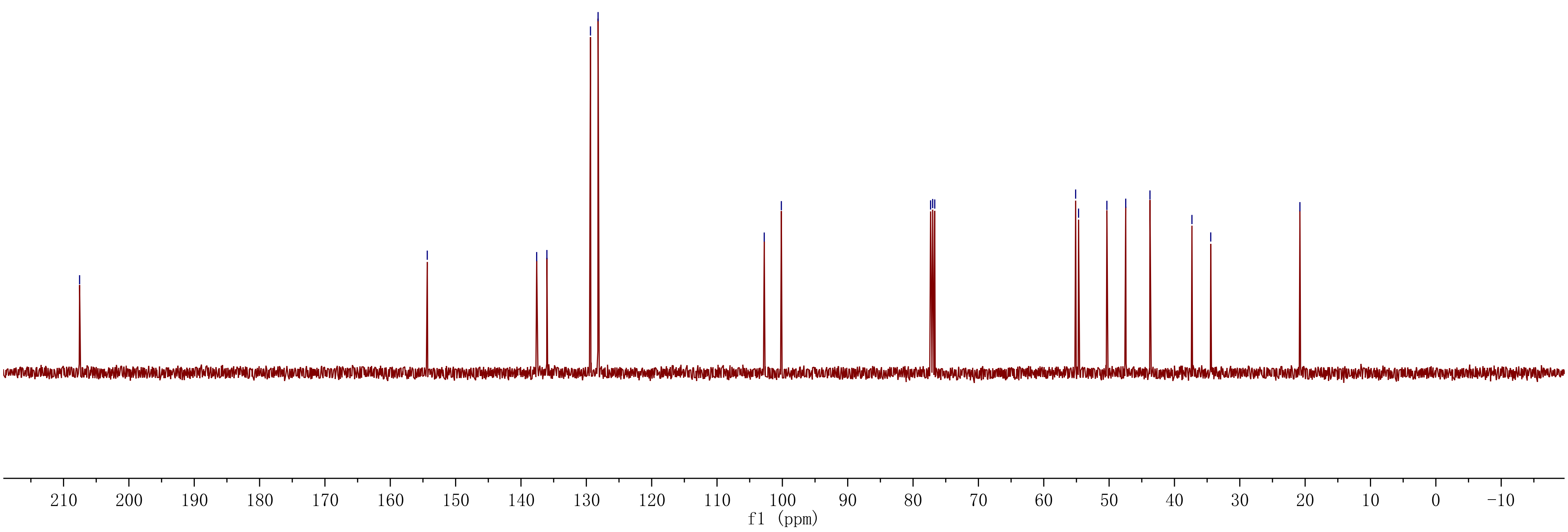

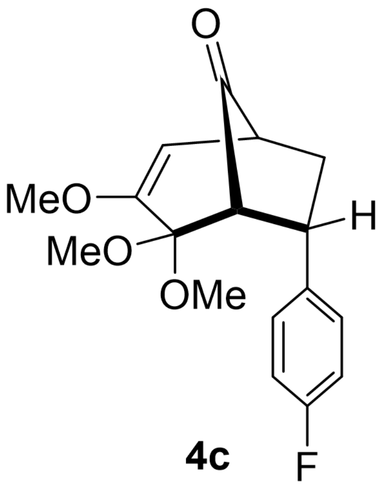

4c

^{1}H NMR (400 MHz, $CDCl_3$)

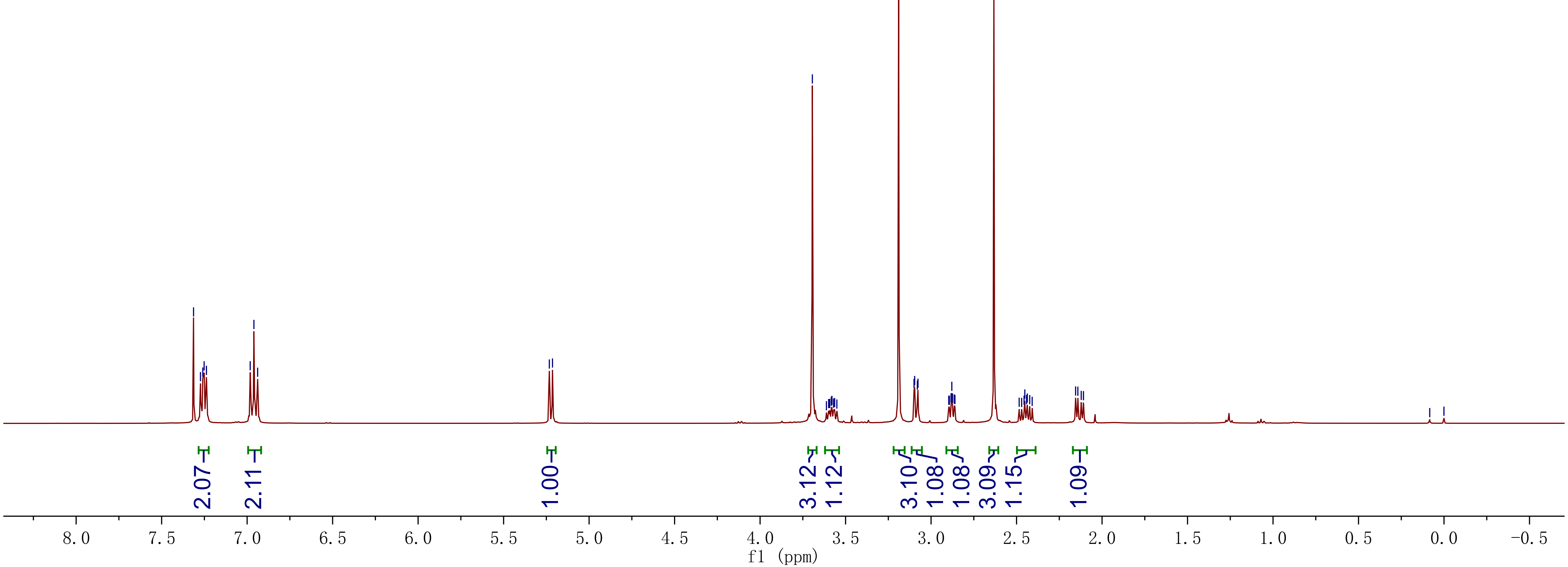

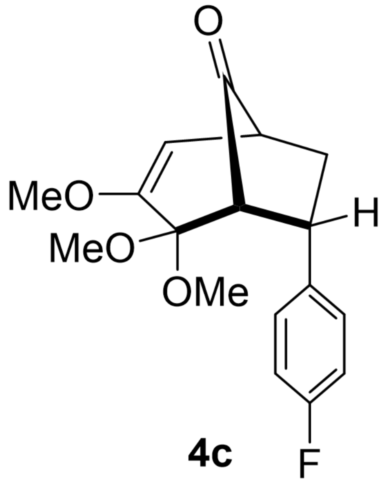

4c

^{13}C NMR (101 MHz, $CDCl_3$)

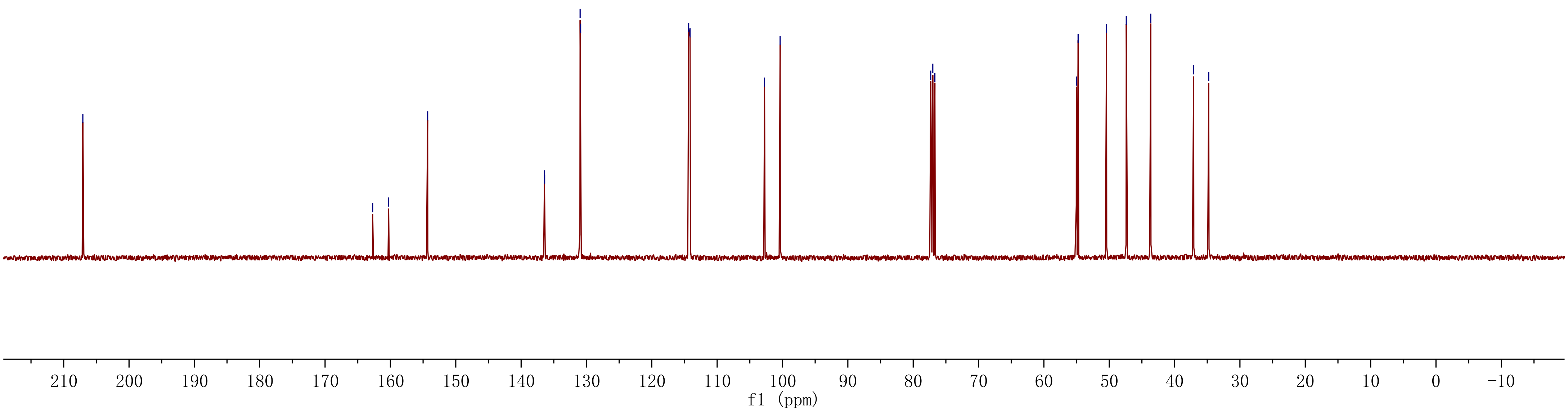

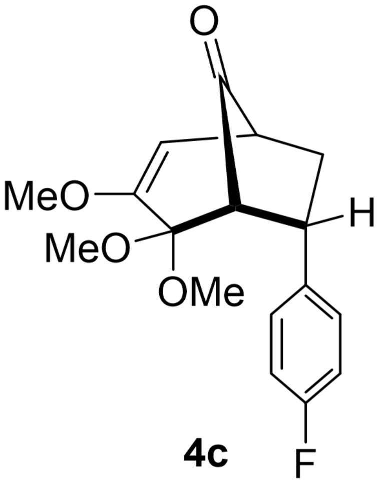

4c

^{19}F NMR (376 MHz, $CDCl_3$)

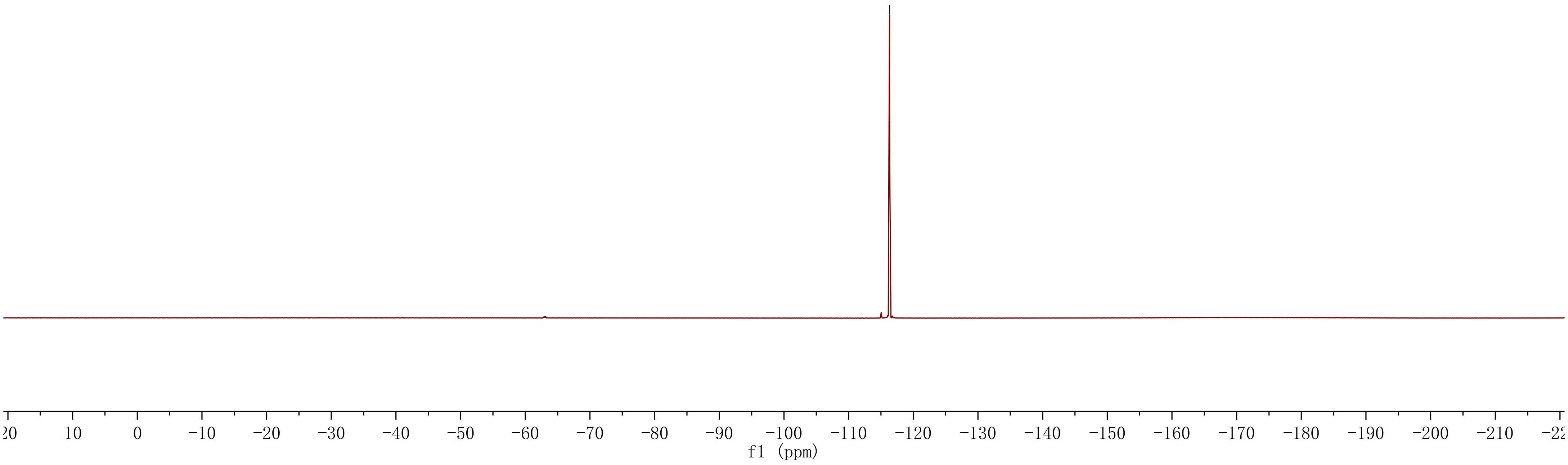

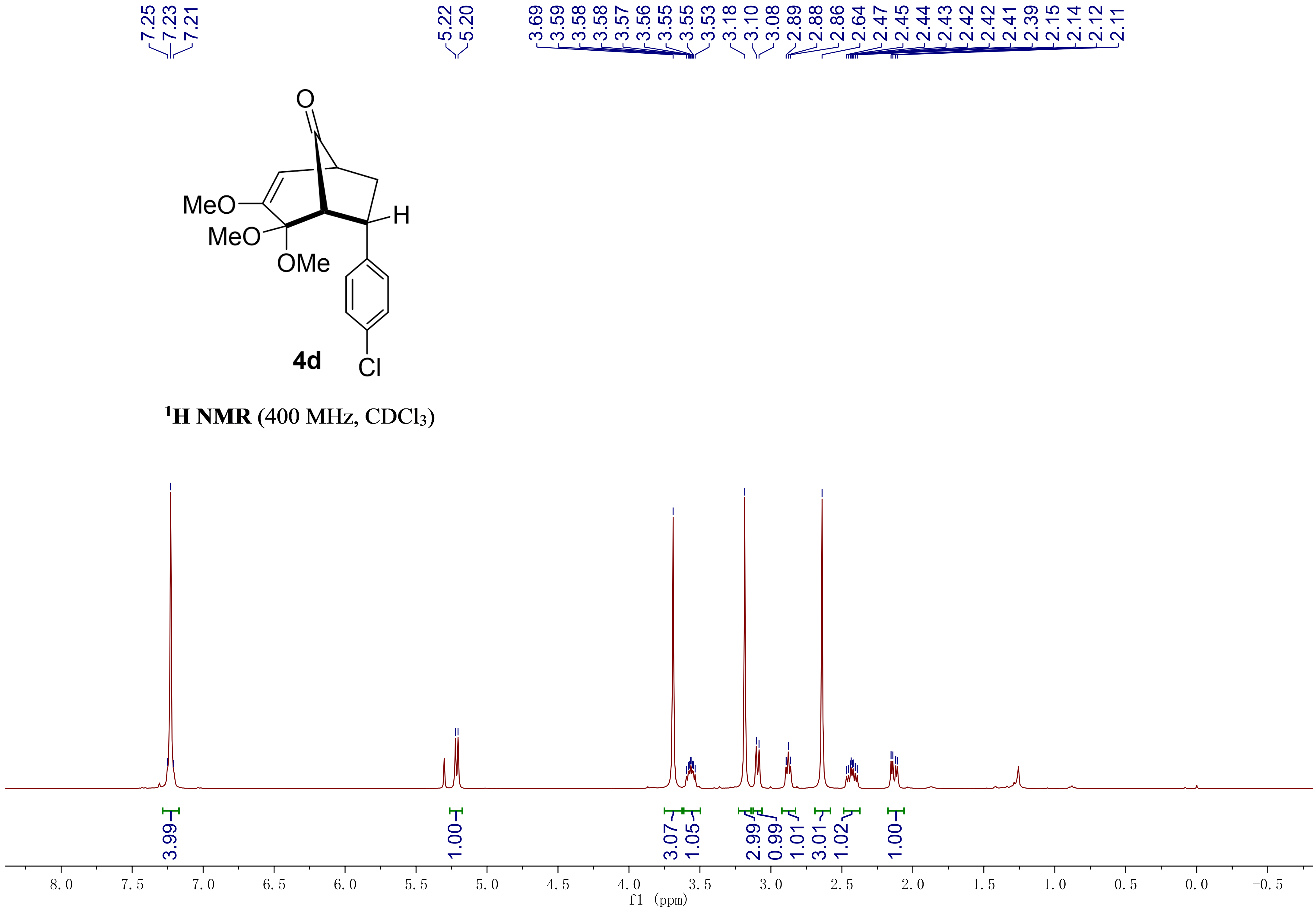
7.25
7.23
7.21
5.22
5.20
3.69
3.59
3.58
3.58
3.57
3.56
3.55
3.55
3.53
3.18
3.10
3.08
2.89
2.88
2.86
2.64
2.47
2.45
2.44
2.43
2.42
2.42
2.41
2.39
2.15
2.14
2.12
2.11
MeO
MeO
OMe
O
H
Cl
4d
1H NMR (400 MHz, CDCl3)
3.99
1.00
3.07
1.05
2.99
0.99
1.01
3.01
1.02
1.00
8.0
7.5
7.0
6.5
6.0
5.5
5.0
4.5
4.0
3.5
3.0
2.5
2.0
1.5
1.0
0.5
0.0
-0.5
f1 (ppm)

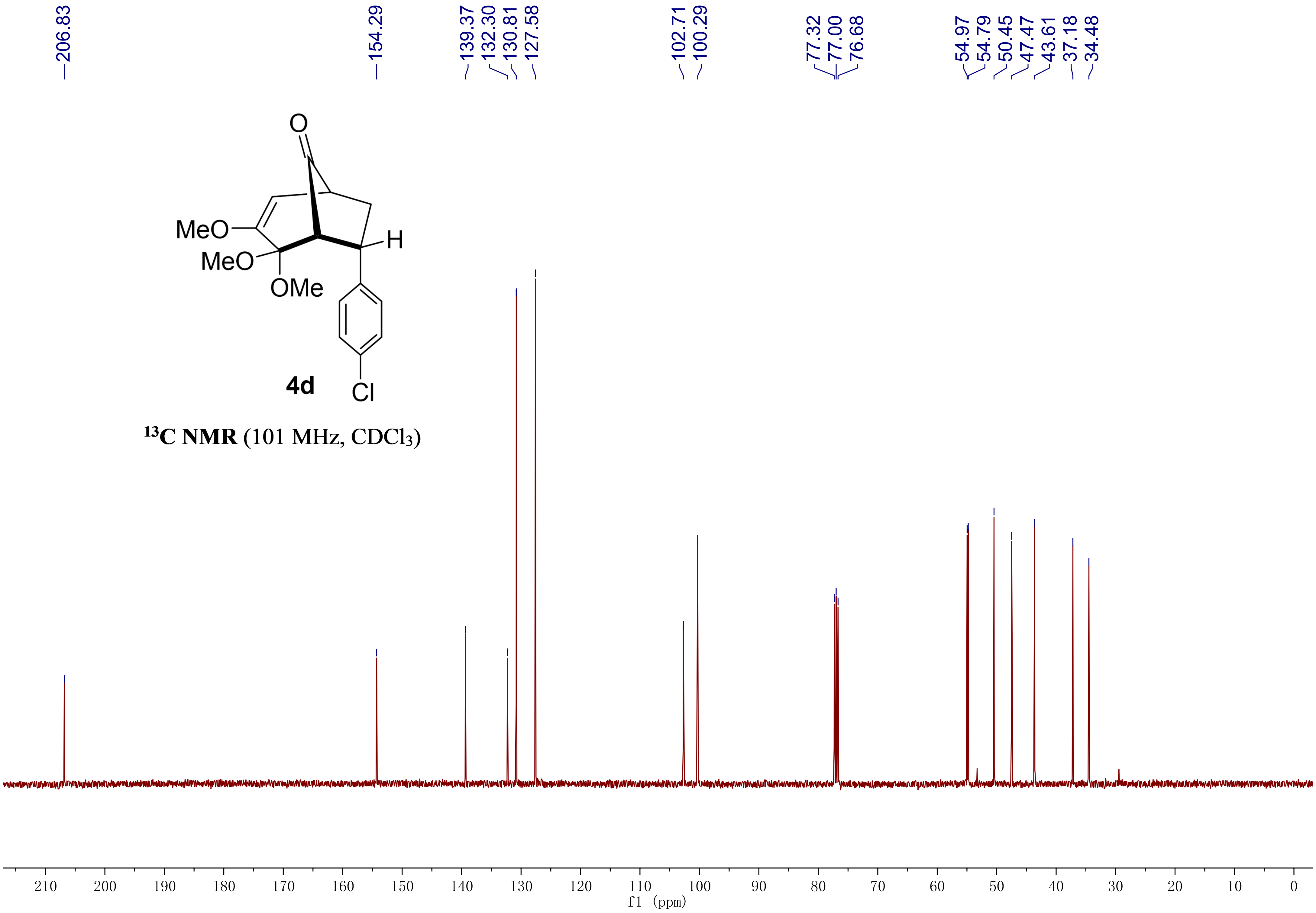
206.83
154.29
139.37
132.30
130.81
127.58
102.71
100.29
77.32
77.00
76.68
54.97
54.79
50.45
47.47
43.61
37.18
34.48
O
MeO
MeO
OMe
H
Cl
4d
13C NMR (101 MHz, CDCl3)
210 200 190 180 170 160 150 140 130 120 110 100 90 80 70 60 50 40 30 20 10 0
f1 (ppm)

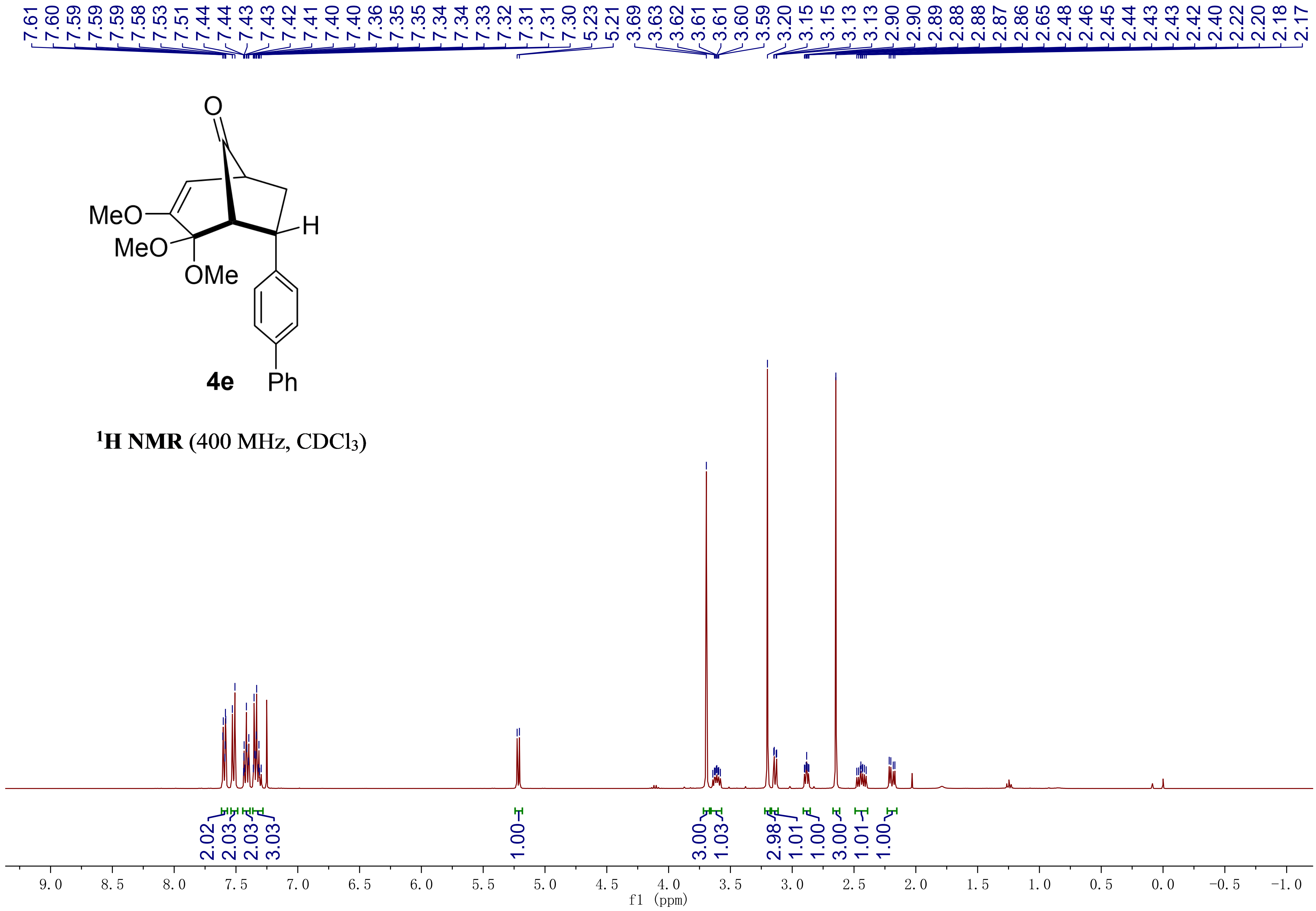
7.61 7.60 7.59 7.59 7.59 7.58 7.53 7.51 7.44 7.44 7.43 7.43 7.42 7.41 7.40 7.40 7.36 7.35 7.35 7.34 7.34 7.33 7.32 7.31 7.31 7.30 5.23 5.21 3.69 3.63 3.62 3.61 3.61 3.60 3.59 3.20 3.15 3.15 3.13 3.13 2.90 2.90 2.89 2.88 2.88 2.87 2.86 2.65 2.48 2.46 2.45 2.44 2.43 2.43 2.42 2.40 2.22 2.20 2.18 2.17
MeO
MeO
OMe
O
H
4e
Ph
¹H NMR (400 MHz, CDCl₃)
2.02 2.03 2.03 3.03 1.00 3.00 1.03 2.98 1.01 1.00 3.00 1.01 1.00
9.0 8.5 8.0 7.5 7.0 6.5 6.0 5.5 5.0 4.5 4.0 3.5 3.0 2.5 2.0 1.5 1.0 0.5 0.0 -0.5 -1.0
f1 (ppm)

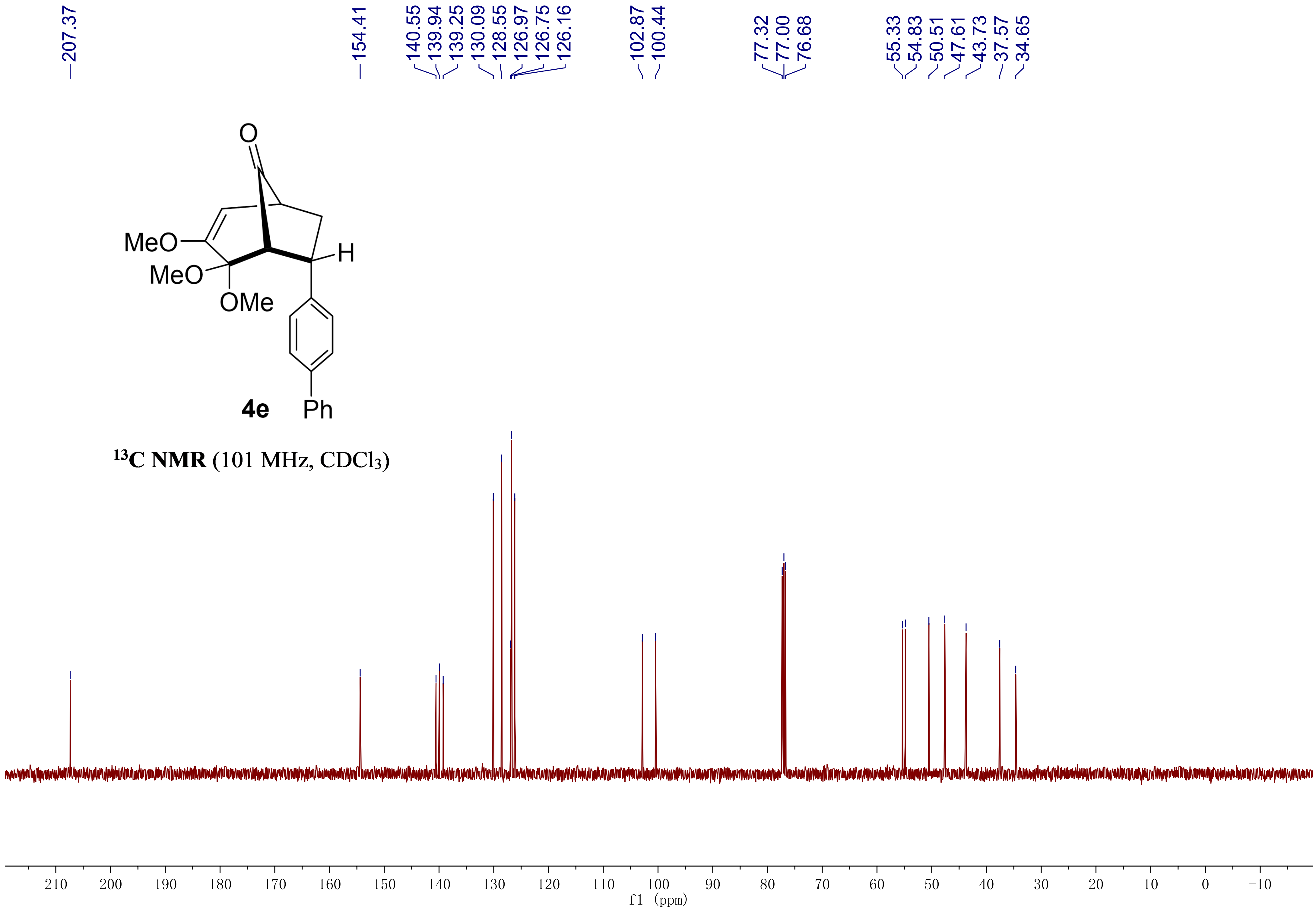

^{13}C NMR (101 MHz, $CDCl_3$)

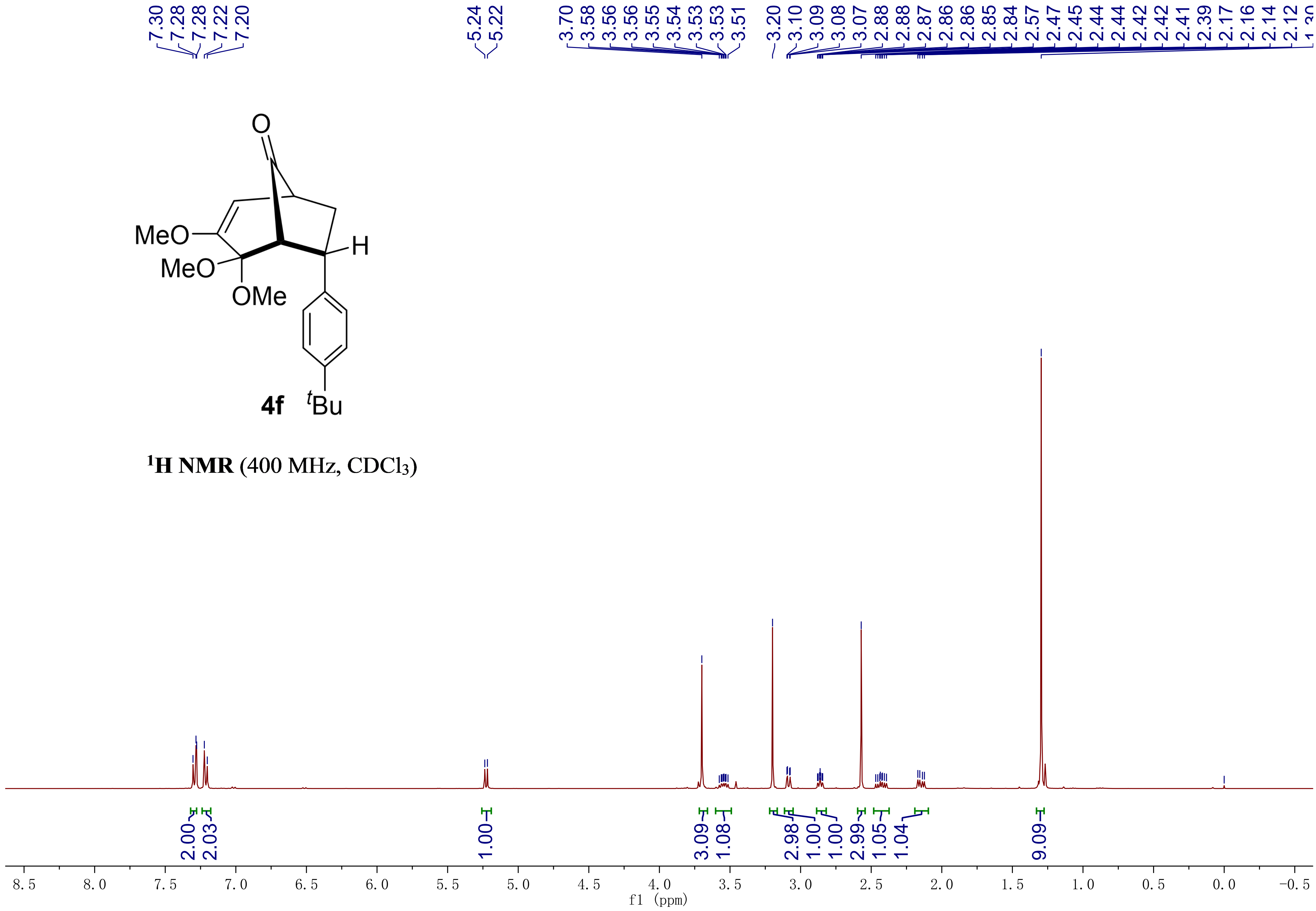

^{1}H NMR (400 MHz, $CDCl_3$)

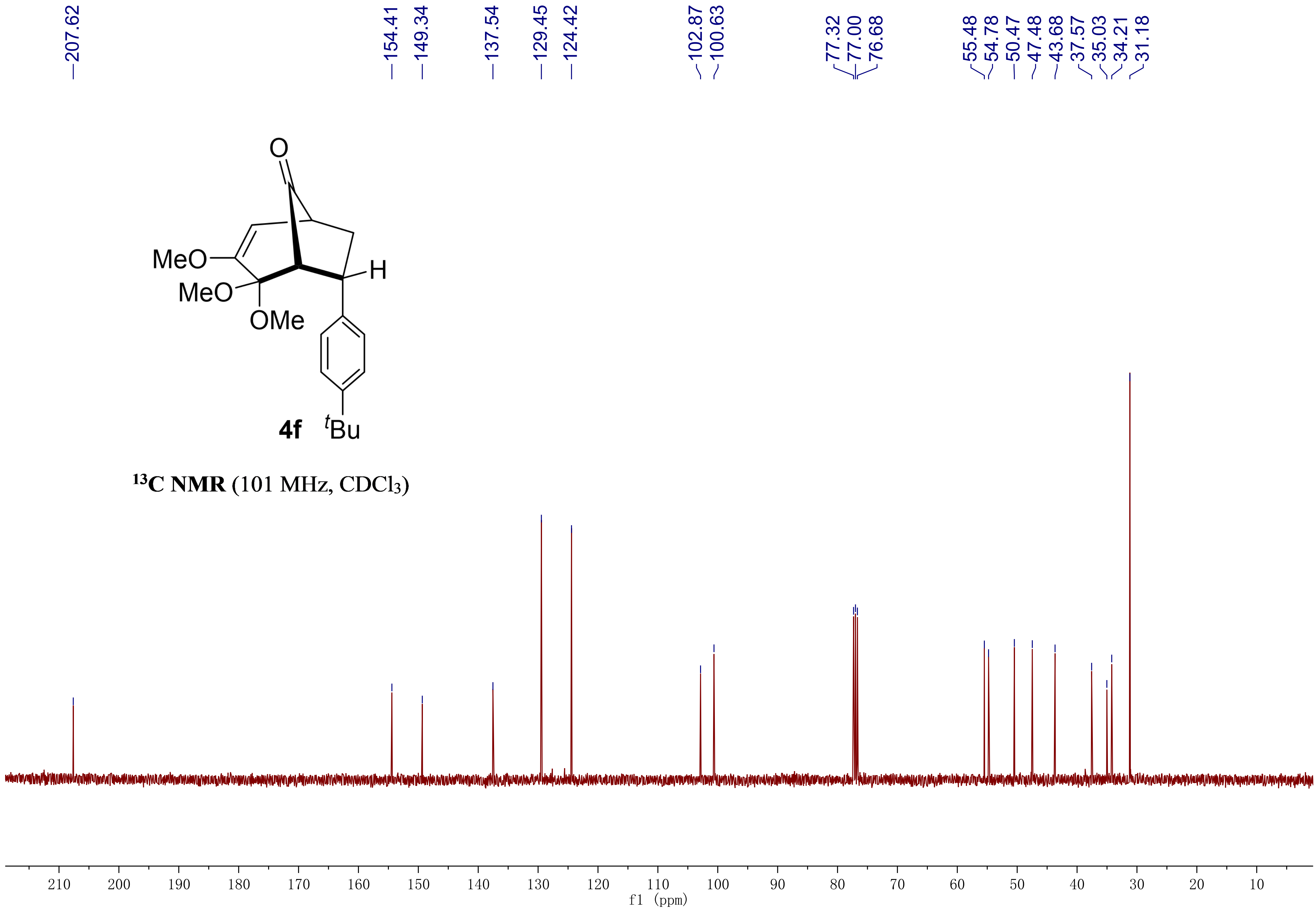
207.62
154.41
149.34
137.54
129.45
124.42
102.87
100.63
77.32
77.00
76.68
55.48
54.78
50.47
47.48
43.68
37.57
35.03
34.21
31.18
O
MeO
MeO
OMe
H
4f
tBu
13C NMR (101 MHz, CDCl3)
210 200 190 180 170 160 150 140 130 120 110 100 90 80 70 60 50 40 30 20 10
f1 (ppm)

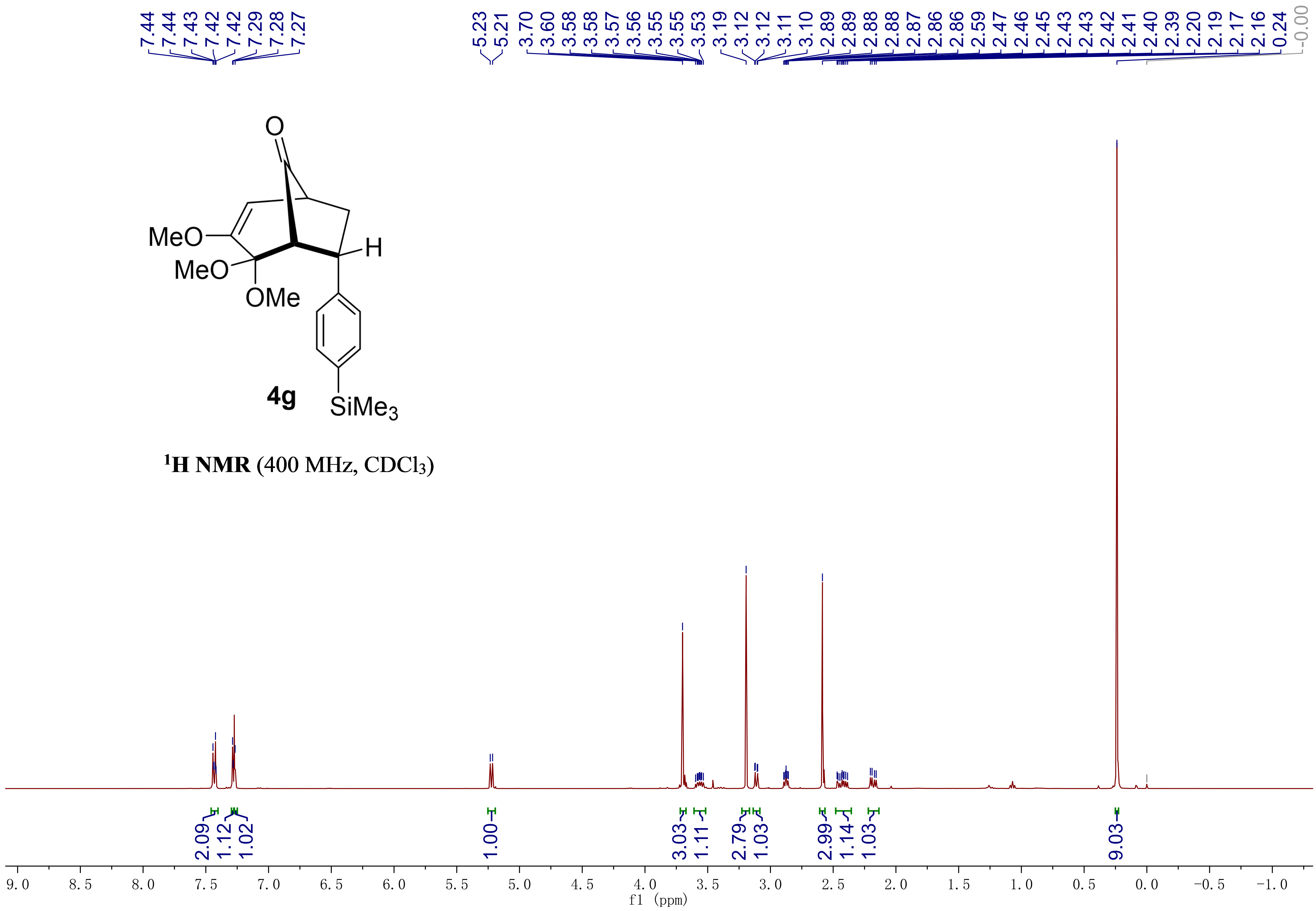
7.44 7.44 7.43 7.42 7.42 7.29 7.28 7.27
5.23 5.21 3.70 3.60 3.58 3.58 3.57 3.56 3.55 3.55 3.53 3.19 3.12 3.12 3.11 3.10 2.89 2.89 2.88 2.88 2.87 2.86 2.86 2.59 2.47 2.46 2.45 2.43 2.43 2.42 2.41 2.40 2.39 2.20 2.19 2.17 2.16 0.24 -0.00
MeO
MeO
OMe
O
H
SiMe3
4g
1H NMR (400 MHz, CDCl3)
2.09 1.12 1.02 1.00 3.03 1.11 2.79 1.03 2.99 1.14 1.03 9.03
9.0 8.5 8.0 7.5 7.0 6.5 6.0 5.5 5.0 4.5 4.0 3.5 3.0 2.5 2.0 1.5 1.0 0.5 0.0 -0.5 -1.0
f1 (ppm)

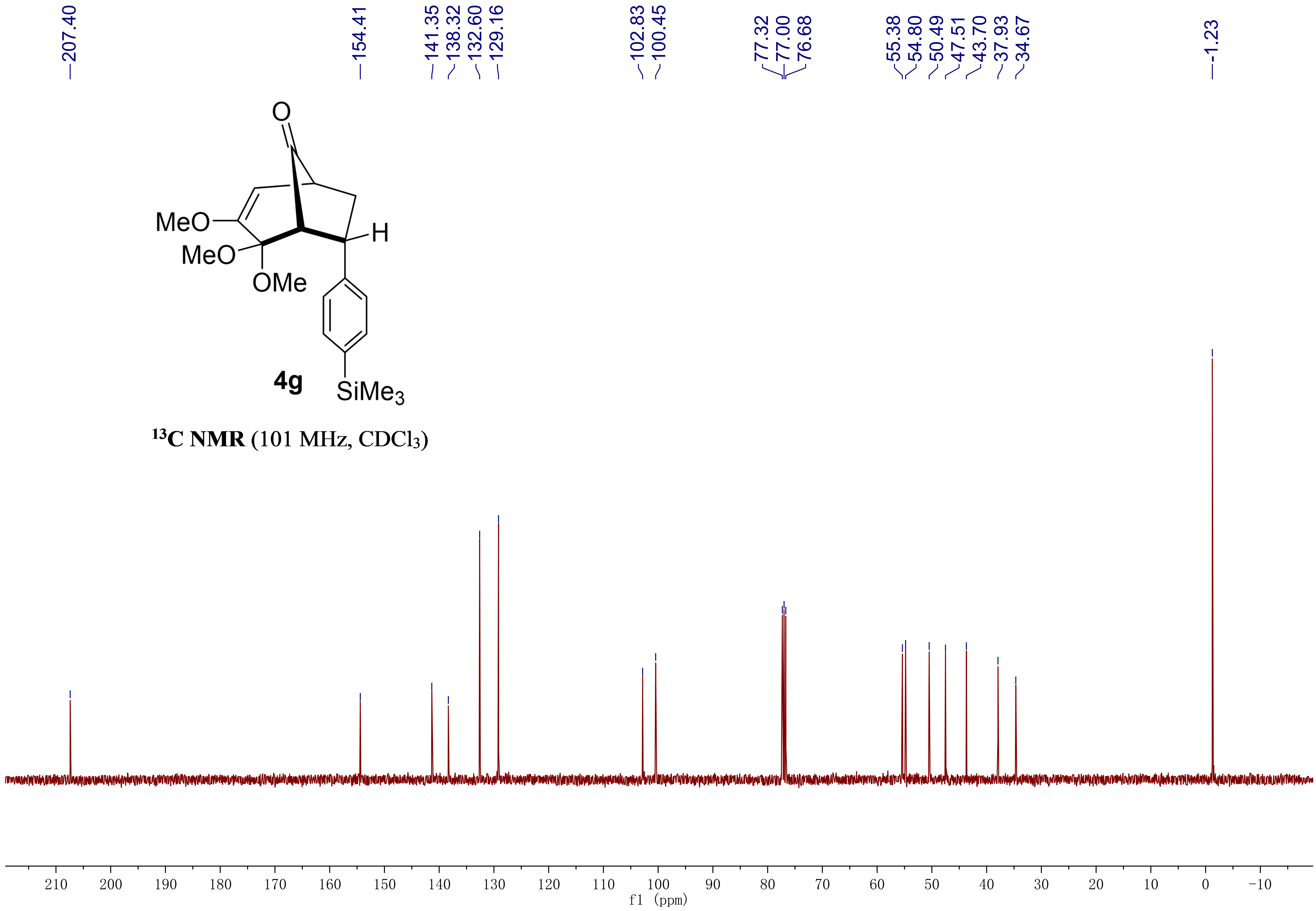

^{13}C NMR (101 MHz, $CDCl_3$)

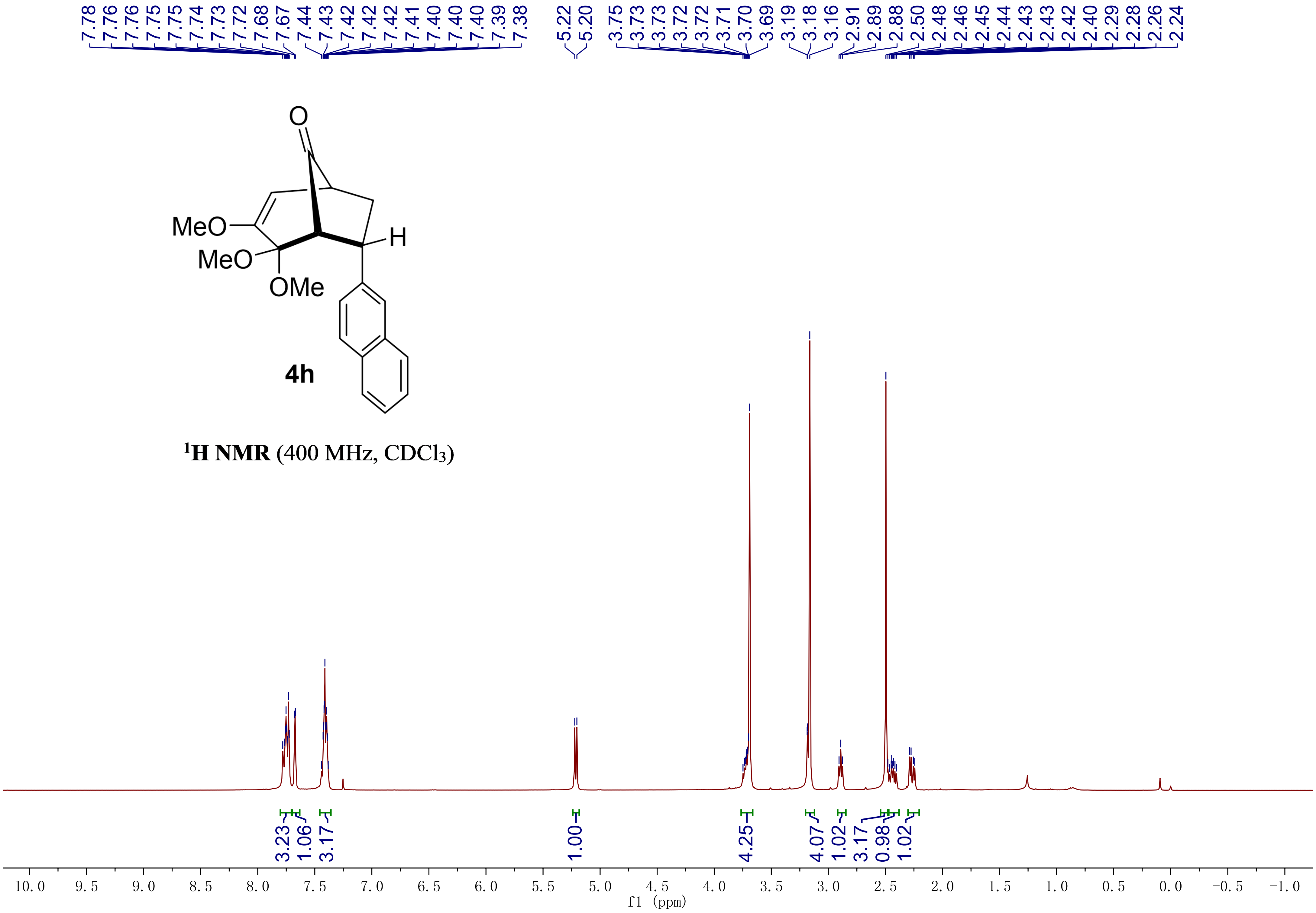
7.78 7.76 7.76 7.75 7.75 7.74 7.73 7.72 7.68 7.67 7.44 7.43 7.42 7.42 7.42 7.41 7.40 7.40 7.40 7.39 7.38
5.22 5.20
3.75 3.73 3.73 3.72 3.72 3.71 3.70 3.69 3.19 3.18 3.16 2.91 2.89 2.88 2.50 2.48 2.46 2.45 2.44 2.43 2.43 2.42 2.40 2.29 2.28 2.26 2.24
O
MeO
MeO
OMe
H
4h
1H NMR (400 MHz, CDCl3)
3.23
1.06
3.17
1.00
4.25
4.07
1.02
3.17
0.98
1.02
10.0 9.5 9.0 8.5 8.0 7.5 7.0 6.5 6.0 5.5 5.0 4.5 4.0 3.5 3.0 2.5 2.0 1.5 1.0 0.5 0.0 -0.5 -1.0
f1 (ppm)

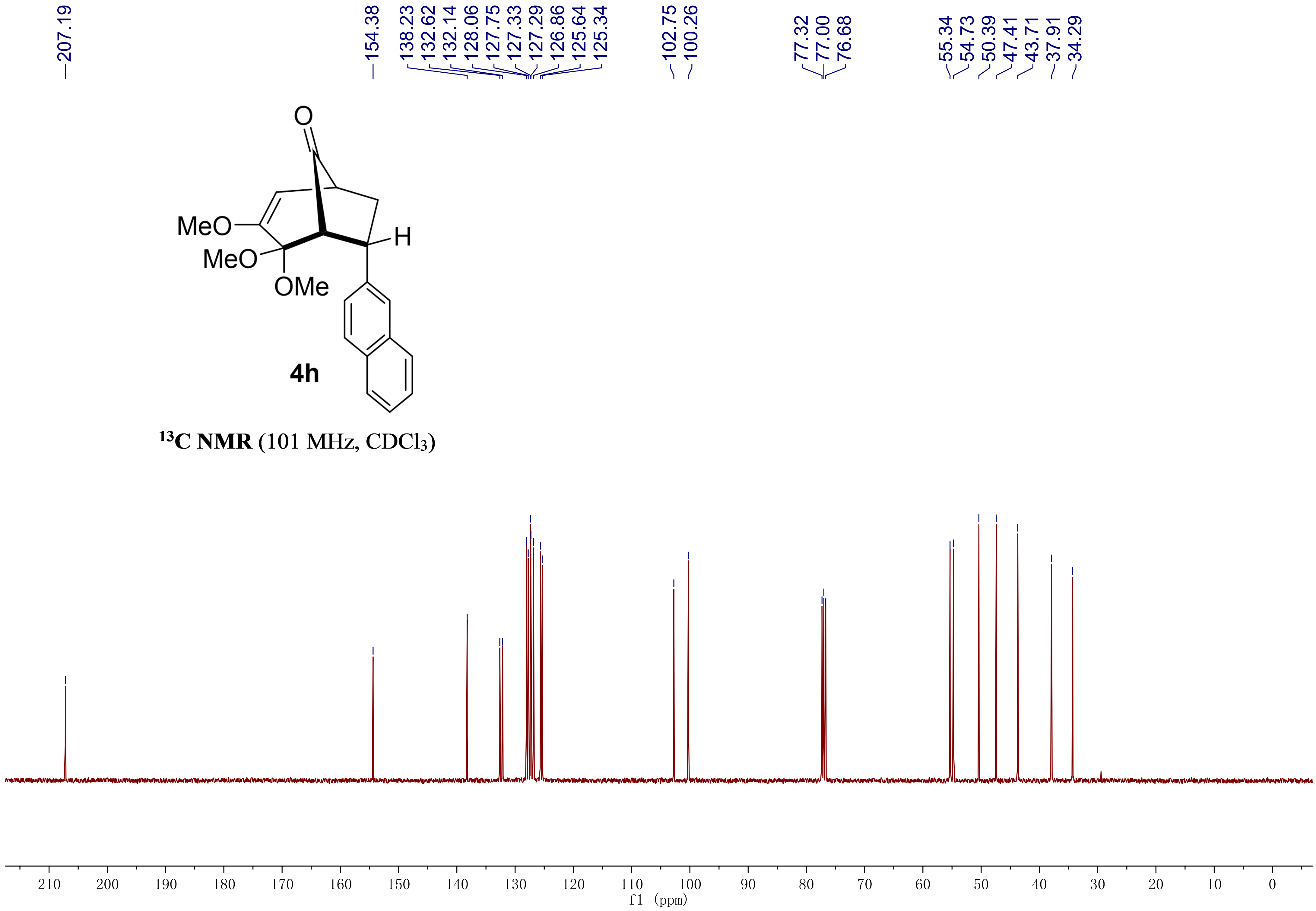

^{13}**C NMR** (101 MHz, $CDCl_3$)

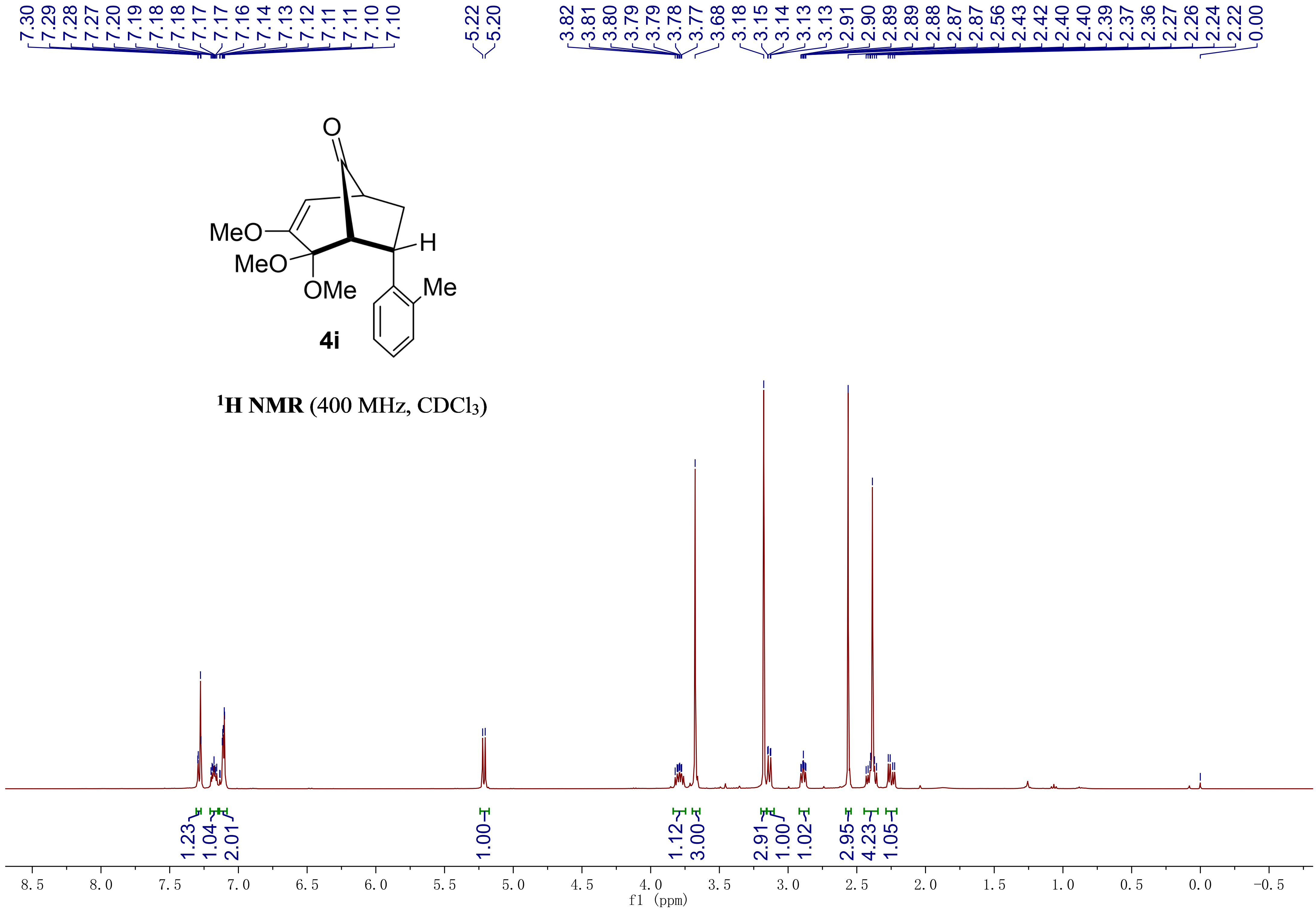
7.30 7.29 7.28 7.27 7.20 7.19 7.18 7.18 7.17 7.17 7.16 7.14 7.13 7.12 7.11 7.11 7.10 7.10
5.22 5.20
3.82 3.81 3.80 3.79 3.79 3.78 3.77 3.68 3.18 3.15 3.14 3.13 3.13 2.91 2.90 2.89 2.89 2.88 2.87 2.87 2.56 2.43 2.42 2.40 2.40 2.39 2.37 2.36 2.27 2.26 2.24 2.22 0.00
O
MeO
MeO
OMe
H
Me
4i
¹H NMR (400 MHz, CDCl₃)
1.23 1.04 2.01
1.00
1.12 3.00
2.91 1.00 1.02
2.95 4.23 1.05
8.5 8.0 7.5 7.0 6.5 6.0 5.5 5.0 4.5 4.0 3.5 3.0 2.5 2.0 1.5 1.0 0.5 0.0 -0.5
f1 (ppm)

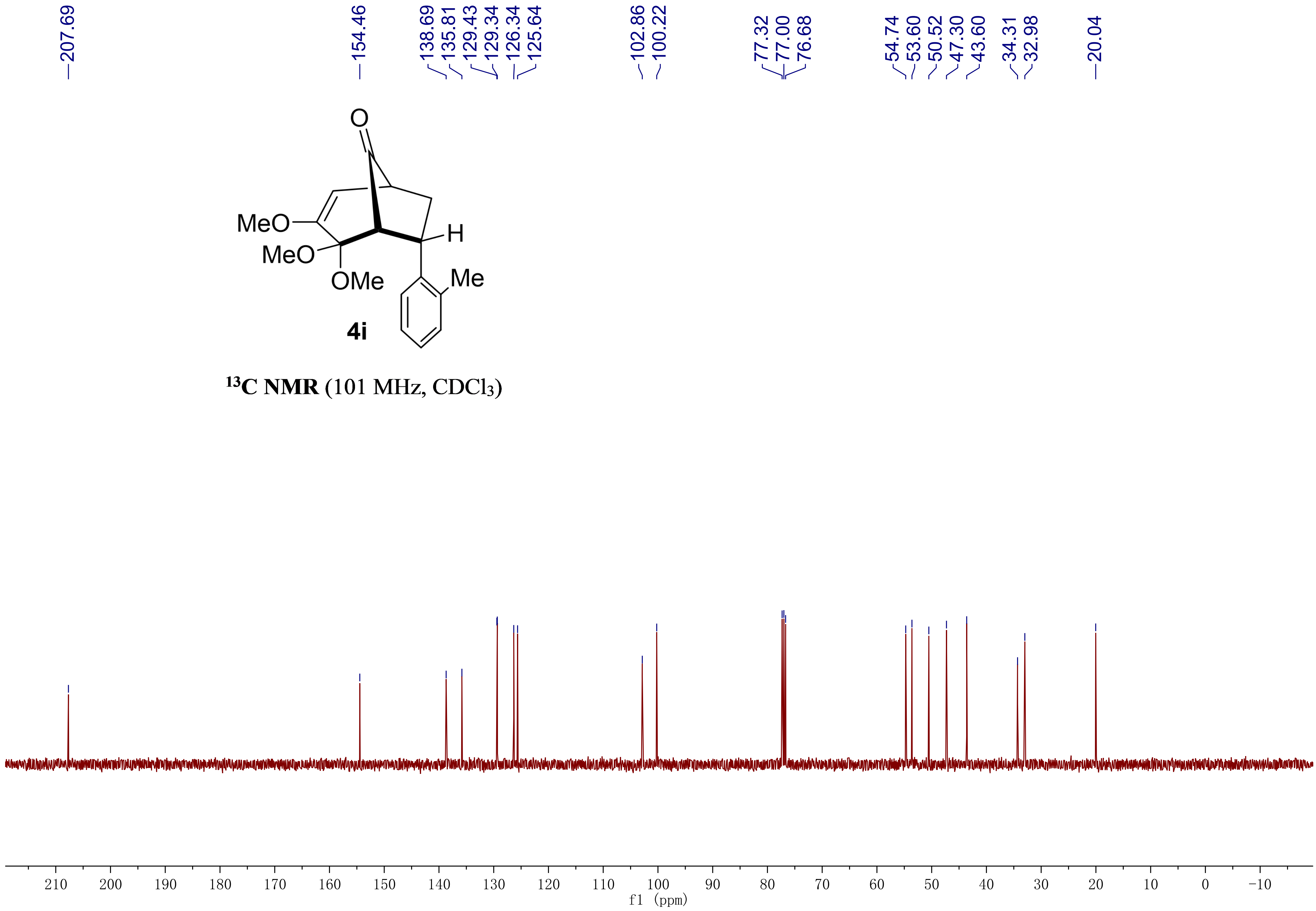

^{13}C NMR (101 MHz, $CDCl_3$)

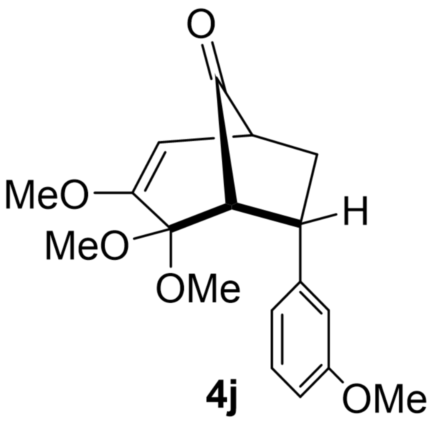

4j

¹H NMR (400 MHz, $CDCl_3$)

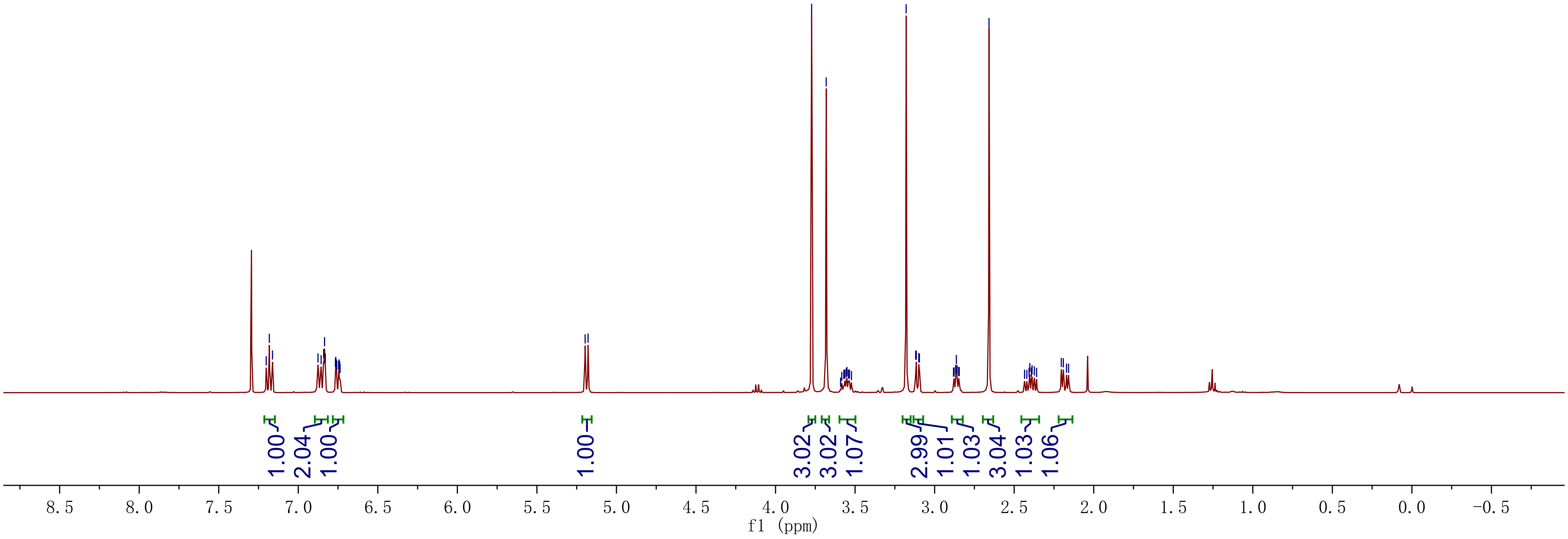

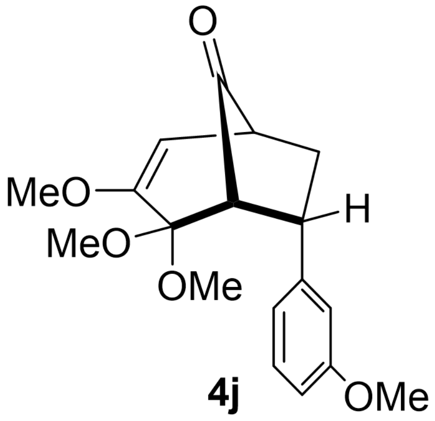

4j

^{13}C NMR (101 MHz, $CDCl_3$)

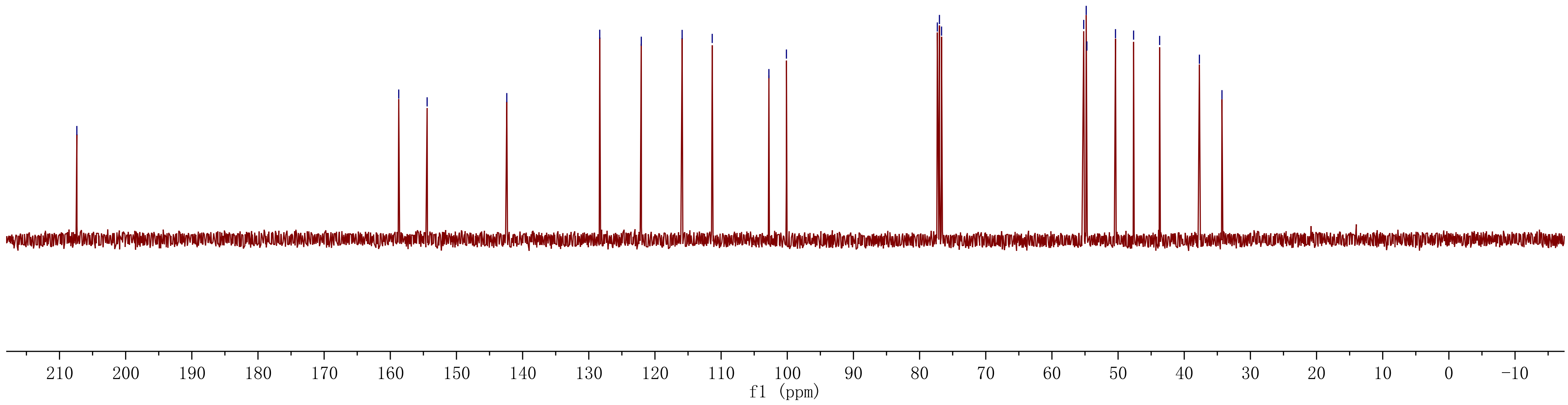

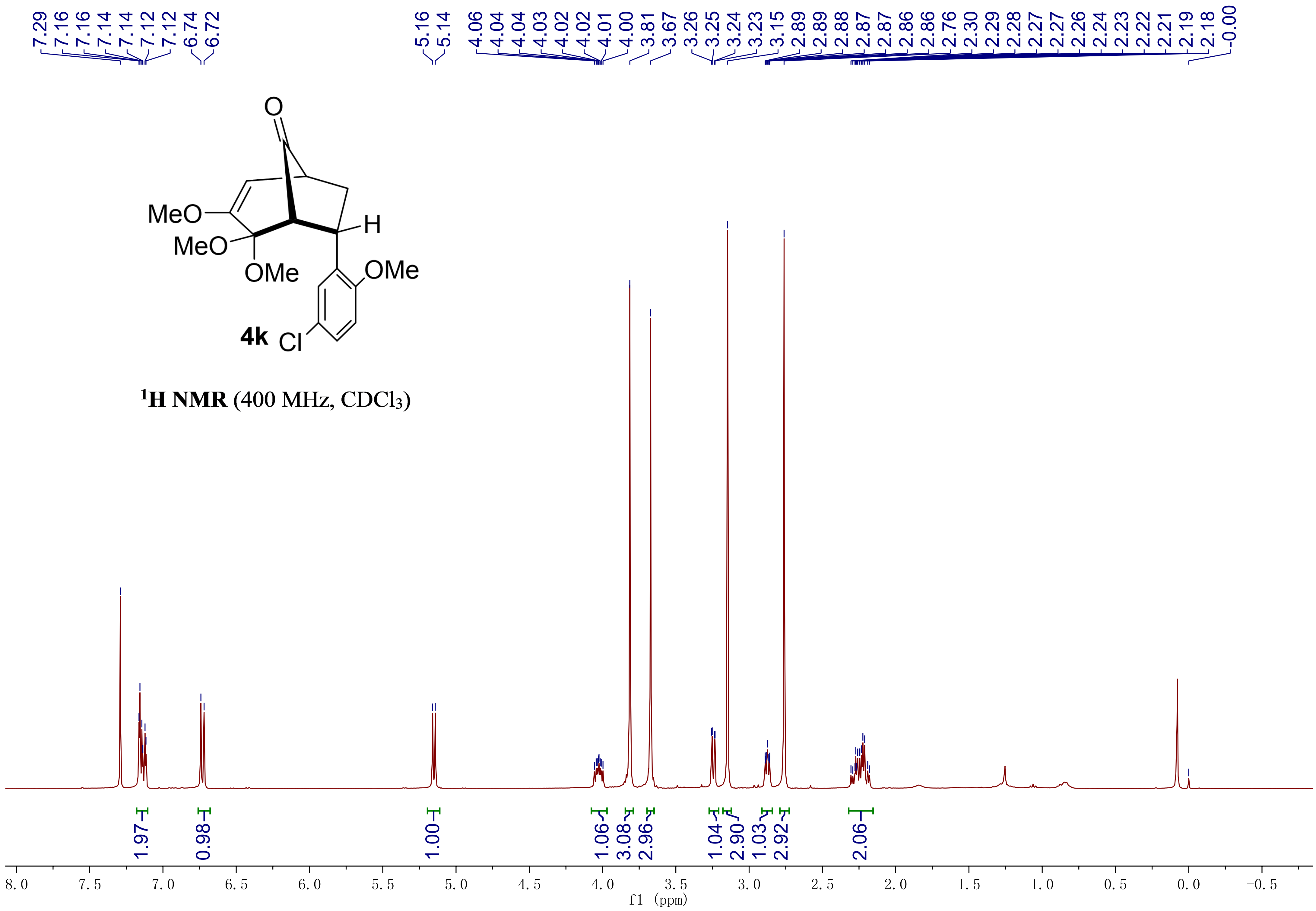

7.29 7.16 7.16 7.14 7.14 7.12 7.12 6.74 6.72
5.16 5.14 4.06 4.04 4.04 4.03 4.02 4.02 4.01 4.00 3.81 3.67 3.26 3.25 3.24 3.23 3.15 2.89 2.89 2.88 2.87 2.87 2.86 2.86 2.76 2.30 2.29 2.28 2.27 2.27 2.26 2.24 2.23 2.22 2.21 2.19 2.18 -0.00
MeO
MeO
OMe
OMe
H
O
Cl
4k
1H NMR (400 MHz, CDCl3)
1.97
0.98
1.00
1.06
3.08
2.96
1.04
2.90
1.03
2.92
2.06
8.0 7.5 7.0 6.5 6.0 5.5 5.0 4.5 4.0 3.5 3.0 2.5 2.0 1.5 1.0 0.5 0.0 -0.5
f1 (ppm)

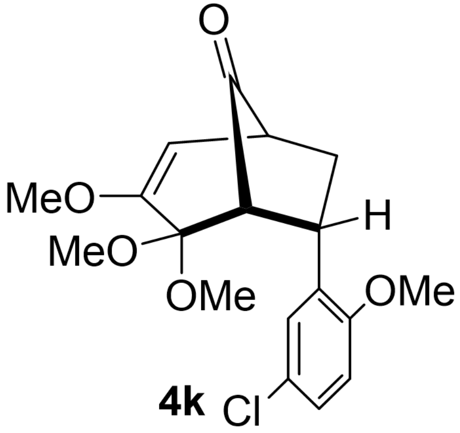

4k

^{13}C NMR (101 MHz, $CDCl_3$)

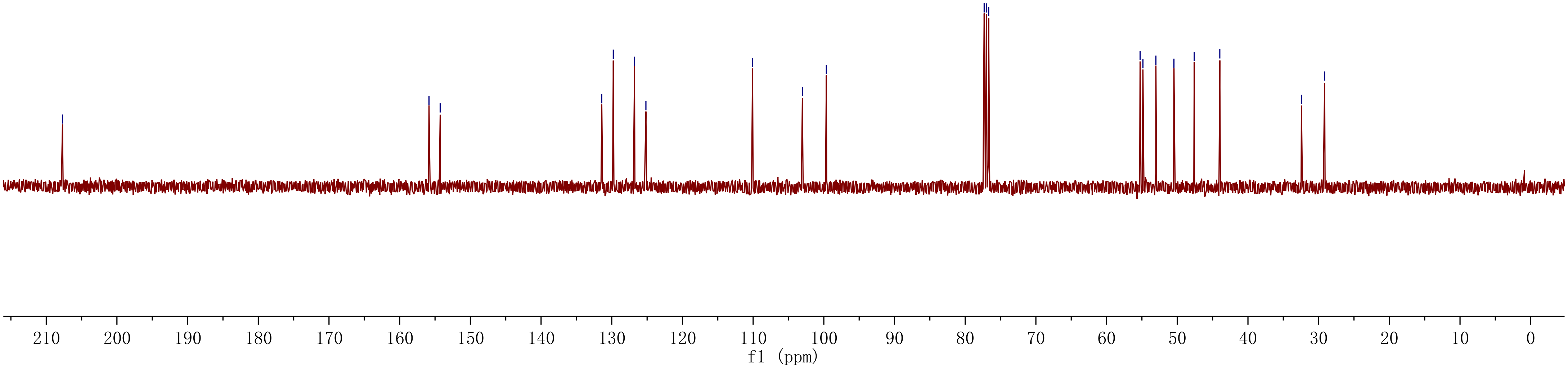

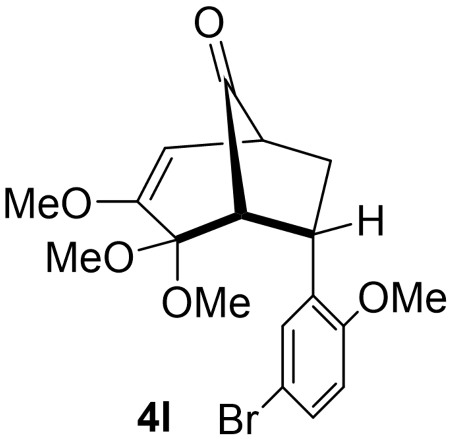

4l

^{1}H NMR (400 MHz, $CDCl_3$)

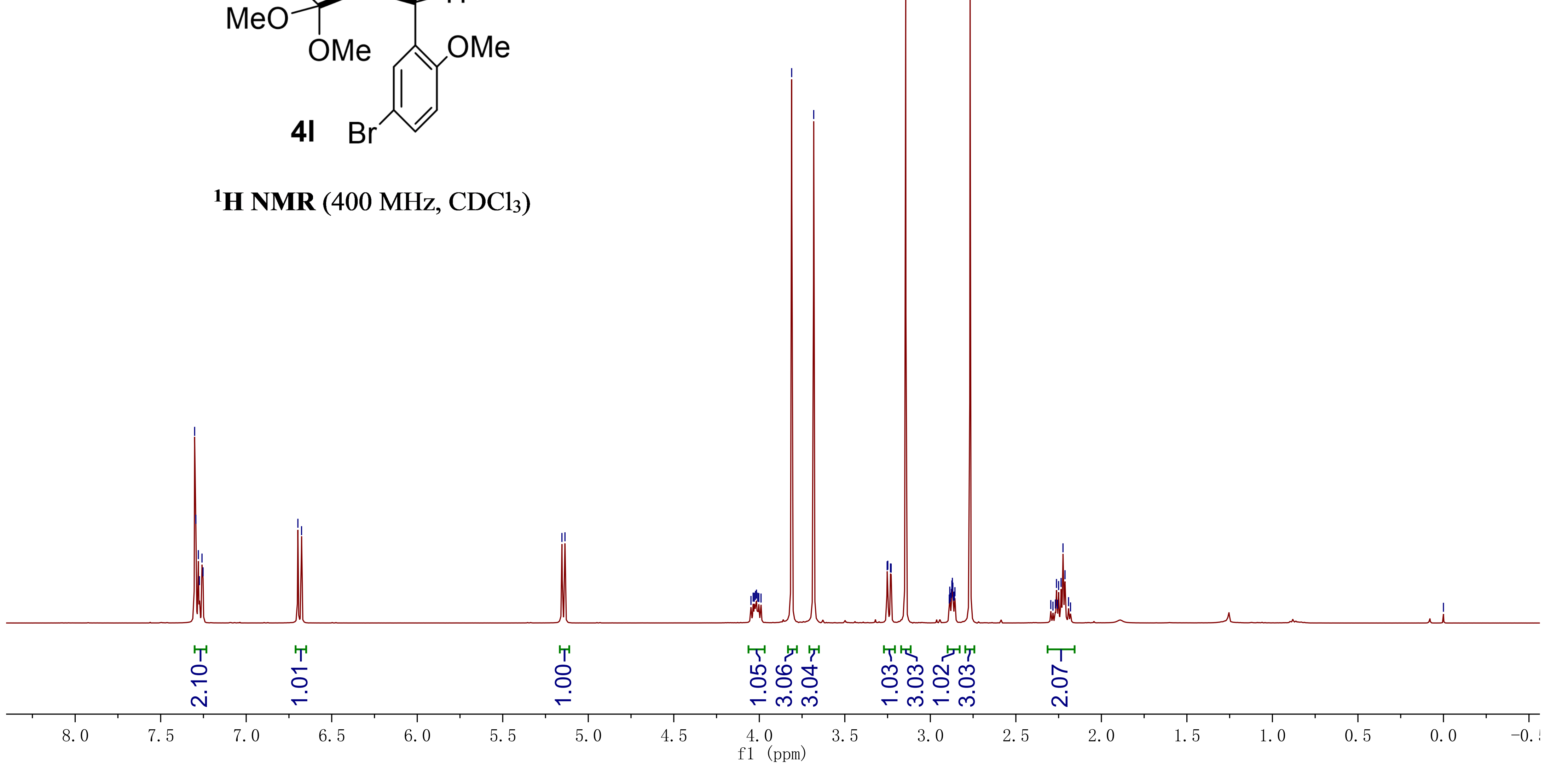

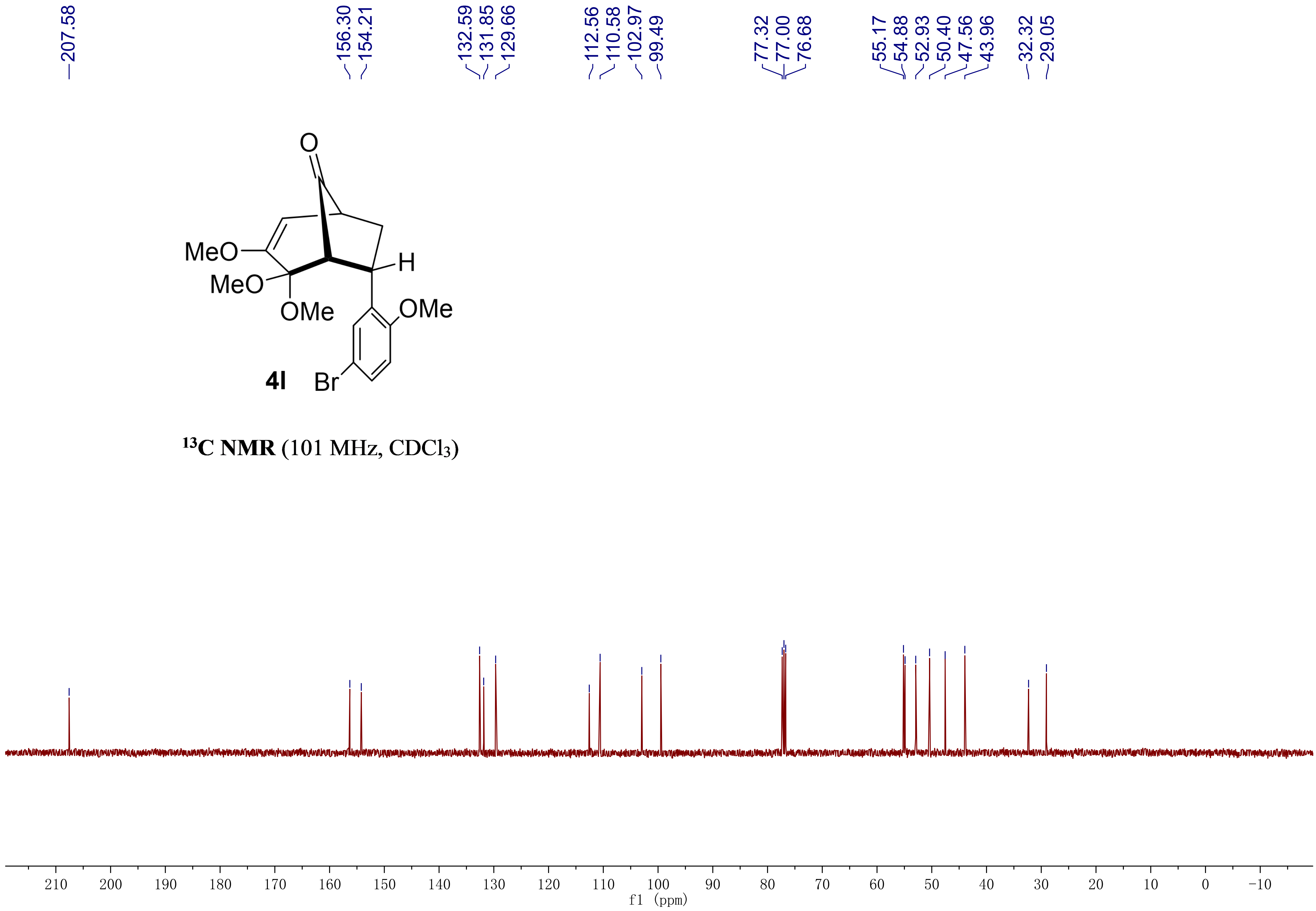
207.58
156.30
154.21
132.59
131.85
129.66
112.56
110.58
102.97
99.49
77.32
77.00
76.68
55.17
54.88
52.93
50.40
47.56
43.96
32.32
29.05
O
MeO
MeO
OMe
H
OMe
4l
Br
^{13}C NMR (101 MHz, CDCl$_3$)
210
200
190
180
170
160
150
140
130
120
110
100
90
80
70
60
50
40
30
20
10
0
−10
f1 (ppm)

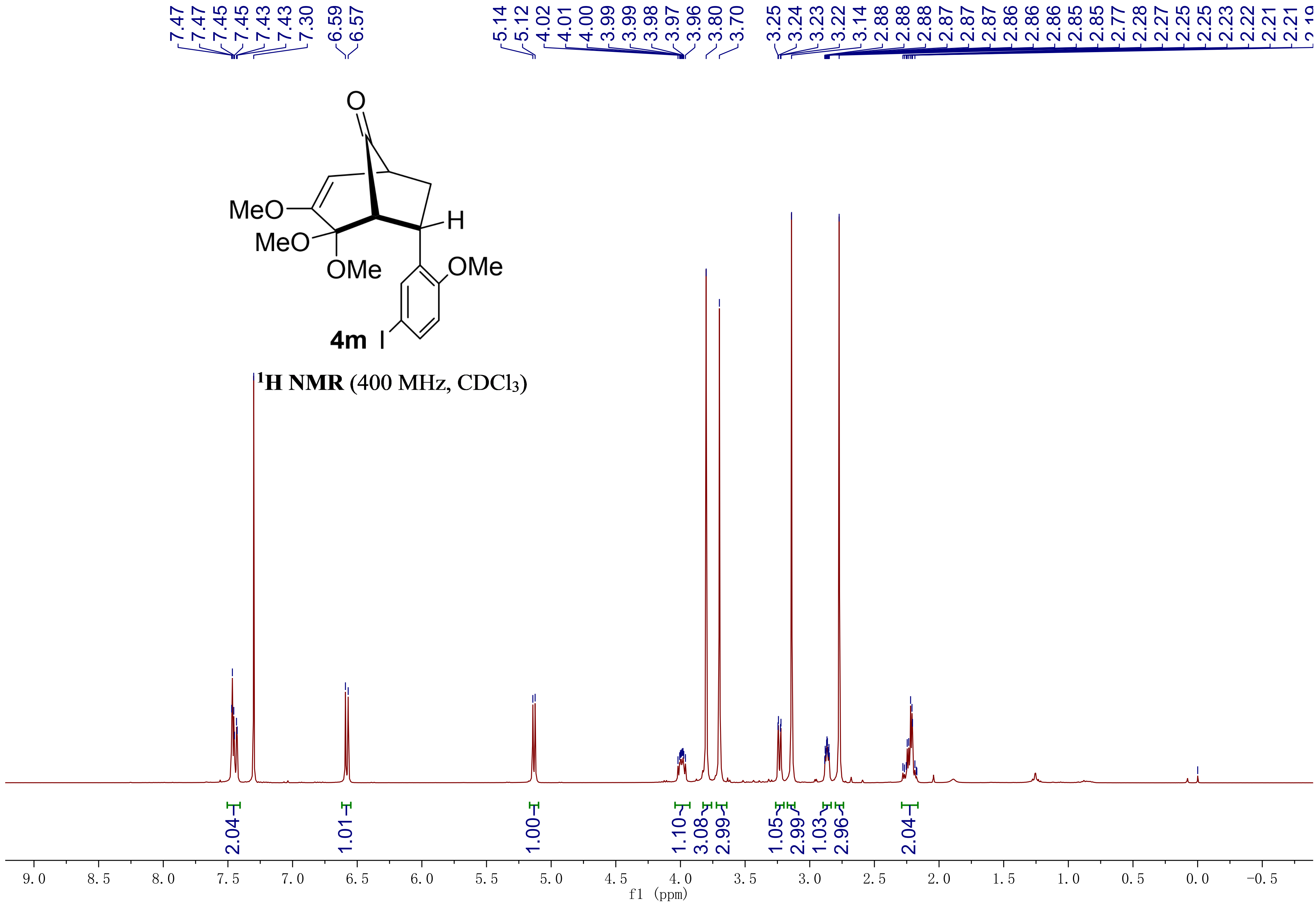
7.47 7.47 7.45 7.45 7.43 7.43 7.30 6.59 6.57
5.14 5.12 4.02 4.01 4.00 3.99 3.99 3.98 3.97 3.96 3.80 3.70
3.25 3.24 3.23 3.22 3.14 2.88 2.88 2.88 2.87 2.87 2.87 2.86 2.86 2.86 2.85 2.85 2.77 2.28 2.27 2.25 2.25 2.23 2.22 2.21 2.21
MeO
MeO
OMe
OMe
H
O
I
4m
1H NMR (400 MHz, CDCl3)
2.04
1.01
1.00
1.10
3.08
2.99
1.05
2.99
1.03
2.96
2.04
9.0 8.5 8.0 7.5 7.0 6.5 6.0 5.5 5.0 4.5 4.0 3.5 3.0 2.5 2.0 1.5 1.0 0.5 0.0 -0.5
f1 (ppm)

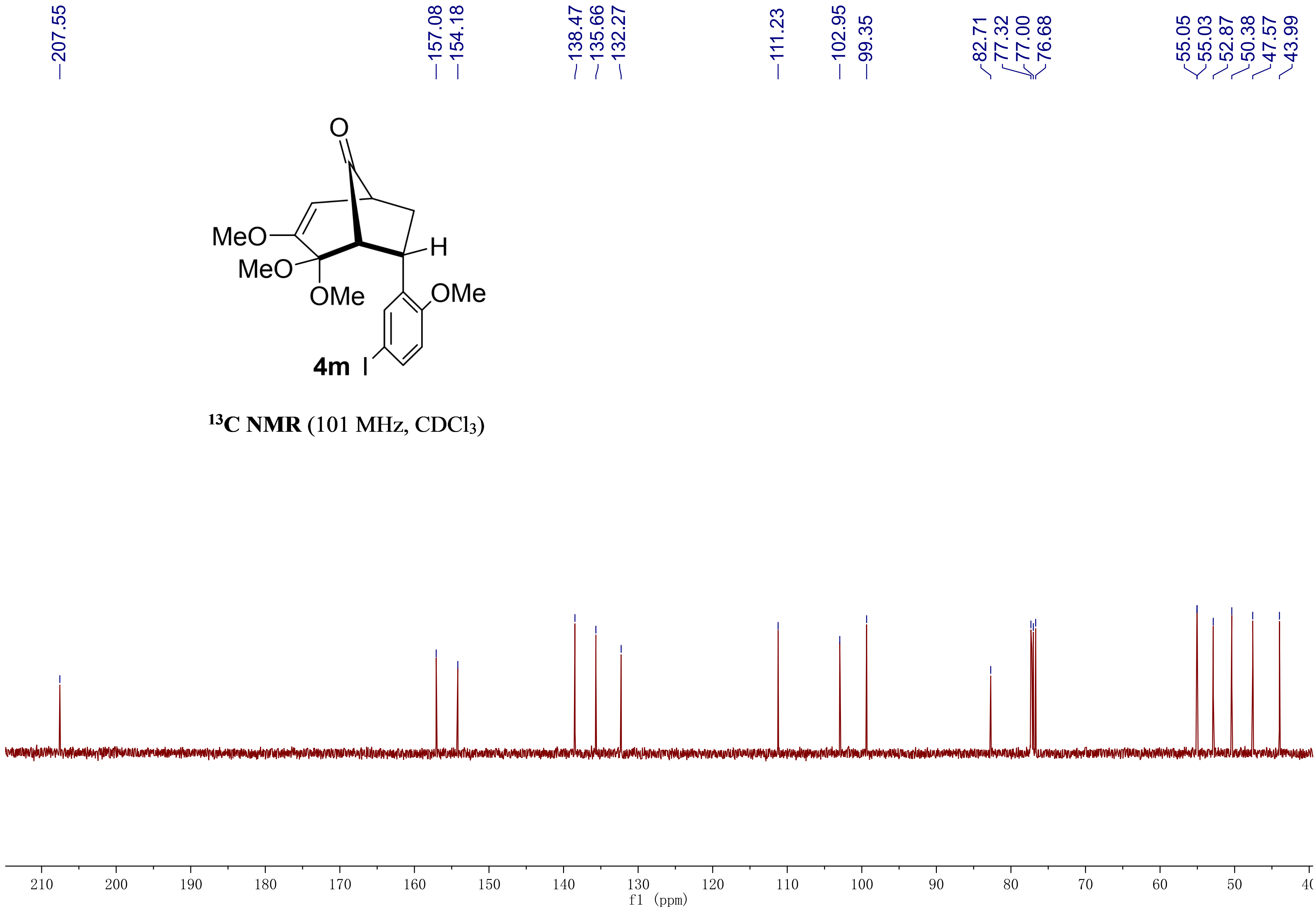

207.55
157.08
154.18
138.47
135.66
132.27
111.23
102.95
99.35
82.71
77.32
77.00
76.68
55.05
55.03
52.87
50.38
47.57
43.99
O
MeO
MeO
OMe
H
OMe
4m
I
13C NMR (101 MHz, CDCl3)
210 200 190 180 170 160 150 140 130 120 110 100 90 80 70 60 50 40
f1 (ppm)

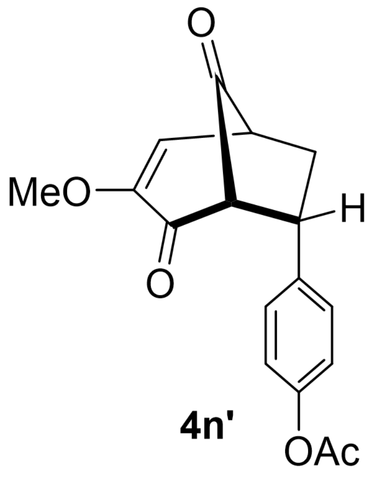

^{1}H NMR (400 MHz, $CDCl_3$)

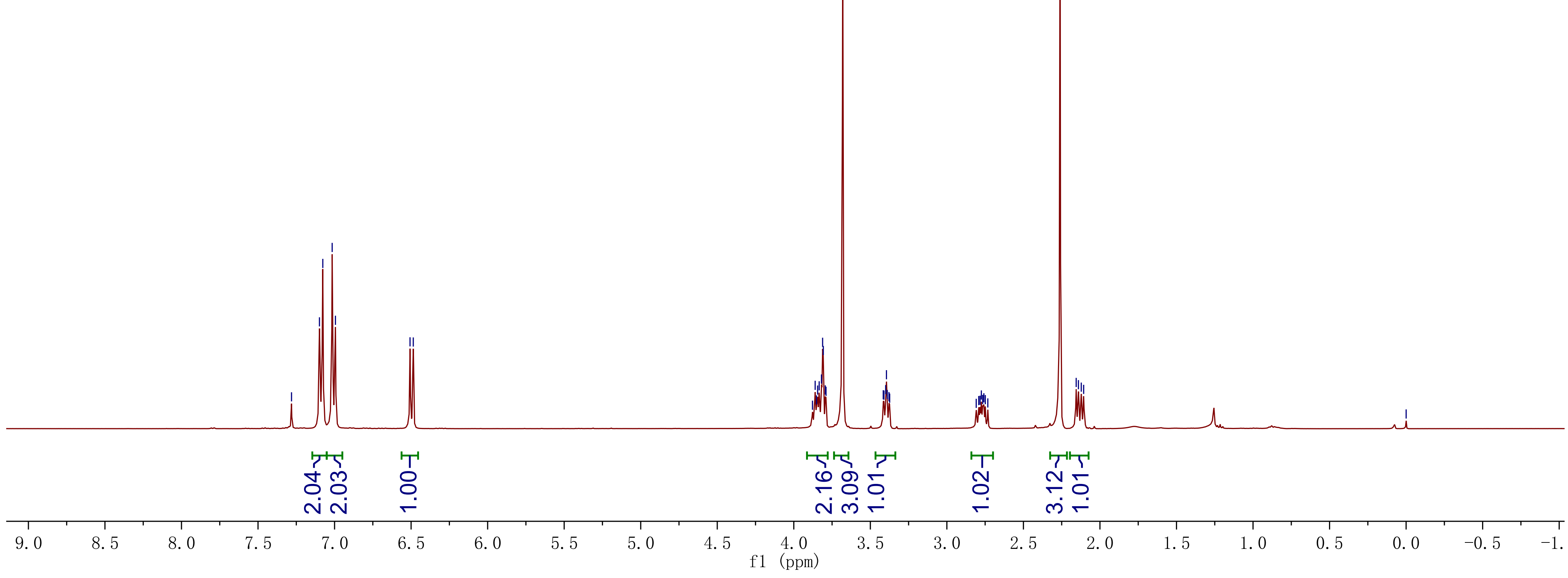

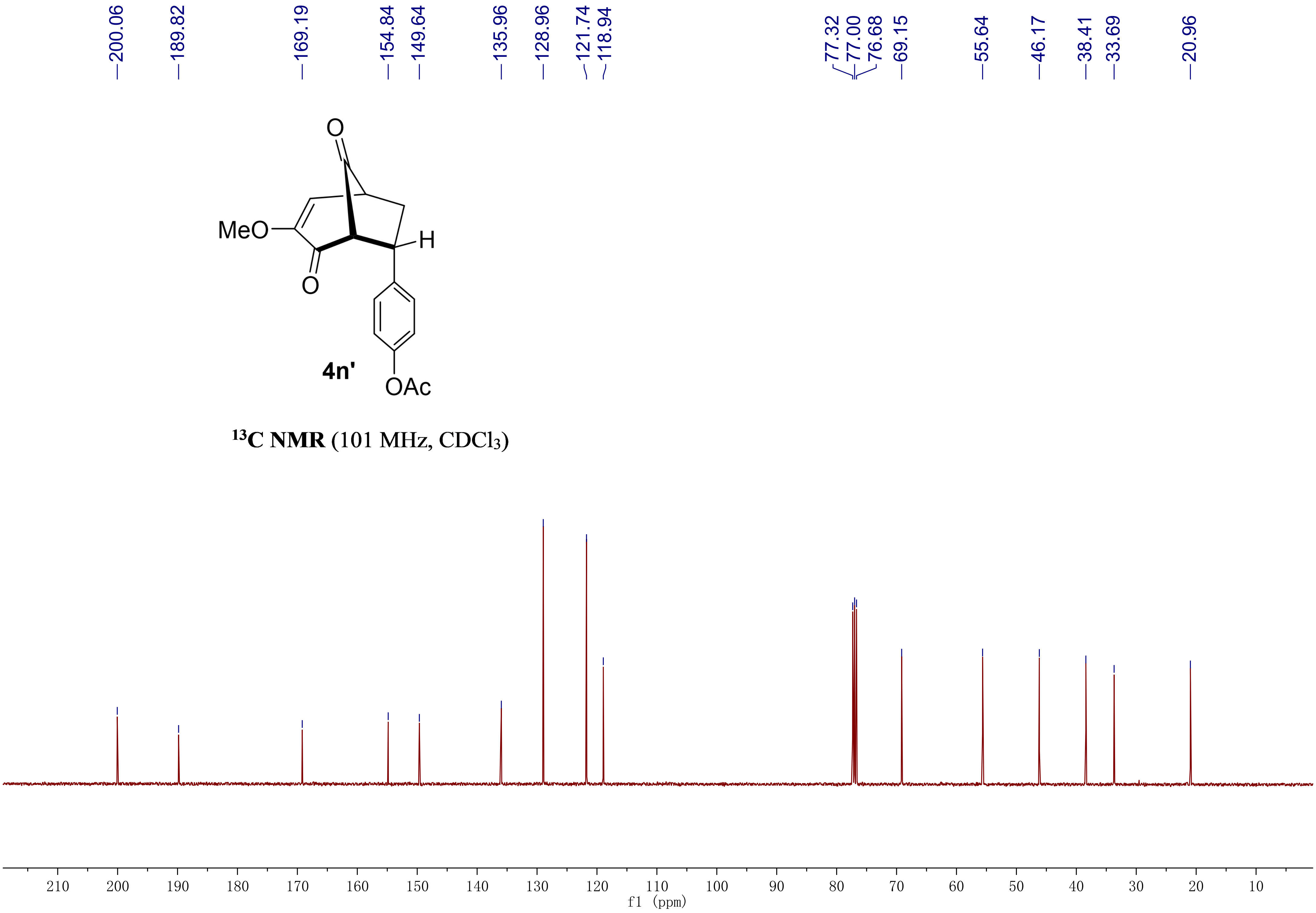

4n'

^{13}C NMR (101 MHz, $CDCl_3$)

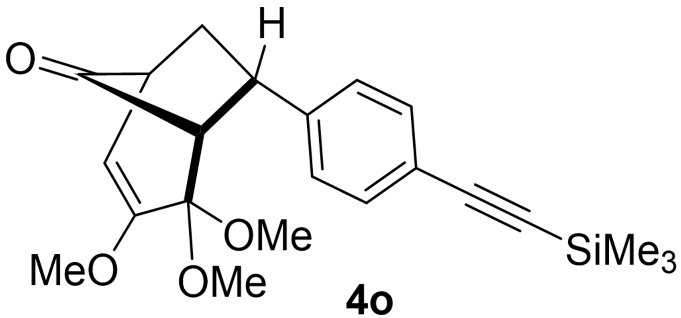

4o

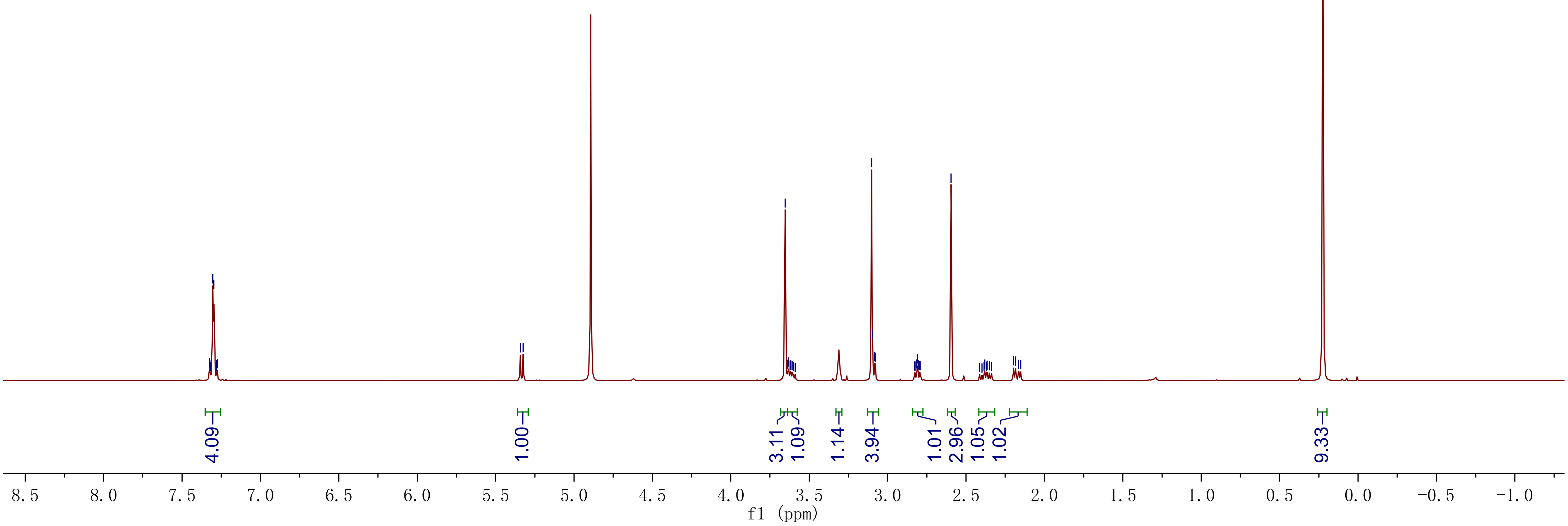

^{1}H NMR (400 MHz, Methanol-*d*$_4$)

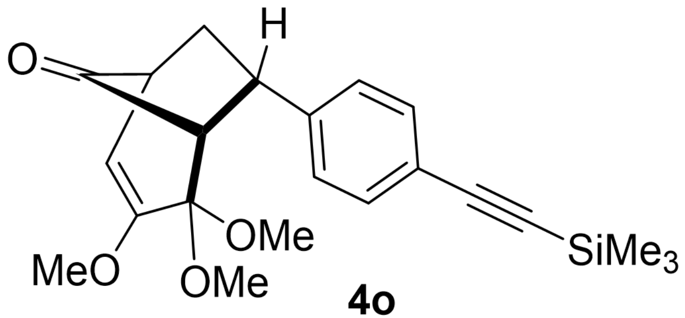

4o

^{13}C NMR (101 MHz, Methanol-d_4)

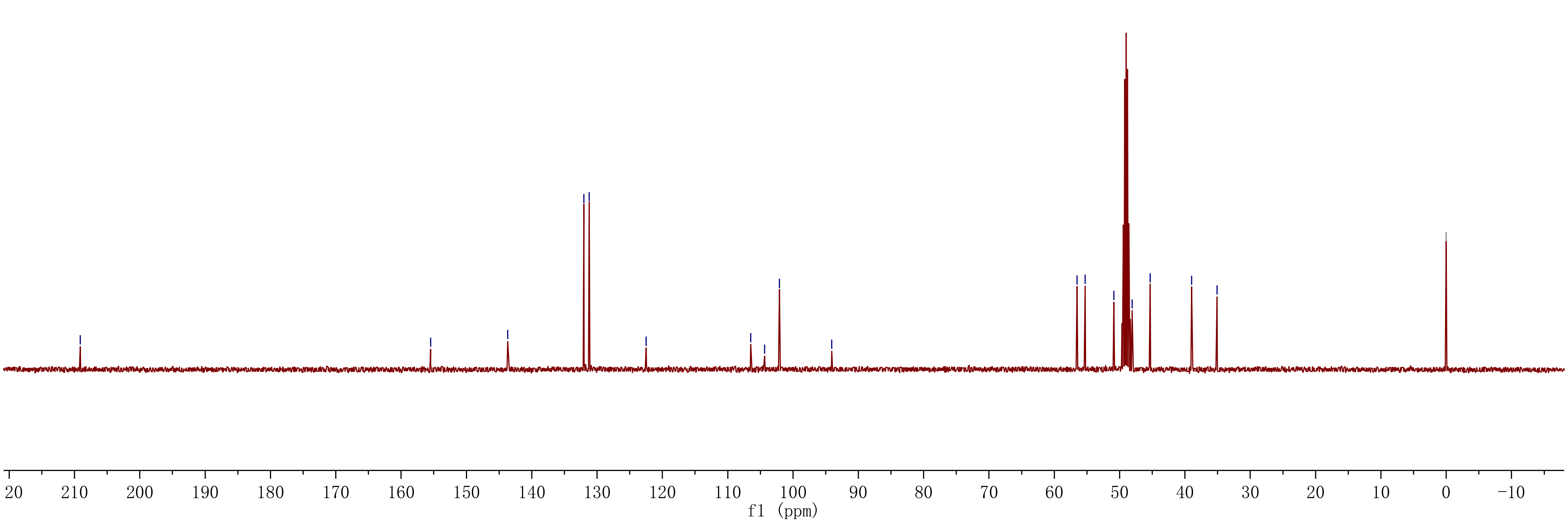

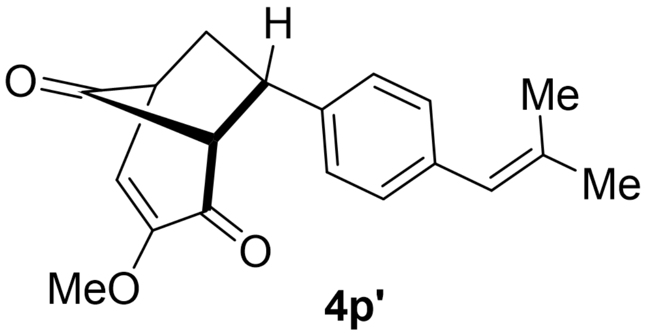

4p'

^{1}H NMR (400 MHz, $CDCl_3$)

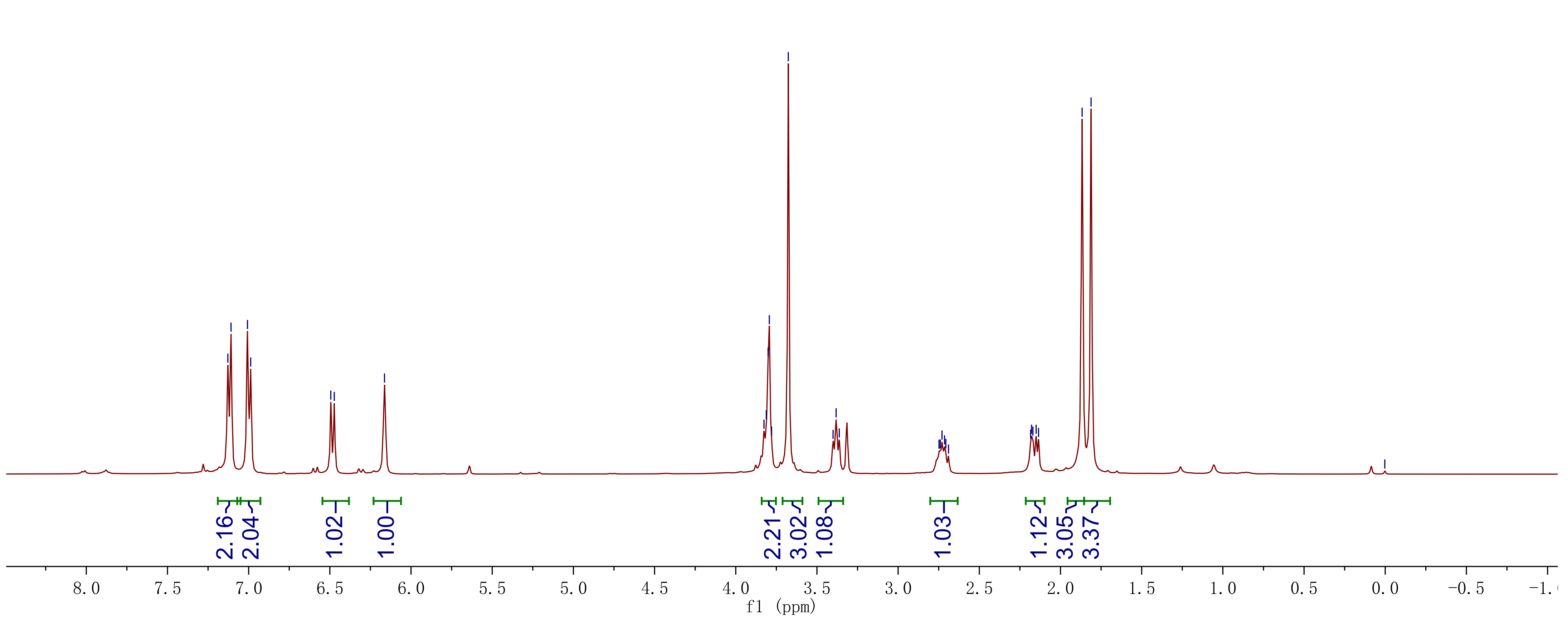

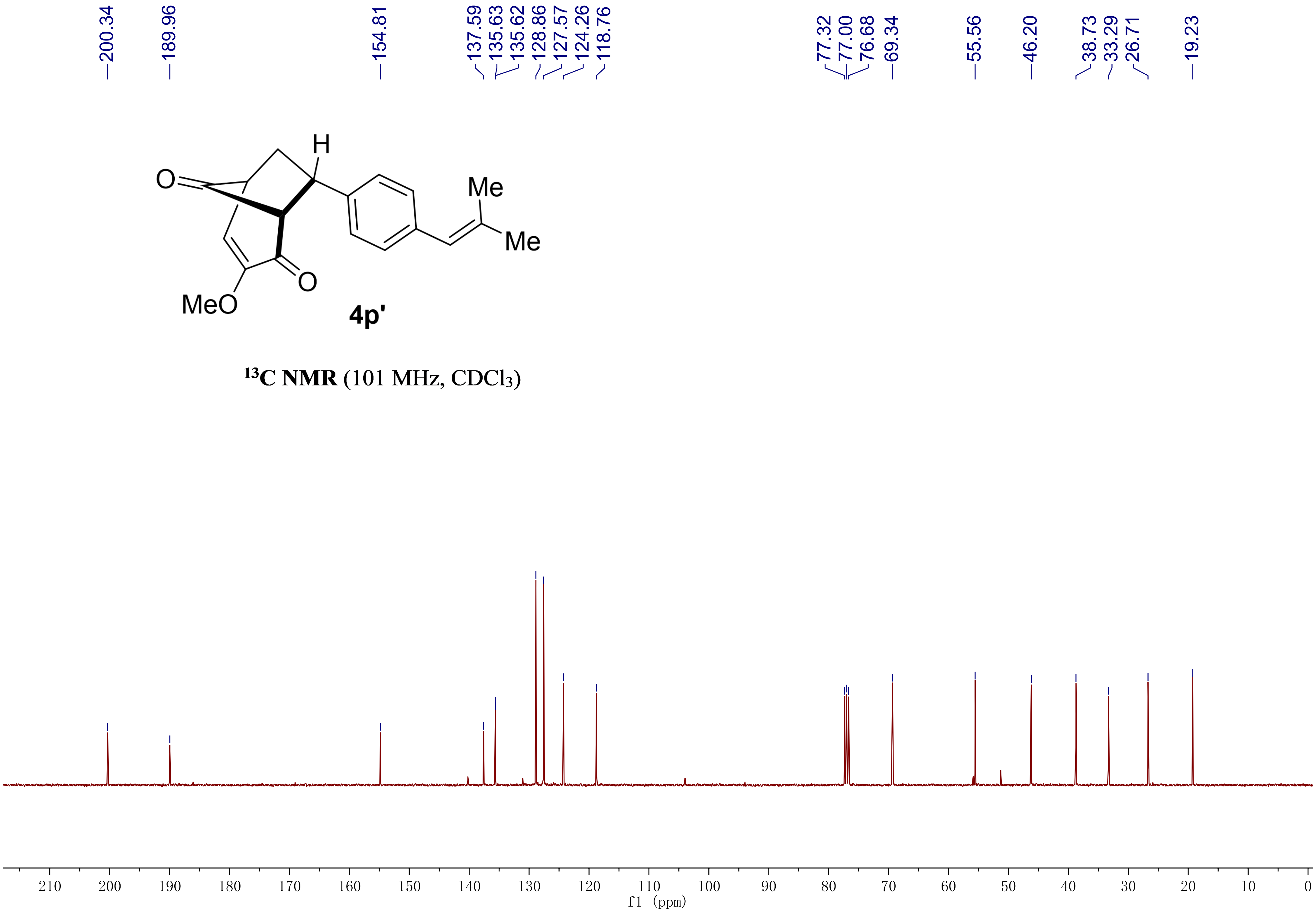

^{13}C NMR (101 MHz, $CDCl_3$)

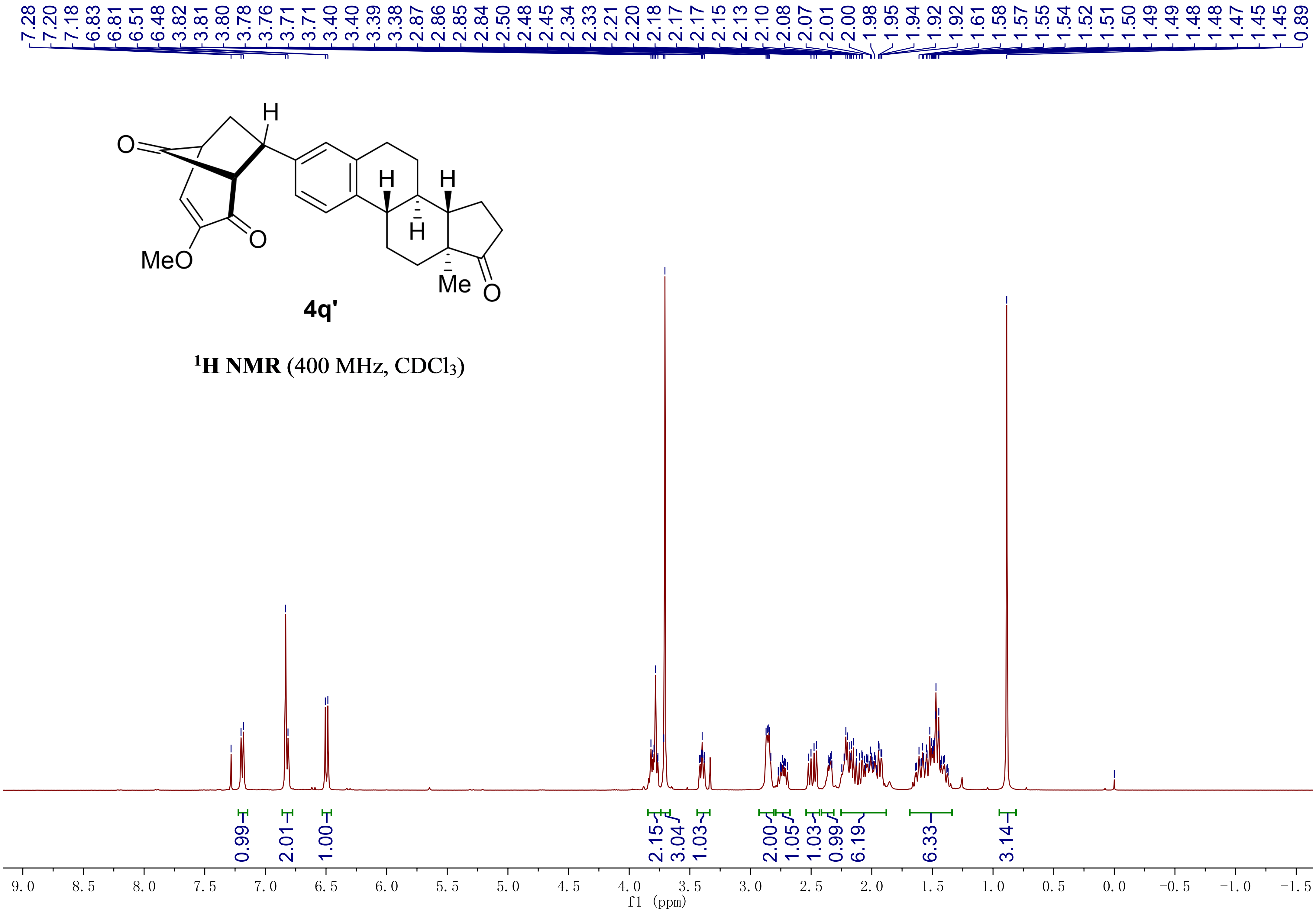

7.28 7.20 7.18 6.83 6.81 6.51 6.48 3.82 3.81 3.80 3.78 3.76 3.71 3.71 3.40 3.40 3.39 3.38 2.87 2.86 2.85 2.84 2.50 2.48 2.45 2.34 2.33 2.21 2.20 2.18 2.17 2.17 2.15 2.13 2.10 2.08 2.07 2.01 2.00 1.98 1.95 1.94 1.92 1.92 1.61 1.58 1.57 1.55 1.54 1.52 1.51 1.50 1.49 1.49 1.48 1.48 1.47 1.45 1.45 0.89
O
H
H
H
MeO
O
H
Me
O
4q'
^{1}H NMR (400 MHz, CDCl$_3$)
0.99
2.01
1.00
2.15
3.04
1.03
2.00
1.05
1.03
0.99
6.19
6.33
3.14
9.0 8.5 8.0 7.5 7.0 6.5 6.0 5.5 5.0 4.5 4.0 3.5 3.0 2.5 2.0 1.5 1.0 0.5 0.0 -0.5 -1.0 -1.5
f1 (ppm)

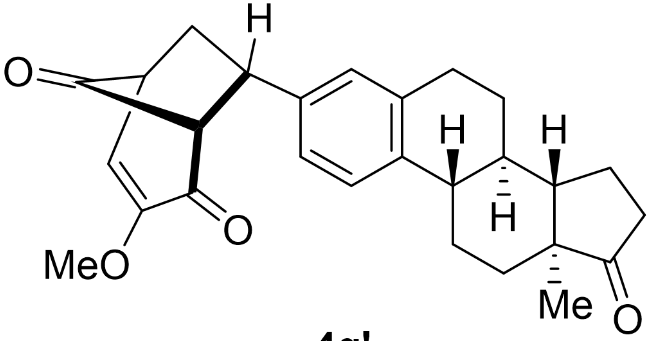

4q'

^{13}C NMR (101 MHz, $CDCl_3$)

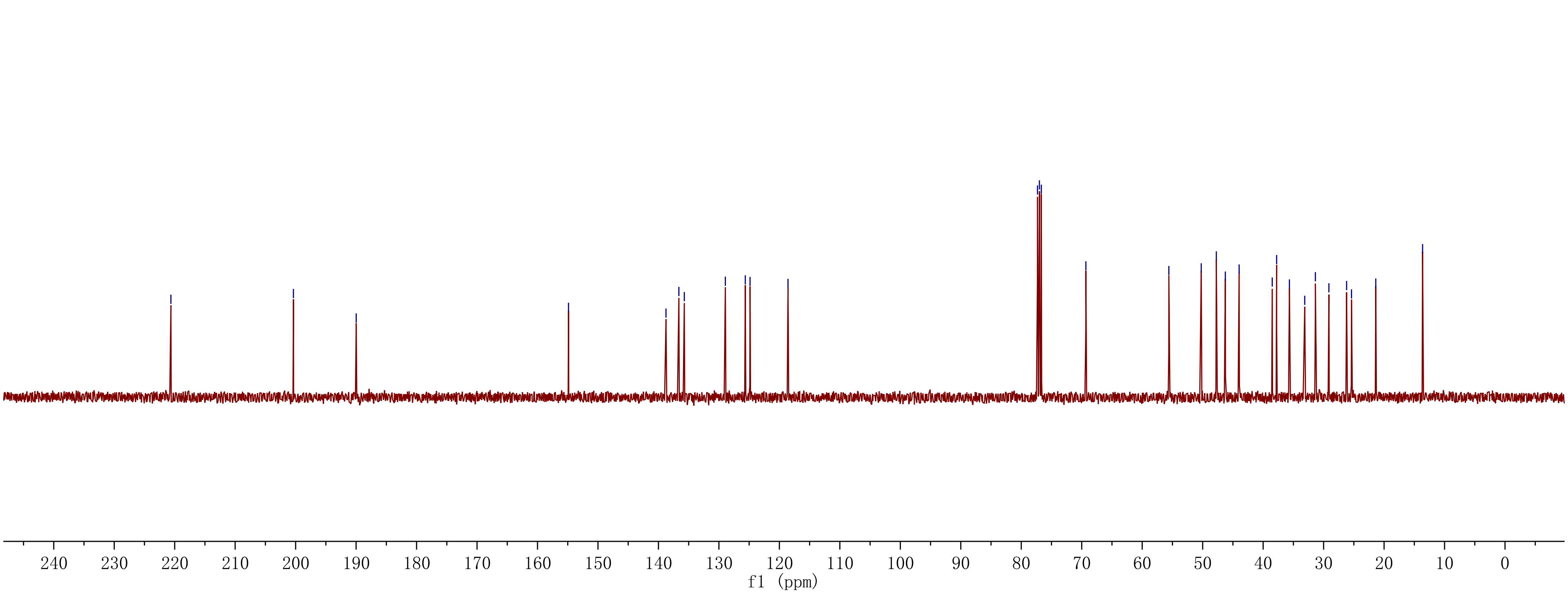

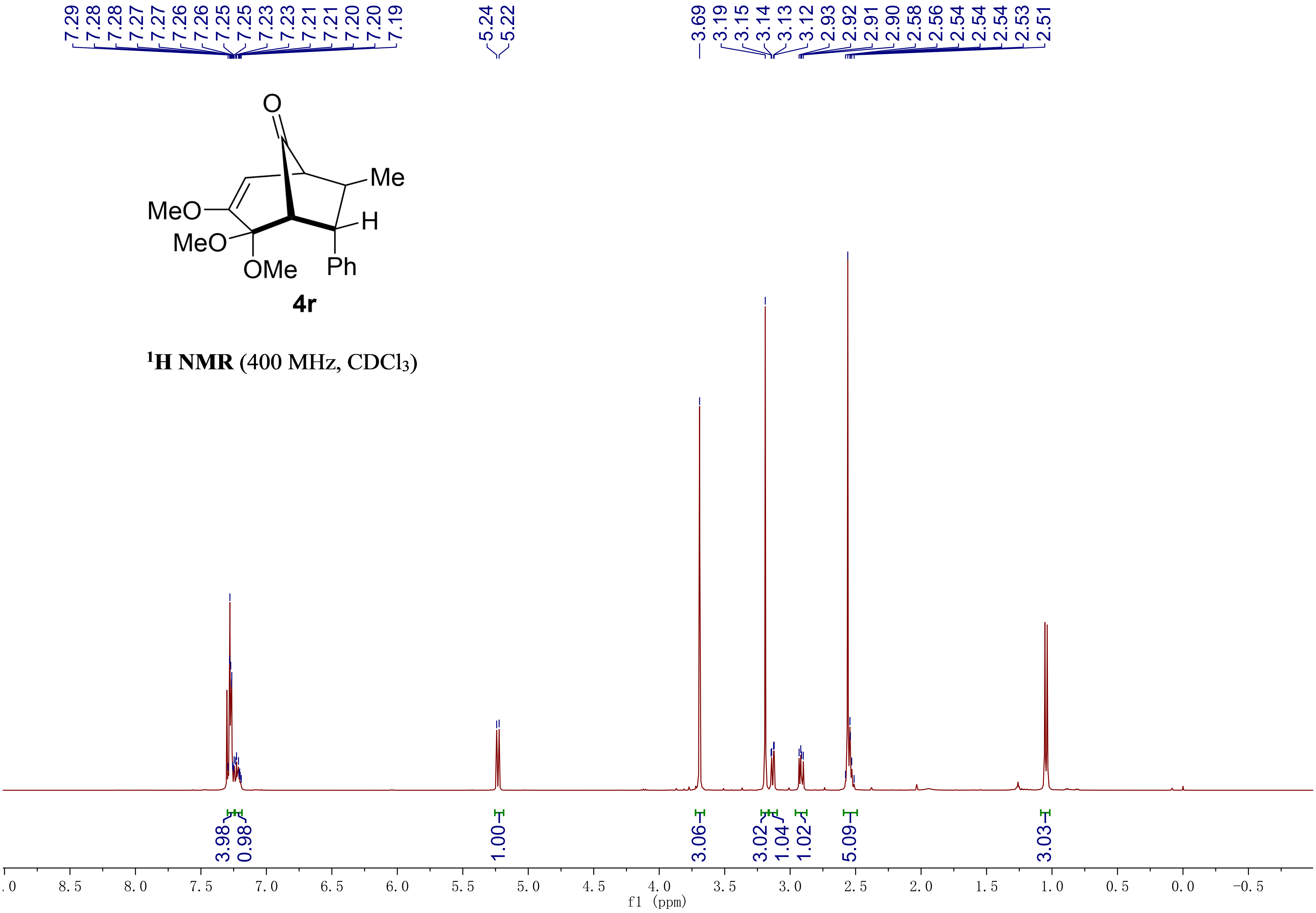
7.29 7.28 7.28 7.27 7.27 7.26 7.26 7.25 7.25 7.23 7.23 7.21 7.21 7.20 7.20 7.19
5.24 5.22
3.69 3.19 3.15 3.14 3.13 3.12 2.93 2.92 2.91 2.90 2.58 2.56 2.54 2.54 2.54 2.53 2.51
O
Me
MeO
H
MeO
OMe
Ph
4r
1H NMR (400 MHz, CDCl3)
3.98 0.98 1.00 3.06 3.02 1.04 1.02 5.09 3.03
.0 8.5 8.0 7.5 7.0 6.5 6.0 5.5 5.0 4.5 4.0 3.5 3.0 2.5 2.0 1.5 1.0 0.5 0.0 -0.5
f1 (ppm)

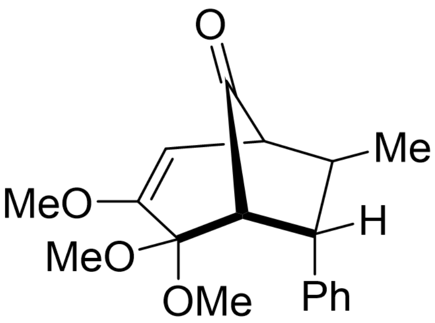

4r

^{13}C NMR (101 MHz, $CDCl_3$)

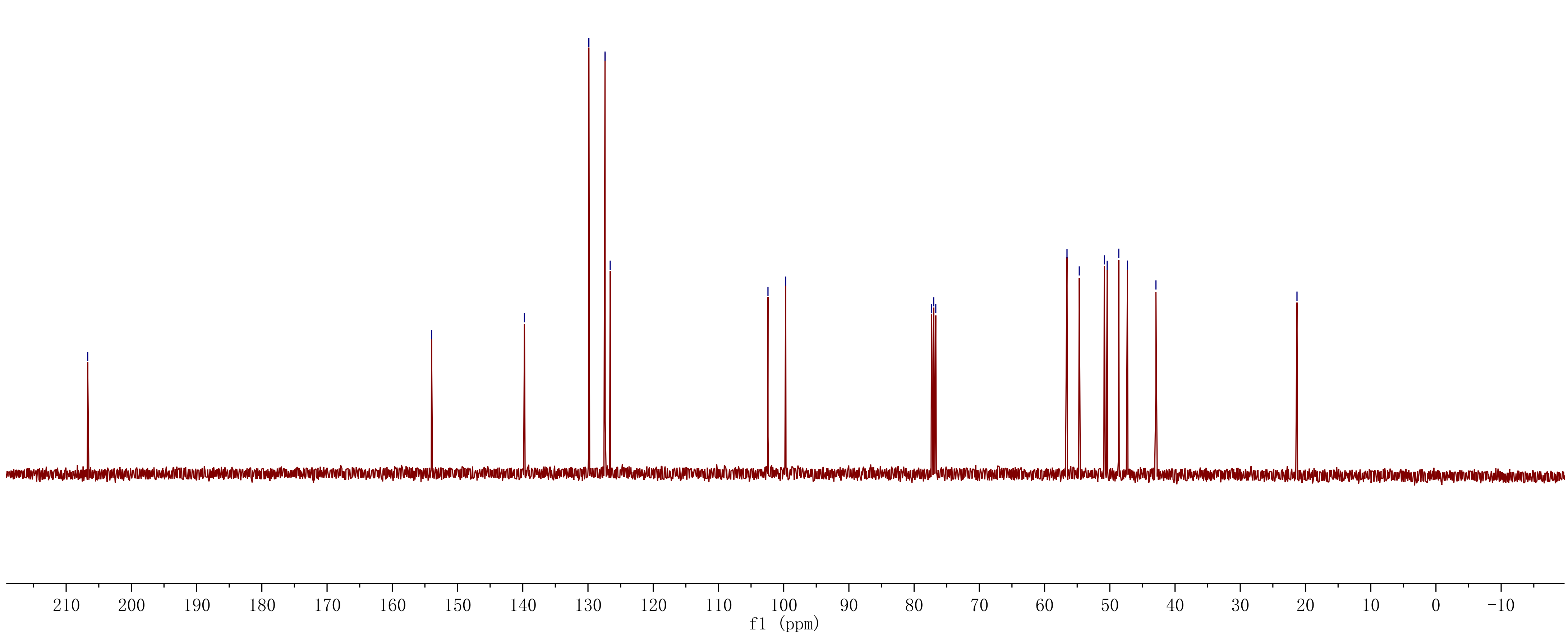

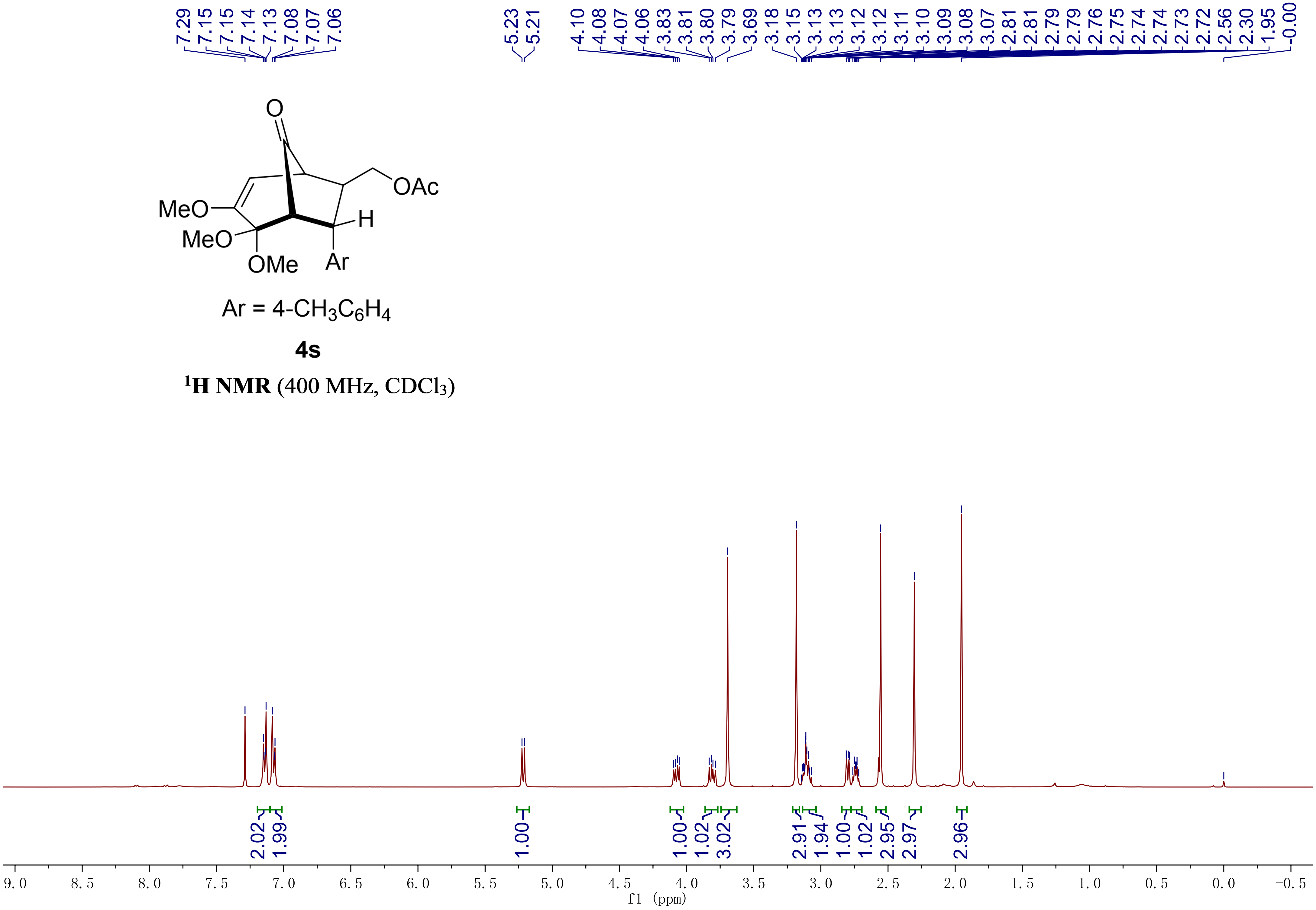

Ar = 4-$CH_3C_6H_4$

4s

^{1}H NMR (400 MHz, $CDCl_3$)

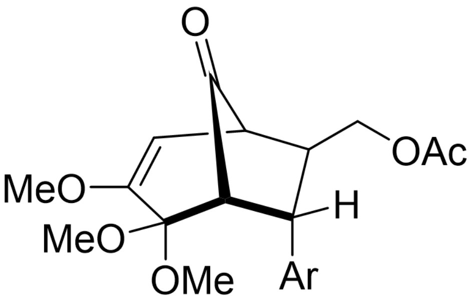

Ar = 4-$CH_3C_6H_4$

4s

^{13}C NMR (101 MHz, $CDCl_3$)

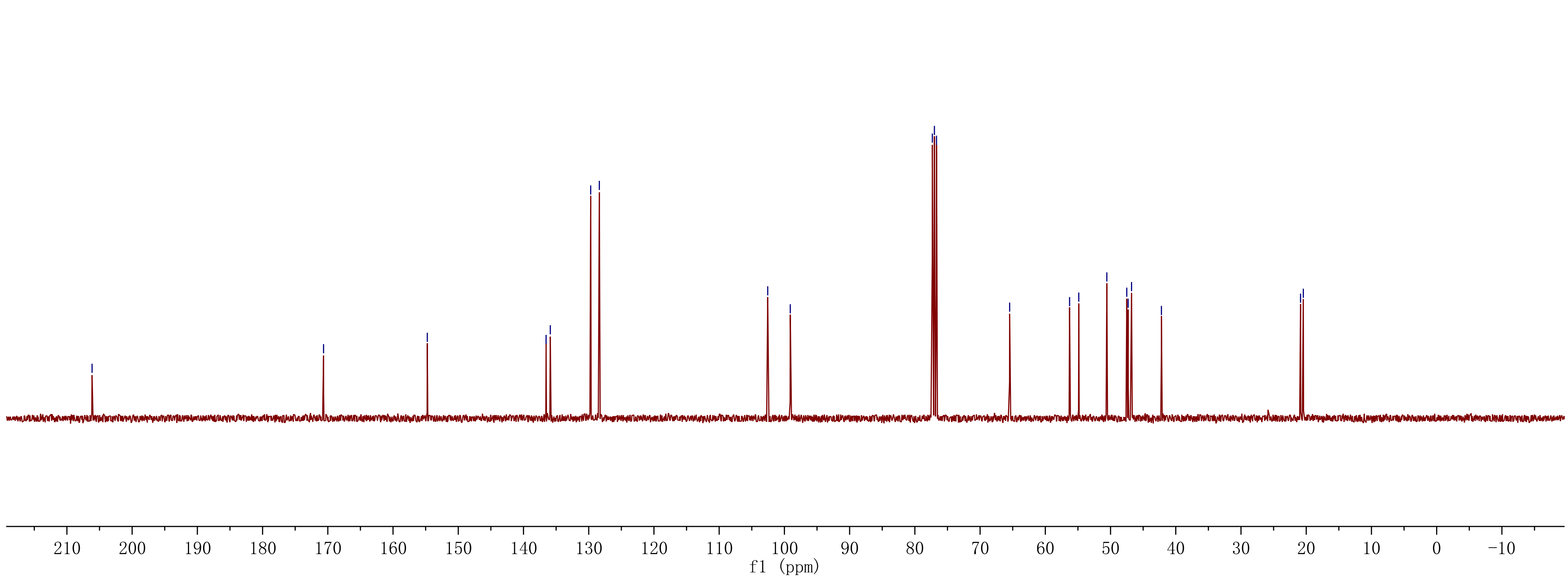

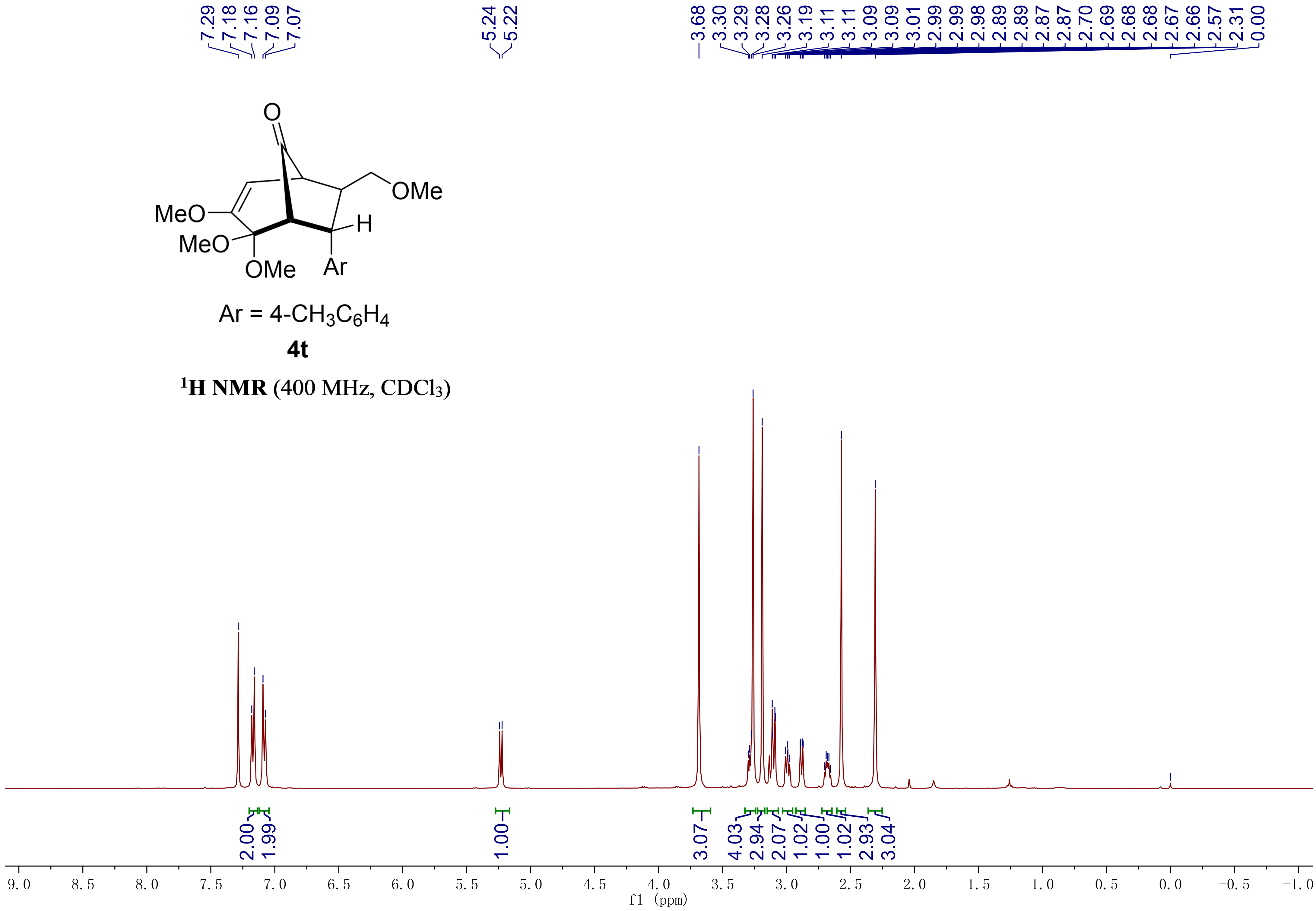
7.29
7.18
7.16
7.09
7.07
5.24
5.22
3.68
3.30
3.29
3.28
3.26
3.19
3.11
3.11
3.09
3.09
3.01
2.99
2.99
2.98
2.89
2.89
2.87
2.87
2.70
2.69
2.68
2.68
2.67
2.66
2.57
2.31
0.00
O
OMe
MeO
MeO
H
OMe
Ar
Ar = 4-CH3C6H4
4t
1H NMR (400 MHz, CDCl3)
2.00
1.99
1.00
3.07
4.03
2.94
2.07
1.02
1.00
1.02
2.93
3.04
9.0
8.5
8.0
7.5
7.0
6.5
6.0
5.5
5.0
4.5
4.0
3.5
3.0
2.5
2.0
1.5
1.0
0.5
0.0
-0.5
-1.0
f1 (ppm)

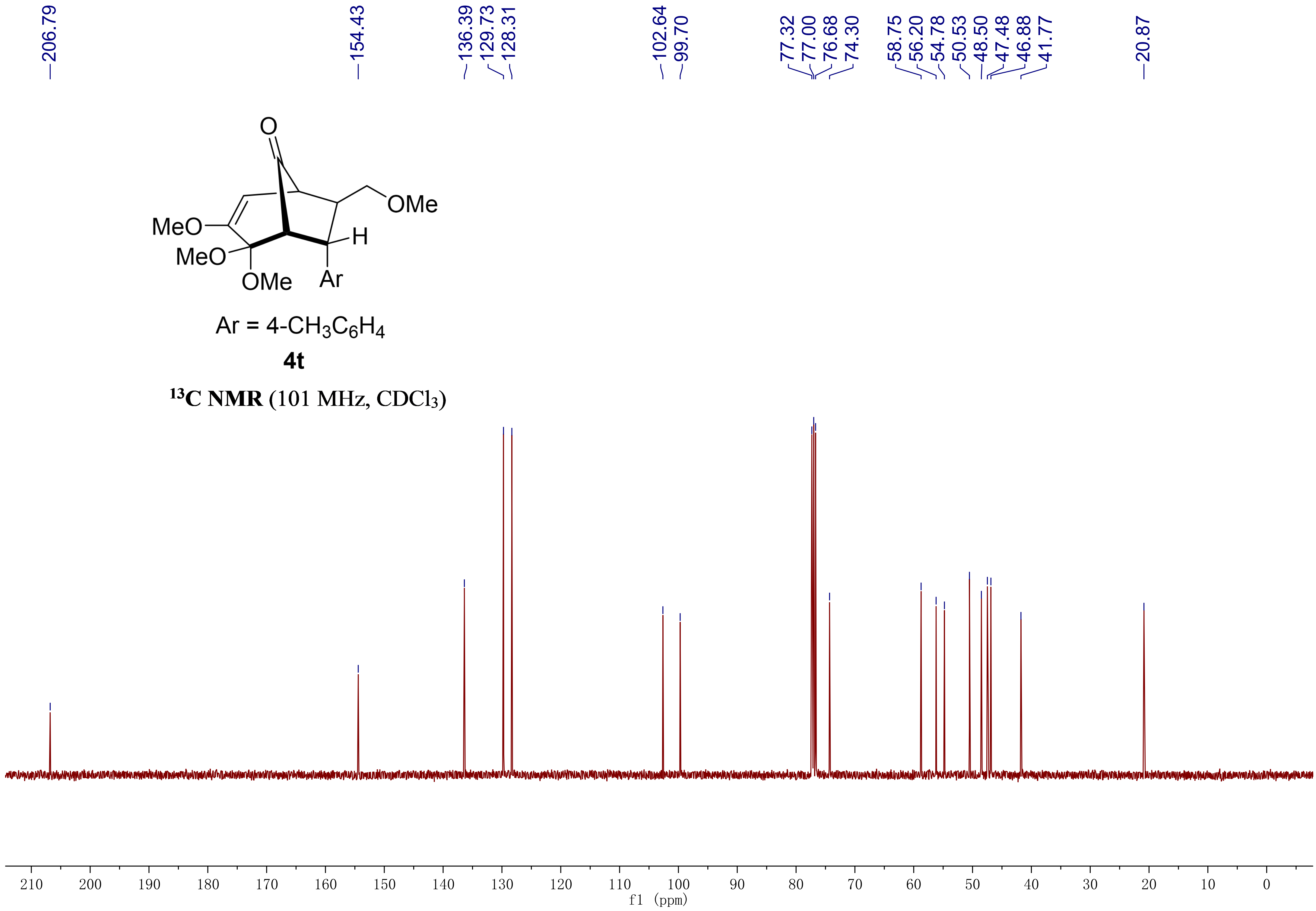

Ar = 4-$CH_3C_6H_4$

4t

^{13}C NMR (101 MHz, $CDCl_3$)

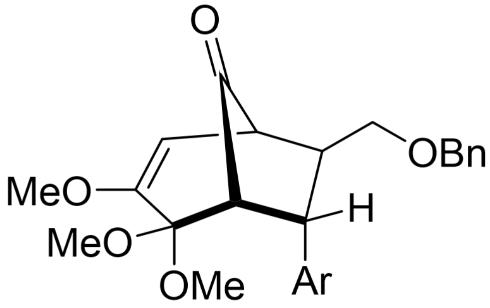

Ar = 4-$CH_3C_6H_4$

4u

^{1}H NMR (400 MHz, $CDCl_3$)

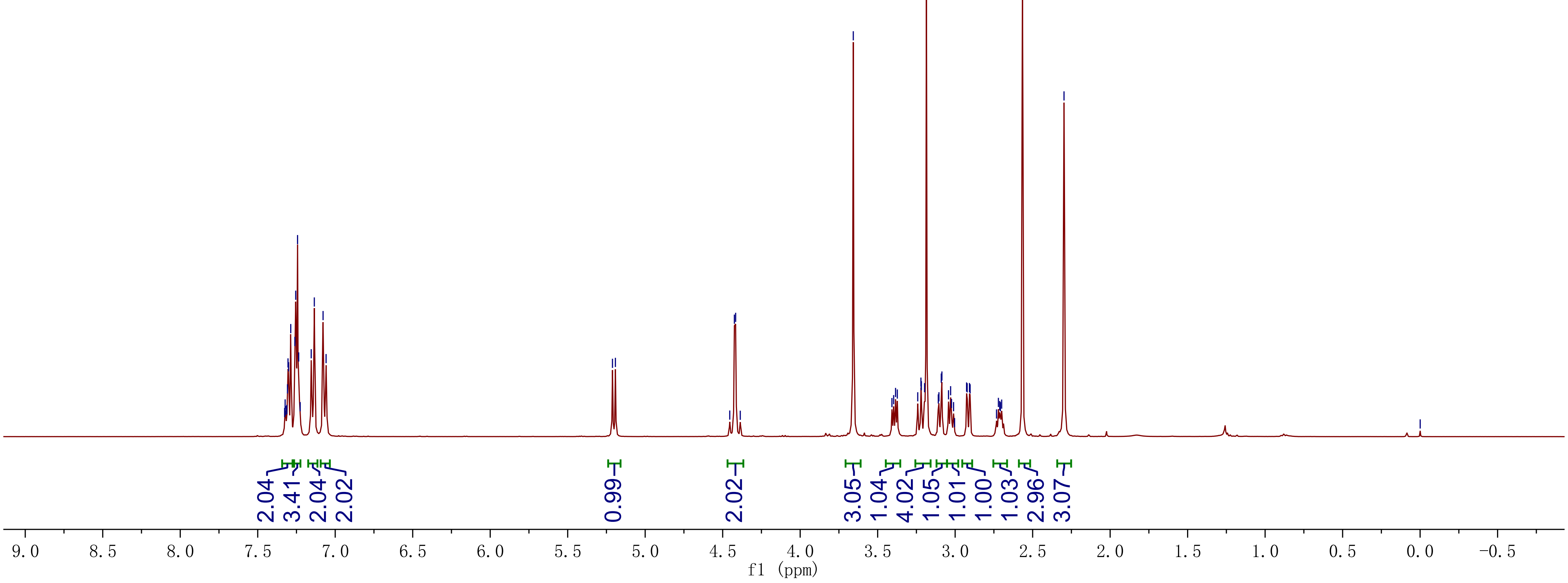

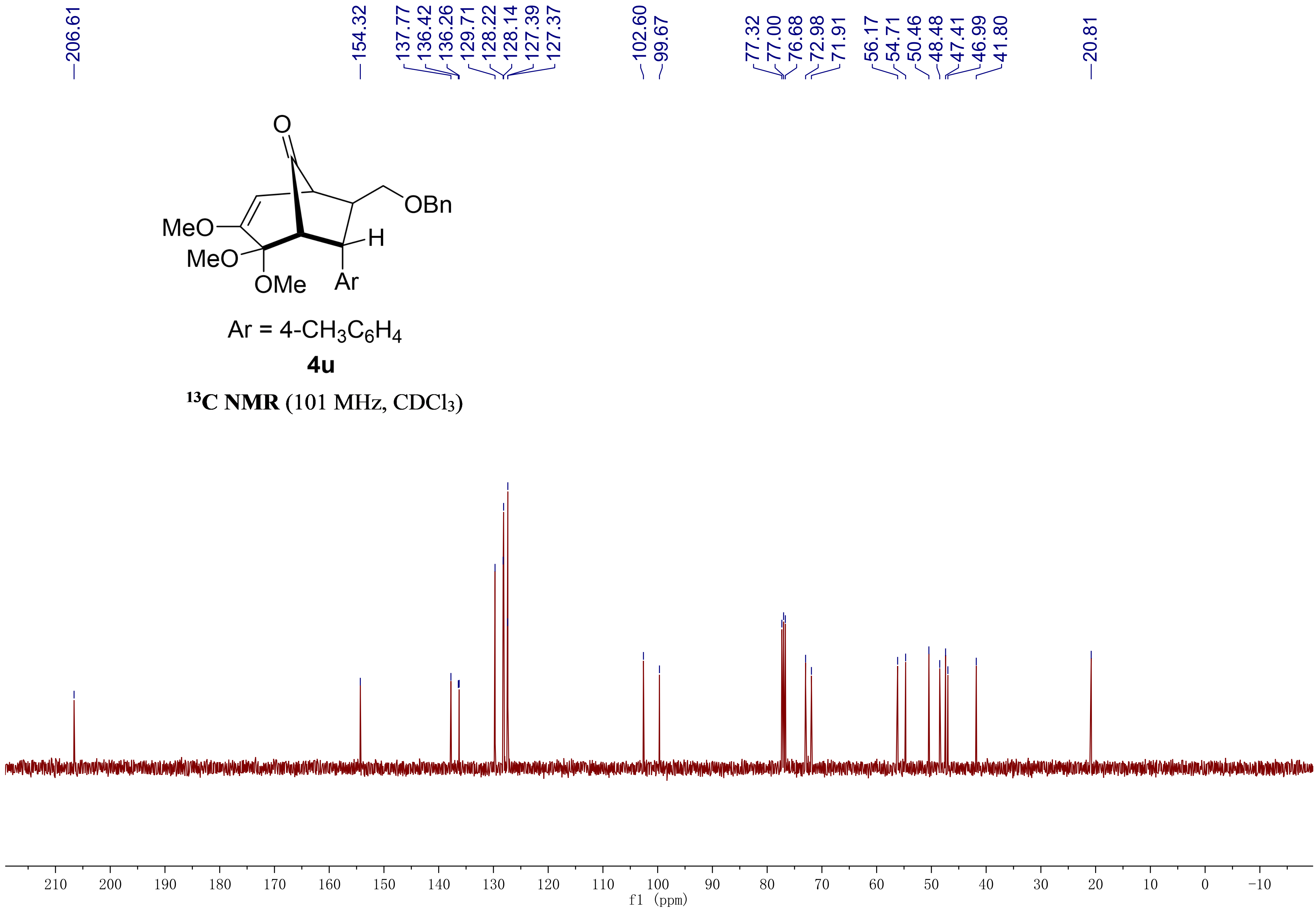
206.61
154.32
137.77
136.42
136.26
129.71
128.22
128.14
127.39
127.37
102.60
99.67
77.32
77.00
76.68
72.98
71.91
56.17
54.71
50.46
48.48
47.41
46.99
41.80
20.81
O
OBn
MeO
H
MeO
OMe
Ar
Ar = 4-$CH_3C_6H_4$
4u
^{13}C NMR (101 MHz, $CDCl_3$)
210
200
190
180
170
160
150
140
130
120
110
100
90
80
70
60
50
40
30
20
10
0
−10
f1 (ppm)

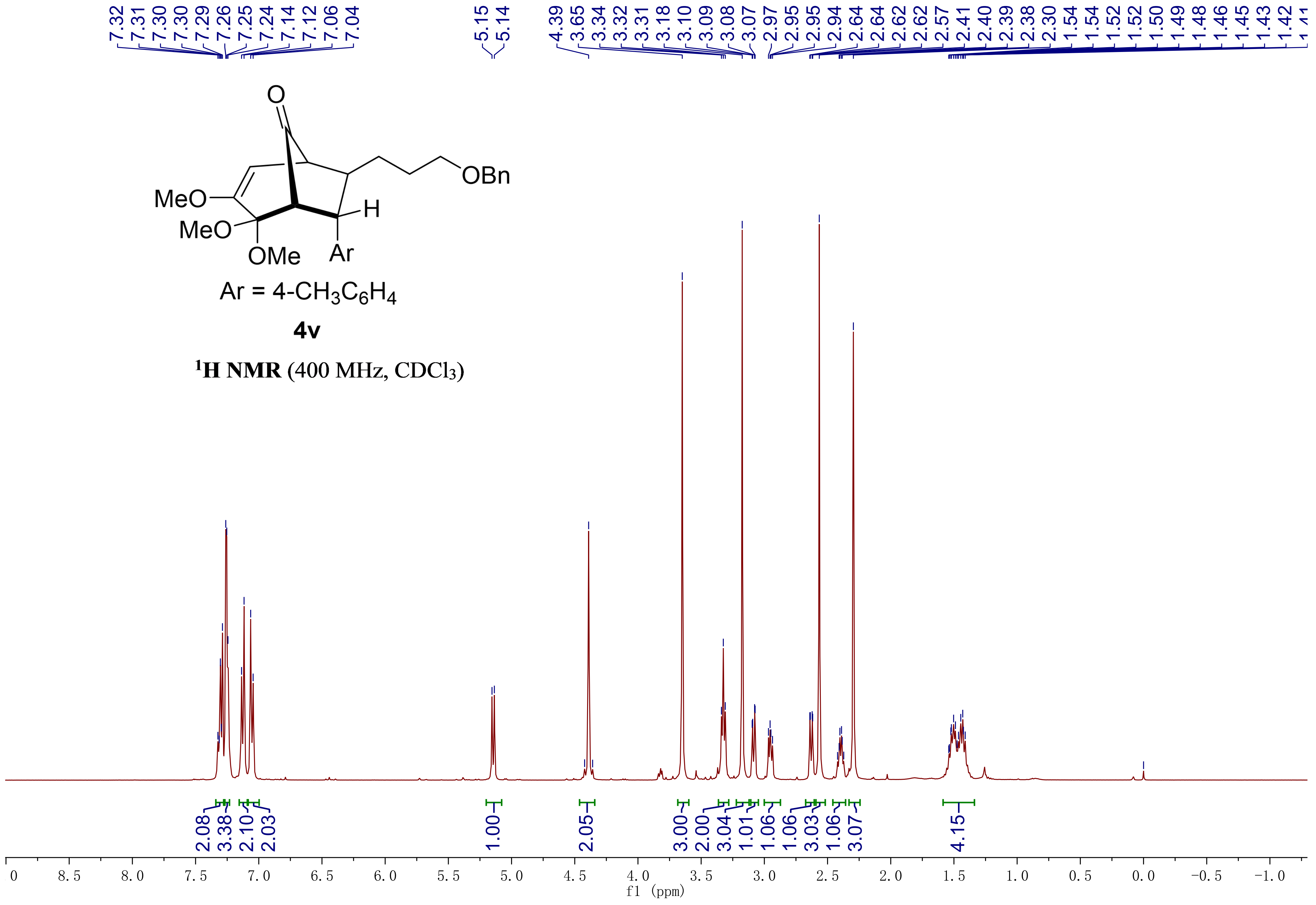

7.32 7.31 7.30 7.30 7.29 7.26 7.25 7.24 7.14 7.12 7.06 7.04
5.15 5.14
4.39 3.65 3.34 3.32 3.31 3.18 3.10 3.09 3.08 3.07 2.97 2.95 2.95 2.94 2.64 2.64 2.62 2.62 2.57 2.41 2.40 2.39 2.38 2.30 1.54 1.54 1.52 1.52 1.50 1.49 1.48 1.46 1.45 1.43 1.42
O
OBn
MeO
MeO
OMe
H
Ar
Ar = 4-CH3C6H4
4v
1H NMR (400 MHz, CDCl3)
2.08 3.38 2.10 2.03
1.00
2.05
3.00 2.00 3.04 1.01 1.06 1.06 3.03 1.06 3.07
4.15
8.5 8.0 7.5 7.0 6.5 6.0 5.5 5.0 4.5 4.0 3.5 3.0 2.5 2.0 1.5 1.0 0.5 0.0 -0.5 -1.0
f1 (ppm)

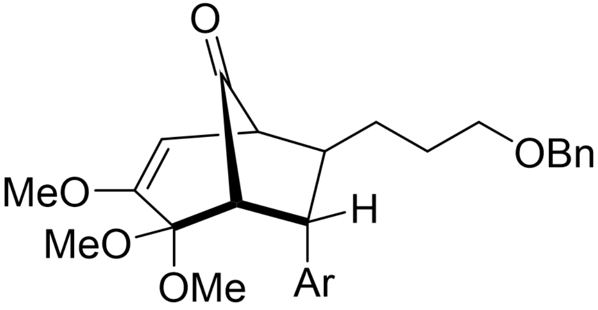

Ar = 4-$CH_3C_6H_4$

4v

^{13}C NMR (101 MHz, $CDCl_3$)

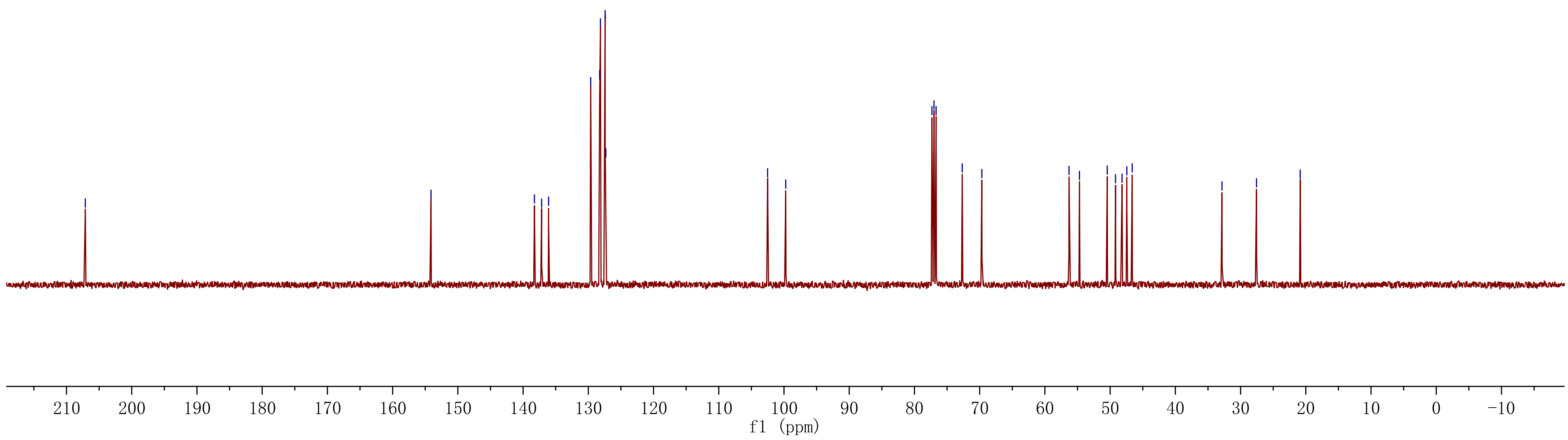

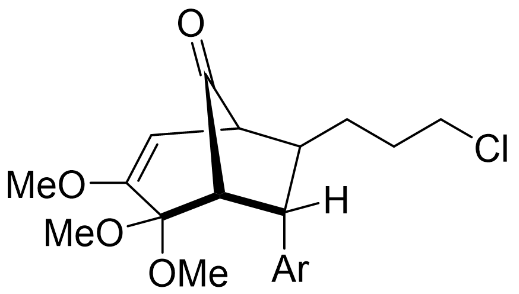

Ar = 4-$CH_3C_6H_4$

4w

[1]**H NMR** (400 MHz, $CDCl_3$)

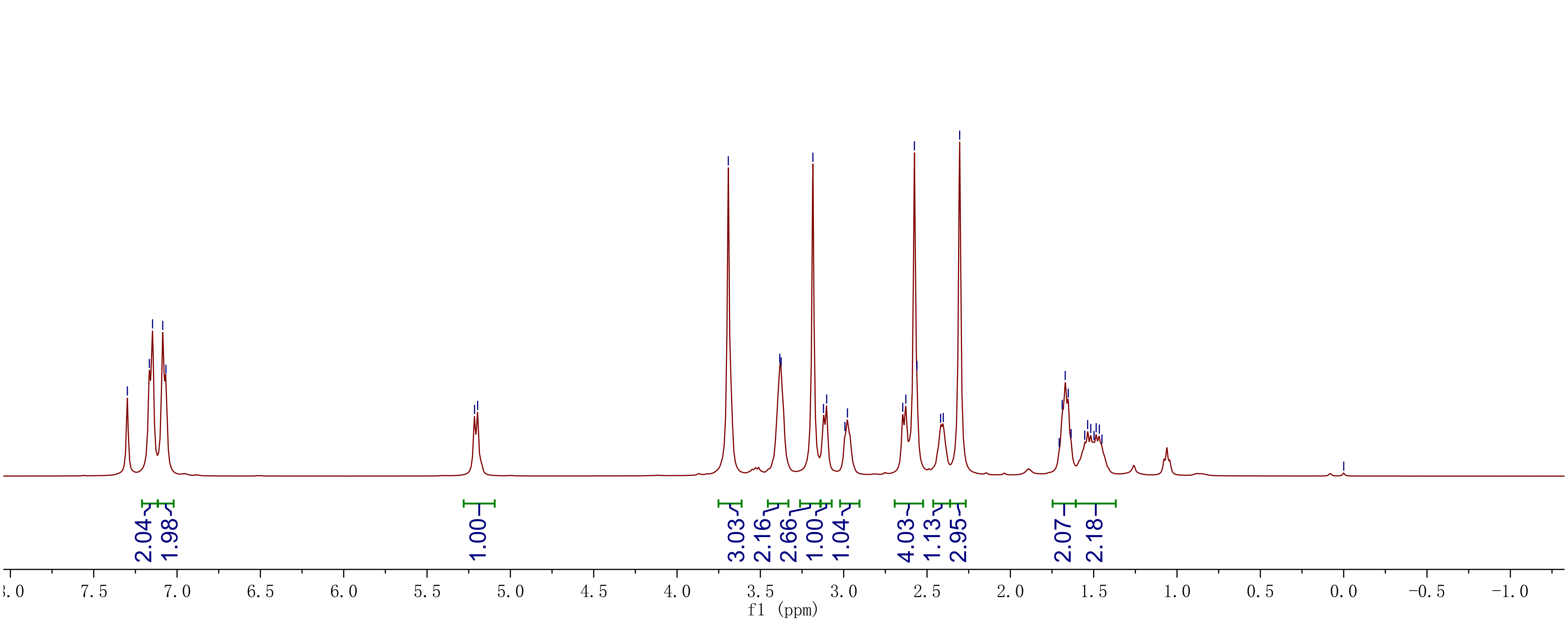

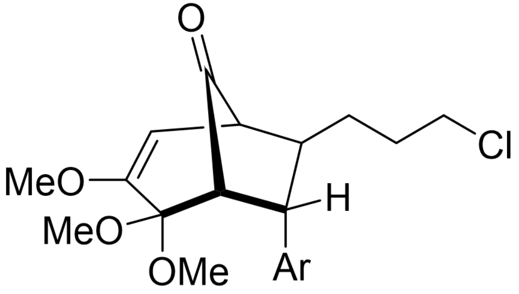

Ar = 4-$CH_3C_6H_4$

4w

^{13}C NMR (101 MHz, $CDCl_3$)

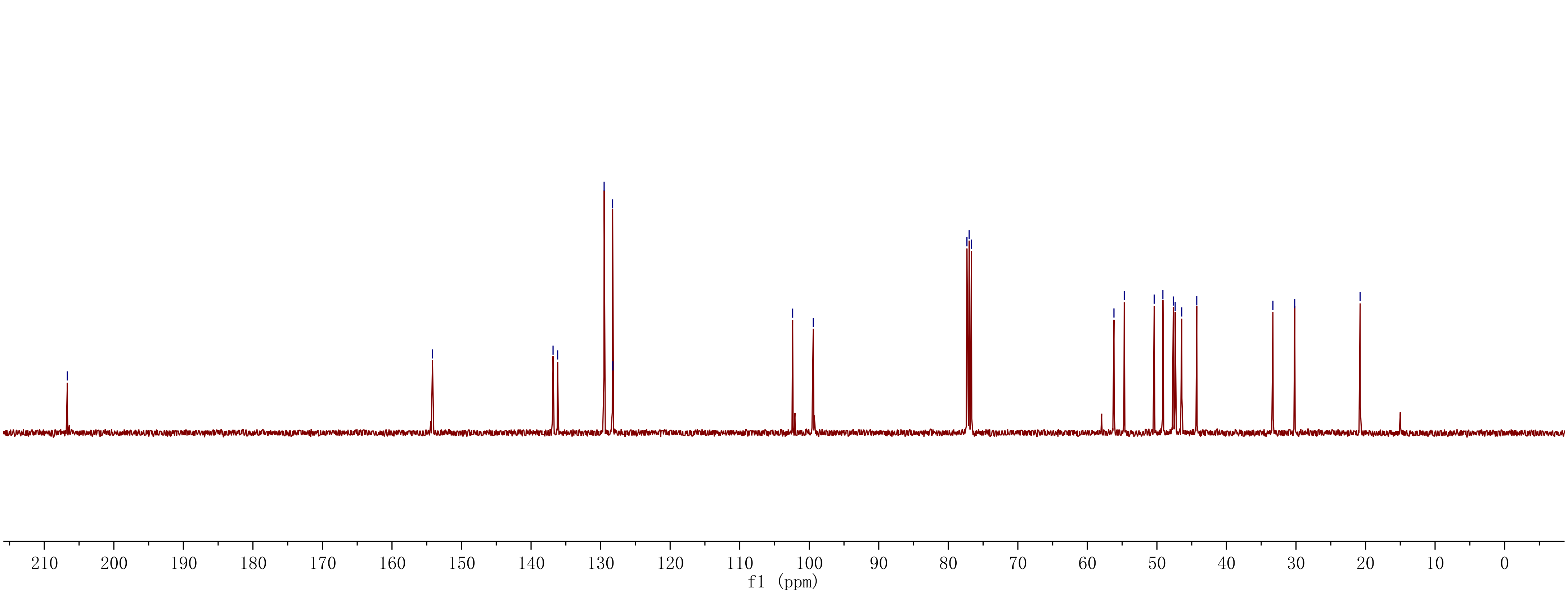

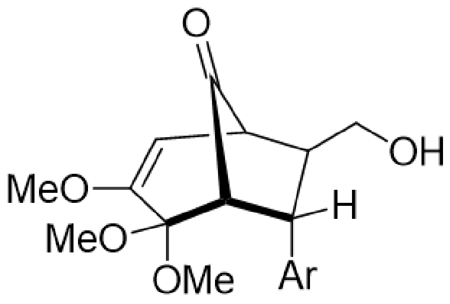

Ar = 4-$CH_3C_6H_4$

4x

^{1}H NMR (400 MHz, $CDCl_3$)

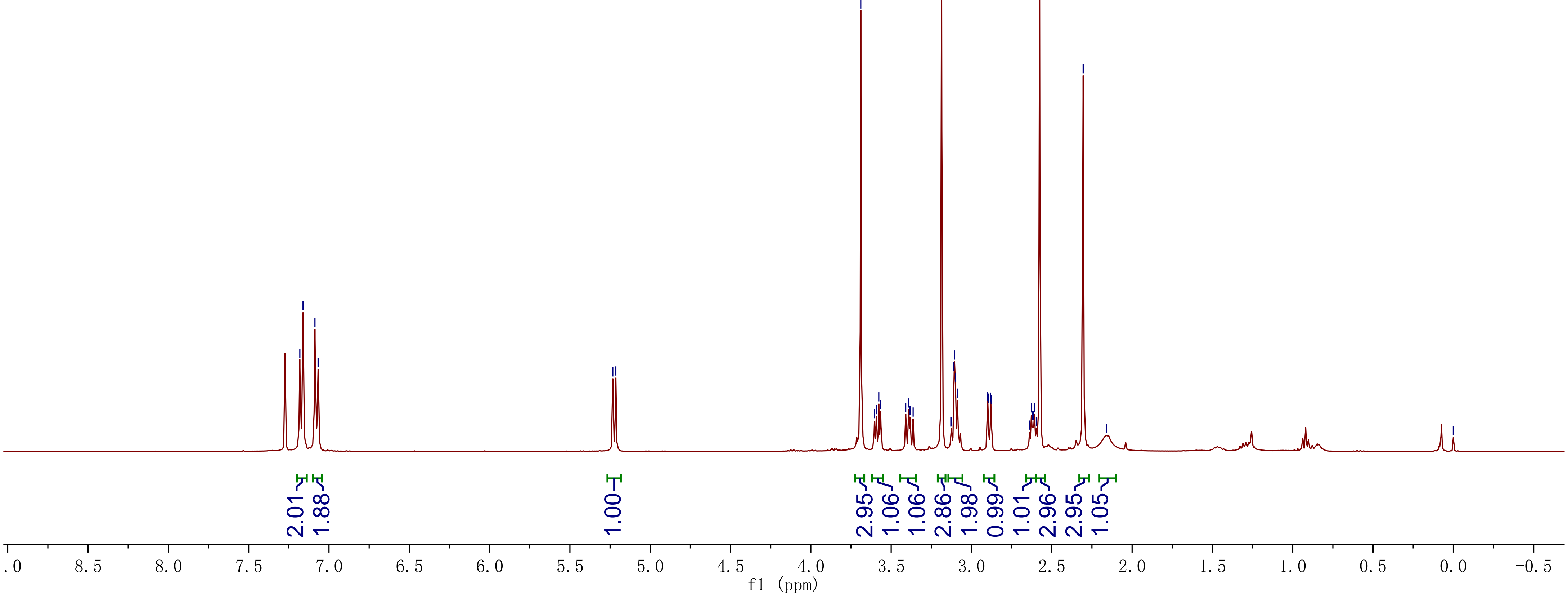

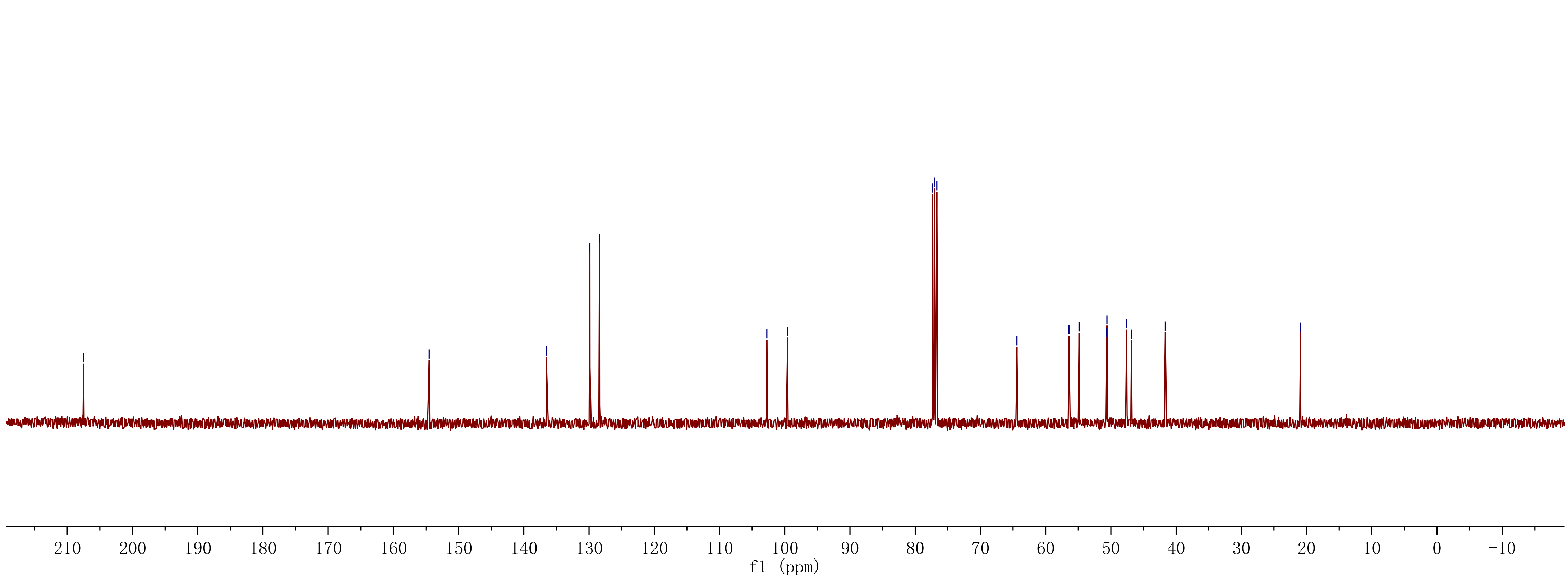

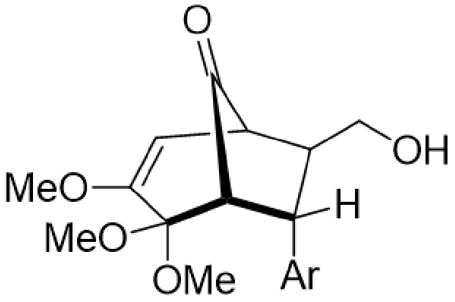

Ar = 4-$CH_3C_6H_4$

4x

^{13}C NMR (101 MHz, $CDCl_3$)

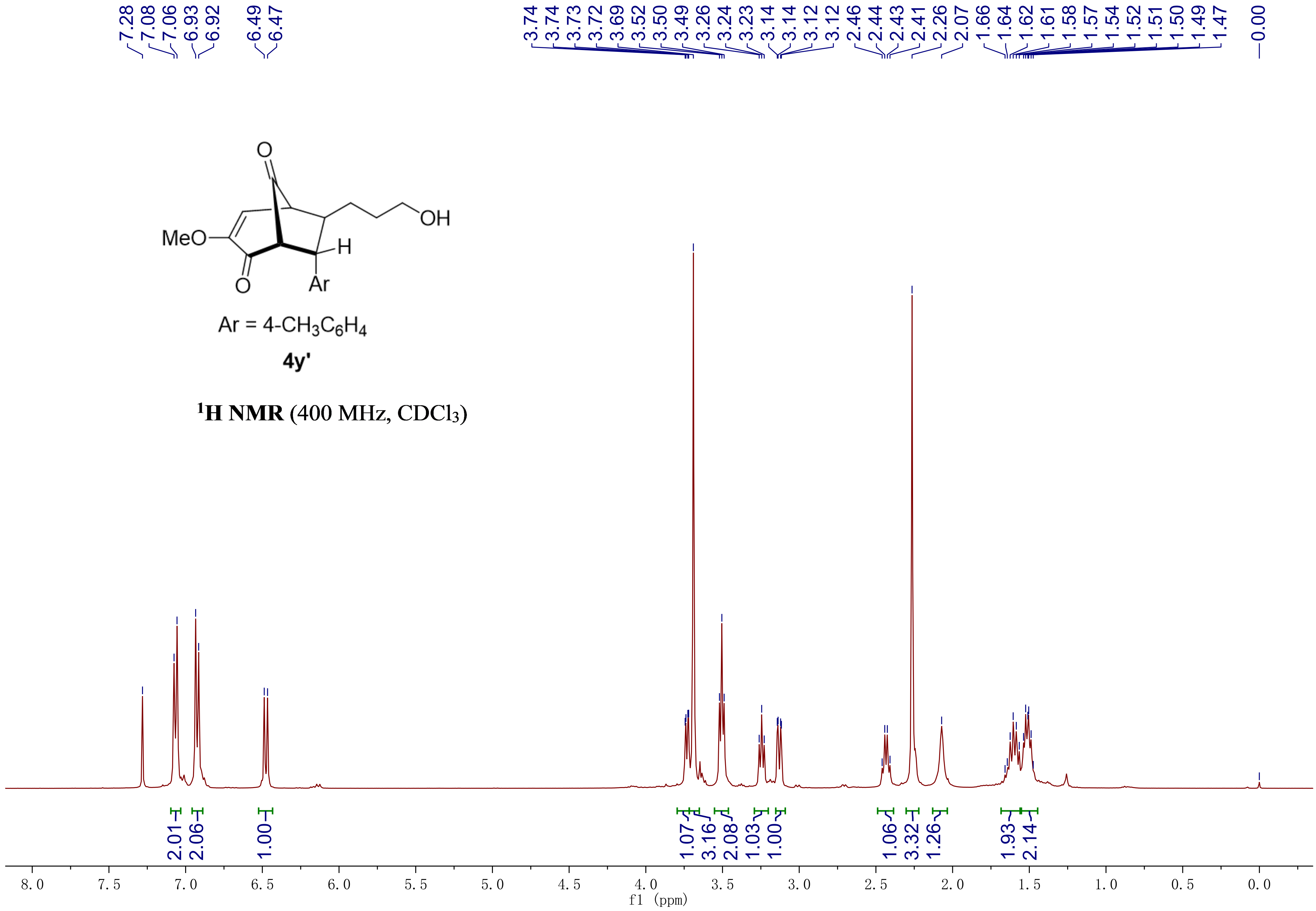

7.28
7.08
7.06
6.93
6.92
6.49
6.47
3.74
3.74
3.73
3.72
3.69
3.52
3.50
3.49
3.26
3.24
3.23
3.14
3.14
3.12
3.12
2.46
2.44
2.43
2.41
2.26
2.07
1.66
1.64
1.62
1.61
1.58
1.57
1.54
1.52
1.51
1.50
1.49
1.47
0.00
MeO
O
O
OH
H
Ar
Ar = 4-CH3C6H4
4y'
1H NMR (400 MHz, CDCl3)
2.01
2.06
1.00
1.07
3.16
2.08
1.03
1.00
1.06
3.32
1.26
1.93
2.14
8.0
7.5
7.0
6.5
6.0
5.5
5.0
4.5
4.0
3.5
3.0
2.5
2.0
1.5
1.0
0.5
0.0
f1 (ppm)

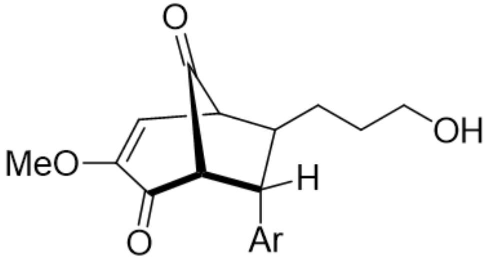

Ar = 4-$CH_3C_6H_4$

4y'

^{13}C NMR (101 MHz, $CDCl_3$)

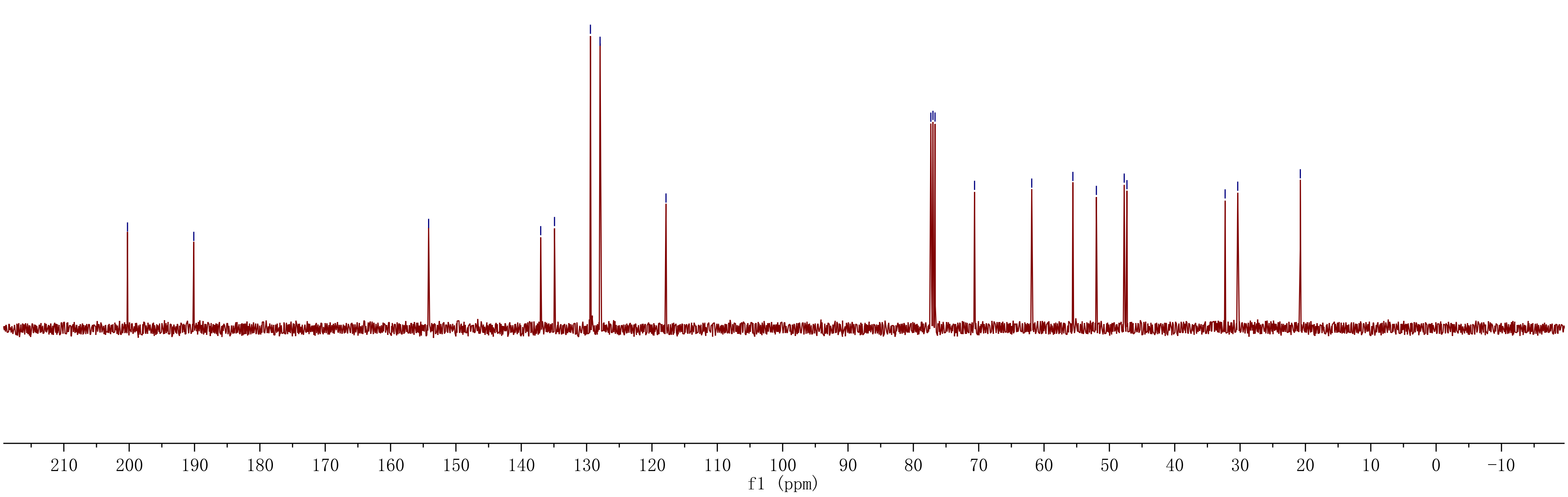

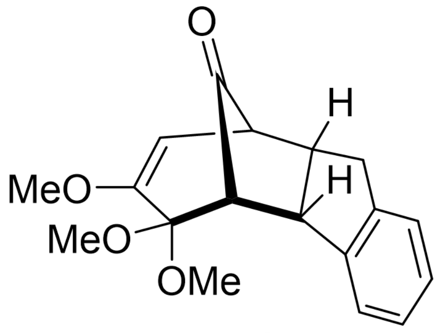

4z

^{1}H NMR (400 MHz, $CDCl_3$)

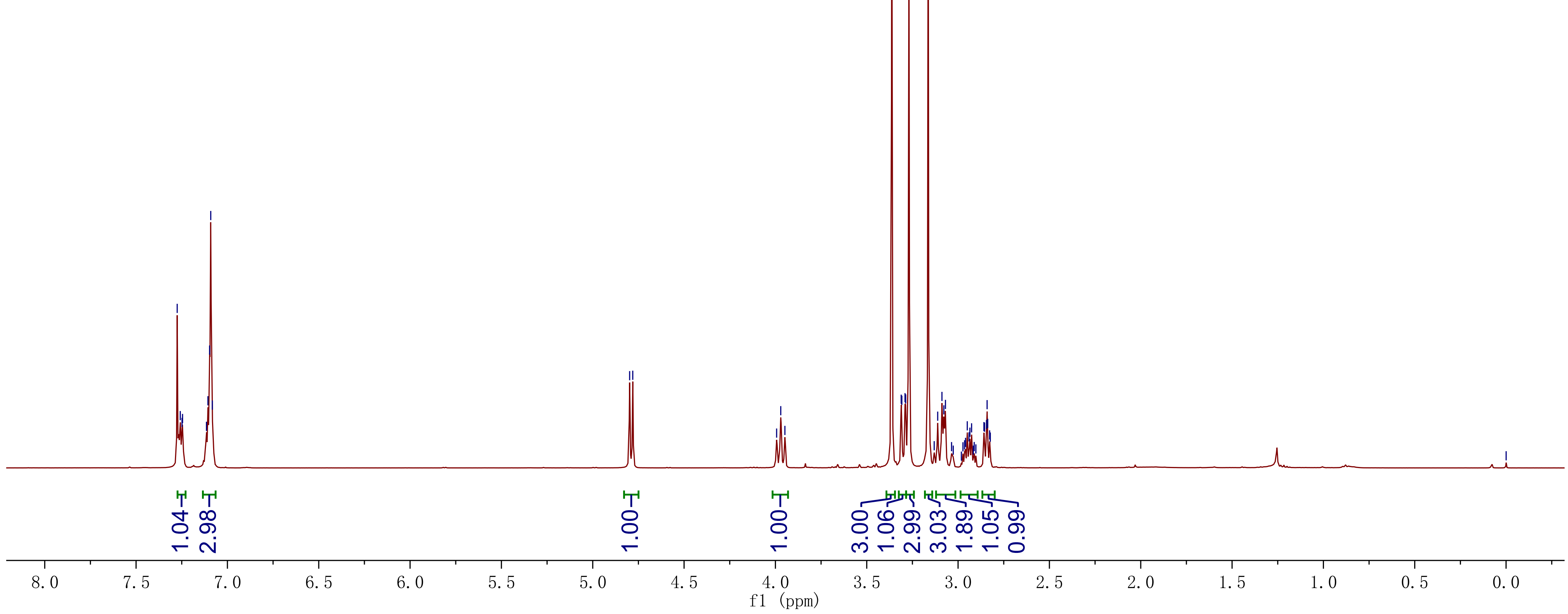

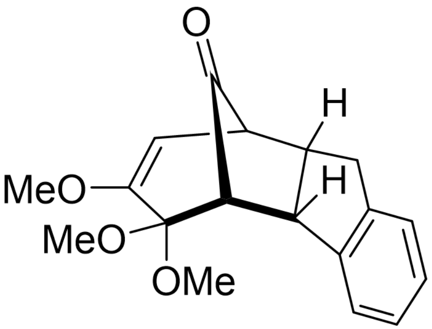

4z

^{13}C NMR (101 MHz, $CDCl_3$)

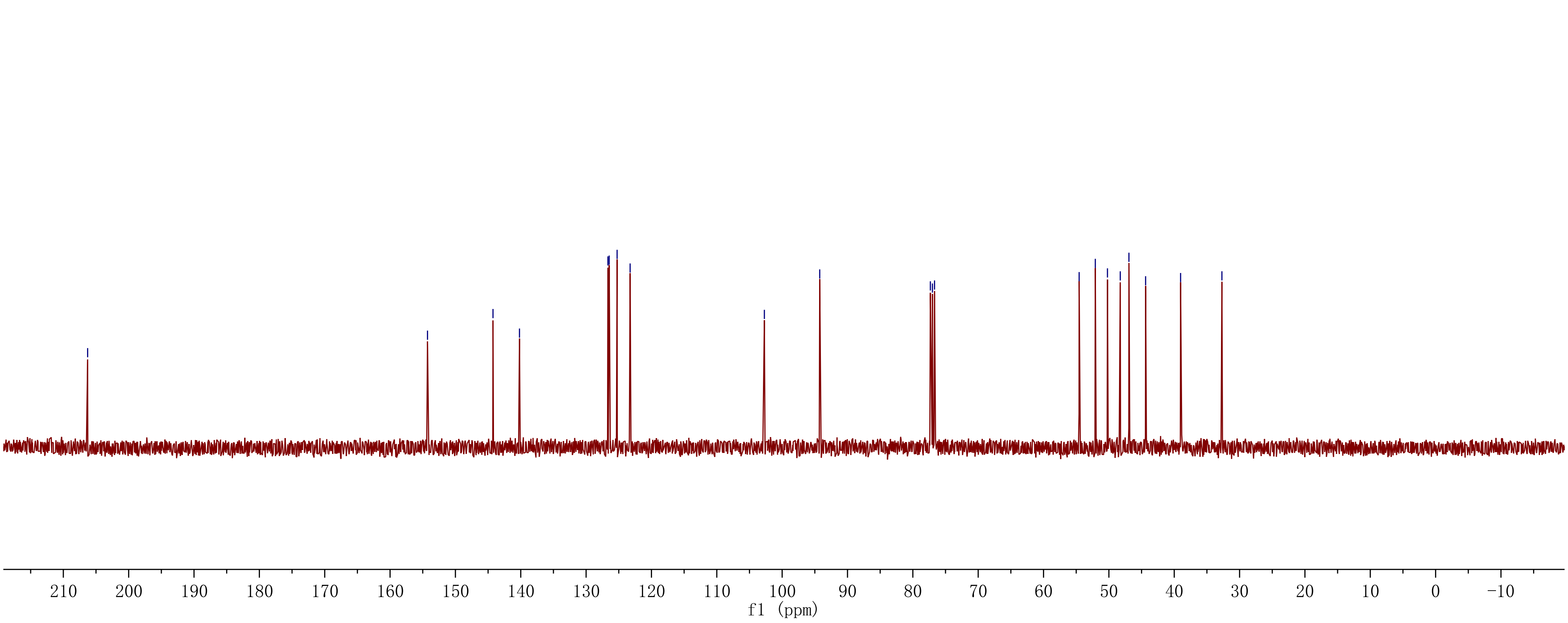

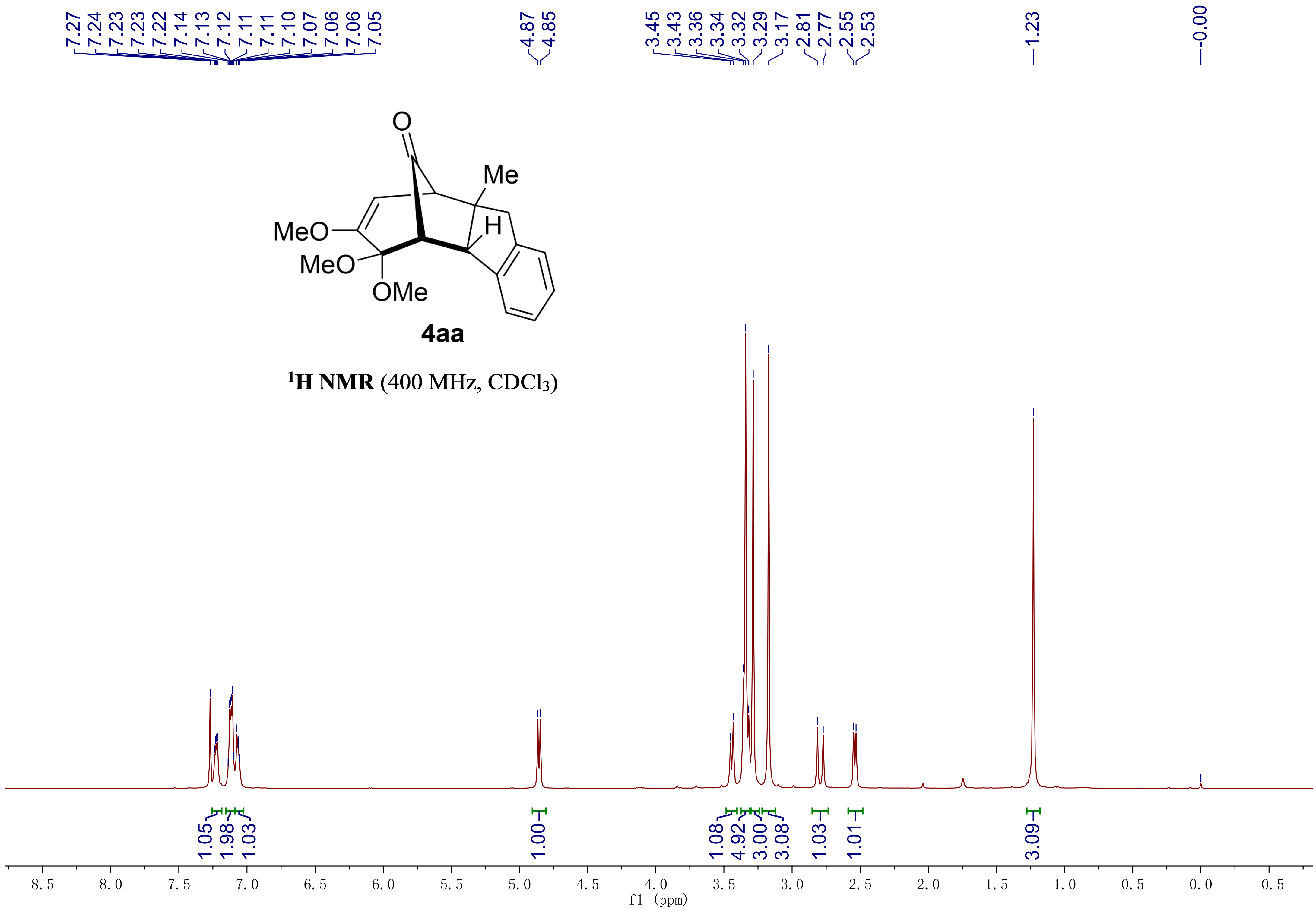

7.27 7.24 7.23 7.23 7.22 7.14 7.13 7.12 7.11 7.11 7.10 7.07 7.06 7.06 7.05
4.87 4.85
3.45 3.43 3.36 3.34 3.32 3.29 3.17 2.81 2.77 2.55 2.53
1.23
-0.00
O
Me
H
MeO
MeO
OMe
4aa
^{1}H NMR (400 MHz, CDCl$_3$)
1.05 1.98 1.03
1.00
1.08 4.92 3.00 3.08
1.03
1.01
3.09
8.5 8.0 7.5 7.0 6.5 6.0 5.5 5.0 4.5 4.0 3.5 3.0 2.5 2.0 1.5 1.0 0.5 0.0 -0.5
f1 (ppm)

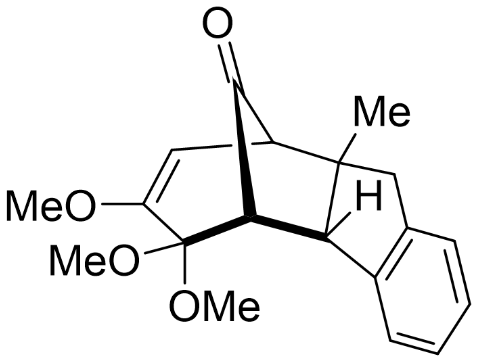

4aa

^{13}C NMR (101 MHz, $CDCl_3$)

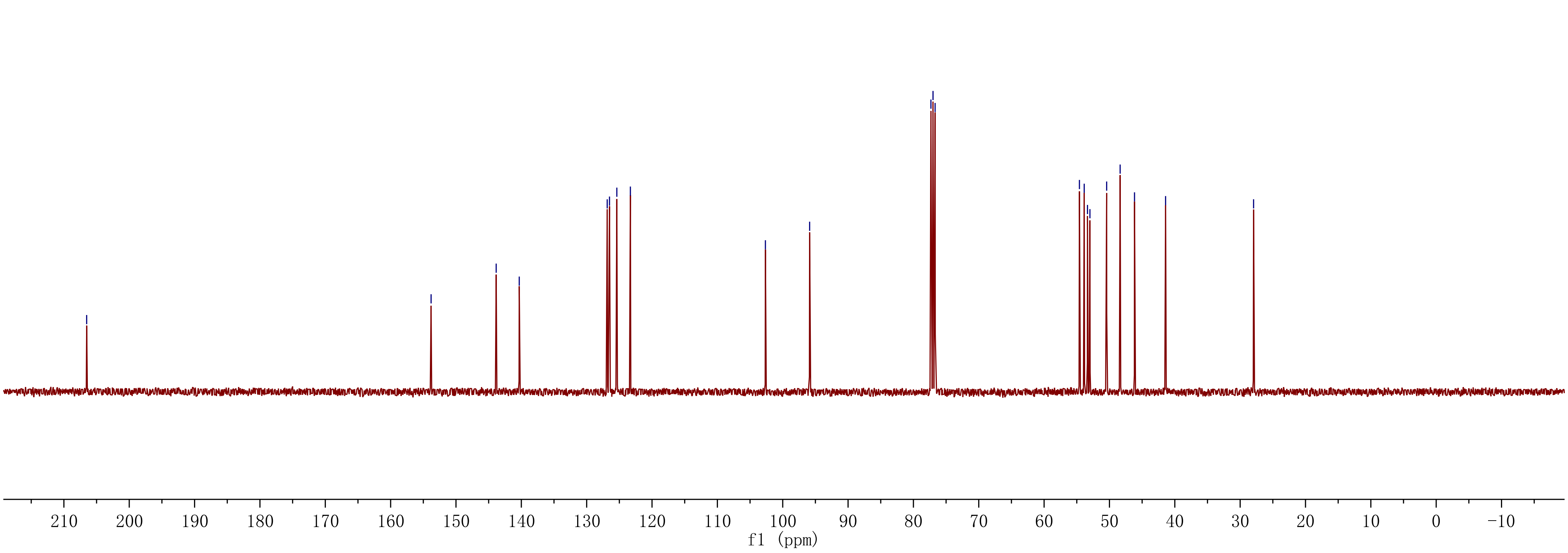

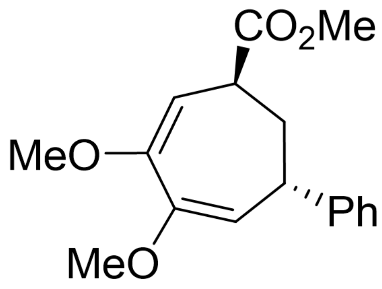

5a

¹H NMR (400 MHz, $CDCl_3$)

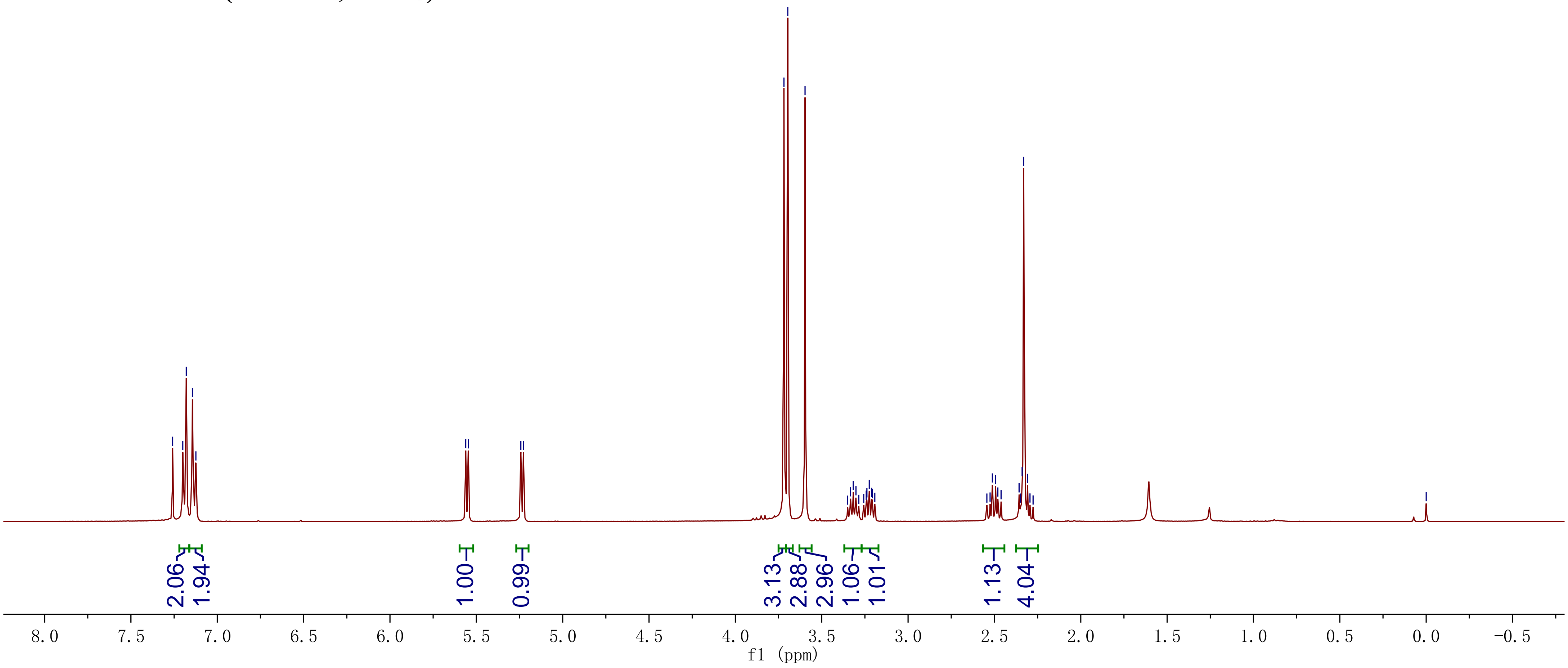

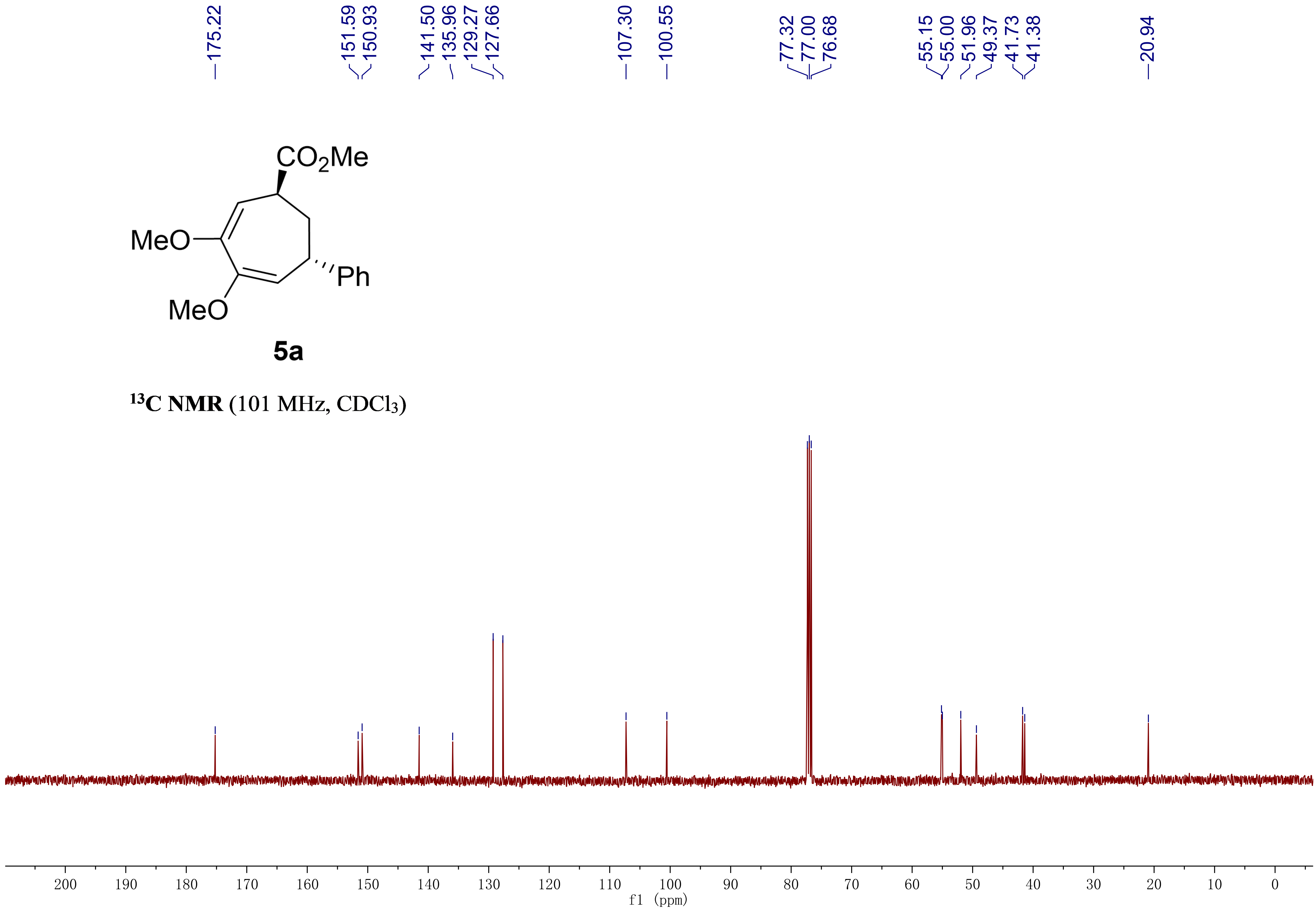
175.22
151.59
150.93
141.50
135.96
129.27
127.66
107.30
100.55
77.32
77.00
76.68
55.15
55.00
51.96
49.37
41.73
41.38
20.94
CO2Me
MeO
MeO
Ph
5a
13C NMR (101 MHz, CDCl3)
200
190
180
170
160
150
140
130
120
110
100
90
80
70
60
50
40
30
20
10
0
f1 (ppm)

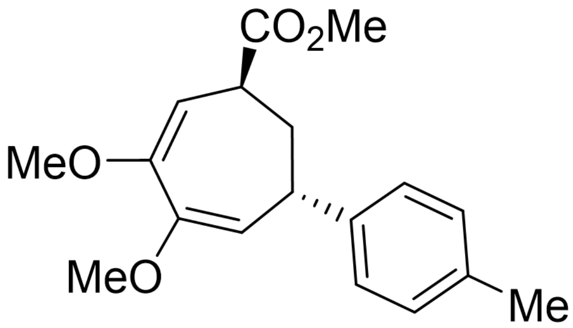

5b

^{1}H NMR (400 MHz, $CDCl_3$)

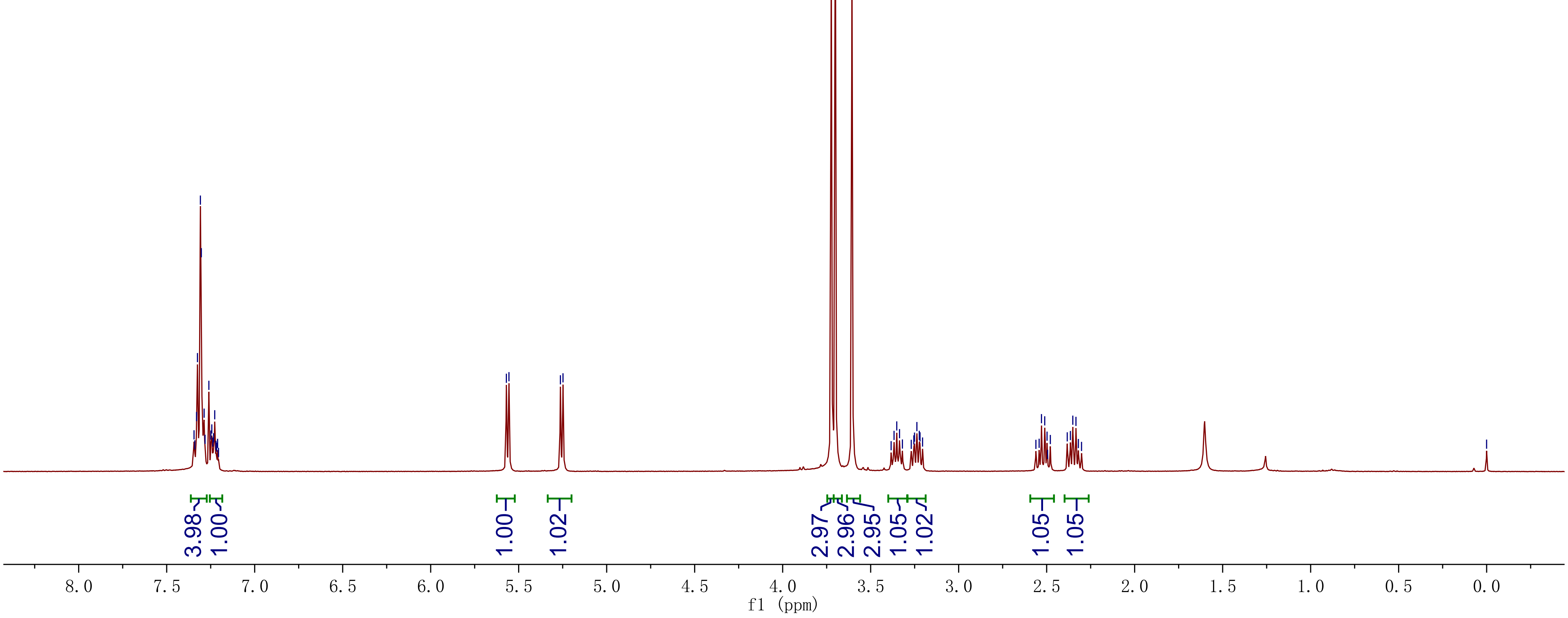

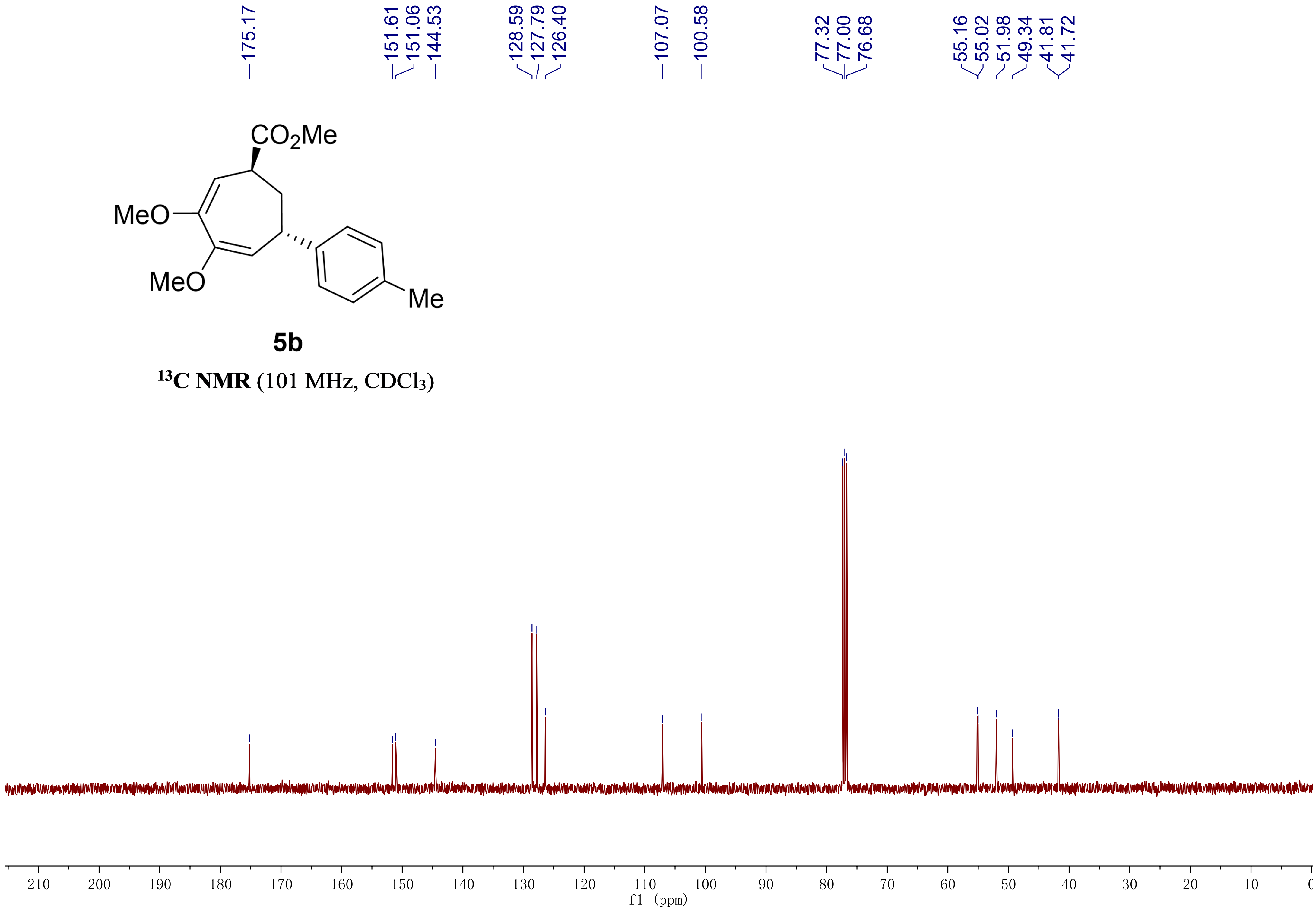

5b

^{13}C NMR (101 MHz, $CDCl_3$)

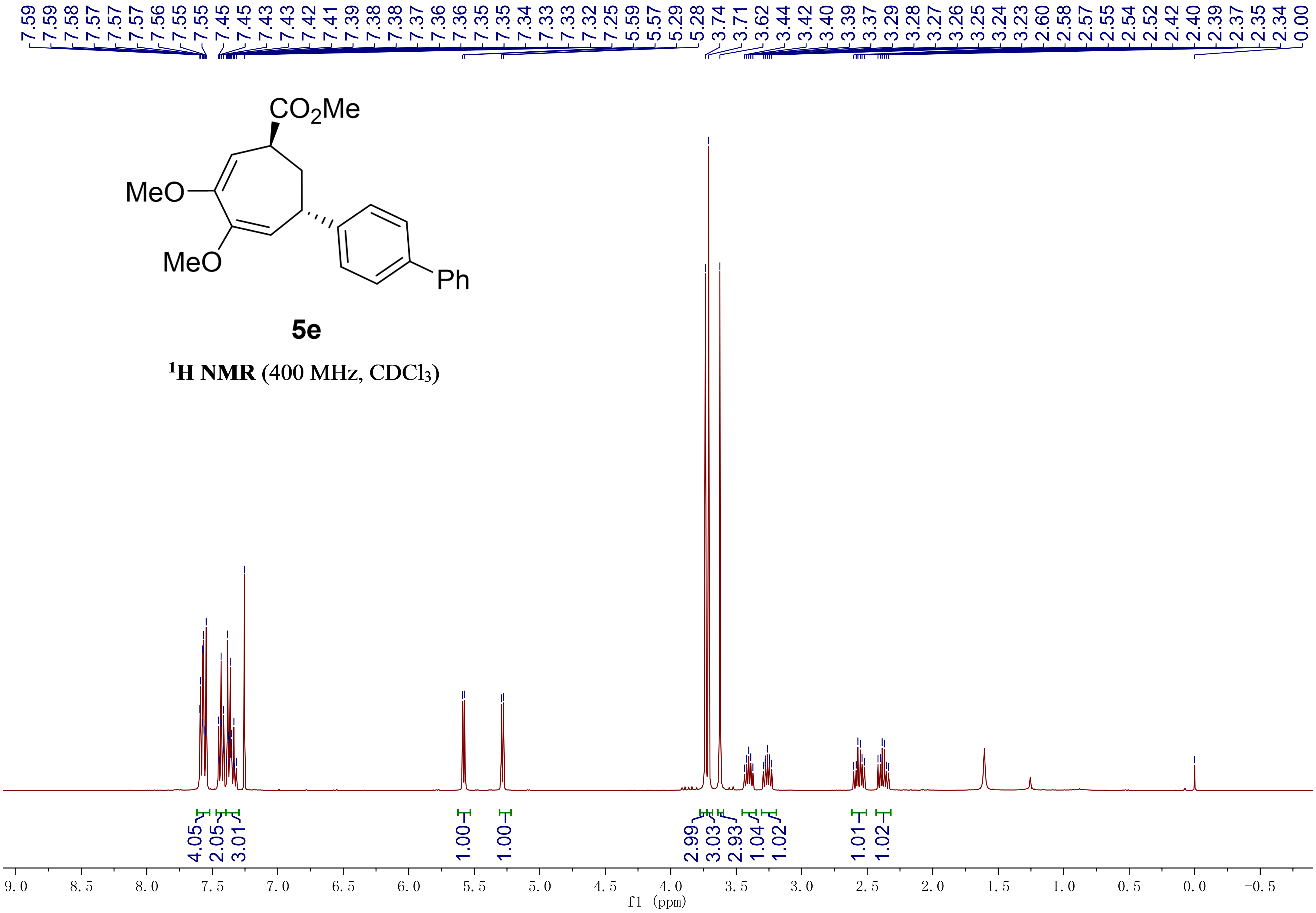

CO2Me
MeO
MeO
Ph
5e
1H NMR (400 MHz, CDCl3)
7.59 7.59 7.58 7.57 7.57 7.57 7.56 7.55 7.55 7.45 7.45 7.43 7.43 7.42 7.41 7.39 7.38 7.38 7.37 7.36 7.36 7.35 7.35 7.34 7.33 7.33 7.32 7.25 5.59 5.57 5.29 5.28 3.74 3.71 3.62 3.44 3.42 3.40 3.39 3.37 3.29 3.28 3.27 3.26 3.25 3.24 3.23 2.60 2.58 2.57 2.55 2.54 2.52 2.42 2.40 2.39 2.37 2.35 2.34 0.00
4.05 2.05 3.01 1.00 1.00 2.99 3.03 2.93 1.04 1.02 1.01 1.02
9.0 8.5 8.0 7.5 7.0 6.5 6.0 5.5 5.0 4.5 4.0 3.5 3.0 2.5 2.0 1.5 1.0 0.5 0.0 -0.5
f1 (ppm)

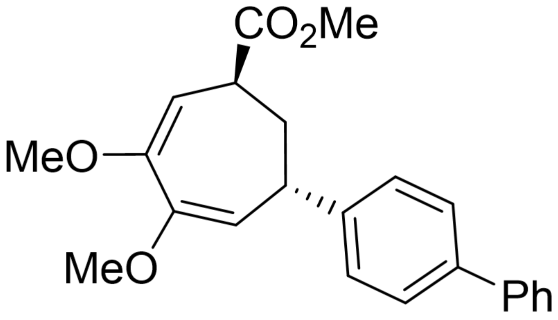

5e

^{13}C NMR (101 MHz, $CDCl_3$)

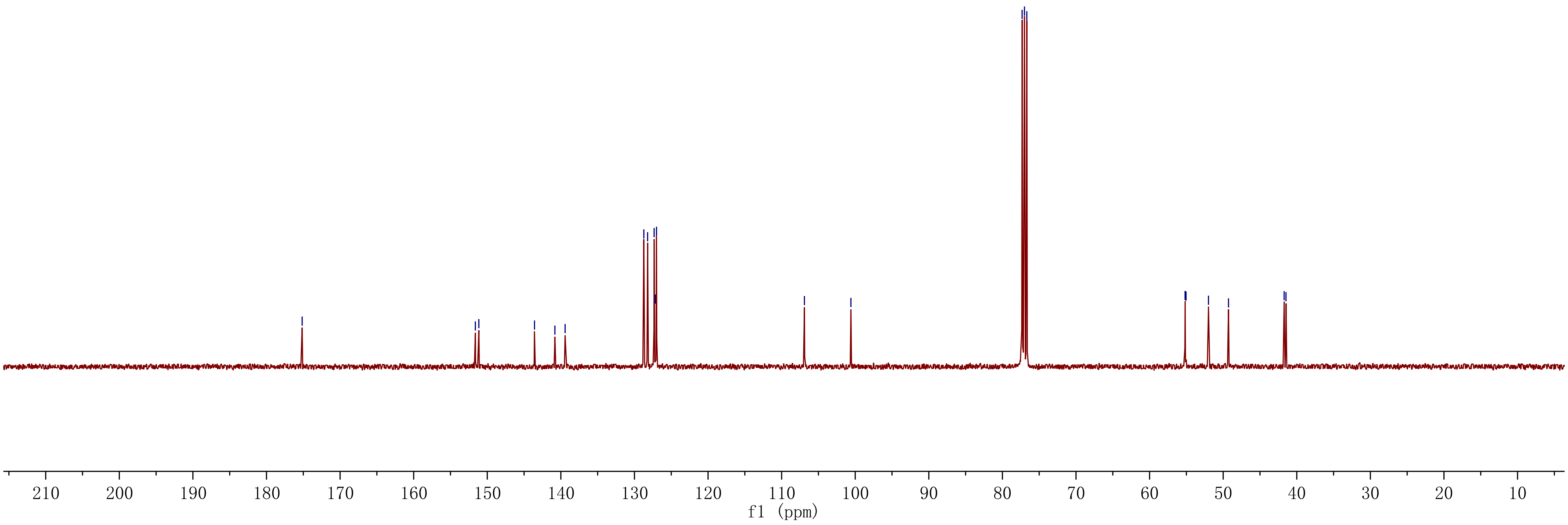

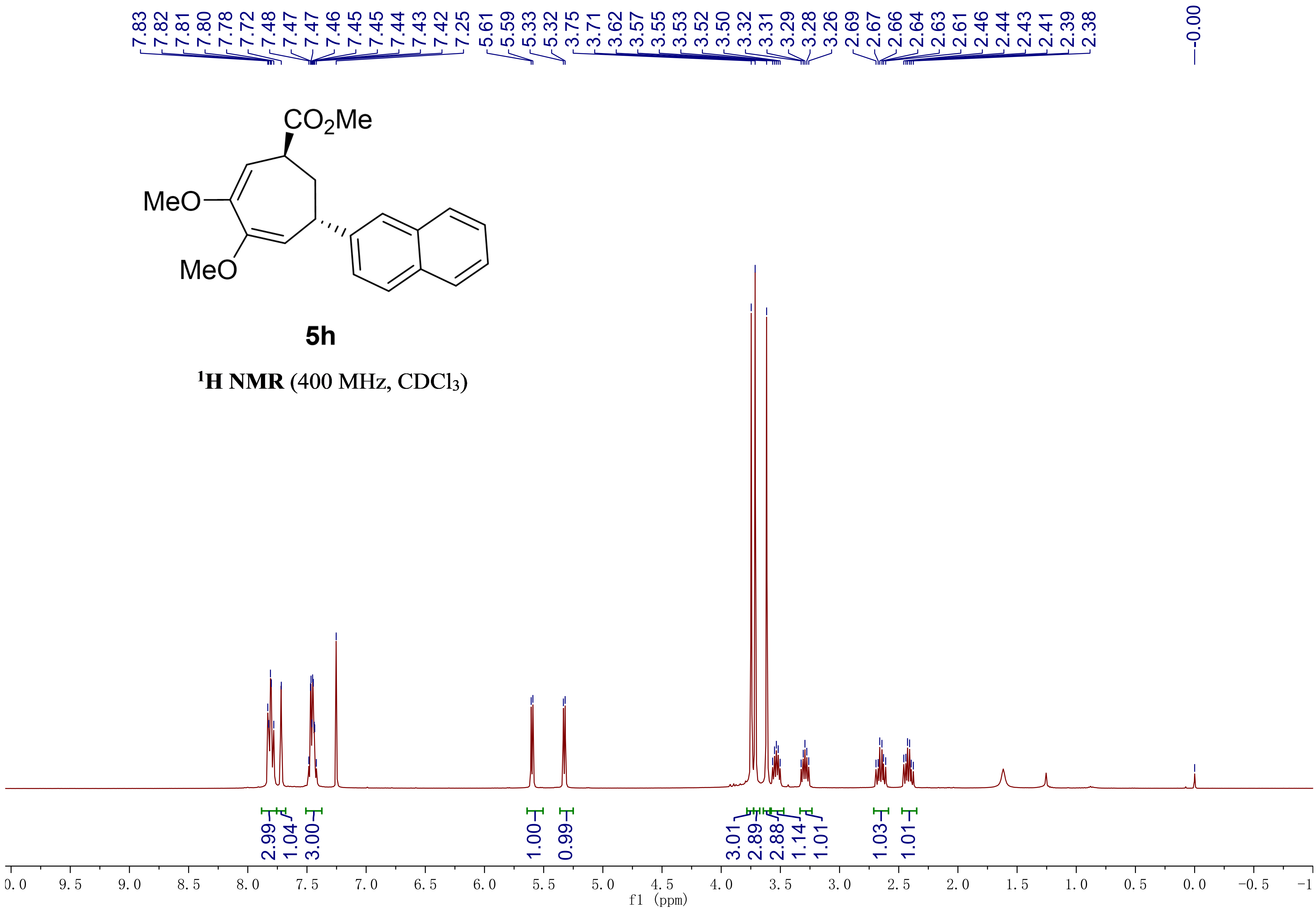
7.83
7.82
7.81
7.80
7.78
7.72
7.48
7.47
7.47
7.46
7.45
7.45
7.44
7.43
7.42
7.25
5.61
5.59
5.33
5.32
3.75
3.71
3.62
3.57
3.55
3.53
3.52
3.50
3.32
3.31
3.29
3.28
3.26
2.69
2.67
2.66
2.64
2.63
2.61
2.46
2.44
2.43
2.41
2.39
2.38
-0.00
CO_2Me
MeO
MeO
5h
^{1}H NMR (400 MHz, $CDCl_3$)
2.99
1.04
3.00
1.00
0.99
3.01
2.89
2.88
1.14
1.01
1.03
1.01
f1 (ppm)

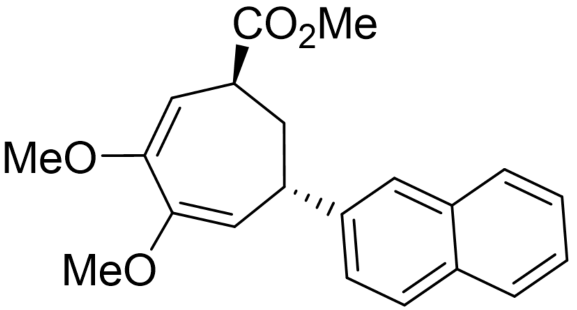

5h

^{13}C NMR (101 MHz, $CDCl_3$)

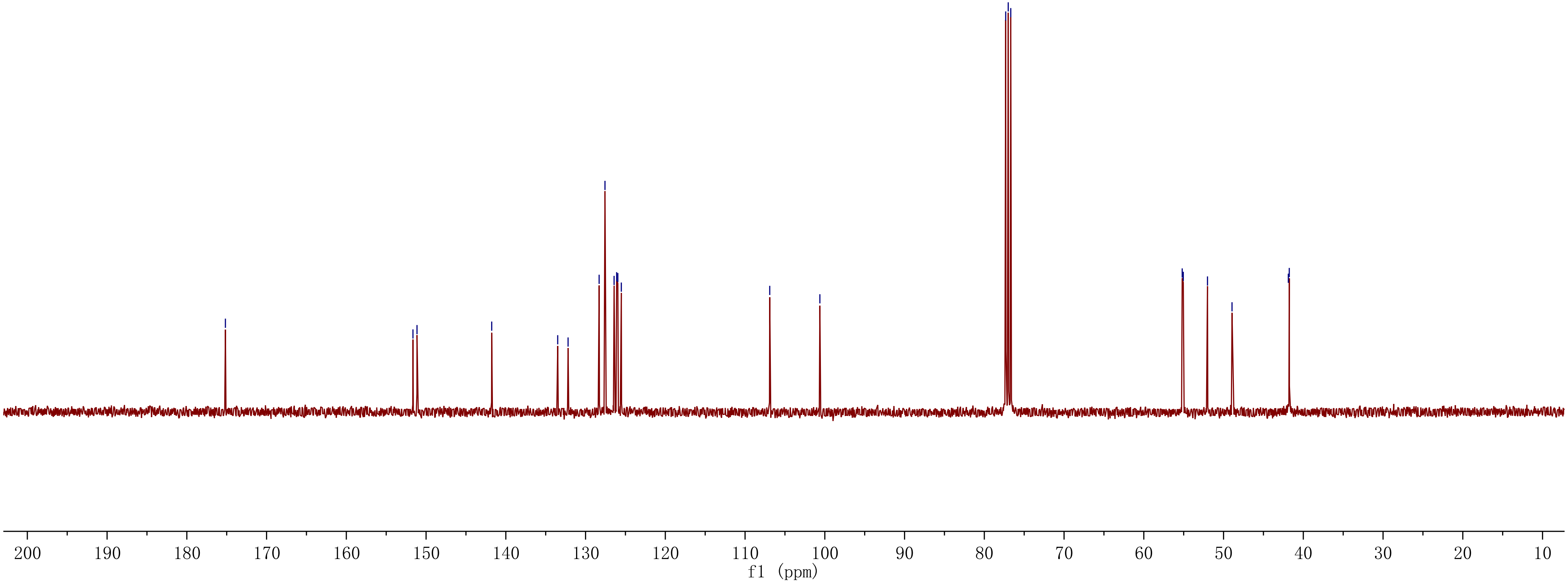

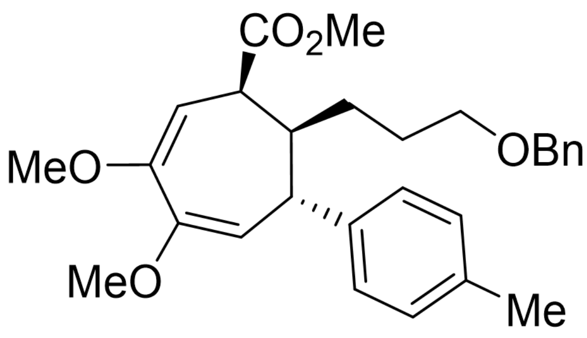

5v

¹H NMR (400 MHz, $CDCl_3$)

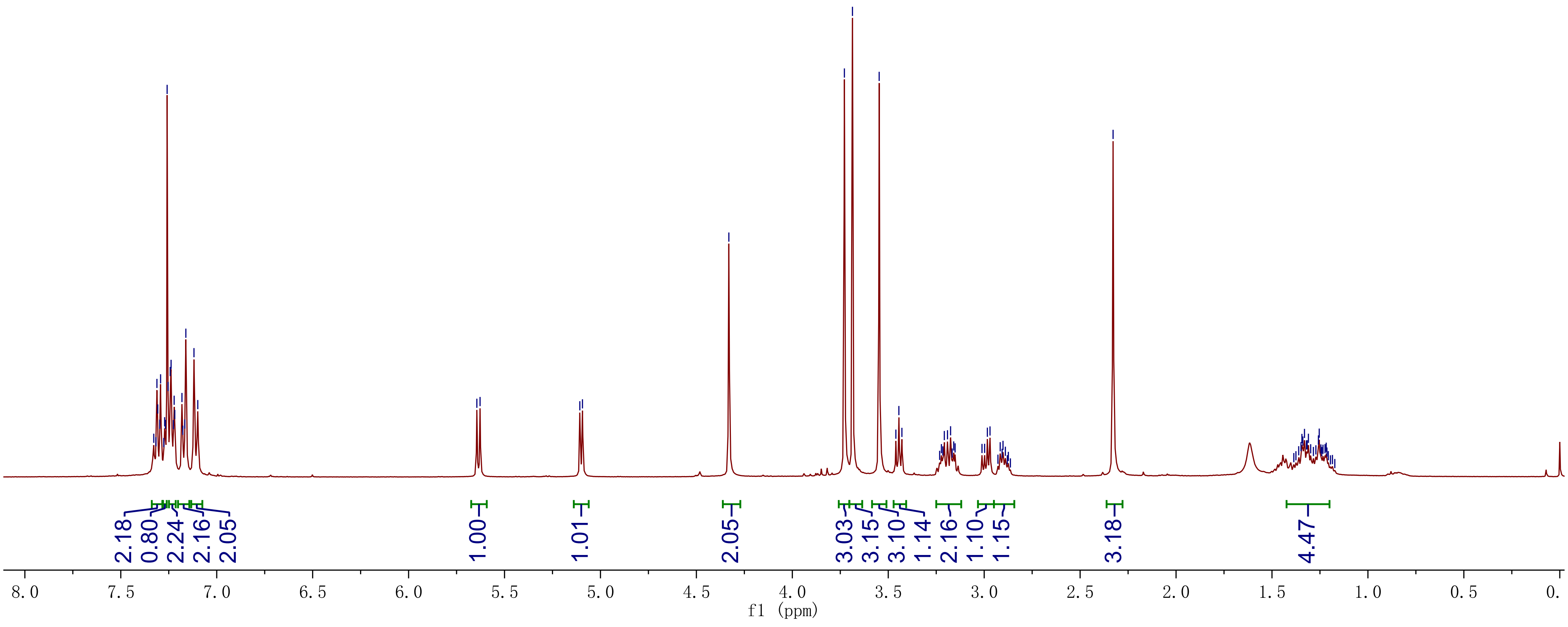

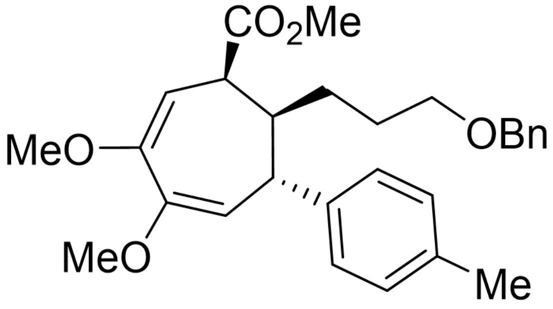

5v

^{13}C NMR (101 MHz, $CDCl_3$)

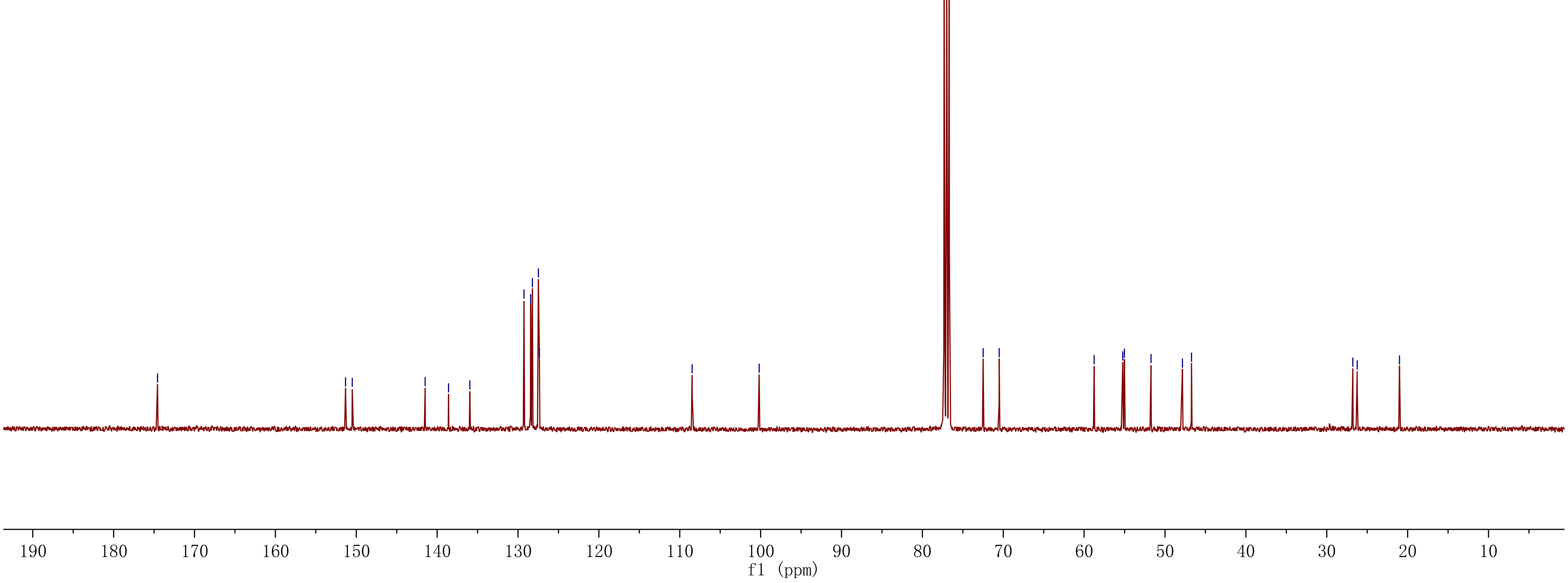

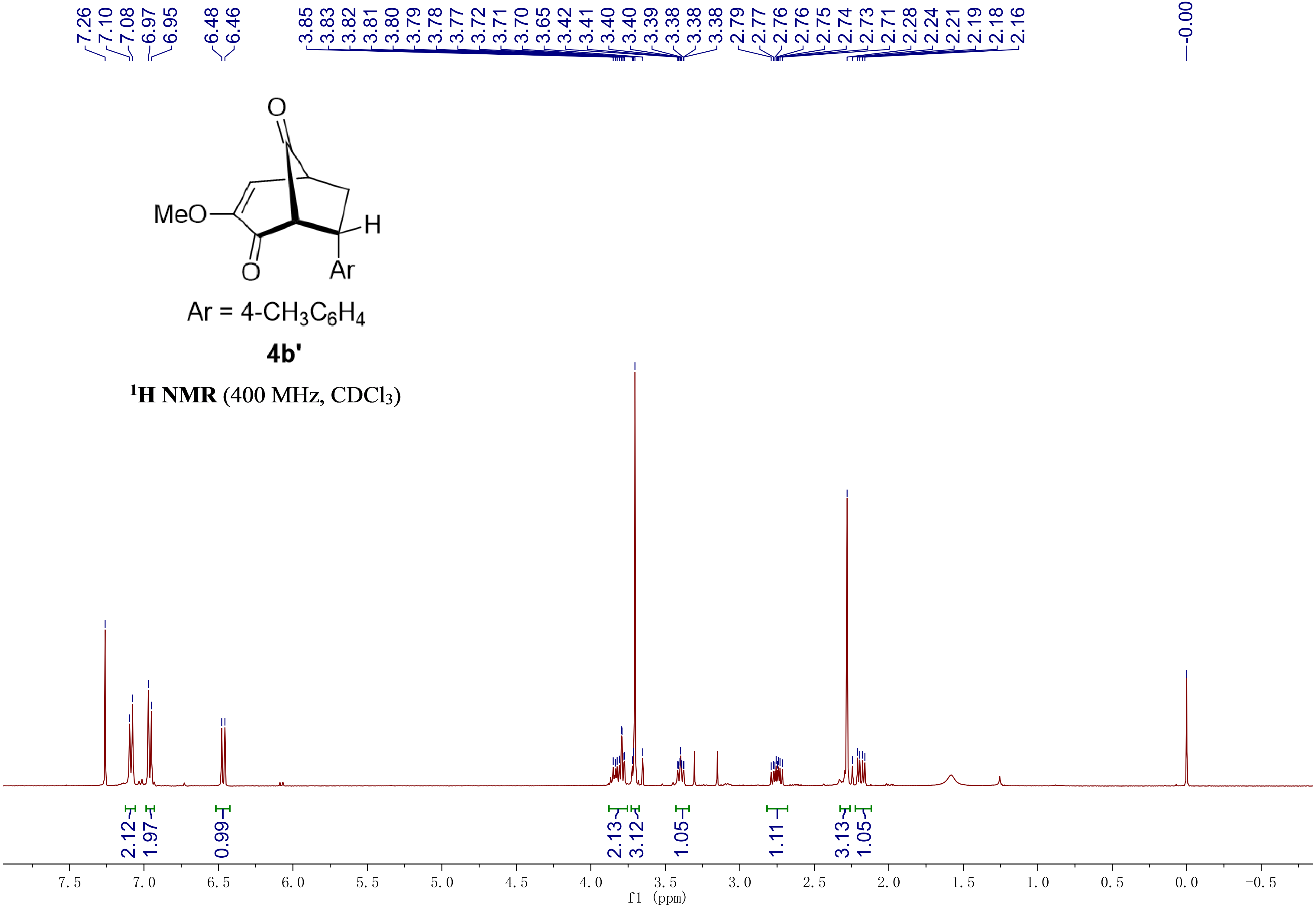
7.26
7.10
7.08
6.97
6.95
6.48
6.46
3.85
3.83
3.82
3.81
3.80
3.79
3.78
3.77
3.72
3.71
3.70
3.65
3.42
3.41
3.40
3.40
3.39
3.38
3.38
3.38
2.79
2.77
2.76
2.76
2.75
2.74
2.73
2.71
2.28
2.24
2.21
2.19
2.18
2.16
-0.00
MeO
H
Ar
Ar = 4-CH3C6H4
4b'
1H NMR (400 MHz, CDCl3)
2.12
1.97
0.99
2.13
3.12
1.05
1.11
3.13
1.05
7.5
7.0
6.5
6.0
5.5
5.0
4.5
4.0
3.5
3.0
2.5
2.0
1.5
1.0
0.5
0.0
-0.5
f1 (ppm)

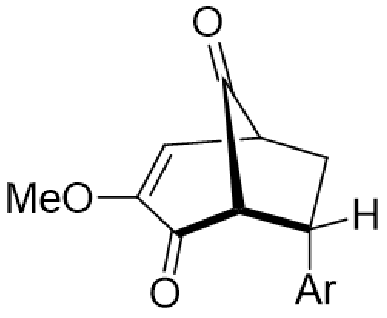

Ar = 4-$CH_3C_6H_4$

4b'

^{13}C NMR (101 MHz, $CDCl_3$)

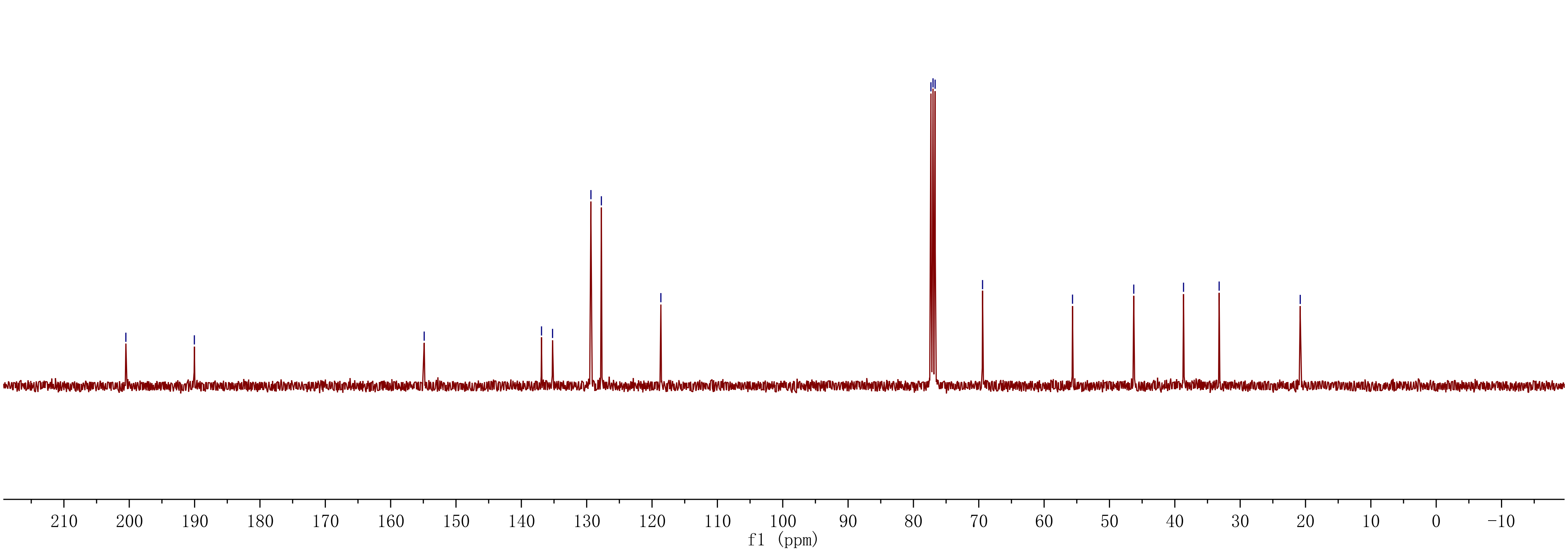

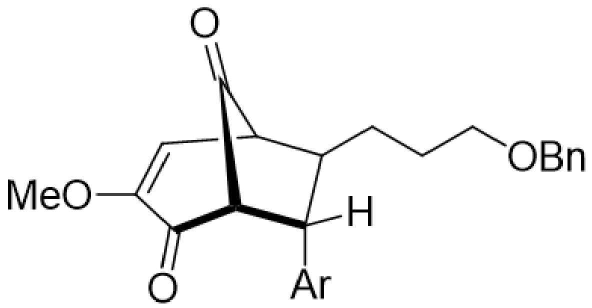

Ar = 4-$CH_3C_6H_4$

4v'

^{1}H NMR (400 MHz, $CDCl_3$)

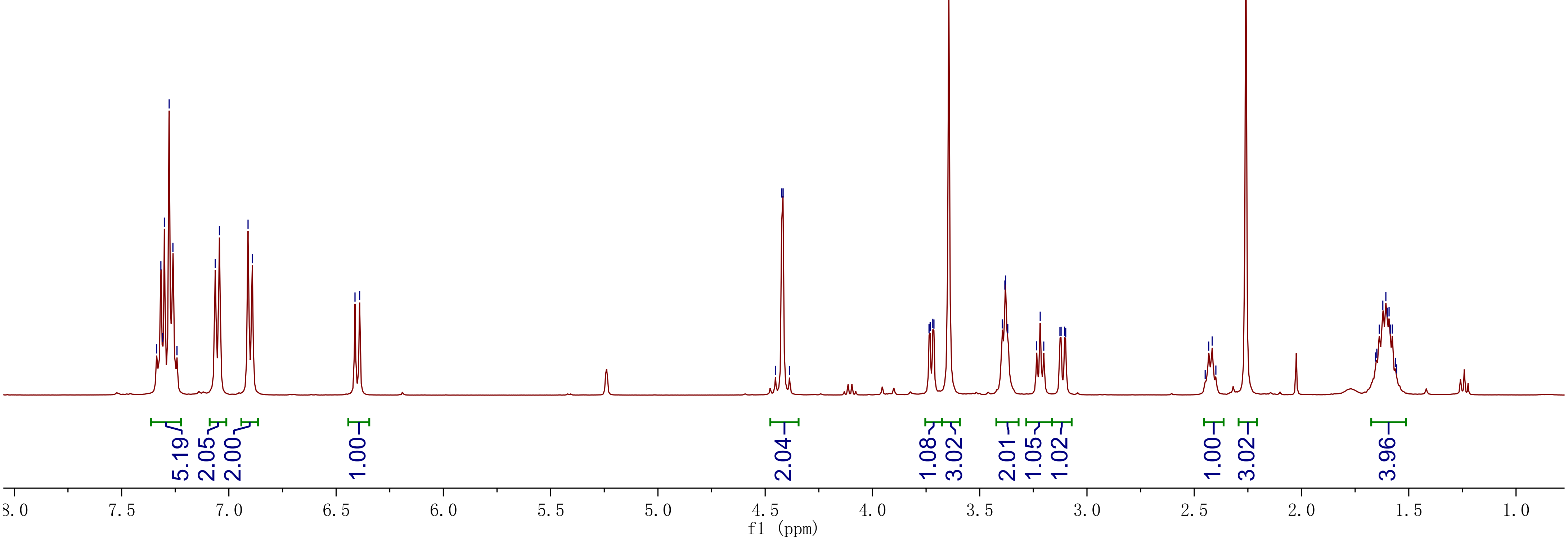

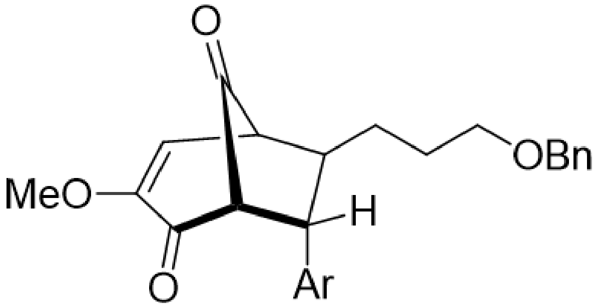

Ar = 4-$CH_3C_6H_4$

4v'

^{13}C NMR (101 MHz, $CDCl_3$)

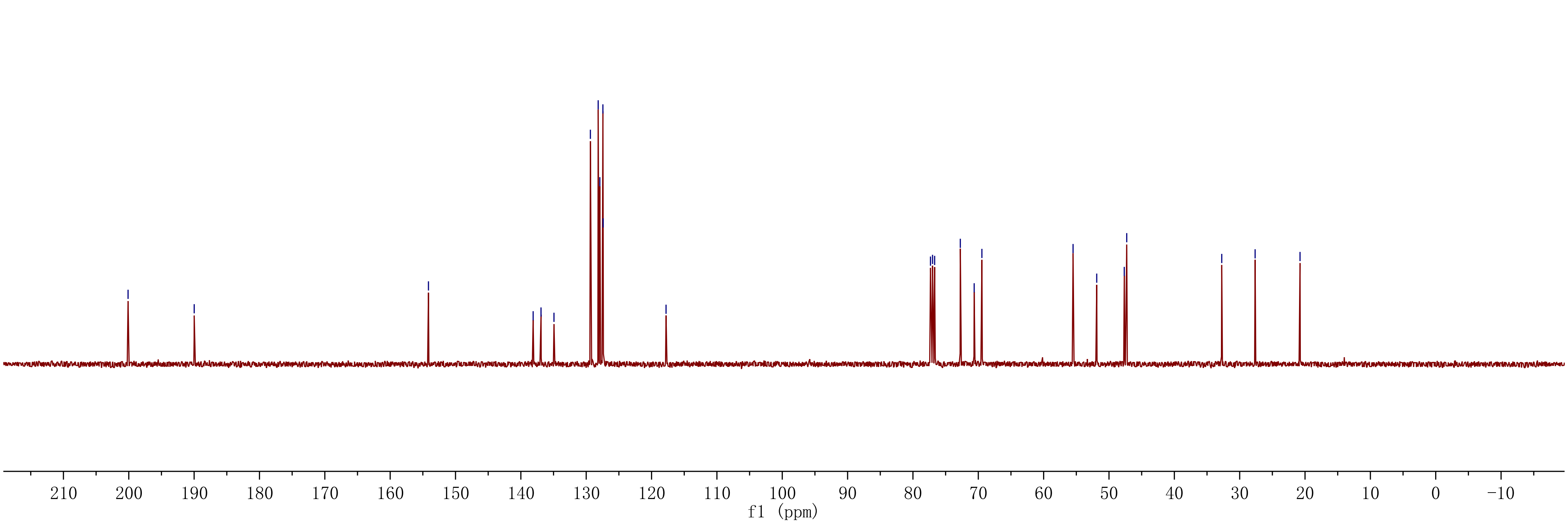

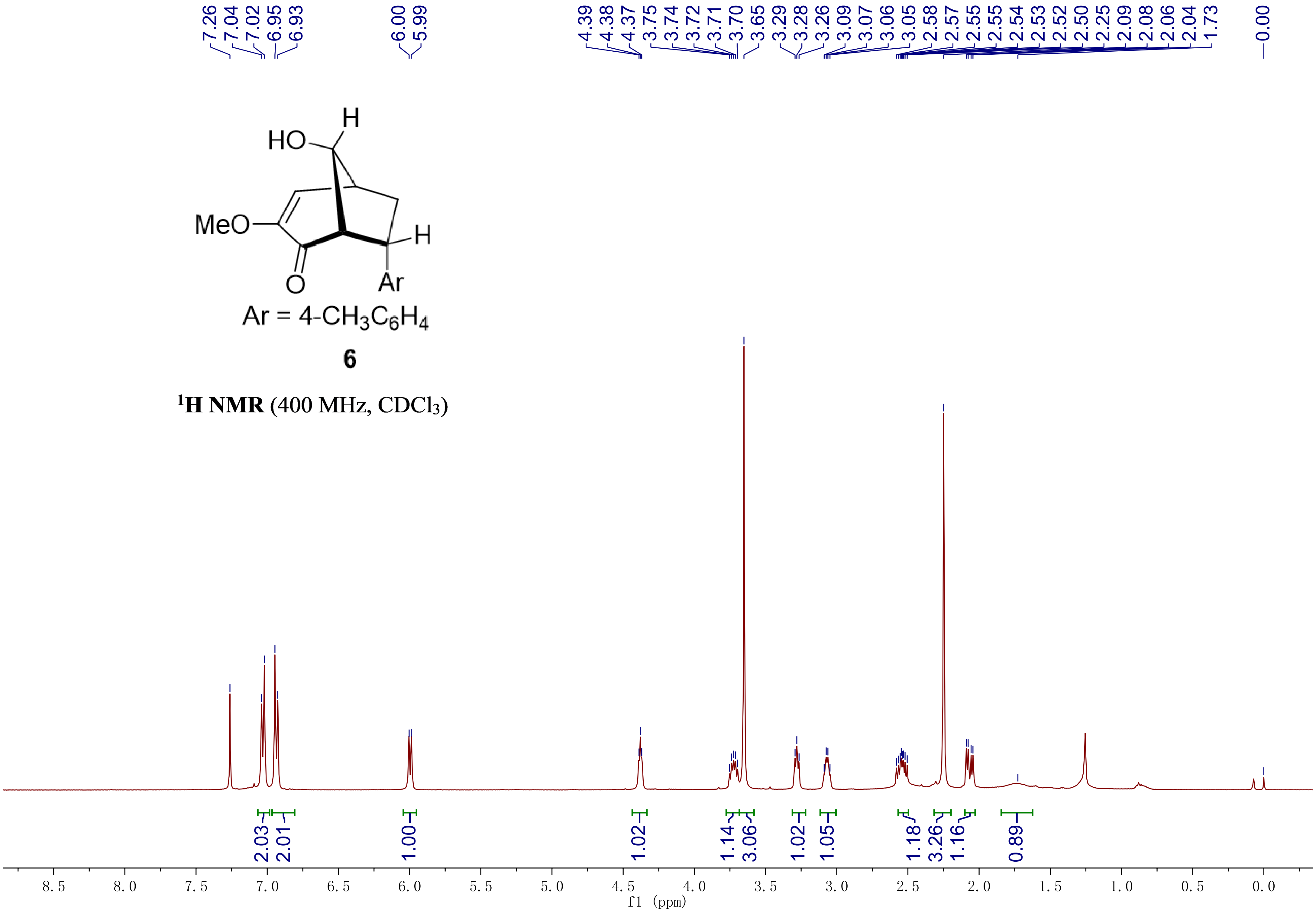
7.26
7.04
7.02
6.95
6.93
6.00
5.99
4.39
4.38
4.37
3.75
3.74
3.72
3.71
3.70
3.65
3.29
3.28
3.26
3.09
3.07
3.06
3.05
2.58
2.57
2.55
2.55
2.54
2.53
2.52
2.50
2.25
2.09
2.08
2.06
2.04
1.73
0.00
HO
H
MeO
H
O
Ar
Ar = 4-CH3C6H4
6
1H NMR (400 MHz, CDCl3)
2.03
2.01
1.00
1.02
1.14
3.06
1.02
1.05
1.18
3.26
1.16
0.89
8.5
8.0
7.5
7.0
6.5
6.0
5.5
5.0
4.5
4.0
3.5
3.0
2.5
2.0
1.5
1.0
0.5
0.0
f1 (ppm)

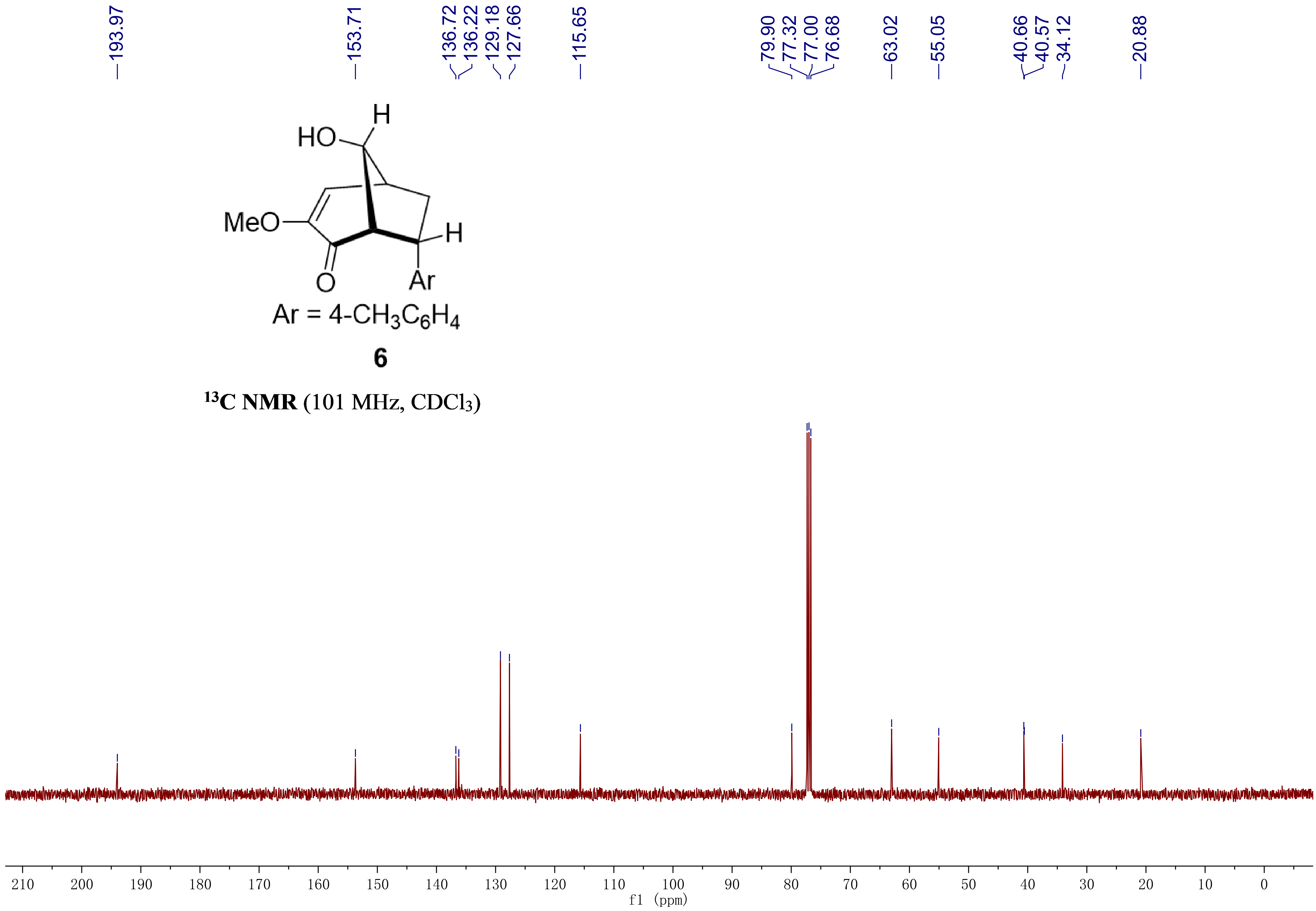

Ar = 4-$CH_3C_6H_4$

6

^{13}C NMR (101 MHz, $CDCl_3$)

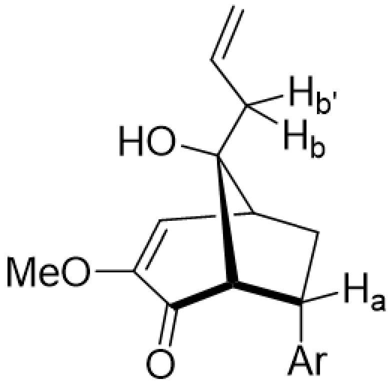

7

^{1}H NMR (400 MHz, $CDCl_3$)

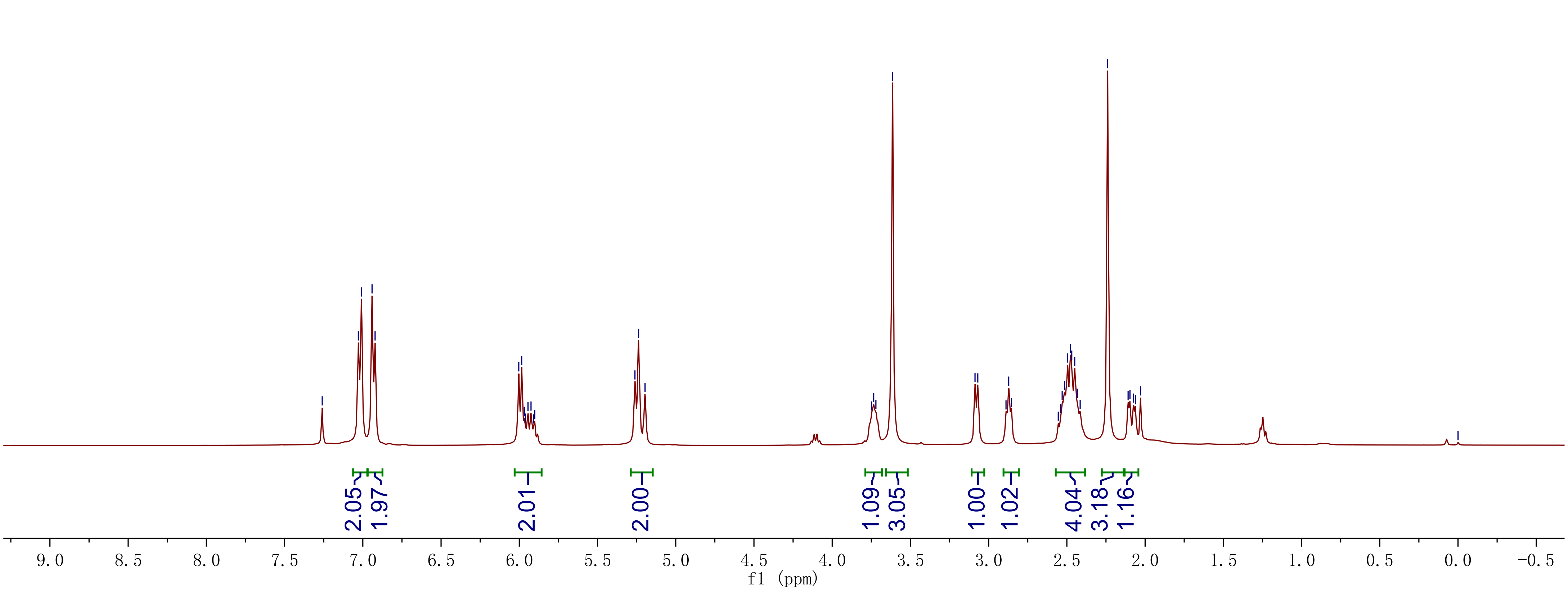

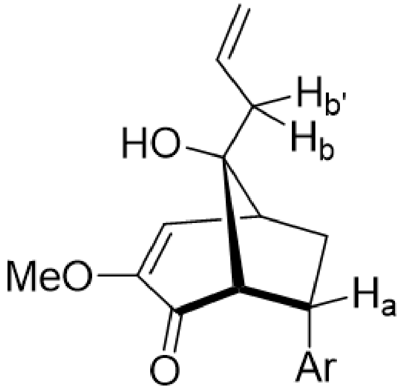

7

^{13}C NMR (101 MHz, $CDCl_3$)

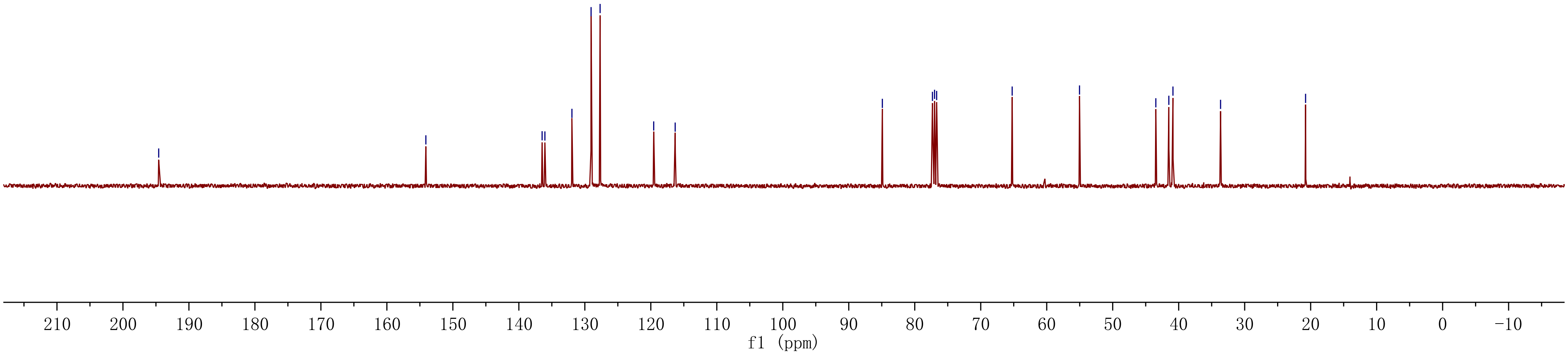

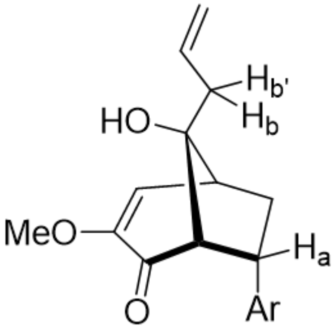

7

(DEPT 135 NMR, $CDCl_3$)

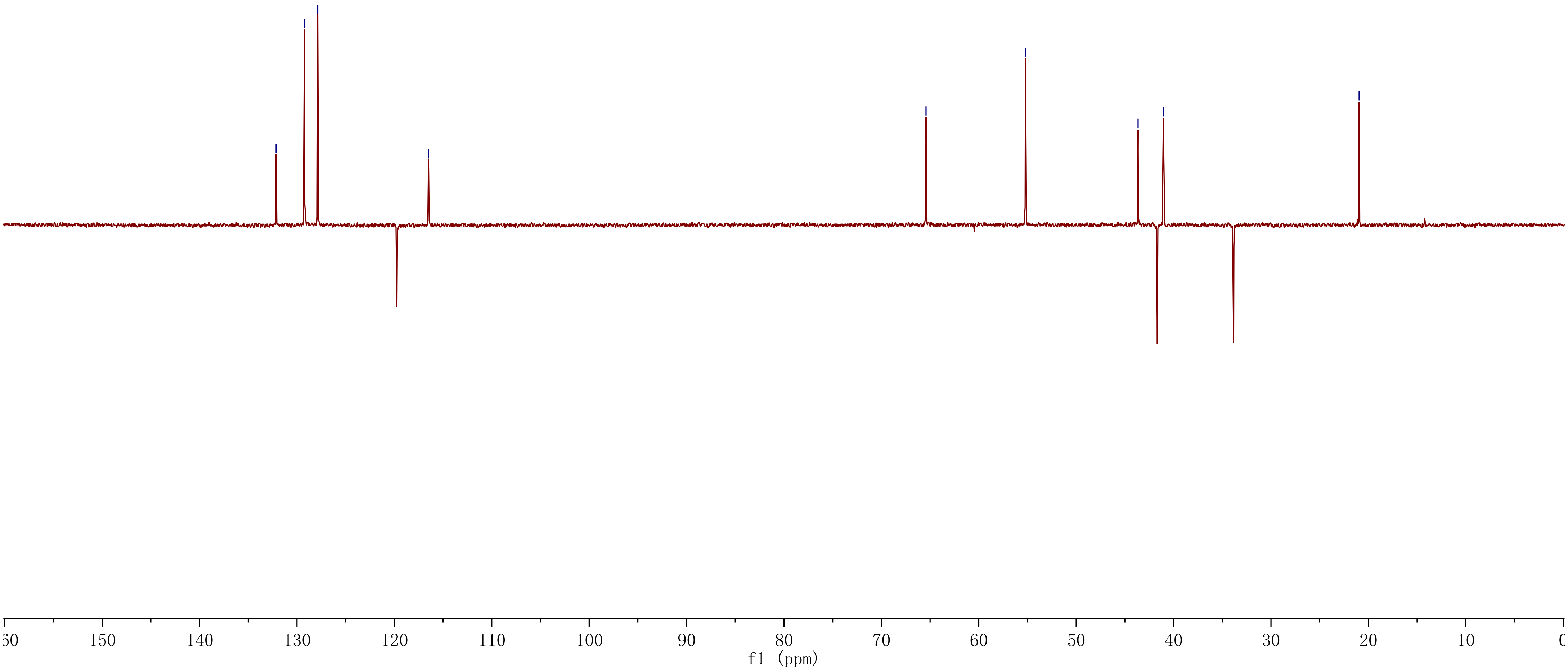

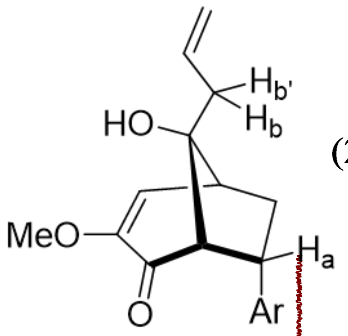

7

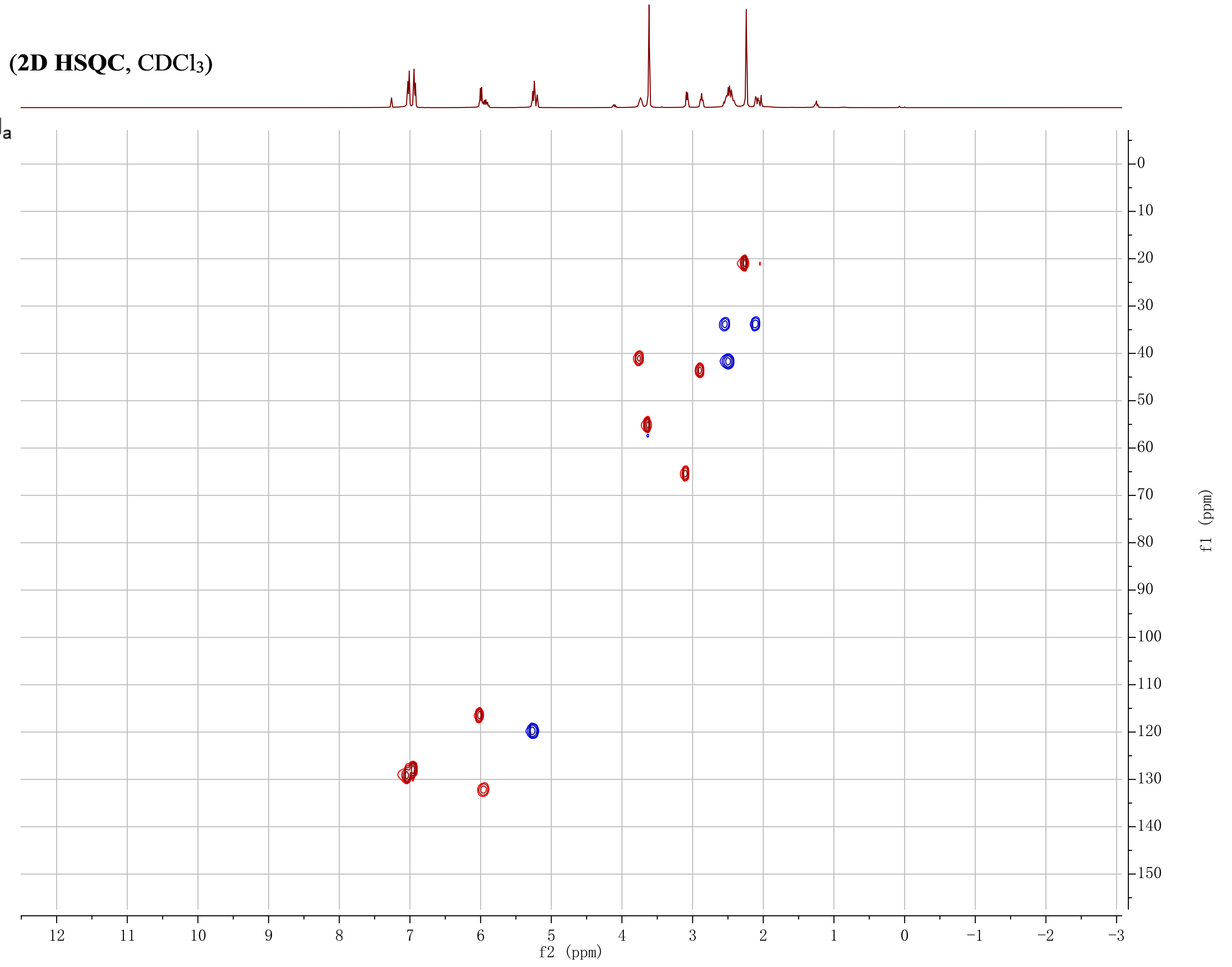

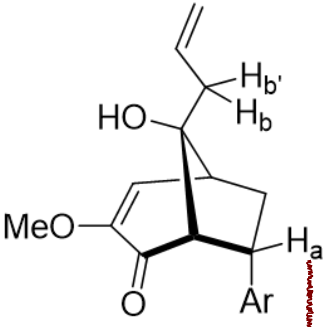

7

(2D HMBC, $CDCl_3$)

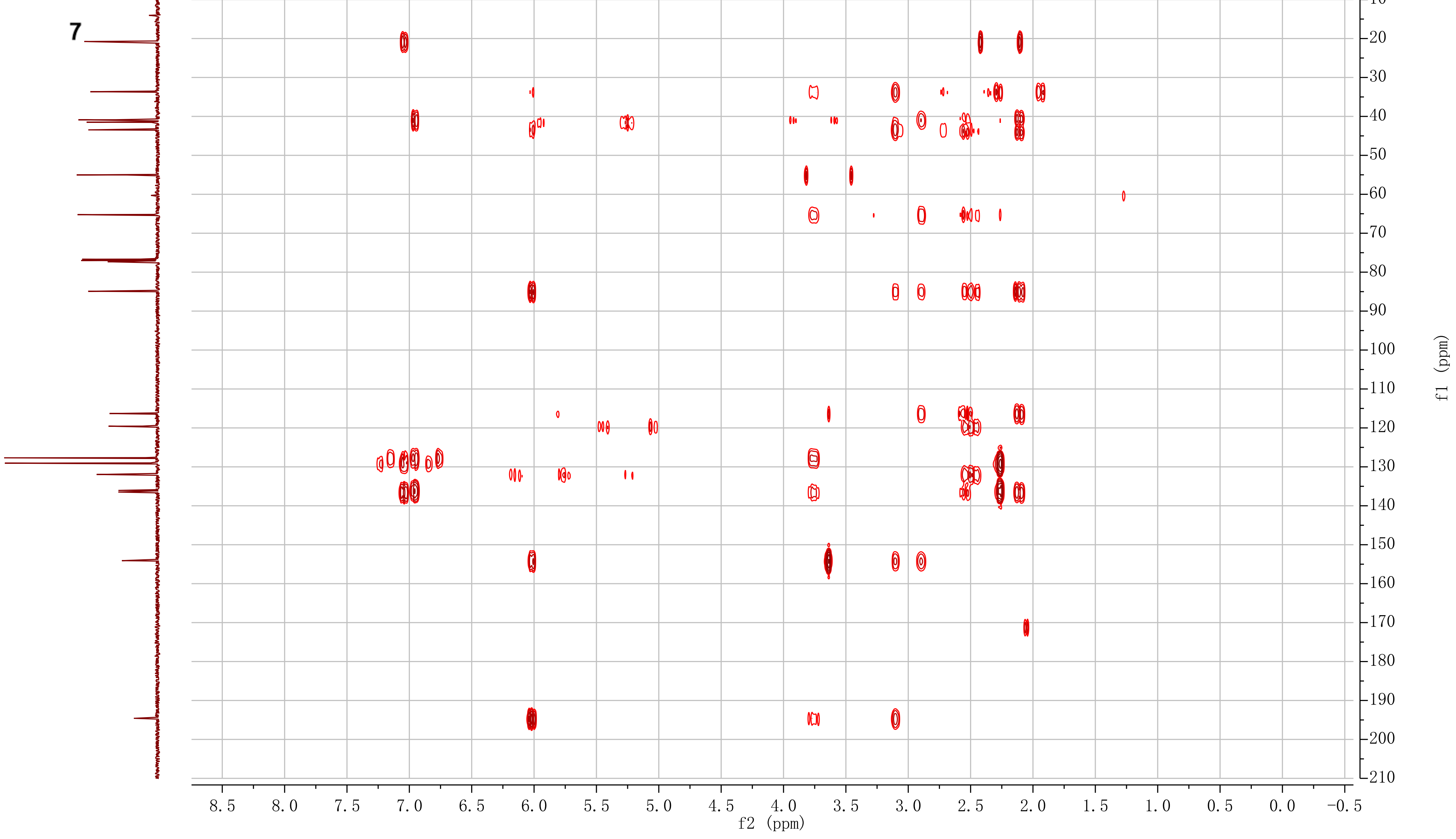

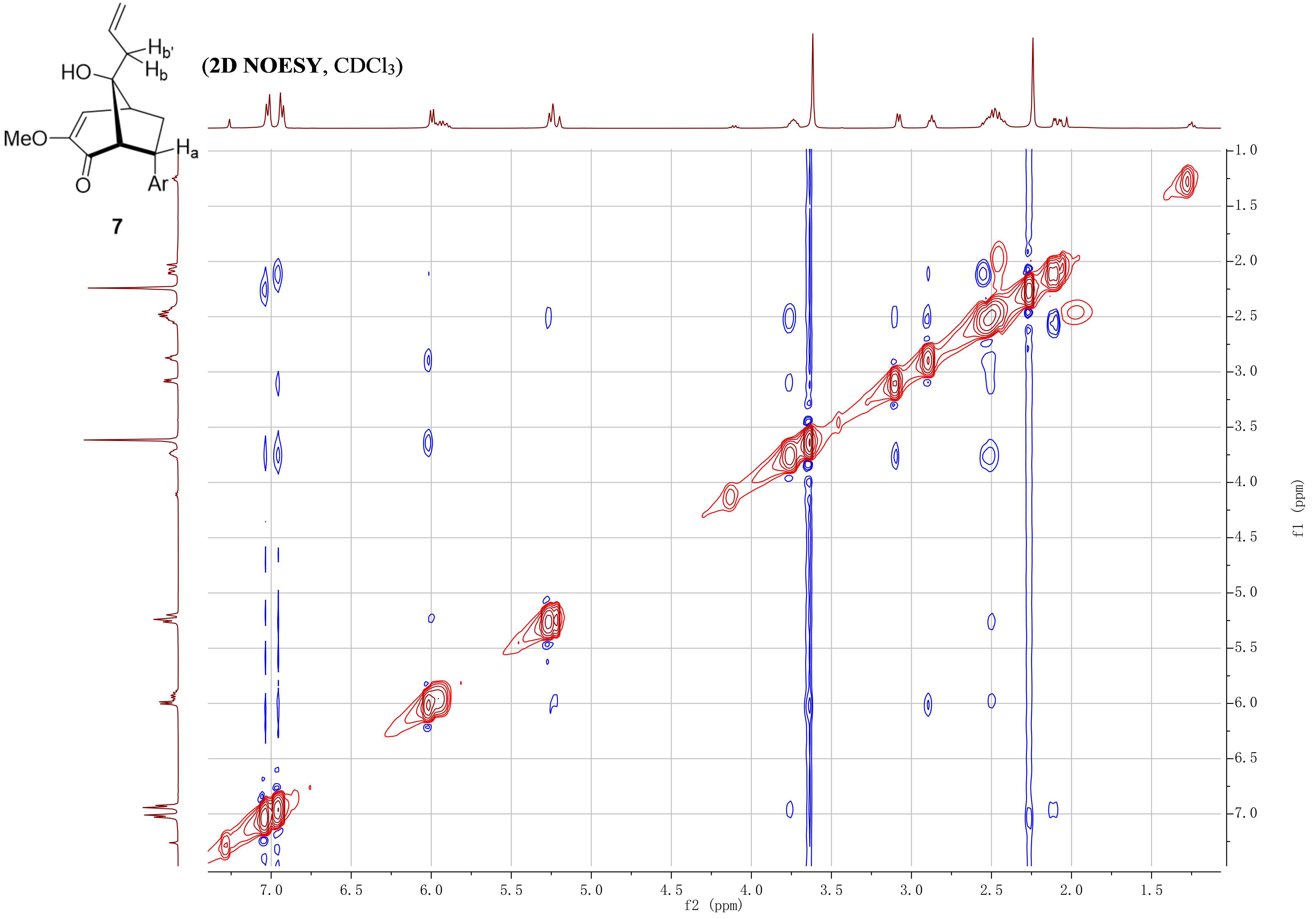

(2D NOESY, CDCl3)
HO
MeO
O
Hb'
Hb
Ha
Ar
7
f2 (ppm)
f1 (ppm)

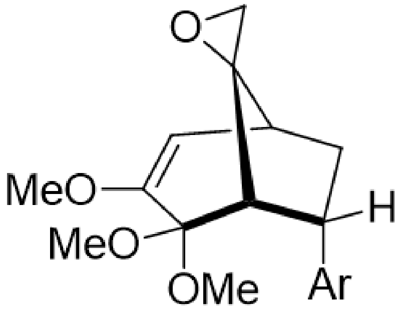

Ar = 4-$CH_3C_6H_4$

8

^{1}H NMR (400 MHz, $CDCl_3$)

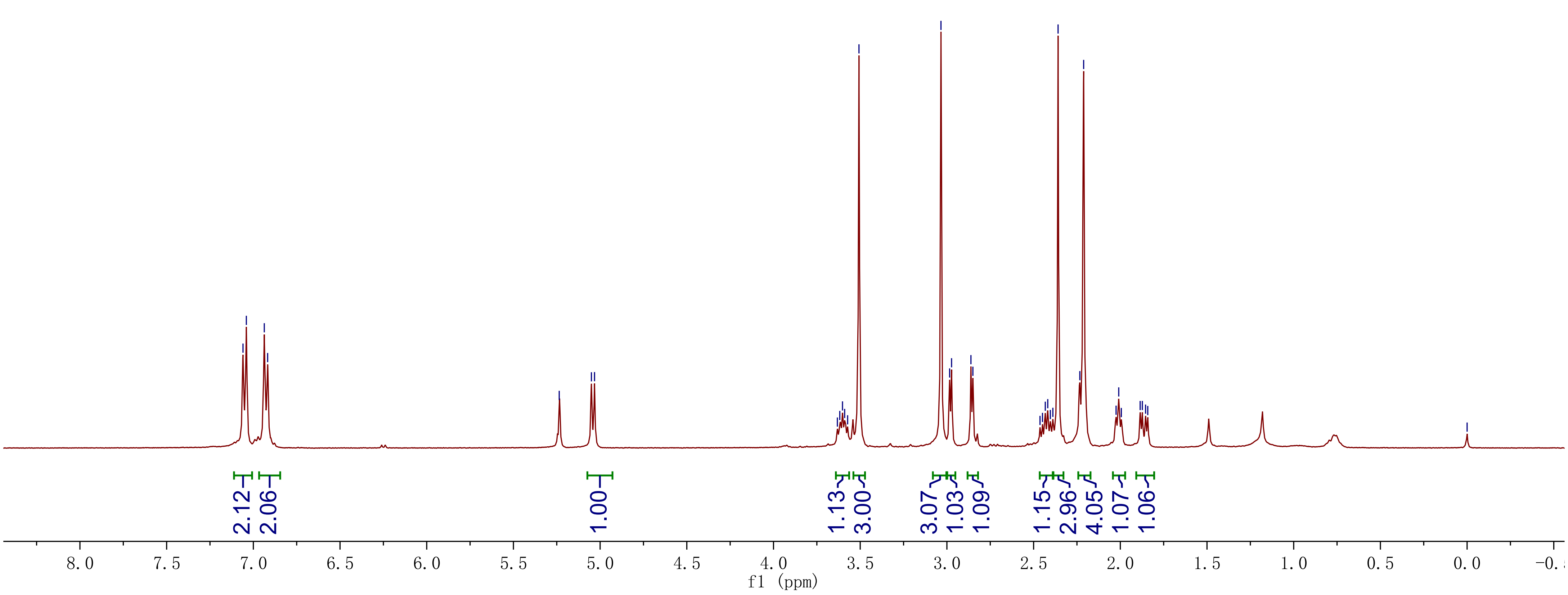

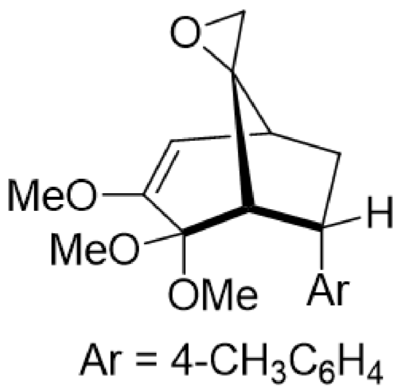

8

^{13}C NMR (101 MHz, $CDCl_3$)

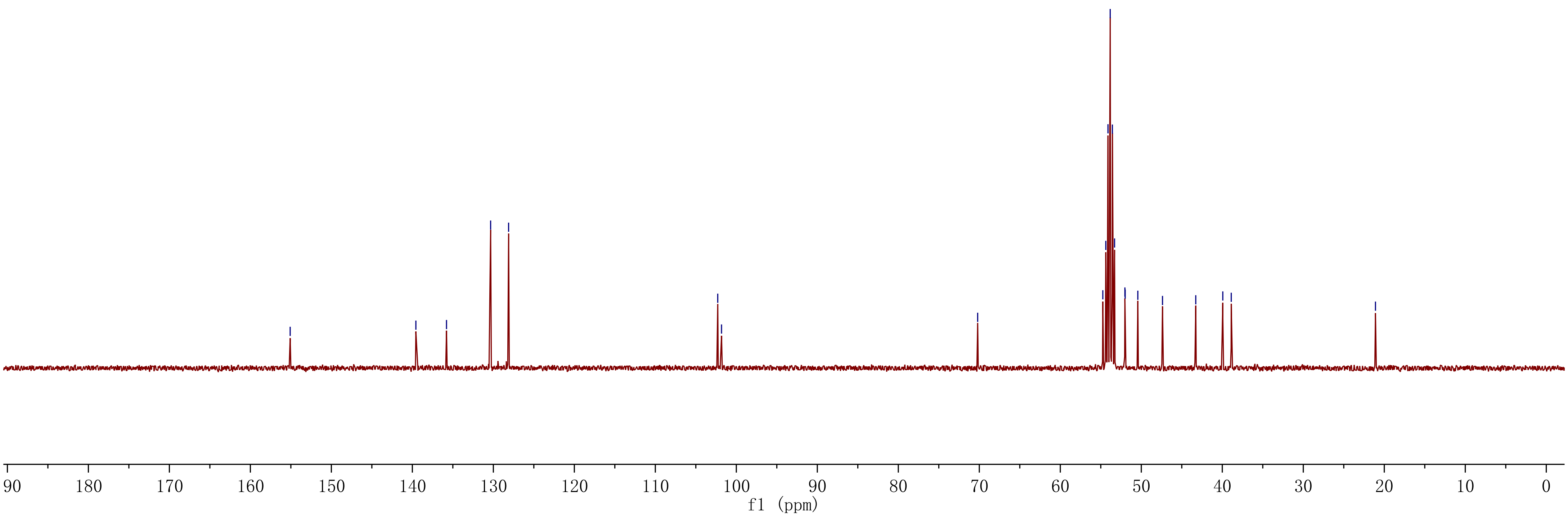

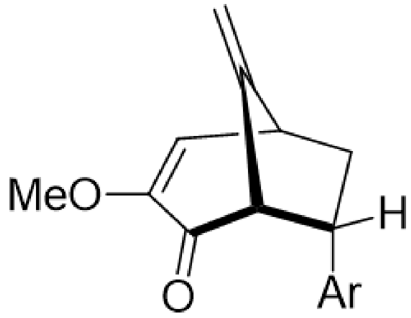

Ar = 4-$CH_3C_6H_4$

9

^{1}H NMR (400 MHz, $CDCl_3$)

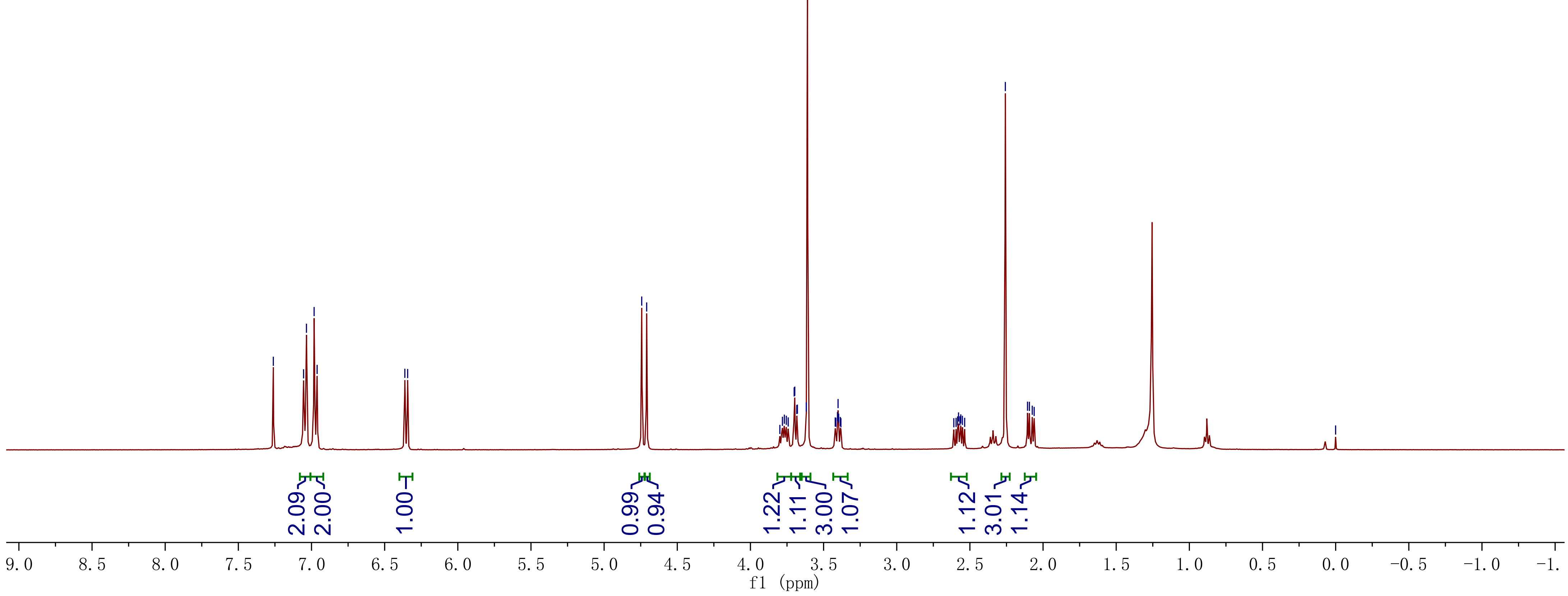

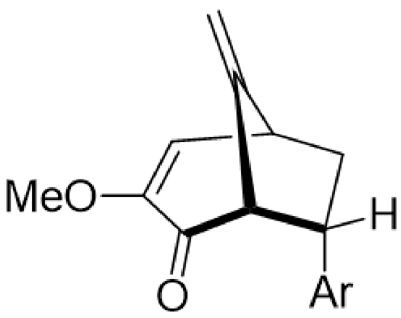

Ar = 4-$CH_3C_6H_4$

9

^{13}C NMR (101 MHz, $CDCl_3$)

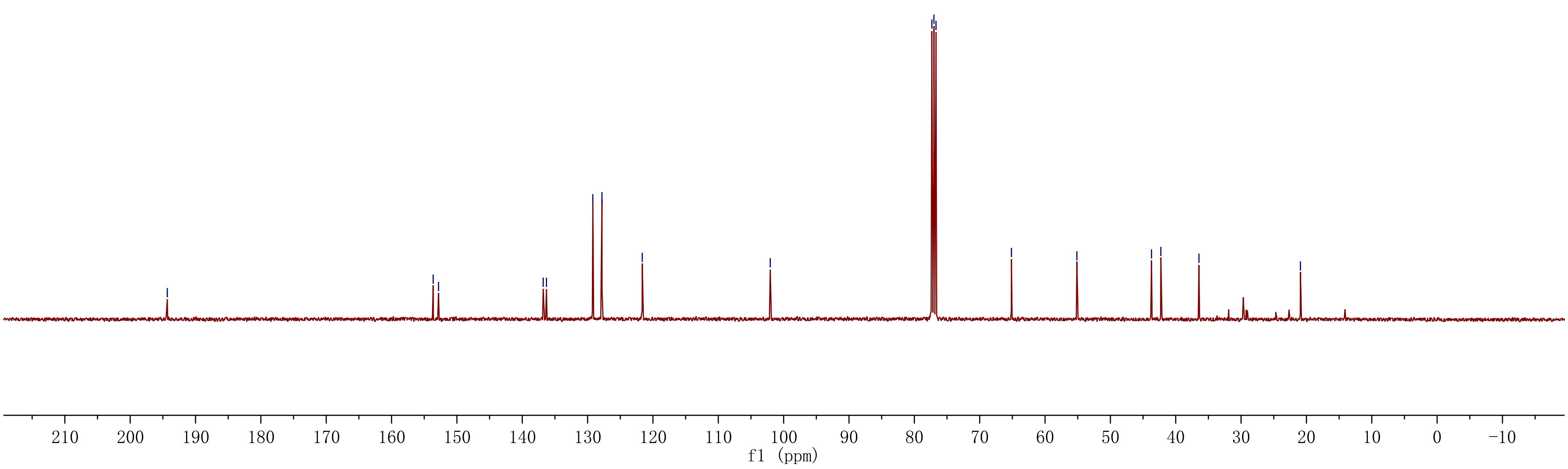

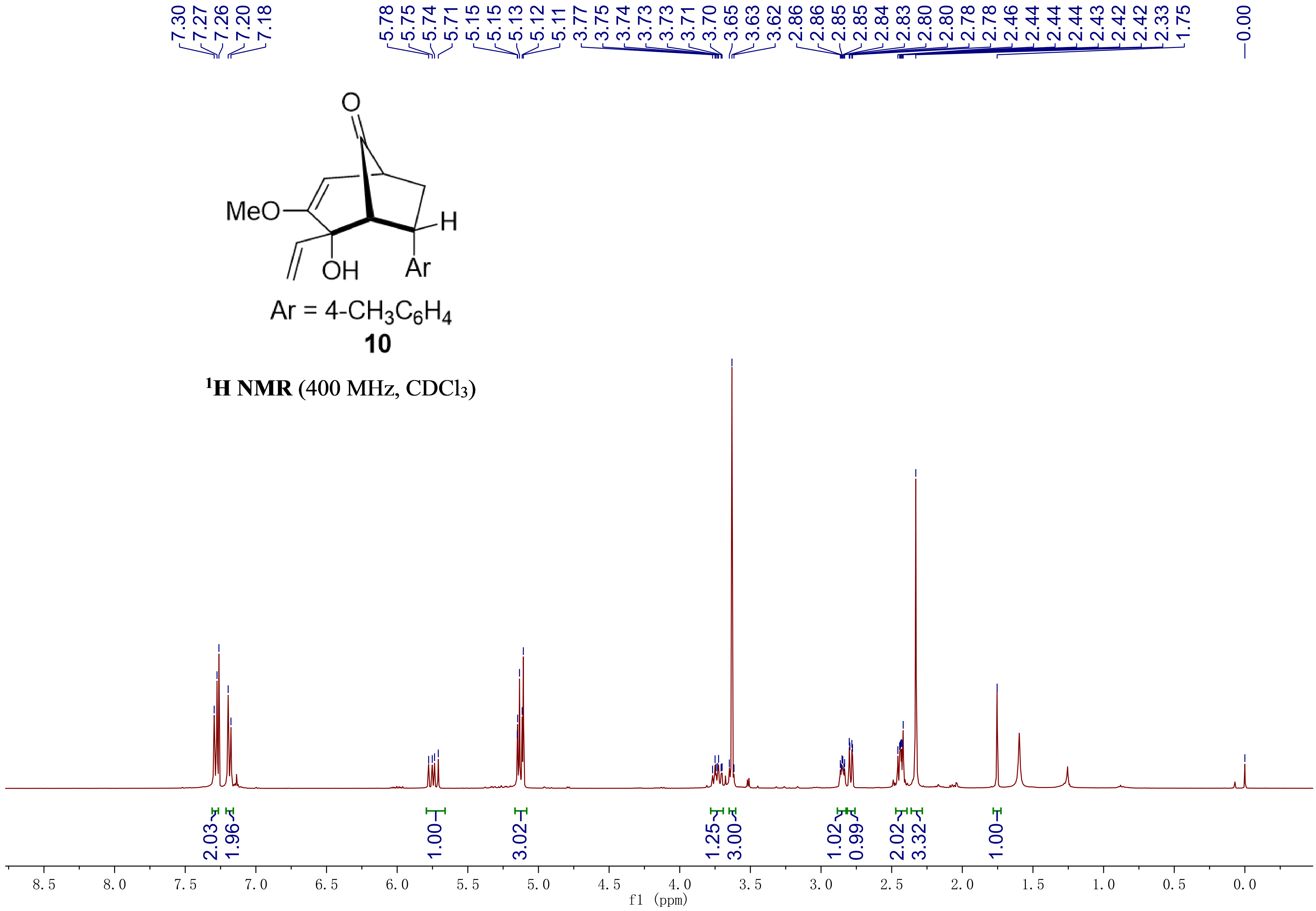

^{1}H NMR (400 MHz, $CDCl_3$)

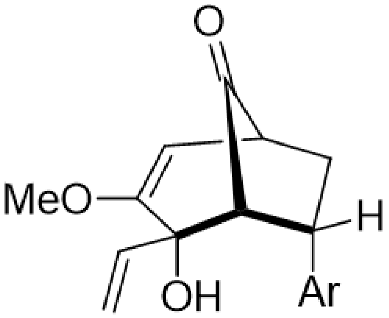

Ar = 4-$CH_3C_6H_4$

10

^{13}C NMR (101 MHz, $CDCl_3$)

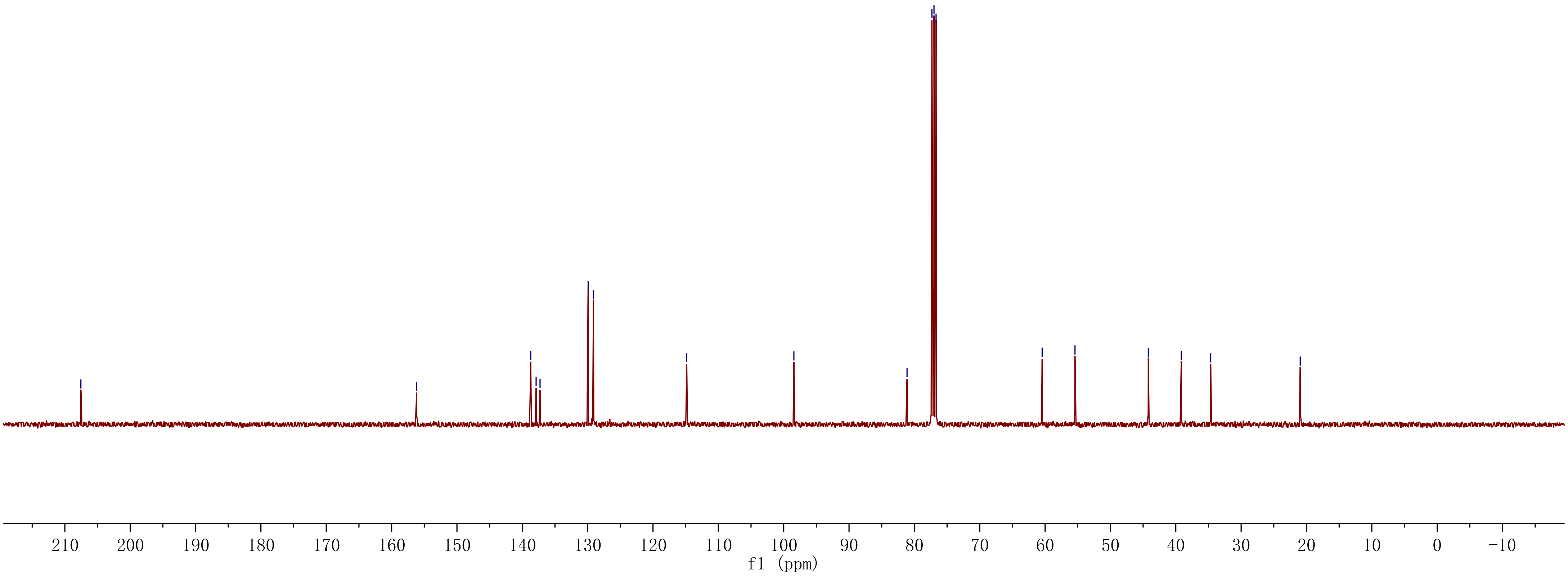

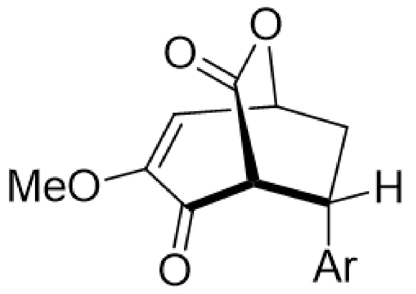

Ar = 4-$CH_3C_6H_4$

11

^{1}H NMR (400 MHz, $CDCl_3$)

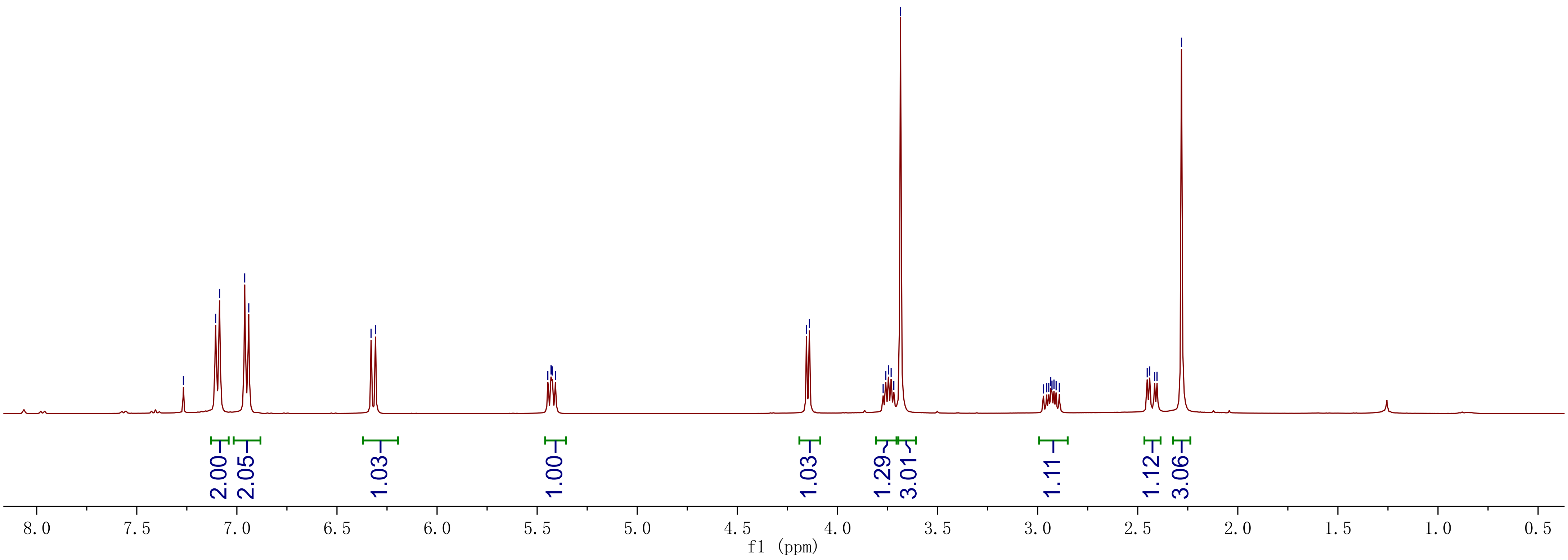

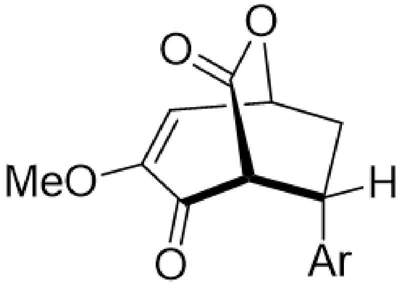

Ar = 4-$CH_3C_6H_4$

11

^{13}C NMR (101 MHz, $CDCl_3$)

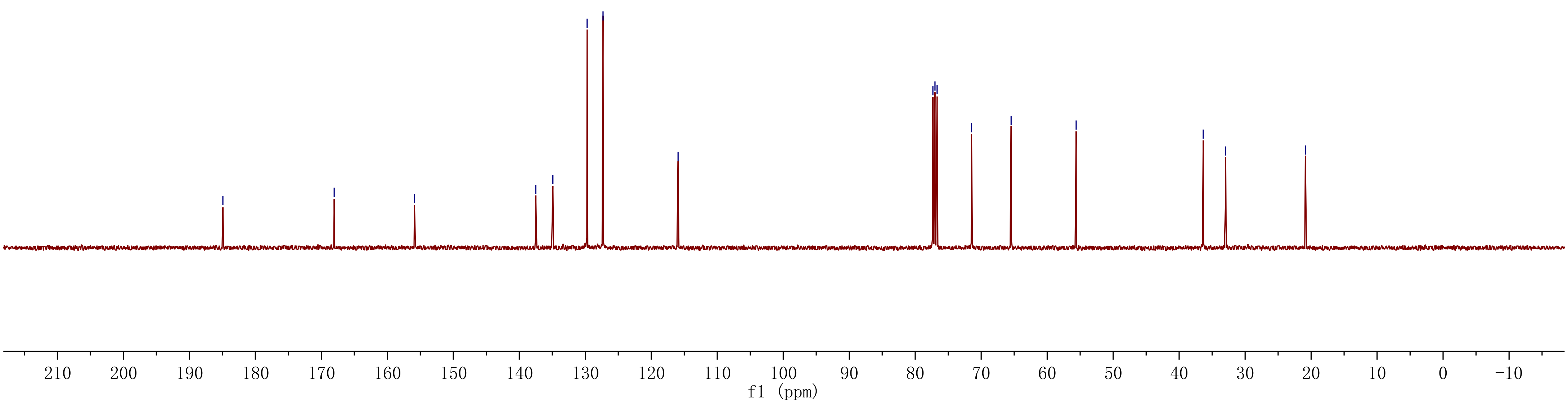

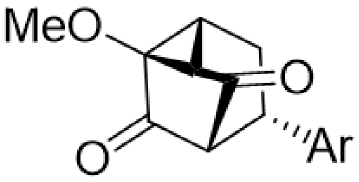

Ar = 4-$CH_3C_6H_4$

12

^{1}H NMR (400 MHz, $CDCl_3$)

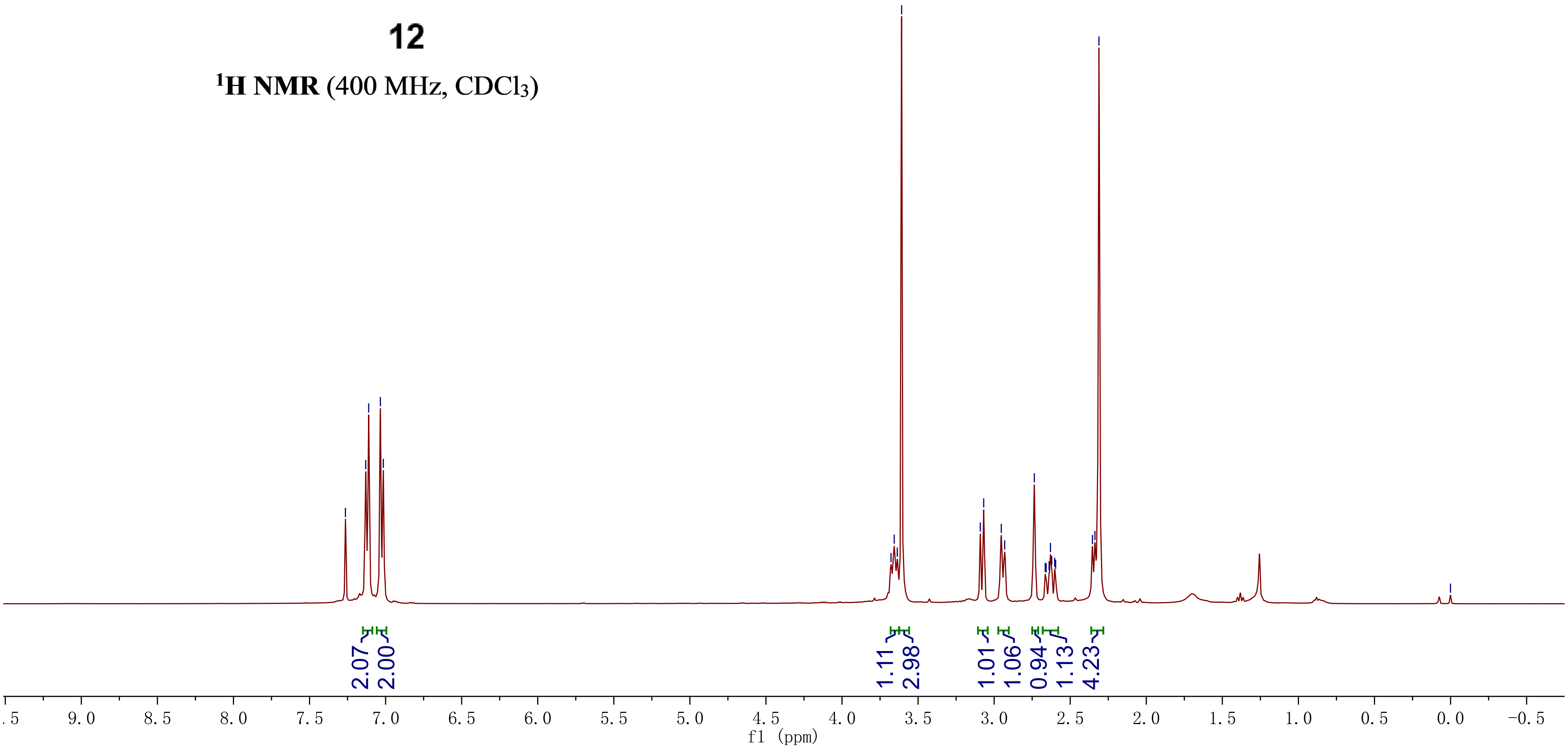

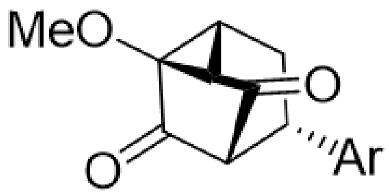

Ar = 4-$CH_3C_6H_4$

12

^{13}C NMR (101 MHz, $CDCl_3$)

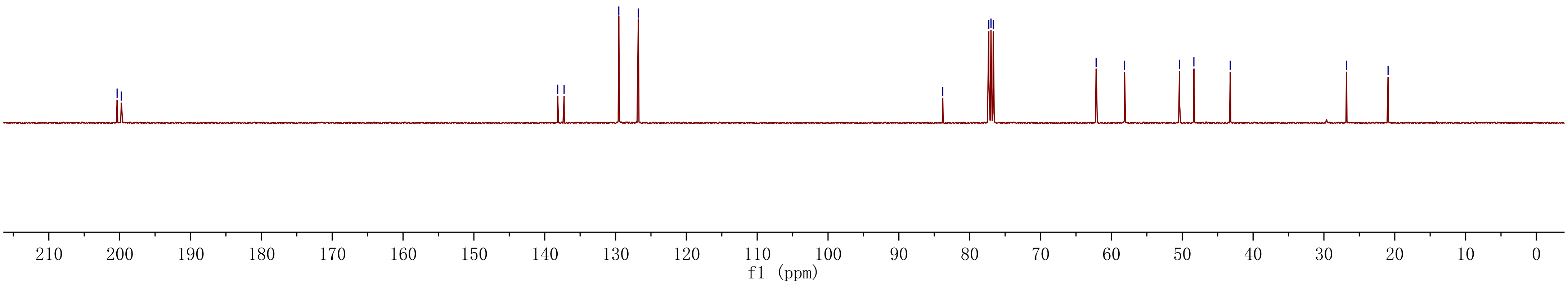

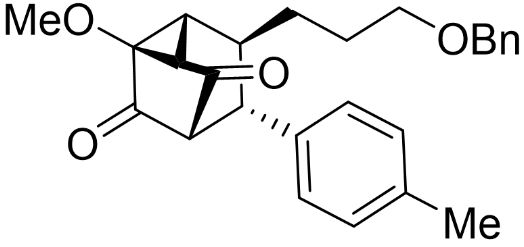

13

^{1}H NMR (400 MHz, $CDCl_3$)

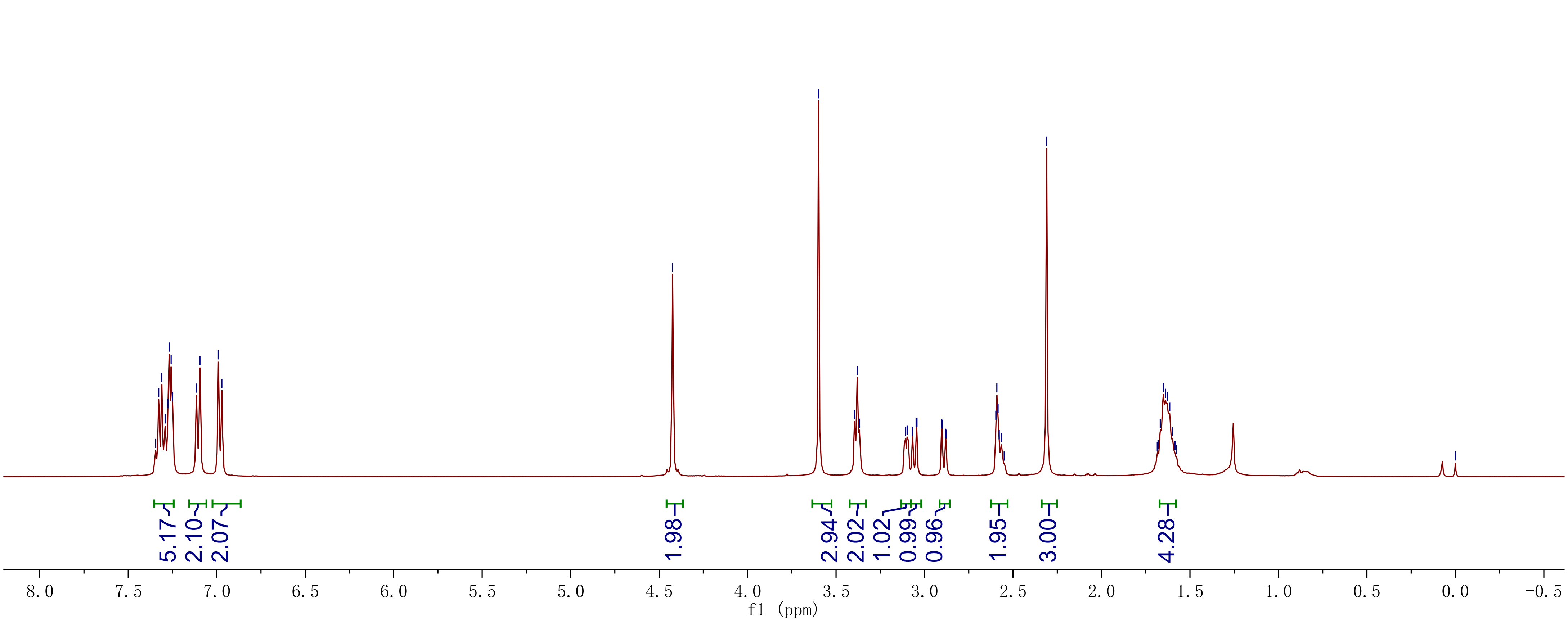

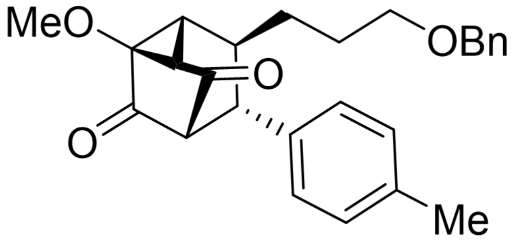

13

^{13}C NMR (101 MHz, $CDCl_3$)

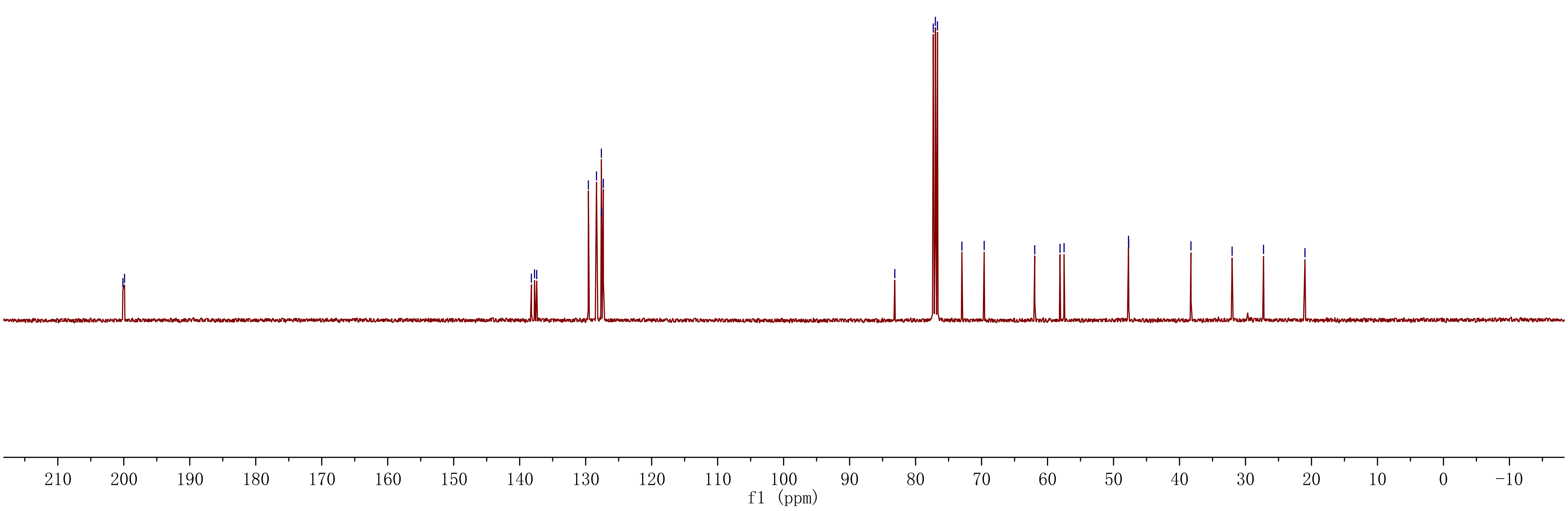

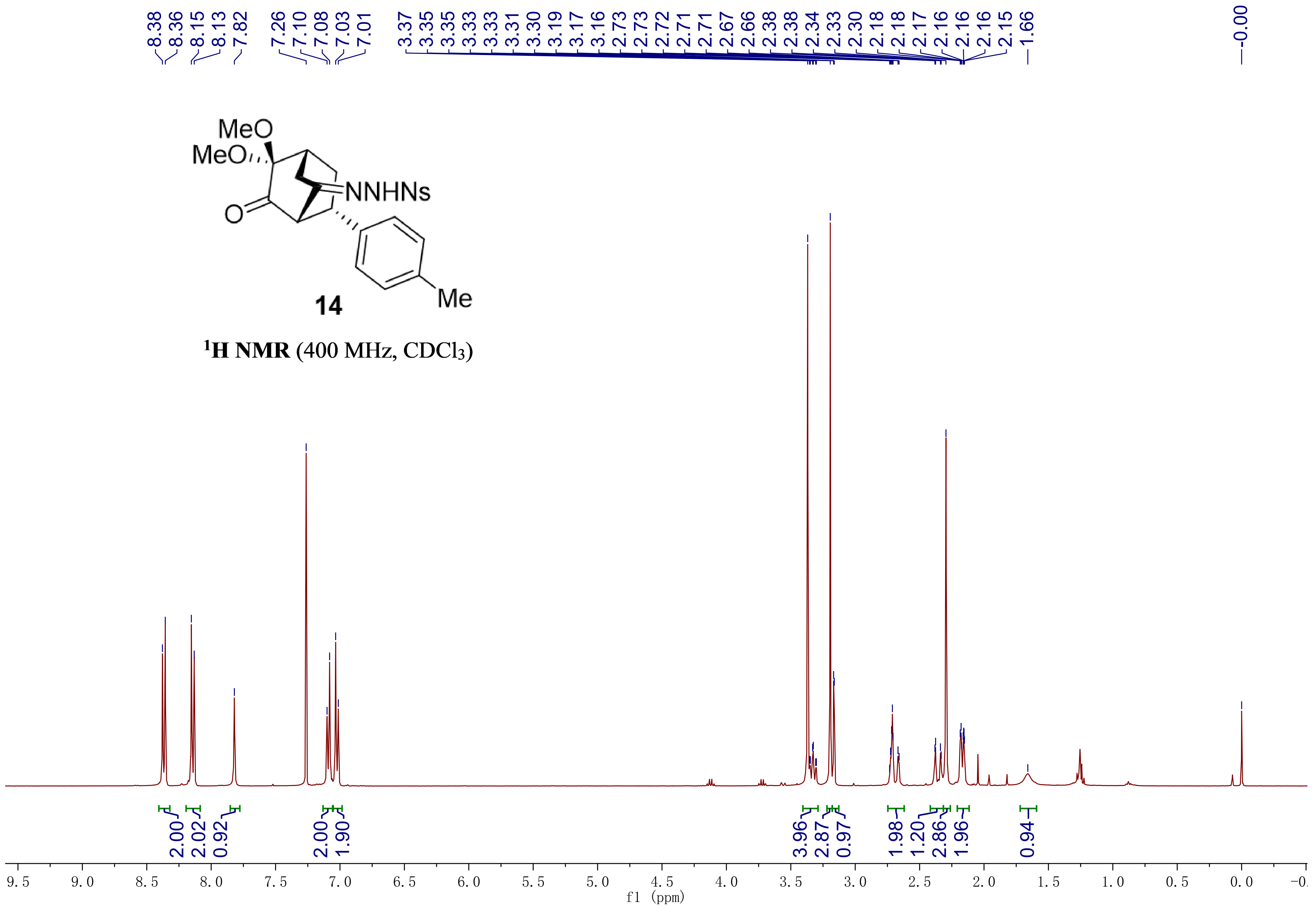
8.38 8.36 8.15 8.13 7.82
7.26 7.10 7.08 7.03 7.01
3.37 3.35 3.35 3.33 3.33 3.31 3.30 3.19 3.17 3.16 2.73 2.73 2.72 2.71 2.71 2.67 2.66 2.38 2.38 2.34 2.33 2.30 2.18 2.18 2.17 2.16 2.16 2.16 2.15 1.66
-0.00
MeO
MeO
NNHNs
O
Me
14
1H NMR (400 MHz, CDCl3)
2.00 2.02 0.92 2.00 1.90 3.96 2.87 0.97 1.98 1.20 2.86 1.96 0.94
9.5 9.0 8.5 8.0 7.5 7.0 6.5 6.0 5.5 5.0 4.5 4.0 3.5 3.0 2.5 2.0 1.5 1.0 0.5 0.0
f1 (ppm)

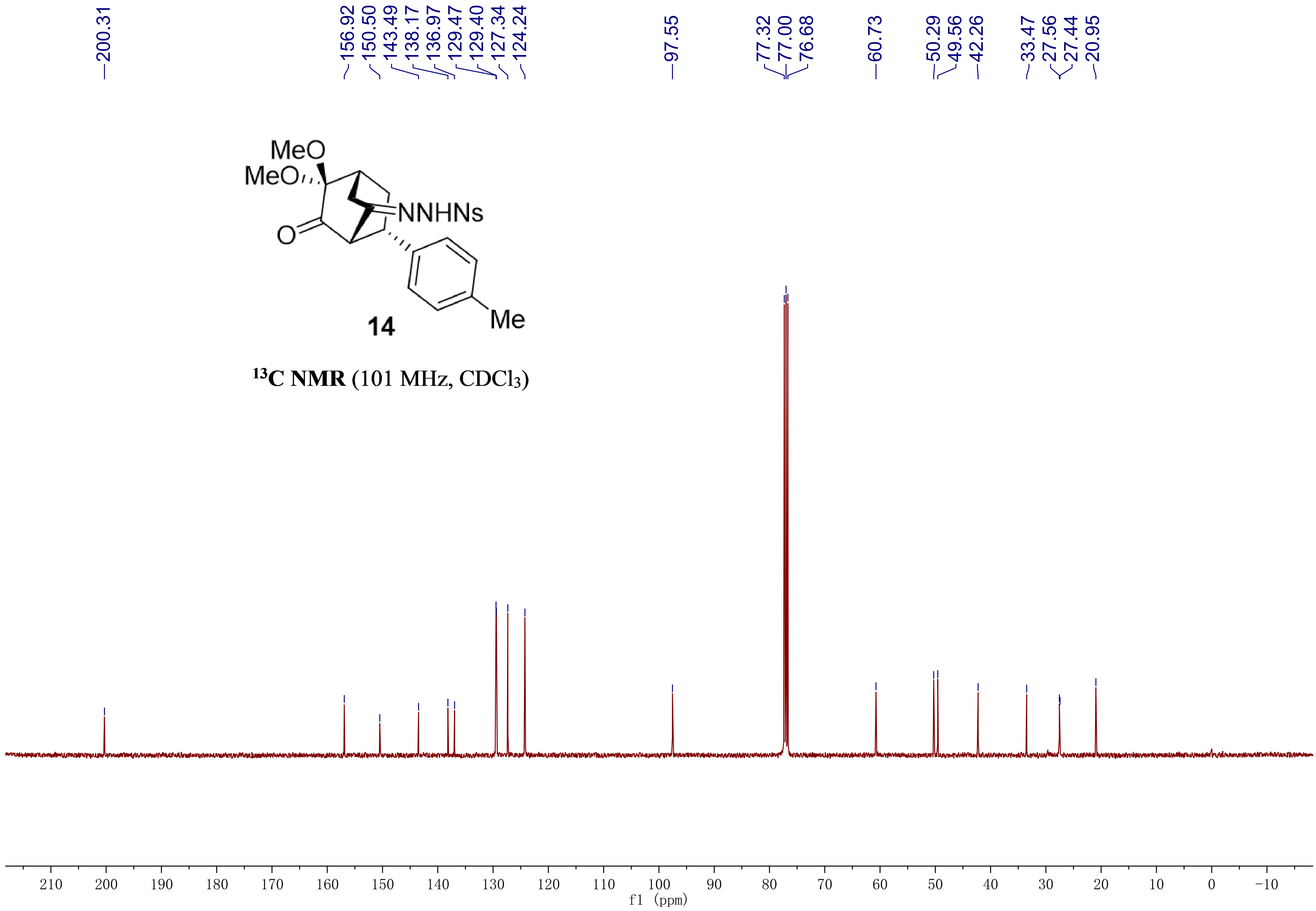

200.31
156.92
150.50
143.49
138.17
136.97
129.47
129.40
127.34
124.24
97.55
77.32
77.00
76.68
60.73
50.29
49.56
42.26
33.47
27.56
27.44
20.95
MeO
MeO
NNHNs
O
Me
14
^{13}C NMR (101 MHz, $CDCl_3$)
210 200 190 180 170 160 150 140 130 120 110 100 90 80 70 60 50 40 30 20 10 0 −10
f1 (ppm)

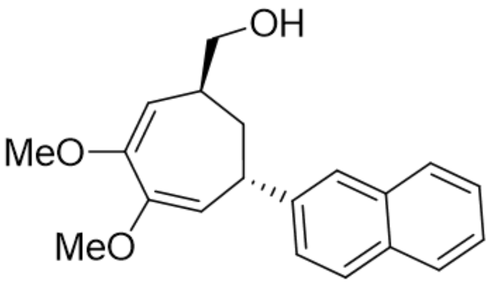

15

^{1}H NMR (400 MHz, $CDCl_3$)

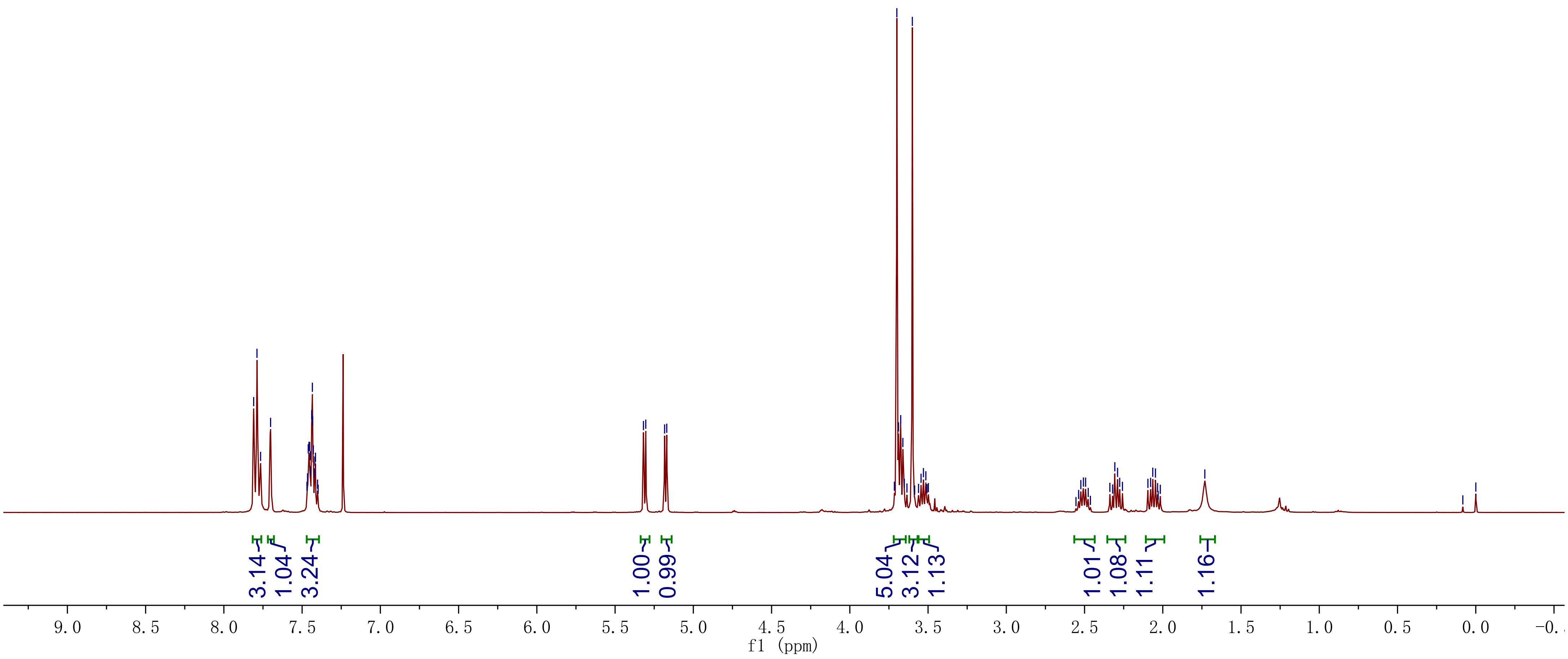

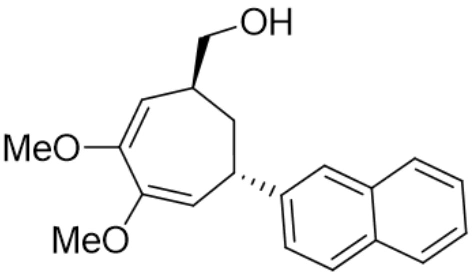

15

^{13}C NMR (101 MHz, $CDCl_3$)

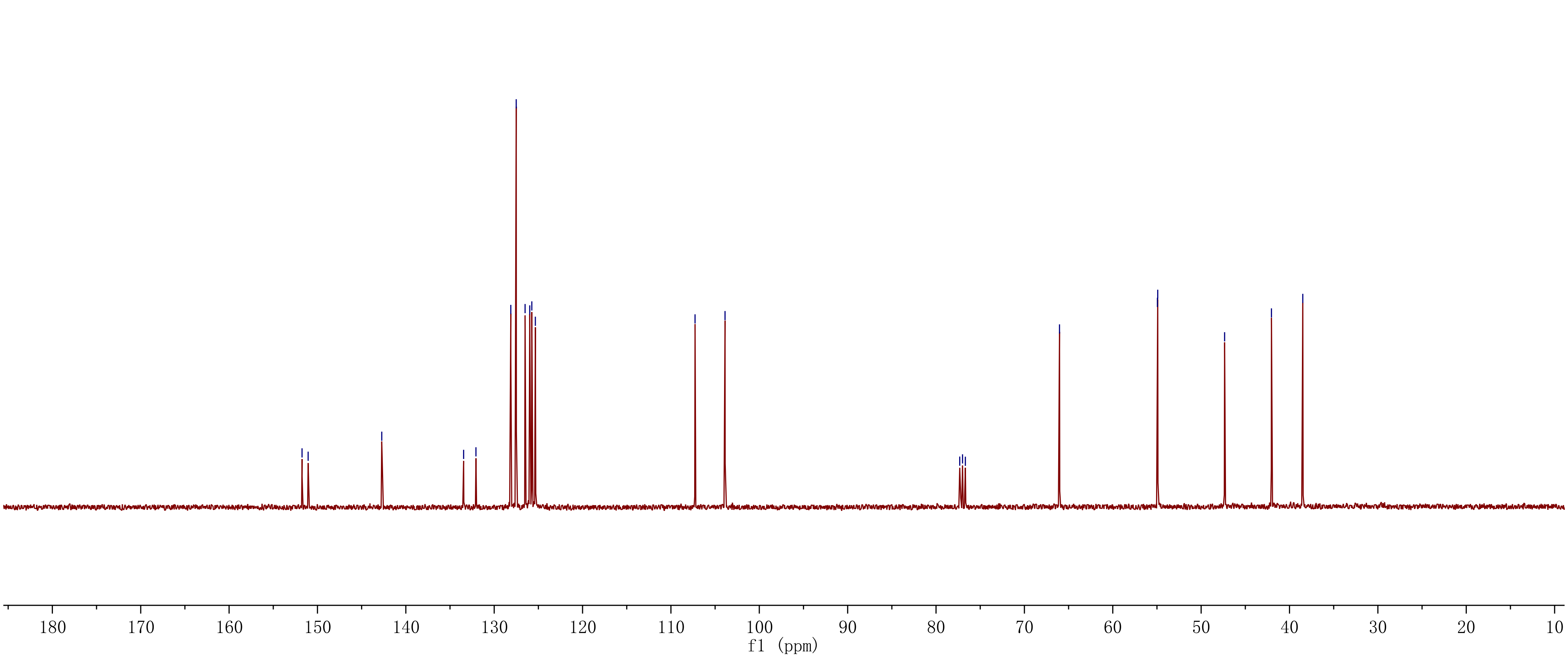

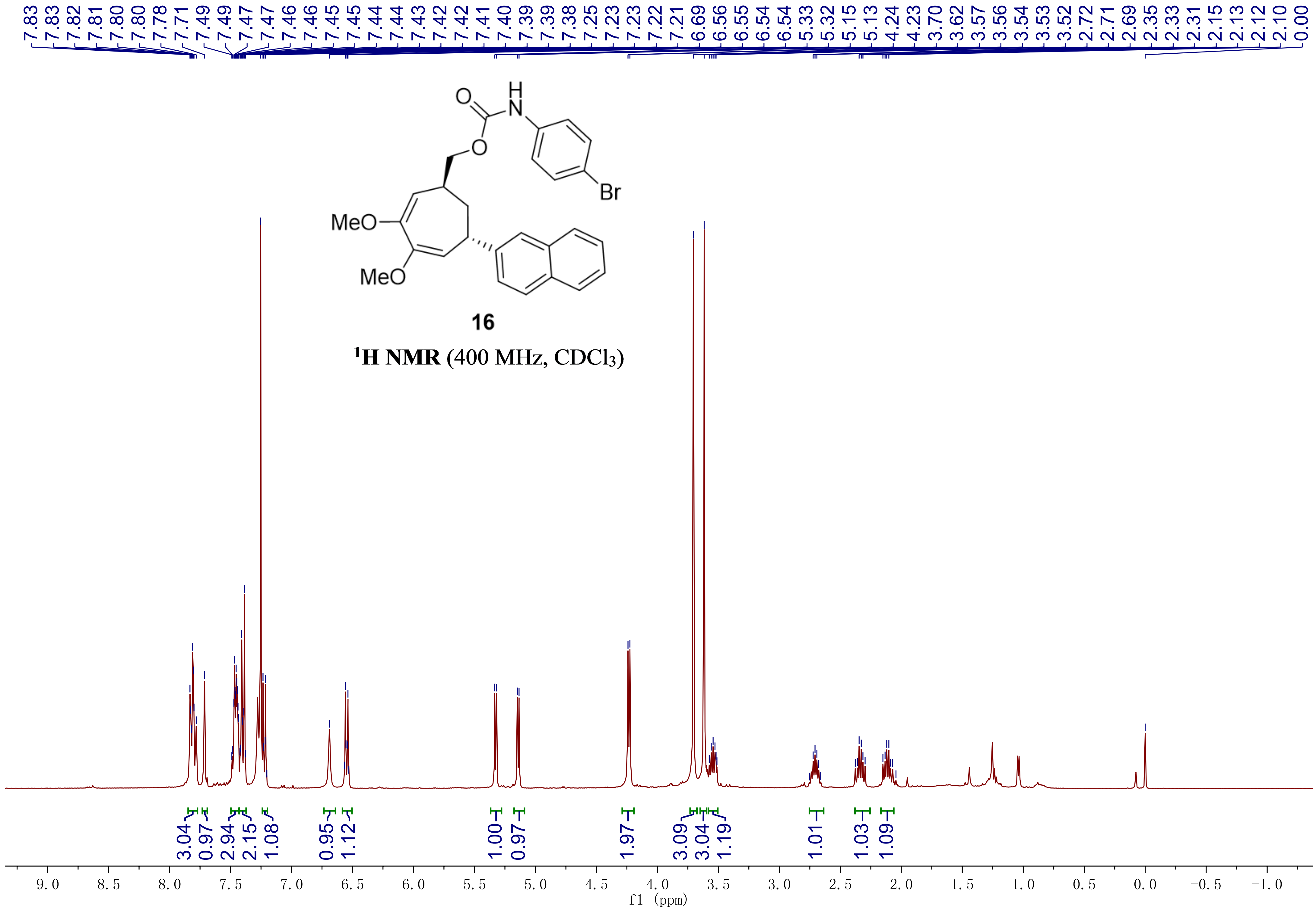

16

^{1}H NMR (400 MHz, $CDCl_3$)

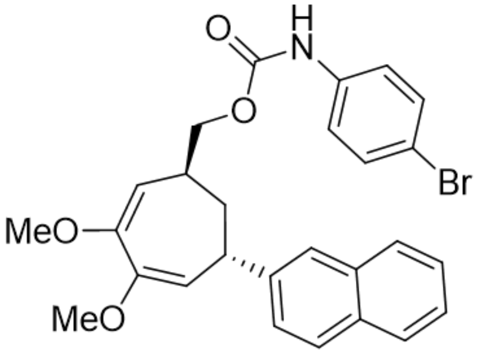

16

^{13}C NMR (101 MHz, $CDCl_3$)

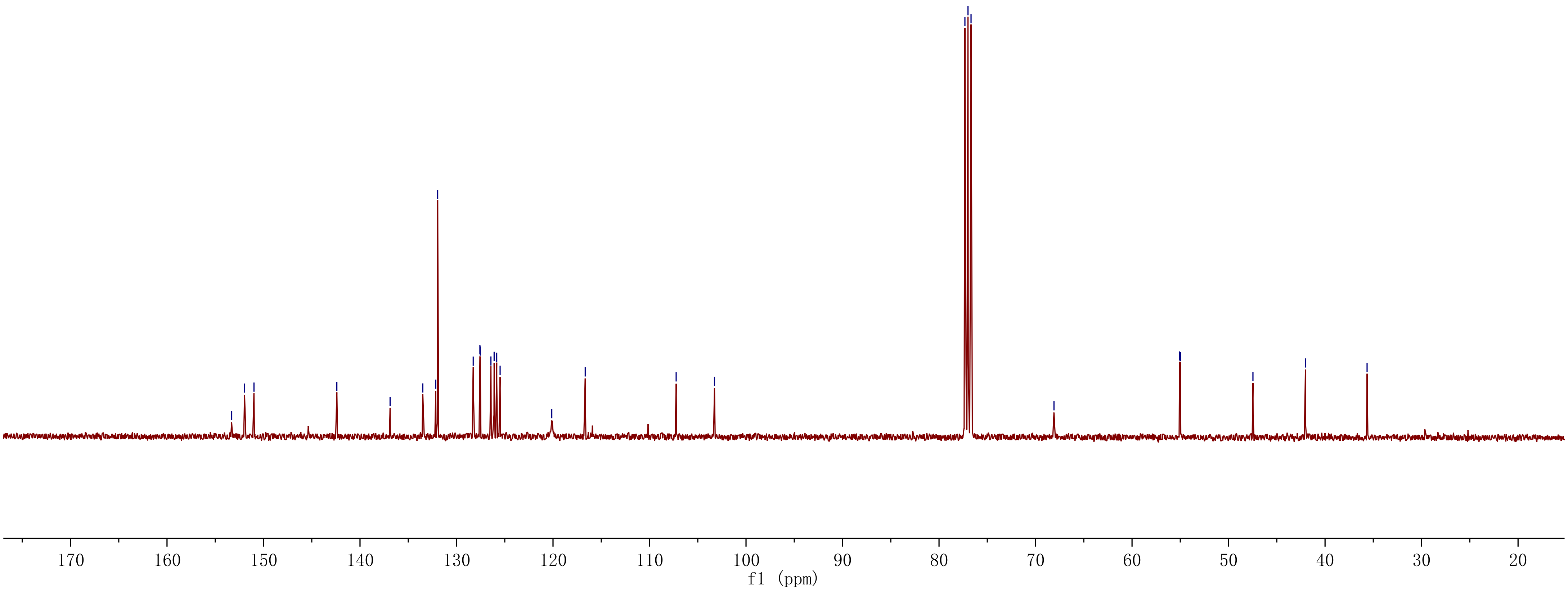

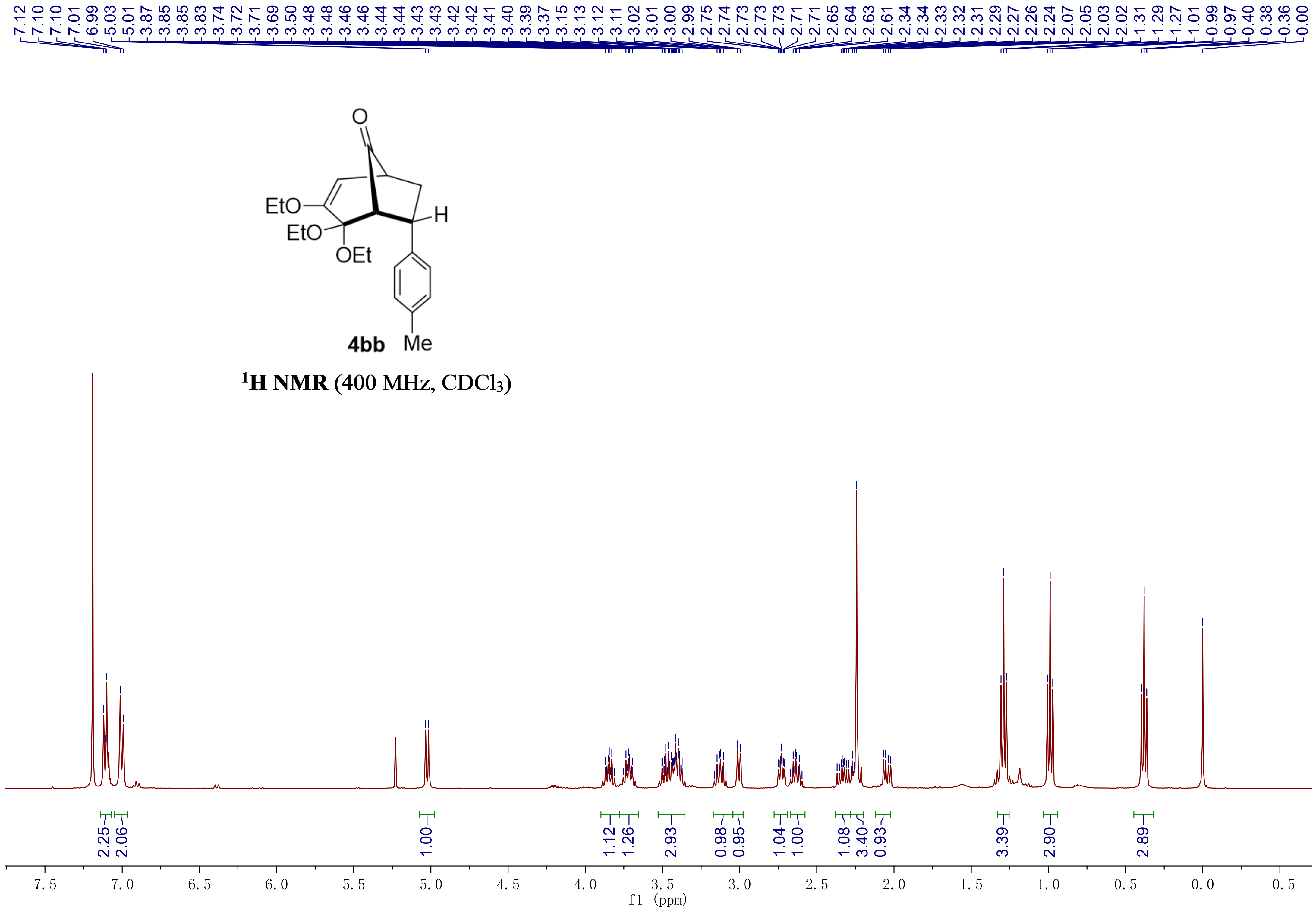
7.12 7.10 7.10 7.01 6.99 5.03 5.01 3.87 3.85 3.85 3.83 3.74 3.72 3.71 3.69 3.50 3.48 3.48 3.46 3.46 3.44 3.44 3.43 3.43 3.42 3.42 3.41 3.40 3.39 3.37 3.15 3.13 3.12 3.11 3.02 3.01 3.00 2.99 2.75 2.74 2.73 2.73 2.73 2.71 2.71 2.65 2.64 2.63 2.61 2.34 2.34 2.33 2.32 2.31 2.29 2.27 2.26 2.24 2.07 2.05 2.03 2.02 1.31 1.29 1.27 1.01 0.99 0.97 0.40 0.38 0.36 0.00
O
EtO
EtO
OEt
H
Me
4bb
1H NMR (400 MHz, CDCl3)
2.25 2.06 1.00 1.12 1.26 2.93 0.98 0.95 1.04 1.00 1.08 3.40 0.93 3.39 2.90 2.89
7.5 7.0 6.5 6.0 5.5 5.0 4.5 4.0 3.5 3.0 2.5 2.0 1.5 1.0 0.5 0.0 -0.5
f1 (ppm)

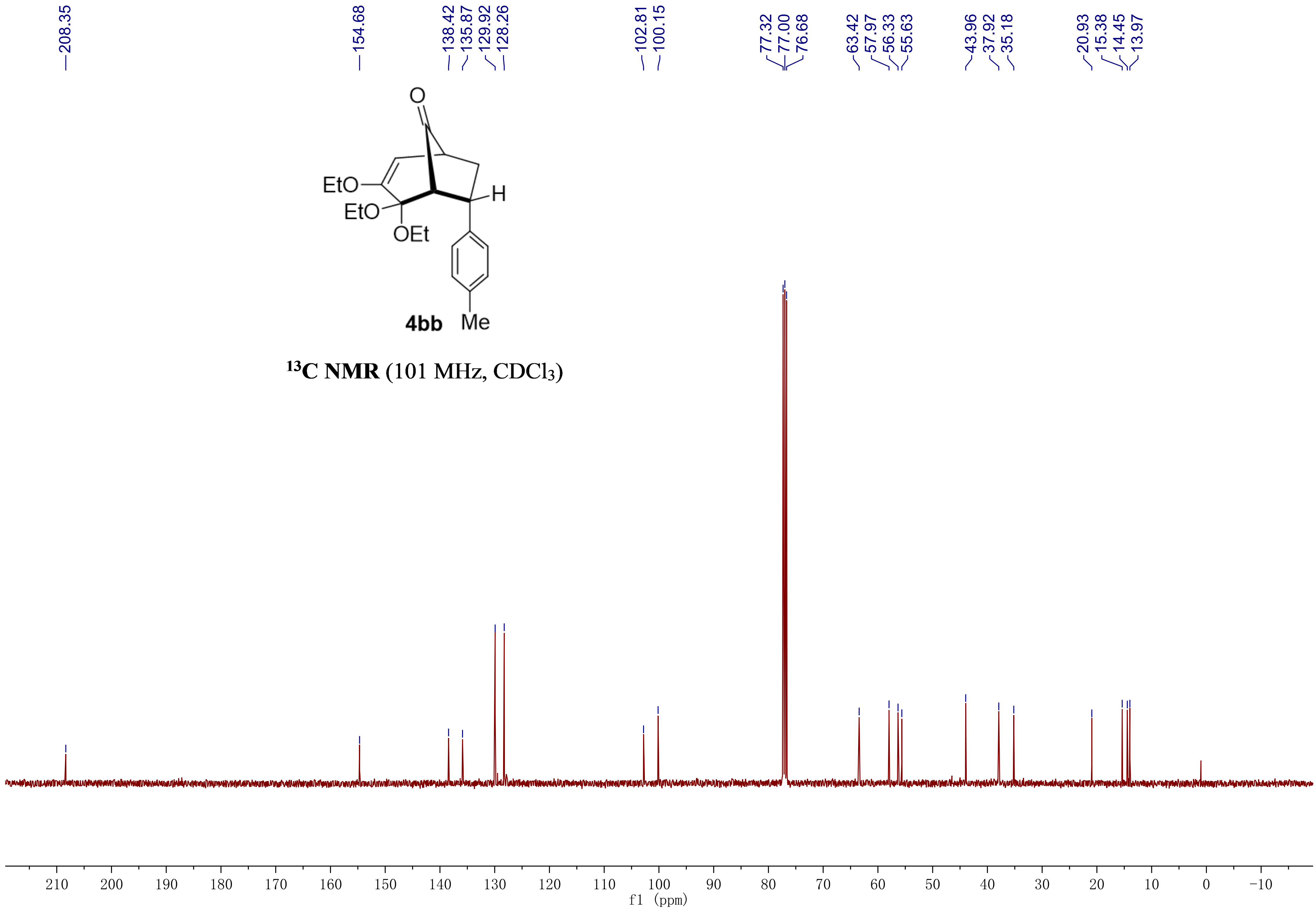

^{13}C NMR (101 MHz, $CDCl_3$)

===

Acq. Operator : SYSTEM Seq. Line : 34
Sample Operator : SYSTEM
Acq. Instrument : HPLC Location : P1-B-01
Injection Date : 2/12/2024 10:34:01 pm Inj : 1
Inj Volume : 2.000 µl
Different Inj Volume from Sample Entry! Actual Inj Volume : 5.000 µl
Method : C:\Users\Public\Documents\ChemStation\1\Data\SUN\SUN 2024-12-02 10-37-19
\ID3-10-20.M (Sequence Method)
Last changed : 2/8/2023 12:18:49 pm by SYSTEM
Additional Info : Peak(s) manually integrated

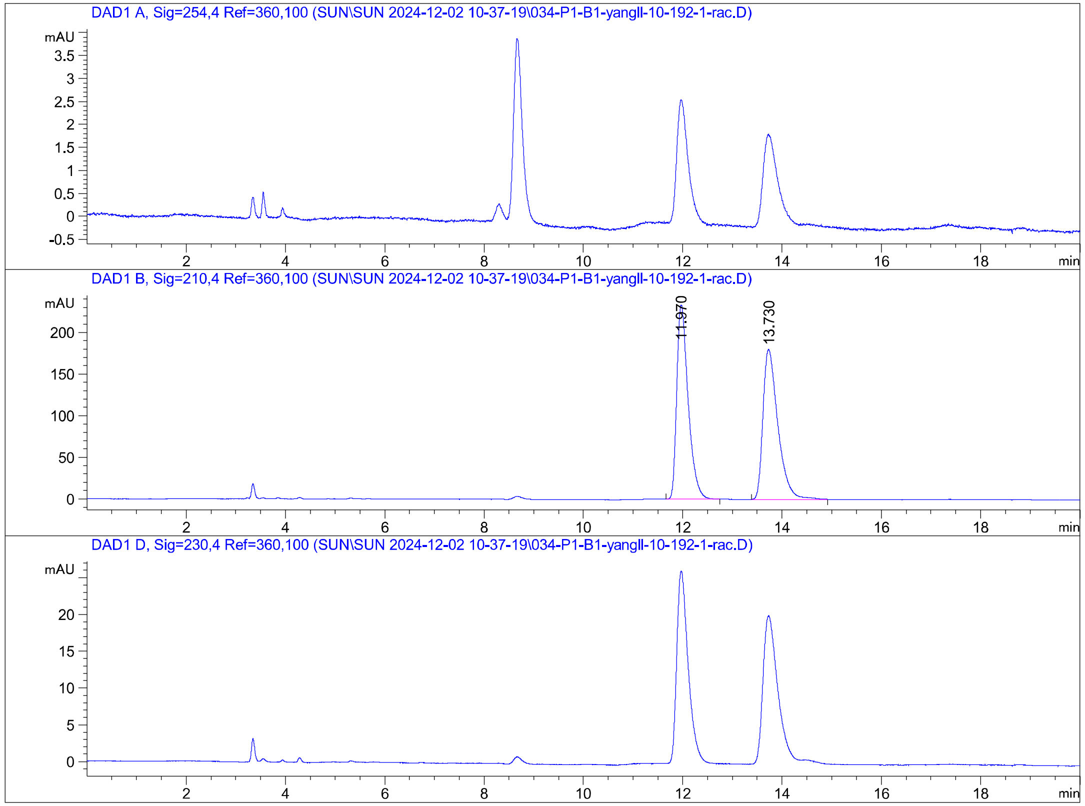

===

Area Percent Report

===

Sorted By : Signal
Multiplier : 1.0000
Dilution : 1.0000
Use Multiplier & Dilution Factor with ISTDs

Data File C:\Users\P...\1\Data\SUN\SUN 2024-12-02 10-37-19\034-P1-B1-yangll-10-192-1-rac.D
Sample Name: yangll-10-192-1-rac

Signal 1: DAD1 A, Sig=254,4 Ref=360,100

Signal 2: DAD1 B, Sig=210,4 Ref=360,100

Peak #	RetTime [min]	Type	Width [min]	Area [mAU*s]	Height [mAU]	Area %
1	11.970	BV R	0.2405	3722.54883	233.53807	49.7530
2	13.730	BB	0.3087	3759.51587	180.33566	50.2470
Totals :				7482.06470	413.87373	

Signal 3: DAD1 D, Sig=230,4 Ref=360,100

===

*** End of Report ***

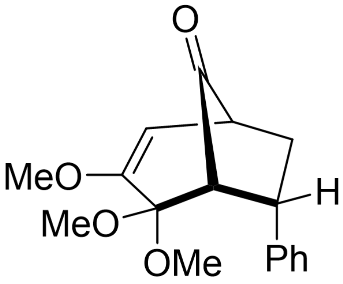

4a

rac

===

Acq. Operator : SYSTEM Seq. Line : 35
Sample Operator : SYSTEM
Acq. Instrument : HPLC Location : P1-B-02
Injection Date : 2/12/2024 10:55:04 pm Inj : 1
Inj Volume : 2.000 µl
Different Inj Volume from Sample Entry! Actual Inj Volume : 5.000 µl
Method : C:\Users\Public\Documents\ChemStation\1\Data\SUN\SUN 2024-12-02 10-37-19
\ID3-10-20.M (Sequence Method)
Last changed : 2/8/2023 12:18:49 pm by SYSTEM
Additional Info : Peak(s) manually integrated

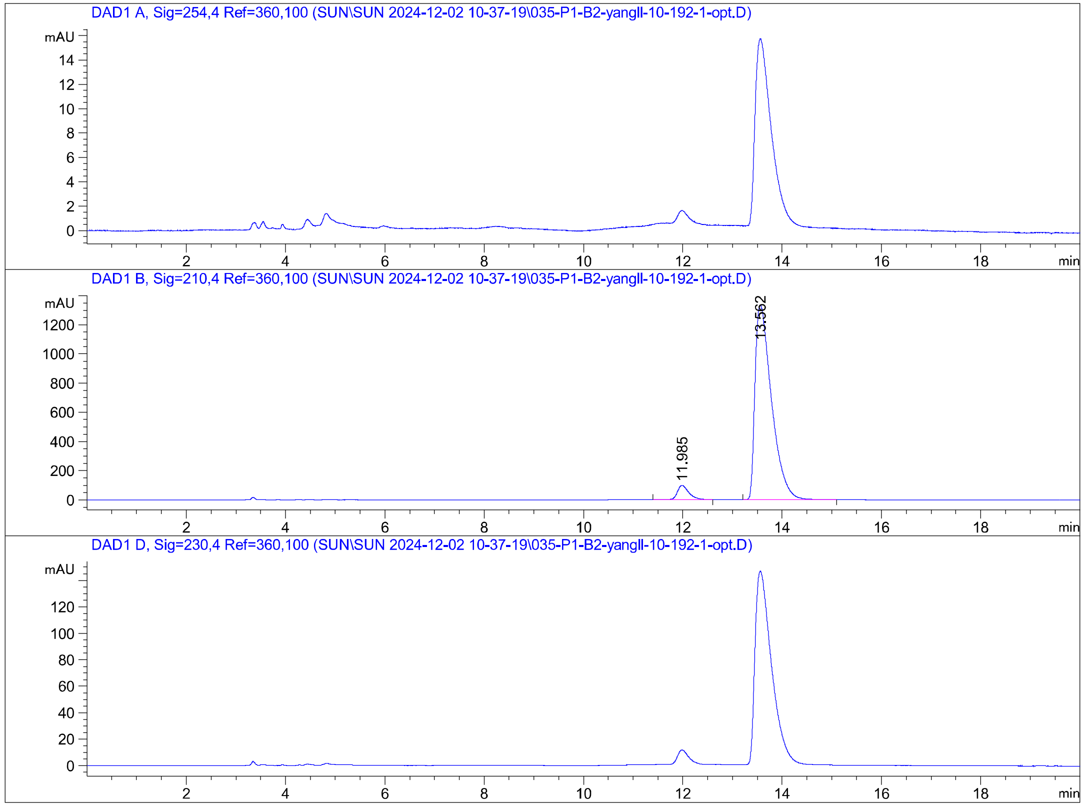

===

Area Percent Report

===

Sorted By : Signal
Multiplier : 1.0000
Dilution : 1.0000
Use Multiplier & Dilution Factor with ISTDs

Data File C:\Users\P...\1\Data\SUN\SUN 2024-12-02 10-37-19\035-P1-B2-yangll-10-192-1-opt.D
Sample Name: yangll-10-192-1-opt

Signal 1: DAD1 A, Sig=254,4 Ref=360,100

Signal 2: DAD1 B, Sig=210,4 Ref=360,100

Peak #	RetTime [min]	Type	Width [min]	Area [mAU*s]	Height [mAU]	Area %
1	11.985	VB R	0.2368	1543.93433	97.47881	4.7797
2	13.562	BV R	0.3178	3.07577e4	1334.76672	95.2203

Totals : 3.23017e4 1432.24554

Signal 3: DAD1 D, Sig=230,4 Ref=360,100

===

*** End of Report ***

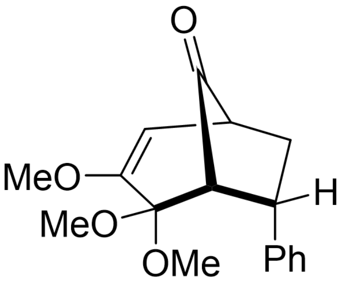

4a

opt

===

```
Acq. Operator   : SYSTEM                         Seq. Line :    2
Acq. Instrument : LC1260                          Location :   P1-A-01
Injection Date  : 2/15/2025 10:41:27 AM                 Inj :    1
                                                Inj Volume : 5.000 µl
Different Inj Volume from Sample Entry!  Actual Inj Volume : 40.000 µl
Acq. Method     : C:\Users\Public\Documents\ChemStation\1\Data\SUN\SUN 2025-02-15 10-28-26
                  \IBN3-30-30.M
Last changed    : 4/8/2024 3:14:14 PM by SYSTEM
Analysis Method : C:\Users\Public\Documents\ChemStation\1\Data\SUN\SUN 2025-02-15 10-28-26
                  \IBN3-30-30.M (Sequence Method)
Last changed    : 2/19/2025 9:42:04 AM by SYSTEM
                  (modified after loading)
Additional Info : Peak(s) manually integrated
```

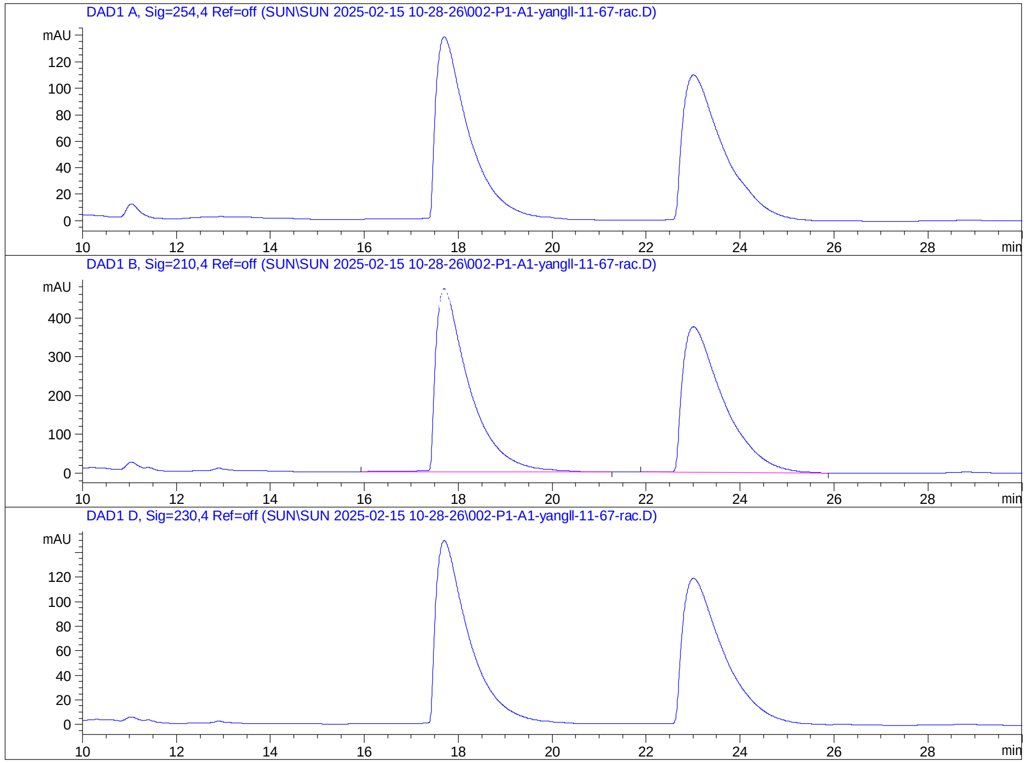

===

Area Percent Report

===

```
Sorted By             :      Signal
Multiplier            :      1.0000
Dilution              :      1.0000
Use Multiplier & Dilution Factor with ISTDs
```


Data File C:\Users\P...ion\1\Data\SUN\SUN 2025-02-15 10-28-26\002-P1-A1-yangll-11-67-rac.D
Sample Name: yangll-11-67-rac

Signal 1: DAD1 A, Sig=254,4 Ref=off

Signal 2: DAD1 B, Sig=210,4 Ref=off

Peak #	RetTime [min]	Type	Width [min]	Area [mAU*s]	Height [mAU]	Area %
1	17.699	BB	0.7175	2.32643e4	471.61127	50.7201
2	23.008	BB	0.9004	2.26037e4	375.42300	49.2799

Totals : 4.58680e4 847.03427

Signal 3: DAD1 D, Sig=230,4 Ref=off

===

*** End of Report ***

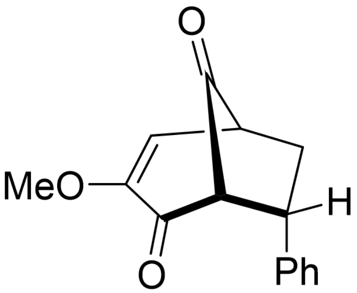

4a'

rac

Data File C:\Users\P...ion\1\Data\SUN\SUN 2025-02-15 10-28-26\003-P1-A2-yangll-11-67-opt.D
Sample Name: yangll-11-67-opt

===

Acq. Operator : SYSTEM   Seq. Line : 3
Acq. Instrument : LC1260   Location : P1-A-02
Injection Date : 2/15/2025 11:12:48 AM   Inj : 1
Inj Volume : 5.000 µl
Different Inj Volume from Sample Entry! Actual Inj Volume : 40.000 µl
Acq. Method : C:\Users\Public\Documents\ChemStation\1\Data\SUN\SUN 2025-02-15 10-28-26\IBN3-30-30.M
Last changed : 4/8/2024 3:14:14 PM by SYSTEM
Analysis Method : C:\Users\Public\Documents\ChemStation\1\Data\SUN\SUN 2025-02-15 10-28-26\IBN3-30-30.M (Sequence Method)
Last changed : 2/19/2025 9:42:04 AM by SYSTEM
(modified after loading)
Additional Info : Peak(s) manually integrated

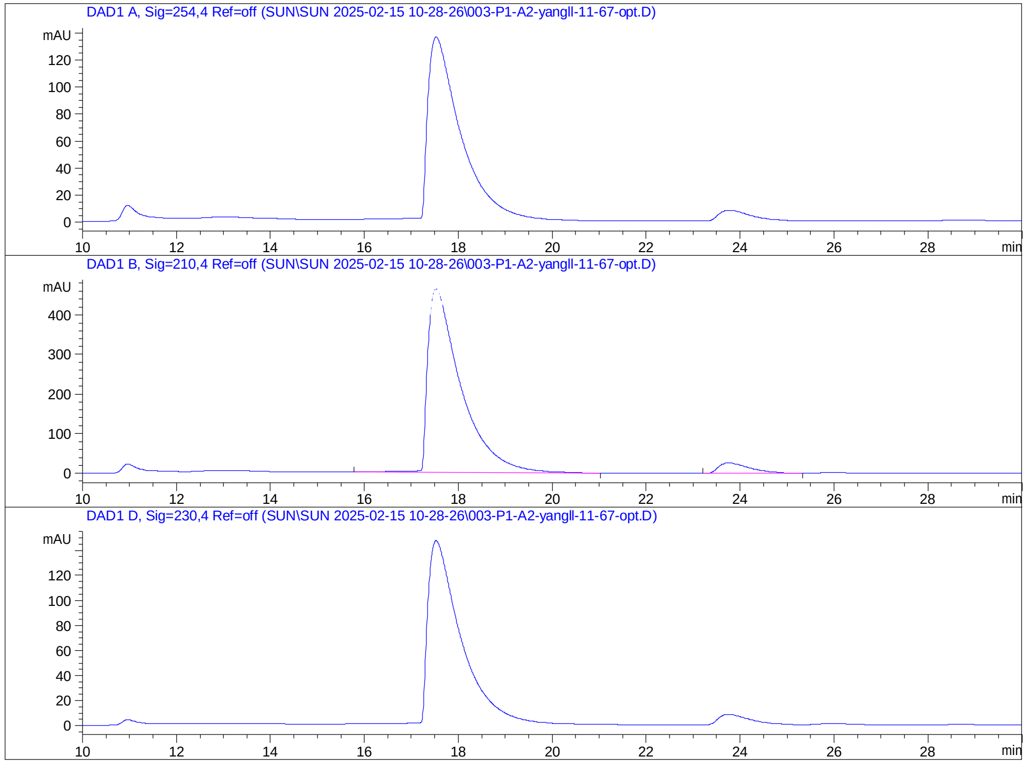

===

Area Percent Report

===

Sorted By : Signal
Multiplier : 1.0000
Dilution : 1.0000
Use Multiplier & Dilution Factor with ISTDs

Data File C:\Users\P...ion\1\Data\SUN\SUN 2025-02-15 10-28-26\003-P1-A2-yangll-11-67-opt.D
Sample Name: yangll-11-67-opt

Signal 1: DAD1 A, Sig=254,4 Ref=off

Signal 2: DAD1 B, Sig=210,4 Ref=off

Peak #	RetTime [min]	Type	Width [min]	Area [mAU*s]	Height [mAU]	Area %
1	17.531	BB	0.7128	2.25925e4	463.42691	95.0026
2	23.752	BB	0.6914	1188.42102	25.89807	4.9974

Totals : 2.37810e4 489.32498

Signal 3: DAD1 D, Sig=230,4 Ref=off

===

*** End of Report ***

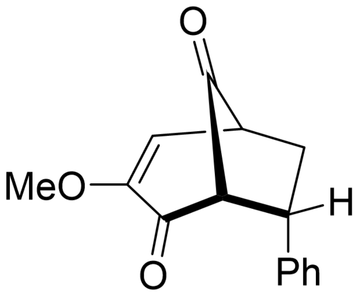

4a'

opt

===

```
Acq. Operator   : SYSTEM                        Seq. Line :  52
Sample Operator : SYSTEM
Acq. Instrument : HPLC                           Location :   P1-B-06
Injection Date  : 10/1/2024 6:54:00 am                Inj :    1
                                              Inj Volume : 2.000 µl
Acq. Method     : C:\Users\Public\Documents\ChemStation\1\Data\SUN\SUN 2024-01-09 09-29-51
                  \ID3-10-30.M
Last changed    : 16/8/2022 10:05:02 am by SYSTEM
Analysis Method : C:\Users\Public\Documents\ChemStation\1\Data\SUN\SUN 2024-01-09 09-29-51
                  \ID3-10-30.M (Sequence Method)
Last changed    : 16/3/2024 3:25:02 pm by SYSTEM
                  (modified after loading)
Additional Info : Peak(s) manually integrated
```

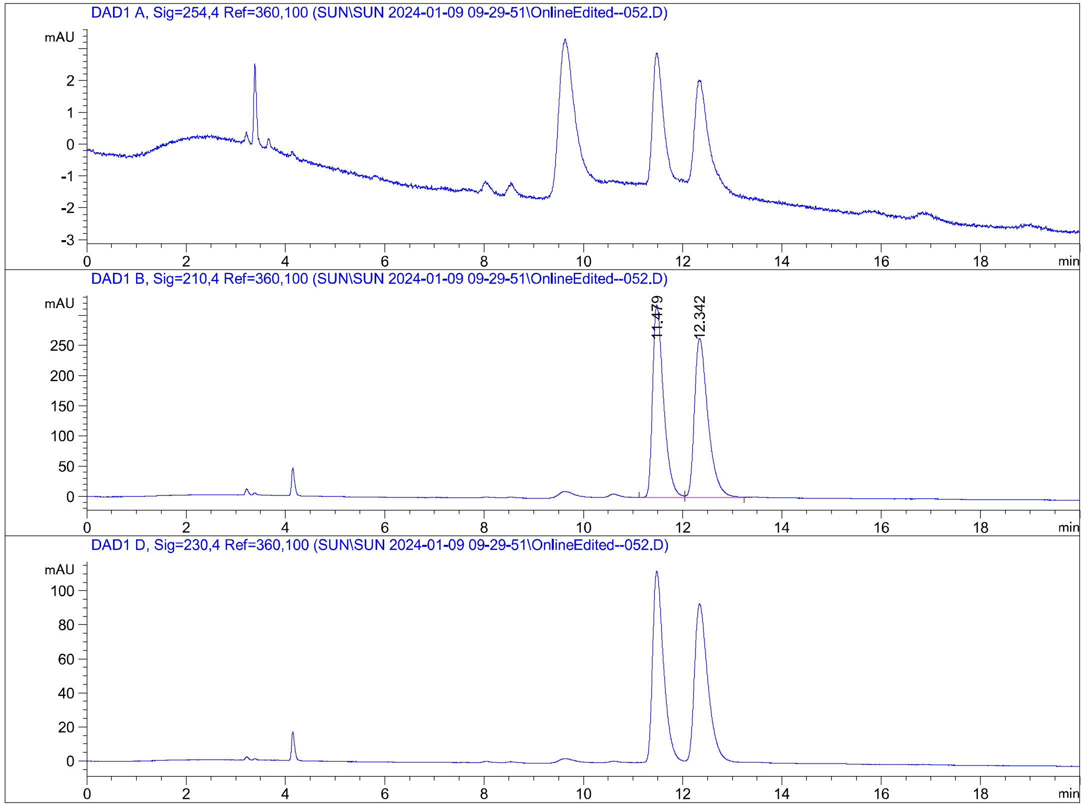

===

Area Percent Report

===

```
Sorted By             :      Signal
Multiplier            :      1.0000
Dilution              :      1.0000
Use Multiplier & Dilution Factor with ISTDs
```


Data File C:\Users\P...\ChemStation\1\Data\SUN\SUN 2024-01-09 09-29-51\OnlineEdited--052.D
Sample Name: yangll-8-173-2-rac

Signal 1: DAD1 A, Sig=254,4 Ref=360,100

Signal 2: DAD1 B, Sig=210,4 Ref=360,100

Peak #	RetTime [min]	Type	Width [min]	Area [mAU*s]	Height [mAU]	Area %
1	11.479	BV	0.2304	4883.15234	318.46036	49.6194
2	12.342	VB	0.2753	4958.06543	263.21414	50.3806

Totals : 9841.21777 581.67450

Signal 3: DAD1 D, Sig=230,4 Ref=360,100

===

*** End of Report ***

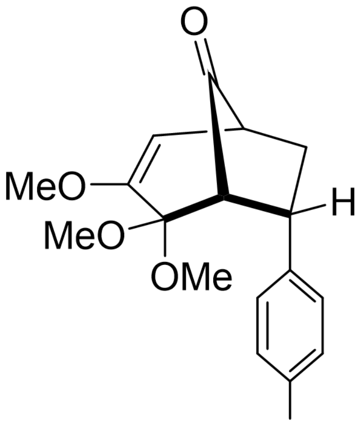

4b

rac

===

Acq. Operator : SYSTEM   Seq. Line : 32
Sample Operator : SYSTEM
Acq. Instrument : HPLC   Location : P1-A-01
Injection Date : 19/3/2024 10:42:57 am   Inj : 1
Inj Volume : 2.000 µl
Different Inj Volume from Sample Entry! Actual Inj Volume : 4.000 µl
Method : C:\Users\Public\Documents\ChemStation\1\Data\SUN\SUN 2024-03-18 22-29-36\ID3-10-20.M (Sequence Method)
Last changed : 2/8/2023 12:18:49 pm by SYSTEM
Additional Info : Peak(s) manually integrated

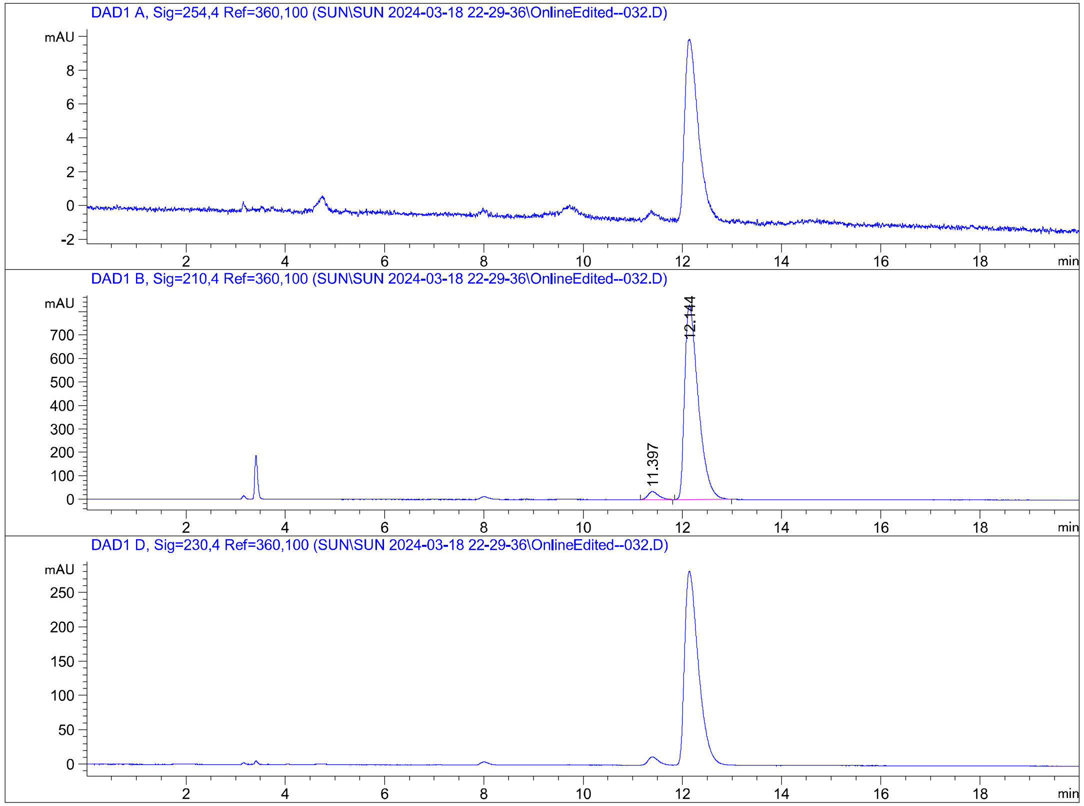

===

Area Percent Report

===

Sorted By : Signal
Multiplier : 1.0000
Dilution : 1.0000
Use Multiplier & Dilution Factor with ISTDs

Data File C:\Users\P...\ChemStation\1\Data\SUN\SUN 2024-03-18 22-29-36\OnlineEdited--032.D
Sample Name: yangll-9-66-opt

Signal 1: DAD1 A, Sig=254,4 Ref=360,100

Signal 2: DAD1 B, Sig=210,4 Ref=360,100

Peak #	RetTime [min]	Type	Width [min]	Area [mAU*s]	Height [mAU]	Area %
1	11.397	VV R	0.1772	507.09100	34.55518	3.1109
2	12.144	VV R	0.2257	1.57935e4	829.07111	96.8891

Totals : 1.63006e4 863.62629

Signal 3: DAD1 D, Sig=230,4 Ref=360,100

===

*** End of Report ***

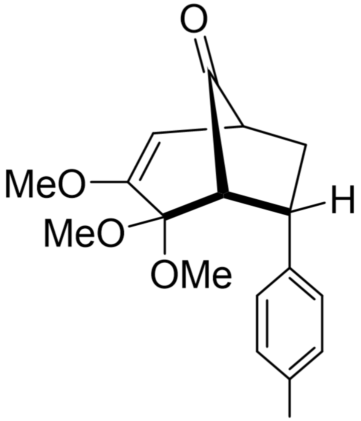

4b

opt

===

Acq. Operator : SYSTEM Seq. Line : 49
Sample Operator : SYSTEM
Acq. Instrument : HPLC Location : P1-B-03
Injection Date : 10/1/2024 5:20:59 am Inj : 1
Inj Volume : 2.000 µl
Acq. Method : C:\Users\Public\Documents\ChemStation\1\Data\SUN\SUN 2024-01-09 09-29-51\ID3-10-30.M
Last changed : 16/8/2022 10:05:02 am by SYSTEM
Analysis Method : C:\Users\Public\Documents\ChemStation\1\Data\SUN\SUN 2024-01-09 09-29-51\ID3-10-30.M (Sequence Method)
Last changed : 16/3/2024 3:25:02 pm by SYSTEM
(modified after loading)
Additional Info : Peak(s) manually integrated

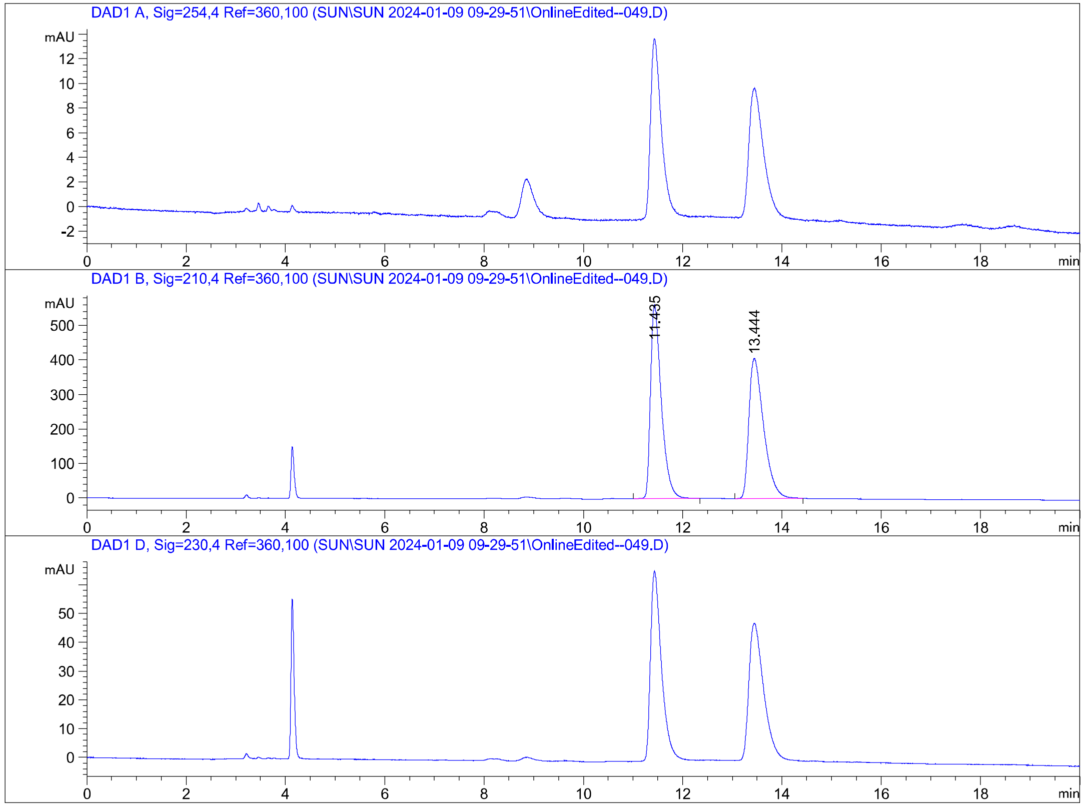

===

Area Percent Report

===

Sorted By : Signal
Multiplier : 1.0000
Dilution : 1.0000
Use Multiplier & Dilution Factor with ISTDs

Data File C:\Users\P...\ChemStation\1\Data\SUN\SUN 2024-01-09 09-29-51\OnlineEdited--049.D
Sample Name: yangll-8-170-2-rac

Signal 1: DAD1 A, Sig=254,4 Ref=360,100

Signal 2: DAD1 B, Sig=210,4 Ref=360,100

Peak #	RetTime [min]	Type	Width [min]	Area [mAU*s]	Height [mAU]	Area %
1	11.435	BV R	0.2285	8517.11621	561.38129	50.3680
2	13.444	VV R	0.2947	8392.64746	405.90927	49.6320

Totals : 1.69098e4 967.29056

Signal 3: DAD1 D, Sig=230,4 Ref=360,100

===

*** End of Report ***

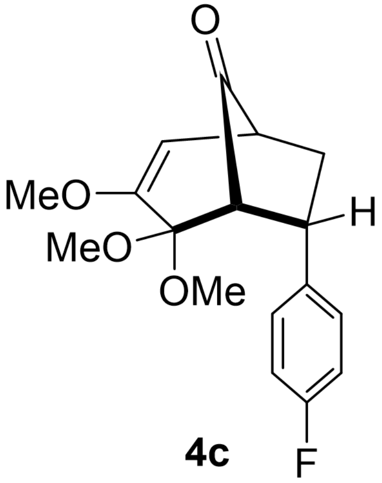

4c

rac

===

Acq. Operator : SYSTEM   Seq. Line : 33
Sample Operator : SYSTEM
Acq. Instrument : HPLC   Location : P1-A-02
Injection Date : 19/3/2024 11:04:17 am   Inj : 1
Inj Volume : 2.000 µl
Different Inj Volume from Sample Entry! Actual Inj Volume : 4.000 µl
Method : C:\Users\Public\Documents\ChemStation\1\Data\SUN\SUN 2024-03-18 22-29-36\ID3-10-20.M (Sequence Method)
Last changed : 2/8/2023 12:18:49 pm by SYSTEM
Additional Info : Peak(s) manually integrated

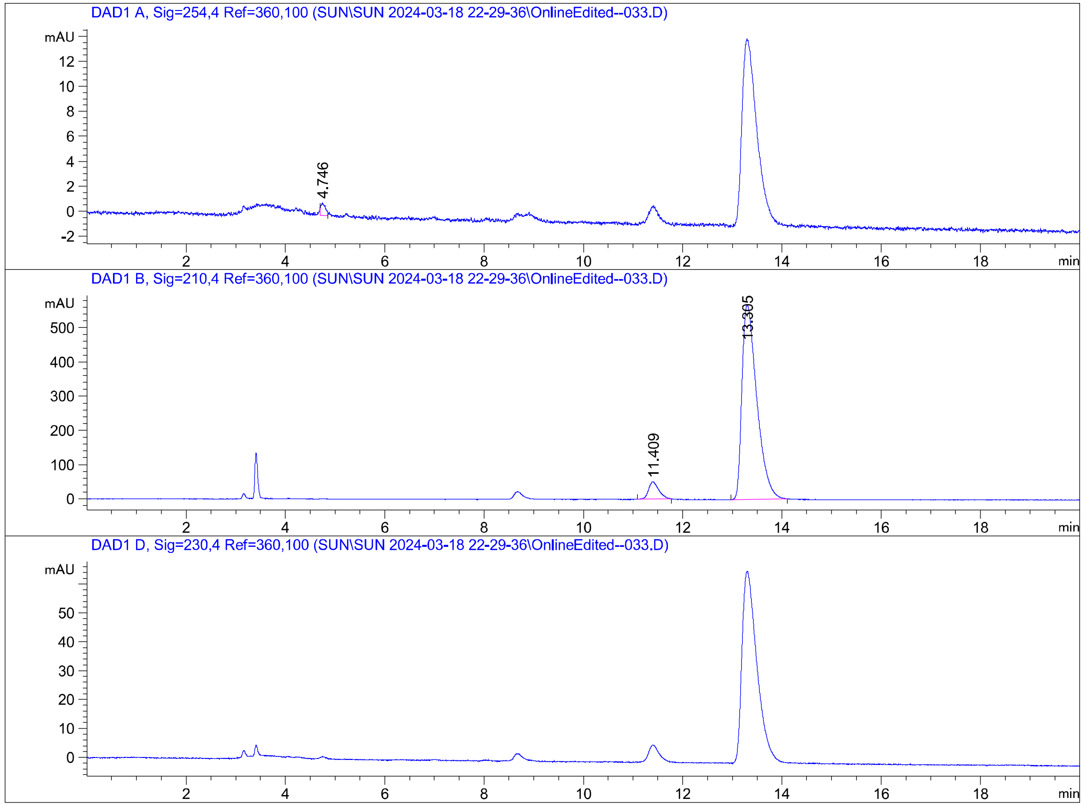

===
Area Percent Report
===

Sorted By : Signal
Multiplier : 1.0000
Dilution : 1.0000
Use Multiplier & Dilution Factor with ISTDs

Data File C:\Users\P...\ChemStation\1\Data\SUN\SUN 2024-03-18 22-29-36\OnlineEdited--033.D
Sample Name: yangll-9-67-opt

Signal 1: DAD1 A, Sig=254,4 Ref=360,100

Peak #	RetTime [min]	Type	Width [min]	Area [mAU*s]	Height [mAU]	Area %
1	4.746	VV	0.0779	6.40648	1.01135	100.0000

Totals : 6.40648 1.01135

Signal 2: DAD1 B, Sig=210,4 Ref=360,100

Peak #	RetTime [min]	Type	Width [min]	Area [mAU*s]	Height [mAU]	Area %
1	11.409	VV R	0.1721	727.30151	50.33514	5.8019
2	13.305	BV R	0.2680	1.18083e4	568.09595	94.1981

Totals : 1.25356e4 618.43109

Signal 3: DAD1 D, Sig=230,4 Ref=360,100

===
*** End of Report ***

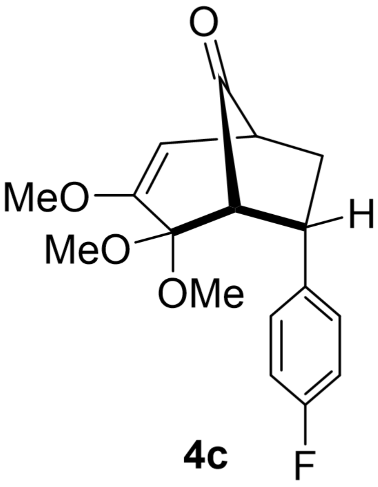

4c

opt

===

Acq. Operator : SYSTEM Seq. Line : 3
Sample Operator : SYSTEM
Acq. Instrument : HPLC Location : P1-B-02
Injection Date : 12/1/2024 6:05:37 pm Inj : 1
Inj Volume : 2.000 µl
Method : C:\Users\Public\Documents\ChemStation\1\Data\SUN\SUN 2024-01-12 17-31-45\ID3-10-20.M (Sequence Method)
Last changed : 2/8/2023 12:18:49 pm by SYSTEM
Additional Info : Peak(s) manually integrated

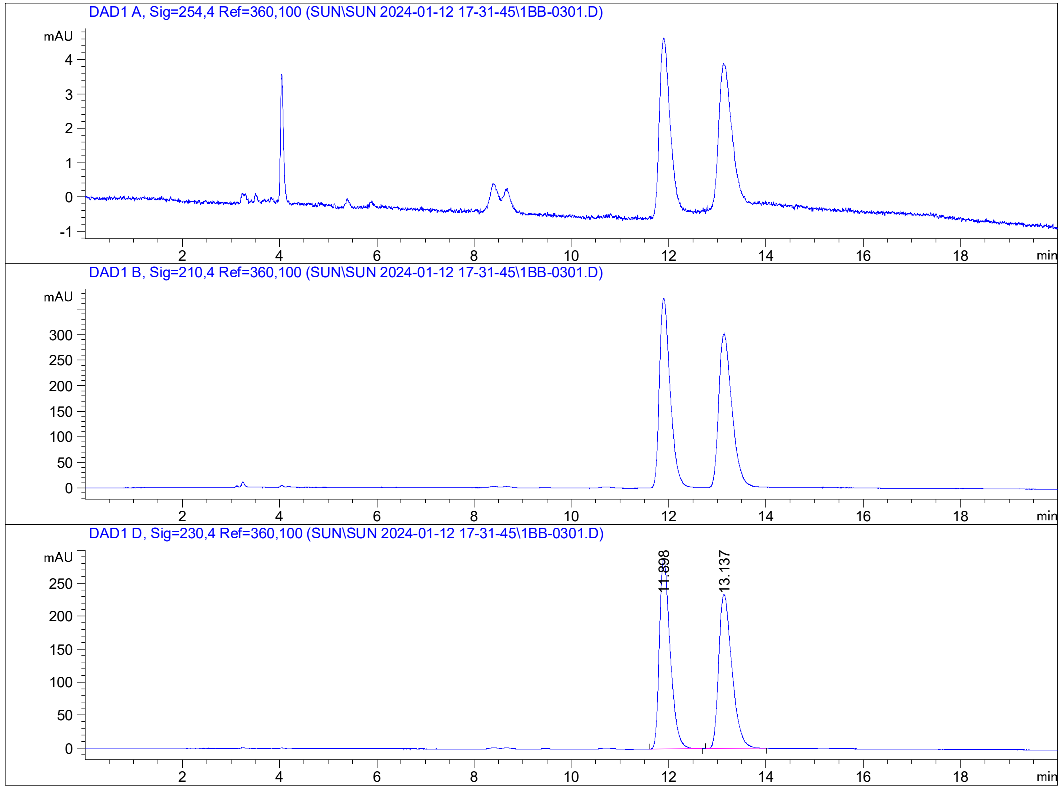

===

Area Percent Report

===

Sorted By : Signal
Multiplier : 1.0000
Dilution : 1.0000
Use Multiplier & Dilution Factor with ISTDs

Data File C:\Users\P...Documents\ChemStation\1\Data\SUN\SUN 2024-01-12 17-31-45\1BB-0301.D
Sample Name: yangll-8-180-2-rac-ID

Signal 1: DAD1 A, Sig=254,4 Ref=360,100

Signal 2: DAD1 B, Sig=210,4 Ref=360,100

Signal 3: DAD1 D, Sig=230,4 Ref=360,100

Peak #	RetTime [min]	Type	Width [min]	Area [mAU*s]	Height [mAU]	Area %
1	11.898	BV R	0.2333	4443.05322	288.35214	50.0736
2	13.137	VB R	0.2817	4429.99219	234.14697	49.9264

Totals : 8873.04541 522.49911

===

*** End of Report ***

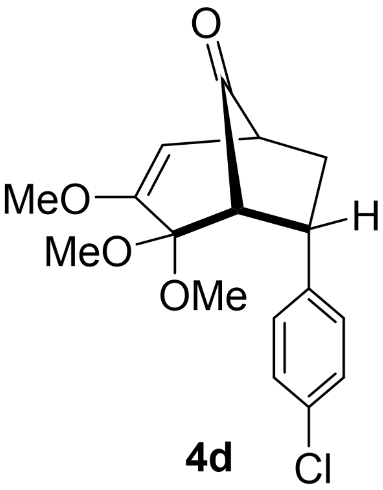

4d

rac

===

```
Acq. Operator   : SYSTEM                         Seq. Line :    9
Sample Operator : SYSTEM
Acq. Instrument : HPLC                            Location :   P1-F-01
Injection Date  : 26/11/2024 2:40:22 pm                Inj :    1
                                               Inj Volume : 2.000 µl
Different Inj Volume from Sample Entry!  Actual Inj Volume : 5.000 µl
Method          : C:\Users\Public\Documents\ChemStation\1\Data\SUN\SUN 2024-11-26 11-44-19
                  \ID3-10-20.M (Sequence Method)
Last changed    : 2/8/2023 12:18:49 pm by SYSTEM
Additional Info : Peak(s) manually integrated
```

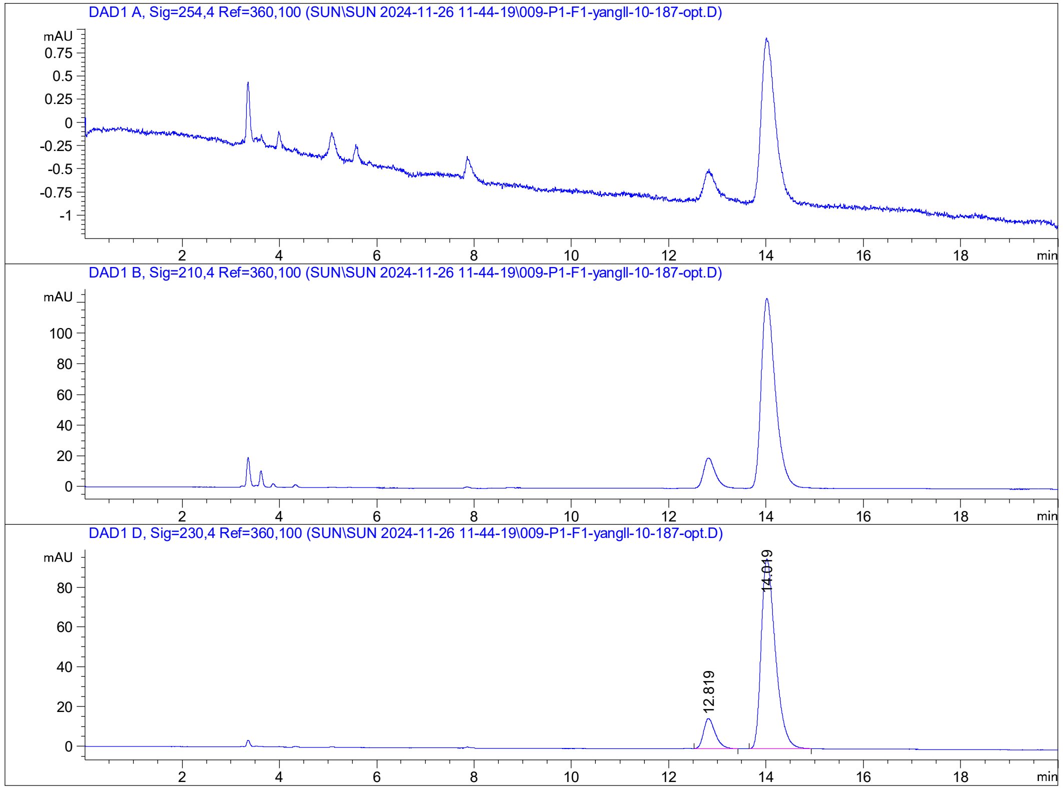

===

Area Percent Report

===

```
Sorted By            :      Signal
Multiplier           :      1.0000
Dilution             :      1.0000
Use Multiplier & Dilution Factor with ISTDs
```


Data File C:\Users\P...on\1\Data\SUN\SUN 2024-11-26 11-44-19\009-P1-F1-yangll-10-187-opt.D
Sample Name: yangll-10-187-opt

Signal 1: DAD1 A, Sig=254,4 Ref=360,100

Signal 2: DAD1 B, Sig=210,4 Ref=360,100

Signal 3: DAD1 D, Sig=230,4 Ref=360,100

Peak #	RetTime [min]	Type	Width [min]	Area [mAU*s]	Height [mAU]	Area %
1	12.819	BB	0.2365	252.83064	15.16660	11.5604
2	14.019	BB	0.3075	1934.20581	95.41724	88.4396

Totals : 2187.03645 110.58384

===

*** End of Report ***

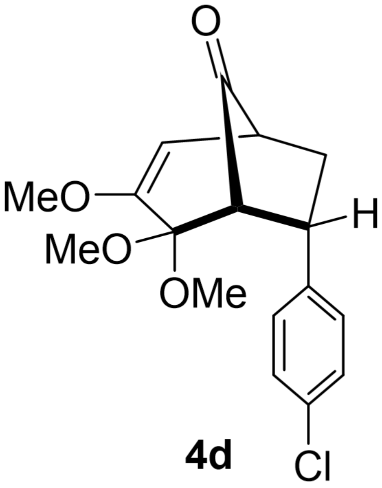

4d

opt

===

Acq. Operator : SYSTEM Seq. Line : 51
Sample Operator : SYSTEM
Acq. Instrument : HPLC Location : P1-B-05
Injection Date : 10/1/2024 6:23:05 am Inj : 1
Inj Volume : 2.000 µl
Method : C:\Users\Public\Documents\ChemStation\1\Data\SUN\SUN 2024-01-09 09-29-51 \ID3-10-30.M (Sequence Method)
Last changed : 16/8/2022 10:05:02 am by SYSTEM
Additional Info : Peak(s) manually integrated

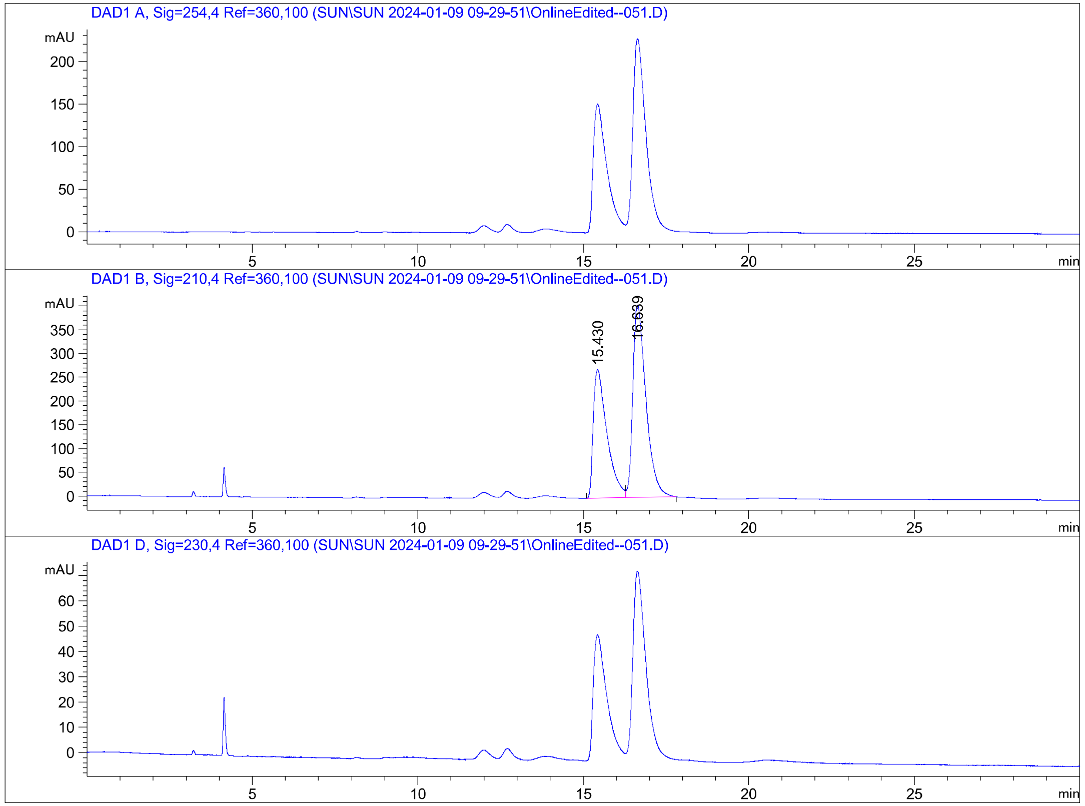

===

Area Percent Report

===

Sorted By : Signal
Multiplier : 1.0000
Dilution : 1.0000
Use Multiplier & Dilution Factor with ISTDs

Data File C:\Users\P...\ChemStation\1\Data\SUN\SUN 2024-01-09 09-29-51\OnlineEdited--051.D
Sample Name: yangll-8-172-2-rac

Signal 1: DAD1 A, Sig=254,4 Ref=360,100

Signal 2: DAD1 B, Sig=210,4 Ref=360,100

Peak #	RetTime [min]	Type	Width [min]	Area [mAU*s]	Height [mAU]	Area %
1	15.430	BV R	0.4040	7788.28516	270.84821	40.9519
2	16.639	VB	0.3864	1.12299e4	405.16495	59.0481

Totals : 1.90181e4 676.01315

Signal 3: DAD1 D, Sig=230,4 Ref=360,100

===

*** End of Report ***

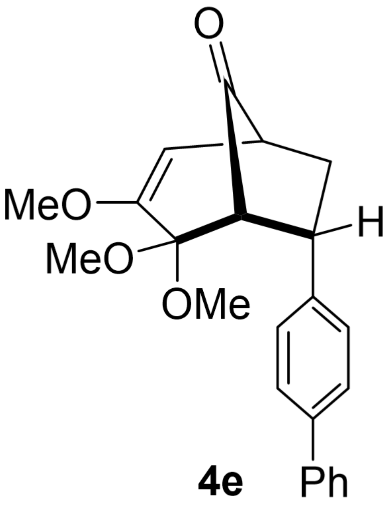

4e

rac

===

Acq. Operator : SYSTEM   Seq. Line : 34
Sample Operator : SYSTEM
Acq. Instrument : HPLC   Location : P1-A-03
Injection Date : 19/3/2024 11:25:30 am   Inj : 1
Inj Volume : 2.000 µl
Different Inj Volume from Sample Entry! Actual Inj Volume : 4.000 µl
Method : C:\Users\Public\Documents\ChemStation\1\Data\SUN\SUN 2024-03-18 22-29-36\ID3-10-30.M (Sequence Method)
Last changed : 16/8/2022 10:05:02 am by SYSTEM
Additional Info : Peak(s) manually integrated

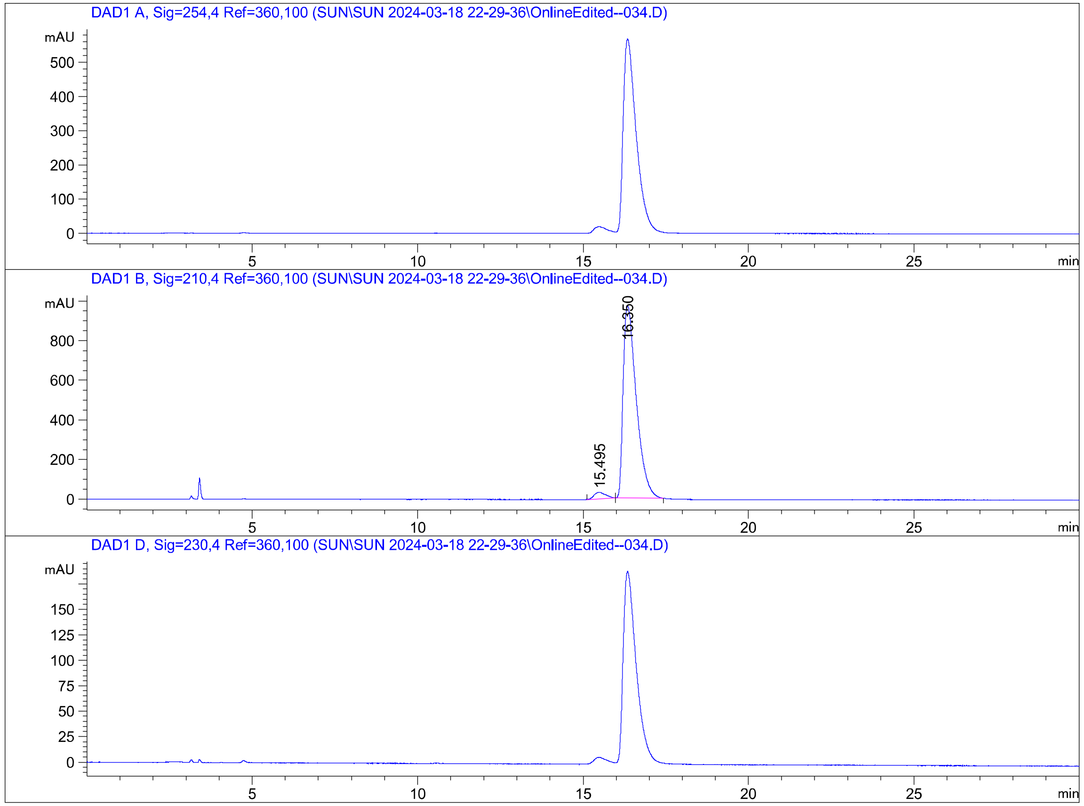

===

Area Percent Report

===

Sorted By : Signal
Multiplier : 1.0000
Dilution : 1.0000
Use Multiplier & Dilution Factor with ISTDs

Data File C:\Users\P...\ChemStation\1\Data\SUN\SUN 2024-03-18 22-29-36\OnlineEdited--034.D
Sample Name: yangll-9-68-opt

Signal 1: DAD1 A, Sig=254,4 Ref=360,100

Signal 2: DAD1 B, Sig=210,4 Ref=360,100

Peak #	RetTime [min]	Type	Width [min]	Area [mAU*s]	Height [mAU]	Area %
1	15.495	MM R	0.4141	800.87793	32.23029	2.9059
2	16.350	BV R	0.3241	2.67598e4	975.32837	97.0941

Totals : 2.75607e4 1007.55866

Signal 3: DAD1 D, Sig=230,4 Ref=360,100

===

*** End of Report ***

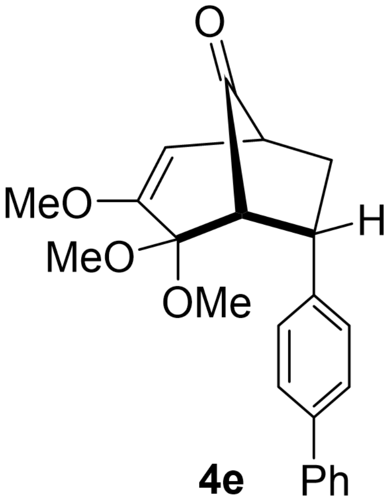

4e

opt

===

Acq. Operator : SYSTEM Seq. Line : 11
Sample Operator : SYSTEM
Acq. Instrument : HPLC Location : P1-B-01
Injection Date : 12/1/2024 8:07:25 pm Inj : 1
Inj Volume : 2.000 µl
Acq. Method : C:\Users\Public\Documents\ChemStation\1\Data\SUN\SUN 2024-01-12 17-31-45\IBN3-10-20.M
Last changed : 12/1/2024 8:17:37 pm by SYSTEM
(modified after loading)
Analysis Method : C:\Users\Public\Documents\ChemStation\1\Data\SUN\SUN 2024-01-12 17-31-45\IBN3-10-20.M (Sequence Method)
Last changed : 12/1/2024 8:22:33 pm by SYSTEM
Additional Info : Peak(s) manually integrated

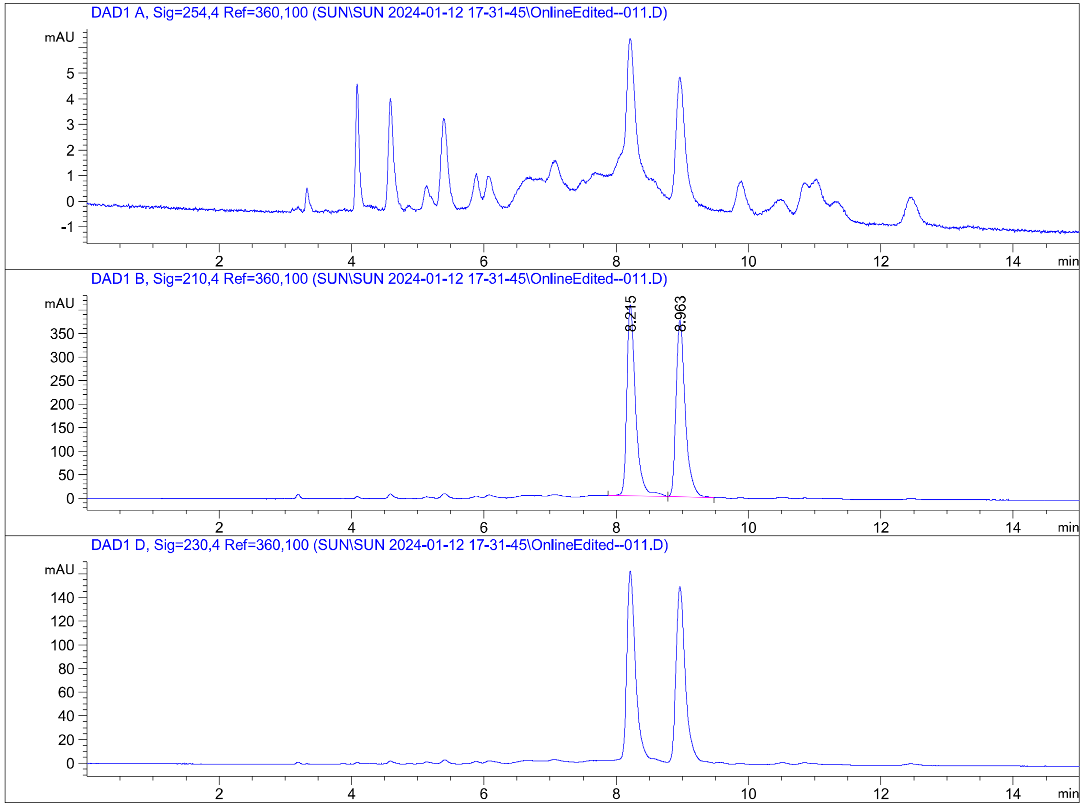

===

Area Percent Report

===

Sorted By : Signal
Multiplier : 1.0000
Dilution : 1.0000
Use Multiplier & Dilution Factor with ISTDs

Data File C:\Users\P...\ChemStation\1\Data\SUN\SUN 2024-01-12 17-31-45\OnlineEdited--011.D
Sample Name: yangll-8-179-2-rac-IB

Signal 1: DAD1 A, Sig=254,4 Ref=360,100

Signal 2: DAD1 B, Sig=210,4 Ref=360,100

Peak #	RetTime [min]	Type	Width [min]	Area [mAU*s]	Height [mAU]	Area %
1	8.215	VV R	0.1358	3736.03516	405.47830	50.9429
2	8.963	BB	0.1447	3597.73779	373.97403	49.0571

Totals : 7333.77295 779.45233

Signal 3: DAD1 D, Sig=230,4 Ref=360,100

===

*** End of Report ***

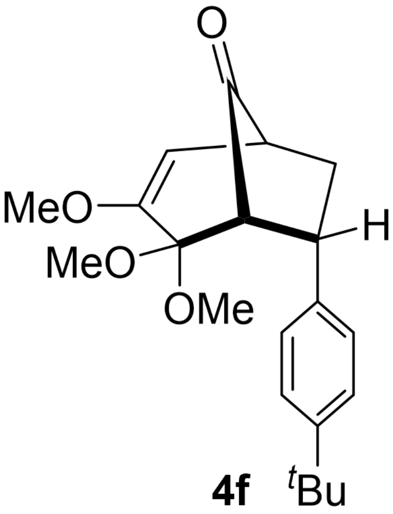

4f

rac

===

Acq. Operator : SYSTEM Seq. Line : 37
Sample Operator : SYSTEM
Acq. Instrument : HPLC Location : P1-A-04
Injection Date : 19/3/2024 12:39:15 pm Inj : 1
Inj Volume : 2.000 µl
Different Inj Volume from Sample Entry! Actual Inj Volume : 4.000 µl
Acq. Method : C:\Users\Public\Documents\ChemStation\1\Data\SUN\SUN 2024-03-18 22-29-36 \IBN3-10-20.M
Last changed : 30/7/2023 1:22:19 pm by SYSTEM
Analysis Method : C:\Users\Public\Documents\ChemStation\1\Data\SUN\SUN 2024-03-18 22-29-36 \IBN3-10-20.M (Sequence Method)
Last changed : 19/3/2024 8:24:17 pm by SYSTEM
(modified after loading)
Additional Info : Peak(s) manually integrated

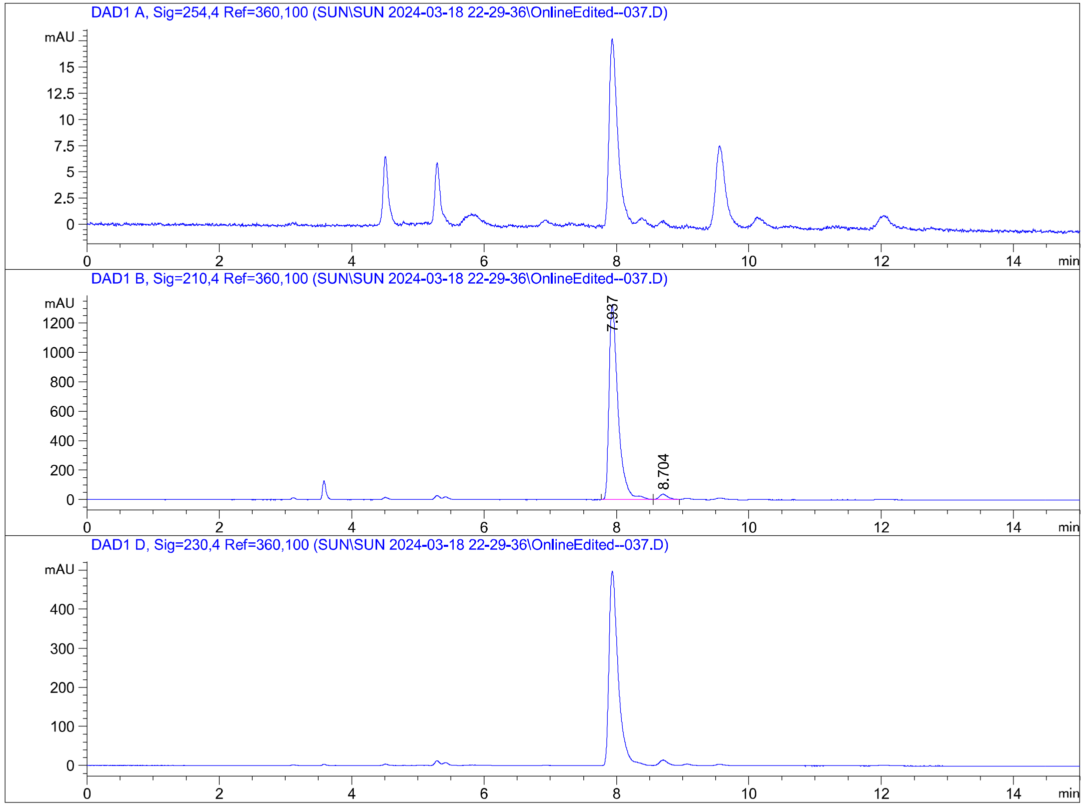

Data File C:\Users\P...\ChemStation\1\Data\SUN\SUN 2024-03-18 22-29-36\OnlineEdited--037.D
Sample Name: yangll-9-69-opt

===

Area Percent Report

===

Sorted By : Signal
Multiplier : 1.0000
Dilution : 1.0000
Use Multiplier & Dilution Factor with ISTDs

Signal 1: DAD1 A, Sig=254,4 Ref=360,100

Signal 2: DAD1 B, Sig=210,4 Ref=360,100

Peak #	RetTime [min]	Type	Width [min]	Area [mAU*s]	Height [mAU]	Area %
1	7.937	BV R	0.1284	1.25020e4	1324.76892	97.6185
2	8.704	BB	0.1176	304.99841	35.37582	2.3815

Totals : 1.28070e4 1360.14474

Signal 3: DAD1 D, Sig=230,4 Ref=360,100

===

*** End of Report ***

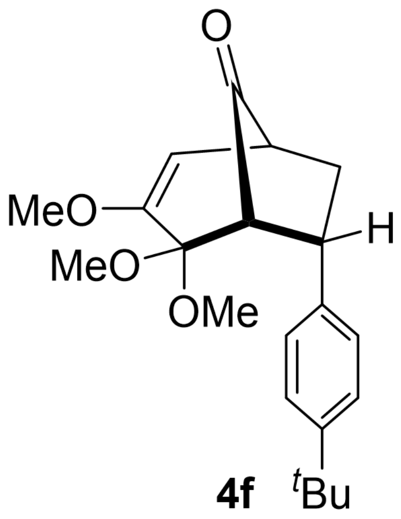

4f

opt

===

Acq. Operator : SYSTEM   Seq. Line : 2
Sample Operator : SYSTEM
Acq. Instrument : HPLC   Location : P1-F-02
Injection Date : 28/1/2024 9:09:15 pm   Inj : 1
Inj Volume : 2.000 µl
Acq. Method : C:\Users\Public\Documents\ChemStation\1\Data\SUN\SUN 2024-01-28 20-56-17\IBN3-10-20.M
Last changed : 30/7/2023 1:22:19 pm by SYSTEM
Analysis Method : C:\Users\Public\Documents\ChemStation\1\Data\SUN\SUN 2024-01-28 20-56-17\IBN3-10-20.M (Sequence Method)
Last changed : 19/3/2024 8:37:52 pm by SYSTEM
(modified after loading)
Additional Info : Peak(s) manually integrated

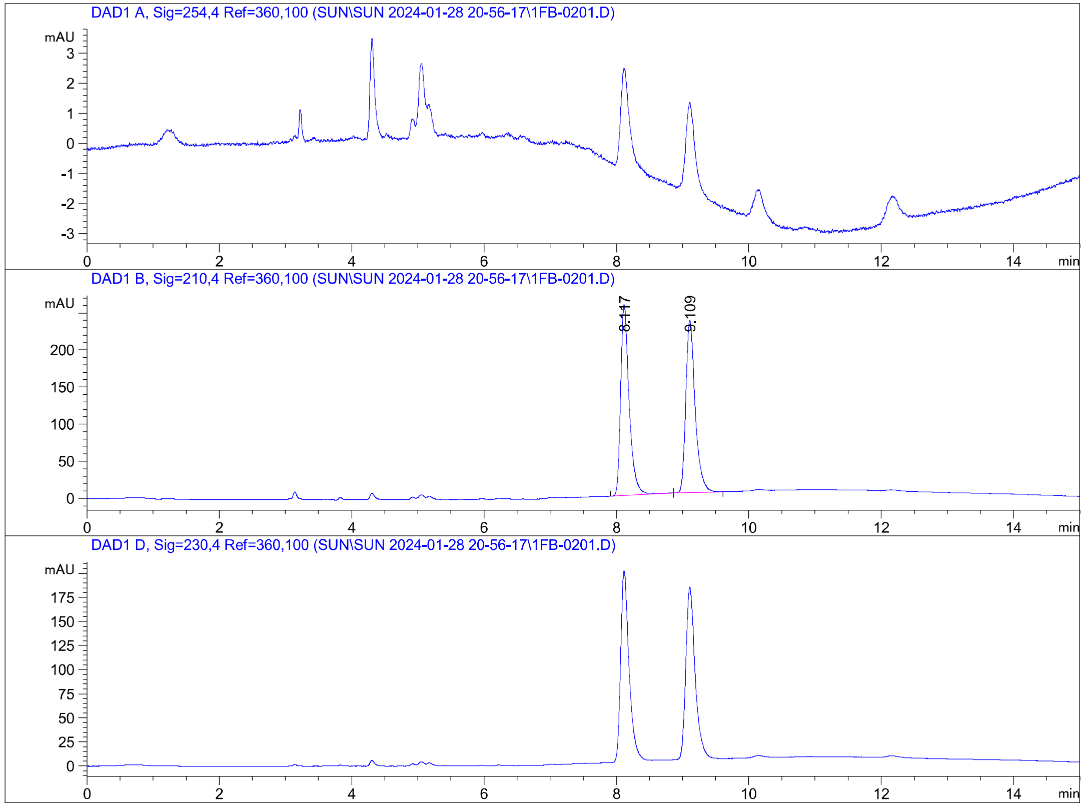

===

Area Percent Report

===

Sorted By : Signal
Multiplier : 1.0000
Dilution : 1.0000
Use Multiplier & Dilution Factor with ISTDs

Data File C:\Users\P...Documents\ChemStation\1\Data\SUN\SUN 2024-01-28 20-56-17\1FB-0201.D
Sample Name: yangll-9-21-rac-IB

Signal 1: DAD1 A, Sig=254,4 Ref=360,100

Signal 2: DAD1 B, Sig=210,4 Ref=360,100

Peak #	RetTime [min]	Type	Width [min]	Area [mAU*s]	Height [mAU]	Area %
1	8.117	BV R	0.1346	2301.49536	256.80316	50.2509
2	9.109	BV R	0.1491	2278.51587	232.01118	49.7491

Totals : 4580.01123 488.81435

Signal 3: DAD1 D, Sig=230,4 Ref=360,100

===

*** End of Report ***

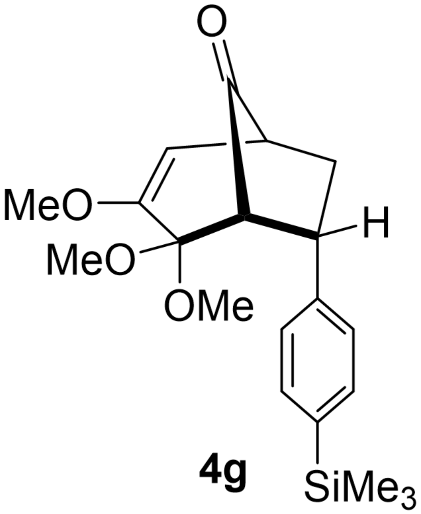

4g

rac

===

Acq. Operator : SYSTEM Seq. Line : 38
Sample Operator : SYSTEM
Acq. Instrument : HPLC Location : P1-A-05
Injection Date : 19/3/2024 1:00:23 pm Inj : 1
Inj Volume : 2.000 µl
Different Inj Volume from Sample Entry! Actual Inj Volume : 4.000 µl
Acq. Method : C:\Users\Public\Documents\ChemStation\1\Data\SUN\SUN 2024-03-18 22-29-36
\IBN3-10-20.M
Last changed : 30/7/2023 1:22:19 pm by SYSTEM
Analysis Method : C:\Users\Public\Documents\ChemStation\1\Data\SUN\SUN 2024-03-18 22-29-36
\IBN3-10-20.M (Sequence Method)
Last changed : 19/3/2024 8:24:17 pm by SYSTEM
(modified after loading)
Additional Info : Peak(s) manually integrated

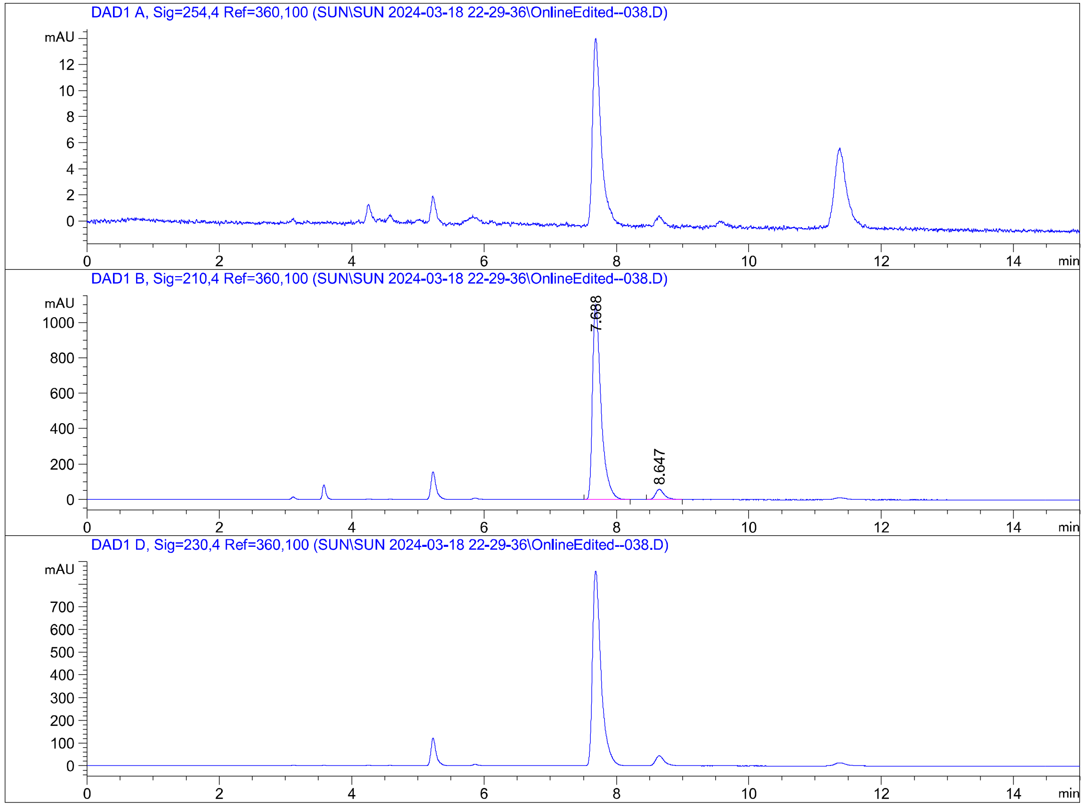

Data File C:\Users\P...\ChemStation\1\Data\SUN\SUN 2024-03-18 22-29-36\OnlineEdited--038.D
Sample Name: yangll-9-71-opt

===
Area Percent Report
===

Sorted By : Signal
Multiplier : 1.0000
Dilution : 1.0000
Use Multiplier & Dilution Factor with ISTDs

Signal 1: DAD1 A, Sig=254,4 Ref=360,100

Signal 2: DAD1 B, Sig=210,4 Ref=360,100

Peak #	RetTime [min]	Type	Width [min]	Area [mAU*s]	Height [mAU]	Area %
1	7.688	VV R	0.1322	9917.03906	1099.93994	94.8765
2	8.647	VV R	0.1211	535.53400	58.34932	5.1235

Totals : 1.04526e4 1158.28926

Signal 3: DAD1 D, Sig=230,4 Ref=360,100

===
*** End of Report ***

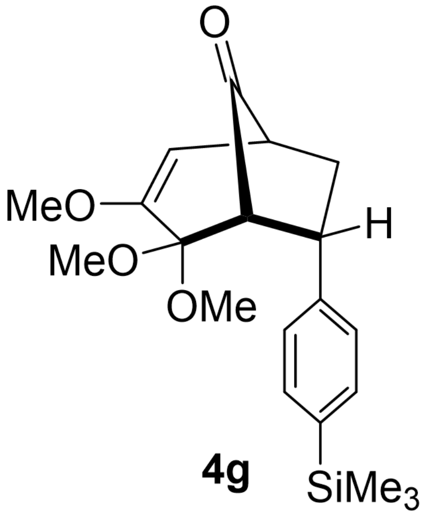

4g

opt

===

Acq. Operator : SYSTEM Seq. Line : 2
Sample Operator : SYSTEM
Acq. Instrument : HPLC Location : P1-A-04
Injection Date : 8/8/2023 11:46:03 pm Inj : 1
Inj Volume : 2.000 µl
Different Inj Volume from Sample Entry! Actual Inj Volume : 1.000 µl
Method : C:\Users\Public\Documents\ChemStation\1\Data\SUN\SUN 2023-08-08 23-34-03 \ID3-10-20.M (Sequence Method)
Last changed : 2/8/2023 12:18:49 pm by SYSTEM
Additional Info : Peak(s) manually integrated

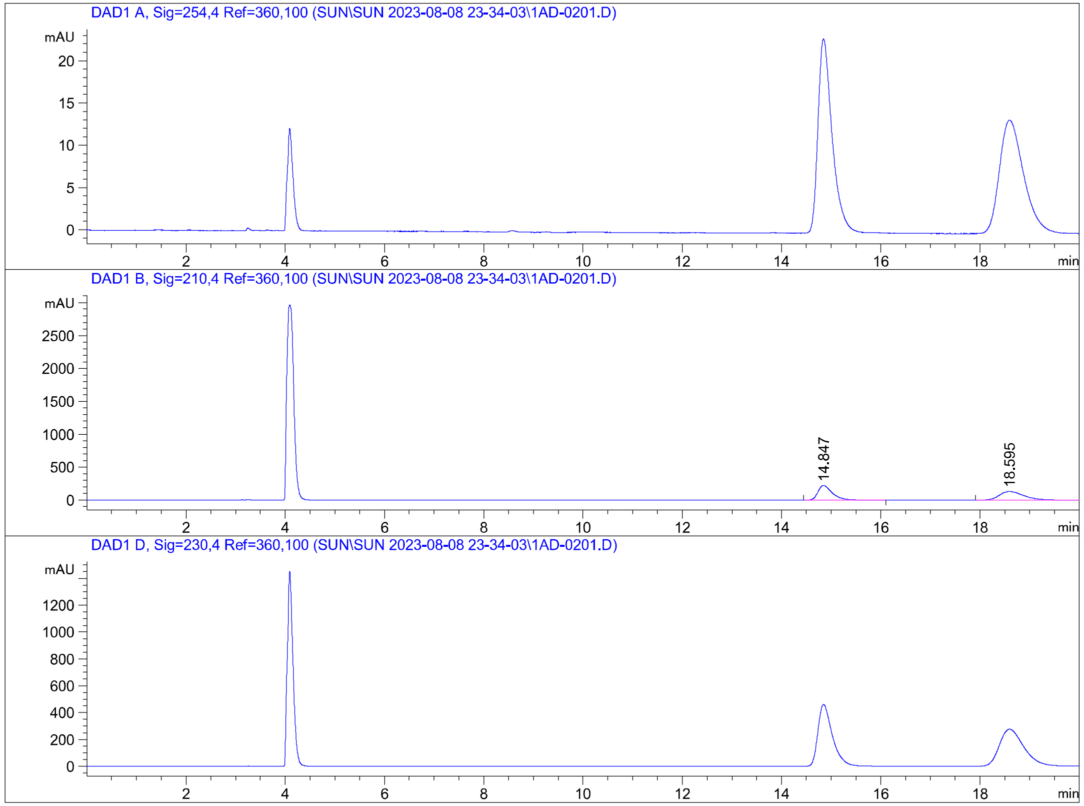

===

Area Percent Report

===

Sorted By : Signal
Multiplier : 1.0000
Dilution : 1.0000
Use Multiplier & Dilution Factor with ISTDs

Data File C:\Users\P...Documents\ChemStation\1\Data\SUN\SUN 2023-08-08 23-34-03\1AD-0201.D
Sample Name: yangll-7-102-rac-ID

Signal 1: DAD1 A, Sig=254,4 Ref=360,100

Signal 2: DAD1 B, Sig=210,4 Ref=360,100

Peak #	RetTime [min]	Type	Width [min]	Area [mAU*s]	Height [mAU]	Area %
1	14.847	BB	0.2959	4420.61572	222.35422	50.0542
2	18.595	BBA	0.4919	4411.03467	129.99049	49.9458

Totals : 8831.65039 352.34471

Signal 3: DAD1 D, Sig=230,4 Ref=360,100

===

*** End of Report ***

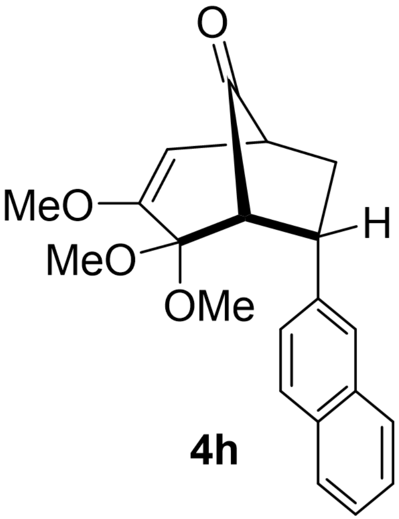

4h

rac

===

Acq. Operator : SYSTEM Seq. Line : 3
Sample Operator : SYSTEM
Acq. Instrument : HPLC Location : P1-A-02
Injection Date : 16/3/2024 10:05:18 am Inj : 1
Inj Volume : 2.000 µl
Different Inj Volume from Sample Entry! Actual Inj Volume : 20.000 µl
Acq. Method : C:\Users\Public\Documents\ChemStation\1\Data\SUN\SUN 2024-03-16 09-25-59 \ID3-10-30.M
Last changed : 16/8/2022 10:05:02 am by SYSTEM
Analysis Method : C:\Users\Public\Documents\ChemStation\1\Data\SUN\SUN 2024-03-16 09-25-59 \ID3-10-30.M (Sequence Method)
Last changed : 16/3/2024 3:13:20 pm by SYSTEM
(modified after loading)
Additional Info : Peak(s) manually integrated

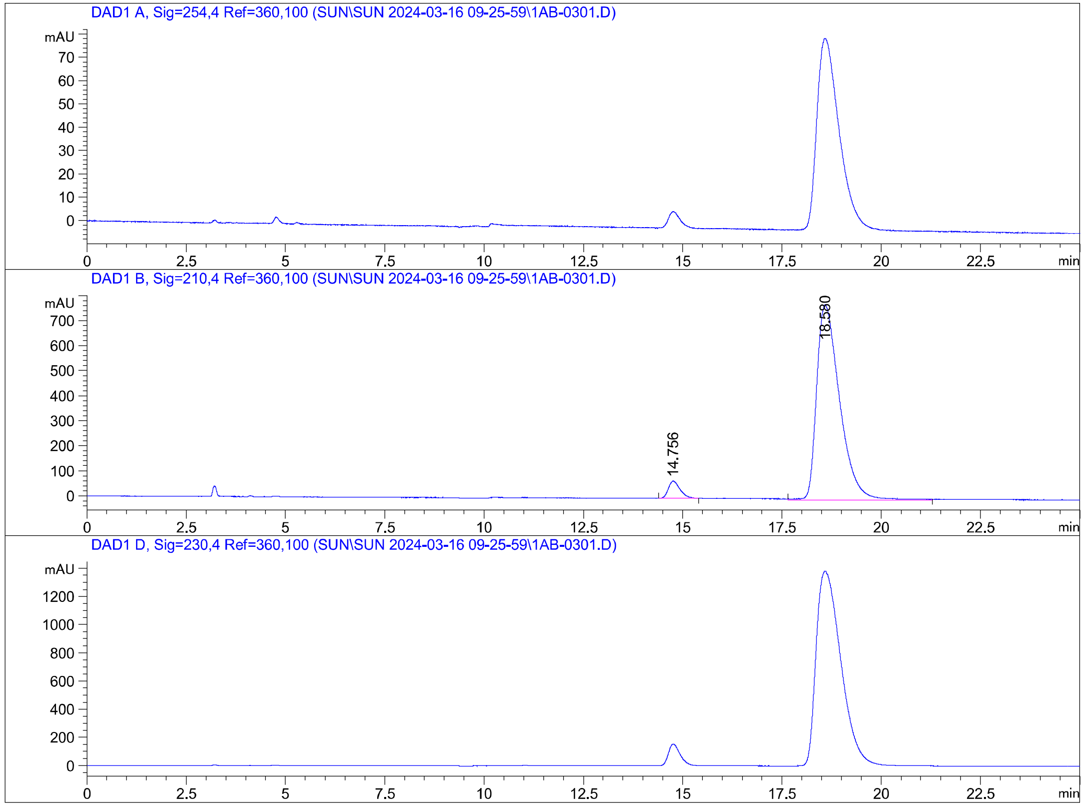

Data File C:\Users\P...Documents\ChemStation\1\Data\SUN\SUN 2024-03-16 09-25-59\1AB-0301.D
Sample Name: yangll-9-65-opt

===

Area Percent Report

===

Sorted By : Signal
Multiplier : 1.0000
Dilution : 1.0000
Use Multiplier & Dilution Factor with ISTDs

Signal 1: DAD1 A, Sig=254,4 Ref=360,100

Signal 2: DAD1 B, Sig=210,4 Ref=360,100

Peak #	RetTime [min]	Type	Width [min]	Area [mAU*s]	Height [mAU]	Area %
1	14.756	VV R	0.2442	1416.89587	69.11530	4.3116
2	18.580	MM R	0.6720	3.14453e4	779.86005	95.6884

Totals : 3.28622e4 848.97534

Signal 3: DAD1 D, Sig=230,4 Ref=360,100

===

*** End of Report ***

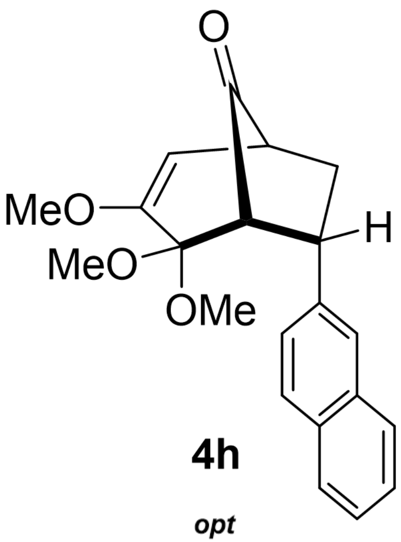

4h

opt

===

Acq. Operator : SYSTEM Seq. Line : 24
Sample Operator : SYSTEM
Acq. Instrument : HPLC Location : P1-A-01
Injection Date : 9/8/2023 6:27:39 am Inj : 1
Inj Volume : 2.000 µl
Different Inj Volume from Sample Entry! Actual Inj Volume : 1.000 µl
Method : C:\Users\Public\Documents\ChemStation\1\Data\SUN\SUN 2023-08-08 23-34-03 \OX3-10-20.M (Sequence Method)
Last changed : 15/8/2022 10:45:05 pm by SYSTEM
Additional Info : Peak(s) manually integrated

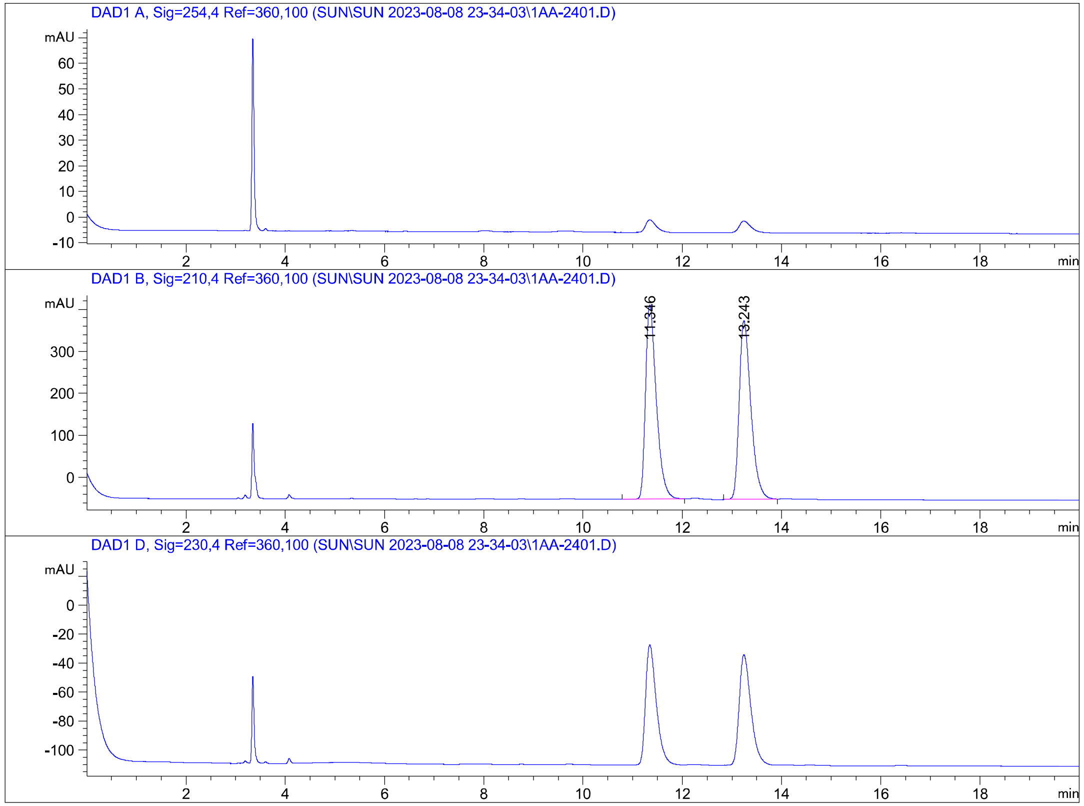

===
Area Percent Report
===

Sorted By : Signal
Multiplier : 1.0000
Dilution : 1.0000
Use Multiplier & Dilution Factor with ISTDs

Signal 1: DAD1 A, Sig=254,4 Ref=360,100

Signal 2: DAD1 B, Sig=210,4 Ref=360,100

Peak #	RetTime [min]	Type	Width [min]	Area [mAU*s]	Height [mAU]	Area %
1	11.346	BB	0.2305	6975.79541	462.34866	50.0470
2	13.243	BB	0.2511	6962.70557	425.03571	49.9530

Totals : 1.39385e4 887.38437

Signal 3: DAD1 D, Sig=230,4 Ref=360,100

===
*** End of Report ***

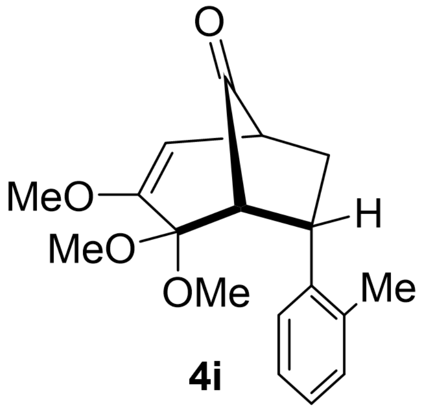

4i

rac

===

Acq. Operator : SYSTEM Seq. Line : 40
Sample Operator : SYSTEM
Acq. Instrument : HPLC Location : P1-A-07
Injection Date : 19/3/2024 1:32:51 pm Inj : 1
Inj Volume : 2.000 µl
Different Inj Volume from Sample Entry! Actual Inj Volume : 4.000 µl
Method : C:\Users\Public\Documents\ChemStation\1\Data\SUN\SUN 2024-03-18 22-29-36 \OX3-10-20.M (Sequence Method)
Last changed : 15/8/2022 10:45:05 pm by SYSTEM
Additional Info : Peak(s) manually integrated

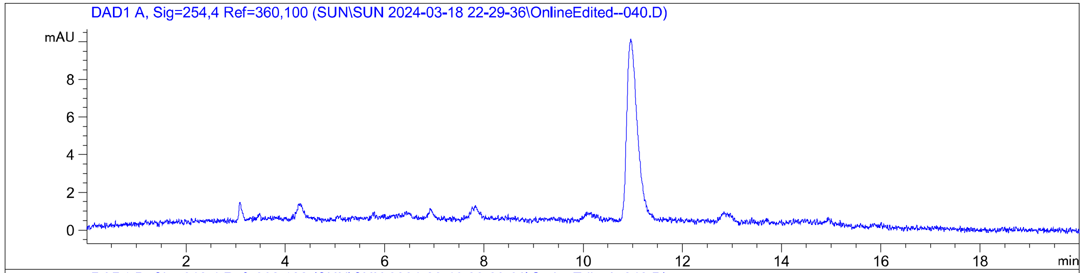

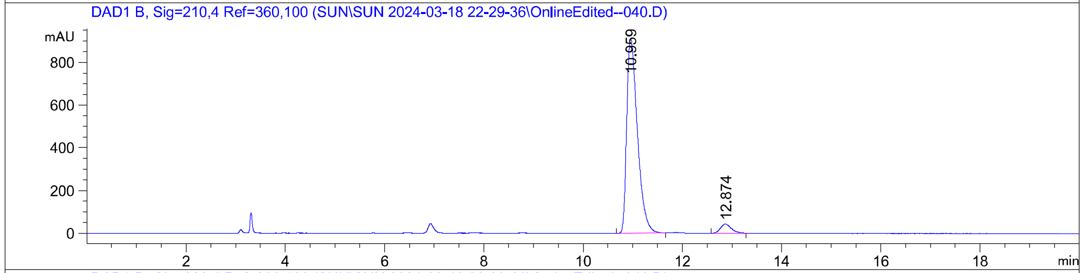

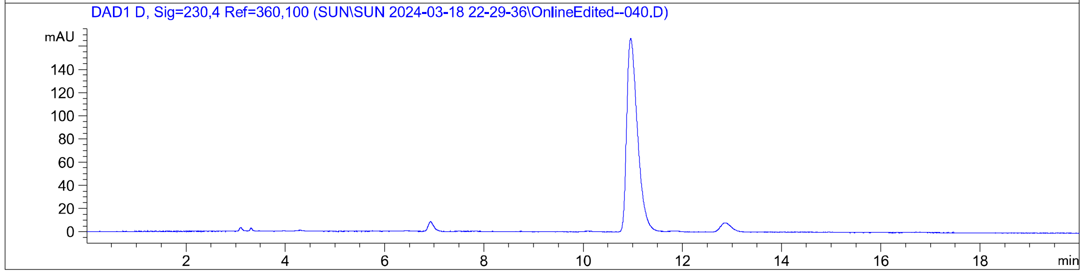

===

Area Percent Report

===

Sorted By : Signal
Multiplier : 1.0000
Dilution : 1.0000
Use Multiplier & Dilution Factor with ISTDs

Data File C:\Users\P...\ChemStation\1\Data\SUN\SUN 2024-03-18 22-29-36\OnlineEdited--040.D
Sample Name: yangll-9-73-opt

Signal 1: DAD1 A, Sig=254,4 Ref=360,100

Signal 2: DAD1 B, Sig=210,4 Ref=360,100

Peak #	RetTime [min]	Type	Width [min]	Area [mAU*s]	Height [mAU]	Area %
1	10.959	VV R	0.1794	1.36774e4	912.12543	95.4748
2	12.874	VV R	0.1824	648.26337	42.75622	4.5252

Totals : 1.43256e4 954.88165

Signal 3: DAD1 D, Sig=230,4 Ref=360,100

===

*** End of Report ***

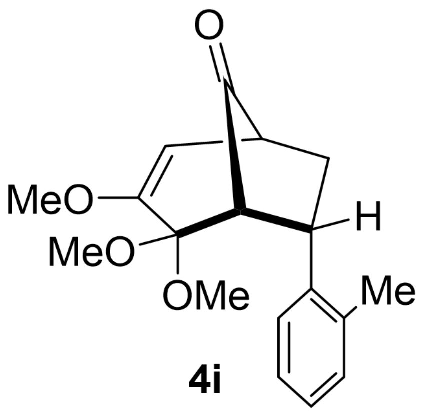

4i

opt

===

Acq. Operator	: SYSTEM	Seq. Line :	50
Sample Operator	: SYSTEM		
Acq. Instrument	: HPLC	Location :	P1-B-04
Injection Date	: 10/1/2024 5:51:56 am	Inj :	1
		Inj Volume :	2.000 µl

Method : C:\Users\Public\Documents\ChemStation\1\Data\SUN\SUN 2024-01-09 09-29-51\ID3-10-30.M (Sequence Method)
Last changed : 16/8/2022 10:05:02 am by SYSTEM
Additional Info : Peak(s) manually integrated

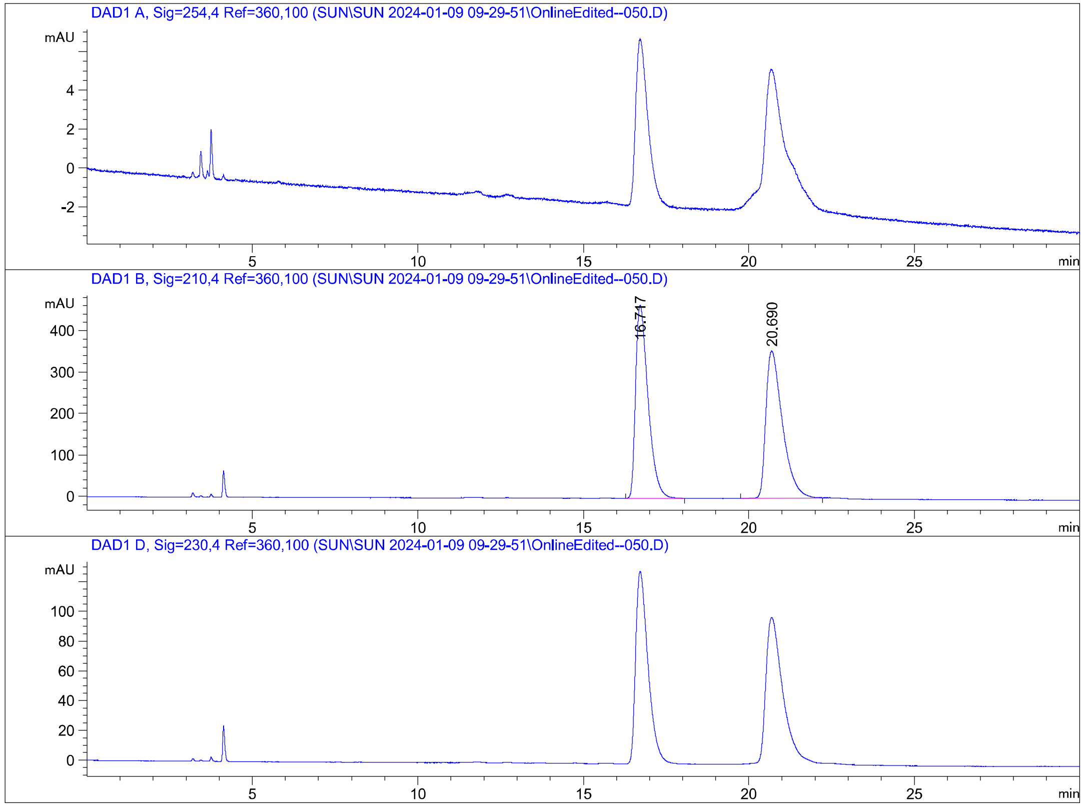

===
Area Percent Report
===

Sorted By : Signal
Multiplier : 1.0000
Dilution : 1.0000
Use Multiplier & Dilution Factor with ISTDs

Signal 1: DAD1 A, Sig=254,4 Ref=360,100

Signal 2: DAD1 B, Sig=210,4 Ref=360,100

Peak #	RetTime [min]	Type	Width [min]	Area [mAU*s]	Height [mAU]	Area %
1	16.717	BB	0.3786	1.22149e4	466.34247	49.6819
2	20.690	VV R	0.4681	1.23714e4	355.37238	50.3181

Totals : 2.45863e4 821.71484

Signal 3: DAD1 D, Sig=230,4 Ref=360,100

===
*** End of Report ***

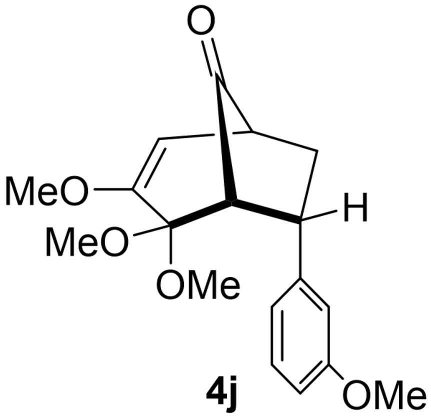

4j

rac

===

```
Acq. Operator    : SYSTEM                        Seq. Line :   35
Sample Operator  : SYSTEM
Acq. Instrument  : HPLC                           Location :   P1-A-06
Injection Date   : 19/3/2024 11:56:38 am               Inj :    1
                                              Inj Volume : 2.000 µl
Different Inj Volume from Sample Entry!  Actual Inj Volume : 4.000 µl
Method           : C:\Users\Public\Documents\ChemStation\1\Data\SUN\SUN 2024-03-18 22-29-36
                  \ID3-10-30.M (Sequence Method)
Last changed     : 16/8/2022 10:05:02 am by SYSTEM
Additional Info  : Peak(s) manually integrated
```

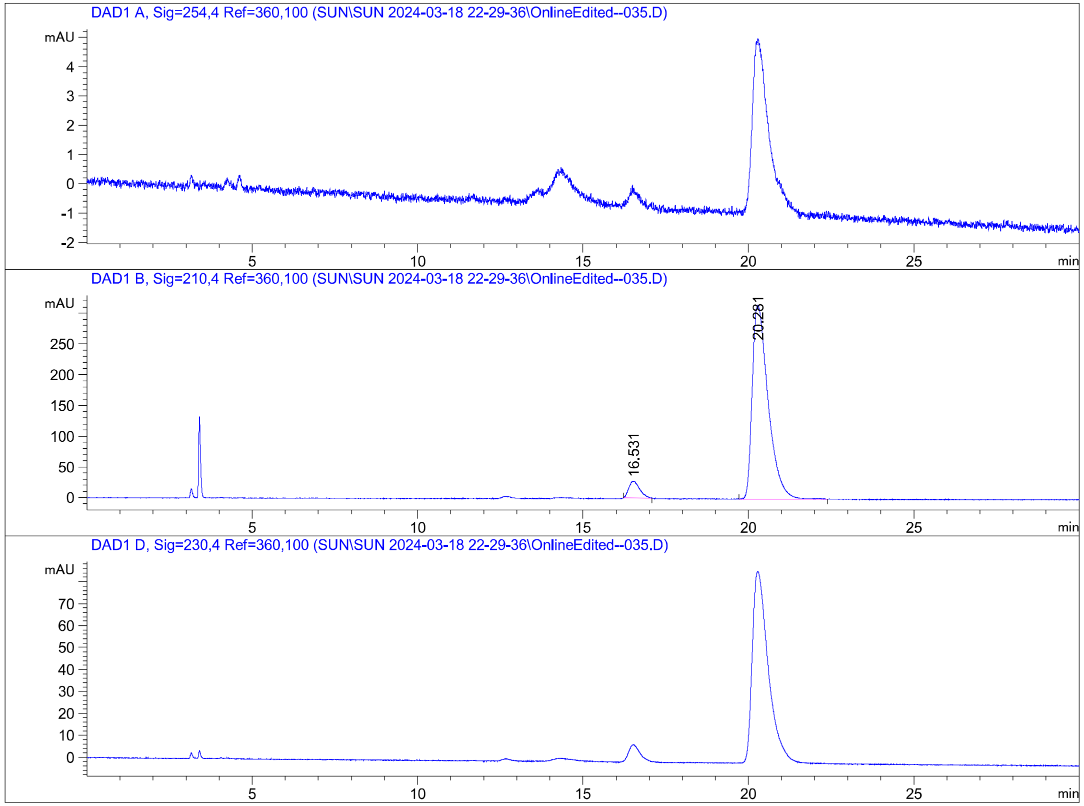

===

Area Percent Report

===

```
Sorted By             :      Signal
Multiplier            :      1.0000
Dilution              :      1.0000
Use Multiplier & Dilution Factor with ISTDs
```


Data File C:\Users\P...\ChemStation\1\Data\SUN\SUN 2024-03-18 22-29-36\OnlineEdited--035.D
Sample Name: yangll-9-72-opt

Signal 1: DAD1 A, Sig=254,4 Ref=360,100

Signal 2: DAD1 B, Sig=210,4 Ref=360,100

Peak #	RetTime [min]	Type	Width [min]	Area [mAU*s]	Height [mAU]	Area %
1	16.531	MM R	0.3773	627.14471	27.70139	5.6041
2	20.281	MM R	0.5570	1.05637e4	316.07806	94.3959

Totals : 1.11908e4 343.77945

Signal 3: DAD1 D, Sig=230,4 Ref=360,100

===

*** End of Report ***

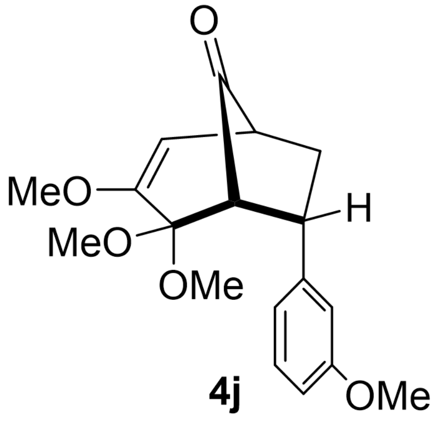

4j

opt

===

```
Acq. Operator   : SYSTEM                    Seq. Line :  29
Sample Operator : SYSTEM
Acq. Instrument : HPLC                       Location :  P1-E-02
Injection Date  : 23/1/2024 10:38:29 am           Inj :  1
                                          Inj Volume : 2.000 µl
Acq. Method     : C:\Users\Public\Documents\ChemStation\1\Data\SUN\SUN 2024-01-22 20-33-33
                  \AD3-10-30.M
Last changed    : 23/1/2024 10:56:34 am by SYSTEM
                  (modified after loading)
Analysis Method : C:\Users\Public\Documents\ChemStation\1\Data\SUN\SUN 2024-01-22 20-33-33
                  \AD3-10-30.M (Sequence Method)
Last changed    : 27/3/2024 6:21:21 pm by SYSTEM
Additional Info : Peak(s) manually integrated
```

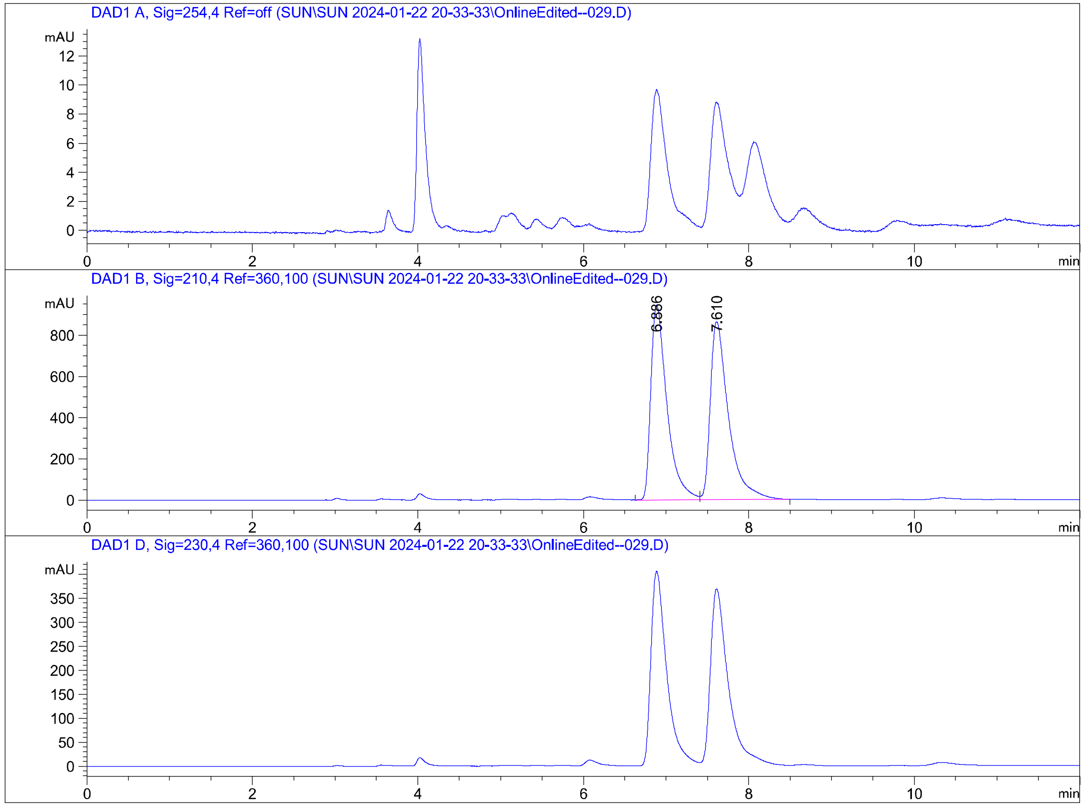

===

Area Percent Report

===

```
Sorted By             :      Signal
Multiplier            :      1.0000
Dilution              :      1.0000
Use Multiplier & Dilution Factor with ISTDs
```


Data File C:\Users\P...\ChemStation\1\Data\SUN\SUN 2024-01-22 20-33-33\OnlineEdited--029.D
Sample Name: yangll-9-7-2-rac-AD

Signal 1: DAD1 A, Sig=254,4 Ref=off

Signal 2: DAD1 B, Sig=210,4 Ref=360,100

Peak #	RetTime [min]	Type	Width [min]	Area [mAU*s]	Height [mAU]	Area %
1	6.886	VV R	0.2016	1.28475e4	945.29120	49.3496
2	7.610	VB	0.2255	1.31861e4	864.06195	50.6504

Totals : 2.60336e4 1809.35315

Signal 3: DAD1 D, Sig=230,4 Ref=360,100

===

*** End of Report ***

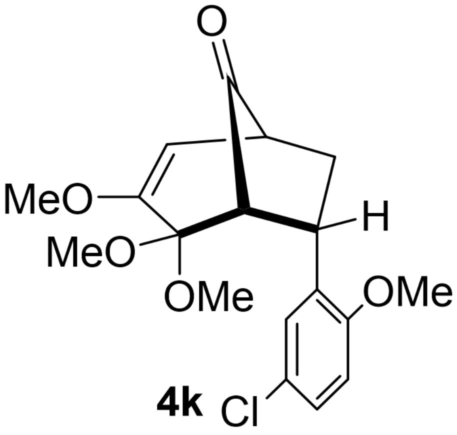

4k

rac

===

Acq. Operator : SYSTEM Seq. Line : 30
Sample Operator : SYSTEM
Acq. Instrument : HPLC Location : P1-D-02
Injection Date : 23/1/2024 10:57:28 am Inj : 1
Inj Volume : 2.000 µl
Acq. Method : C:\Users\Public\Documents\ChemStation\1\Data\SUN\SUN 2024-01-22 20-33-33\AD3-10-30.M
Last changed : 23/1/2024 10:56:34 am by SYSTEM
Analysis Method : C:\Users\Public\Documents\ChemStation\1\Data\SUN\SUN 2024-01-22 20-33-33\AD3-10-30.M (Sequence Method)
Last changed : 27/3/2024 6:21:21 pm by SYSTEM
Additional Info : Peak(s) manually integrated

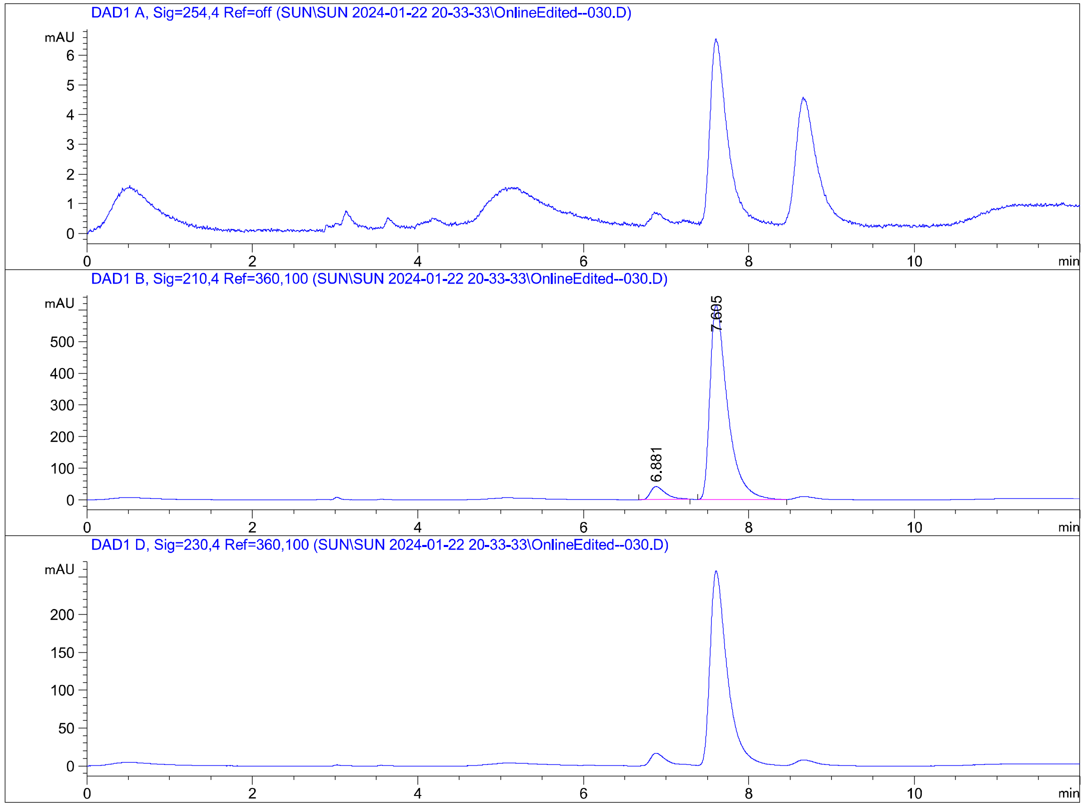

===

Area Percent Report

===

Sorted By : Signal
Multiplier : 1.0000
Dilution : 1.0000
Use Multiplier & Dilution Factor with ISTDs

Data File C:\Users\P...\ChemStation\1\Data\SUN\SUN 2024-01-22 20-33-33\OnlineEdited--030.D
Sample Name: yangll-9-7-opt

Signal 1: DAD1 A, Sig=254,4 Ref=off

Signal 2: DAD1 B, Sig=210,4 Ref=360,100

Peak #	RetTime [min]	Type	Width [min]	Area [mAU*s]	Height [mAU]	Area %
1	6.881	MM R	0.2104	515.50055	40.84082	5.3660
2	7.605	VV	0.2143	9091.20508	615.55621	94.6340

Totals : 9606.70563 656.39703

Signal 3: DAD1 D, Sig=230,4 Ref=360,100

===

*** End of Report ***

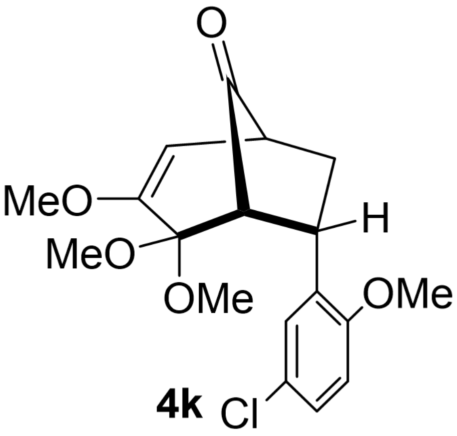

4k

opt

Data File C:\Users\P...\1\Data\SUN\SUN 2024-03-07 14-14-51\002-P1-B1-yangll-9-48-rac2-AD.D
Sample Name: yangll-9-48-rac2-AD

===

Acq. Operator : SYSTEM Seq. Line : 2
Acq. Instrument : LC1260 Location : P1-B-01
Injection Date : 3/7/2024 2:27:36 PM Inj : 1
Inj Volume : 5.000 µl
Different Inj Volume from Sample Entry! Actual Inj Volume : 4.000 µl
Acq. Method : C:\Users\Public\Documents\ChemStation\1\Data\SUN\SUN 2024-03-07 14-14-51\AD3-10-20.M
Last changed : 3/7/2024 1:13:10 PM by SYSTEM
Analysis Method : C:\Users\Public\Documents\ChemStation\1\Data\SUN\SUN 2024-03-07 14-14-51\AD3-10-20.M (Sequence Method)
Last changed : 3/27/2024 6:31:37 PM by SYSTEM
(modified after loading)
Additional Info : Peak(s) manually integrated

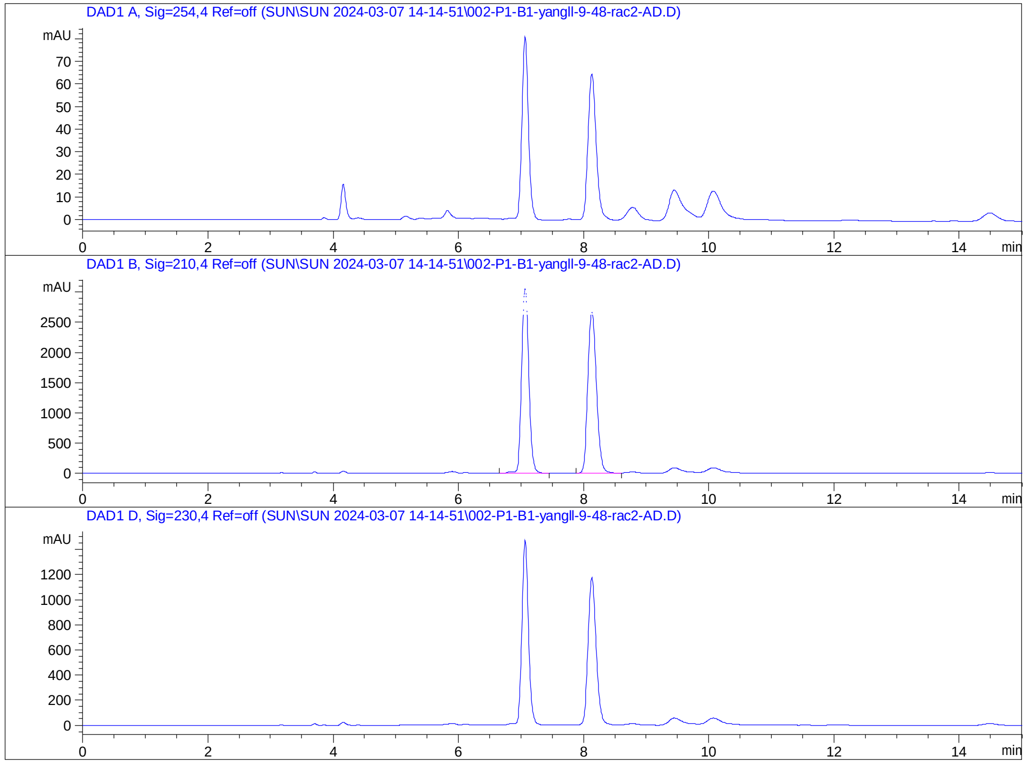

===

Area Percent Report

===

Sorted By : Signal
Multiplier : 1.0000
Dilution : 1.0000
Use Multiplier & Dilution Factor with ISTDs

Data File C:\Users\P...\1\Data\SUN\SUN 2024-03-07 14-14-51\002-P1-B1-yangll-9-48-rac2-AD.D
Sample Name: yangll-9-48-rac2-AD

Signal 1: DAD1 A, Sig=254,4 Ref=off

Signal 2: DAD1 B, Sig=210,4 Ref=off

Peak #	RetTime [min]	Type	Width [min]	Area [mAU*s]	Height [mAU]	Area %
1	7.065	BB R	0.1237	2.41192e4	3052.07471	48.8434
2	8.131	MM R	0.1581	2.52615e4	2662.61792	51.1566

Totals : 4.93806e4 5714.69263

Signal 3: DAD1 D, Sig=230,4 Ref=off

===

*** End of Report ***

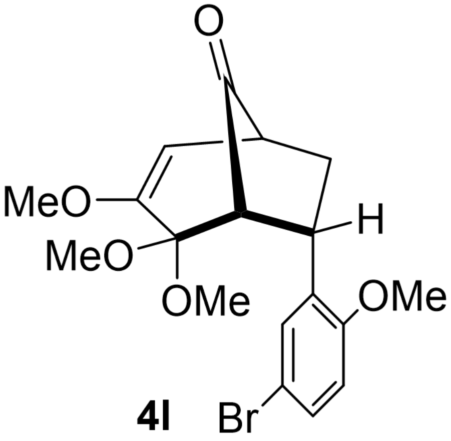

4l

rac

Data File C:\Users\P...1\Data\SUN\SUN 2024-03-07 14-14-51\008-P1-D1-yangll-9-48-2-opt-AD.D
Sample Name: yangll-9-48-2-opt-AD

===

Acq. Operator : SYSTEM Seq. Line : 8
Acq. Instrument : LC1260 Location : P1-D-01
Injection Date : 3/7/2024 3:56:29 PM Inj : 1
Inj Volume : 5.000 µl
Different Inj Volume from Sample Entry! Actual Inj Volume : 2.000 µl
Acq. Method : C:\Users\Public\Documents\ChemStation\1\Data\SUN\SUN 2024-03-07 14-14-51\AD3-10-20.M
Last changed : 3/7/2024 4:09:35 PM by SYSTEM (modified after loading)
Analysis Method : C:\Users\Public\Documents\ChemStation\1\Data\SUN\SUN 2024-03-07 14-14-51\AD3-10-20.M (Sequence Method)
Last changed : 3/27/2024 6:31:37 PM by SYSTEM (modified after loading)
Additional Info : Peak(s) manually integrated

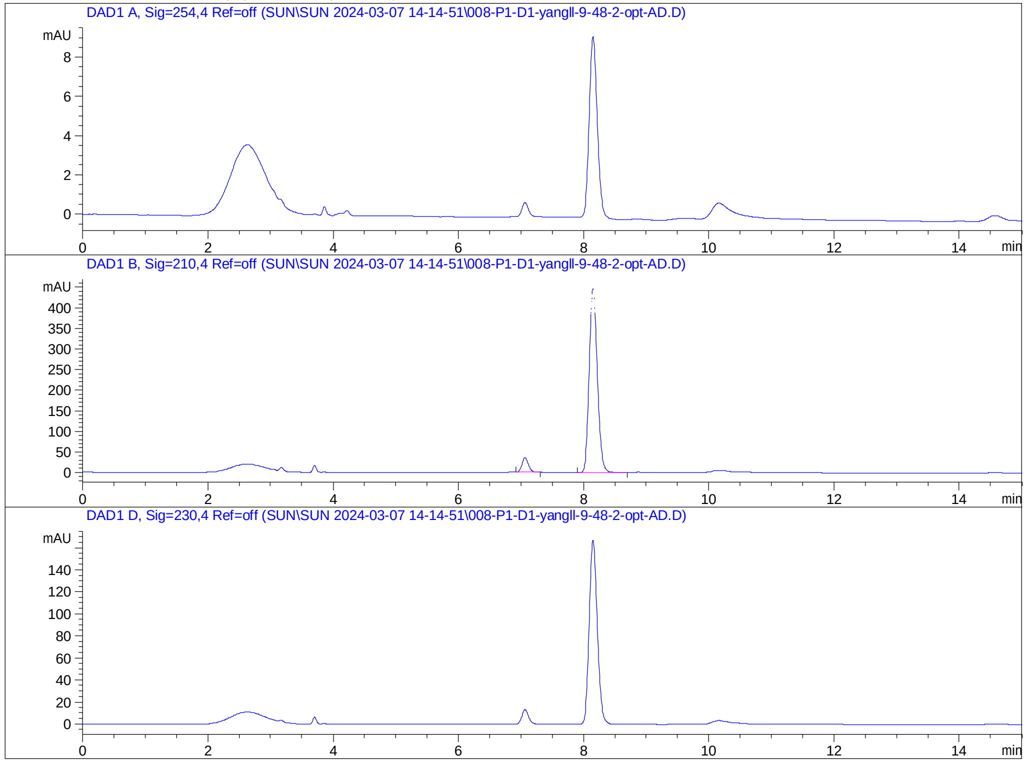

Data File C:\Users\P...1\Data\SUN\SUN 2024-03-07 14-14-51\008-P1-D1-yangll-9-48-2-opt-AD.D
Sample Name: yangll-9-48-2-opt-AD

===
Area Percent Report
===

Sorted By : Signal
Multiplier : 1.0000
Dilution : 1.0000
Use Multiplier & Dilution Factor with ISTDs

Signal 1: DAD1 A, Sig=254,4 Ref=off

Signal 2: DAD1 B, Sig=210,4 Ref=off

Peak #	RetTime [min]	Type	Width [min]	Area [mAU*s]	Height [mAU]	Area %
1	7.066	MM R	0.1182	254.21092	35.85629	5.9622
2	8.151	BB	0.1384	4009.47241	448.00226	94.0378

Totals : 4263.68333 483.85854

Signal 3: DAD1 D, Sig=230,4 Ref=off

===
*** End of Report ***

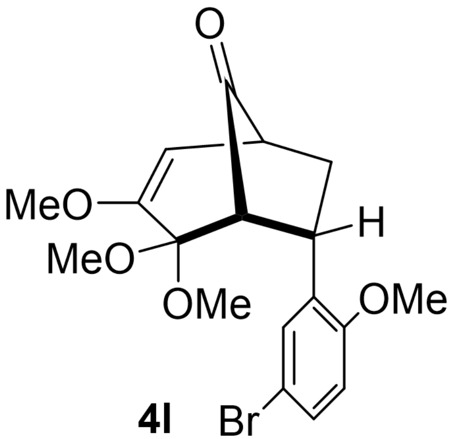

4l

opt

===

Acq. Operator : SYSTEM Seq. Line : 3
Acq. Instrument : LC1260 Location : P1-B-02
Injection Date : 3/7/2024 2:48:36 PM Inj : 1
Inj Volume : 5.000 µl
Different Inj Volume from Sample Entry! Actual Inj Volume : 4.000 µl
Acq. Method : C:\Users\Public\Documents\ChemStation\1\Data\SUN\SUN 2024-03-07 14-14-51\AD3-10-20.M
Last changed : 3/7/2024 1:13:10 PM by SYSTEM
Analysis Method : C:\Users\Public\Documents\ChemStation\1\Data\SUN\SUN 2024-03-07 14-14-51\AD3-10-20.M (Sequence Method)
Last changed : 3/27/2024 6:31:37 PM by SYSTEM
(modified after loading)
Additional Info : Peak(s) manually integrated

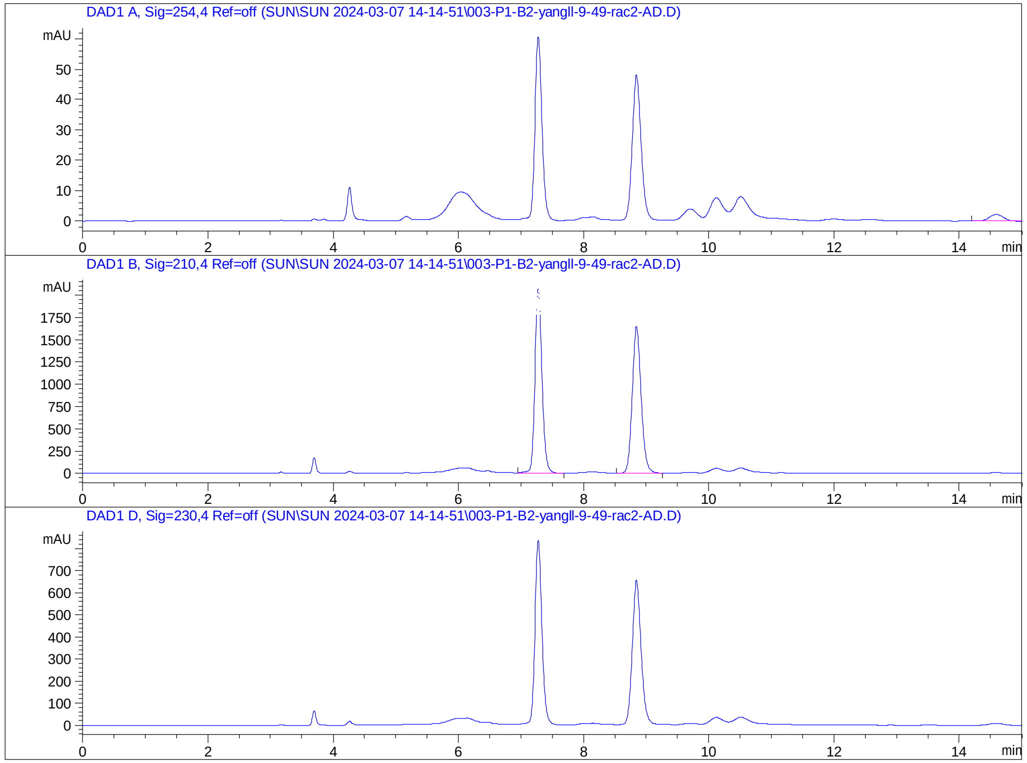

===

Area Percent Report

===

Sorted By : Signal
Multiplier : 1.0000
Dilution : 1.0000
Use Multiplier & Dilution Factor with ISTDs

Data File C:\Users\P...\1\Data\SUN\SUN 2024-03-07 14-14-51\003-P1-B2-yangll-9-49-rac2-AD.D
Sample Name: yangll-9-49-rac2-AD

Signal 1: DAD1 A, Sig=254,4 Ref=off

Peak #	RetTime [min]	Type	Width [min]	Area [mAU*s]	Height [mAU]	Area %
1	14.594	BB	0.2488	35.56068	2.22106	100.0000
Totals :				35.56068	2.22106	

Signal 2: DAD1 B, Sig=210,4 Ref=off

Peak #	RetTime [min]	Type	Width [min]	Area [mAU*s]	Height [mAU]	Area %
1	7.281	MM R	0.1317	1.63406e4	2067.57593	49.9963
2	8.850	MM R	0.1657	1.63430e4	1643.98279	50.0037
Totals :				3.26835e4	3711.55872	

Signal 3: DAD1 D, Sig=230,4 Ref=off

===

*** End of Report ***

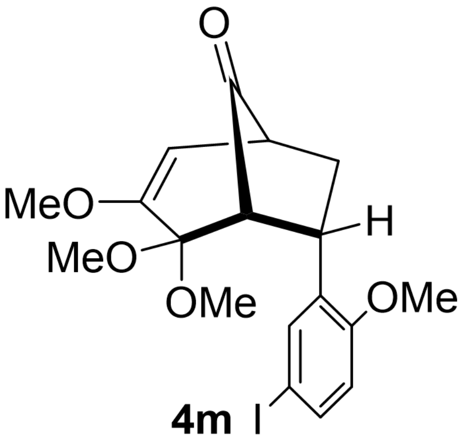

4m

rac

===

Acq. Operator : SYSTEM Seq. Line : 9
Acq. Instrument : LC1260 Location : P1-D-02
Injection Date : 3/7/2024 4:12:26 PM Inj : 1
Inj Volume : 5.000 µl
Different Inj Volume from Sample Entry! Actual Inj Volume : 2.000 µl
Acq. Method : C:\Users\Public\Documents\ChemStation\1\Data\SUN\SUN 2024-03-07 14-14-51\AD3-10-20.M
Last changed : 3/7/2024 4:09:35 PM by SYSTEM
Analysis Method : C:\Users\Public\Documents\ChemStation\1\Data\SUN\SUN 2024-03-07 14-14-51\AD3-10-20.M (Sequence Method)
Last changed : 3/27/2024 6:31:37 PM by SYSTEM
(modified after loading)
Additional Info : Peak(s) manually integrated

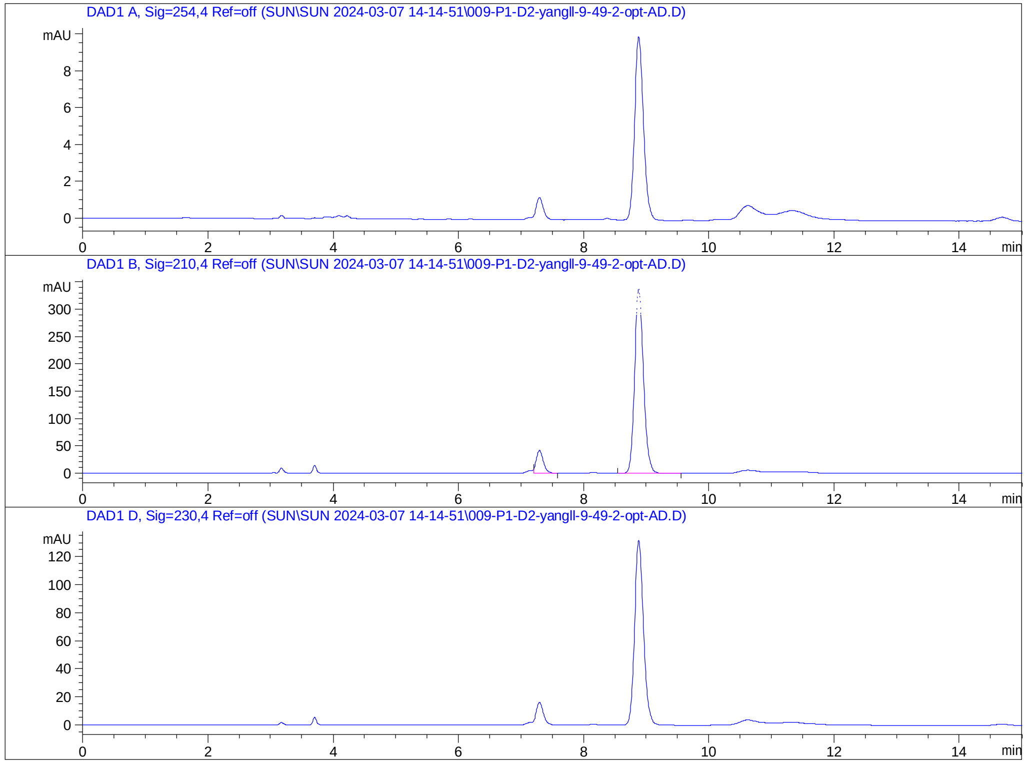

===

Area Percent Report

===

Sorted By : Signal
Multiplier : 1.0000
Dilution : 1.0000
Use Multiplier & Dilution Factor with ISTDs

Data File C:\Users\P...1\Data\SUN\SUN 2024-03-07 14-14-51\009-P1-D2-yangll-9-49-2-opt-AD.D
Sample Name: yangll-9-49-2-opt-AD

Signal 1: DAD1 A, Sig=254,4 Ref=off

Signal 2: DAD1 B, Sig=210,4 Ref=off

Peak #	RetTime [min]	Type	Width [min]	Area [mAU*s]	Height [mAU]	Area %
1	7.297	MM R	0.1300	325.85385	41.76998	8.8534
2	8.885	BB	0.1537	3354.68677	338.39551	91.1466

Totals : 3680.54062 380.16549

Signal 3: DAD1 D, Sig=230,4 Ref=off

===
*** End of Report ***

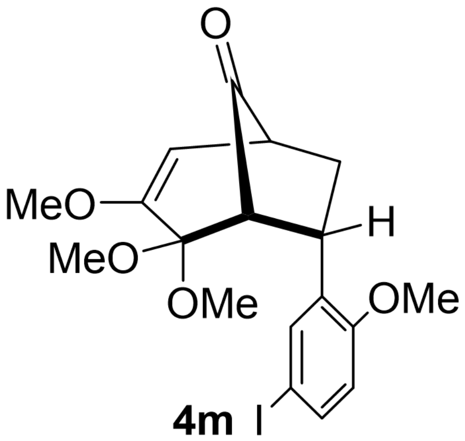

4m

opt

Data File C:\Users\P...\1\Data\SUN\SUN 2024-07-29 15-47-34\002-P1-F1-yangll-10-33-rac-IB.D
Sample Name: yangll-10-33-rac-IB

===

Acq. Operator : SYSTEM Seq. Line : 2
Acq. Instrument : LC1260 Location : P1-F-01
Injection Date : 7/29/2024 4:00:27 PM Inj : 1
Inj Volume : 5.000 µl
Different Inj Volume from Sample Entry! Actual Inj Volume : 10.000 µl
Acq. Method : C:\Users\Public\Documents\ChemStation\1\Data\SUN\SUN 2024-07-29 15-47-34 \IBN3-30-30.M
Last changed : 7/29/2024 4:13:26 PM by SYSTEM (modified after loading)
Analysis Method : C:\Users\Public\Documents\ChemStation\1\Data\SUN\SUN 2024-07-29 15-47-34 \IBN3-30-30.M (Sequence Method)
Last changed : 7/29/2024 4:50:32 PM by SYSTEM
Additional Info : Peak(s) manually integrated

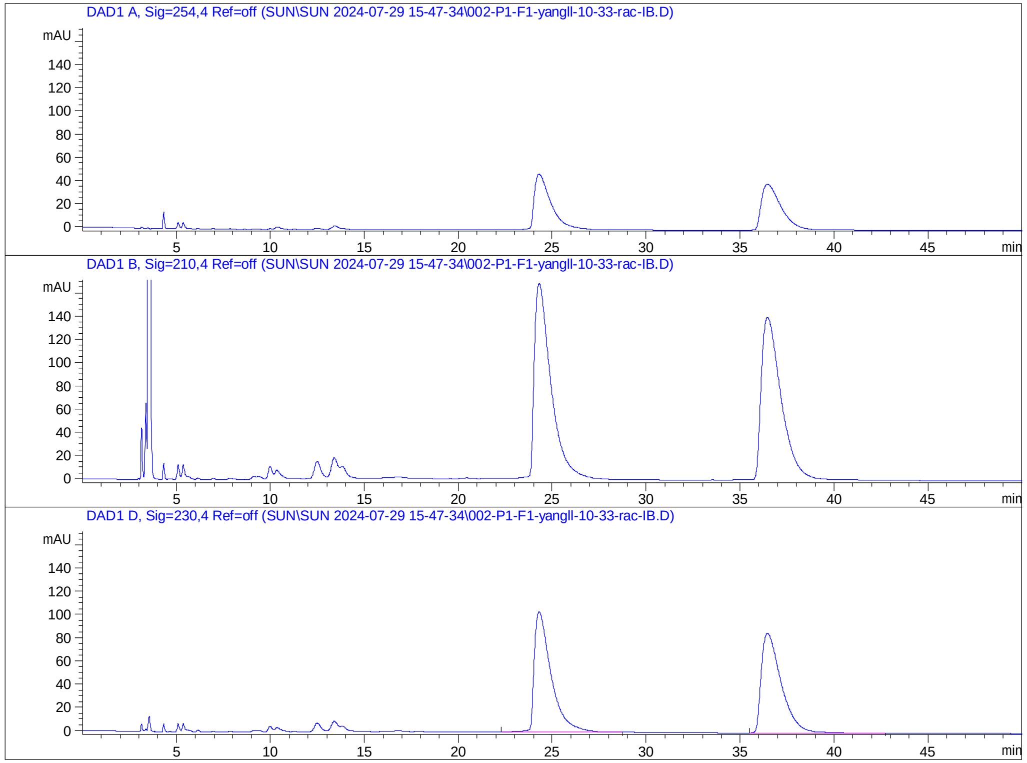

===

Area Percent Report

===

Sorted By : Signal
Multiplier : 1.0000
Dilution : 1.0000
Use Multiplier & Dilution Factor with ISTDs

Data File C:\Users\P...\1\Data\SUN\SUN 2024-07-29 15-47-34\002-P1-F1-yangll-10-33-rac-IB.D
Sample Name: yangll-10-33-rac-IB

Signal 1: DAD1 A, Sig=254,4 Ref=off

Signal 2: DAD1 B, Sig=210,4 Ref=off

Signal 3: DAD1 D, Sig=230,4 Ref=off

Peak #	RetTime [min]	Type	Width [min]	Area [mAU*s]	Height [mAU]	Area %
1	24.306	BB	0.9334	6481.06641	103.37818	50.7883
2	36.463	BB	1.1270	6279.87646	85.49470	49.2117

Totals : 1.27609e4 188.87288

===

*** End of Report ***

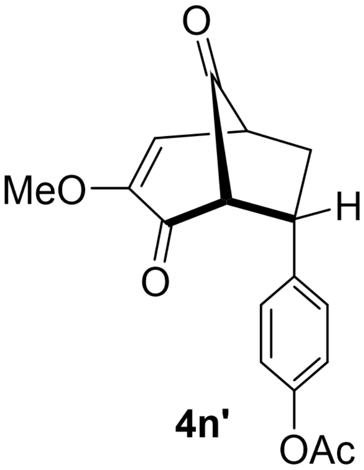

4n'

rac

===

Acq. Operator : SYSTEM Seq. Line : 6
Acq. Instrument : LC1260 Location : P1-F-04
Injection Date : 7/28/2024 8:15:47 PM Inj : 1
Inj Volume : 5.000 µl
Acq. Method : C:\Users\Public\Documents\ChemStation\1\Data\SUN\SUN 2024-07-28 17-08-50 \IBN3-30-30.M
Last changed : 7/28/2024 6:04:19 PM by SYSTEM
Analysis Method : C:\Users\Public\Documents\ChemStation\1\Data\SUN\SUN 2024-07-28 17-08-50 \IBN3-30-30.M (Sequence Method)
Last changed : 7/29/2024 4:38:01 PM by SYSTEM
(modified after loading)
Additional Info : Peak(s) manually integrated

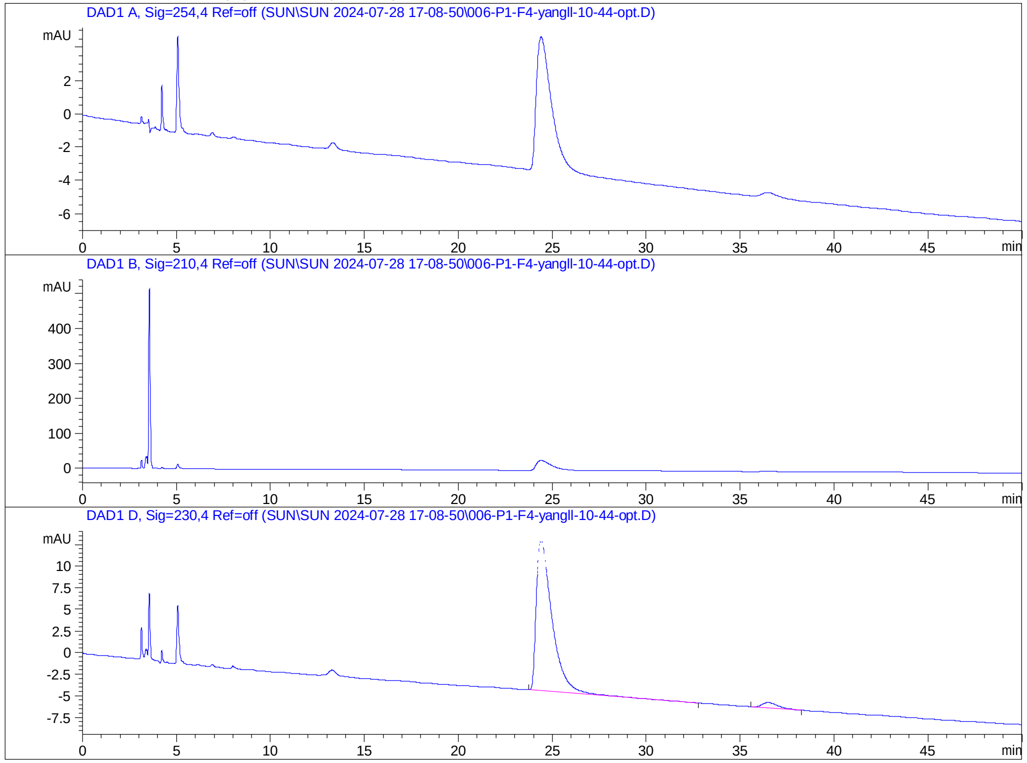

===
Area Percent Report
===

Sorted By : Signal
Multiplier : 1.0000
Dilution : 1.0000
Use Multiplier & Dilution Factor with ISTDs

Data File C:\Users\P...ion\1\Data\SUN\SUN 2024-07-28 17-08-50\006-P1-F4-yangll-10-44-opt.D
Sample Name: yangll-10-44-opt

Signal 1: DAD1 A, Sig=254,4 Ref=off

Signal 2: DAD1 B, Sig=210,4 Ref=off

Signal 3: DAD1 D, Sig=230,4 Ref=off

Peak #	RetTime [min]	Type	Width [min]	Area [mAU*s]	Height [mAU]	Area %
1	24.410	BB	0.8718	1019.56311	17.39616	96.2658
2	36.522	MM R	1.0136	39.54974	6.50315e-1	3.7342

Totals : 1059.11285 18.04648

===
*** End of Report ***

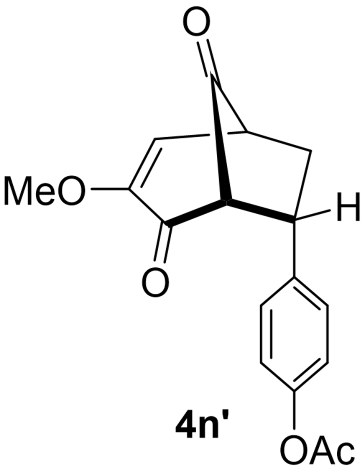

4n'

opt

===

```
Acq. Operator   : SYSTEM                          Seq. Line :    4
Acq. Instrument : LC1260                           Location :   P1-A-01
Injection Date  : 12/2/2024 10:18:39 PM                  Inj :    1
                                                Inj Volume : 5.000 µl
Different Inj Volume from Sample Entry!  Actual Inj Volume : 2.000 µl
Acq. Method     : C:\Users\Public\Documents\ChemStation\1\Data\SUN\SUN 2024-12-02 21-37-50
                  \IBN3-10-20.M
Last changed    : 5/1/2024 8:18:08 PM by SYSTEM
Analysis Method : C:\Users\Public\Documents\ChemStation\1\Data\SUN\SUN 2024-12-02 21-37-50
                  \IBN3-10-20.M (Sequence Method)
Last changed    : 12/3/2024 9:32:14 AM by SYSTEM
                  (modified after loading)
Additional Info : Peak(s) manually integrated
```

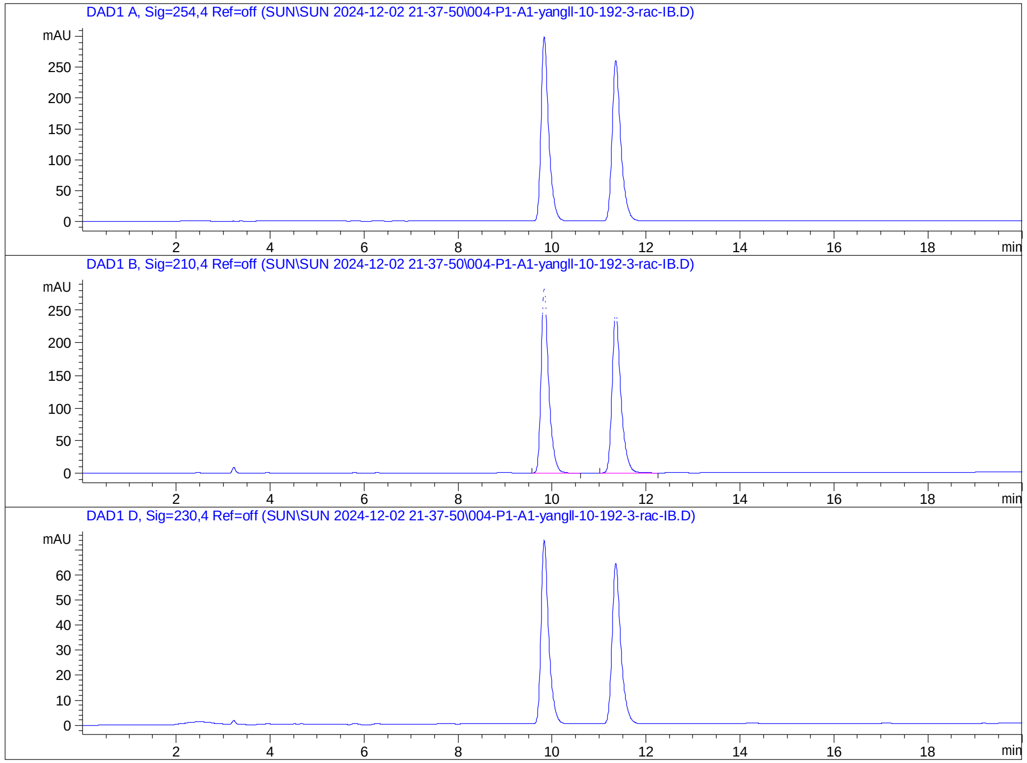

===

Area Percent Report

===

```
Sorted By             :      Signal
Multiplier            :      1.0000
Dilution              :      1.0000
Use Multiplier & Dilution Factor with ISTDs
```


Data File C:\Users\P...Data\SUN\SUN 2024-12-02 21-37-50\004-P1-A1-yangll-10-192-3-rac-IB.D
Sample Name: yangll-10-192-3-rac-IB

Signal 1: DAD1 A, Sig=254,4 Ref=off

Signal 2: DAD1 B, Sig=210,4 Ref=off

Peak #	RetTime [min]	Type	Width [min]	Area [mAU*s]	Height [mAU]	Area %
1	9.833	BB	0.1705	3203.96289	282.19235	49.9971
2	11.357	BB	0.1959	3204.33667	246.03540	50.0029

Totals : 6408.29956 528.22775

Signal 3: DAD1 D, Sig=230,4 Ref=off

===

*** End of Report ***

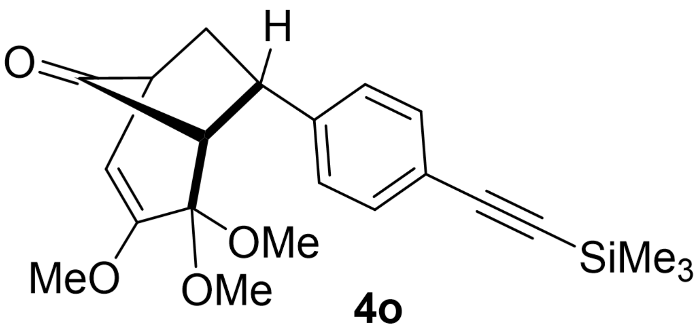

4o

rac

===

Acq. Operator : SYSTEM   Seq. Line : 5
Acq. Instrument : LC1260   Location : P1-A-02
Injection Date : 12/2/2024 10:39:35 PM   Inj : 1
Inj Volume : 5.000 µl
Different Inj Volume from Sample Entry! Actual Inj Volume : 2.000 µl
Acq. Method : C:\Users\Public\Documents\ChemStation\1\Data\SUN\SUN 2024-12-02 21-37-50\IBN3-10-20.M
Last changed : 5/1/2024 8:18:08 PM by SYSTEM
Analysis Method : C:\Users\Public\Documents\ChemStation\1\Data\SUN\SUN 2024-12-02 21-37-50\IBN3-10-20.M (Sequence Method)
Last changed : 12/3/2024 9:32:14 AM by SYSTEM
(modified after loading)
Additional Info : Peak(s) manually integrated

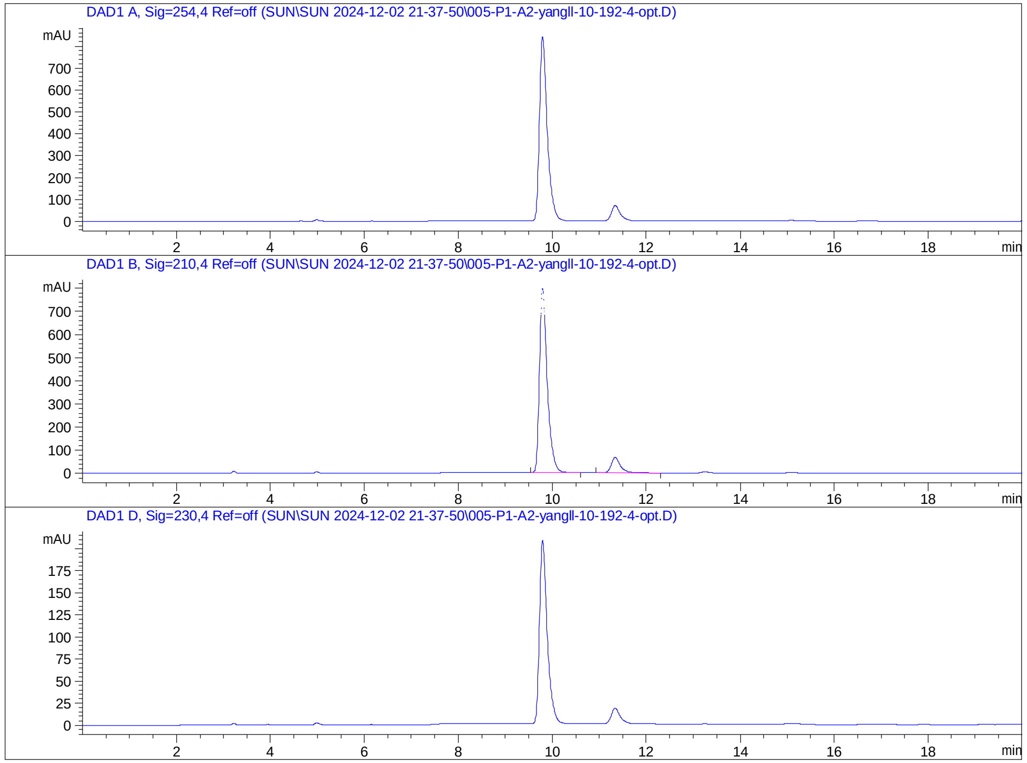

===

Area Percent Report

===

Sorted By : Signal
Multiplier : 1.0000
Dilution : 1.0000
Use Multiplier & Dilution Factor with ISTDs

Data File C:\Users\P...\1\Data\SUN\SUN 2024-12-02 21-37-50\005-P1-A2-yangll-10-192-4-opt.D
Sample Name: yangll-10-192-4-opt

Signal 1: DAD1 A, Sig=254,4 Ref=off

Signal 2: DAD1 B, Sig=210,4 Ref=off

Peak #	RetTime [min]	Type	Width [min]	Area [mAU*s]	Height [mAU]	Area %
1	9.793	BB	0.1755	9231.21094	794.73938	91.2367
2	11.338	BB	0.1989	886.66455	66.76888	8.7633

Totals : 1.01179e4 861.50826

Signal 3: DAD1 D, Sig=230,4 Ref=off

===

*** End of Report ***

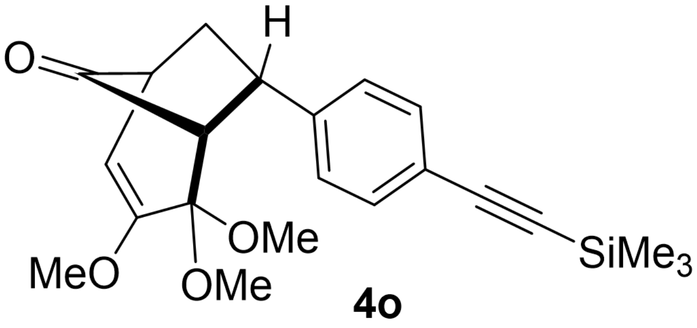

4o

opt

===

Acq. Operator : SYSTEM Seq. Line : 2
Acq. Instrument : LC1260 Location : P1-F-01
Injection Date : 7/28/2024 5:21:39 PM Inj : 1
Inj Volume : 5.000 µl
Different Inj Volume from Sample Entry! Actual Inj Volume : 10.000 µl
Acq. Method : C:\Users\Public\Documents\ChemStation\1\Data\SUN\SUN 2024-07-28 17-08-50\IBN3-30-30.M
Last changed : 7/28/2024 5:33:04 PM by SYSTEM (modified after loading)
Analysis Method : C:\Users\Public\Documents\ChemStation\1\Data\SUN\SUN 2024-07-28 17-08-50\IBN3-30-30.M (Sequence Method)
Last changed : 7/29/2024 10:40:16 AM by SYSTEM (modified after loading)
Additional Info : Peak(s) manually integrated

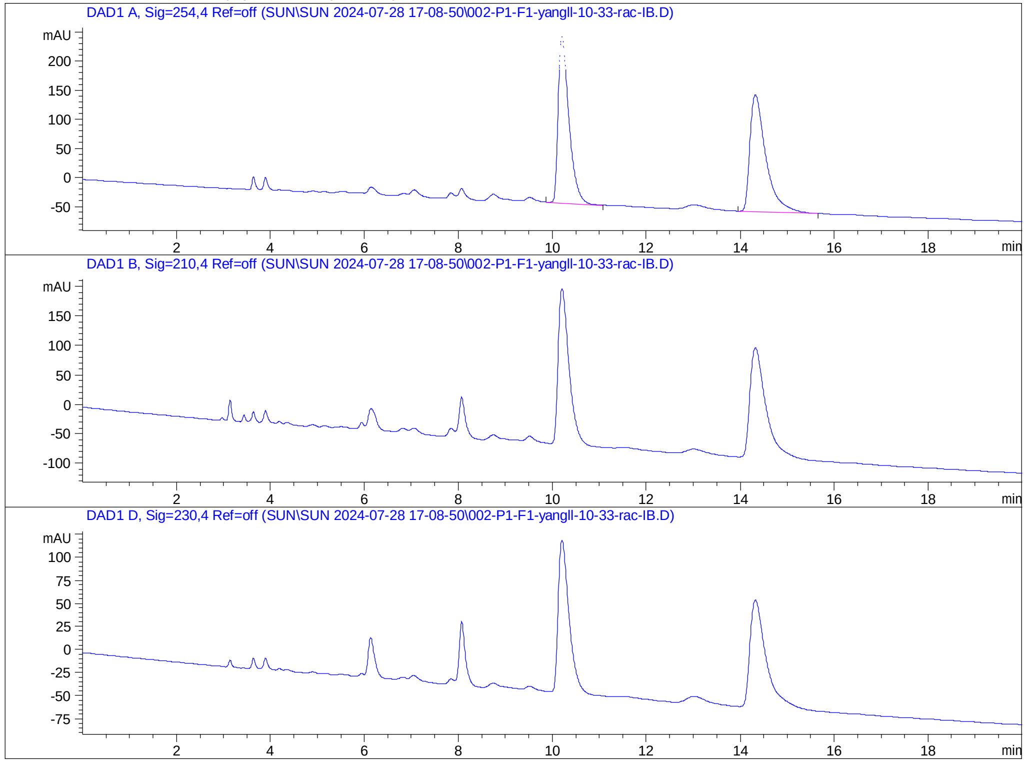

Data File C:\Users\P...\1\Data\SUN\SUN 2024-07-28 17-08-50\002-P1-F1-yangll-10-33-rac-IB.D
Sample Name: yangll-10-33-rac-IB

===

Area Percent Report

===

Sorted By : Signal
Multiplier : 1.0000
Dilution : 1.0000
Use Multiplier & Dilution Factor with ISTDs

Signal 1: DAD1 A, Sig=254,4 Ref=off

Peak #	RetTime [min]	Type	Width [min]	Area [mAU*s]	Height [mAU]	Area %
1	10.208	MM R	0.2701	4645.59473	286.64413	49.2744
2	14.323	MM R	0.3961	4782.40479	201.21130	50.7256

Totals : 9427.99951 487.85544

Signal 2: DAD1 B, Sig=210,4 Ref=off

Signal 3: DAD1 D, Sig=230,4 Ref=off

===

*** End of Report ***

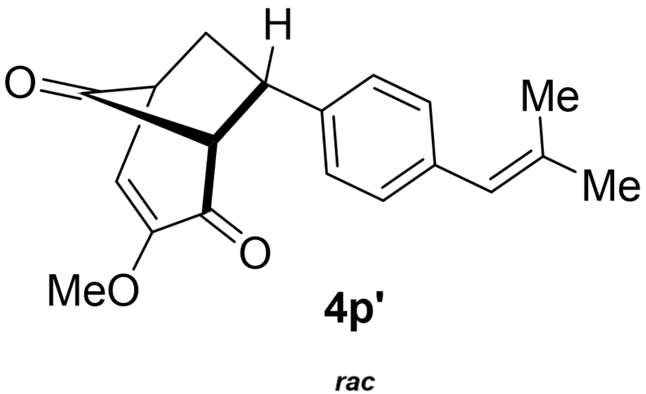

4p'

rac

===

Acq. Operator : SYSTEM Seq. Line : 5
Acq. Instrument : LC1260 Location : P1-F-03
Injection Date : 7/28/2024 7:24:44 PM Inj : 1
Inj Volume : 5.000 µl
Acq. Method : C:\Users\Public\Documents\ChemStation\1\Data\SUN\SUN 2024-07-28 17-08-50 \IBN3-30-30.M
Last changed : 7/28/2024 6:04:19 PM by SYSTEM
Analysis Method : C:\Users\Public\Documents\ChemStation\1\Data\SUN\SUN 2024-07-28 17-08-50 \IBN3-30-30.M (Sequence Method)
Last changed : 7/29/2024 10:42:09 AM by SYSTEM
(modified after loading)
Additional Info : Peak(s) manually integrated

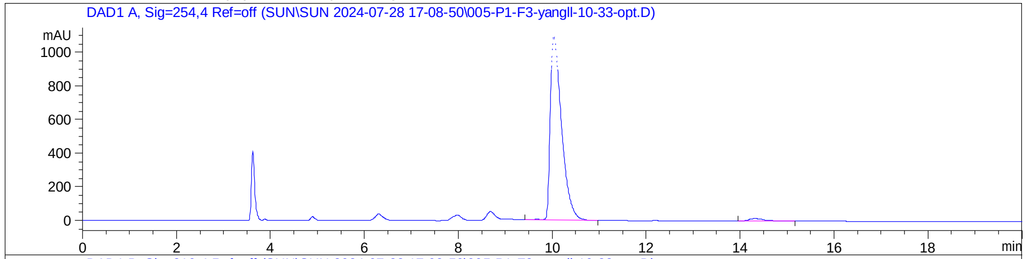

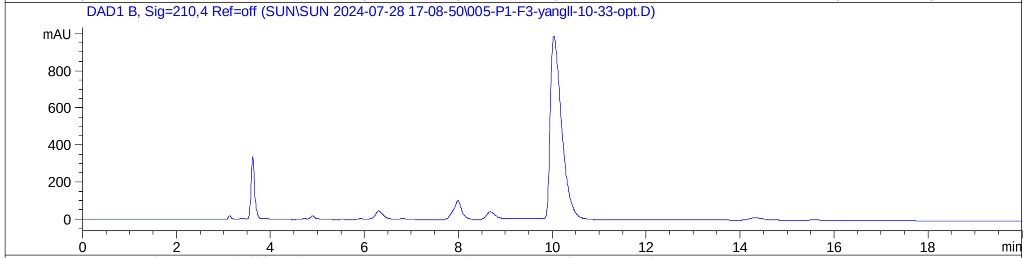

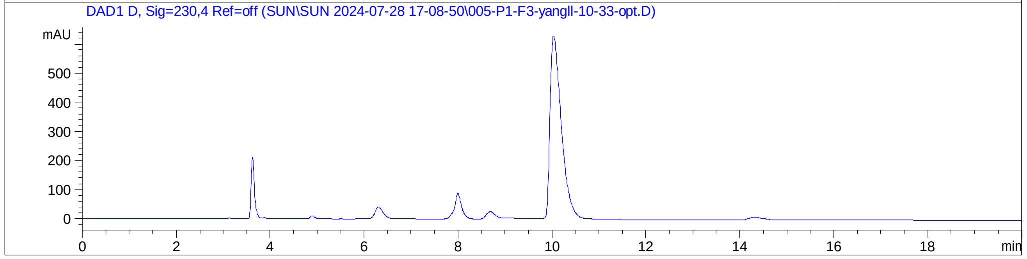

===
Area Percent Report
===

Sorted By : Signal
Multiplier : 1.0000
Dilution : 1.0000
Use Multiplier & Dilution Factor with ISTDs

Data File C:\Users\P...ion\1\Data\SUN\SUN 2024-07-28 17-08-50\005-P1-F3-yangll-10-33-opt.D
Sample Name: yangll-10-33-opt

Signal 1: DAD1 A, Sig=254,4 Ref=off

Peak #	RetTime [min]	Type	Width [min]	Area [mAU*s]	Height [mAU]	Area %
1	10.039	BB	0.2560	1.83016e4	1089.43506	98.2156
2	14.320	BB	0.3225	332.50375	15.48526	1.7844

Totals : 1.86342e4 1104.92031

Signal 2: DAD1 B, Sig=210,4 Ref=off

Signal 3: DAD1 D, Sig=230,4 Ref=off

===
*** End of Report ***

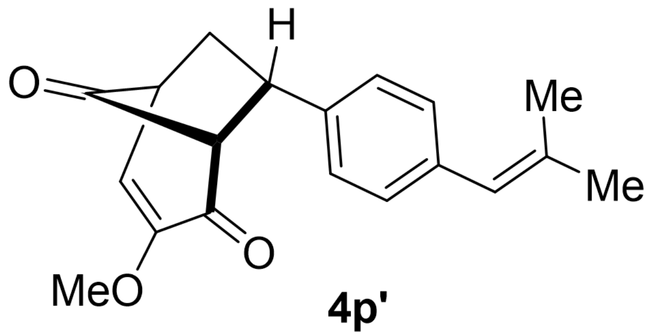

4p'

opt

===

Acq. Operator : SYSTEM Seq. Line : 43
Sample Operator : SYSTEM
Acq. Instrument : HPLC Location : P1-A-01
Injection Date : 29/7/2023 11:32:46 pm Inj : 1
Inj Volume : 2.000 µl
Acq. Method : C:\Users\Public\Documents\ChemStation\1\Data\SUN\SUN 2023-07-29 09-59-19\ID3-10-20.M
Last changed : 19/8/2022 10:53:06 pm by SYSTEM
Analysis Method : C:\Users\Public\Documents\ChemStation\1\Data\SUN\SUN 2023-07-29 09-59-19\ID3-10-20.M (Sequence Method)
Last changed : 8/5/2024 10:21:52 pm by SYSTEM
(modified after loading)
Additional Info : Peak(s) manually integrated

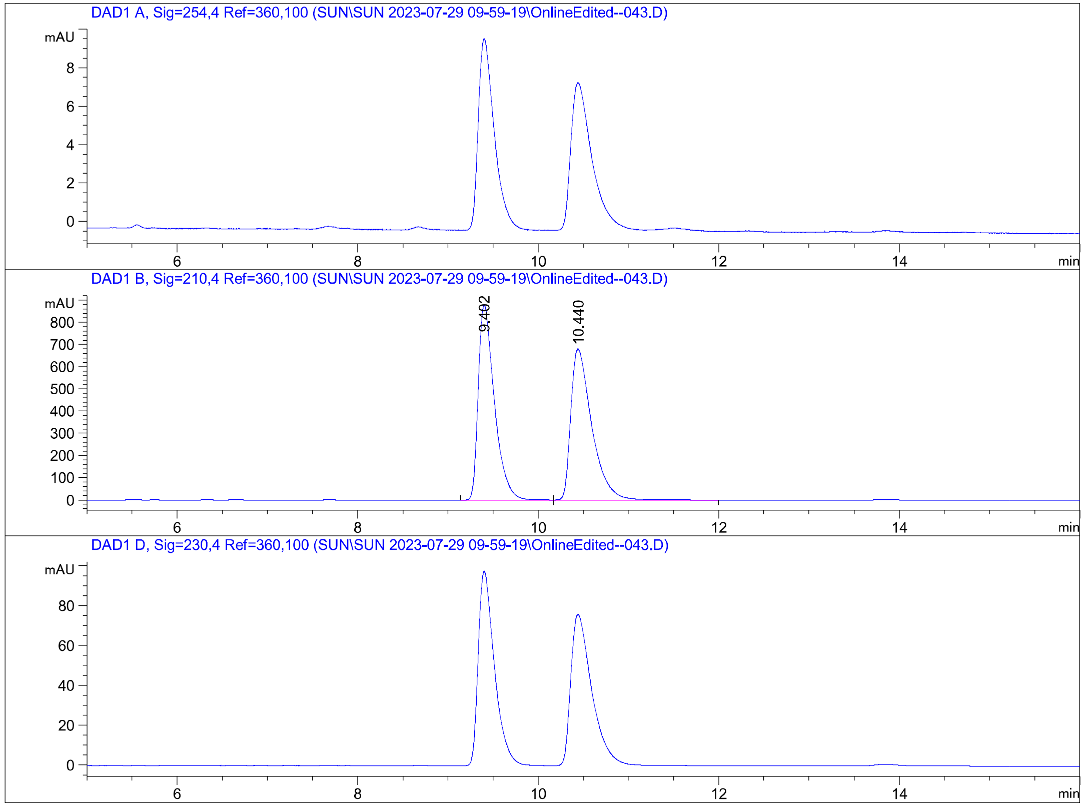

===

Area Percent Report

===

Sorted By : Signal
Multiplier : 1.0000
Dilution : 1.0000
Use Multiplier & Dilution Factor with ISTDs

Data File C:\Users\P...\ChemStation\1\Data\SUN\SUN 2023-07-29 09-59-19\OnlineEdited--043.D
Sample Name: yangll-7-88-2-rac-id

Signal 1: DAD1 A, Sig=254,4 Ref=360,100

Signal 2: DAD1 B, Sig=210,4 Ref=360,100

Peak #	RetTime [min]	Type	Width [min]	Area [mAU*s]	Height [mAU]	Area %
1	9.402	BB	0.1870	1.09156e4	878.47919	49.9714
2	10.440	BB	0.2425	1.09281e4	679.87140	50.0286

Totals : 2.18438e4 1558.35059

Signal 3: DAD1 D, Sig=230,4 Ref=360,100

===

*** End of Report ***

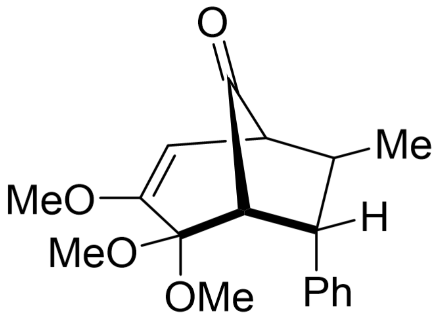

4r

rac

===

Acq. Operator : SYSTEM   Seq. Line : 37
Sample Operator : SYSTEM
Acq. Instrument : HPLC   Location : P1-A-05
Injection Date : 12/11/2023 8:47:18 am   Inj : 1
Inj Volume : 2.000 µl
Acq. Method : C:\Users\Public\Documents\ChemStation\1\Data\SUN\SUN 2023-11-11 18-12-21 \ID3-10-20.M
Last changed : 2/8/2023 12:18:49 pm by SYSTEM
Analysis Method : C:\Users\Public\Documents\ChemStation\1\Data\SUN\SUN 2023-11-11 18-12-21 \ID3-10-20.M (Sequence Method)
Last changed : 8/5/2024 10:17:20 pm by SYSTEM
(modified after loading)
Additional Info : Peak(s) manually integrated

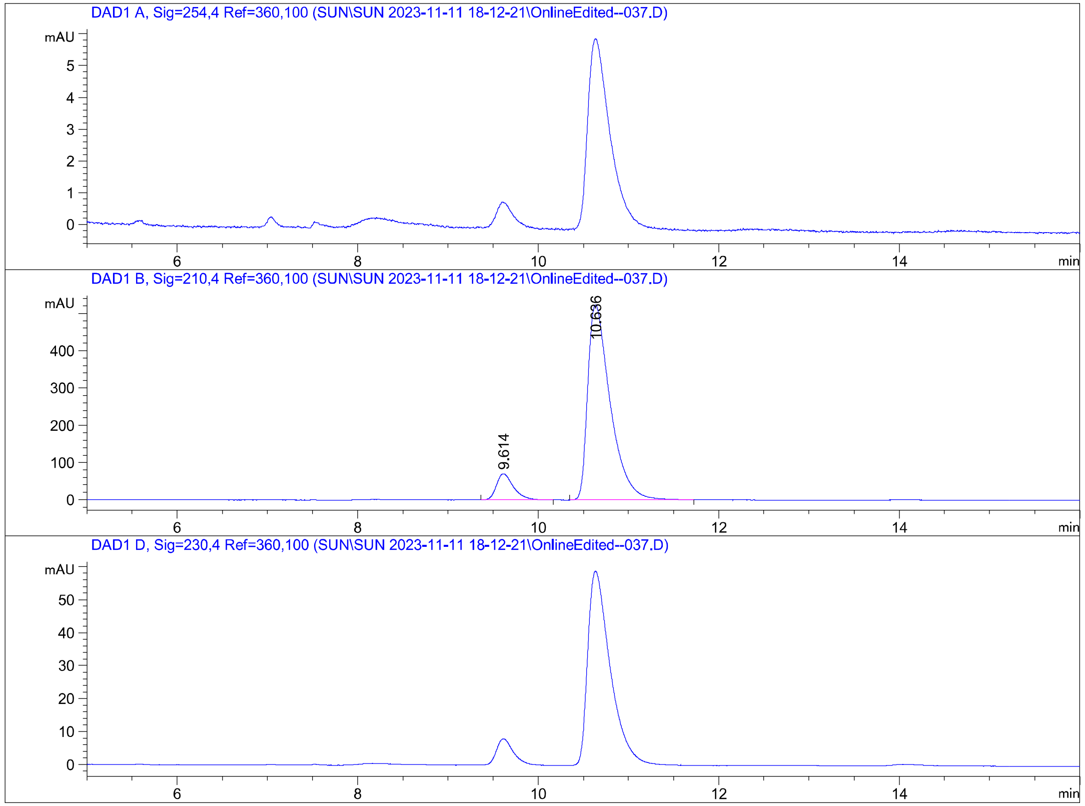

===

Area Percent Report

===

Sorted By : Signal
Multiplier : 1.0000
Dilution : 1.0000
Use Multiplier & Dilution Factor with ISTDs

Data File C:\Users\P...\ChemStation\1\Data\SUN\SUN 2023-11-11 18-12-21\OnlineEdited--037.D
Sample Name: yangll-8-53-1

Signal 1: DAD1 A, Sig=254,4 Ref=360,100

Signal 2: DAD1 B, Sig=210,4 Ref=360,100

Peak #	RetTime [min]	Type	Width [min]	Area [mAU*s]	Height [mAU]	Area %
1	9.614	BB	0.1901	908.13403	70.10370	9.1489
2	10.636	BV R	0.2554	9017.98438	522.49200	90.8511

Totals : 9926.11841 592.59570

Signal 3: DAD1 D, Sig=230,4 Ref=360,100

===

*** End of Report ***

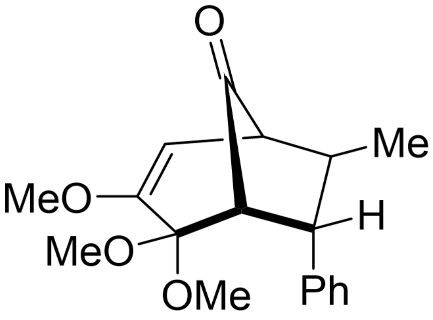

4r

opt

===

Acq. Operator : SYSTEM Seq. Line : 36
Sample Operator : SYSTEM
Acq. Instrument : HPLC Location : P1-F-04
Injection Date : 31/1/2024 10:41:27 pm Inj : 1
Inj Volume : 2.000 µl
Different Inj Volume from Sample Entry! Actual Inj Volume : 3.000 µl
Acq. Method : C:\Users\Public\Documents\ChemStation\1\Data\SUN\SUN 2024-01-31 10-36-02 \ID3-20-30.M
Last changed : 31/1/2024 11:09:54 pm by SYSTEM (modified after loading)
Analysis Method : C:\Users\Public\Documents\ChemStation\1\Data\SUN\SUN 2024-01-31 10-36-02 \ID3-20-30.M (Sequence Method)
Last changed : 31/1/2024 11:13:35 pm by SYSTEM
Additional Info : Peak(s) manually integrated

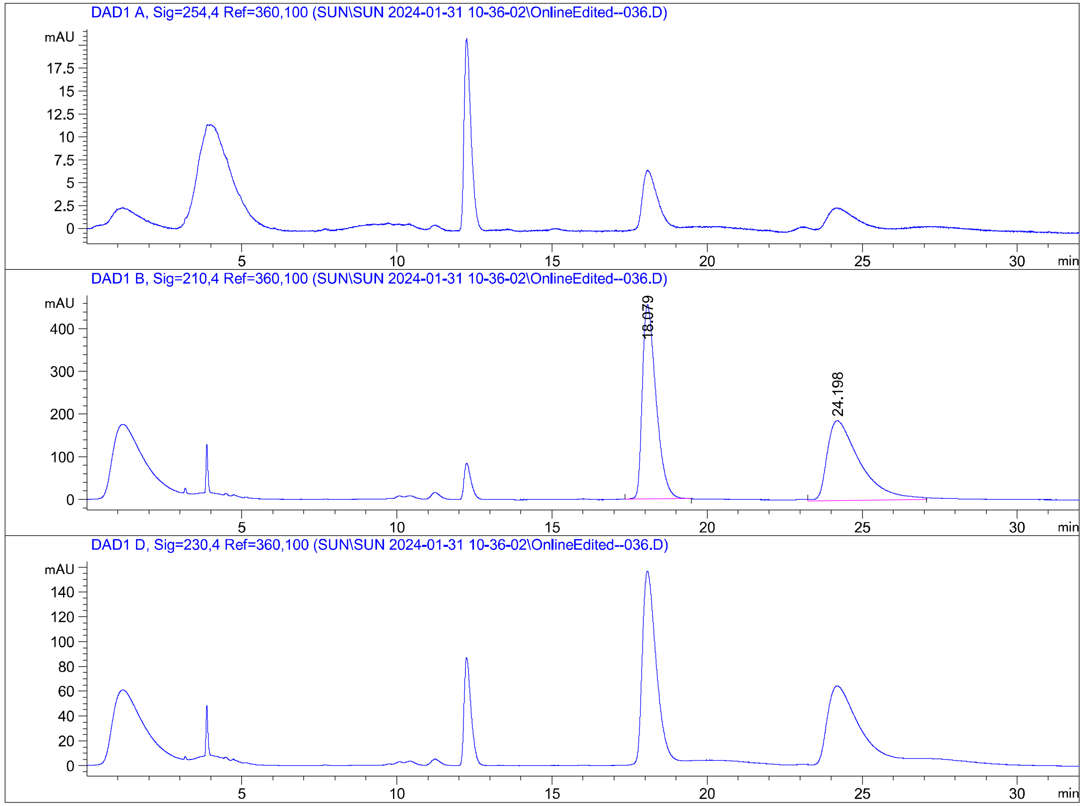

Data File C:\Users\P...\ChemStation\1\Data\SUN\SUN 2024-01-31 10-36-02\OnlineEdited--036.D
Sample Name: yangll-9-35-rac-ID2

===

Area Percent Report

===

Sorted By : Signal
Multiplier : 1.0000
Dilution : 1.0000
Use Multiplier & Dilution Factor with ISTDs

Signal 1: DAD1 A, Sig=254,4 Ref=360,100

Signal 2: DAD1 B, Sig=210,4 Ref=360,100

Peak #	RetTime [min]	Type	Width [min]	Area [mAU*s]	Height [mAU]	Area %
1	18.079	VV R	0.4208	1.40149e4	455.67743	50.4690
2	24.198	MM R	1.2235	1.37544e4	187.36086	49.5310
Totals :				2.77694e4	643.03828	

Signal 3: DAD1 D, Sig=230,4 Ref=360,100

===

*** End of Report ***

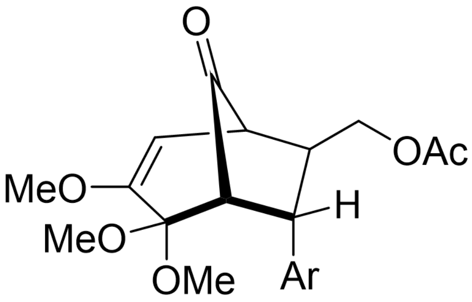

Ar = 4-$CH_3C_6H_4$

4s

rac

===

```
Acq. Operator   : SYSTEM                        Seq. Line :  26
Sample Operator : SYSTEM
Acq. Instrument : HPLC                           Location :  P1-A-02
Injection Date  : 8/5/2024 1:49:23 am                 Inj :  1
                                               Inj Volume : 2.000 µl
Different Inj Volume from Sample Entry!  Actual Inj Volume : 5.000 µl
Method          : C:\Users\Public\Documents\ChemStation\1\Data\SUN\SUN 2024-05-07 18-15-38
                  \ID3-20-40.M (Sequence Method)
Last changed    : 4/1/2023 3:55:58 pm by SYSTEM
Additional Info : Peak(s) manually integrated
```

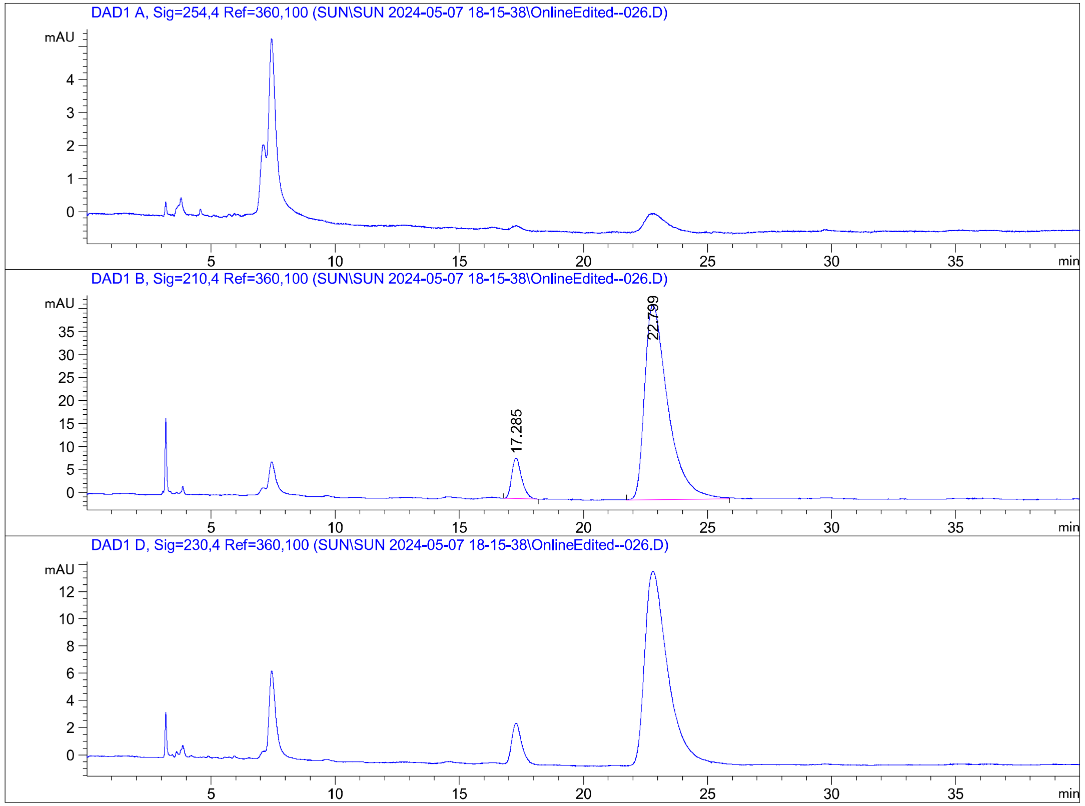

===
Area Percent Report
===

```
Sorted By             :      Signal
Multiplier            :      1.0000
Dilution              :      1.0000
Use Multiplier & Dilution Factor with ISTDs
```


Data File C:\Users\P...\ChemStation\1\Data\SUN\SUN 2024-05-07 18-15-38\OnlineEdited--026.D
Sample Name: yangll-9-126-opt

Signal 1: DAD1 A, Sig=254,4 Ref=360,100

Signal 2: DAD1 B, Sig=210,4 Ref=360,100

Peak #	RetTime [min]	Type	Width [min]	Area [mAU*s]	Height [mAU]	Area %
1	17.285	BB	0.3346	244.87628	8.82883	8.1339
2	22.799	MM R	1.0872	2765.70557	42.39845	91.8661

Totals : 3010.58185 51.22729

Signal 3: DAD1 D, Sig=230,4 Ref=360,100

===
*** End of Report ***

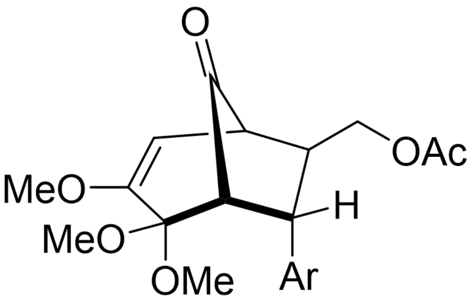

Ar = 4-$CH_3C_6H_4$

4s

opt

===

Acq. Operator : SYSTEM Seq. Line : 10
Sample Operator : SYSTEM
Acq. Instrument : HPLC Location : P1-F-03
Injection Date : 3/2/2024 6:44:26 pm Inj : 1
Inj Volume : 2.000 µl
Different Inj Volume from Sample Entry! Actual Inj Volume : 4.000 µl
Acq. Method : C:\Users\Public\Documents\ChemStation\1\Data\SUN\SUN 2024-02-03 16-03-18\IBN3-10-20.M
Last changed : 30/7/2023 1:22:19 pm by SYSTEM
Analysis Method : C:\Users\Public\Documents\ChemStation\1\Data\SUN\SUN 2024-02-03 16-03-18\IBN3-10-20.M (Sequence Method)
Last changed : 7/5/2024 8:40:10 pm by SYSTEM
(modified after loading)
Additional Info : Peak(s) manually integrated

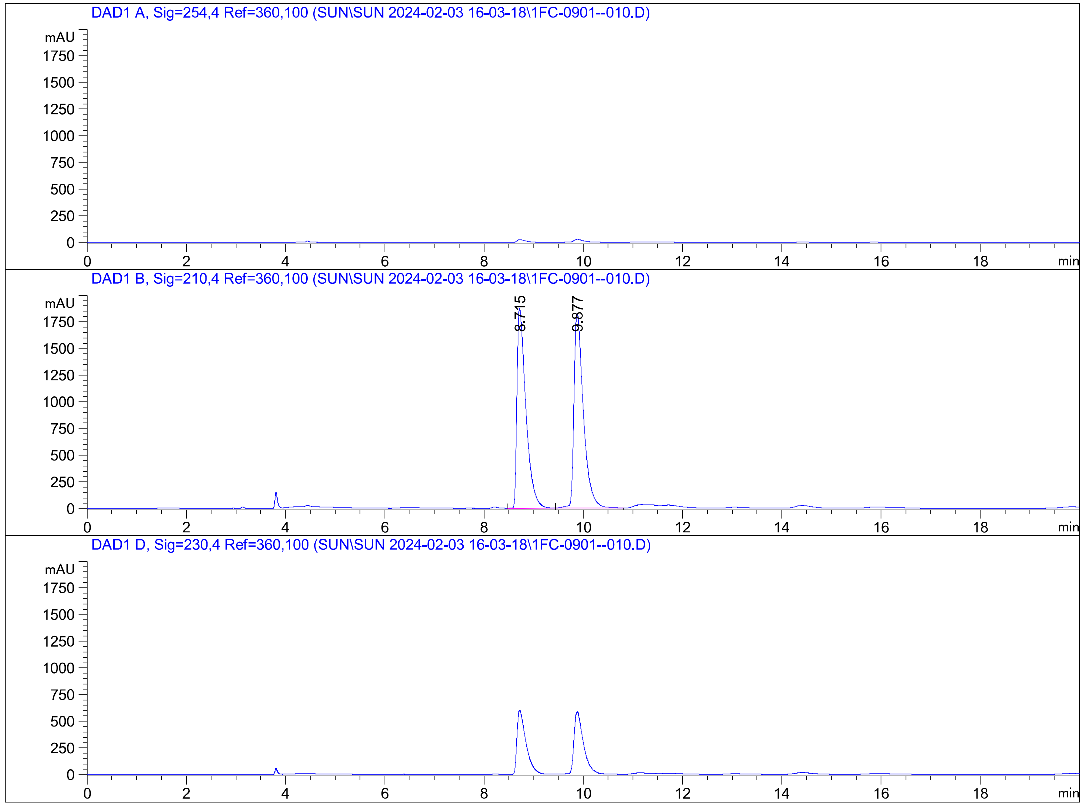

Data File C:\Users\P...ents\ChemStation\1\Data\SUN\SUN 2024-02-03 16-03-18\1FC-0901--010.D
Sample Name: yangll-9-41-rac-IB

===

Area Percent Report

===

Sorted By : Signal
Multiplier : 1.0000
Dilution : 1.0000
Use Multiplier & Dilution Factor with ISTDs

Signal 1: DAD1 A, Sig=254,4 Ref=360,100

Signal 2: DAD1 B, Sig=210,4 Ref=360,100

Peak #	RetTime [min]	Type	Width [min]	Area [mAU*s]	Height [mAU]	Area %
1	8.715	BB	0.1664	2.34916e4	1872.51733	49.3254
2	9.877	BB	0.1973	2.41342e4	1824.20911	50.6746

Totals : 4.76258e4 3696.72644

Signal 3: DAD1 D, Sig=230,4 Ref=360,100

===

*** End of Report ***

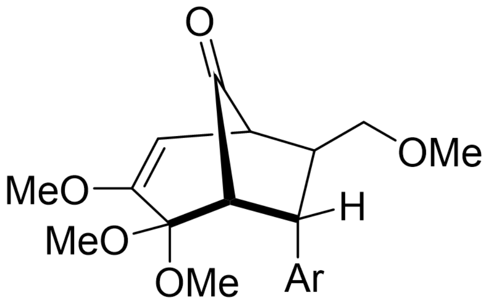

Ar = 4-$CH_3C_6H_4$

4t

rac

===

Acq. Operator : SYSTEM Seq. Line : 24
Sample Operator : SYSTEM
Acq. Instrument : HPLC Location : P1-A-01
Injection Date : 8/5/2024 1:17:20 am Inj : 1
Inj Volume : 2.000 µl
Different Inj Volume from Sample Entry! Actual Inj Volume : 5.000 µl
Method : C:\Users\Public\Documents\ChemStation\1\Data\SUN\SUN 2024-05-07 18-15-38
\IBN3-10-20.M (Sequence Method)
Last changed : 30/7/2023 1:22:19 pm by SYSTEM
Additional Info : Peak(s) manually integrated

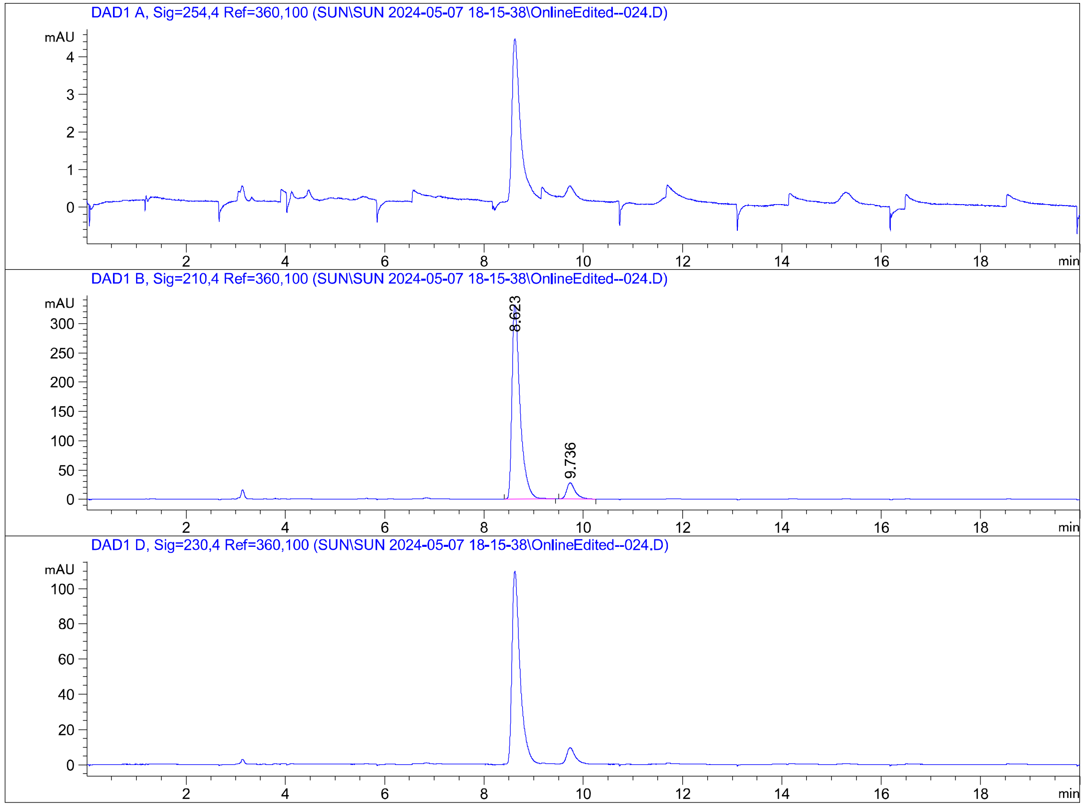

===
Area Percent Report
===

Sorted By : Signal
Multiplier : 1.0000
Dilution : 1.0000
Use Multiplier & Dilution Factor with ISTDs

Data File C:\Users\P...\ChemStation\1\Data\SUN\SUN 2024-05-07 18-15-38\OnlineEdited--024.D
Sample Name: yangll-9-122-OPT

Signal 1: DAD1 A, Sig=254,4 Ref=360,100

Signal 2: DAD1 B, Sig=210,4 Ref=360,100

Peak #	RetTime [min]	Type	Width [min]	Area [mAU*s]	Height [mAU]	Area %
1	8.623	BV R	0.1692	3750.45483	331.00540	91.9791
2	9.736	BB	0.1771	327.05249	27.52389	8.0209

Totals : 4077.50732 358.52929

Signal 3: DAD1 D, Sig=230,4 Ref=360,100

===
*** End of Report ***

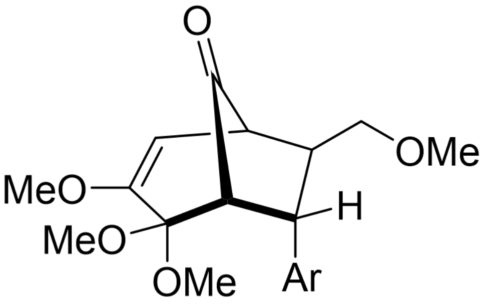

Ar = 4-$CH_3C_6H_4$

4t

opt

===

Acq. Operator : SYSTEM Seq. Line : 22
Sample Operator : SYSTEM
Acq. Instrument : HPLC Location : P1-F-04
Injection Date : 3/2/2024 10:28:54 pm Inj : 1
Inj Volume : 2.000 µl
Different Inj Volume from Sample Entry! Actual Inj Volume : 1.000 µl
Acq. Method : C:\Users\Public\Documents\ChemStation\1\Data\SUN\SUN 2024-02-03 16-03-18\AD3-10-30.M
Last changed : 24/10/2022 9:57:21 am by SYSTEM
Analysis Method : C:\Users\Public\Documents\ChemStation\1\Data\SUN\SUN 2024-02-03 16-03-18\AD3-10-30.M (Sequence Method)
Last changed : 4/5/2024 11:35:54 pm by SYSTEM
(modified after loading)
Additional Info : Peak(s) manually integrated

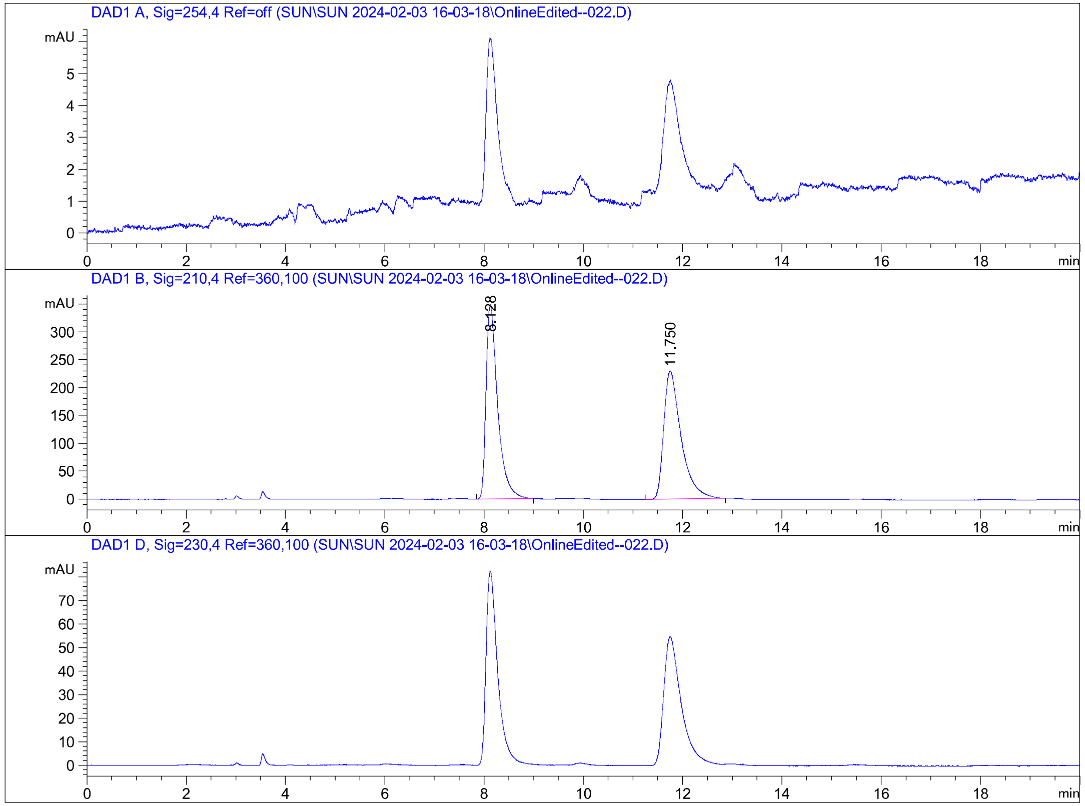

Data File C:\Users\P...\ChemStation\1\Data\SUN\SUN 2024-02-03 16-03-18\OnlineEdited--022.D
Sample Name: yangll-9-42-rac-AD

===
Area Percent Report
===

Sorted By : Signal
Multiplier : 1.0000
Dilution : 1.0000
Use Multiplier & Dilution Factor with ISTDs

Signal 1: DAD1 A, Sig=254,4 Ref=off

Signal 2: DAD1 B, Sig=210,4 Ref=360,100

Peak #	RetTime [min]	Type	Width [min]	Area [mAU*s]	Height [mAU]	Area %
1	8.128	BV R	0.2366	5644.49805	348.23495	50.2419
2	11.750	VV R	0.3396	5590.15381	229.97829	49.7581

Totals : 1.12347e4 578.21324

Signal 3: DAD1 D, Sig=230,4 Ref=360,100

===
*** End of Report ***

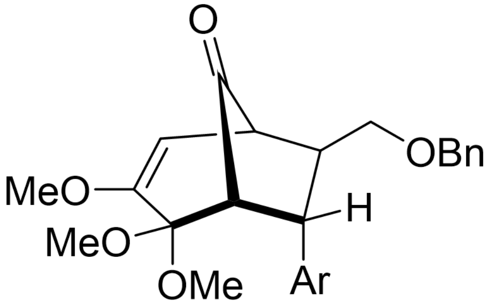

Ar = 4-$CH_3C_6H_4$

4u

rac

===

Acq. Operator : SYSTEM Seq. Line : 5
Sample Operator : SYSTEM
Acq. Instrument : HPLC Location : P1-B-03
Injection Date : 4/5/2024 4:56:26 pm Inj : 1
Inj Volume : 2.000 µl
Method : C:\Users\Public\Documents\ChemStation\1\Data\SUN\SUN 2024-05-04 15-50-03
\AD3-10-20.M (Sequence Method)
Last changed : 15/8/2022 10:21:32 pm by SYSTEM
Additional Info : Peak(s) manually integrated

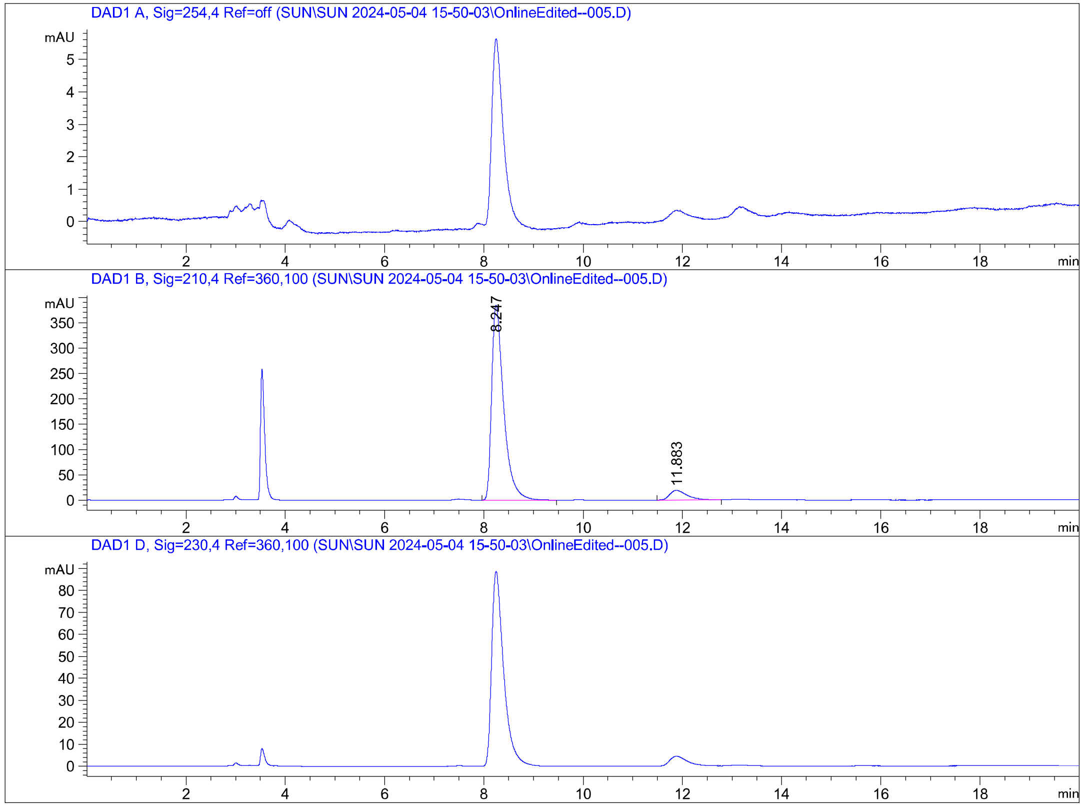

===
Area Percent Report
===

Sorted By : Signal
Multiplier : 1.0000
Dilution : 1.0000
Use Multiplier & Dilution Factor with ISTDs

Signal 1: DAD1 A, Sig=254,4 Ref=off

Signal 2: DAD1 B, Sig=210,4 Ref=360,100

Peak #	RetTime [min]	Type	Width [min]	Area [mAU*s]	Height [mAU]	Area %
1	8.247	BB	0.2498	6455.82959	384.64929	93.2929
2	11.883	BB	0.3001	464.13208	19.13023	6.7071

Totals : 6919.96167 403.77952

Signal 3: DAD1 D, Sig=230,4 Ref=360,100

===
*** End of Report ***

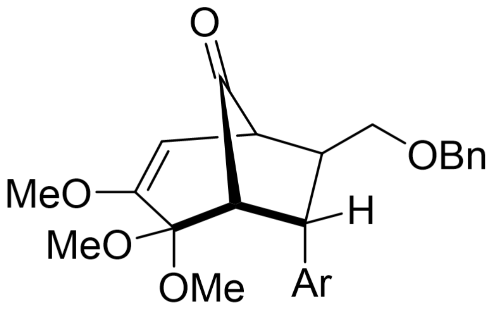

Ar = 4-$CH_3C_6H_4$

4u

opt

===

Acq. Operator : SYSTEM Seq. Line : 3
Acq. Instrument : LC1260 Location : P1-C-01
Injection Date : 3/9/2024 4:09:55 PM Inj : 1
Inj Volume : 5.000 µl
Acq. Method : C:\Users\Public\Documents\ChemStation\1\Data\SUN\SUN 2024-03-09 15-36-55\AD3-10-20.M
Last changed : 3/7/2024 1:13:10 PM by SYSTEM
Analysis Method : C:\Users\Public\Documents\ChemStation\1\Data\SUN\SUN 2024-03-09 15-36-55\AD3-10-20.M (Sequence Method)
Last changed : 6/3/2024 10:38:26 PM by SYSTEM
(modified after loading)
Additional Info : Peak(s) manually integrated

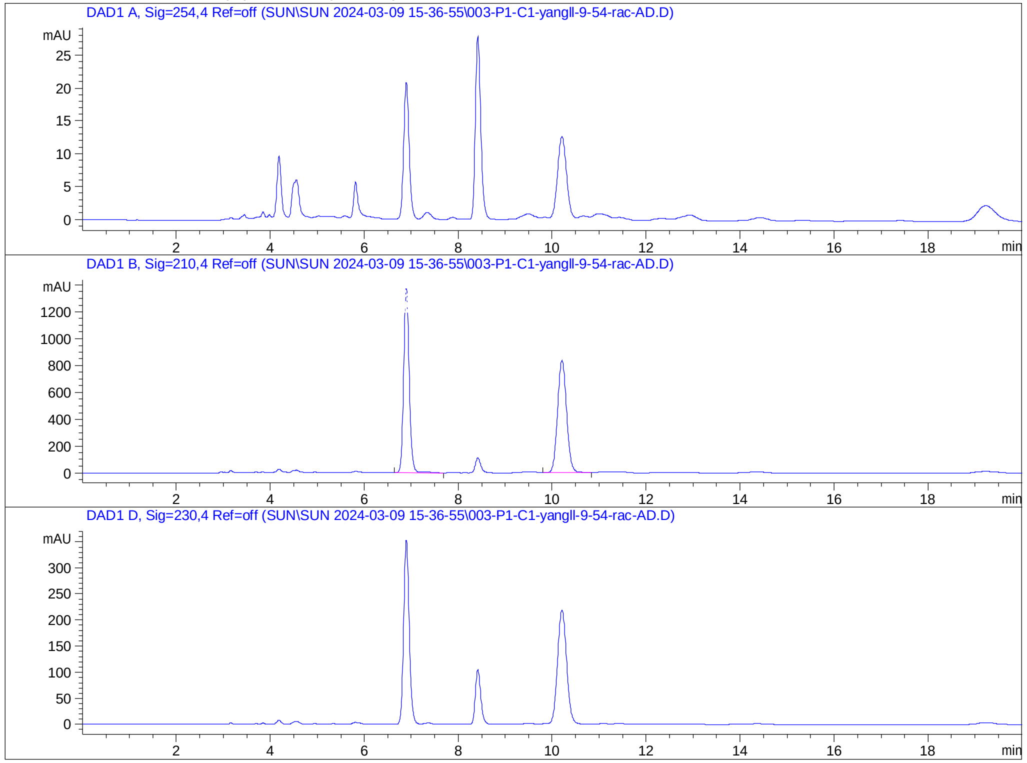

===

Area Percent Report

===

Sorted By : Signal
Multiplier : 1.0000
Dilution : 1.0000
Use Multiplier & Dilution Factor with ISTDs

Signal 1: DAD1 A, Sig=254,4 Ref=off

Signal 2: DAD1 B, Sig=210,4 Ref=off

Peak #	RetTime [min]	Type	Width [min]	Area [mAU*s]	Height [mAU]	Area %
1	6.895	BB R	0.1226	1.12242e4	1375.11523	50.0734
2	10.211	MM R	0.2226	1.11913e4	838.05603	49.9266

Totals : 2.24156e4 2213.17126

Signal 3: DAD1 D, Sig=230,4 Ref=off

===

*** End of Report ***

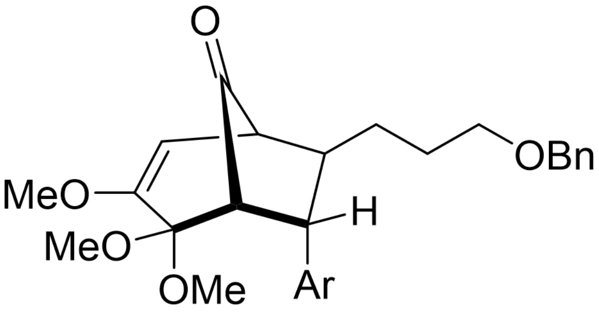

Ar = 4-$CH_3C_6H_4$

4v

rac

===

```
Acq. Operator   : SYSTEM                        Seq. Line :    2
Acq. Instrument : LC1260                         Location :    P1-B-02
Injection Date  : 5/4/2024 6:32:06 PM                 Inj :    1
                                               Inj Volume : 5.000 µl
Different Inj Volume from Sample Entry!  Actual Inj Volume : 2.000 µl
Acq. Method     : C:\Users\Public\Documents\ChemStation\1\Data\SUN\SUN 2024-05-04 18-19-14
                  \AD3-10-20.M
Last changed    : 3/7/2024 1:13:10 PM by SYSTEM
Analysis Method : C:\Users\Public\Documents\ChemStation\1\Data\SUN\SUN 2024-05-04 18-19-14
                  \AD3-10-20.M (Sequence Method)
Last changed    : 5/4/2024 11:21:46 PM by SYSTEM
                  (modified after loading)
Additional Info : Peak(s) manually integrated
```

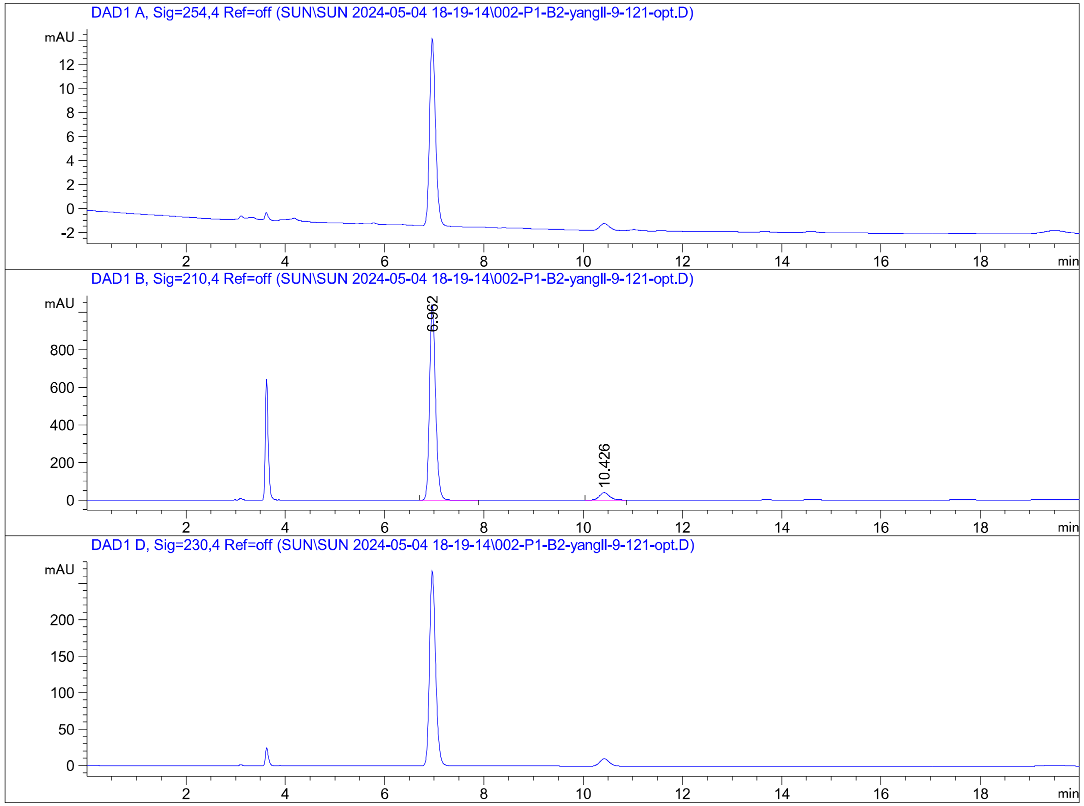

===

Area Percent Report

===

```
Sorted By             :      Signal
Multiplier            :      1.0000
Dilution              :      1.0000
Use Multiplier & Dilution Factor with ISTDs
```


Data File C:\Users\P...ion\1\Data\SUN\SUN 2024-05-04 18-19-14\002-P1-B2-yangll-9-121-opt.D
Sample Name: yangll-9-121-opt

Signal 1: DAD1 A, Sig=254,4 Ref=off

Signal 2: DAD1 B, Sig=210,4 Ref=off

Peak #	RetTime [min]	Type	Width [min]	Area [mAU*s]	Height [mAU]	Area %
1	6.962	BB	0.1284	8751.15625	1037.70300	94.2825
2	10.426	BB	0.2105	530.68634	39.05636	5.7175

Totals : 9281.84259 1076.75936

Signal 3: DAD1 D, Sig=230,4 Ref=off

===

*** End of Report ***

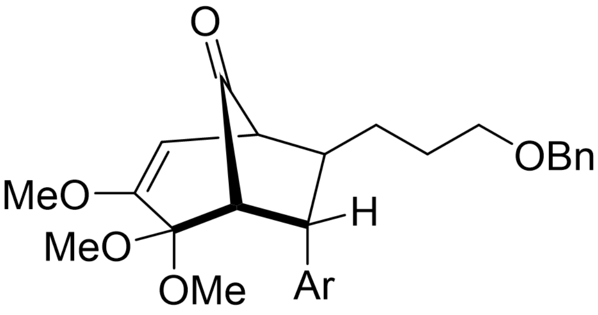

Ar = 4-$CH_3C_6H_4$

4v

opt

===

Acq. Operator : SYSTEM Seq. Line : 3
Acq. Instrument : LC1260 Location : P1-C-01
Injection Date : 3/13/2024 3:54:12 PM Inj : 1
Inj Volume : 5.000 µl
Different Inj Volume from Sample Entry! Actual Inj Volume : 4.000 µl
Acq. Method : C:\Users\Public\Documents\ChemStation\1\Data\SUN\SUN 2024-03-13 15-31-12\AD3-10-20.M
Last changed : 3/13/2024 4:06:52 PM by SYSTEM
(modified after loading)
Analysis Method : C:\Users\Public\Documents\ChemStation\1\Data\SUN\SUN 2024-03-13 15-31-12\AD3-10-20.M (Sequence Method)
Last changed : 5/7/2024 8:05:27 PM by SYSTEM
(modified after loading)
Additional Info : Peak(s) manually integrated

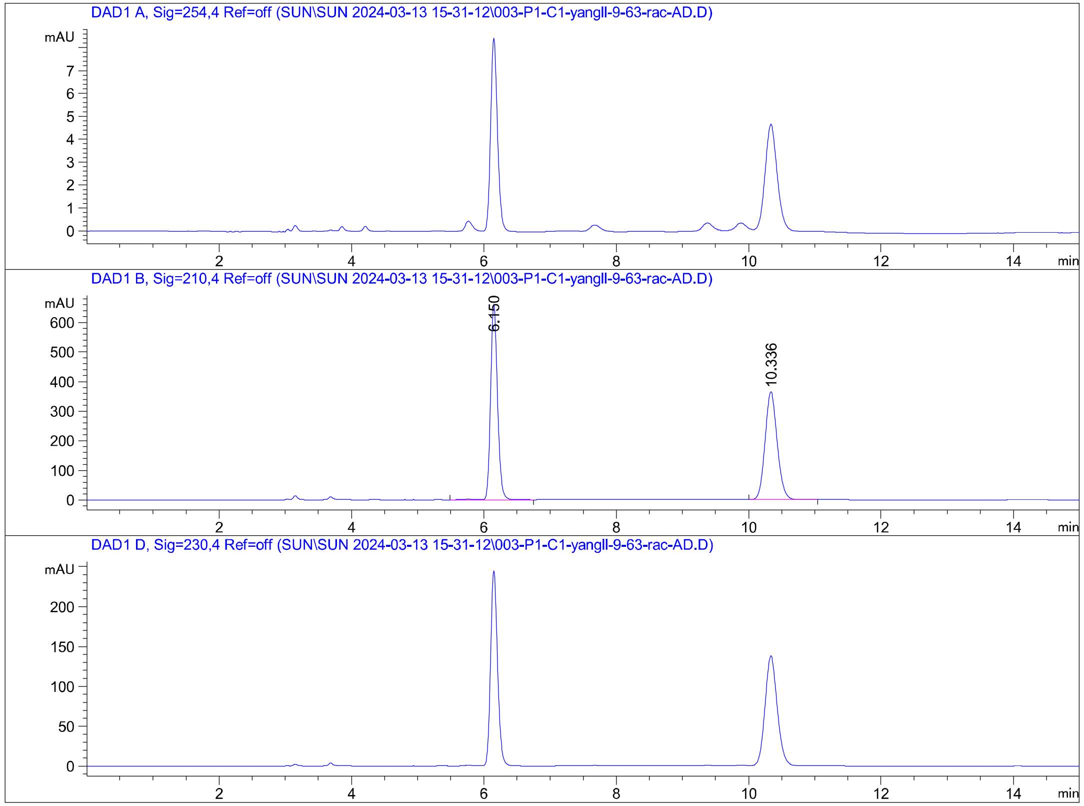

Data File C:\Users\P...n\1\Data\SUN\SUN 2024-03-13 15-31-12\003-P1-C1-yangll-9-63-rac-AD.D
Sample Name: yangll-9-63-rac-AD

===
Area Percent Report
===

Sorted By : Signal
Multiplier : 1.0000
Dilution : 1.0000
Use Multiplier & Dilution Factor with ISTDs

Signal 1: DAD1 A, Sig=254,4 Ref=off

Signal 2: DAD1 B, Sig=210,4 Ref=off

Peak #	RetTime [min]	Type	Width [min]	Area [mAU*s]	Height [mAU]	Area %
1	6.150	BB R	0.1085	4675.57129	659.11737	50.0746
2	10.336	BB	0.1990	4661.63721	365.08173	49.9254

Totals : 9337.20850 1024.19910

Signal 3: DAD1 D, Sig=230,4 Ref=off

===
*** End of Report ***

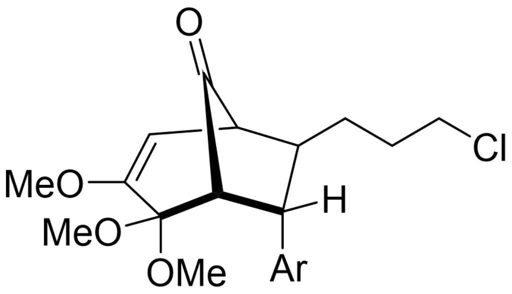

Ar = 4-$CH_3C_6H_4$

4w

rac

===

Acq. Operator : SYSTEM Seq. Line : 28
Sample Operator : SYSTEM
Acq. Instrument : HPLC Location : P1-A-03
Injection Date : 8/5/2024 2:41:35 am Inj : 1
Inj Volume : 2.000 µl
Different Inj Volume from Sample Entry! Actual Inj Volume : 5.000 µl
Acq. Method : C:\Users\Public\Documents\ChemStation\1\Data\SUN\SUN 2024-05-07 18-15-38\AD3-10-20.M
Last changed : 15/8/2022 10:21:32 pm by SYSTEM
Analysis Method : C:\Users\Public\Documents\ChemStation\1\Data\SUN\SUN 2024-05-07 18-15-38\AD3-10-20.M (Sequence Method)
Last changed : 10/7/2024 10:22:16 am by SYSTEM
(modified after loading)
Additional Info : Peak(s) manually integrated

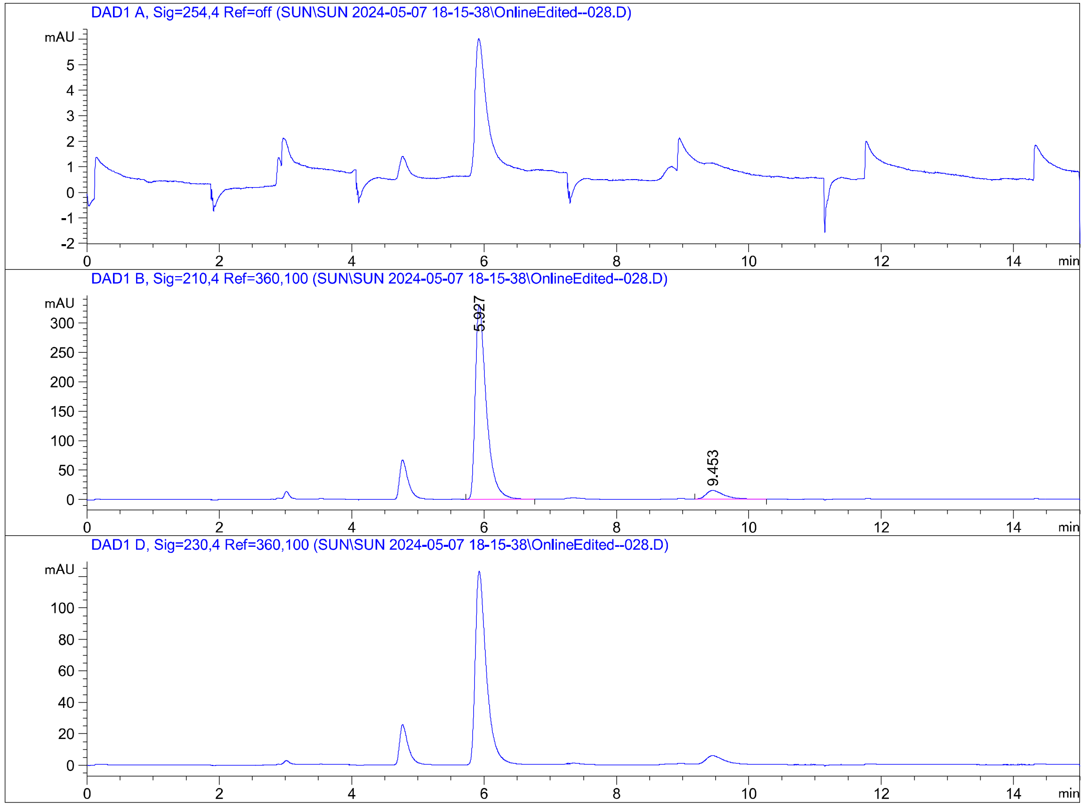

Data File C:\Users\P...\ChemStation\1\Data\SUN\SUN 2024-05-07 18-15-38\OnlineEdited--028.D
Sample Name: yangll-9-123-opt

===

Area Percent Report

===

Sorted By : Signal
Multiplier : 1.0000
Dilution : 1.0000
Use Multiplier & Dilution Factor with ISTDs

Signal 1: DAD1 A, Sig=254,4 Ref=off

Signal 2: DAD1 B, Sig=210,4 Ref=360,100

Peak #	RetTime [min]	Type	Width [min]	Area [mAU*s]	Height [mAU]	Area %
1	5.927	BB	0.1715	3835.53174	330.27234	93.4187
2	9.453	BB	0.2584	270.21271	14.46591	6.5813

Totals : 4105.74445 344.73825

Signal 3: DAD1 D, Sig=230,4 Ref=360,100

===

*** End of Report ***

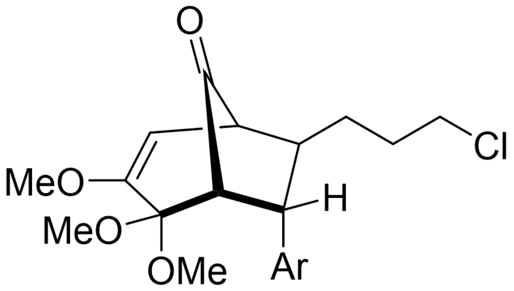

Ar = 4-$CH_3C_6H_4$

4w

opt

Data File C:\Users\P...\Data\SUN\SUN 2024-06-03 20-11-16\002-P1-A1-yangll-9-167-2-rac-IB.D
Sample Name: yangll-9-167-2-rac-IB

===

Acq. Operator	: SYSTEM	Seq. Line :	2
Acq. Instrument	: LC1260	Location :	P1-A-01
Injection Date	: 6/3/2024 8:24:11 PM	Inj :	1
		Inj Volume :	5.000 µl

Different Inj Volume from Sample Entry! Actual Inj Volume : 4.000 µl
Acq. Method : C:\Users\Public\Documents\ChemStation\1\Data\SUN\SUN 2024-06-03 20-11-16\IBN3-20-40.M
Last changed : 6/3/2024 8:37:43 PM by SYSTEM (modified after loading)
Analysis Method : C:\Users\Public\Documents\ChemStation\1\Data\SUN\SUN 2024-06-03 20-11-16\IBN3-20-40.M (Sequence Method)
Last changed : 6/3/2024 10:46:02 PM by SYSTEM (modified after loading)
Additional Info : Peak(s) manually integrated

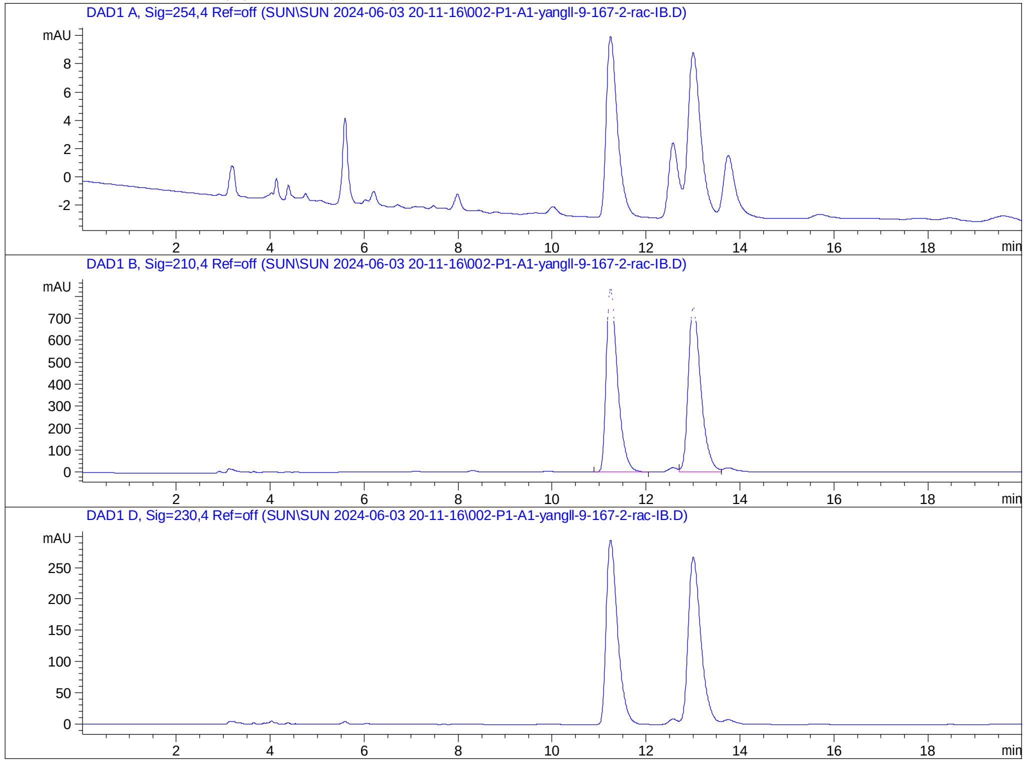

Data File C:\Users\P...\Data\SUN\SUN 2024-06-03 20-11-16\002-P1-A1-yangll-9-167-2-rac-IB.D
Sample Name: yangll-9-167-2-rac-IB

===

Area Percent Report

===

Sorted By : Signal
Multiplier : 1.0000
Dilution : 1.0000
Use Multiplier & Dilution Factor with ISTDs

Signal 1: DAD1 A, Sig=254,4 Ref=off

Signal 2: DAD1 B, Sig=210,4 Ref=off

Peak #	RetTime [min]	Type	Width [min]	Area [mAU*s]	Height [mAU]	Area %
1	11.243	MM R	0.2817	1.41160e4	835.07404	50.1431
2	13.006	MM R	0.3117	1.40354e4	750.35895	49.8569
Totals :				2.81515e4	1585.43298	

Signal 3: DAD1 D, Sig=230,4 Ref=off

===

*** End of Report ***

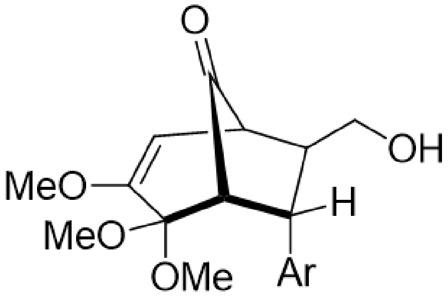

Ar = 4-$CH_3C_6H_4$

4x

rac

Data File C:\Users\P...n\1\Data\SUN\SUN 2024-06-03 22-27-34\002-P1-A2-yangll-9-167-2-opt.D
Sample Name: yangll-9-167-2-opt

===

Acq. Operator : SYSTEM Seq. Line : 2
Acq. Instrument : LC1260 Location : P1-A-02
Injection Date : 6/3/2024 10:40:23 PM Inj : 1
Inj Volume : 5.000 µl
Different Inj Volume from Sample Entry! Actual Inj Volume : 4.000 µl
Acq. Method : C:\Users\Public\Documents\ChemStation\1\Data\SUN\SUN 2024-06-03 22-27-34\IBN3-20-40.M
Last changed : 6/3/2024 11:02:08 PM by SYSTEM (modified after loading)
Analysis Method : C:\Users\Public\Documents\ChemStation\1\Data\SUN\SUN 2024-06-03 22-27-34\IBN3-20-40.M (Sequence Method)
Last changed : 6/3/2024 11:04:00 PM by SYSTEM (modified after loading)
Additional Info : Peak(s) manually integrated

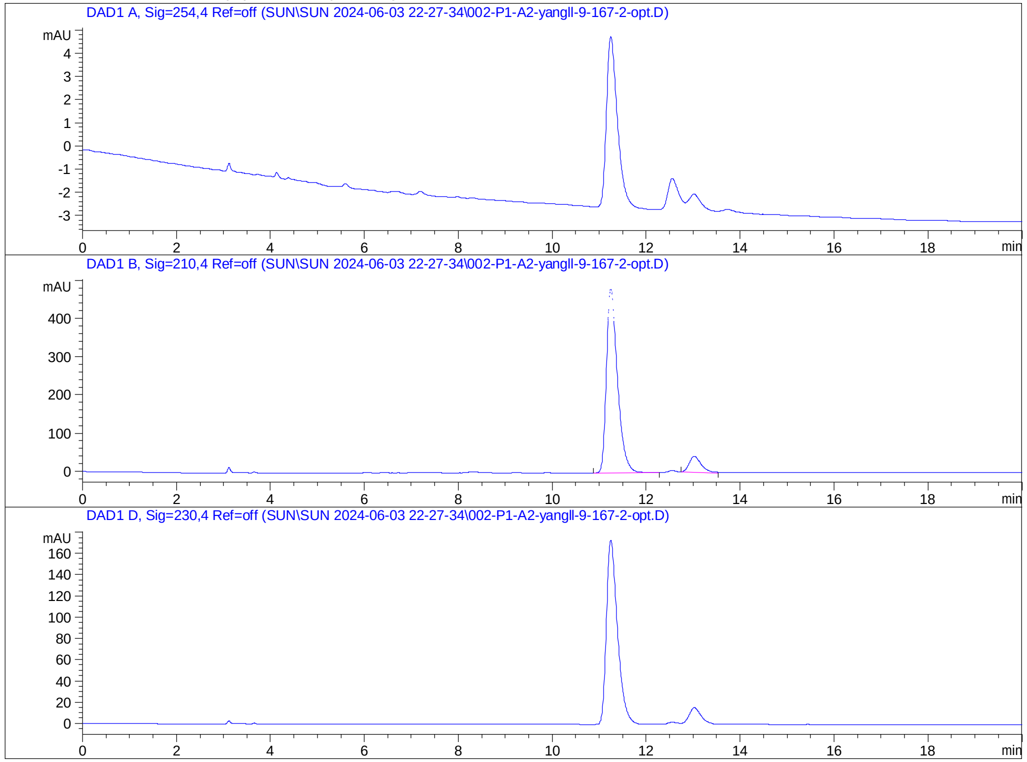

Data File C:\Users\P...n\1\Data\SUN\SUN 2024-06-03 22-27-34\002-P1-A2-yangll-9-167-2-opt.D
Sample Name: yangll-9-167-2-opt

===
Area Percent Report
===

Sorted By : Signal
Multiplier : 1.0000
Dilution : 1.0000
Use Multiplier & Dilution Factor with ISTDs

Signal 1: DAD1 A, Sig=254,4 Ref=off

Signal 2: DAD1 B, Sig=210,4 Ref=off

Peak #	RetTime [min]	Type	Width [min]	Area [mAU*s]	Height [mAU]	Area %
1	11.246	BB	0.2494	7915.11475	482.58633	91.0191
2	13.026	MM R	0.3051	780.99054	42.66690	8.9809

Totals : 8696.10529 525.25324

Signal 3: DAD1 D, Sig=230,4 Ref=off

===
*** End of Report ***

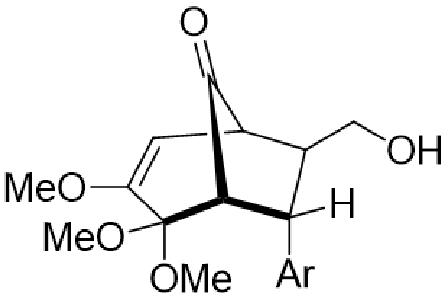

Ar = 4-$CH_3C_6H_4$

4x

opt

===

```
Acq. Operator   : SYSTEM                        Seq. Line :   2
Acq. Instrument : LC1260                         Location :   P1-A-01
Injection Date  : 4/16/2024 9:12:15 PM                Inj :   1
                                               Inj Volume : 5.000 µl
Different Inj Volume from Sample Entry!  Actual Inj Volume : 10.000 µl
Method          : C:\Users\Public\Documents\ChemStation\1\Data\SUN\SUN 2024-04-16 21-00-02
                  \IBN3-30-30.M (Sequence Method)
Last changed    : 4/8/2024 3:14:14 PM by SYSTEM
Additional Info : Peak(s) manually integrated
```

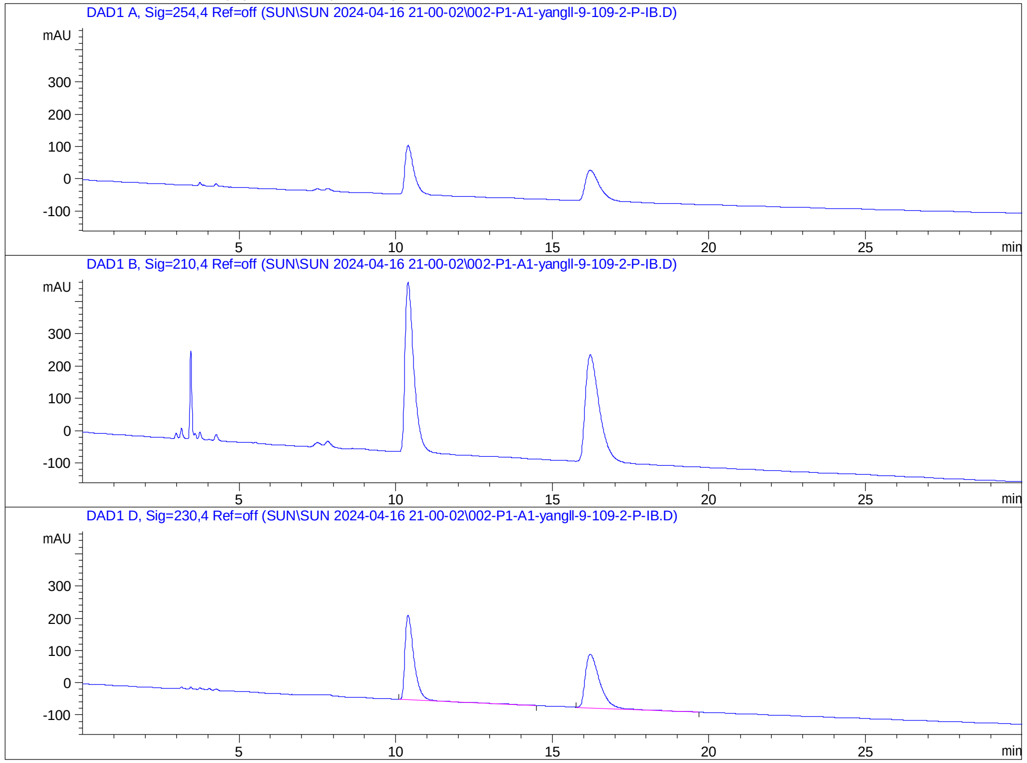

===
Area Percent Report
===

```
Sorted By             :      Signal
Multiplier            :      1.0000
Dilution              :      1.0000
Use Multiplier & Dilution Factor with ISTDs
```


Data File C:\Users\P...\1\Data\SUN\SUN 2024-04-16 21-00-02\002-P1-A1-yangll-9-109-2-P-IB.D
Sample Name: yangll-9-109-2-P-IB

Signal 1: DAD1 A, Sig=254,4 Ref=off

Signal 2: DAD1 B, Sig=210,4 Ref=off

Signal 3: DAD1 D, Sig=230,4 Ref=off

Peak #	RetTime [min]	Type	Width [min]	Area [mAU*s]	Height [mAU]	Area %
1	10.396	BB	0.3045	5275.02393	262.40845	50.0486
2	16.211	BBA	0.4833	5264.77832	166.33392	49.9514
Totals :				1.05398e4	428.74237	

===
*** End of Report ***

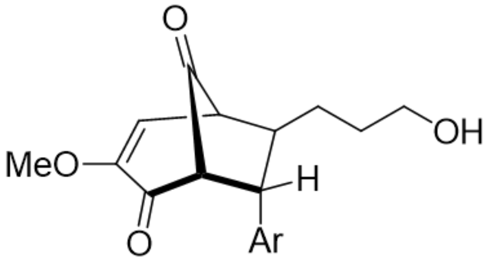

Ar = 4-$CH_3C_6H_4$

4y'

rac

===

Acq. Operator : SYSTEM   Seq. Line : 2
Acq. Instrument : LC1260   Location : P1-A-03
Injection Date : 5/31/2024 10:19:51 AM   Inj : 1
Inj Volume : 5.000 µl
Different Inj Volume from Sample Entry! Actual Inj Volume : 1.000 µl
Acq. Method : C:\Users\Public\Documents\ChemStation\1\Data\SUN\SUN 2024-05-31 10-07-05 \IBN3-30-30.M
Last changed : 4/8/2024 3:14:14 PM by SYSTEM
Analysis Method : C:\Users\Public\Documents\ChemStation\1\Data\SUN\SUN 2024-05-31 10-07-05 \IBN3-30-30.M (Sequence Method)
Last changed : 6/4/2024 11:01:26 AM by SYSTEM
(modified after loading)
Additional Info : Peak(s) manually integrated

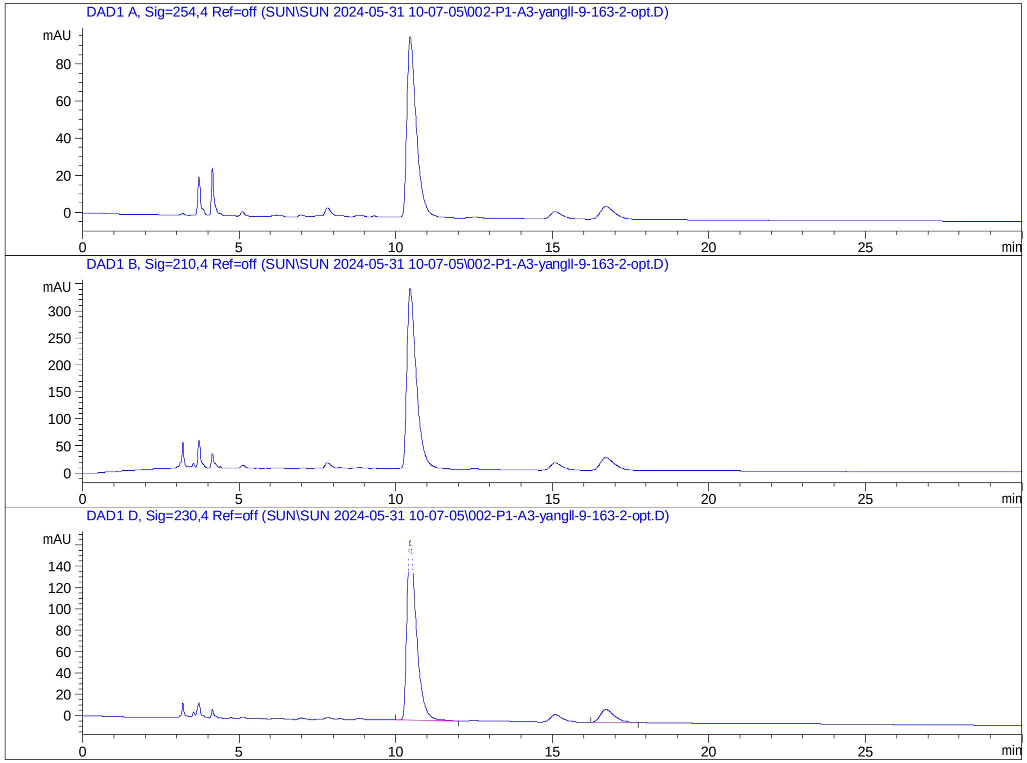

===

Area Percent Report

===

Sorted By : Signal
Multiplier : 1.0000
Dilution : 1.0000
Use Multiplier & Dilution Factor with ISTDs

Data File C:\Users\P...n\1\Data\SUN\SUN 2024-05-31 10-07-05\002-P1-A3-yangll-9-163-2-opt.D
Sample Name: yangll-9-163-2-opt

Signal 1: DAD1 A, Sig=254,4 Ref=off

Signal 2: DAD1 B, Sig=210,4 Ref=off

Signal 3: DAD1 D, Sig=230,4 Ref=off

Peak #	RetTime [min]	Type	Width [min]	Area [mAU*s]	Height [mAU]	Area %
1	10.462	BB	0.3206	3593.65625	168.63922	90.3724
2	16.714	MM R	0.5339	382.84116	11.95151	9.6276

Totals : 3976.49741 180.59073

===

*** End of Report ***

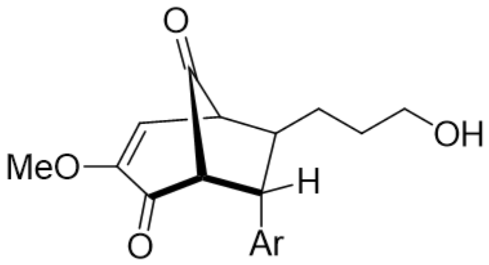

Ar = 4-$CH_3C_6H_4$

4y'

opt

===

Acq. Operator : SYSTEM Seq. Line : 2
Sample Operator : SYSTEM
Acq. Instrument : HPLC Location : P1-A-01
Injection Date : 4/11/2023 12:30:27 pm Inj : 1
Inj Volume : 2.000 µl
Different Inj Volume from Sample Entry! Actual Inj Volume : 5.000 µl
Acq. Method : C:\Users\Public\Documents\ChemStation\1\Data\SUN\SUN 2023-11-04 12-17-11\ID3-10-20.M
Last changed : 2/8/2023 12:18:49 pm by SYSTEM
Analysis Method : C:\Users\Public\Documents\ChemStation\1\Data\SUN\SUN 2023-11-04 12-17-11\ID3-10-20.M (Sequence Method)
Last changed : 8/5/2024 10:26:21 pm by SYSTEM
(modified after loading)
Additional Info : Peak(s) manually integrated

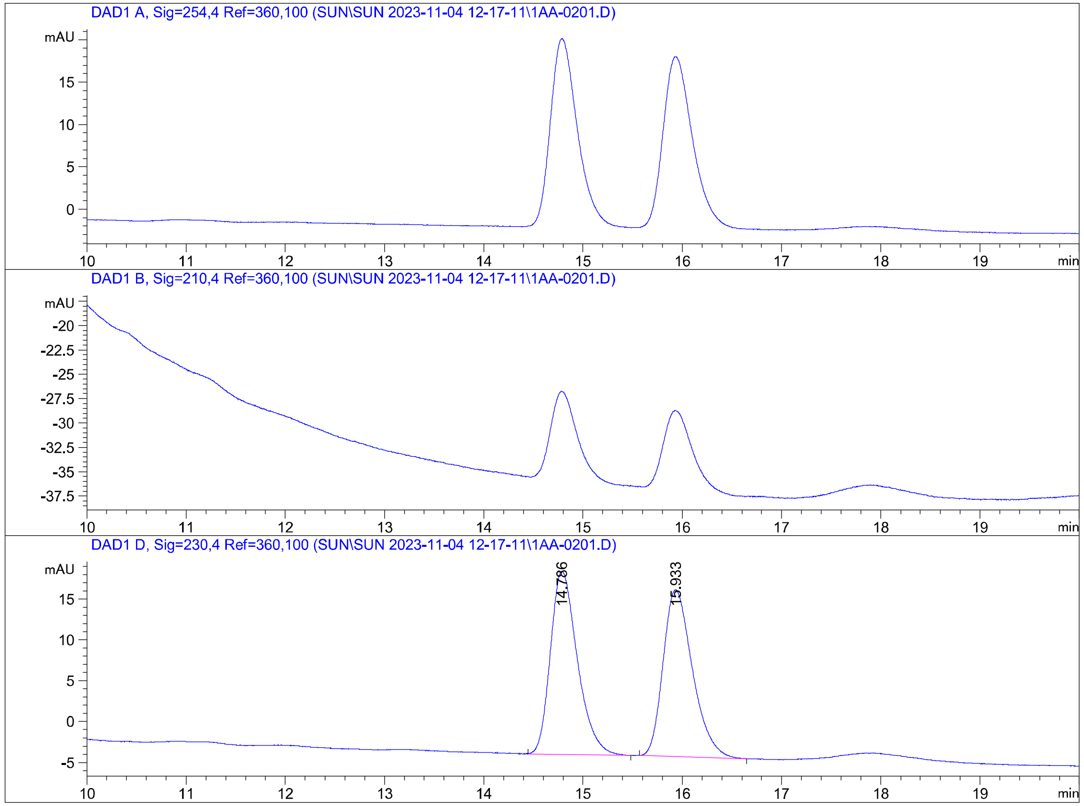

Data File C:\Users\P...Documents\ChemStation\1\Data\SUN\SUN 2023-11-04 12-17-11\1AA-0201.D
Sample Name: yangll-8-32-1a

===

Area Percent Report

===

Sorted By : Signal
Multiplier : 1.0000
Dilution : 1.0000
Use Multiplier & Dilution Factor with ISTDs

Signal 1: DAD1 A, Sig=254,4 Ref=360,100

Signal 2: DAD1 B, Sig=210,4 Ref=360,100

Signal 3: DAD1 D, Sig=230,4 Ref=360,100

Peak #	RetTime [min]	Type	Width [min]	Area [mAU*s]	Height [mAU]	Area %
1	14.786	BB	0.2704	424.11368	22.44680	50.1876
2	15.933	BB	0.2848	420.94312	20.43360	49.8124
Totals :				845.05679	42.88040	

===

*** End of Report ***

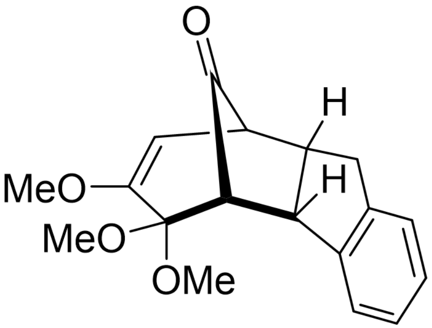

4z

rac

===

Acq. Operator : SYSTEM Seq. Line : 39
Sample Operator : SYSTEM
Acq. Instrument : HPLC Location : P1-A-07
Injection Date : 12/11/2023 9:39:44 am Inj : 1
Inj Volume : 2.000 µl
Acq. Method : C:\Users\Public\Documents\ChemStation\1\Data\SUN\SUN 2023-11-11 18-12-21\ID3-10-20.M
Last changed : 2/8/2023 12:18:49 pm by SYSTEM
Analysis Method : C:\Users\Public\Documents\ChemStation\1\Data\SUN\SUN 2023-11-11 18-12-21\ID3-10-20.M (Sequence Method)
Last changed : 8/5/2024 10:25:05 pm by SYSTEM
Additional Info : Peak(s) manually integrated

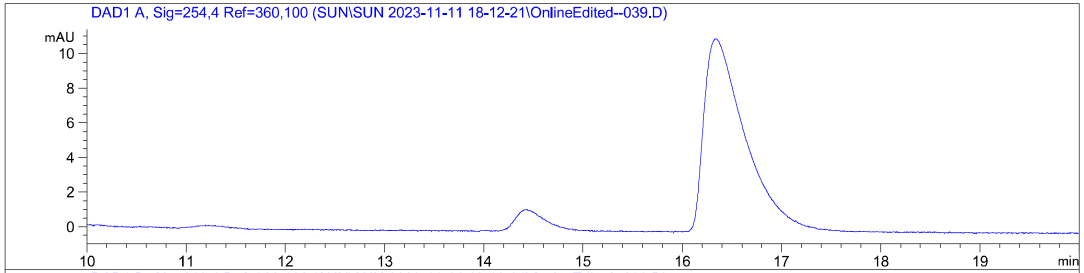

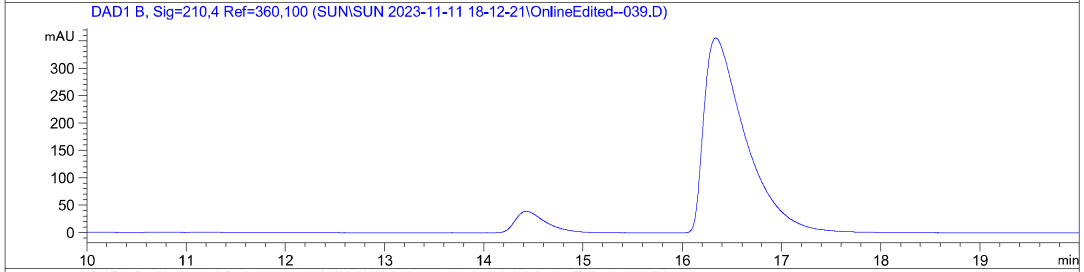

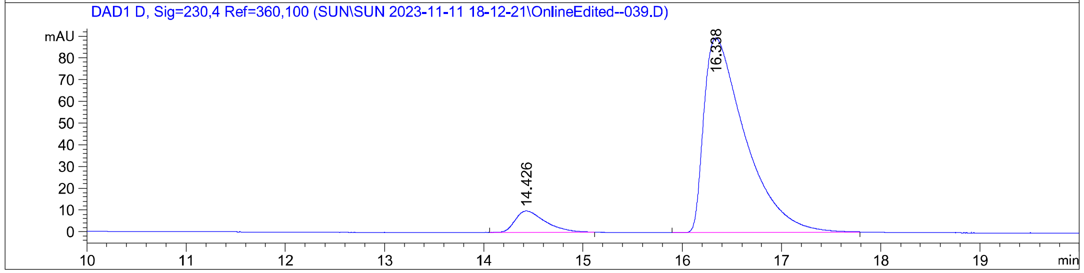

===
Area Percent Report
===

Sorted By : Signal
Multiplier : 1.0000
Dilution : 1.0000
Use Multiplier & Dilution Factor with ISTDs

Data File C:\Users\P...\ChemStation\1\Data\SUN\SUN 2023-11-11 18-12-21\OnlineEdited--039.D
Sample Name: yangll-8-53-3

Signal 1: DAD1 A, Sig=254,4 Ref=360,100

Signal 2: DAD1 B, Sig=210,4 Ref=360,100

Signal 3: DAD1 D, Sig=230,4 Ref=360,100

Peak #	RetTime [min]	Type	Width [min]	Area [mAU*s]	Height [mAU]	Area %
1	14.426	MM R	0.3569	211.85413	9.89458	7.5536
2	16.338	MM R	0.4831	2592.81665	89.44315	92.4464

Totals : 2804.67078 99.33773

===
*** End of Report ***

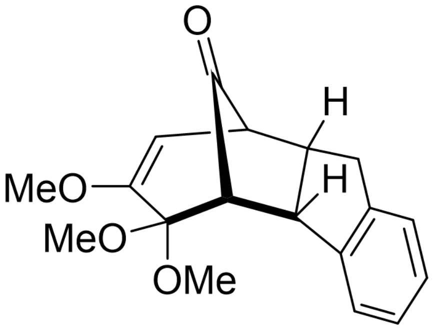

4z

opt

===

Acq. Operator : SYSTEM   Seq. Line :   9
Sample Operator : SYSTEM
Acq. Instrument : HPLC   Location :   P1-F-02
Injection Date : 3/2/2024 6:23:24 pm   Inj :   1
Inj Volume : 2.000 µl
Different Inj Volume from Sample Entry! Actual Inj Volume : 4.000 µl
Method : C:\Users\Public\Documents\ChemStation\1\Data\SUN\SUN 2024-02-03 16-03-18\IBN3-10-20.M (Sequence Method)
Last changed : 30/7/2023 1:22:19 pm by SYSTEM
Additional Info : Peak(s) manually integrated

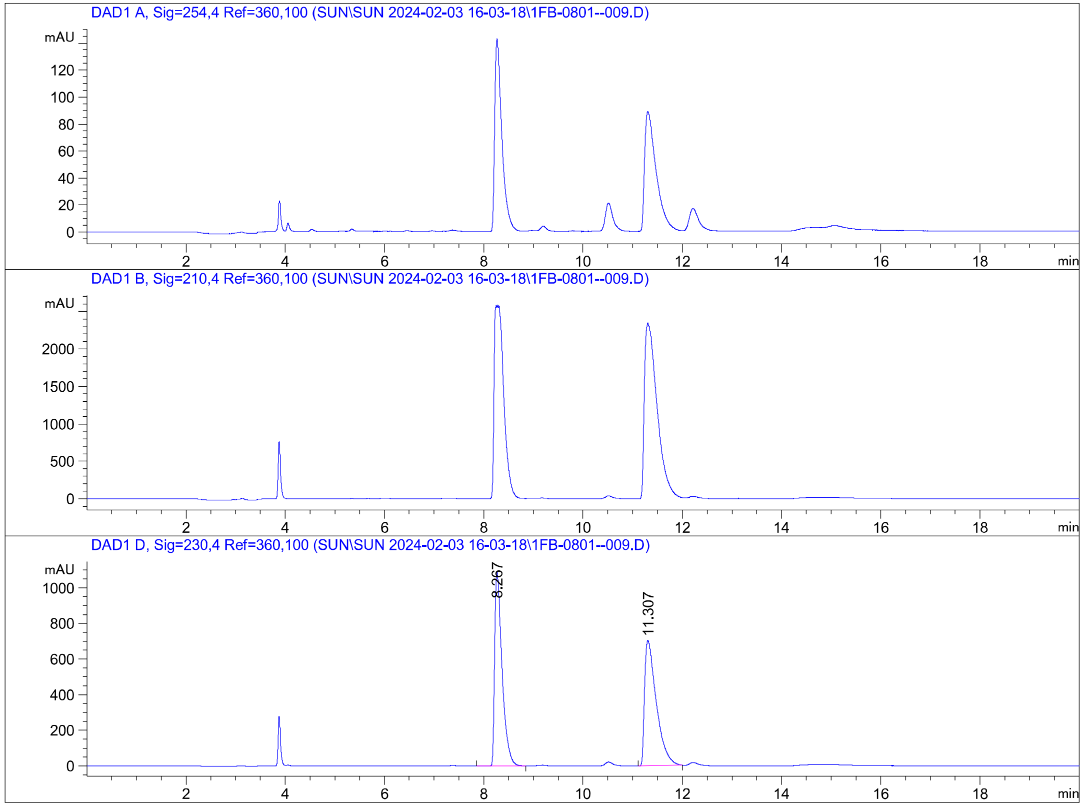

===
Area Percent Report
===

Sorted By : Signal
Multiplier : 1.0000
Dilution : 1.0000
Use Multiplier & Dilution Factor with ISTDs

Data File C:\Users\P...ents\ChemStation\1\Data\SUN\SUN 2024-02-03 16-03-18\1FB-0801--009.D
Sample Name: yangll-9-40-rac-IB

Signal 1: DAD1 A, Sig=254,4 Ref=360,100

Signal 2: DAD1 B, Sig=210,4 Ref=360,100

Signal 3: DAD1 D, Sig=230,4 Ref=360,100

Peak #	RetTime [min]	Type	Width [min]	Area [mAU*s]	Height [mAU]	Area %
1	8.267	VB R	0.1583	1.13534e4	1092.34583	49.6825
2	11.307	MM R	0.2728	1.14985e4	702.50079	50.3175

Totals : 2.28519e4 1794.84662

===
*** End of Report ***

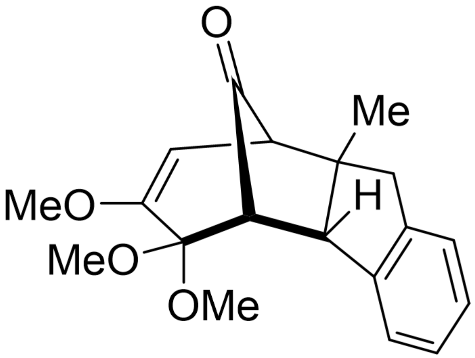

4aa

rac

===

Acq. Operator	: SYSTEM	Seq. Line :	2
Sample Operator	: SYSTEM		
Acq. Instrument	: HPLC	Location :	P1-E-01
Injection Date	: 4/2/2024 1:59:22 pm	Inj :	1
		Inj Volume :	2.000 µl

Method : C:\Users\Public\Documents\ChemStation\1\Data\SUN\SUN 2024-02-04 13-46-27\IBN3-10-20.M (Sequence Method)
Last changed : 30/7/2023 1:22:19 pm by SYSTEM
Additional Info : Peak(s) manually integrated

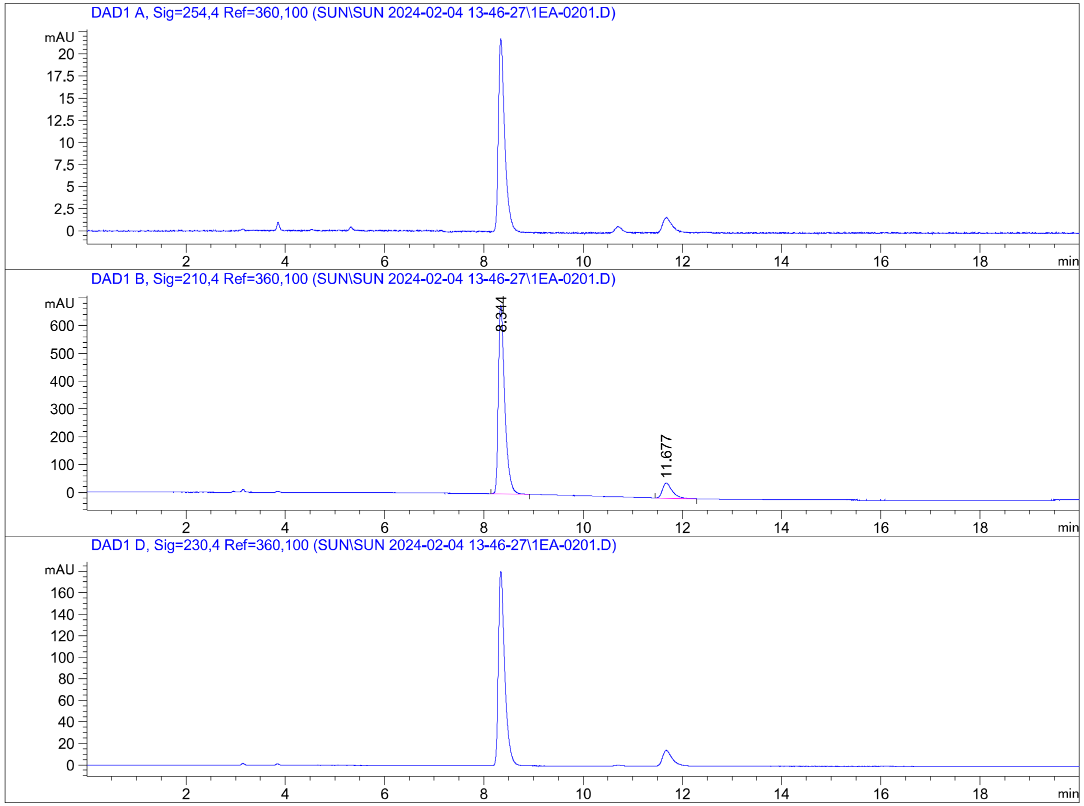

===
Area Percent Report
===

Sorted By : Signal
Multiplier : 1.0000
Dilution : 1.0000
Use Multiplier & Dilution Factor with ISTDs

Data File C:\Users\P...Documents\ChemStation\1\Data\SUN\SUN 2024-02-04 13-46-27\1EA-0201.D
Sample Name: yangll-9-40-opt

Signal 1: DAD1 A, Sig=254,4 Ref=360,100

Signal 2: DAD1 B, Sig=210,4 Ref=360,100

Peak #	RetTime [min]	Type	Width [min]	Area [mAU*s]	Height [mAU]	Area %
1	8.344	BV R	0.1346	6057.18115	678.65936	89.2216
2	11.677	BV R	0.1922	731.73962	54.46878	10.7784
Totals :				6788.92078	733.12814	

Signal 3: DAD1 D, Sig=230,4 Ref=360,100

===
*** End of Report ***

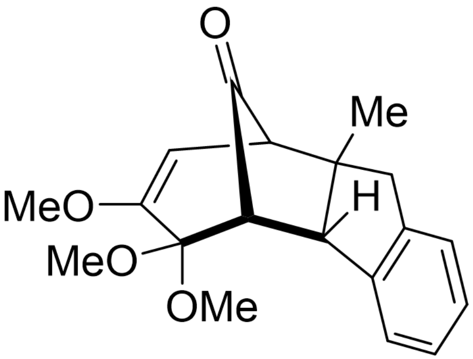

4aa

opt

===

Acq. Operator : SYSTEM Seq. Line : 52
Sample Operator : SYSTEM
Acq. Instrument : HPLC Location : P1-A-01
Injection Date : 1/12/2023 4:12:23 pm Inj : 1
Inj Volume : 2.000 µl
Acq. Method : C:\Users\Public\Documents\ChemStation\1\Data\SUN\SUN 2023-11-30 18-47-28\AD3-10-15.M
Last changed : 28/12/2022 10:29:24 pm by SYSTEM
Analysis Method : C:\Users\Public\Documents\ChemStation\1\Data\SUN\SUN 2023-11-30 18-47-28\AD3-10-15.M (Sequence Method)
Last changed : 10/8/2024 8:15:49 pm by SYSTEM
(modified after loading)
Additional Info : Peak(s) manually integrated

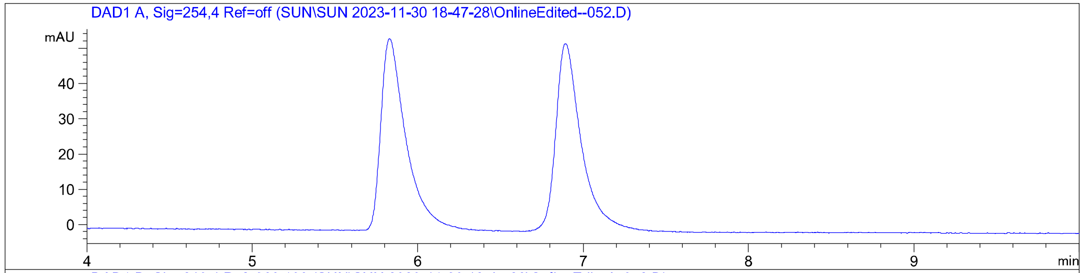

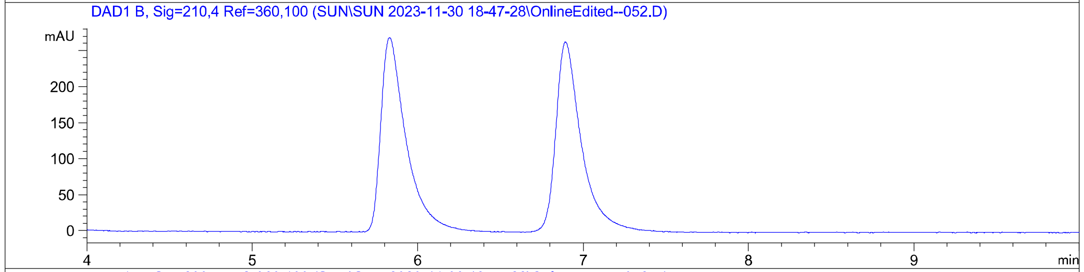

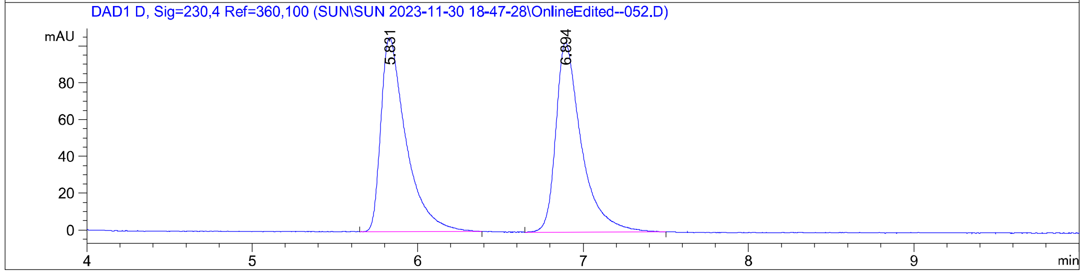

===
Area Percent Report
===

Sorted By : Signal
Multiplier : 1.0000
Dilution : 1.0000
Use Multiplier & Dilution Factor with ISTDs

Data File C:\Users\P...\ChemStation\1\Data\SUN\SUN 2023-11-30 18-47-28\OnlineEdited--052.D
Sample Name: yangll-8-94-rac-AD3

Signal 1: DAD1 A, Sig=254,4 Ref=off

Signal 2: DAD1 B, Sig=210,4 Ref=360,100

Signal 3: DAD1 D, Sig=230,4 Ref=360,100

Peak #	RetTime [min]	Type	Width [min]	Area [mAU*s]	Height [mAU]	Area %
1	5.831	VV R	0.1515	1115.30774	105.37798	50.1098
2	6.894	BV R	0.1496	1110.41821	103.16044	49.8902

Totals : 2225.72595 208.53841

===
*** End of Report ***

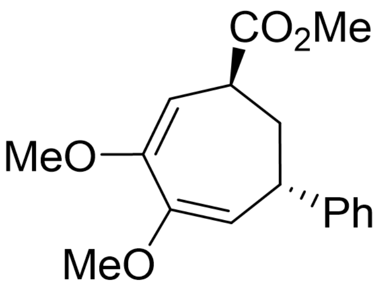

5a

rac

===

Acq. Operator : SYSTEM Seq. Line : 8
Sample Operator : SYSTEM
Acq. Instrument : HPLC Location : P1-A-01
Injection Date : 11/12/2024 8:28:27 pm Inj : 1
Inj Volume : 2.000 µl
Different Inj Volume from Sample Entry! Actual Inj Volume : 5.000 µl
Acq. Method : C:\Users\Public\Documents\ChemStation\1\Data\SUN\SUN 2024-12-11 18-18-10\AD3-10-15.M
Last changed : 28/12/2022 10:29:24 pm by SYSTEM
Analysis Method : C:\Users\Public\Documents\ChemStation\1\Data\SUN\SUN 2024-12-11 18-18-10\AD3-10-15.M (Sequence Method)
Last changed : 11/12/2024 9:19:39 pm by SYSTEM
(modified after loading)
Additional Info : Peak(s) manually integrated

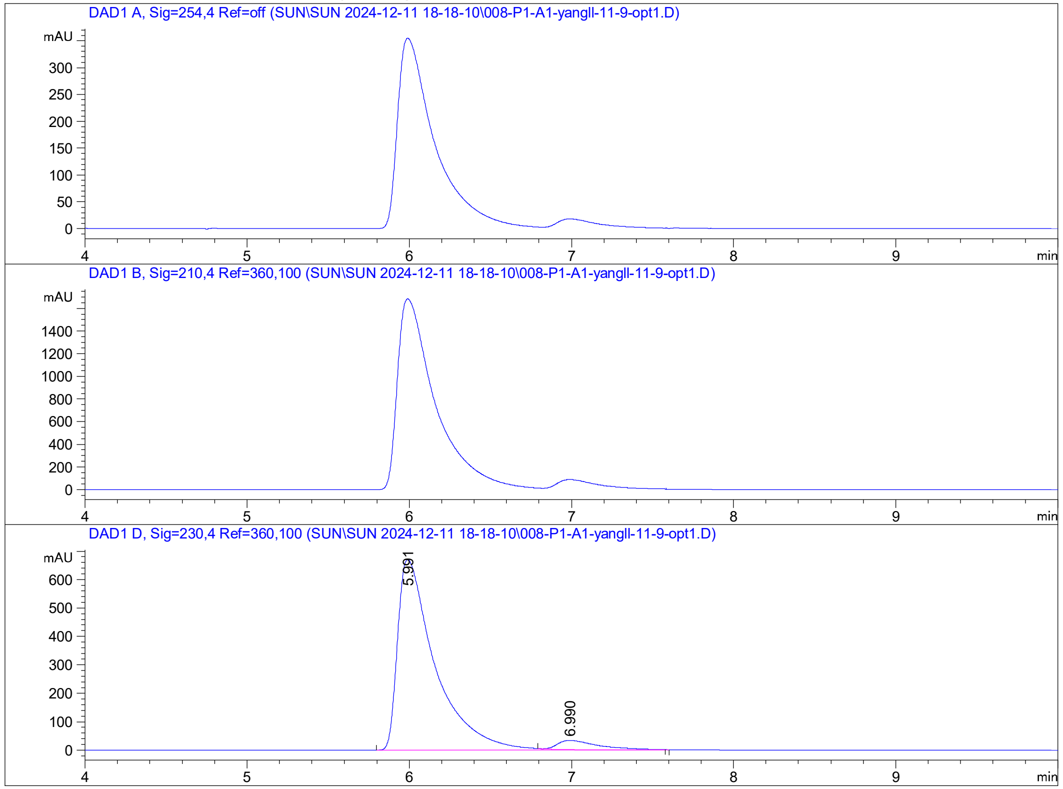

Data File C:\Users\P...ion\1\Data\SUN\SUN 2024-12-11 18-18-10\008-P1-A1-yangll-11-9-opt1.D
Sample Name: yangll-11-9-opt1

===

Area Percent Report

===

Sorted By : Signal
Multiplier : 1.0000
Dilution : 1.0000
Use Multiplier & Dilution Factor with ISTDs

Signal 1: DAD1 A, Sig=254,4 Ref=off

Signal 2: DAD1 B, Sig=210,4 Ref=360,100

Signal 3: DAD1 D, Sig=230,4 Ref=360,100

Peak #	RetTime [min]	Type	Width [min]	Area [mAU*s]	Height [mAU]	Area %
1	5.991	BV R	0.2338	1.07392e4	670.85266	95.0494
2	6.990	VV E	0.2412	559.35077	32.10987	4.9506

Totals : 1.12986e4 702.96254

===

*** End of Report ***

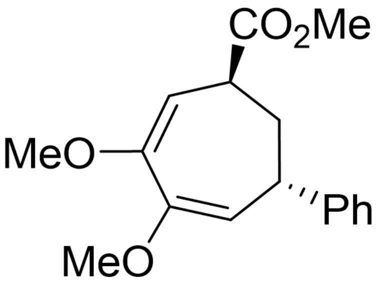

5a

opt

===

Acq. Operator : SYSTEM Seq. Line : 59
Sample Operator : SYSTEM
Acq. Instrument : HPLC Location : P1-A-06
Injection Date : 10/1/2024 10:11:13 am Inj : 1
Inj Volume : 2.000 µl
Acq. Method : C:\Users\Public\Documents\ChemStation\1\Data\SUN\SUN 2024-01-09 09-29-51
\AD3-10-30.M
Last changed : 24/10/2022 9:57:21 am by SYSTEM
Analysis Method : C:\Users\Public\Documents\ChemStation\1\Data\SUN\SUN 2024-01-09 09-29-51
\AD3-10-30.M (Sequence Method)
Last changed : 10/8/2024 7:39:24 pm by SYSTEM
(modified after loading)
Additional Info : Peak(s) manually integrated

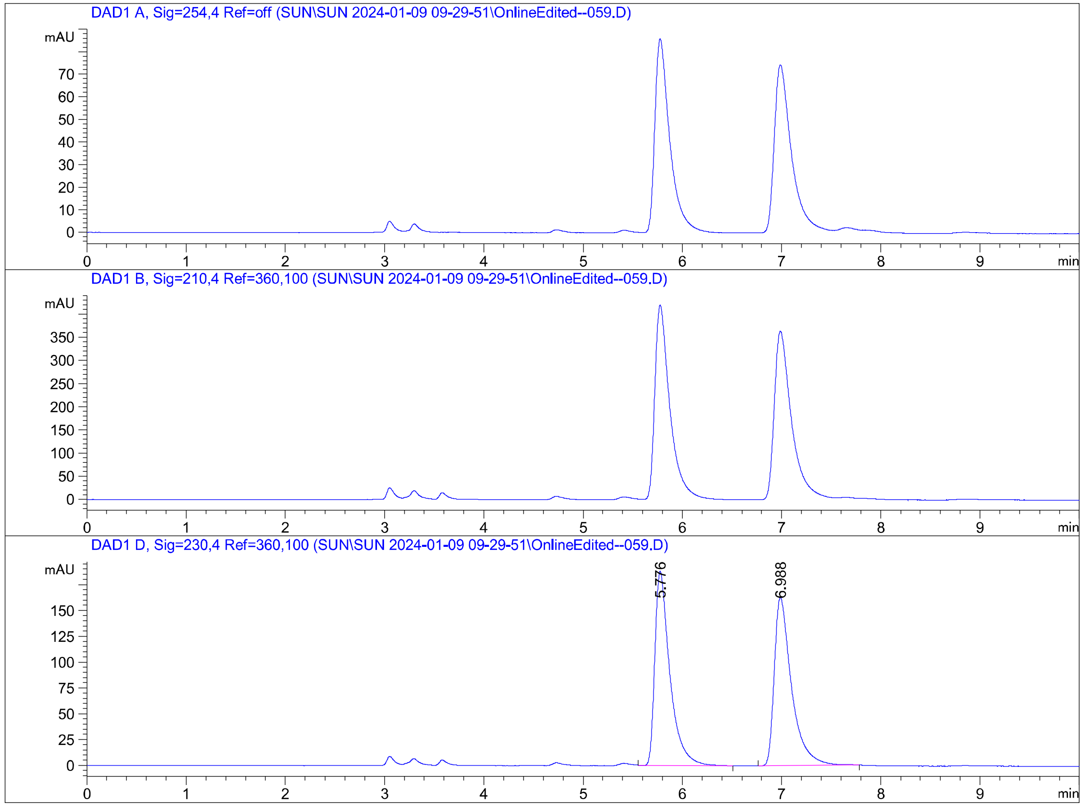

===
Area Percent Report
===

Sorted By : Signal
Multiplier : 1.0000
Dilution : 1.0000
Use Multiplier & Dilution Factor with ISTDs

Data File C:\Users\P...\ChemStation\1\Data\SUN\SUN 2024-01-09 09-29-51\OnlineEdited--059.D
Sample Name: yangll-8-173-1-rac

Signal 1: DAD1 A, Sig=254,4 Ref=off

Signal 2: DAD1 B, Sig=210,4 Ref=360,100

Signal 3: DAD1 D, Sig=230,4 Ref=360,100

Peak #	RetTime [min]	Type	Width [min]	Area [mAU*s]	Height [mAU]	Area %
1	5.776	MM R	0.1757	1983.32556	188.18565	50.1054
2	6.988	BV R	0.1789	1974.98425	162.94347	49.8946

Totals : 3958.30981 351.12912

===
*** End of Report ***

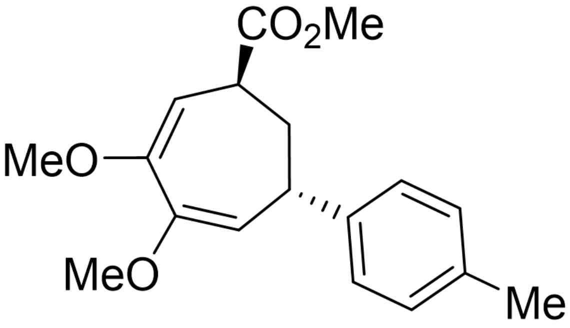

5b

rac

===

Acq. Operator : SYSTEM Seq. Line : 2
Sample Operator : SYSTEM
Acq. Instrument : HPLC Location : P1-F-03
Injection Date : 10/8/2024 9:07:46 am Inj : 1
Inj Volume : 2.000 µl
Acq. Method : C:\Users\Public\Documents\ChemStation\1\Data\SUN\SUN 2024-08-10 08-54-37\AD3-10-15.M
Last changed : 28/12/2022 10:29:24 pm by SYSTEM
Analysis Method : C:\Users\Public\Documents\ChemStation\1\Data\SUN\SUN 2024-08-10 08-54-37\AD3-10-15.M (Sequence Method)
Last changed : 10/8/2024 7:38:17 pm by SYSTEM
Additional Info : Peak(s) manually integrated

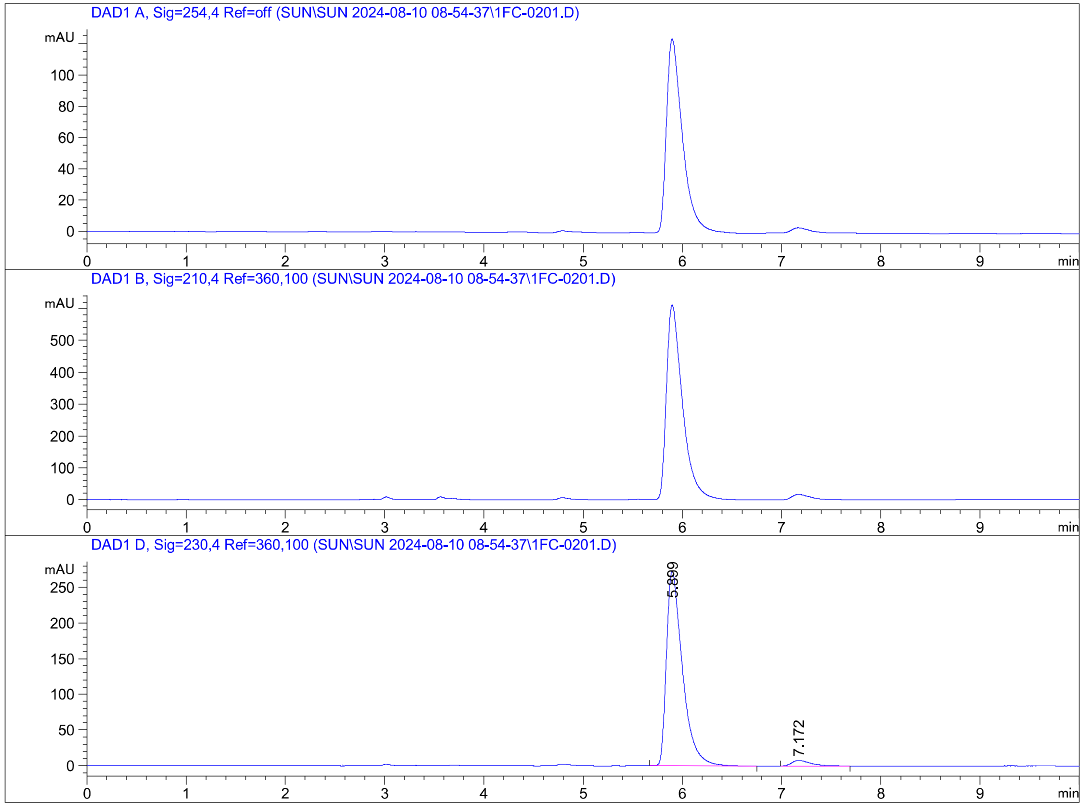

===
Area Percent Report
===

Sorted By : Signal
Multiplier : 1.0000
Dilution : 1.0000
Use Multiplier & Dilution Factor with ISTDs

Data File C:\Users\P...Documents\ChemStation\1\Data\SUN\SUN 2024-08-10 08-54-37\1FC-0201.D
Sample Name: yangll-10-64-opt

Signal 1: DAD1 A, Sig=254,4 Ref=off

Signal 2: DAD1 B, Sig=210,4 Ref=360,100

Signal 3: DAD1 D, Sig=230,4 Ref=360,100

Peak #	RetTime [min]	Type	Width [min]	Area [mAU*s]	Height [mAU]	Area %
1	5.899	MM R	0.1871	3063.32764	272.93842	96.8077
2	7.172	MM R	0.2178	101.01421	7.72906	3.1923

Totals : 3164.34185 280.66747

===
*** End of Report ***

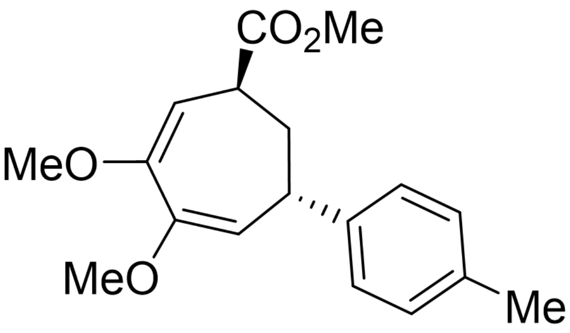

5b

opt

===

Acq. Operator : SYSTEM Seq. Line : 58
Sample Operator : SYSTEM
Acq. Instrument : HPLC Location : P1-A-05
Injection Date : 10/1/2024 9:40:19 am Inj : 1
Inj Volume : 2.000 µl
Acq. Method : C:\Users\Public\Documents\ChemStation\1\Data\SUN\SUN 2024-01-09 09-29-51\AD3-10-30.M
Last changed : 24/10/2022 9:57:21 am by SYSTEM
Analysis Method : C:\Users\Public\Documents\ChemStation\1\Data\SUN\SUN 2024-01-09 09-29-51\AD3-10-30.M (Sequence Method)
Last changed : 10/8/2024 7:44:32 pm by SYSTEM
(modified after loading)
Additional Info : Peak(s) manually integrated

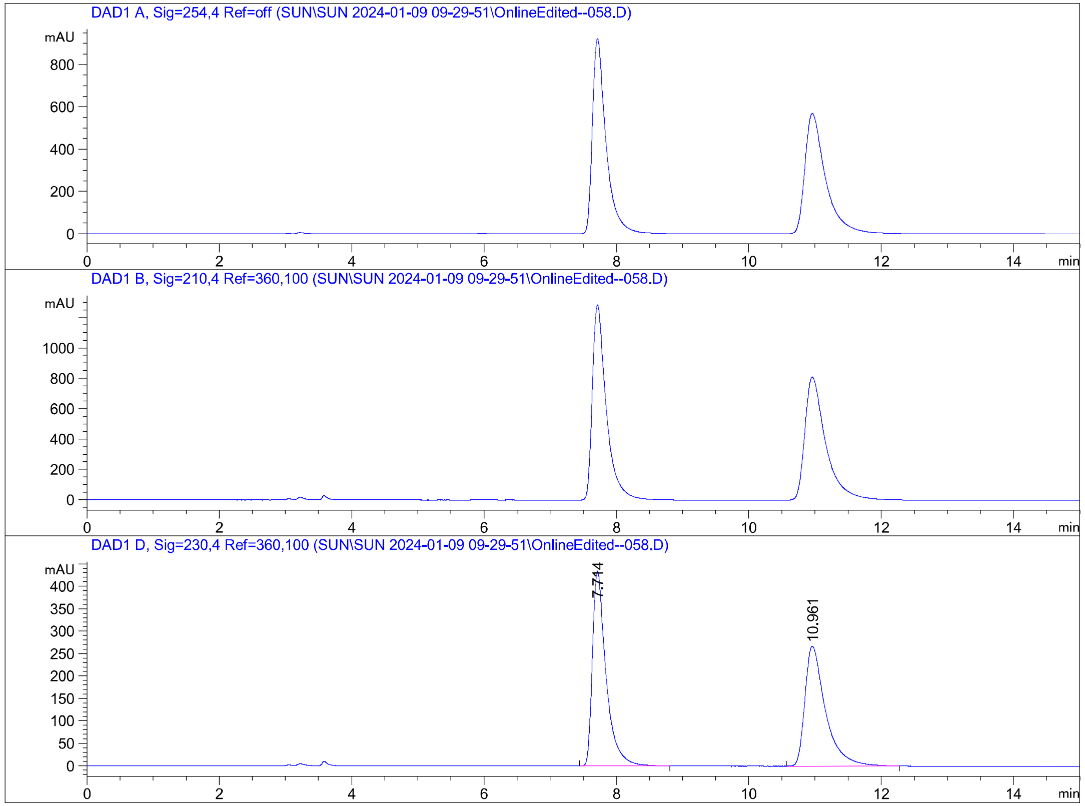

===

Area Percent Report

===

Sorted By : Signal
Multiplier : 1.0000
Dilution : 1.0000
Use Multiplier & Dilution Factor with ISTDs

Data File C:\Users\P...\ChemStation\1\Data\SUN\SUN 2024-01-09 09-29-51\OnlineEdited--058.D
Sample Name: yangll-8-172-1-rac

Signal 1: DAD1 A, Sig=254,4 Ref=off

Signal 2: DAD1 B, Sig=210,4 Ref=360,100

Signal 3: DAD1 D, Sig=230,4 Ref=360,100

Peak #	RetTime [min]	Type	Width [min]	Area [mAU*s]	Height [mAU]	Area %
1	7.714	BV R	0.2129	6210.19189	434.17889	51.8389
2	10.961	BV R	0.3153	5769.59424	267.30835	48.1611

Totals : 1.19798e4 701.48724

===

*** End of Report ***

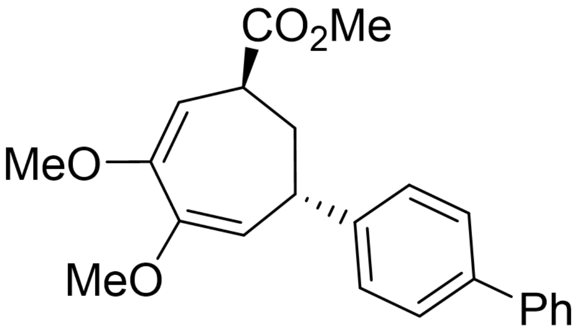

5e

rac

===

```
Acq. Operator   : SYSTEM                        Seq. Line :    3
Sample Operator : SYSTEM
Acq. Instrument : HPLC                           Location :   P1-F-04
Injection Date  : 10/8/2024 9:23:45 am                Inj :    1
                                              Inj Volume : 2.000 µl
Acq. Method     : C:\Users\Public\Documents\ChemStation\1\Data\SUN\SUN 2024-08-10 08-54-37
                  \AD3-10-15.M
Last changed    : 28/12/2022 10:29:24 pm by SYSTEM
Analysis Method : C:\Users\Public\Documents\ChemStation\1\Data\SUN\SUN 2024-08-10 08-54-37
                  \AD3-10-15.M (Sequence Method)
Last changed    : 10/8/2024 7:41:36 pm by SYSTEM
                  (modified after loading)
Additional Info : Peak(s) manually integrated
```

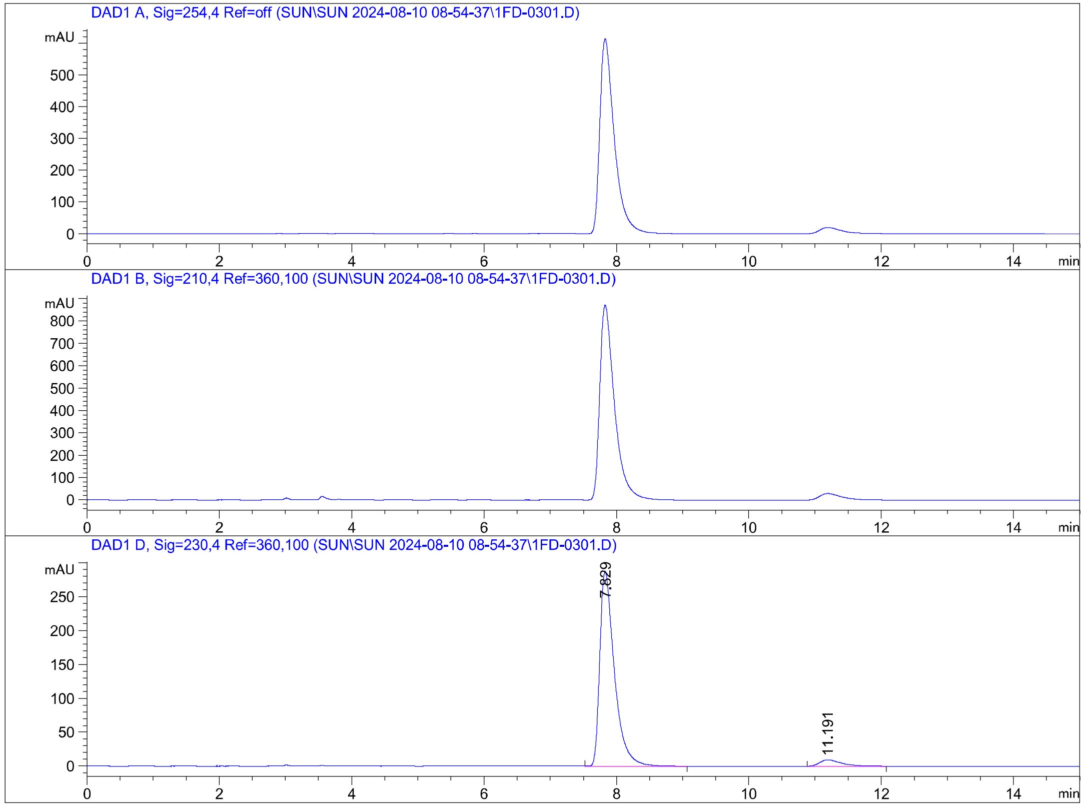

===

Area Percent Report

===

```
Sorted By             :      Signal
Multiplier            :      1.0000
Dilution              :      1.0000
Use Multiplier & Dilution Factor with ISTDs
```


Data File C:\Users\P...Documents\ChemStation\1\Data\SUN\SUN 2024-08-10 08-54-37\1FD-0301.D
Sample Name: yangll-10-66-opt

Signal 1: DAD1 A, Sig=254,4 Ref=off

Signal 2: DAD1 B, Sig=210,4 Ref=360,100

Signal 3: DAD1 D, Sig=230,4 Ref=360,100

Peak #	RetTime [min]	Type	Width [min]	Area [mAU*s]	Height [mAU]	Area %
1	7.829	MM R	0.2550	4392.30664	287.09988	95.4155
2	11.191	MM R	0.3787	211.03951	9.28823	4.5845

Totals : 4603.34615 296.38811

===

*** End of Report ***

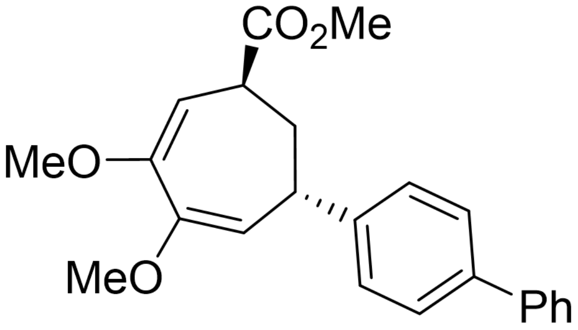

5e

opt

===

Acq. Operator : SYSTEM   Seq. Line : 16
Sample Operator : SYSTEM
Acq. Instrument : HPLC   Location : P1-A-01
Injection Date : 8/12/2023 10:14:12 pm   Inj : 1
Inj Volume : 2.000 µl
Different Inj Volume from Sample Entry! Actual Inj Volume : 1.000 µl
Acq. Method : C:\Users\Public\Documents\ChemStation\1\Data\SUN\SUN 2023-12-08 18-34-53\AD3-10-20.M
Last changed : 15/8/2022 10:21:32 pm by SYSTEM
Analysis Method : C:\Users\Public\Documents\ChemStation\1\Data\SUN\SUN 2023-12-08 18-34-53\AD3-10-20.M (Sequence Method)
Last changed : 10/8/2024 7:52:42 pm by SYSTEM
(modified after loading)
Additional Info : Peak(s) manually integrated

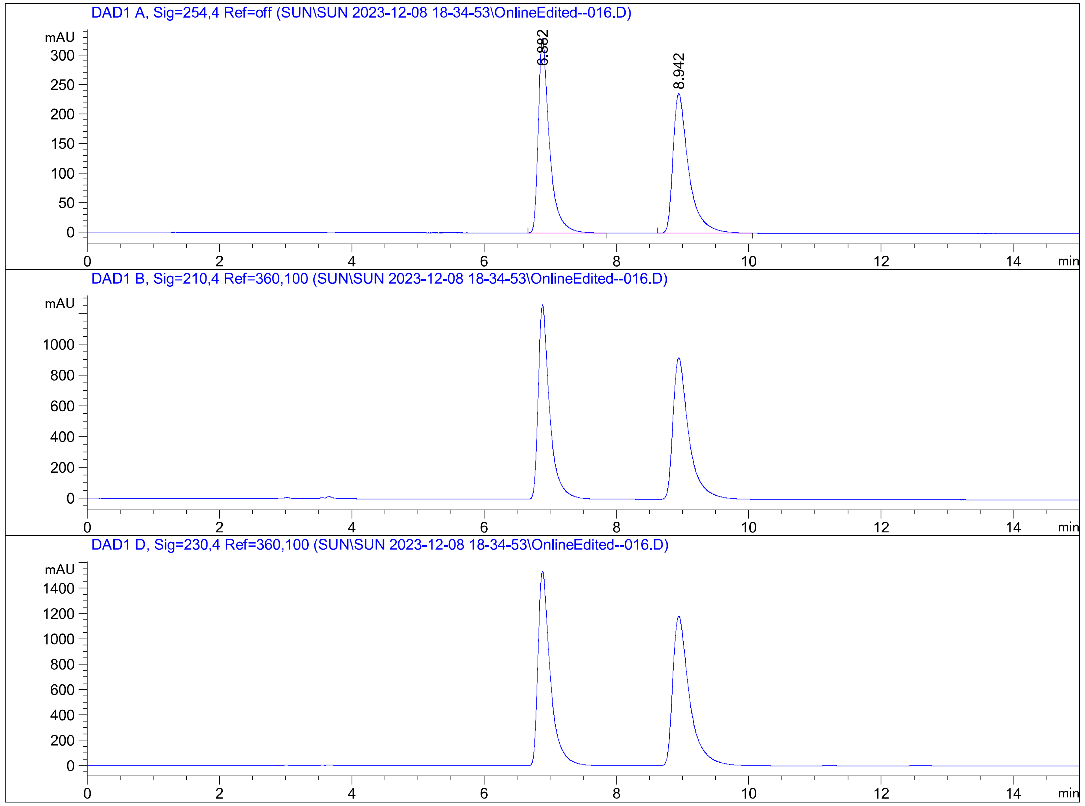

Data File C:\Users\P...\ChemStation\1\Data\SUN\SUN 2023-12-08 18-34-53\OnlineEdited--016.D
Sample Name: yangll-8-116-rac-AD

===
Area Percent Report
===

Sorted By : Signal
Multiplier : 1.0000
Dilution : 1.0000
Use Multiplier & Dilution Factor with ISTDs

Signal 1: DAD1 A, Sig=254,4 Ref=off

Peak #	RetTime [min]	Type	Width [min]	Area [mAU*s]	Height [mAU]	Area %
1	6.882	BB	0.1742	3867.92456	328.95850	50.0387
2	8.942	BB	0.2428	3861.93921	236.13535	49.9613
Totals :				7729.86377	565.09384	

Signal 2: DAD1 B, Sig=210,4 Ref=360,100

Signal 3: DAD1 D, Sig=230,4 Ref=360,100

===
*** End of Report ***

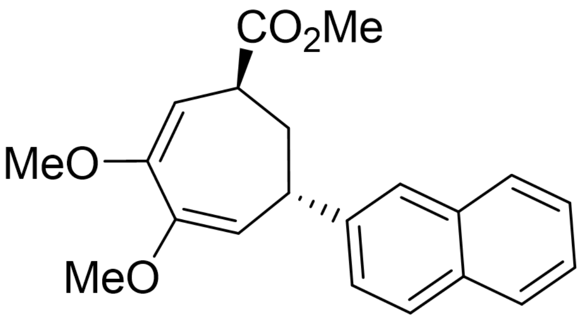

5h

rac

===

Acq. Operator : SYSTEM Seq. Line : 30
Sample Operator : SYSTEM
Acq. Instrument : HPLC Location : P1-A-01
Injection Date : 2/12/2024 9:34:21 pm Inj : 1
Inj Volume : 2.000 µl
Different Inj Volume from Sample Entry! Actual Inj Volume : 10.000 µl
Method : C:\Users\Public\Documents\ChemStation\1\Data\SUN\SUN 2024-12-02 10-37-19
\AD3-10-15.M (Sequence Method)
Last changed : 28/12/2022 10:29:24 pm by SYSTEM
Additional Info : Peak(s) manually integrated

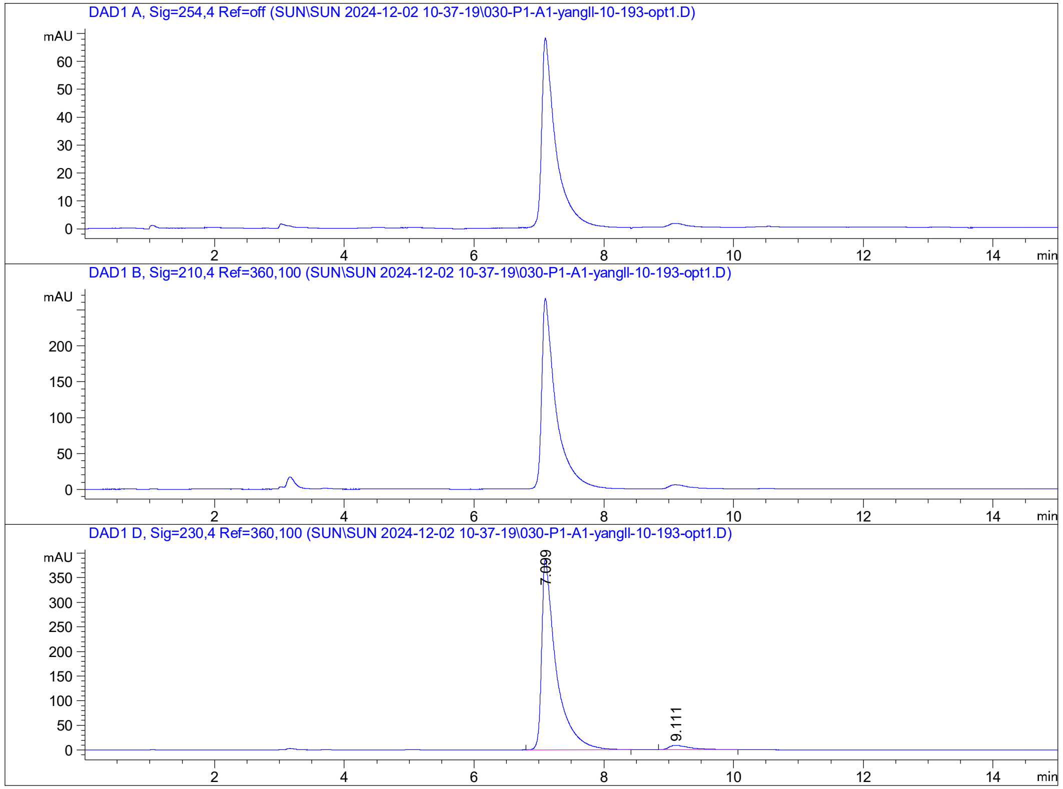

===

Area Percent Report

===

Sorted By : Signal
Multiplier : 1.0000
Dilution : 1.0000
Use Multiplier & Dilution Factor with ISTDs

Data File C:\Users\P...n\1\Data\SUN\SUN 2024-12-02 10-37-19\030-P1-A1-yangll-10-193-opt1.D
Sample Name: yangll-10-193-opt1

Signal 1: DAD1 A, Sig=254,4 Ref=off

Signal 2: DAD1 B, Sig=210,4 Ref=360,100

Signal 3: DAD1 D, Sig=230,4 Ref=360,100

Peak #	RetTime [min]	Type	Width [min]	Area [mAU*s]	Height [mAU]	Area %
1	7.099	BB	0.2103	6027.55322	387.78851	96.6215
2	9.111	BB	0.2783	210.76463	8.96342	3.3785

Totals : 6238.31786 396.75194

===

*** End of Report ***

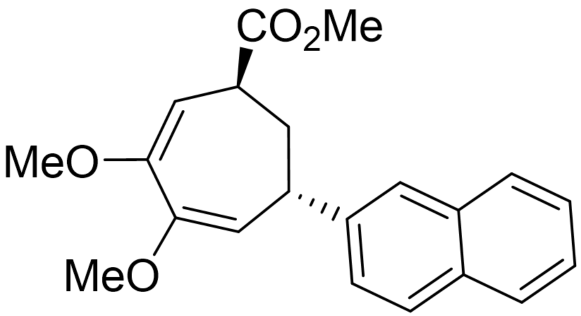

5h

opt

===

Acq. Operator : SYSTEM   Seq. Line : 23
Sample Operator : SYSTEM
Acq. Instrument : HPLC   Location : P1-F-02
Injection Date : 9/8/2024 11:34:40 pm   Inj : 1
Inj Volume : 2.000 µl
Method : C:\Users\Public\Documents\ChemStation\1\Data\SUN\SUN 2024-08-09 16-53-26
\AD3-10-10.M (Sequence Method)
Last changed : 15/8/2022 10:41:36 pm by SYSTEM
Additional Info : Peak(s) manually integrated

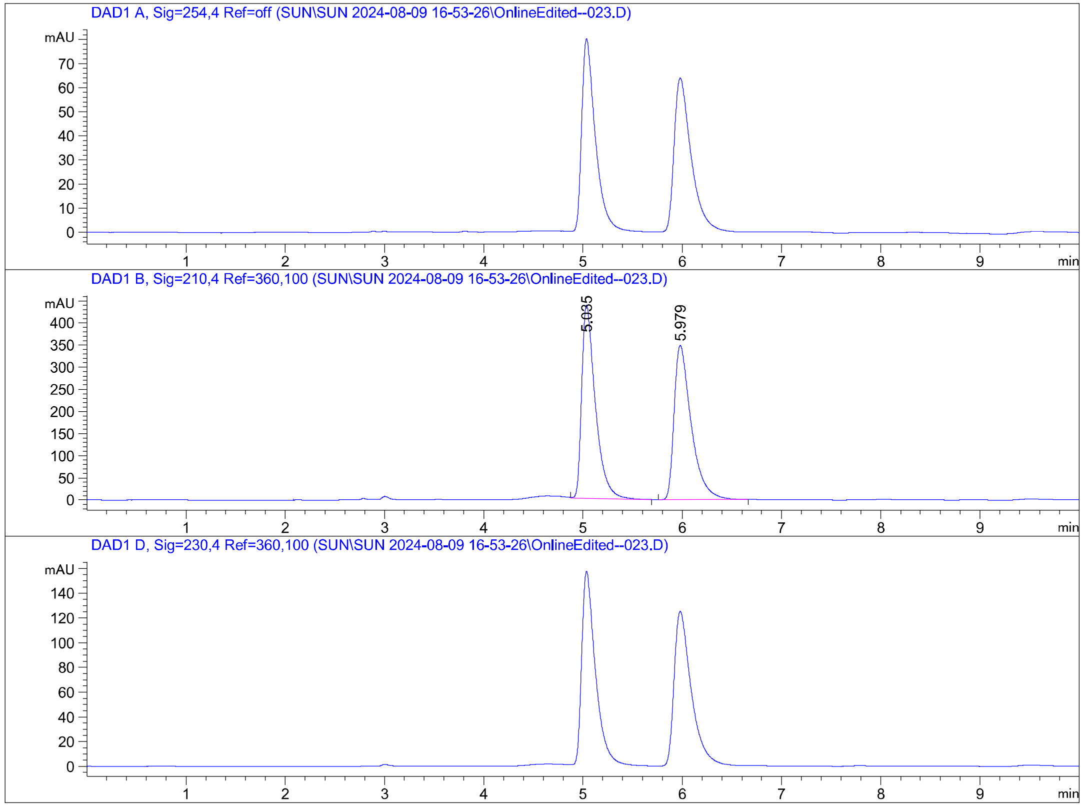

===

Area Percent Report

===

Sorted By : Signal
Multiplier : 1.0000
Dilution : 1.0000
Use Multiplier & Dilution Factor with ISTDs

Data File C:\Users\P...\ChemStation\1\Data\SUN\SUN 2024-08-09 16-53-26\OnlineEdited--023.D
Sample Name: yangll-10-68-rac

Signal 1: DAD1 A, Sig=254,4 Ref=off

Signal 2: DAD1 B, Sig=210,4 Ref=360,100

Peak #	RetTime [min]	Type	Width [min]	Area [mAU*s]	Height [mAU]	Area %
1	5.035	MM R	0.1594	4171.86914	436.32040	50.0117
2	5.979	BB	0.1790	4169.92090	348.75497	49.9883

Totals : 8341.79004 785.07538

Signal 3: DAD1 D, Sig=230,4 Ref=360,100

===

*** End of Report ***

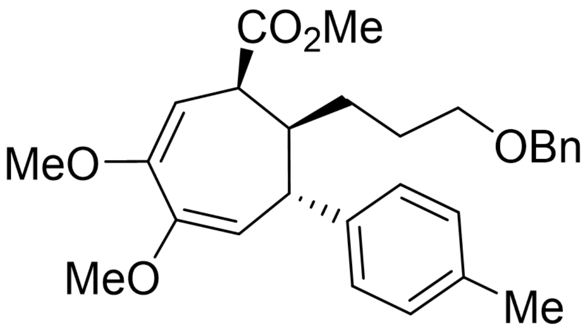

5v

rac

```
=====================================================================
Acq. Operator   : SYSTEM                    Seq. Line :  22
Sample Operator : SYSTEM
Acq. Instrument : HPLC                       Location :   P1-F-01
Injection Date  : 9/8/2024 11:23:41 pm            Inj :   1
                                           Inj Volume : 2.000 µl
Method          : C:\Users\Public\Documents\ChemStation\1\Data\SUN\SUN 2024-08-09 16-53-26
                  \AD3-10-10.M (Sequence Method)
Last changed    : 15/8/2022 10:41:36 pm by SYSTEM
Additional Info : Peak(s) manually integrated
```

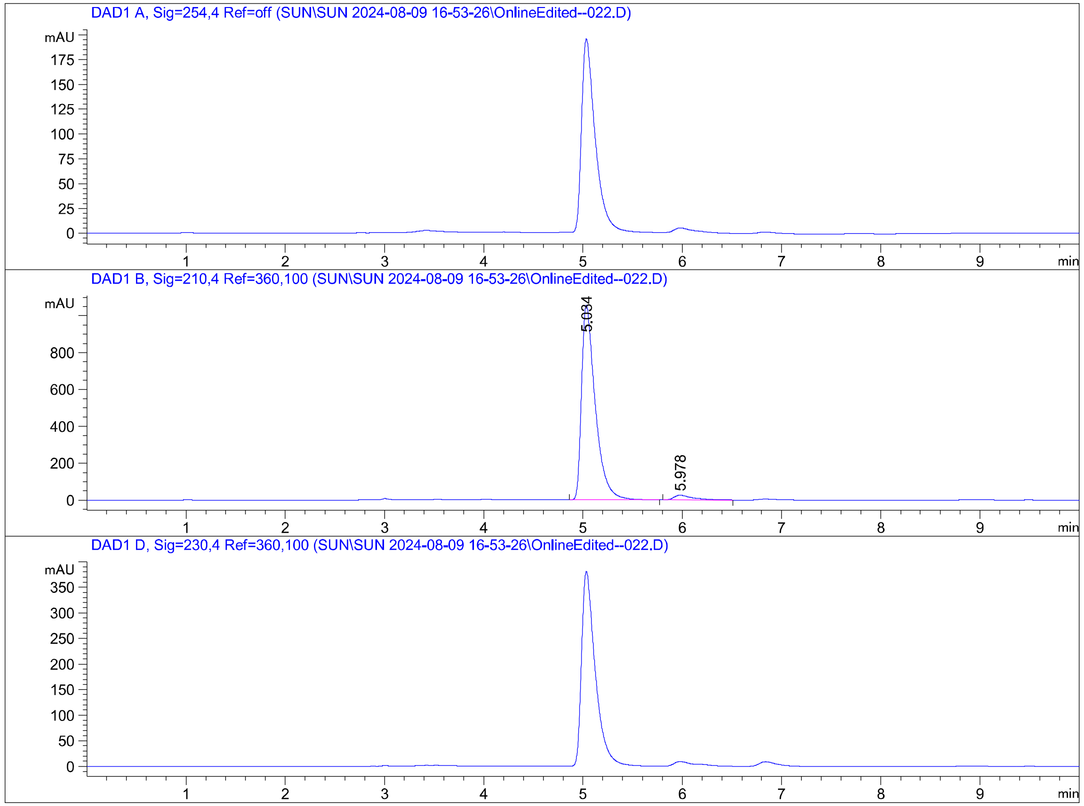


```
=====================================================================
                         Area Percent Report
=====================================================================

Sorted By             :      Signal
Multiplier            :      1.0000
Dilution              :      1.0000
Use Multiplier & Dilution Factor with ISTDs
```


Data File C:\Users\P...\ChemStation\1\Data\SUN\SUN 2024-08-09 16-53-26\OnlineEdited--022.D
Sample Name: yangll-10-68-opt

Signal 1: DAD1 A, Sig=254,4 Ref=off

Signal 2: DAD1 B, Sig=210,4 Ref=360,100

Peak #	RetTime [min]	Type	Width [min]	Area [mAU*s]	Height [mAU]	Area %
1	5.034	MM R	0.1601	1.01478e4	1056.12048	96.8080
2	5.978	MM R	0.2178	334.60223	25.60877	3.1920
Totals :				1.04824e4	1081.72926	

Signal 3: DAD1 D, Sig=230,4 Ref=360,100

```
=====================================================================
                         *** End of Report ***
```

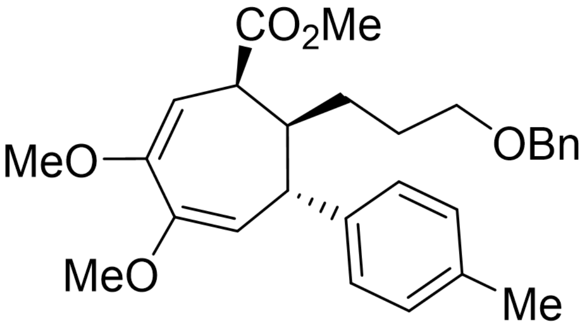

5v

opt

===

Acq. Operator : SYSTEM Seq. Line : 11
Sample Operator : SYSTEM
Acq. Instrument : HPLC Location : P1-A-01
Injection Date : 3/12/2024 10:37:39 pm Inj : 1
Inj Volume : 2.000 µl
Different Inj Volume from Sample Entry! Actual Inj Volume : 5.000 µl
Acq. Method : C:\Users\Public\Documents\ChemStation\1\Data\SUN\SUN 2024-12-03 19-34-37\IBN3-30-30.M
Last changed : 3/12/2024 10:53:39 pm by SYSTEM
(modified after loading)
Analysis Method : C:\Users\Public\Documents\ChemStation\1\Data\SUN\SUN 2024-12-03 19-34-37\IBN3-30-30.M (Sequence Method)
Last changed : 3/12/2024 10:57:47 pm by SYSTEM
Additional Info : Peak(s) manually integrated

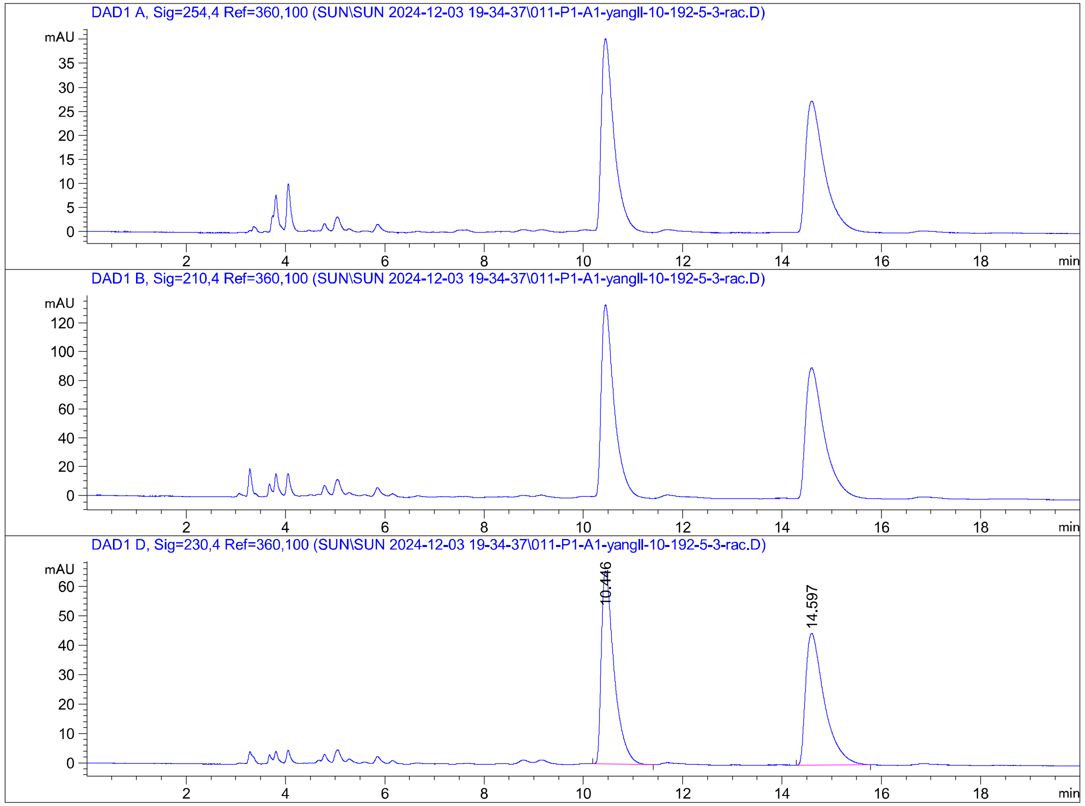

Data File C:\Users\P...\Data\SUN\SUN 2024-12-03 19-34-37\011-P1-A1-yangll-10-192-5-3-rac.D
Sample Name: yangll-10-192-5-3-rac

===

Area Percent Report

===

Sorted By : Signal
Multiplier : 1.0000
Dilution : 1.0000
Use Multiplier & Dilution Factor with ISTDs

Signal 1: DAD1 A, Sig=254,4 Ref=360,100

Signal 2: DAD1 B, Sig=210,4 Ref=360,100

Signal 3: DAD1 D, Sig=230,4 Ref=360,100

Peak #	RetTime [min]	Type	Width [min]	Area [mAU*s]	Height [mAU]	Area %
1	10.446	BB	0.2653	1176.89819	65.60094	50.0959
2	14.597	BB	0.3740	1172.39136	44.77780	49.9041

Totals : 2349.28955 110.37873

===

*** End of Report ***

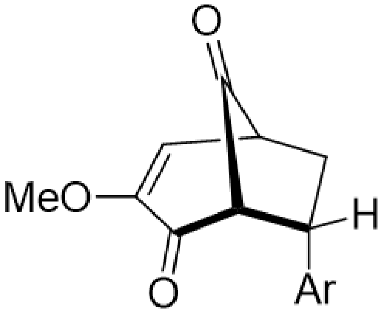

Ar = 4-$CH_3C_6H_4$

4b'

rac

===

```
Acq. Operator   : SYSTEM                        Seq. Line :  16
Sample Operator : SYSTEM
Acq. Instrument : HPLC                           Location :   P1-A-02
Injection Date  : 3/12/2024 11:50:29 pm               Inj :    1
                                               Inj Volume : 2.000 µl
Different Inj Volume from Sample Entry!  Actual Inj Volume : 20.000 µl
Method          : C:\Users\Public\Documents\ChemStation\1\Data\SUN\SUN 2024-12-03 19-34-37
                  \IBN3-30-30.M (Sequence Method)
Last changed    : 3/12/2024 10:57:47 pm by SYSTEM
Additional Info : Peak(s) manually integrated
```

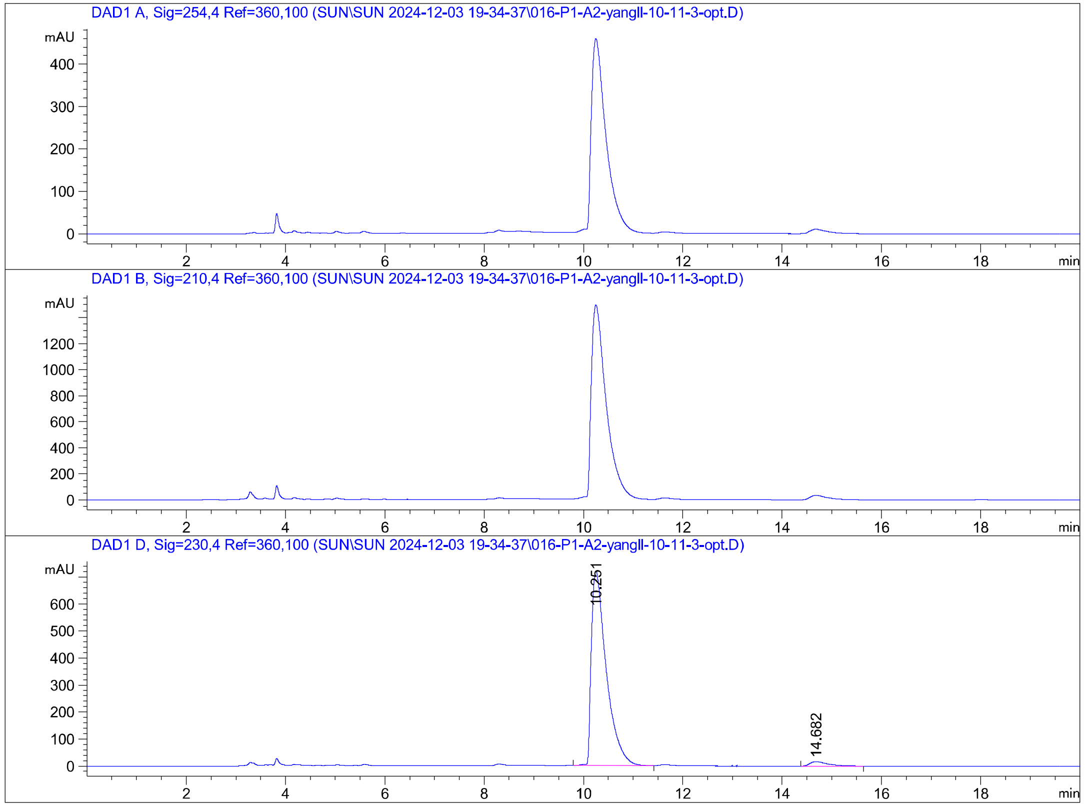

===

Area Percent Report

===

```
Sorted By             :      Signal
Multiplier            :      1.0000
Dilution              :      1.0000
Use Multiplier & Dilution Factor with ISTDs
```


Data File C:\Users\P...n\1\Data\SUN\SUN 2024-12-03 19-34-37\016-P1-A2-yangll-10-11-3-opt.D
Sample Name: yangll-10-11-3-opt

Signal 1: DAD1 A, Sig=254,4 Ref=360,100

Signal 2: DAD1 B, Sig=210,4 Ref=360,100

Signal 3: DAD1 D, Sig=230,4 Ref=360,100

Peak #	RetTime [min]	Type	Width [min]	Area [mAU*s]	Height [mAU]	Area %
1	10.251	BV	0.3135	1.50633e4	720.27679	97.3966
2	14.682	BB	0.3217	402.63361	16.47241	2.6034

Totals : 1.54659e4 736.74921

===

*** End of Report ***

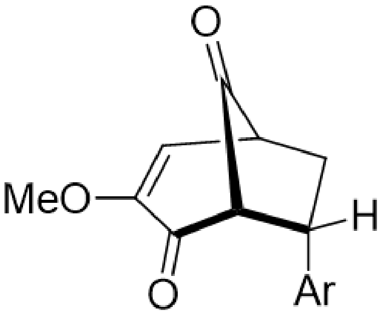

Ar = 4-$CH_3C_6H_4$

4b'

opt

===

Acq. Operator : SYSTEM Seq. Line : 4
Sample Operator : SYSTEM
Acq. Instrument : HPLC Location : P1-A-01
Injection Date : 14/6/2024 6:40:27 pm Inj : 1
Inj Volume : 2.000 µl
Different Inj Volume from Sample Entry! Actual Inj Volume : 5.000 µl
Method : C:\Users\Public\Documents\ChemStation\1\Data\SUN\SUN 2024-06-14 18-02-28 \AD3-30-15.M (Sequence Method)
Last changed : 27/8/2023 6:30:58 pm by SYSTEM
Additional Info : Peak(s) manually integrated

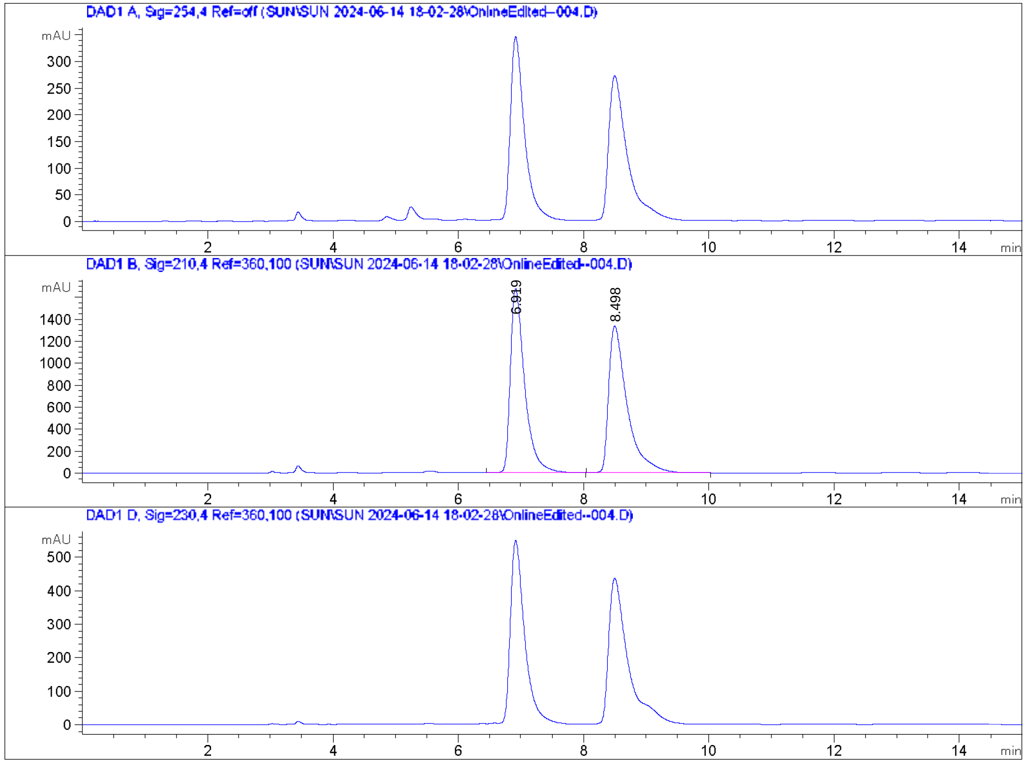

===

Area Percent Report

===

Sorted By : Signal
Multiplier : 1.0000
Dilution : 1.0000
Use Multiplier & Dilution Factor with ISTDs

Signal 1: DAD1 A, Sig=254,4 Ref=off

Signal 2: DAD1 B, Sig=210,4 Ref=360,100

Peak #	RetTime [min]	Type	Width [min]	Area [mAU*s]	Height [mAU]	Area %
1	6.919	VB R	0.2379	2.65543e4	1673.50562	49.2336
2	8.498	BB	0.3028	2.73811e4	1334.69373	50.7664

Totals : 5.39354e4 3008.19934

Signal 3: DAD1 D, Sig=230,4 Ref=360,100

===

*** End of Report ***

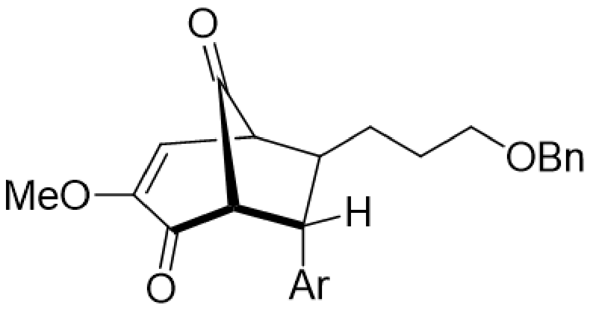

Ar = 4-$CH_3C_6H_4$

4v'

rac

===

Acq. Operator : SYSTEM Seq. Line : 5
Sample Operator : SYSTEM
Acq. Instrument : HPLC Location : P1-A-02
Injection Date : 14/6/2024 6:56:40 pm Inj : 1
Inj Volume : 2.000 µl
Different Inj Volume from Sample Entry! Actual Inj Volume : 5.000 µl
Method : C:\Users\Public\Documents\ChemStation\1\Data\SUN\SUN 2024-06-14 18-02-28 \AD3-30-15.M (Sequence Method)
Last changed : 27/8/2023 6:30:58 pm by SYSTEM
Additional Info : Peak(s) manually integrated

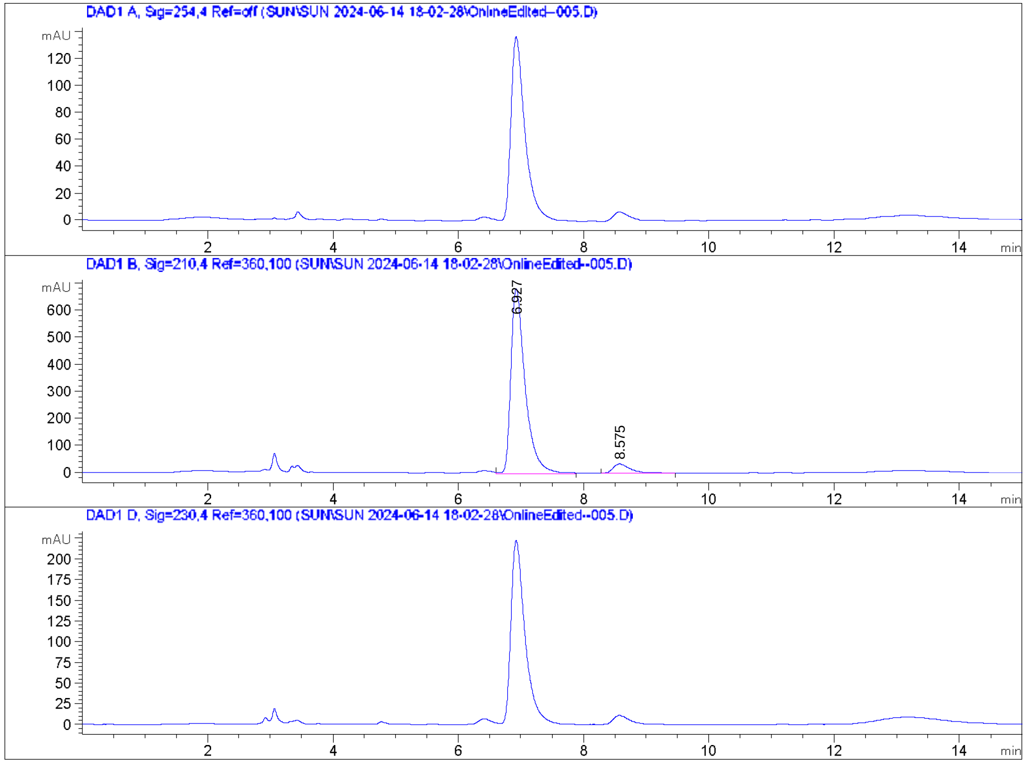

===
Area Percent Report
===

Sorted By : Signal
Multiplier : 1.0000
Dilution : 1.0000
Use Multiplier & Dilution Factor with ISTDs

Data File C:\Users\P...\ChemStation\1\Data\SUN\SUN 2024-06-14 18-02-28\OnlineEdited--005.D
Sample Name: yangll-9-184-opt

Signal 1: DAD1 A, Sig=254,4 Ref=off

Signal 2: DAD1 B, Sig=210,4 Ref=360,100

Peak #	RetTime [min]	Type	Width [min]	Area [mAU*s]	Height [mAU]	Area %
1	6.927	MM R	0.2654	1.08869e4	683.66504	94.4345
2	8.575	BB	0.2716	641.61792	34.01271	5.5655

Totals : 1.15285e4 717.67775

Signal 3: DAD1 D, Sig=230,4 Ref=360,100

===
*** End of Report ***

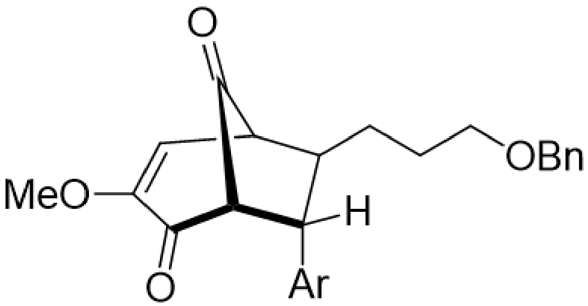

Ar = 4-$CH_3C_6H_4$

4v'

opt

===

Acq. Operator : SYSTEM Seq. Line : 5
Acq. Instrument : LC1260 Location : P2-A-03
Injection Date : 7/3/2024 10:13:45 PM Inj : 1
Inj Volume : 5.000 µl
Different Inj Volume from Sample Entry! Actual Inj Volume : 20.000 µl
Acq. Method : C:\Users\Public\Documents\ChemStation\1\Data\SUN\SUN 2024-07-03 19-47-43\ID3-20-30.M
Last changed : 5/8/2024 10:09:31 AM by SYSTEM
Analysis Method : C:\Users\Public\Documents\ChemStation\1\Data\SUN\SUN 2024-07-03 19-47-43\ID3-20-30.M (Sequence Method)
Last changed : 7/9/2024 8:22:43 PM by SYSTEM
(modified after loading)
Additional Info : Peak(s) manually integrated

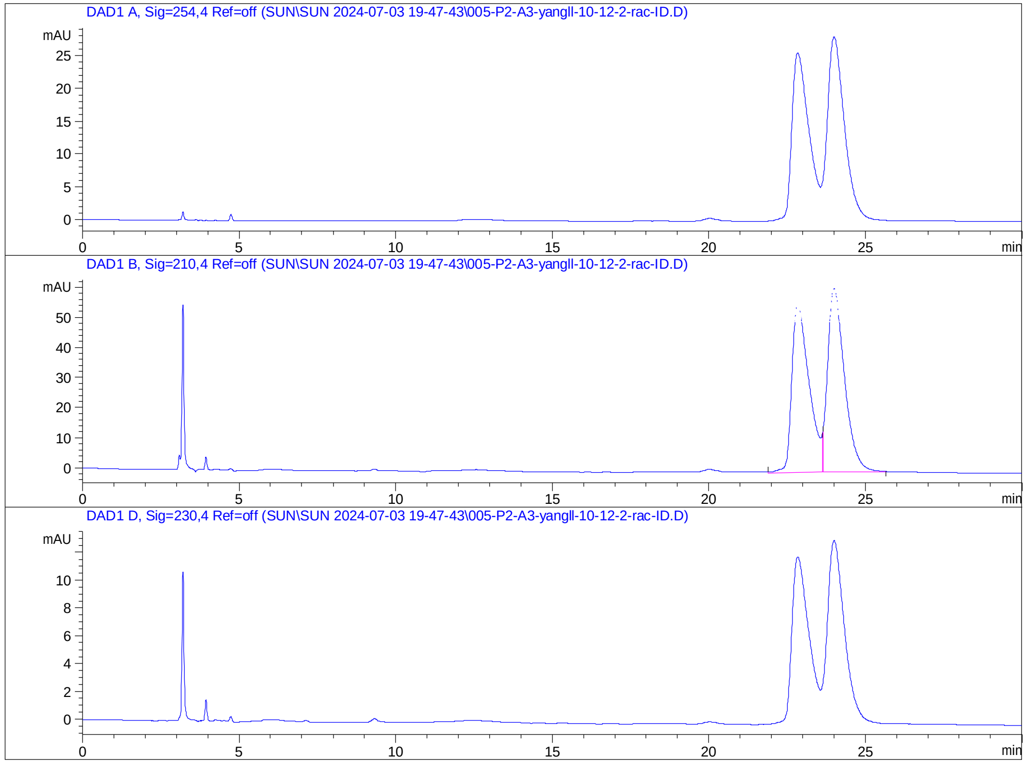

===
Area Percent Report
===

Sorted By : Signal
Multiplier : 1.0000
Dilution : 1.0000
Use Multiplier & Dilution Factor with ISTDs

Data File C:\Users\P...\Data\SUN\SUN 2024-07-03 19-47-43\005-P2-A3-yangll-10-12-2-rac-ID.D
Sample Name: yangll-10-12-2-rac-ID

Signal 1: DAD1 A, Sig=254,4 Ref=off

Signal 2: DAD1 B, Sig=210,4 Ref=off

Peak #	RetTime [min]	Type	Width [min]	Area [mAU*s]	Height [mAU]	Area %
1	22.843	MM R	0.6470	2168.32837	55.85618	48.6581
2	24.006	MM R	0.6261	2287.92871	60.90644	51.3419

Totals : 4456.25708 116.76262

Signal 3: DAD1 D, Sig=230,4 Ref=off

===
*** End of Report ***

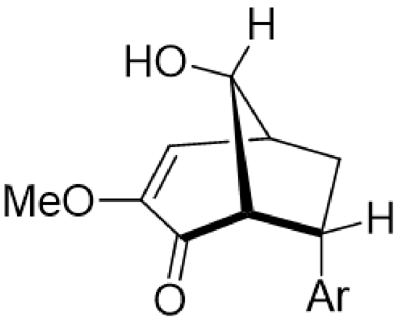

Ar = 4-$CH_3C_6H_4$

6

rac

Data File C:\Users\P...n\1\Data\SUN\SUN 2024-07-03 19-47-43\006-P1-F2-yangll-10-12-2-opt.D
Sample Name: yangll-10-12-2-opt

===

Acq. Operator : SYSTEM   Seq. Line : 6
Acq. Instrument : LC1260   Location : P1-F-02
Injection Date : 7/3/2024 10:44:52 PM   Inj : 1
Inj Volume : 5.000 µl
Different Inj Volume from Sample Entry! Actual Inj Volume : 10.000 µl
Acq. Method : C:\Users\Public\Documents\ChemStation\1\Data\SUN\SUN 2024-07-03 19-47-43\ID3-20-30.M
Last changed : 5/8/2024 10:09:31 AM by SYSTEM
Analysis Method : C:\Users\Public\Documents\ChemStation\1\Data\SUN\SUN 2024-07-03 19-47-43\ID3-20-30.M (Sequence Method)
Last changed : 7/9/2024 8:22:43 PM by SYSTEM
(modified after loading)
Additional Info : Peak(s) manually integrated

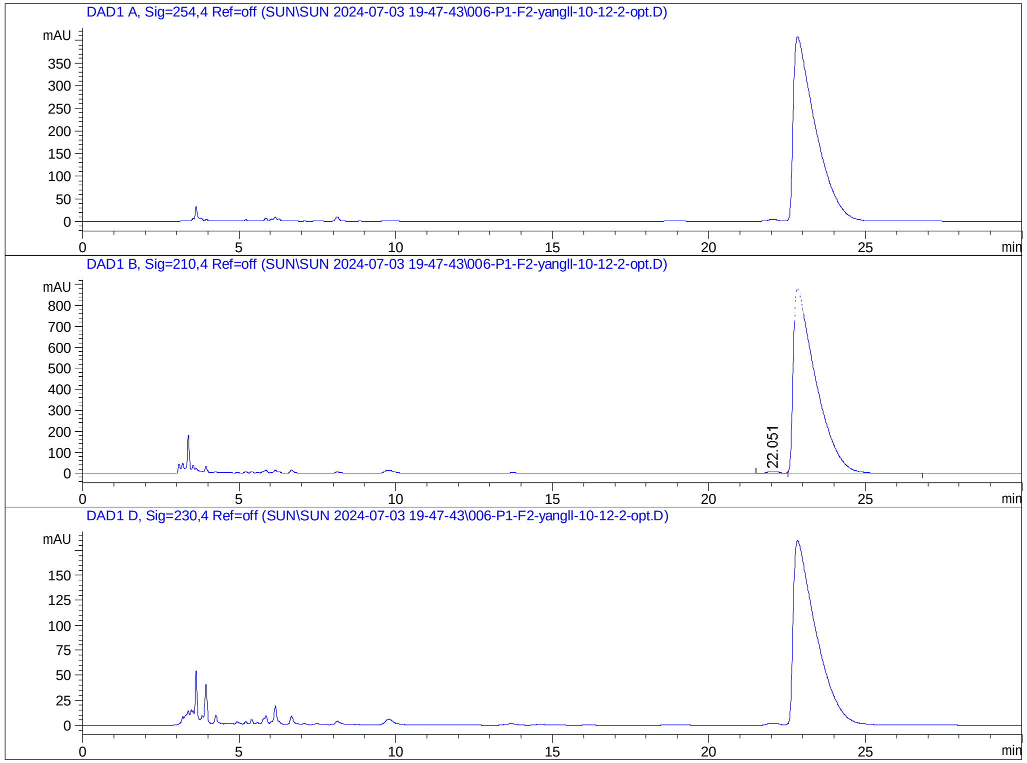

===

Area Percent Report

===

Sorted By : Signal
Multiplier : 1.0000
Dilution : 1.0000
Use Multiplier & Dilution Factor with ISTDs

Data File C:\Users\P...n\1\Data\SUN\SUN 2024-07-03 19-47-43\006-P1-F2-yangll-10-12-2-opt.D
Sample Name: yangll-10-12-2-opt

Signal 1: DAD1 A, Sig=254,4 Ref=off

Signal 2: DAD1 B, Sig=210,4 Ref=off

Peak #	RetTime [min]	Type	Width [min]	Area [mAU*s]	Height [mAU]	Area %
1	22.051	BV E	0.4222	236.37573	8.72043	0.5364
2	22.839	BB R	0.6930	4.38320e4	880.22943	99.4636

Totals : 4.40684e4 888.94986

Signal 3: DAD1 D, Sig=230,4 Ref=off

===

*** End of Report ***

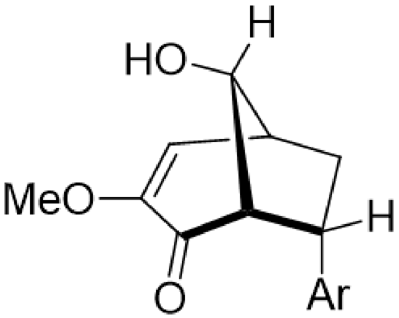

Ar = 4-$CH_3C_6H_4$

6

opt

===

Acq. Operator : SYSTEM   Seq. Line : 4
Acq. Instrument : LC1260   Location : P1-A-01
Injection Date : 6/19/2024 3:25:12 PM   Inj : 1
Inj Volume : 5.000 µl
Acq. Method : C:\Users\Public\Documents\ChemStation\1\Data\SUN\SUN 2024-06-19 14-30-23\IBN3-20-40.M
Last changed : 5/14/2024 9:29:06 AM by SYSTEM
Analysis Method : C:\Users\Public\Documents\ChemStation\1\Data\SUN\SUN 2024-06-19 14-30-23\IBN3-20-40.M (Sequence Method)
Last changed : 6/25/2024 5:59:47 PM by SYSTEM
(modified after loading)
Additional Info : Peak(s) manually integrated

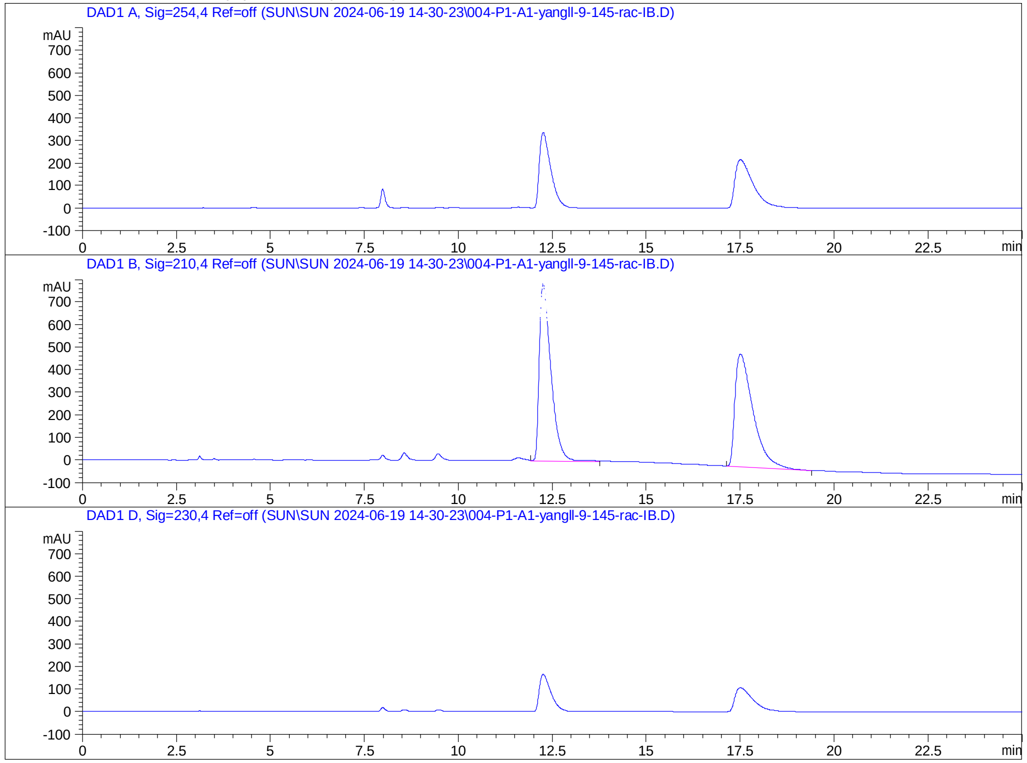

===

Area Percent Report

===

Sorted By : Signal
Multiplier : 1.0000
Dilution : 1.0000
Use Multiplier & Dilution Factor with ISTDs

Data File C:\Users\P...\1\Data\SUN\SUN 2024-06-19 14-30-23\004-P1-A1-yangll-9-145-rac-IB.D
Sample Name: yangll-9-145-rac-IB

Signal 1: DAD1 A, Sig=254,4 Ref=off

Signal 2: DAD1 B, Sig=210,4 Ref=off

Peak #	RetTime [min]	Type	Width [min]	Area [mAU*s]	Height [mAU]	Area %
1	12.256	MM R	0.3617	1.70592e4	786.07092	50.3308
2	17.509	BB	0.5100	1.68350e4	500.98010	49.6692

Totals : 3.38942e4 1287.05103

Signal 3: DAD1 D, Sig=230,4 Ref=off

===

*** End of Report ***

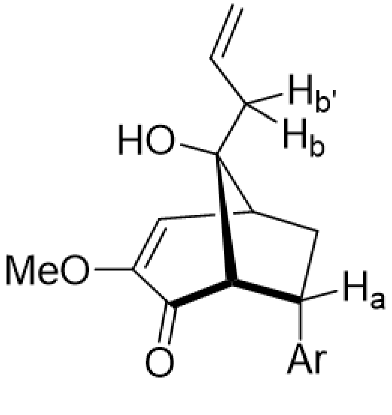

7

rac

===

```
Acq. Operator   : SYSTEM                        Seq. Line :    2
Acq. Instrument : LC1260                         Location :   P1-F-01
Injection Date  : 6/25/2024 2:43:04 PM                Inj :    1
                                               Inj Volume : 5.000 µl
Acq. Method     : C:\Users\Public\Documents\ChemStation\1\Data\SUN\SUN 2024-06-25 14-30-19
                  \IBN3-20-30.M
Last changed    : 6/25/2024 2:29:39 PM by SYSTEM
Analysis Method : C:\Users\Public\Documents\ChemStation\1\Data\SUN\SUN 2024-06-25 14-30-19
                  \IBN3-20-30.M (Sequence Method)
Last changed    : 6/25/2024 5:56:15 PM by SYSTEM
                  (modified after loading)
Additional Info : Peak(s) manually integrated
```

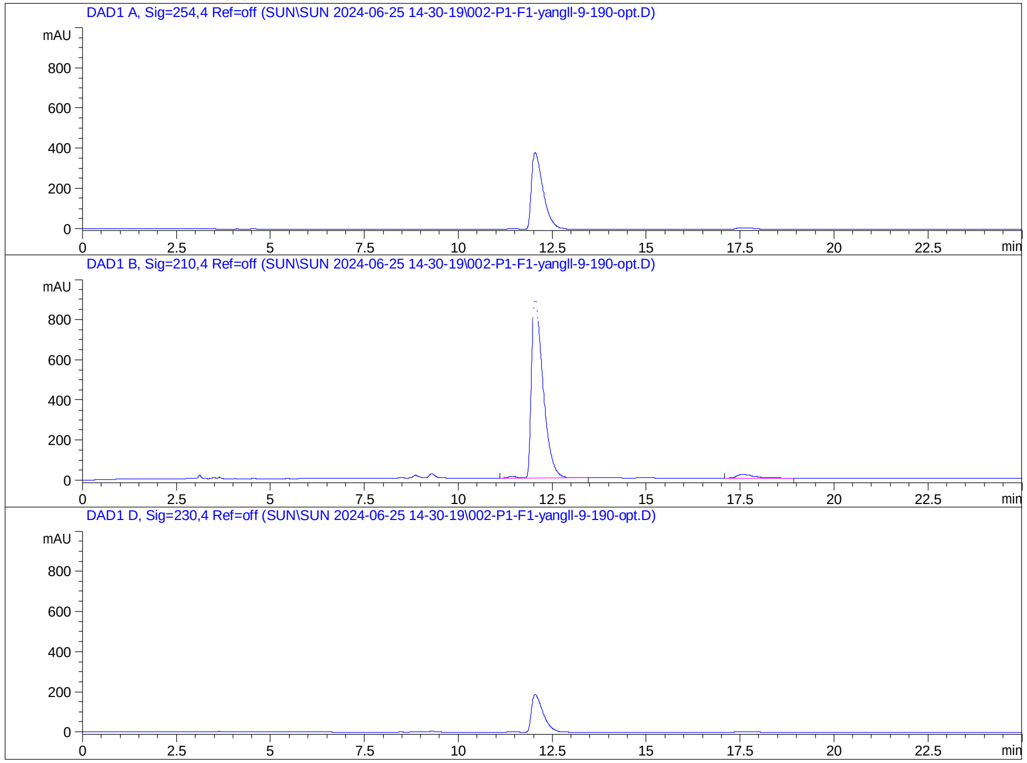

===

Area Percent Report

===

```
Sorted By             :      Signal
Multiplier            :      1.0000
Dilution              :      1.0000
Use Multiplier & Dilution Factor with ISTDs
```


Signal 1: DAD1 A, Sig=254,4 Ref=off

Signal 2: DAD1 B, Sig=210,4 Ref=off

Peak #	RetTime [min]	Type	Width [min]	Area [mAU*s]	Height [mAU]	Area %
1	12.047	BB R	0.3290	1.93342e4	891.80865	96.9831
2	17.574	BB	0.4729	601.44409	19.02016	3.0169
Totals :				1.99356e4	910.82882	

Signal 3: DAD1 D, Sig=230,4 Ref=off

===

*** End of Report ***

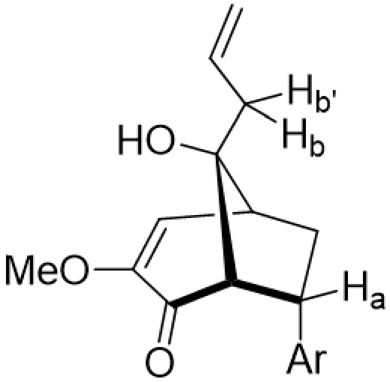

7

opt

===

Acq. Operator	: SYSTEM	Seq. Line :	2
Acq. Instrument	: LC1260	Location :	P1-F-01
Injection Date	: 6/30/2024 12:55:13 PM	Inj :	1
		Inj Volume :	5.000 µl

Acq. Method : C:\Users\Public\Documents\ChemStation\1\Data\SUN\SUN 2024-06-30 12-42-26 \IBN3-05-15.M
Last changed : 3/25/2024 4:07:02 PM by SYSTEM
Analysis Method : C:\Users\Public\Documents\ChemStation\1\Data\SUN\SUN 2024-06-30 12-42-26 \IBN3-05-15.M (Sequence Method)
Last changed : 6/30/2024 2:01:26 PM by SYSTEM (modified after loading)
Additional Info : Peak(s) manually integrated

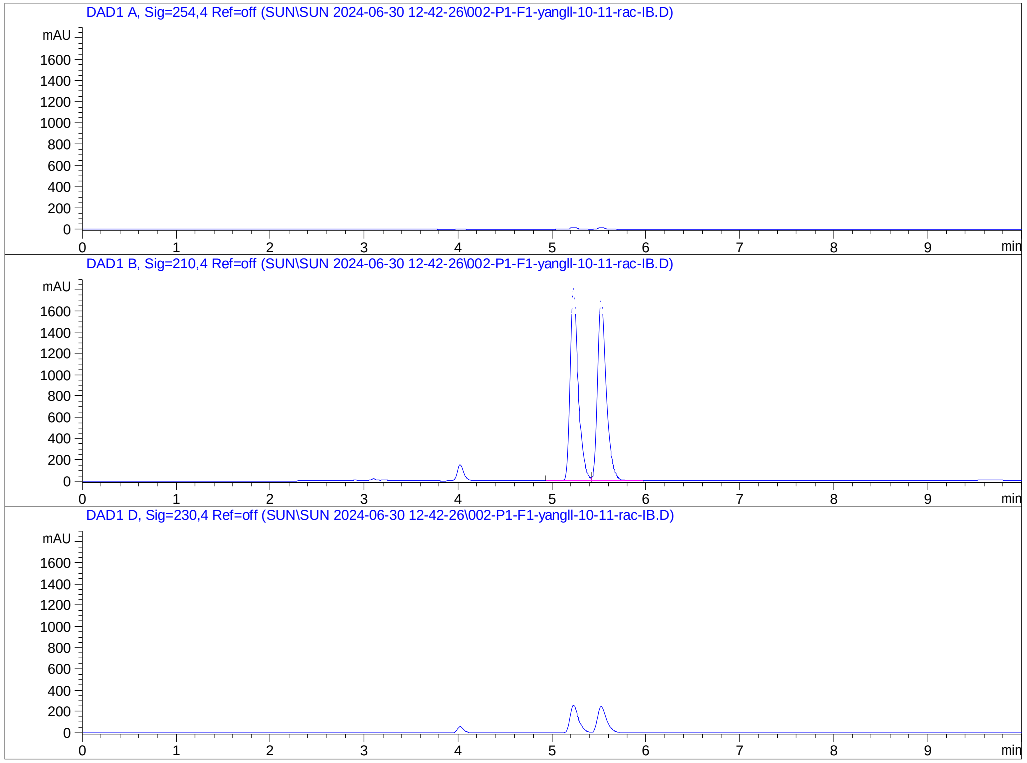

===
Area Percent Report
===

Sorted By : Signal
Multiplier : 1.0000
Dilution : 1.0000
Use Multiplier & Dilution Factor with ISTDs

Data File C:\Users\P...\1\Data\SUN\SUN 2024-06-30 12-42-26\002-P1-F1-yangll-10-11-rac-IB.D
Sample Name: yangll-10-11-rac-IB

Signal 1: DAD1 A, Sig=254,4 Ref=off

Signal 2: DAD1 B, Sig=210,4 Ref=off

Peak #	RetTime [min]	Type	Width [min]	Area [mAU*s]	Height [mAU]	Area %
1	5.230	BV R	0.0894	1.07753e4	1811.93262	49.8043
2	5.524	VB	0.0938	1.08599e4	1716.13159	50.1957
Totals :				2.16352e4	3528.06421	

Signal 3: DAD1 D, Sig=230,4 Ref=off

===
*** End of Report ***

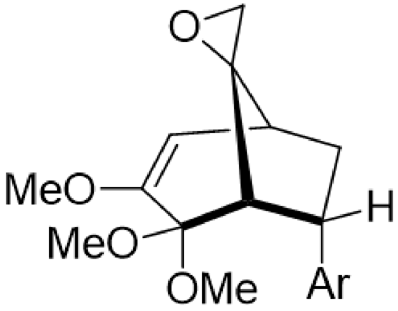

Ar = 4-$CH_3C_6H_4$

8

rac

Data File C:\Users\P...ion\1\Data\SUN\SUN 2024-07-02 11-53-10\003-P1-F2-yangll-10-11-opt.D
Sample Name: yangll-10-11-opt

===

Acq. Operator : SYSTEM   Seq. Line : 3
Acq. Instrument : LC1260   Location : P1-F-02
Injection Date : 7/2/2024 12:22:01 PM   Inj : 1
Inj Volume : 5.000 µl
Acq. Method : C:\Users\Public\Documents\ChemStation\1\Data\SUN\SUN 2024-07-02 11-53-10 \IBN3-05-15.M
Last changed : 3/25/2024 4:07:02 PM by SYSTEM
Analysis Method : C:\Users\Public\Documents\ChemStation\1\Data\SUN\SUN 2024-07-02 11-53-10 \IBN3-05-15.M (Sequence Method)
Last changed : 7/9/2024 8:39:57 PM by SYSTEM
(modified after loading)
Additional Info : Peak(s) manually integrated

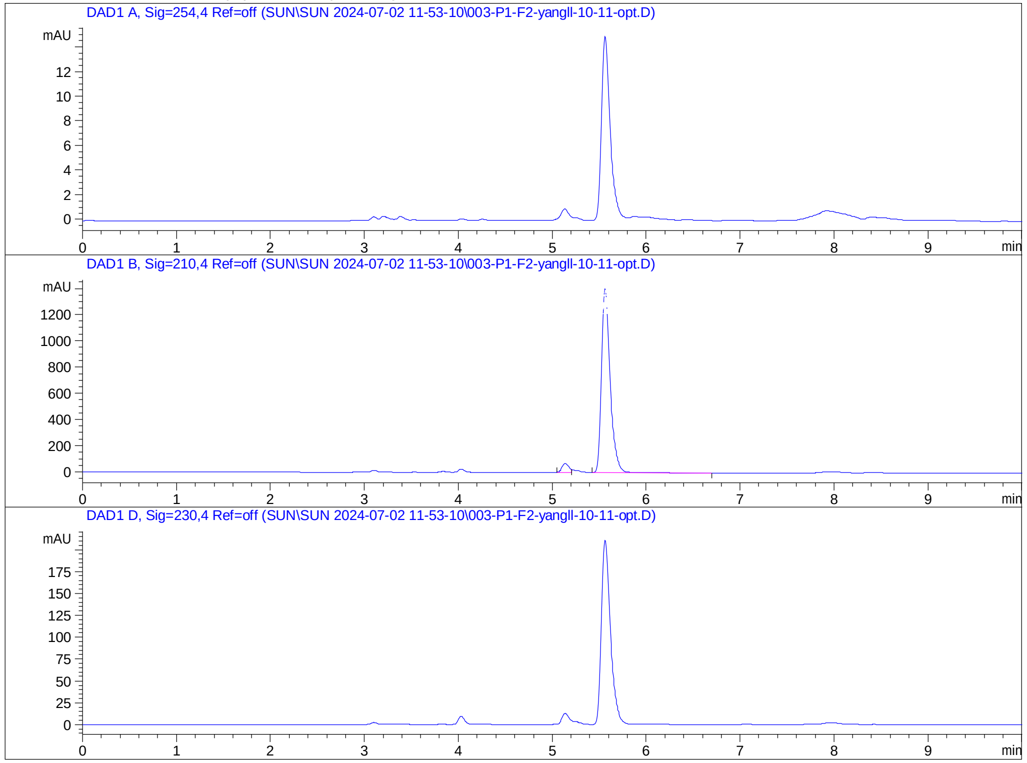

===
Area Percent Report
===

Sorted By : Signal
Multiplier : 1.0000
Dilution : 1.0000
Use Multiplier & Dilution Factor with ISTDs

Signal 1: DAD1 A, Sig=254,4 Ref=off

Signal 2: DAD1 B, Sig=210,4 Ref=off

Peak #	RetTime [min]	Type	Width [min]	Area [mAU*s]	Height [mAU]	Area %
1	5.143	MM R	0.0864	360.30386	69.52289	3.8588
2	5.564	VB	0.0964	8976.80176	1407.77527	96.1412

Totals : 9337.10562 1477.29816

Signal 3: DAD1 D, Sig=230,4 Ref=off

===
*** End of Report ***

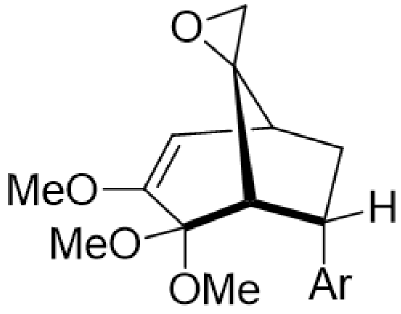

Ar = 4-$CH_3C_6H_4$

8

opt

===

Acq. Operator : SYSTEM Seq. Line : 9
Acq. Instrument : LC1260 Location : P2-A-01
Injection Date : 7/9/2024 9:39:23 PM Inj : 1
Inj Volume : 5.000 µl
Acq. Method : C:\Users\Public\Documents\ChemStation\1\Data\SUN\SUN 2024-07-09 19-30-21\ID3-20-30.M
Last changed : 7/9/2024 9:39:13 PM by SYSTEM (modified after loading)
Analysis Method : C:\Users\Public\Documents\ChemStation\1\Data\SUN\SUN 2024-07-09 19-30-21\ID3-20-30.M (Sequence Method)
Last changed : 7/9/2024 10:06:13 PM by SYSTEM (modified after loading)
Additional Info : Peak(s) manually integrated

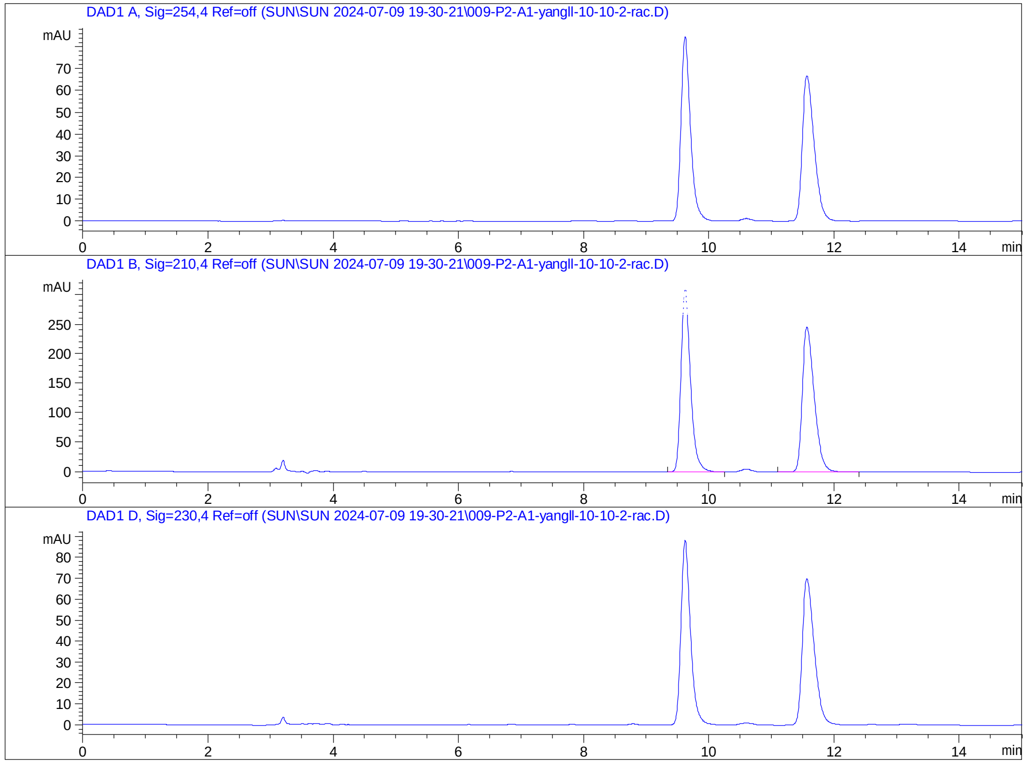

===

Area Percent Report

===

Sorted By : Signal
Multiplier : 1.0000
Dilution : 1.0000
Use Multiplier & Dilution Factor with ISTDs

Data File C:\Users\P...n\1\Data\SUN\SUN 2024-07-09 19-30-21\009-P2-A1-yangll-10-10-2-rac.D
Sample Name: yangll-10-10-2-rac

Signal 1: DAD1 A, Sig=254,4 Ref=off

Signal 2: DAD1 B, Sig=210,4 Ref=off

Peak #	RetTime [min]	Type	Width [min]	Area [mAU*s]	Height [mAU]	Area %
1	9.618	BB	0.1645	3313.64575	310.43695	50.3277
2	11.566	BB	0.2054	3270.49170	245.55669	49.6723

Totals : 6584.13745 555.99364

Signal 3: DAD1 D, Sig=230,4 Ref=off

===

*** End of Report ***

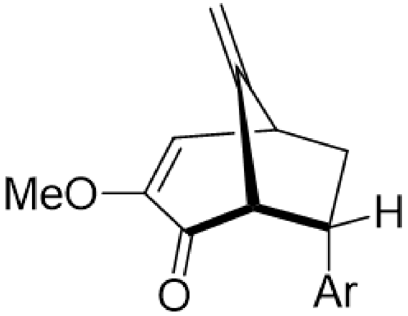

Ar = 4-$CH_3C_6H_4$

9

rac

Data File C:\Users\P...n\1\Data\SUN\SUN 2024-07-09 19-30-21\008-P1-F1-yangll-10-10-2-opt.D
Sample Name: yangll-10-10-2-opt

===

Acq. Operator	: SYSTEM	Seq. Line :	8
Acq. Instrument	: LC1260	Location :	P1-F-01
Injection Date	: 7/9/2024 9:11:00 PM	Inj :	1
		Inj Volume :	5.000 µl

Acq. Method : C:\Users\Public\Documents\ChemStation\1\Data\SUN\SUN 2024-07-09 19-30-21 \ID3-20-30.M
Last changed : 7/9/2024 9:38:27 PM by SYSTEM (modified after loading)
Analysis Method : C:\Users\Public\Documents\ChemStation\1\Data\SUN\SUN 2024-07-09 19-30-21 \ID3-20-30.M (Sequence Method)
Last changed : 7/9/2024 9:57:59 PM by SYSTEM (modified after loading)
Additional Info : Peak(s) manually integrated

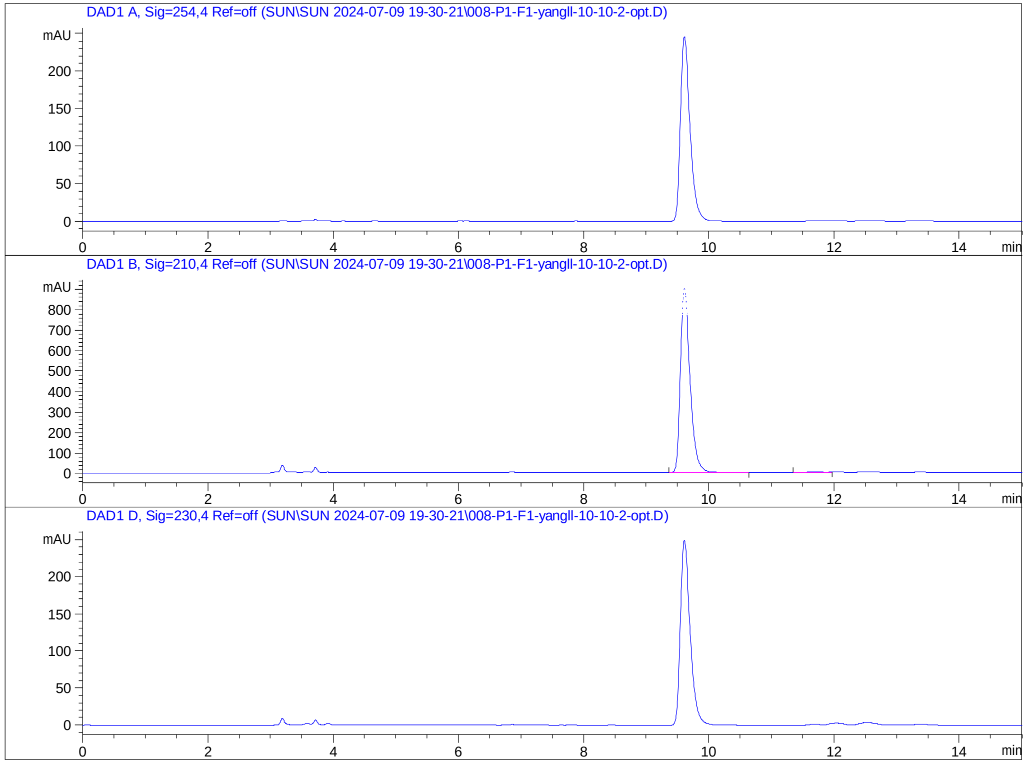

===

Area Percent Report

===

Sorted By	:	Signal
Multiplier	:	1.0000
Dilution	:	1.0000

Use Multiplier & Dilution Factor with ISTDs

Data File C:\Users\P...n\1\Data\SUN\SUN 2024-07-09 19-30-21\008-P1-F1-yangll-10-10-2-opt.D
Sample Name: yangll-10-10-2-opt

Signal 1: DAD1 A, Sig=254,4 Ref=off

Signal 2: DAD1 B, Sig=210,4 Ref=off

Peak #	RetTime [min]	Type	Width [min]	Area [mAU*s]	Height [mAU]	Area %
1	9.607	BB	0.1659	9692.75977	898.45673	99.3611
2	11.688	MM R	0.2567	62.32311	4.04696	0.6389

Totals : 9755.08287 902.50368

Signal 3: DAD1 D, Sig=230,4 Ref=off

===

*** End of Report ***

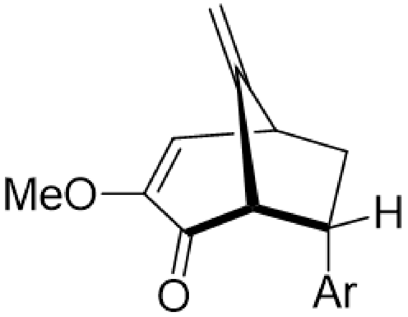

Ar = 4-$CH_3C_6H_4$

9

opt

===

Acq. Operator : SYSTEM Seq. Line : 12
Acq. Instrument : LC1260 Location : P1-F-02
Injection Date : 7/9/2024 10:27:23 PM Inj : 1
Inj Volume : 5.000 µl
Acq. Method : C:\Users\Public\Documents\ChemStation\1\Data\SUN\SUN 2024-07-09 19-30-21\IBN3-20-20.M
Last changed : 7/9/2024 10:40:48 PM by SYSTEM (modified after loading)
Analysis Method : C:\Users\Public\Documents\ChemStation\1\Data\SUN\SUN 2024-07-09 19-30-21\IBN3-20-20.M (Sequence Method)
Last changed : 7/9/2024 10:43:11 PM by SYSTEM (modified after loading)
Additional Info : Peak(s) manually integrated

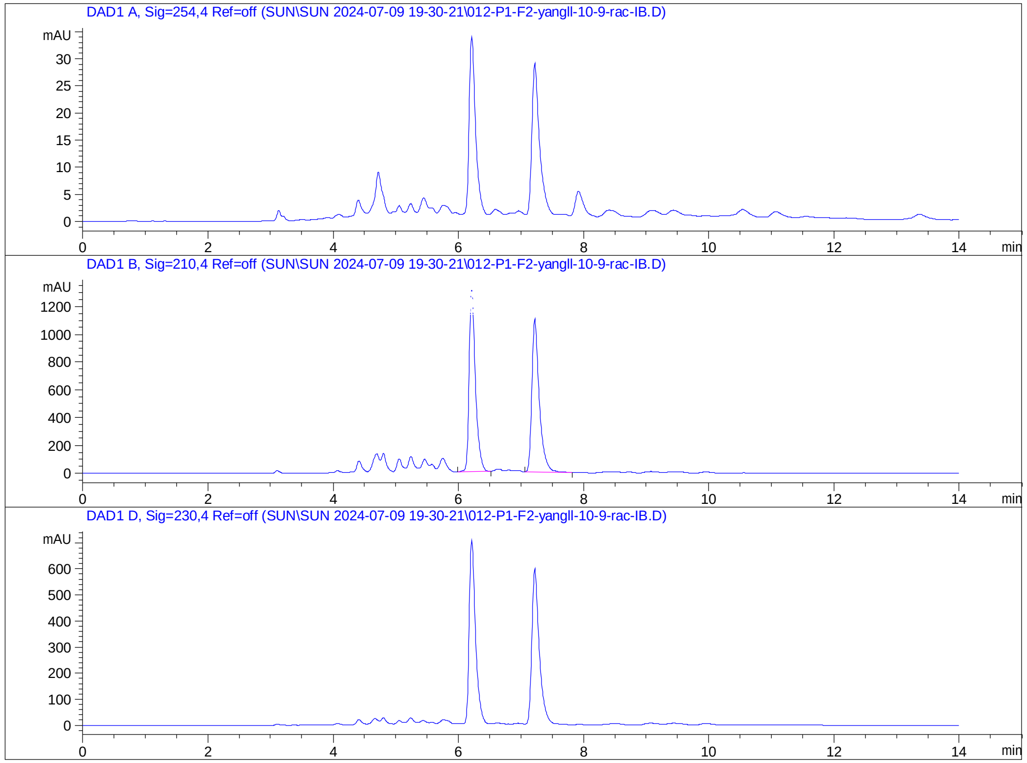

===
Area Percent Report
===

Sorted By : Signal
Multiplier : 1.0000
Dilution : 1.0000
Use Multiplier & Dilution Factor with ISTDs

Data File C:\Users\P...n\1\Data\SUN\SUN 2024-07-09 19-30-21\012-P1-F2-yangll-10-9-rac-IB.D
Sample Name: yangll-10-9-rac-IB

Signal 1: DAD1 A, Sig=254,4 Ref=off

Signal 2: DAD1 B, Sig=210,4 Ref=off

Peak #	RetTime [min]	Type	Width [min]	Area [mAU*s]	Height [mAU]	Area %
1	6.218	BB	0.1056	9225.88086	1319.85779	49.6925
2	7.223	BB	0.1245	9340.04590	1106.32861	50.3075

Totals : 1.85659e4 2426.18640

Signal 3: DAD1 D, Sig=230,4 Ref=off

===
*** End of Report ***

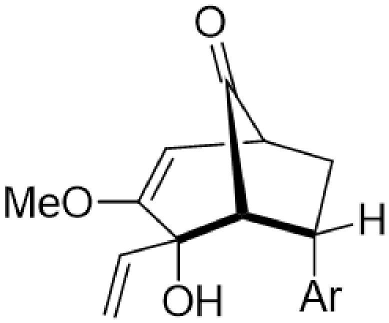

Ar = 4-CH$_3$C$_6$H$_4$
10
rac

===

Acq. Operator : SYSTEM   Seq. Line : 13
Acq. Instrument : LC1260   Location : P1-F-03
Injection Date : 7/9/2024 10:42:38 PM   Inj : 1
Inj Volume : 5.000 µl
Acq. Method : C:\Users\Public\Documents\ChemStation\1\Data\SUN\SUN 2024-07-09 19-30-21\IBN3-20-20.M
Last changed : 7/9/2024 10:40:48 PM by SYSTEM
Analysis Method : C:\Users\Public\Documents\ChemStation\1\Data\SUN\SUN 2024-07-09 19-30-21\IBN3-20-20.M (Sequence Method)
Last changed : 7/10/2024 9:48:00 AM by SYSTEM
(modified after loading)
Additional Info : Peak(s) manually integrated

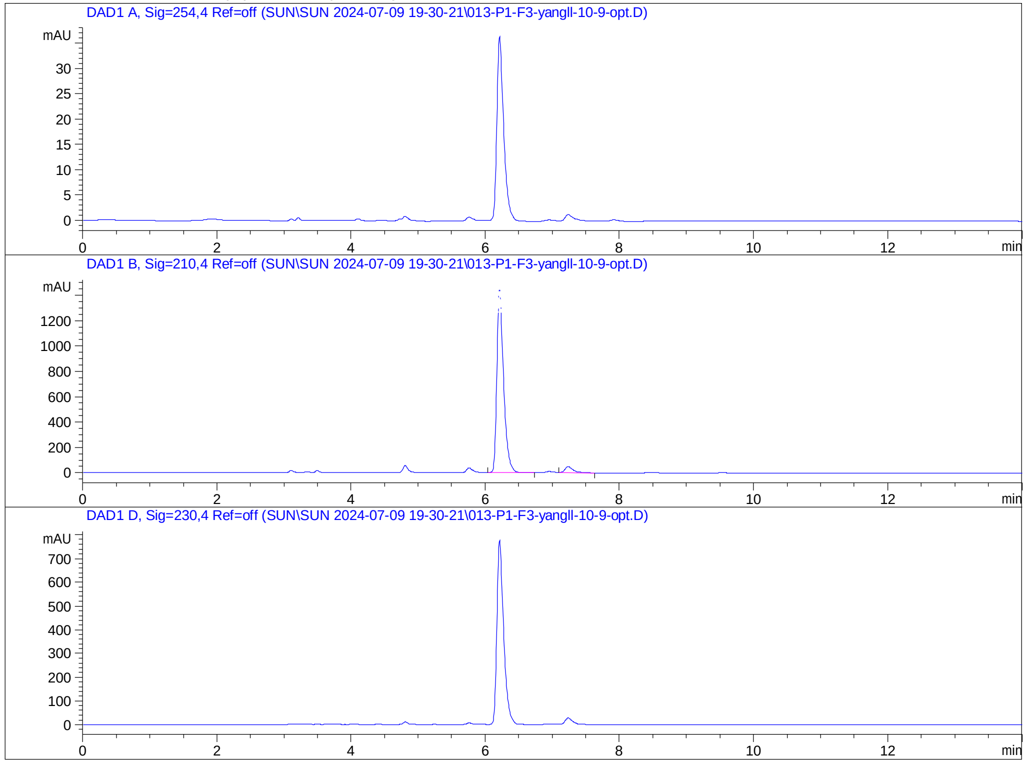

===
Area Percent Report
===

Sorted By : Signal
Multiplier : 1.0000
Dilution : 1.0000
Use Multiplier & Dilution Factor with ISTDs

Data File C:\Users\P...tion\1\Data\SUN\SUN 2024-07-09 19-30-21\013-P1-F3-yangll-10-9-opt.D
Sample Name: yangll-10-9-opt

Signal 1: DAD1 A, Sig=254,4 Ref=off

Signal 2: DAD1 B, Sig=210,4 Ref=off

Peak #	RetTime [min]	Type	Width [min]	Area [mAU*s]	Height [mAU]	Area %
1	6.215	VB	0.1056	1.01727e4	1454.76379	96.0337
2	7.236	VB	0.1253	420.14096	50.35328	3.9663

Totals : 1.05929e4 1505.11707

Signal 3: DAD1 D, Sig=230,4 Ref=off

===
*** End of Report ***

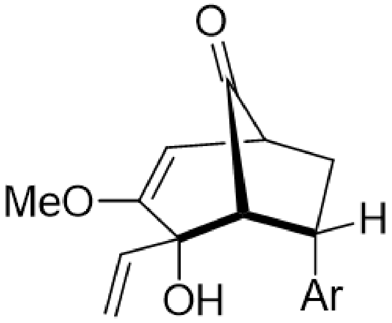

Ar = $4\text{-}CH_3C_6H_4$

10

opt

Data File C:\Users\P...\1\Data\SUN\SUN 2024-06-13 21-33-37\002-P1-F1-yangll-9-177-rac-OD.D
Sample Name: yangll-9-177-rac-OD

===

Acq. Operator : SYSTEM Seq. Line : 2
Acq. Instrument : LC1260 Location : P1-F-01
Injection Date : 6/13/2024 9:44:29 PM Inj : 1
Inj Volume : 5.000 µl
Different Inj Volume from Sample Entry! Actual Inj Volume : 20.000 µl
Acq. Method : C:\Users\Public\Documents\ChemStation\1\Data\SUN\SUN 2024-06-13 21-33-37\OD3-50-30.M
Last changed : 6/13/2024 9:24:07 PM by SYSTEM
Analysis Method : C:\Users\Public\Documents\ChemStation\1\Data\SUN\SUN 2024-06-13 21-33-37\OD3-50-30.M (Sequence Method)
Last changed : 6/25/2024 6:05:43 PM by SYSTEM
(modified after loading)
Additional Info : Peak(s) manually integrated

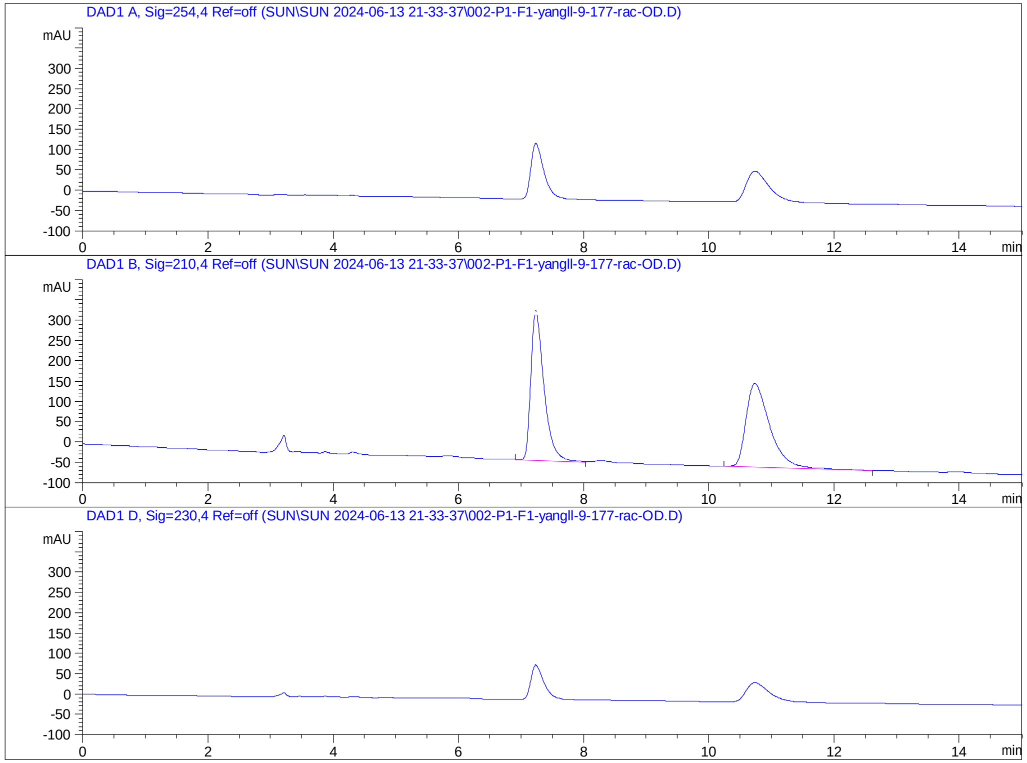

===

Area Percent Report

===

Sorted By : Signal
Multiplier : 1.0000
Dilution : 1.0000
Use Multiplier & Dilution Factor with ISTDs

Data File C:\Users\P...\1\Data\SUN\SUN 2024-06-13 21-33-37\002-P1-F1-yangll-9-177-rac-OD.D
Sample Name: yangll-9-177-rac-OD

Signal 1: DAD1 A, Sig=254,4 Ref=off

Signal 2: DAD1 B, Sig=210,4 Ref=off

Peak #	RetTime [min]	Type	Width [min]	Area [mAU*s]	Height [mAU]	Area %
1	7.238	MM R	0.2450	5419.68799	368.63217	49.9453
2	10.737	BB	0.4011	5431.55273	206.35652	50.0547

Totals : 1.08512e4 574.98869

Signal 3: DAD1 D, Sig=230,4 Ref=off

===

*** End of Report ***

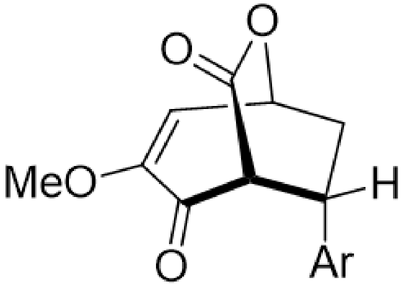

Ar = 4-$CH_3C_6H_4$

11

rac

===

Acq. Operator : SYSTEM   Seq. Line : 4
Acq. Instrument : LC1260   Location : P1-F-02
Injection Date : 6/25/2024 3:25:06 PM   Inj : 1
Inj Volume : 5.000 µl
Acq. Method : C:\Users\Public\Documents\ChemStation\1\Data\SUN\SUN 2024-06-25 14-30-19 \OD3-50-20.M
Last changed : 6/13/2024 9:24:25 PM by SYSTEM
Analysis Method : C:\Users\Public\Documents\ChemStation\1\Data\SUN\SUN 2024-06-25 14-30-19 \OD3-50-20.M (Sequence Method)
Last changed : 6/25/2024 5:52:22 PM by SYSTEM
(modified after loading)
Additional Info : Peak(s) manually integrated

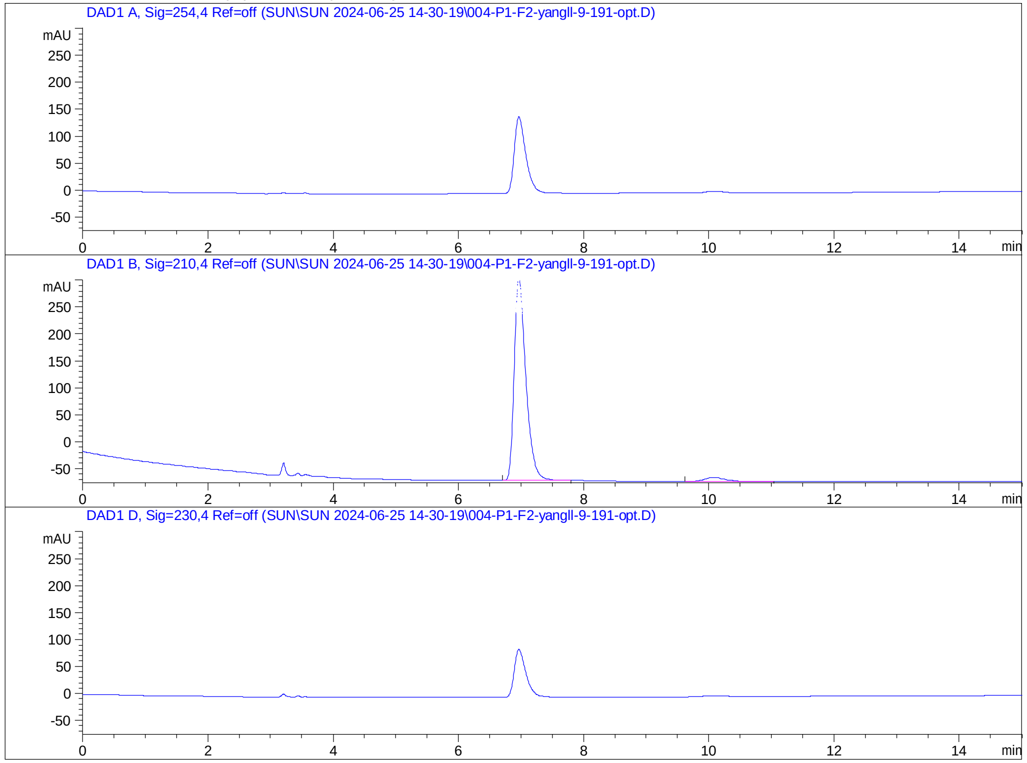

===

Area Percent Report

===

Sorted By : Signal
Multiplier : 1.0000
Dilution : 1.0000
Use Multiplier & Dilution Factor with ISTDs

Data File C:\Users\P...ion\1\Data\SUN\SUN 2024-06-25 14-30-19\004-P1-F2-yangll-9-191-opt.D
Sample Name: yangll-9-191-opt

Signal 1: DAD1 A, Sig=254,4 Ref=off

Signal 2: DAD1 B, Sig=210,4 Ref=off

Peak #	RetTime [min]	Type	Width [min]	Area [mAU*s]	Height [mAU]	Area %
1	6.964	BB	0.1969	4913.16699	379.75684	96.9137
2	10.077	BB	0.3332	156.46350	7.15283	3.0863

Totals : 5069.63049 386.90966

Signal 3: DAD1 D, Sig=230,4 Ref=off

===

*** End of Report ***

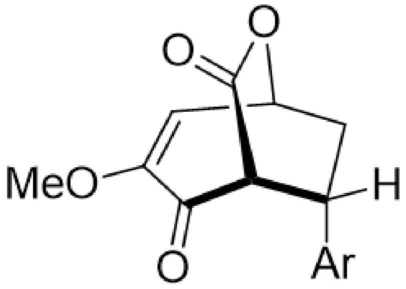

Ar = 4-$CH_3C_6H_4$

11

opt

```
=====================================================================
Acq. Operator   : SYSTEM                        Seq. Line :    8
Acq. Instrument : LC1260                         Location :    P1-F-02
Injection Date  : 6/27/2024 12:03:47 AM               Inj :    1
                                               Inj Volume : 5.000 µl
Method          : C:\Users\Public\Documents\ChemStation\1\Data\SUN\SUN 2024-06-26 21-25-02
                  \ID3-30-30.M (Sequence Method)
Last changed    : 6/26/2024 11:09:41 PM by SYSTEM
Additional Info : Peak(s) manually integrated
```

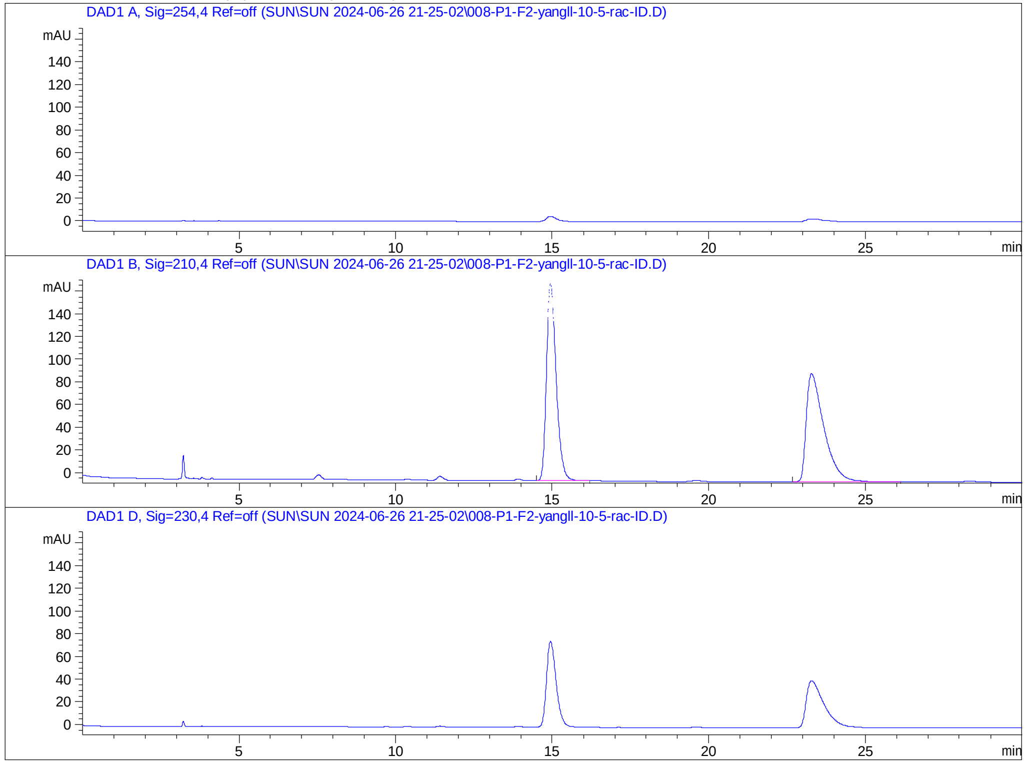


```
=====================================================================
                         Area Percent Report
=====================================================================

Sorted By             :      Signal
Multiplier            :      1.0000
Dilution              :      1.0000
Use Multiplier & Dilution Factor with ISTDs
```


Data File C:\Users\P...n\1\Data\SUN\SUN 2024-06-26 21-25-02\008-P1-F2-yangll-10-5-rac-ID.D
Sample Name: yangll-10-5-rac-ID

Signal 1: DAD1 A, Sig=254,4 Ref=off

Signal 2: DAD1 B, Sig=210,4 Ref=off

Peak #	RetTime [min]	Type	Width [min]	Area [mAU*s]	Height [mAU]	Area %
1	14.948	BB	0.3320	3756.46924	173.87833	49.9821
2	23.284	BB	0.5881	3759.16260	95.32287	50.0179

Totals : 7515.63184 269.20119

Signal 3: DAD1 D, Sig=230,4 Ref=off

```
=====================================================================
                          *** End of Report ***
```

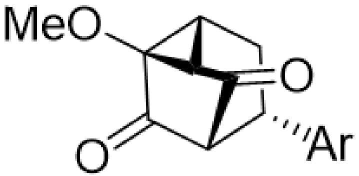

Ar = 4-$CH_3C_6H_4$

12

rac

===

Acq. Operator : SYSTEM   Seq. Line : 5
Acq. Instrument : LC1260   Location : P1-F-04
Injection Date : 6/28/2024 12:20:45 PM   Inj : 1
Inj Volume : 5.000 µl
Acq. Method : C:\Users\Public\Documents\ChemStation\1\Data\SUN\SUN 2024-06-28 10-51-24 \ID3-30-30.M
Last changed : 6/26/2024 11:09:41 PM by SYSTEM
Analysis Method : C:\Users\Public\Documents\ChemStation\1\Data\SUN\SUN 2024-06-28 10-51-24 \ID3-30-30.M (Sequence Method)
Last changed : 6/28/2024 2:51:25 PM by SYSTEM
(modified after loading)
Additional Info : Peak(s) manually integrated

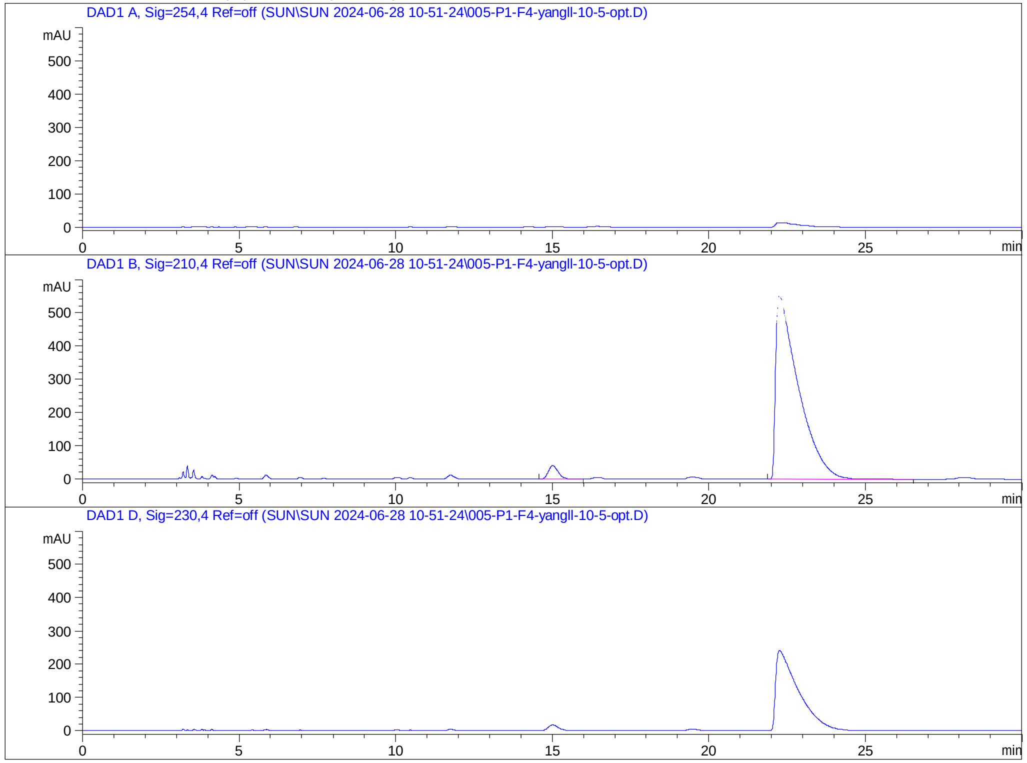

===
Area Percent Report
===

Sorted By : Signal
Multiplier : 1.0000
Dilution : 1.0000
Use Multiplier & Dilution Factor with ISTDs

Data File C:\Users\P...tion\1\Data\SUN\SUN 2024-06-28 10-51-24\005-P1-F4-yangll-10-5-opt.D
Sample Name: yangll-10-5-opt

Signal 1: DAD1 A, Sig=254,4 Ref=off

Signal 2: DAD1 B, Sig=210,4 Ref=off

Peak #	RetTime [min]	Type	Width [min]	Area [mAU*s]	Height [mAU]	Area %
1	15.017	BB	0.3424	883.79993	39.91051	3.0868
2	22.268	BB	0.7036	2.77479e4	552.82501	96.9132

Totals : 2.86317e4 592.73553

Signal 3: DAD1 D, Sig=230,4 Ref=off

===
*** End of Report ***

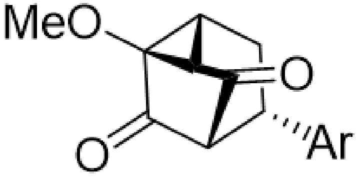

Ar = 4-$CH_3C_6H_4$

12

opt

===

Acq. Operator : SYSTEM Seq. Line : 5
Acq. Instrument : LC1260 Location : P1-F-01
Injection Date : 6/26/2024 11:00:49 PM Inj : 1
Inj Volume : 5.000 µl
Method : C:\Users\Public\Documents\ChemStation\1\Data\SUN\SUN 2024-06-26 21-25-02\IBN3-20-20.M (Sequence Method)
Last changed : 5/13/2024 2:34:23 PM by SYSTEM
Additional Info : Peak(s) manually integrated

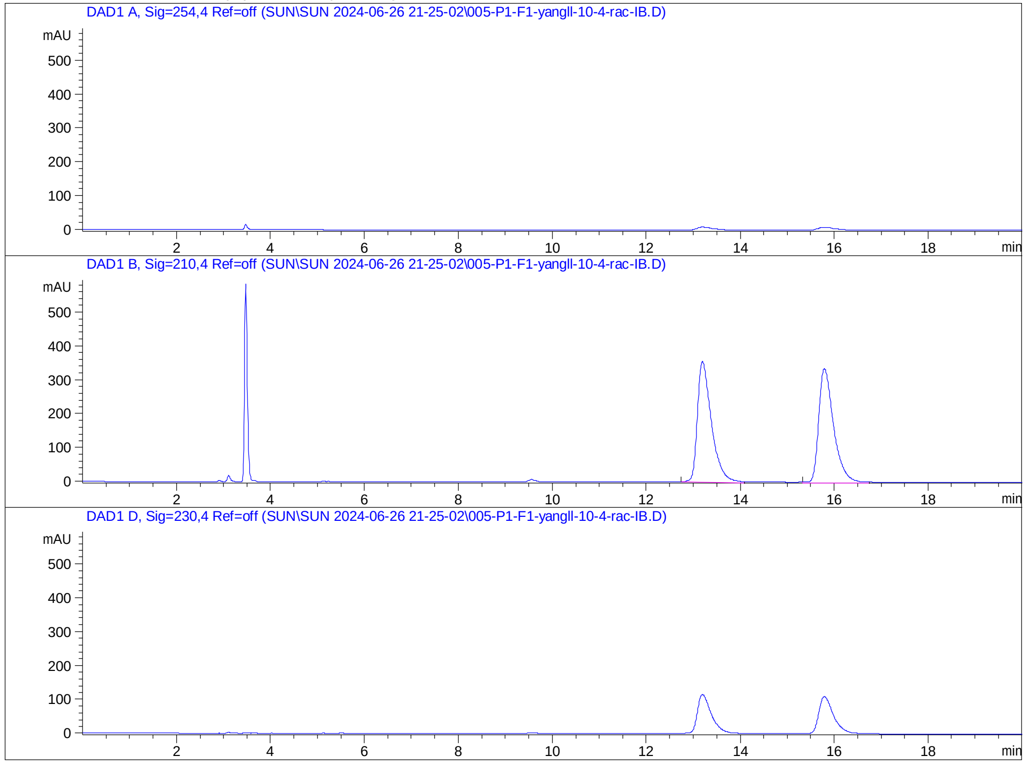

===

Area Percent Report

===

Sorted By : Signal
Multiplier : 1.0000
Dilution : 1.0000
Use Multiplier & Dilution Factor with ISTDs

Signal 1: DAD1 A, Sig=254,4 Ref=off

Signal 2: DAD1 B, Sig=210,4 Ref=off

Peak #	RetTime [min]	Type	Width [min]	Area [mAU*s]	Height [mAU]	Area %
1	13.199	MM R	0.3483	7452.50146	356.59360	49.8916
2	15.794	MM R	0.3699	7484.89160	337.28671	50.1084

Totals : 1.49374e4 693.88031

Signal 3: DAD1 D, Sig=230,4 Ref=off

===

*** End of Report ***

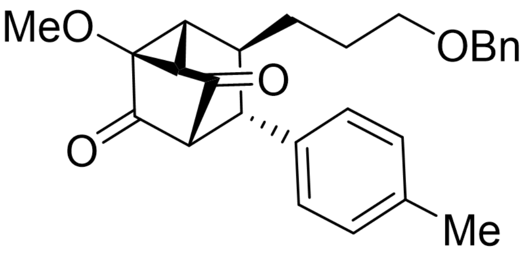

13

rac

Data File C:\Users\P...tion\1\Data\SUN\SUN 2024-06-28 10-51-24\002-P1-F3-yangll-10-4-opt.D
Sample Name: yangll-10-4-opt

===

Acq. Operator : SYSTEM Seq. Line : 2
Acq. Instrument : LC1260 Location : P1-F-03
Injection Date : 6/28/2024 11:07:41 AM Inj : 1
Inj Volume : 5.000 µl
Different Inj Volume from Sample Entry! Actual Inj Volume : 2.000 µl
Acq. Method : C:\Users\Public\Documents\ChemStation\1\Data\SUN\SUN 2024-06-28 10-51-24\IBN3-20-30.M
Last changed : 6/25/2024 2:29:39 PM by SYSTEM
Analysis Method : C:\Users\Public\Documents\ChemStation\1\Data\SUN\SUN 2024-06-28 10-51-24\IBN3-20-30.M (Sequence Method)
Last changed : 6/28/2024 12:14:52 PM by SYSTEM
(modified after loading)
Additional Info : Peak(s) manually integrated

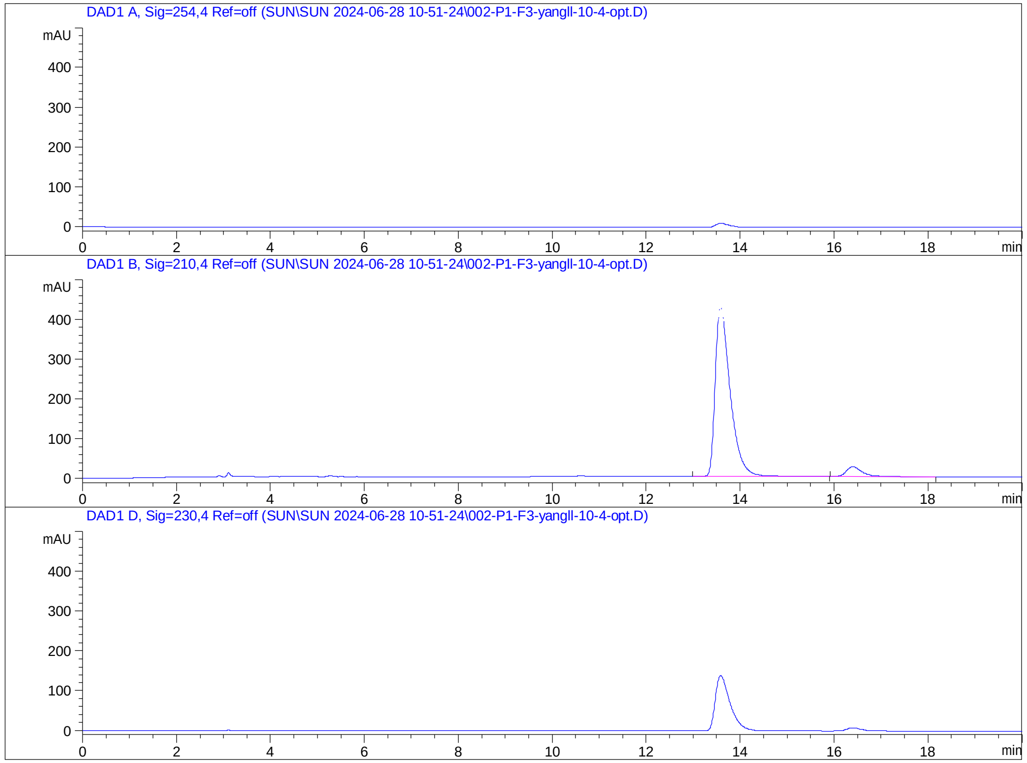

===

Area Percent Report

===

Sorted By : Signal
Multiplier : 1.0000
Dilution : 1.0000
Use Multiplier & Dilution Factor with ISTDs

Data File C:\Users\P...tion\1\Data\SUN\SUN 2024-06-28 10-51-24\002-P1-F3-yangll-10-4-opt.D
Sample Name: yangll-10-4-opt

Signal 1: DAD1 A, Sig=254,4 Ref=off

Signal 2: DAD1 B, Sig=210,4 Ref=off

Peak #	RetTime [min]	Type	Width [min]	Area [mAU*s]	Height [mAU]	Area %
1	13.586	BB	0.3305	9401.77734	427.62296	94.2741
2	16.403	BB	0.3471	571.03033	24.75761	5.7259

Totals : 9972.80768 452.38056

Signal 3: DAD1 D, Sig=230,4 Ref=off

===

*** End of Report ***

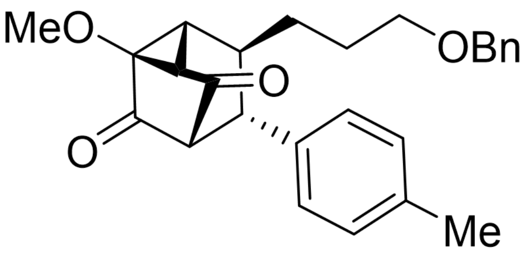

13

opt

Data File C:\Users\P...n\1\Data\SUN\SUN 2024-12-06 14-06-39\006-P1-A1-yangll-11-4-rac-IC.D
Sample Name: yangll-11-4-rac-IC

===

Acq. Operator : SYSTEM Seq. Line : 6
Acq. Instrument : LC1260 Location : P1-A-01
Injection Date : 12/6/2024 3:53:31 PM Inj : 1
Inj Volume : 5.000 µl
Different Inj Volume from Sample Entry! Actual Inj Volume : 10.000 µl
Acq. Method : C:\Users\Public\Documents\ChemStation\1\Data\SUN\SUN 2024-12-06 14-06-39\IC3-30-50.M
Last changed : 4/17/2024 2:33:10 PM by SYSTEM
Analysis Method : C:\Users\Public\Documents\ChemStation\1\Data\SUN\SUN 2024-12-06 14-06-39\IC3-30-50.M (Sequence Method)
Last changed : 12/6/2024 6:05:27 PM by SYSTEM
(modified after loading)
Additional Info : Peak(s) manually integrated

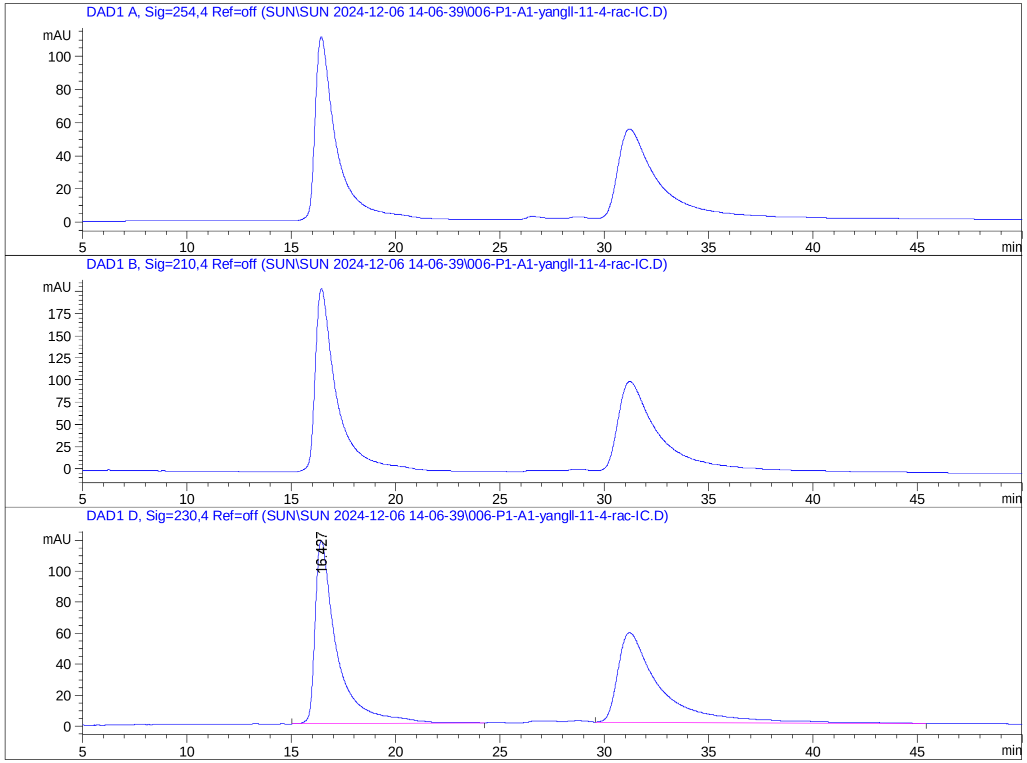

===

Area Percent Report

===

Sorted By : Signal
Multiplier : 1.0000
Dilution : 1.0000
Use Multiplier & Dilution Factor with ISTDs

Data File C:\Users\P...n\1\Data\SUN\SUN 2024-12-06 14-06-39\006-P1-A1-yangll-11-4-rac-IC.D
Sample Name: yangll-11-4-rac-IC

Signal 1: DAD1 A, Sig=254,4 Ref=off

Signal 2: DAD1 B, Sig=210,4 Ref=off

Signal 3: DAD1 D, Sig=230,4 Ref=off

Peak #	RetTime [min]	Type	Width [min]	Area [mAU*s]	Height [mAU]	Area %
1	16.427	BB	1.0375	8646.05371	118.19043	51.6875
2	31.204	BB	1.9835	8081.51318	57.72066	48.3125

Totals : 1.67276e4 175.91109

===

*** End of Report ***

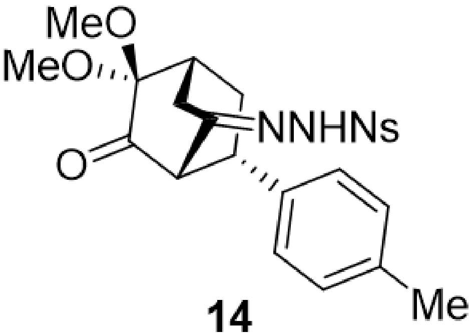

14

rac

Data File C:\Users\P...tion\1\Data\SUN\SUN 2024-12-06 14-06-39\007-P1-A2-yangll-11-4-opt.D
Sample Name: yangll-11-4-opt

===

```
Acq. Operator   : SYSTEM                         Seq. Line :    7
Acq. Instrument : LC1260                          Location :   P1-A-02
Injection Date  : 12/6/2024 4:44:32 PM                 Inj :    1
                                                Inj Volume : 5.000 µl
Different Inj Volume from Sample Entry!  Actual Inj Volume : 10.000 µl
Acq. Method     : C:\Users\Public\Documents\ChemStation\1\Data\SUN\SUN 2024-12-06 14-06-39
                  \IC3-30-50.M
Last changed    : 4/17/2024 2:33:10 PM by SYSTEM
Analysis Method : C:\Users\Public\Documents\ChemStation\1\Data\SUN\SUN 2024-12-06 14-06-39
                  \IC3-30-50.M (Sequence Method)
Last changed    : 12/6/2024 6:05:27 PM by SYSTEM
                  (modified after loading)
Additional Info : Peak(s) manually integrated
```

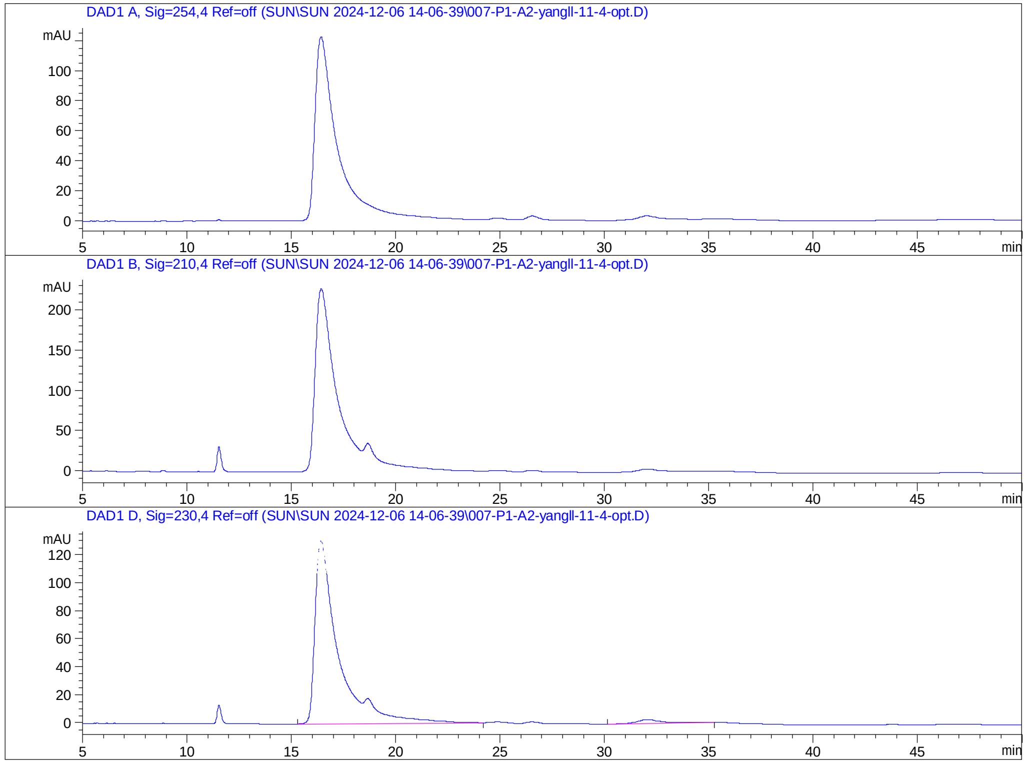

===

Area Percent Report

===

```
Sorted By             :      Signal
Multiplier            :      1.0000
Dilution              :      1.0000
Use Multiplier & Dilution Factor with ISTDs
```


Signal 1: DAD1 A, Sig=254,4 Ref=off

Peak #	RetTime [min]	Type	Width [min]	Area [mAU*s]	Height [mAU]	Area %
1	3.391	BV	0.1004	10.12429	1.43305	100.0000

Totals : 10.12429 1.43305

Signal 2: DAD1 B, Sig=210,4 Ref=off

Signal 3: DAD1 D, Sig=230,4 Ref=off

Peak #	RetTime [min]	Type	Width [min]	Area [mAU*s]	Height [mAU]	Area %
1	16.422	BB R	1.0479	1.02687e4	131.01762	97.5407
2	32.060	MM R	1.5129	258.90527	2.85216	2.4593

Totals : 1.05276e4 133.86978

===

*** End of Report ***

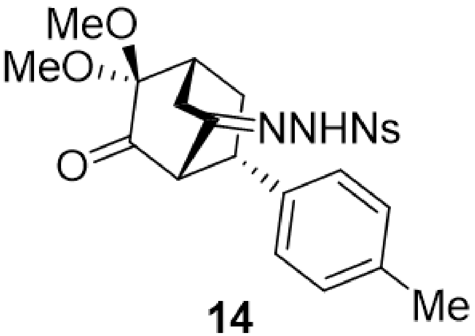

14

opt

===

Acq. Operator : SYSTEM Seq. Line : 2
Acq. Instrument : LC1260 Location : P1-A-01
Injection Date : 12/4/2024 3:24:35 PM Inj : 1
Inj Volume : 5.000 µl
Acq. Method : C:\Users\Public\Documents\ChemStation\1\Data\SUN\SUN 2024-12-04 15-11-46\IBN3-20-20.M
Last changed : 5/13/2024 2:34:23 PM by SYSTEM
Analysis Method : C:\Users\Public\Documents\ChemStation\1\Data\SUN\SUN 2024-12-04 15-11-46\IBN3-20-20.M (Sequence Method)
Last changed : 12/4/2024 6:32:33 PM by SYSTEM
(modified after loading)
Additional Info : Peak(s) manually integrated

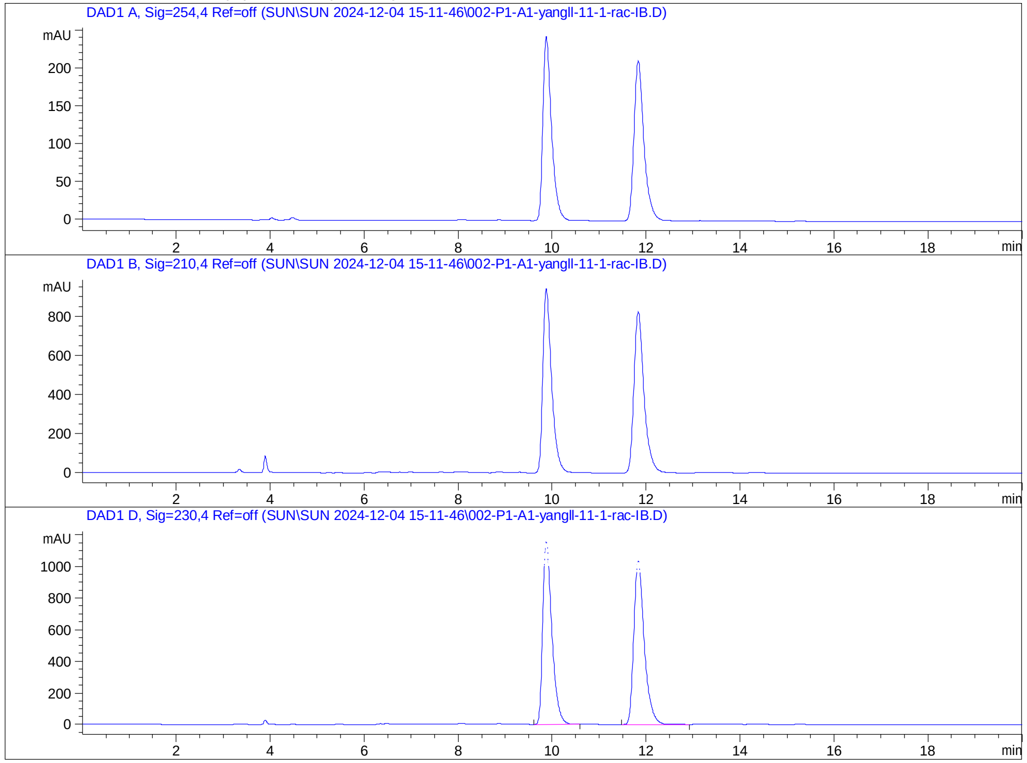

===
Area Percent Report
===

Sorted By : Signal
Multiplier : 1.0000
Dilution : 1.0000
Use Multiplier & Dilution Factor with ISTDs

Data File C:\Users\P...n\1\Data\SUN\SUN 2024-12-04 15-11-46\002-P1-A1-yangll-11-1-rac-IB.D
Sample Name: yangll-11-1-rac-IB

Signal 1: DAD1 A, Sig=254,4 Ref=off

Signal 2: DAD1 B, Sig=210,4 Ref=off

Signal 3: DAD1 D, Sig=230,4 Ref=off

Peak #	RetTime [min]	Type	Width [min]	Area [mAU*s]	Height [mAU]	Area %
1	9.877	BB	0.2060	1.57937e4	1166.19641	49.5338
2	11.836	BB	0.2350	1.60910e4	1037.38843	50.4662

Totals : 3.18848e4 2203.58484

===
*** End of Report ***

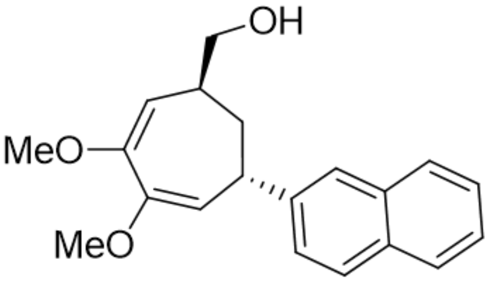

15

rac

===

Acq. Operator : SYSTEM Seq. Line : 1
Acq. Instrument : LC1260 Location : P1-A-01
Injection Date : 12/4/2024 5:52:37 PM Inj : 1
Inj Volume : 5.000 µl
Different Inj Volume from Sample Entry! Actual Inj Volume : 1.000 µl
Acq. Method : C:\Users\Public\Documents\ChemStation\1\Data\SUN\SUN 2024-12-04 17-50-49\IBN3-20-20.M
Last changed : 5/13/2024 2:34:23 PM by SYSTEM
Analysis Method : C:\Users\Public\Documents\ChemStation\1\Data\SUN\SUN 2024-12-04 17-50-49\IBN3-20-20.M (Sequence Method)
Last changed : 12/4/2024 6:30:09 PM by SYSTEM
(modified after loading)
Additional Info : Peak(s) manually integrated

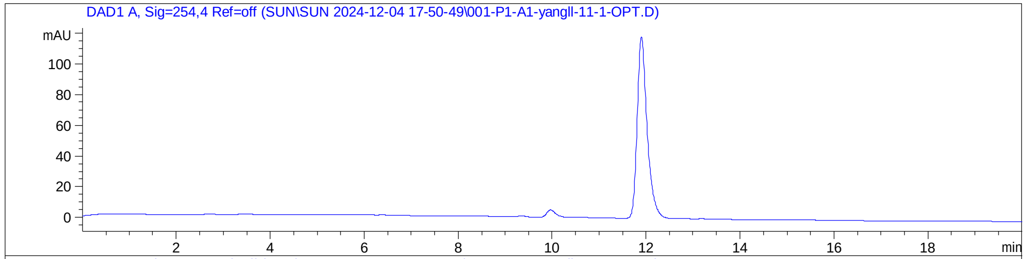

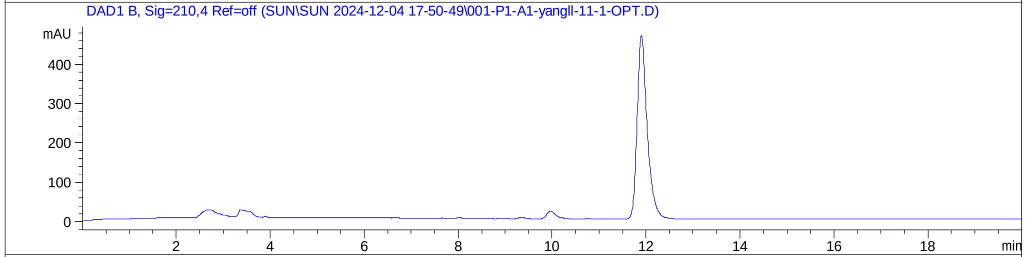

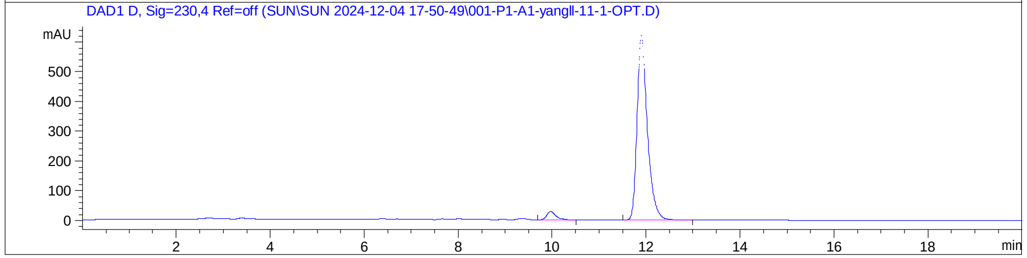

===
Area Percent Report
===

Sorted By : Signal
Multiplier : 1.0000
Dilution : 1.0000
Use Multiplier & Dilution Factor with ISTDs

Data File C:\Users\P...tion\1\Data\SUN\SUN 2024-12-04 17-50-49\001-P1-A1-yangll-11-1-OPT.D
Sample Name: yangll-11-1-OPT

Signal 1: DAD1 A, Sig=254,4 Ref=off

Signal 2: DAD1 B, Sig=210,4 Ref=off

Signal 3: DAD1 D, Sig=230,4 Ref=off

Peak #	RetTime [min]	Type	Width [min]	Area [mAU*s]	Height [mAU]	Area %
1	9.968	BB	0.1988	358.27451	27.36168	3.6600
2	11.903	BB	0.2296	9430.60156	619.64166	96.3400

Totals : 9788.87607 647.00335

===
*** End of Report ***

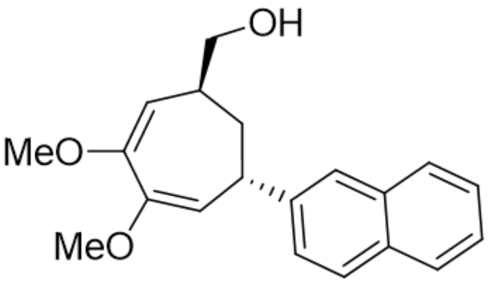

15

opt

===

Acq. Operator : SYSTEM Seq. Line : 2
Acq. Instrument : LC1260 Location : P1-F-01
Injection Date : 12/21/2024 1:09:51 PM Inj : 1
Inj Volume : 5.000 µl
Different Inj Volume from Sample Entry! Actual Inj Volume : 10.000 µl
Acq. Method : C:\Users\Public\Documents\ChemStation\1\Data\SUN\SUN 2024-12-21 12-57-01\IA3-10-40.M
Last changed : 12/21/2024 12:56:47 PM by SYSTEM
Analysis Method : C:\Users\Public\Documents\ChemStation\1\Data\SUN\SUN 2024-12-21 12-57-01\IA3-10-40.M (Sequence Method)
Last changed : 12/21/2024 3:20:50 PM by SYSTEM
(modified after loading)
Additional Info : Peak(s) manually integrated

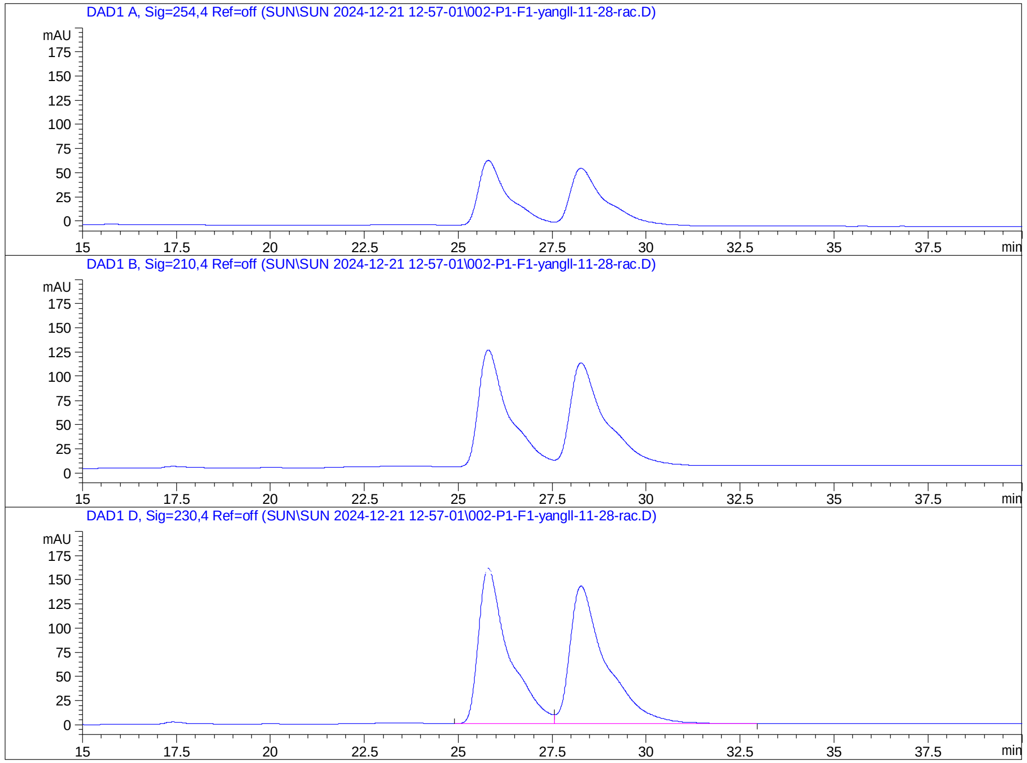

===
Area Percent Report
===

Sorted By : Signal
Multiplier : 1.0000
Dilution : 1.0000
Use Multiplier & Dilution Factor with ISTDs

Data File C:\Users\P...ion\1\Data\SUN\SUN 2024-12-21 12-57-01\002-P1-F1-yangll-11-28-rac.D
Sample Name: yangll-11-28-rac

Signal 1: DAD1 A, Sig=254,4 Ref=off

Signal 2: DAD1 B, Sig=210,4 Ref=off

Signal 3: DAD1 D, Sig=230,4 Ref=off

Peak #	RetTime [min]	Type	Width [min]	Area [mAU*s]	Height [mAU]	Area %
1	25.796	BV	0.8087	9087.35352	160.95575	48.9031
2	28.265	VB	0.9499	9495.02832	142.39746	51.0969

Totals : 1.85824e4 303.35321

===
*** End of Report ***

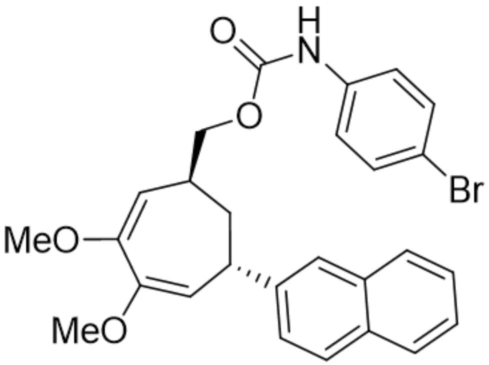

16

rac

===

```
Acq. Operator   : SYSTEM                        Seq. Line :    3
Acq. Instrument : LC1260                         Location :   P1-F-02
Injection Date  : 12/21/2024 1:50:55 PM               Inj :    1
                                               Inj Volume : 5.000 µl
Different Inj Volume from Sample Entry!  Actual Inj Volume : 20.000 µl
Acq. Method     : C:\Users\Public\Documents\ChemStation\1\Data\SUN\SUN 2024-12-21 12-57-01
                  \IA3-10-40.M
Last changed    : 12/21/2024 12:56:47 PM by SYSTEM
Analysis Method : C:\Users\Public\Documents\ChemStation\1\Data\SUN\SUN 2024-12-21 12-57-01
                  \IA3-10-40.M (Sequence Method)
Last changed    : 12/21/2024 3:19:49 PM by SYSTEM
                  (modified after loading)
Additional Info : Peak(s) manually integrated
```

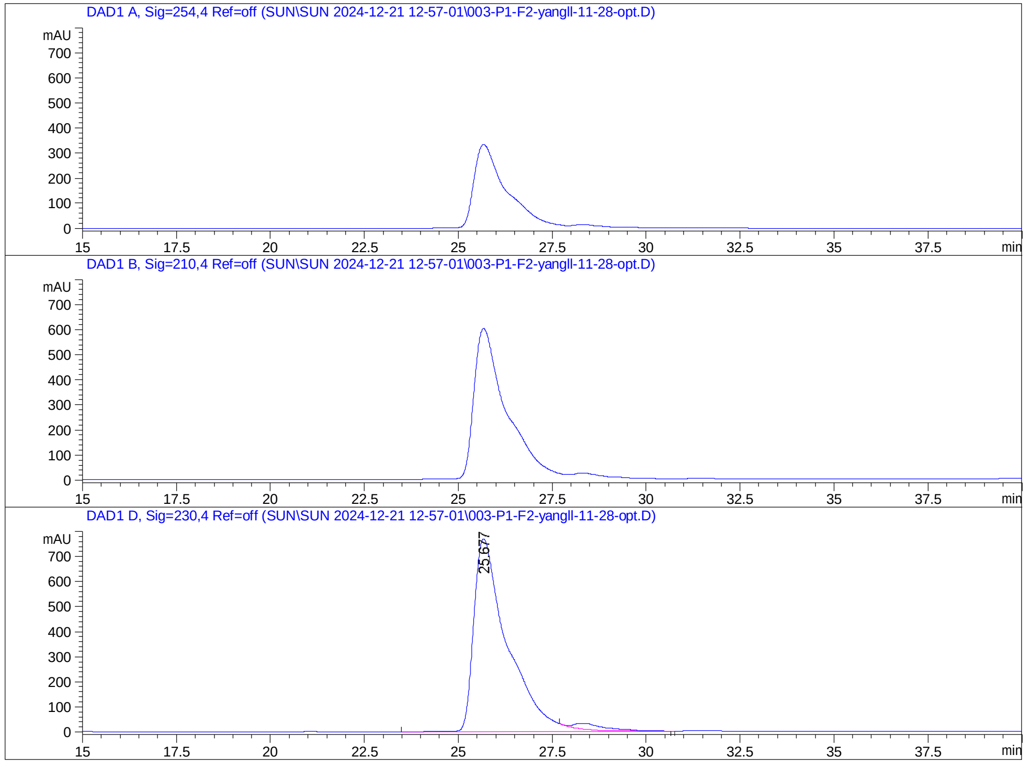

===

Area Percent Report

===

```
Sorted By             :      Signal
Multiplier            :      1.0000
Dilution              :      1.0000
Use Multiplier & Dilution Factor with ISTDs
```


Data File C:\Users\P...ion\1\Data\SUN\SUN 2024-12-21 12-57-01\003-P1-F2-yangll-11-28-opt.D
Sample Name: yangll-11-28-opt

Signal 1: DAD1 A, Sig=254,4 Ref=off

Signal 2: DAD1 B, Sig=210,4 Ref=off

Signal 3: DAD1 D, Sig=230,4 Ref=off

Peak #	RetTime [min]	Type	Width [min]	Area [mAU*s]	Height [mAU]	Area %
1	25.677	BB R	0.8906	4.78772e4	768.72290	97.0895
2	28.316	VB E	0.8992	1435.23254	22.28879	2.9105

Totals : 4.93125e4 791.01169

===

*** End of Report ***

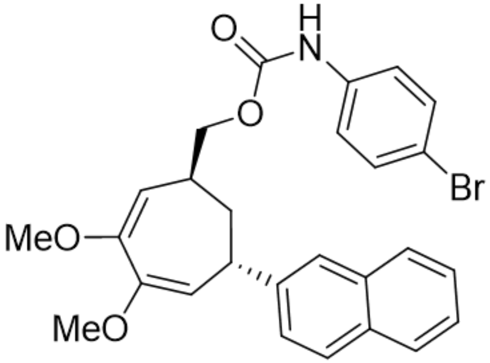

16

opt

===

Acq. Operator : SYSTEM   Seq. Line : 2
Acq. Instrument : LC1260   Location : P1-A-01
Injection Date : 5/30/2024 9:26:29 PM   Inj : 1
Inj Volume : 5.000 µl
Different Inj Volume from Sample Entry! Actual Inj Volume : 1.000 µl
Acq. Method : C:\Users\Public\Documents\ChemStation\1\Data\SUN\SUN 2024-05-30 21-13-41\IN3-10-20.M
Last changed : 5/20/2024 1:11:23 PM by SYSTEM
Analysis Method : C:\Users\Public\Documents\ChemStation\1\Data\SUN\SUN 2024-05-30 21-13-41\IN3-10-20.M (Sequence Method)
Last changed : 12/18/2024 3:31:32 PM by SYSTEM
(modified after loading)
Additional Info : Peak(s) manually integrated

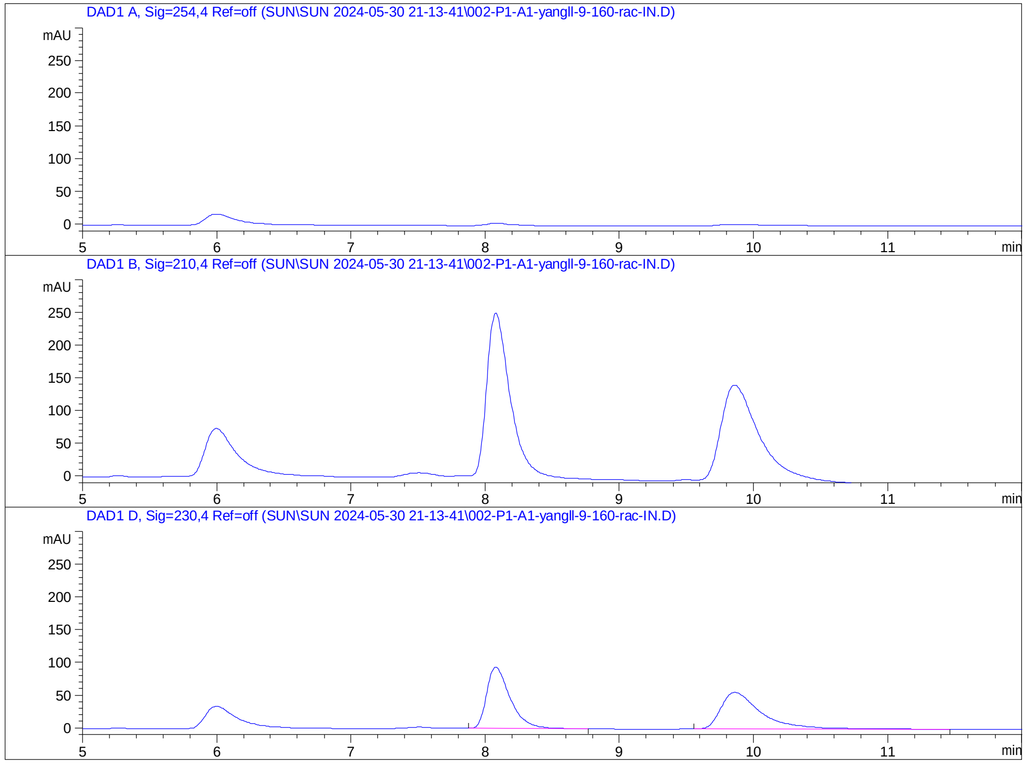

===

Area Percent Report

===

Sorted By : Signal
Multiplier : 1.0000
Dilution : 1.0000
Use Multiplier & Dilution Factor with ISTDs

Data File C:\Users\P...\1\Data\SUN\SUN 2024-05-30 21-13-41\002-P1-A1-yangll-9-160-rac-IN.D
Sample Name: yangll-9-160-rac-IN

Signal 1: DAD1 A, Sig=254,4 Ref=off

Signal 2: DAD1 B, Sig=210,4 Ref=off

Signal 3: DAD1 D, Sig=230,4 Ref=off

Peak #	RetTime [min]	Type	Width [min]	Area [mAU*s]	Height [mAU]	Area %
1	8.082	BB	0.1834	1137.42017	93.81633	49.8045
2	9.863	BB	0.3062	1146.34900	55.67275	50.1955

Totals : 2283.76917 149.48908

===

*** End of Report ***

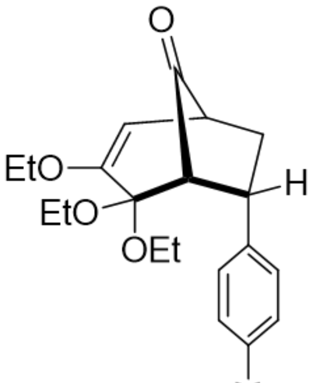

4bb

rac

===

```
Acq. Operator   : SYSTEM                        Seq. Line :   6
Acq. Instrument : LC1260                         Location :   P1-F-03
Injection Date  : 7/6/2024 3:22:17 PM                 Inj :   1
                                               Inj Volume : 5.000 µl
Acq. Method     : C:\Users\Public\Documents\ChemStation\1\Data\SUN\SUN 2024-07-06 13-15-30
                  \IN3-10-20.M
Last changed    : 5/20/2024 1:11:23 PM by SYSTEM
Analysis Method : C:\Users\Public\Documents\ChemStation\1\Data\SUN\SUN 2024-07-06 13-15-30
                  \IN3-10-20.M (Sequence Method)
Last changed    : 11/25/2024 7:48:43 PM by SYSTEM
                  (modified after loading)
Additional Info : Peak(s) manually integrated
```

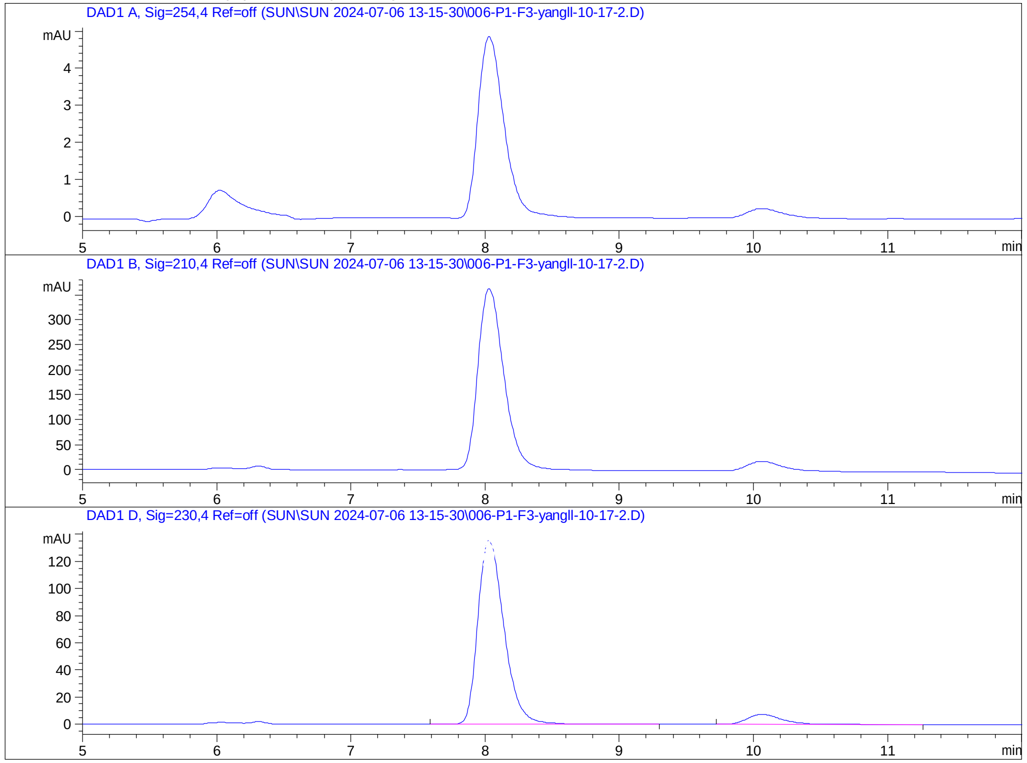

===
Area Percent Report
===

```
Sorted By             :      Signal
Multiplier            :      1.0000
Dilution              :      1.0000
Use Multiplier & Dilution Factor with ISTDs
```


Data File C:\Users\P...ation\1\Data\SUN\SUN 2024-07-06 13-15-30\006-P1-F3-yangll-10-17-2.D
Sample Name: yangll-10-17-2

Signal 1: DAD1 A, Sig=254,4 Ref=off

Signal 2: DAD1 B, Sig=210,4 Ref=off

Signal 3: DAD1 D, Sig=230,4 Ref=off

Peak #	RetTime [min]	Type	Width [min]	Area [mAU*s]	Height [mAU]	Area %
1	8.027	BB	0.2127	1847.85571	135.80112	93.0938
2	10.060	BB	0.2789	137.08437	7.51118	6.9062

Totals : 1984.94008 143.31230

===
*** End of Report ***

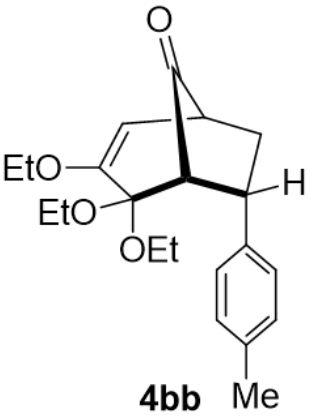

4bb

opt (100 mol% **3i**)
